# High-Throughput Chemical Screening and Structure-Based Models to Predict hERG Inhibition

**DOI:** 10.3390/biology11020209

**Published:** 2022-01-28

**Authors:** Shagun Krishna, Alexandre Borrel, Ruili Huang, Jinghua Zhao, Menghang Xia, Nicole Kleinstreuer

**Affiliations:** 1 Division of the National Toxicology Program, National Institute of Environmental Health Sciences (NIEHS), Research Triangle, NC 27560, USA; shagun.krishna@nih.gov; 2 Silent Spring Institute, Newton, MA 02460, USA; borrel@silentspring.org; 3 Division of Preclinical Innovation, National Center for Advancing Translational Sciences (NCATS), Bethesda, MD 20892-4874, USA; ruili.huang@nih.gov (R.H.); jinghua.zhao@nih.gov (J.Z.); mxia@mail.nih.gov (M.X.)

**Keywords:** cardiovascular, hERG, Tox21 high-throughput screening, environmental chemicals, in silico modeling, QSAR models

## Abstract

**Simple Summary:**

Cardiovascular disease is the leading cause of death for people of most ethnicities in the United States. The human ether-a-go-go-related gene (hERG) potassium channel plays a pivotal role in cardiac rhythm regulation, and cardiotoxicity associated with hERG inhibition by drug molecules and environmental chemicals is a major public health concern. An evaluation of the effect of environmental chemicals on hERG channel function can help inform the potential public health risks of these compounds. To assess the cardiotoxic effect of diverse drugs and environmental compounds, the Tox21 federal research program has screened a collection of 9667 chemicals for inhibitory activity against the hERG channel. A set of molecular descriptors covering physicochemical and structural properties of chemicals, self-organizing maps, and hierarchical clustering were applied to characterize the chemicals inhibiting hERG. Machine learning approaches were applied to build robust statistical models that can predict the probability of any new chemical to cause cardiotoxicity via this mechanism.

**Abstract:**

Chemical inhibition of the human ether-a -go-go-related gene (hERG) potassium channel leads to a prolonged QT interval that can contribute to severe cardiotoxicity. The adverse effects of hERG inhibition are one of the principal causes of drug attrition in clinical and pre-clinical development. Preliminary studies have demonstrated that a wide range of environmental chemicals and toxicants may also inhibit the hERG channel and contribute to the pathophysiology of cardiovascular (CV) diseases. As part of the US federal Tox21 program, the National Center for Advancing Translational Science (NCATS) applied a quantitative high throughput screening (qHTS) approach to screen the Tox21 library of 10,000 compounds (~7871 unique chemicals) at 14 concentrations in triplicate to identify chemicals perturbing hERG activity in the U2OS cell line thallium flux assay platform. The qHTS cell-based thallium influx assay provided a robust and reliable dataset to evaluate the ability of thousands of drugs and environmental chemicals to inhibit hERG channel protein, and the use of chemical structure-based clustering and chemotype enrichment analysis facilitated the identification of molecular features that are likely responsible for the observed hERG activity. We employed several machine-learning approaches to develop QSAR prediction models for the assessment of hERG liabilities for drug-like and environmental chemicals. The training set was compiled by integrating hERG bioactivity data from the ChEMBL database with the Tox21 qHTS thallium flux assay data. The best results were obtained with the random forest method (~92.6% balanced accuracy). The data and scripts used to generate hERG prediction models are provided in an open-access format as key in vitro and in silico tools that can be applied in a translational toxicology pipeline for drug development and environmental chemical screening.

## 1. Introduction

The human ether-a-go-go-related gene (hERG) potassium channel, a member of the family of voltage-gated potassium channels (KCNH2), plays a pivotal role in cardiac rhythm regulation, especially in the repolarization of the cardiac action potential. Inhibition of hERG can lead to a prolongation of the QT interval, which, in the worst case, triggers torsade de pointes arrhythmia, which progresses to ventricular fibrillation and sudden death [1,2,3].

In the drug discovery field, predicting hERG inhibition is a pressing issue since many low molecular weight drug-like molecules are reported to have varying levels of inhibitory potential [4,5]. In addition, many drugs have been withdrawn from the market due to cardiotoxicity via high affinity to, and inhibition of, the hERG channel [6]. The regulatory basis for safety pharmacology studies is defined by the international conference on harmonization (ICH) guidelines, e.g., the non-clinical guideline S7B [7], and the first cardiotoxicity guideline for drugs was adopted in 2005 [8]. Today, testing compounds with off targets, such as the hERG channel, during early drug development has become routine [9], and the FDA requires almost all new low molecular weight drugs to be assessed in a “thorough QT” clinical study to determine their potential to prolong the heart-rate-corrected QT interval [10]. However, most screening methods use animal or ex vivo modeling, which is distinctly low-throughput, costly, requires relatively large quantities of chemicals, and presents both ethical and species extrapolation issues. Over the last 20 years, a large number of in silico methods have been developed [11,12], and the FDA recently launched a major initiative via the Comprehensive In Vitro Pro-Arrhythmia Assay (CiPA) program to join such in silico models with in vitro data from engineered human cells and stem cell-derived cardiomyocytes to provide faster and cheaper approaches to estimate cardiotoxicity in early drug development steps [13]. 

Drug-like molecules are not unique in their ability to interact with the hERG target and produce cardiotoxic effects. It has been reported that some natural products are also able to inhibit hERG [9], as well as environmental chemicals such as quaternary ammonium compounds [14]. In fact, a wide range of environmental toxicants may have the potential to contribute to the pathophysiology of CV diseases [15], but the underlying mechanisms and consequences of real-world exposures require further investigation. To date, more than 100,000 chemicals have been introduced into commerce without substantive, or any, toxicological testing [16]. These synthetic chemicals are widely used in transportation, manufacturing, agriculture, food, and pharmaceutical industries, and may cause environmental pollution and human exposure via contamination of air, soil, water, and food. An evaluation of the effect of environmental chemicals on hERG channel function can provide critical information regarding the potential CV risks of these compounds on public health.

To meet the needs of toxicity testing in the 21st century, the US federal Tox21 research program was established. This partnership, between the Division of the National Toxicology Program (NTP) at the National Institute of Environmental Health Sciences, the Environmental Protection Agency (EPA), the National Center for Advancing Translational Science (NCATS), and the Food and Drug Administration (FDA), focuses on driving the evolution of toxicology by developing methods to rapidly and efficiently evaluate the safety of commercial chemicals, pesticides, food additives/contaminants, personal care product ingredients, and medical products. The goals of Tox21 are to identify the mechanisms of compound action at the molecular and cellular level, prioritize chemicals for further toxicological evaluation, and develop useful predictive models of in vivo biological responses [14,17,18]. 

To assess the effect of environmental chemicals on hERG channels as part of the Tox21 program, NCATS has screened the Tox21 10K chemical library, a collection of 9667 chemicals representing 7871 unique structures [19], using a cell-based thallium influx assay in a quantitative high-throughput screening (qHTS) format [14]. Here, we present the results of this screening effort, and, utilizing a set of molecular descriptors covering physicochemical and 1D and 2D chemical properties, we applied Self-Organizing Maps (SOM) and hierarchical clustering to characterize chemicals inhibiting hERG. Machine learning and deep learning approaches were applied to build statistical quantitative structure-activity relationship (QSAR) models to predict the probability of a chemical inhibiting hERG in this thallium flux assay. This tiered clustering and predictive modeling approach will assist in the detection of environmental chemicals that merit more extensive evaluation for cardiotoxicity and provides potentially useful structural information for predicting the ability of new chemical entities to induce hERG inhibition.

## 2. Materials and Methods

### 2.1. Cell Culture

The hERG-U2OS cell line was purchased from Codex BioSolution, Inc. (Gaithersburg, MD, USA). All the cell culture reagents were obtained from Invitrogen (Life Technologies, Madison, WI, USA). The hERG-U2OS cells were cultured in DMEM with Glutamax containing 10% fetal bovine serum (FBS), 1% of Non-Essential AA (NEAA), 50 U/mL penicillin, 50 μg/mL streptomycin, and 1 µg/mL puromycin. The cells were maintained at 37 °C under a humidified atmosphere and 5% CO_2_.

### 2.2. Thallium Flux Assay

A FluxORTM thallium flux assay was performed in hERG-U2OS cells with stable hERG expression, as previously described [20]. In this assay, thallium ions are used as surrogates for K^+^ ions to monitor the activity of the hERG K^+^ channel. Astemizole purchased from Sigma–Aldrich (St. Louis, MO, USA), a known hERG channel blocker, was used as the positive control to monitor assay performance. The hERG-U2OS cells suspended in culture medium were dispensed at 1000 cells/well/3μL into 1536-well, black wall/clear-bottom plates using a Multidrop Combi dispenser (Thermo Fisher, Waltham, MA, USA). After the assay plates were incubated at 37 °C overnight, 3 μL of Loading Buffer provided from FluxOR II Potassium Ion Channel Assay Kit (Life Technologies, Carlsbad, CA, USA) was added to each assay well. The assay plates were then incubated at room temperature in the dark for 1 h, followed by the addition of 23 nL of compound, dissolved in DMSO, DMSO only, or positive control, to the assay plate. Each compound was tested at 15 concentrations in three independent runs. After the plates were incubated at room temperature for 10 min, 1 μL of Stimulus Buffer from FluxOR II Potassium Ion Channel Assay Kit was added into each assay well, and the fluorescence intensity (480 nm excitation/540 nm emission) was measured continuously for 2 min at 1 s intervals using a Functional Drug Screening System (FDSS) 7000EX kinetic plate reader (Hamamatsu, Japan). 

The Tox21 chemical library contains approximately 10,000 (8947 unique) small molecules, including pesticides, drugs, industrial chemicals, and food additives commercially sourced by NCATS, NTP, and the EPA [18]. At the time when the hERG assay was screened, the library contained 7871 unique compounds with physical samples available for screening. Multiple criteria were used to select each small molecule, including properties allowing for high-throughput screens (HTS) (molecular weight, volatility, solubility, logP), commercial availability, possible and definite environmental hazards, or exposure concerns, and cost. A diverse group of 88 compounds was selected to be used as an internal control and plated in duplicate on each library plate to perform reproducibility analysis as well as to determine positional plate effects. Each compound in the Tox21 10K compound library was subjected to analytical chemistry quality control (QC), which provides information on the purity and identity of each sample. The QC results and other annotations for each individual compound were made publicly available at https://tripod.nih.gov/tox21/samples; accessed on 14 January 2022. The compounds were serially diluted in Dimethylsulfoxide (DMSO) to 15 concentrations in 1536-well plates, covering a concentration range of up to four orders of magnitude.

### 2.3. Active Chemical Identification

From the Tox21 chemical library, 7871 unique chemicals were tested on FluxOR thallium influx assay in U20S cells in triplicate. Many of these chemicals were sourced independently by NTP/EPA/NCATS from different vendors or the same vendor but different batches; these chemicals have been tested in replicates. Compound concentration-response data analysis was performed as previously described [21,22]. First, raw plate reads for each titration point were normalized relative to the positive control compound (Astemizole: −100%) and DMSO-only wells (0%) as follows:

% Activity = [(Vcompound − VDMSO)/(VDMSO − Vpos)] × 100,

where Vcompound denotes the compound well values, Vpos denotes the median value of the positive control wells, and VDMSO denotes the median values of the DMSO-only wells. The dataset was then corrected using the DMSO-only compound plates at the beginning and end of the compound plate stack by applying an in-house pattern correction algorithm [23]. The half-maximum inhibition values (IC_50_) for each compound and maximum response (efficacy) values were obtained by fitting the concentration-response curves of each compound to a four-parameter Hill equation [24]. Compounds were designated as Classes 1–4 according to the type of concentration-response curve observed [22]. Curve classes were further combined with efficacy and converted to a numeric rank such that more potent and efficacious compounds with higher quality curves were assigned a higher rank. The curve rank is a value ranging from −9 to +9, with −9 to −1 indicating an inhibitory ability, 1 to 9 indicating an activating ability, and 0 meaning inactivity [25,26]. In this study, activity outcomes were defined based on curve ranks as follows: inactive, rank 0; active antagonist −5 to −9, agonist 9 to 5, inconclusive 4 to −4 [21]. To identify chemicals with the potential to inhibit the hERG channel, we selected only antagonist chemicals with curve rank −5 to −9. To increase the confidence of the measurements, only IC_50_ with efficacies above 30% were considered. Next, we analyzed the concentration-response curve for each chemical manually and identified a limited number of chemicals for which the dose-response curve was not significant. Concentration-response curves for these chemicals are available in Supplementary Material File S1 (in red color). Chemical affinity and active/inactive classifications are available in Supplementary Material File S2.

### 2.4. Data Preparation for Molecular Modeling

The chemicals used in the study were represented by a unique SMILES string format and Chemical Abstract Services Registry Number (CASRN). Structure preparation and curation followed the best practices in the field [27,28]. The standardization of the dataset was performed using the following steps: removal of hydrogen atoms, sanitization, removal of any metal ions, stereochemistry check, desolvation, and sieving of salt fragments. Mixtures were excluded in an early step. From the 7871 unique chemicals tested, 7186 chemicals passed through the structure curation process and were used for clustering and QSAR modeling.

From each curated structure, a set of 1D and 2D descriptors were computed using the RDKit package (v. 2020_09_5) in Python 3.7 (https://www.rdkit.org/; accessed on 20 January 2022). An additional set of physicochemical descriptors were predicted on each chemical using the OPERA models (v. 2.7) [29]. 

Only informative and non-correlated descriptors were selected from the initial set of descriptors. First, descriptors having a null variance or the same value for more than 90% of the chemicals were removed. Subsequently, for the remaining descriptors, the pairwise Pearson’s correlation coefficient (ρ) was computed. These were clustered based on ρ > 0.9, and only one descriptor from each cluster was randomly selected for further analysis. Molecular descriptor computation was performed in Python 3.7.

### 2.5. Chemical Category Assignments

To further examine patterns of chemical activity based on the assay outcomes, we assigned a descriptive category type to each chemical based on information obtained from the US EPA Consumer Products Database [30], the Toxic Substances Control Act chemical list (TSCA), and approved drugs lists are available in the EPA chemical dashboard (https://comptox.epa.gov/dashboard; accessed on 20 January 2022).

### 2.6. Structural Clustering

The Tox21 chemical library was clustered into 225 clusters based solely on structural similarity using the selected descriptors and SOM algorithm [31]. The number of clusters was chosen to achieve a balance between cluster number and the number of chemicals by cluster, in accordance with previous studies [32,33]. Hierarchical clustering was performed based on a Euclidian distance matrix and a Ward linkage. Clustering was developed using the R (v. 4.1.0) libraries kohonen (v. 3.0.10), factoextra (v. 3.0.2), ggtree (v. 2.4.1), ape (v. 5.5), and phangorm (v. 2.7.1). 

### 2.7. Chemotype Enrichment Analysis

Both active and inactive chemicals were described using structural chemotypes for further exploration and characterization of assay activity patterns. We examined the chemotypes represented in the top active drug chemicals and other environmental chemical categories with the ChemoTyper application (available at: https://chemotyper.org/; accessed on 20 January 2022), developed by Molecular Networks GmbH and Altamira LLC [34] and compared those trends relative to inactive chemicals to identify structural feature enrichment. The search included both generic structural fragments and Ashby Tennant structural alerts for DNA reactivity [35,36]. One-tailed two-proportion Z-tests were conducted using the continuity correction to compare the proportion of each chemotype (*n*  =  723) in the active chemicals (*n*= 549) with the proportion in inactive chemicals (*n* = 6627) to identify significantly enriched chemotypes in the active chemical space.

### 2.8. QSAR Modeling 

Classification QSAR models were developed to discriminate active and inactive chemicals based on (a) the NCATS thallium flux assay and (b) an expanded dataset, including curated results from the ChEMBL database. The QSAR modeling workflow was conducted according to the best practices in the fields [37,38,39]; details are provided in subsequent sections.

### 2.9. Machine Learning 

Five machine learning-based approaches were used to generate QSAR classification models to predict chemical hERG inhibition potential: (i) classification and regression tree (CART) [40]; (ii) neural network (NN) [41]; (iii) support vector machine (SVM) with a linear, radial, and sigmoid kernel [42]; (iv) random forests (RF) [43]; and (v) linear discriminant analysis (LDA) based on Fisher’s linear discriminant methods [44]. These five approaches have been chosen to cover a large set of methods, including linear and non-linear approaches. QSAR models were built using R (4.1.0) packages: pls (v. 2.8-0), random forest (v. 4.6-14), rpart (v. 3.1.0), e1071 (v. 1.7-8), nnet (v. 7.3-16), and caret (v. 6.0-88). In addition to the classic machine learning approaches, a classification deep neural network (DNN) model was developed [45]. The models were built using Keras 2.3.1 (2020) python deep learning library with TensorFlow 2.1.0 (2020) as the background. Hyperparameters were optimized using a grid optimization on 10-fold cross-validation. Supplementary Information Table S1 reports grids of optimization and parameters and hyperparameters chosen.

A similar protocol to that applied in [32] was used. Briefly, each model was tuned via a grid optimization as appropriate for the machine learning algorithm, and parameters/models were chosen to maximize 10-fold cross-validation performance using Matthew’s correlation coefficient (MCC). The MCC criterion is considered optimal to analyze the QSAR model performance, as it represents the correlation between the observed and predicted classification with value ranges from −1 (random prediction) to +1 (perfect prediction) and is a statistical metric that is least affected by the imbalance in the dataset. 

### 2.10. Under-Sampling Protocol

For the NCATS assay results, considering the unbalanced dataset, i.e., more inactive chemicals (6598 inactive and 549 active chemicals), under-sampling was applied via random selection of inactive chemicals to yield a ratio of 75% inactive and 25% active chemicals. This under-sampling was applied to the training set (covering 85% of the dataset), where each model was built five times with a different inactive set (1556 inactive chemicals and 467 active chemicals) to cover the full training set of chemicals. Model performance was reported as mean with standard deviation on the five repetitions for the training set, cross-validation, and external (hold-out) test set (the remaining 15% of the data). 

### 2.11. Evaluation of the Classification Model Performance

The generated models were evaluated for their performance by calculating the number of true positives (TP), true negatives (TN), false positives (FP), and false negatives (FN). TP is the number of hERG antagonists that were predicted as antagonists by the generated models, TN is the number of inactive chemicals that were predicted as inactive, FP represents the inactive chemicals predicted as hERG antagonists, and FN represents the number of hERG antagonists predicted incorrectly as inactive molecules. From those numbers, performance was computed using sensitivity (SE), specificity (SP), the overall prediction accuracy (Q), the balanced accuracy (Qb, average of sensitivity and specificity), and the Matthews’s correlation coefficient (MCC):

SE = TP/(TP + FN)

SP = TN/(TN + FP)

Q = (TP + TN)/(TP + TN + FP + FN)

Qb = (SE + SP)/2

MCC = (TP.TN − FP. FN)/√((TP + FP) (TP + FN)(TN + FP)(TN + FN))

### 2.12. Dataset Enrichment 

**Figure 1:**
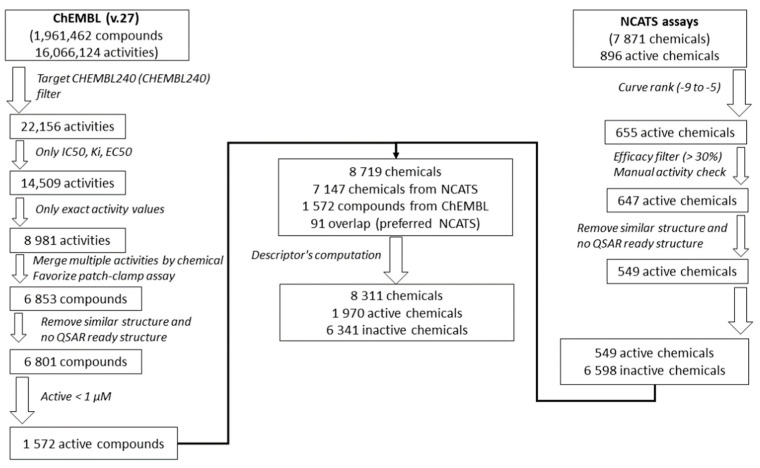
Workflow of the Tox21 dataset enrichment using the ChEMBL database.

To further develop and test the QSAR models, the Tox21 dataset was enriched using the ChEMBL database (Version 27) [46], processed using a similar approach as that applied in [47], Figure 1. First, activities on the hERG target (ChEMBL240) were extracted from the database. Only IC_50_, Ki, and EC_50_, with exact activity values, were considered. In the case of multiple activity values for one chemical, activities from patch-clamp assays, considered as the golden standard for hERG studies [48,49], were prioritized. If multiple patch-clamp activity values were still available for one chemical, activities were averaged, or the chemical was removed in case of more than one log10 difference between activity values. Then, chemical structures were prepared in a QSAR-ready format, see previous section. Chemicals without QSAR-ready structures were removed, and in the case of duplicates, only one structure was chosen randomly. Finally, only active chemicals with an activity ≤ 1 µM, i.e., with pAffinity (−log10(affinity)) ≥ 6 [50], were retained and used to enrich the Tox21 chemical dataset. Only 48 active chemicals were found to be overlapping in both sets. The coefficient of correlation between the −log10 activities is equal to 0.65 (or 0.85) if only patch-clamp assays in ChEMBL are considered), Figure S1. 

### 2.13. Validation Sets

A first test set was built from the initial chemical set using a random selection of 15% of all data, taking into consideration the balance between active and inactive chemicals. Next, two external validation test sets were extracted from the literature. The first was a qHTS dataset generated using the comparable protocol, U2OS cell lines using the FluxORTM thallium flux approach, and the same NCATS laboratory, previously applied to identify small molecule inhibitors of the human hERG channel activity (PubChem ID: AID588834) [14]. It included 5381 substances, where 3894 were shared with the dataset analyzed here. Only non-overlapping chemicals with the Tox21 chemical library were used for the external test set. For the second test set, we performed a literature review using the SysRev platform (https://sysrev.com/u/2376/p/35538; accessed on 20 January 2022). The search strategy included the terms (“herg” or “ether-à-go-go-related potassium channel” or “KCNH”) and “inhibition” in the PubMed database. The search returned 915 articles, which were screened by title/abstract screening using a Sysrev data extraction form to determine inclusion/exclusion and the affinity of the tested agents for hERG inhibition (https://sysrev.com/; accessed on 20 January 2022). 

### 2.14. Applicability Domain (AD)

**Figure 2:**
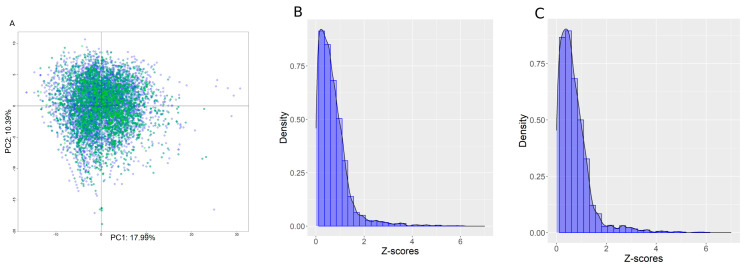
(**A**) PCA plot in the two first dimensions computed using the filtered set of descriptors on the training set. Blue represents chemicals in the training set, and green is the internal test set. PCA covered, in the first two dimensions, 28.3% of the descriptor variability. (**B**,**C**) Z-score distributions for the training and internal test sets, respectively.

For each chemical, the model applicability domain was determined using a Z-score computed from the distribution of the Euclidian distances between the centroid of the principal component analysis (PCA) from the training set. The PCA was computed on the Tox21 chemical library using the selected descriptors set, see Figure 2 panel A. The PCA centroid was computed in *n* dimensions covering at least 80% of the descriptor variability. Next, a distribution of all distances between chemicals and the centroid was used to compute a Z-score for each chemical using the formula:

$$Z_{i}=(x_{i}-\bar{X})/S$$

where Z_i_ is the Z-score for chemical i, x_i_ is the Euclidean distance between chemical i and the centroid, $\bar{X}$ is the average of all Euclidean distances to the centroid, and S is the standard deviation of all Euclidian distances to the centroid. 

Figure 2, panels B and C, show the Z-score distribution for the training and internal test sets. Distributions were remarkably similar, with an average Z-score equal to 0.71 ± 0.73 for the training set and 0.71 ± 0.74 for the internal test set, which demonstrates good segregation of the chemicals between the two sets. Roughly, a chemical with a Z-score < 2 was fully inside the AD, a Z-score between 2 and 4 was at the border of the AD, and a Z-score > 4 was out of the AD.

All scripts used for this project were developed using python 3.7 and R > 3.6 and are available on GitHub: (https://github.com/ABorrel/cardiotox_hERG; accessed on 20 January 2022) for data mining and clustering, and (https://github.com/ABorrel/QSAR-QSPR; accessed on 20 January 2022) for QSAR modeling. Supplementary Material File S3 contains descriptor sets for the 7180 chemicals used in the modeling. Supplementary Material File S4 contains the activity of the enriched dataset. Supplementary Material File S5 includes all descriptor sets used for both the Tox21 tested chemical library and the enriched set. 

## 3. Results

### 3.1. Chemical Activity for hERG Inhibition

**Figure 3:**
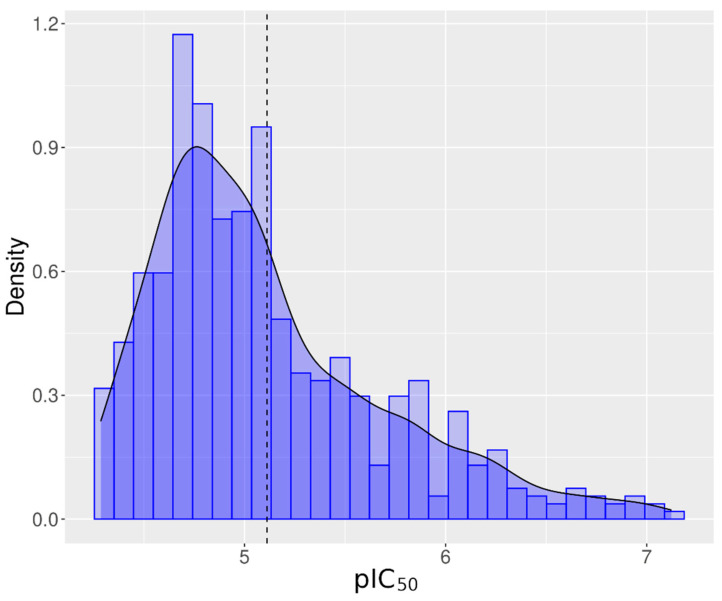
Distribution of pIC_50_ (−log(IC_50_)) in M) for all active chemicals for hERG. Vertical dash line represents the distribution mean. The bell shape line represents the normal distribution of the pIC_50_.

Of the 7871 unique chemicals evaluated from the Tox21 10K chemical library, 896 chemicals (11.38%) inhibited hERG channel activity, referred to as antagonists. Eighty-four percent of the tested chemicals (6616) did not alter hERG activity, and 4.3% (339 chemicals) exhibited responses that were considered inconclusive because of incomplete responses or missing signals. Table 1 summarizes the number of active chemicals filtered through each step. In the end, 549 antagonist chemicals representing 7.78% of the Tox21 chemical library were considered reliable hERG inhibitors in this assay and utilized for structural analysis. The average IC_50_ was equal to 7.59 µM ± 5.64 µM. The distribution of the pIC_50_ is shown in Figure 3. Peak activity was observed around pIC_50_ (−log10(IC_50_)) equal to 4.8, which is equivalent to an IC_50_ equal to 15.85 µM. In Supplementary Material File S6, active chemicals are presented, ranked by activity and class.

### 3.2. Active Chemical Categories

To characterize active chemicals by substance type and use case, the Tox21 10K chemical library was classified using the US EPA consumer products database [30], the Toxic Substances Control Act chemical list (TSCA), and lists of approved drugs available via the EPA chemical dashboard (https://comptox.epa.gov/dashboard; accessed on 20 January 2022). Based on these resources, 4950 of the 8305 unique chemicals included in the Tox21 chemical library could be classified into 80 classes. The most populated classes of active chemicals are represented in Supplemental Figure S2, Panel A. From the 549 active chemicals, we were able to classify 216 chemicals, Supplemental Figure S2 Panel B. Most of the active chemicals are drug chemicals (125/549), followed by toxic substances in the TSCA (65/549) list, and pesticides (50/549). Interestingly, five UV absorber chemicals were found to be active in this assay. However, there is an important risk that those chemicals are interferent chemicals because they absorb light (around 400 nm) at a wavelength close to the fluorescence technology measurements (480 nm). 

### 3.3. Most Active Chemicals 

The 896 compounds that decreased hERG channel activity had IC_50_ values ranging from 0.075 µM to 72 μM, with 65 chemicals having an IC_50_ < 1 μM. The selective dopamine reuptake inhibitor GBR 12909 dihydrochloride (Vanoxerine) was the most potent compound, with an IC_50_ of 0.075 µM. Other potent compounds included Eliprodil (IC_50_ = 0.09 µM), Amperozide hydrochloride (IC_50_ = 0.91 µM), Lidoflazine (IC_50_ = 0.11 µM), Dofetilide IC_50_ = 0.13 µM), Ritanserin (IC_50_ = 0.18 µM), Astemizole (IC_50_ = 0.22 µM), Terfenadine (IC_50_ = 0.27 µM), Cisapride (IC_50_ = 0.32 µM), Domperidone (IC_50_ = 0.32 µM), Haloperidol (IC_50_ = 0.57 µM), Bepridil (IC_50_ = 0.55 µM), Loperamide (IC_50_ = 0.62 µM), and Amiodarone (IC_50_ = 0.64 µM). In the literature, all of these drugs are reported to cause hERG inhibition [51,52,53]. However, very few environmental chemicals were in the list of chemicals with IC_50_ < 1 µM. The concentration response curve of these chemicals is presented in Supplementary Material File S1 (blue color). The 65 chemicals with IC_50_ < 1 µM are listed in Supplementary Material File S2.

### 3.4. Assay Dependent Potency Shift 

**Figure 4:**
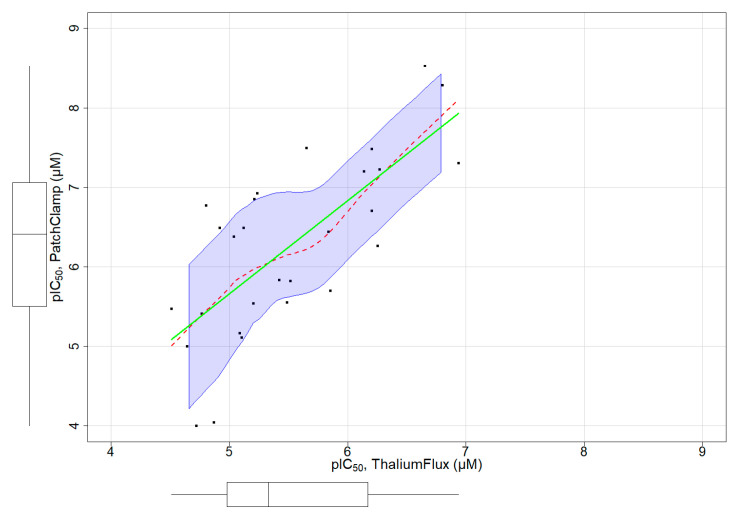
Linear relationship of pIC_50_ obtained from thallium flux (x-axis) and patch-clamp (y-axis) assays for a small subset of chemicals. The regression line and the non-parametric regression line are represented in green and red colors, respectively.

A subset of chemicals in our dataset had both thallium flux assay results and patch-clamp assay data reported in ChEMBL (Figure S3). Figure 4 depicts a distribution plot of pIC_50_ values for 28 chemicals with both assay types, demonstrating a clear linear relationship between both assays. We fit a regression model with this limited set of chemicals to characterize the relationship between the patch-clamp and thallium flux assay outcomes (Figure 4). The model fit is below:

Patch Clamp  =  −0.1953 + (1.17 ∗ Thallium Flux)

The values of R-squared and adjusted R-squared were 0.5256 and 0.5074, respectively. The *p*-value was 1.279 × 10^−5^. From these results, we confirm a significant linear relationship, showing a potency shift from the thallium flux assay (less potent) to the patch-clamp (more potent). This linear relationship can be used as an adjustment factor when applied to the pIC_50_ obtained from the Thallium flux assay to predict the activity of chemicals in patch-clamp assays. 

### 3.5. Structural Activity Patterns

**Figure 5:**
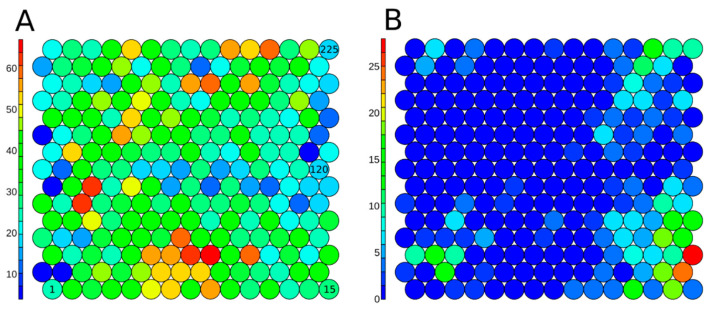
Structure-based SOM on the 7187 structure-curated Tox21 chemicals, including 225 clusters in total, colored based on the number of chemicals included in each cluster (**A**) for all Tox21 structure-curated chemicals, and (**B**) on active chemicals (7.78%) in the hERG inhibition assay. The serial numbers of clusters are given inside the circles (Cluster 1-Cluster 225).

The 7187 unique chemicals from the Tox21 10K library were clustered using a SOM approach from a set of non-correlated and informative 1D-2D structural descriptors. Chemicals were clustered in 225 clusters to optimize segregation. The SOM with all chemicals from the set is presented in Figure 5 panel A. On average, each cluster was composed of 36 ± 14 chemicals. Cluster 91 had the minimum number with four chemicals, and Cluster 39 had the maximum number with 67 chemicals. In Figure 5, panel B, the structural clusters are colored based on the number of active chemicals. The hERG inhibitors are largely grouped into one corner of the SOM map, demonstrating that these chemicals share some structural patterns and motivating the construction of QSAR models.

**Figure 6:**
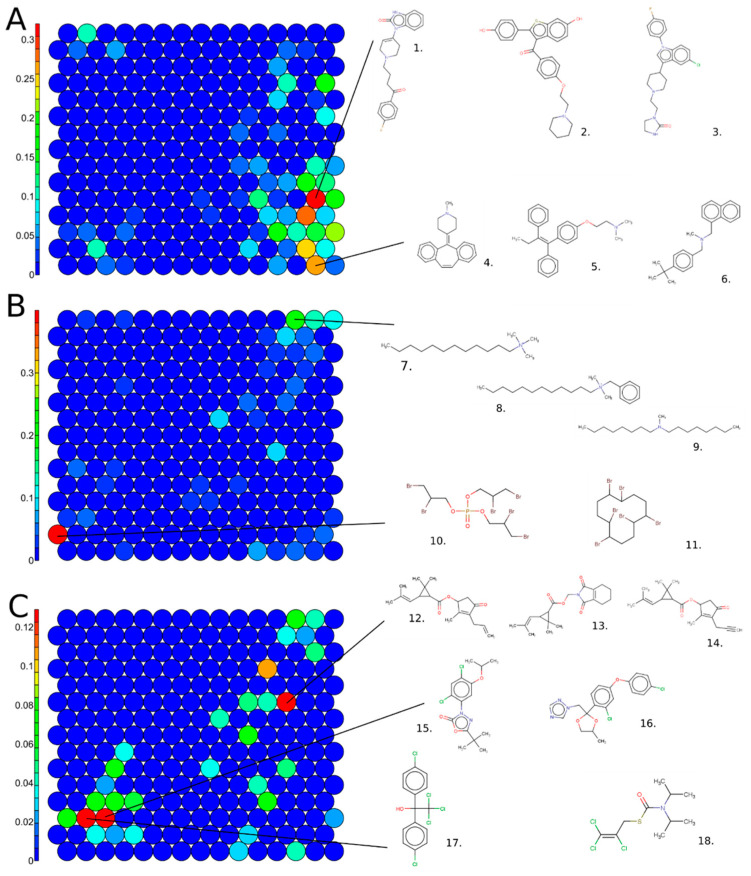
Structure-based SOM on the structure-curated Tox21 chemicals, including 225 clusters in total, color scales represent (**A**) percentage of active chemical in drug class, (**B**) percentage of active chemical in TSCA class, and (**C**) percentage of active chemical in pesticide class. Examples of chemical structures for enriched clusters are displayed. Cluster 74: 1. 548-73-2, 2. 84449-90-1, 3. 106516-24-9; cluster 14: 4. 129-03-3, 5. 10540-29-1, 6. 101828-21-1; Cluster 223: 7. 112-00-5, 8. 1119-94-4, 9. 4455-26-9; Cluster 16: 10. 126-72-7, 11. 3194-55-6; Cluster 148: 12. 584-79-2, 13. 7696-12-0, 14. 23031-36-913; Cluster 33: 15. 19666-30-9, 16. 119446-68-3; and Cluster 32: 17. 115-32-2 and 18. 2003-17-5.

In Figure 6, the same SOM map is colored using the percentage of active chemicals by cluster for drugs (A), pesticides (B), and TSCA chemicals (C). In Figure 6A, the structural clusters 14 and 74 are highlighted because they are enriched in hERG inhibitors that are drugs. Cluster 74 includes 64% of the drugs, such as Droperidol (1. 548-73-2), Raloxifene (2. 84449-90-1), or Sertindole (3. 106516-24-9). Structurally, those chemicals include a benzimidazole group ramified with oxygen groups, such as ketones or alcohols, or with chlorine-derived groups. Adjacently in the SOM, similar sub-structures were found in Cluster 59, which was also enriched with drugs that are hERG inhibitors. Cluster 14 includes drugs, such as Cyproheptadine (4. 129-03-3), Tamoxifen (5. 10540-29-1), and Butenafine (6. 101828-21-1). Structurally, chemicals in this cluster include tertiary/secondary amines and have substitutions on nitrogen with bulky groups, as well as aromatic ring substructures.

Clusters 16 and 223 are the most enriched in TSCA chemicals active for hERG inhibition. In Cluster 223, most of the chemicals included a long aliphatic chain ramified with a tertiary amine, e.g., Dodecyltrimethylammonium chloride (7. 112-00-5) and Benzyldimethyldodecylammonium chloride (8. 139-07-1). Chemicals can also include a secondary amine, for example, N-Methyldioctylamine (9. 4455-26-9). These chemicals are mostly used as lubricants in the hydraulic manufacturing field. Cluster 16 includes bromine chemicals, such as Tris(2,3-dibromopropyl) phosphate (10. 126-72-7) or Hexabromocyclododecane (11. 3194-55-6), used as flame retardants or thermal insulators. 

Pesticide antagonists for hERG are mostly found in clusters 148, 32, and 33. Cluster 148 contains pyrethroids, characterized by a pyran core, ramified with an ether group, such as Allethrin (12. 584-79-2), Tetramethrin (13. 7696-12-0), and prallethrin (14. 23031-36-9). Cluster 33 includes mostly chemicals ramified with benzo chlorine groups, e.g., Oxadiazon (15. 19666-30-9) and Difenoconazole (16. 2003-17-5). Finally, Cluster 32 is populated with organochlorine pesticides such as Dicofol, a DDT derivative (17. 115-32-2), or Tri-alate (18. 2303-17-5).

**Figure 7:**
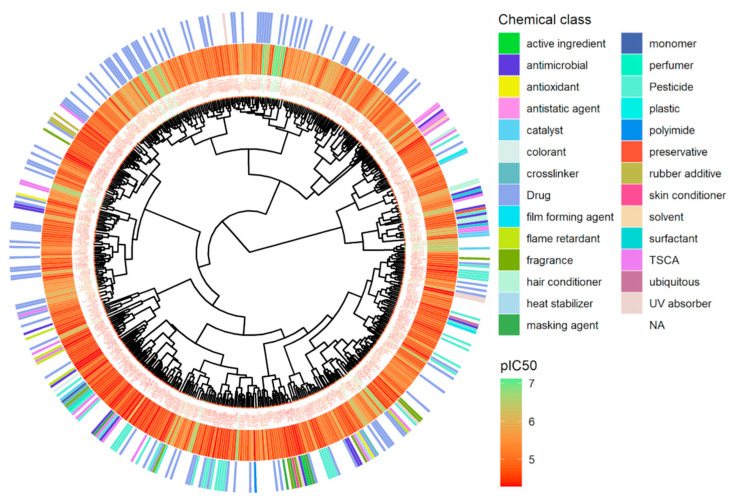
Hierarchical clustering of antagonist chemicals for hERG. Hierarchical clustering is applied using Euclidean distance computed from a set of non-redundant molecular descriptors and Ward linkage, see Methods. Potency in terms of pIC_50_ (M) for each chemical is represented using a continuous color scale from red to green and chemical classes are displayed via a discrete color scale.

Hierarchical clustering using Euclidean distance and Ward linkage shows that active chemicals in the same class are clustered together (Figure 7). The most active chemicals, colored in green, are drugs clustered on the top of the tree, while most of the pesticides are in the bottom. The ranking of all active chemicals is available in Supplementary Material File S2.

### 3.6. Chemotype Enrichment

**Figure 8:**
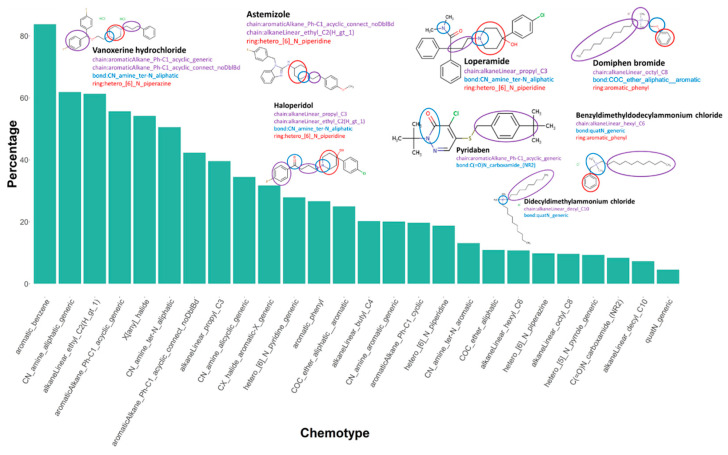
Active chemicals enriched for specific chemotypes. One-tailed two-proportion Z-tests were conducted with the continuity correction of the 729 chemotypes to compare the proportion of each chemotype in the active chemicals and inactive chemicals. Few of the 106 enriched chemotypes in active chemicals are shown here with the example chemicals, color-coded to link the chemotype name with the example chemical structure. Purple: chain, blue: bond, and red: rings.

We identified chemotypes, or structural features, that are present at significantly higher proportions in the active set relative to the inactive chemicals. The 106 enriched chemotypes are listed in Supplementary Material File S7. We noted that multiple heterocyclic ring-like chemotypes were included in the drug chemicals, for instance, hetero_[6]_N_piperazine and hetero_[6]_N_piperidine. Additionally, drug chemicals also demonstrated the presence of small-chain chemotypes, such as aromaticAlkane_Ph-C1_acyclic_generic and aromaticAlkane_Ph-C1_acyclic_connect_noDblBd. The enriched chemotypes in the drug class also included multiple CN bond chemotypes, such as CN_amine_ter-N_aliphatic. Many environmental chemicals demonstrated the presence of quaternary ammonium bond-like chemotypes, including quaternary ammonium chemicals (QAC), domiphen bromide, didecyldimethylammonium chloride, and benzyldimethyldodecylammonium chloride. Our data are consistent with a previous study identifying QACs as potent inhibitors of hERG potassium channels [14]. These chemicals also contained bulky alkyl chain-like chemotypes, such as alkaneLinear_hexyl_C6, alkaneLinear_octyl_C8, and alkaneLinear_decyl_C10, relative to pharmaceuticals. The details and definitions of these chemotypes can be found in Supplementary Material File S7. Figure 8 shows the enriched chemotypes in active chemicals with examples from both drug and environmental chemical classes. 

### 3.7. QSAR Classification Models for hERG Inhibition Using the Tox21 FluxOR Thallium Influx Assay Dataset

It is evident from the above unsupervised statistical analyses that chemicals exhibiting hERG inhibition have common structural properties. Based on selected structural and physico-chemical molecular descriptors, QSAR models were developed to predict chemical hERG inhibition potential. We applied multiple linear and non-linear machine learning algorithms, including decision trees and deep learning, see Methods, to build classification models. To handle the imbalanced set, i.e., only 7.8% active chemicals, an under-sampling approach was used, and each model was a combination of five models built with 467 active and 1556 inactive chemicals randomly chosen from 85% of the dataset used for the training. QSAR performances are reported in Table 2 for 10-fold cross-validation (CV) on the training sets and on the test set (15% of the dataset).

The evaluation of performance criteria revealed that RF, SVM-radial, and DNN models outperformed other models. The MCCs obtained for the RF models were 0.657 ± 0.014 and 0.557 ± 0.019 for the 10-fold CV of the full training set and the internal test set, respectively. These results confirm the reliability of the model and dispel concerns of overfitting, despite the relatively high performance of the undersampled training set (0.999 ± 0.005). Models based on DNN and SVM-radial exhibit close performance with CV MCCs of 0.659 ± 0.021 and 0.685 ± 0.016, and test set MCCs of 0.517 ± 0.041 and 0.563 ± 0.02, respectively. SVM models with a radial kernel outperformed other SVM models with linear or sigmoid kernels (CV MCC of 0.629 ± 0.013 and 0.587 ± 0.009, respectively). The CART, NN, and LDA models were weaker, with CV MCCs equal to 0.561 ± 0.019, 0.526 ± 0.057, and 0.629 ± 0.008.

### 3.8. QSAR Classification Models for hERG Inhibition Using the Enriched Dataset

**Figure 9:**
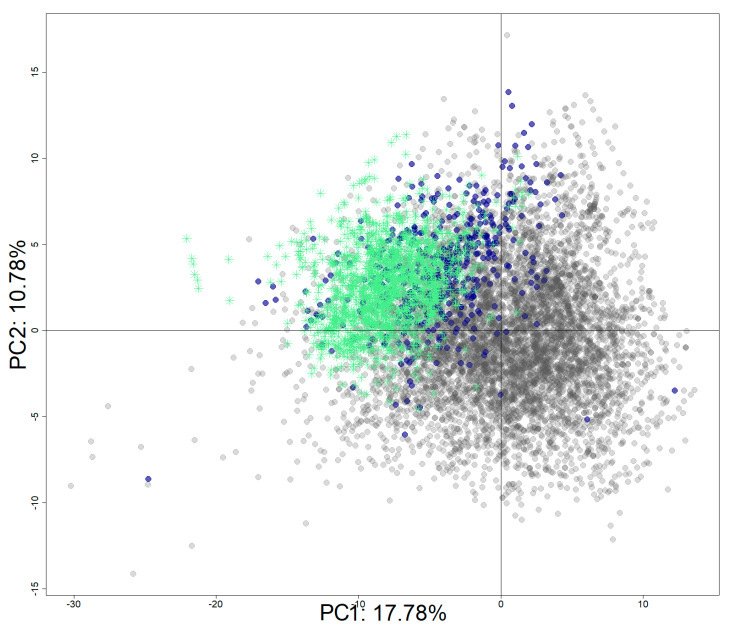
PCA projection of the enriched dataset (Tox21 + ChEMBL). PCA was computed using the Tox21 chemical library. Tox21 chemicals are colored in grey for inactive chemicals and in blue for active chemicals. Active chemicals extracted from the ChEMBL database are represented with green stars.

The Tox21 chemical set was enriched with identified hERG inhibitors from ChEMBL (v.27). The final set includes 8311 chemicals with 1970 active chemicals and 6341 inactive chemicals. Figure 9 shows the projection of the active chemicals from ChEMBL onto a principal component analysis (PCA) plot based on the Tox21 10K chemical library, where active chemicals from both source databases clustered together (top left quadrant), as did inactive chemicals (bottom right). It is important to note that most of the chemicals are drugs or drug-like molecules in accordance with the database composition [46]. 

The same QSAR protocol was applied on this dataset, except that undersampling was not performed due to the high range of active chemicals in the set (24%). Model performance is reported in Table 3, and most algorithms demonstrated elevated statistics in comparison to the previous models. The average 10-fold CV accuracy for this dataset was 0.935, while that of the QSAR models developed using the Tox21 10K chemical library was only 0.891. The performance discrepancy between machine learning approaches was less than the QSAR models built using the non-enriched chemical set. As previously, RF performed slightly better than other models with a CV MCC of 0.863 and the tested MCC of 0.857. SVM with a radial kernel performed better (CV MCC of 0.870) than other SVM models using a linear and sigmoid kernel (CV MCC of 0.847 and 0.831, respectively). CART and NN machine learning remained weaker than other models with CV MCC equal to 0.684 and 0.779, respectively. LDA and DNN performed well with CV MCC equal to 0.828 and 0.836.

### 3.9. Significant Molecular Descriptors

**Figure 10:**
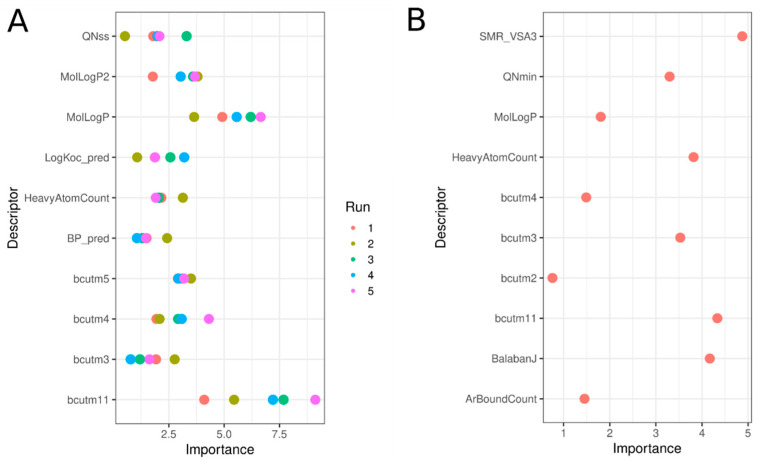
Variable importance plots for the top 10 descriptors involved in the RF models predicting hERG inhibition using the Tox21 chemical set (**A**) and the enriched set Tox21-ChEMBL (**B**). In A, five values for each descriptor are reported, corresponding to each of the five sub-models developed for the under-sampling approach.

One advantage of the RF model is the ability to see which descriptors have the most influence on model performance. The top 10 most important descriptors in RF models built using the Tox21 chemical library and the enriched set are shown in Figure 10, with details in Table 4. Using the original chemical set, Panel A, values are reported for each of the five sub-models built with the undersampling approach. Here, a balance between physico-chemical and structural descriptors achieved significant segregation between active and inactive chemicals and allowed profiling of the active chemicals. Chemicals predicted as hERG inhibitors were bigger than inactive chemicals, as indicated by the number of heavy atoms (average of 25 vs. 16 for inactive), increased aromatic bond count, i.e., ~16 for active and only ~6 for inactive chemicals, and burden descriptors (bcut, directly correlated with the chemical mass). Partition coefficient-related descriptors (LogP_pred, MolLogP, and MolLogP2) were higher for active chemicals, as were the charge-type descriptors (QNss and QNmin), indicating that active chemicals included more charged nitrogen atoms.

### 3.10. External Validation of the QSAR Models 

To further check model robustness and practical applicability, additional external chemical test sets were predicted (Table 5), beginning with PubChem (ID: AID588834). This dataset was developed using a similar assay protocol applied to the Tox21 chemicals library. An overlap of 3894 chemicals was found in both sets. The correlation between pIC_50_s on the active chemicals found in both sets was 0.86 (Supplemental Figure S4), confirming that these datasets, generated at different points in time using similar protocols, gave close results for overlapping compounds. 

**Figure 11:**
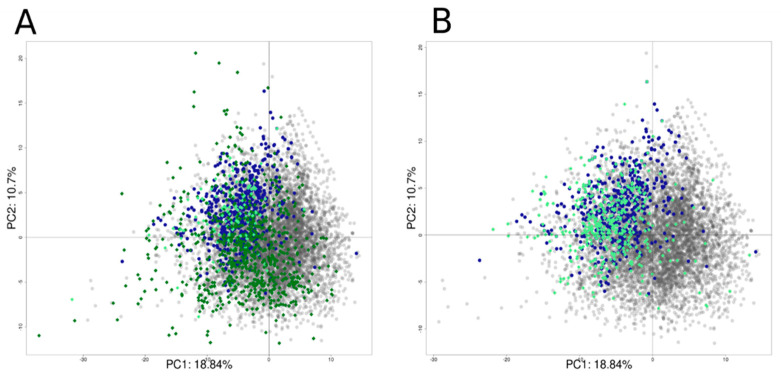
Projection of the external sets AID58834 (**A**) and external inhibitors set (**B**) on the PCA computed using the Tox21 chemicals library. Active chemicals in the Tox21 chemical library are represented in blue dots, inactive chemicals in grey dots, and external set chemicals are represented using a rhombus shape in light green for active and dark green for inactive chemicals.

Only non-overlapping chemicals with defined structures (*n* = 859), not included in the Tox21 dataset, were predicted. This (PubChem) external test set included 113 actives and 741 inactive chemicals. The second (Lit-based) external test set was extracted from a literature search that identified 392 potential hERG inhibitors with defined structures. Figure 11 shows the PCA plot computed using the Tox21 chemical library with the external test set projections. Most of the active chemicals from both the PubChem set (Figure 11A) and Lit-based set (Figure 11B) are found in the left top quadrant overlapping chemicals found active from the Tox21 chemical library, as confirmed with the applicability domain score. The average Z-score for the PubChem set was equal to 1.27 ± 1.30 (0.71 ± 0.75 for Tox21 chemical library set), with only 104 chemicals with a Z-score above four, and for the Lit-based set, the average Z-score was equal to 0.879 ± 0.963, with only 37 chemicals with a Z-score above two.

The best QSAR models developed previously were applied to predict the activity of these sets of chemicals. For the PubChem test set, the RF models built using the Tox21 chemical library and the enriched set performed well with MCCs equal to 0.631 and 0.641, respectively. QSAR models built using the enriched set performed slightly better with an improvement of the specificity criteria, from 0.966 to 0.984, explained by the fact that more active chemicals were used to train the model. DNN-based models underperformed RF models using both training sets with MCCs equal to 0.245 and 0.248 for models built with the original and enriched set, due primarily to the specificity criteria that drop from 0.966 and 0.984 to 0.661 and 0.690 from the RF to the DNN models. However, these DNN models had better sensitivity, i.e., ability to predict active chemicals, on both models (0.690 and 0.717 versus 0.619 and 0.549 with the RF models). SVM models that exhibited good performance in cross-validation and in internal test sets underperformed RF and DNN models with a weak sensitivity, below 0.20, and are reported in Supplementary Information Table S2.

For the Lit-based dataset, the best model was only able to well predict 161 active chemicals (TP) out of the 392. DNN models performed better than RF models, which confirms the ability of the DNN to predict better active chemicals than RF models. On this dataset, models trained with only the Tox21 chemical library gave better performances than models trained using the enriched set, 161 TP and 157 TP, respectively. 

Consensus models built using the average probability for each chemical of the DNN and RF prediction were also attempted and applied for both external test sets. These models performed as an average of the RN and DNN for the PubChem test set and did not improve the RF performances on the Lit-based test set. 

## 4. Discussion

Here, we report results from a high-throughput chemical screen in a cell-based thallium influx assay to profile the hERG inhibition potential of the diverse Tox21 10K chemical library, including drugs, environmental and industrial chemicals, and personal care product ingredients, and the application of artificial intelligence to train predictive models based on these data. The compounds were screened in triplicate, providing a robust assessment of the assay technical performance and building assurance in the chemical activity calls. The approach utilized in this study applies a qHTS approach combined with QSAR analysis to build in silico screening tools for predictive safety assessment and identification of structural features modulating hERG channel activity. 

The Tox21 10K chemical library used for this screening effort was a far more diverse chemical space than previously reported in the literature with respect to understanding potential chemical effects on cardiac action potential, covering not only drugs but also environmental and industrial chemicals with widespread potential human exposure. Of the total active chemicals (*n* = 549) identified in this study, 63 had an IC_50_ < 1 μM against the hERG channel. We found that many of the active chemicals belonged to categories of chemicals designed to be bioactive (i.e., pharmaceuticals, insecticides, surfactants, and fungicides), with the majority of highly active chemicals being drugs. The most potent compound in this assay was the GBR 12909 dihydrochloride (Vanoxerine), with an IC_50_ of 0.075 µM. Vanoxerine, a piperazine derivative, a potent and selective dopamine reuptake inhibitor, initially developed as an antipsychotic and antidepressant. Our results are consistent with previous reports that it was evaluated as an antiarrhythmic drug and is a potent cardiac hERG channel blocker with IC_50_ reported as 0.22 and 0.00084 μM in thallium flux [54] and patch-clamp assay, respectively [55]. Eliprodil, Pimozide, Fluspirilene, Sertindole, Ritanserin, Bromperidol, Trifluperidol, and Haloperidol are some of the most active drugs found in the assay. All of these are used as antipsychotic drugs and, interestingly, contain a piperidine moiety, which may be identified as a flag in lead compound selection. There are multiple studies reporting various adverse clinical CV effects of these drugs, ranging from alteration in heart rate (HR) and changes in blood pressure (BP) to more severe effects such as QT prolongation and congestive heart failure [56,57,58,59,60]. In addition to this, we found several other classes of drugs showing activity against hERG in the present assay, such as antihistamine drugs (Astemizole, Terfenadine, Clemastine, Ebastine), prokinetic drugs (Cisapride, Domperidone), and some chemotherapeutic agents (Lapatinib, Bosutinib methanoate). The ability of these non-CV drugs to induce QT interval prolongations or arrhythmias has been established by various studies [61,62,63,64,65,66,67,68]. One of the most well-known chemotherapeutic agents with cardiotoxic effects is 5-fluorouracil, which was tested here, but was inactive, consistent with the hypothesized mechanisms of direct cellular damage and/or ischemia, rather than hERG channel inhibition [69]. As expected, several antiarrhythmic drugs, such as Lidoflazine, Dofetilide, Bepridil, and Amiodarone, demonstrated activity here. Multiple beta-blockers used as antiarrhythmic drugs, such as Sotalol and Labetalol, were also found to be active in the analysis [70]. 

Methyltrioctylammonium trifluoromethanesulfonate, tributyltetradecylphosphonium chloride, tetraoctylphosphonium bromide, hydramethylnon, 3-Didecyl-2-methylimidazolium chloride, basic blue 7, didecyldimethylammonium chloride, tetra-N-octylammonium bromide, and benzethonium chloride are some of the environmental chemicals found to be most active in the assay. Many of these chemicals are quaternary ammonium compounds (QACs). QACs are highly hydrophobic chemicals, possessing surfactant properties and, due to their detergent properties, are frequently utilized in disinfectants [71,72]. Three of these chemicals, namely didecyldimethylammonium chloride, tetra-N-octylammonium bromide, and benzethonium chloride, were previously known to exert hERG inhibitory effects in reported studies [14,73,74]. QACs have seen a dramatic increase in use and human exposure potential due to increased disinfection and cleaning protocols resulting from the SARS-CoV-2 outbreak and associated COVID-19 pandemic [75,76,77]. Many other pesticides and flame retardants, such as Kepone, Endosulfan, Pyridaben, Dinocap, o p’-DDT, allethrin, lindane, parathion, prallethrin, and triphenyl phosphate, also demonstrated inhibitory activity against the hERG channel in the assay. 

By applying structure-based clustering and cheminformatics analyses, we identified enriched chemotypes that may be important structural features contributing to activity in the hERG inhibition assays. Multiple enriched chemotypes in drug molecules contained piperidine, piperazine-like heterocyclic rings, and CN bonds, while environmental chemicals contained quaternary N bonds and long alkyl chain-like chemotypes. 

In recent years, many articles on the prediction of cardiotoxicity based on machine learning algorithms have been published, and several computational methods have been established for predicting the cardiotoxicity of chemicals [78,79,80,81]. Most of them focus on drug-like or drug optimization molecules. Multiple QSAR models are available for hERG. However, model comparisons are challenging due to the use of in-house test sets, lack of available code to generate external predictions or incompatible assay technologies. For example, in [78], the authors used a ChEMBL v22 dataset (*n* = 8705) to generate three classification models, i.e., random forest, multi-layer perceptron, and sequential minimal optimization. The best performing consensus model was validated by an in-house external test set (*n* = 585) with an accuracy of 0.93, which was not available for us to compare our model predictions against. Another study describes the generation of SVM, RF, and extreme gradient boosting classification models using ChEMBL v24 and a threshold of 30 μM to define hERG blockers and non-blockers (*n* = 1865) [80]. In [79], authors also generated ensemble models by fusing a subset of base classifiers via averaging their predictive probability. The accuracy of the best performing ensemble model on the external set (*n* = 407) was found to be 0.79. Using the benchmark models developed in [47], models are available and performed better on the dataset built using the assays AID588834 with an MCC equal to 0.87. However, this set is fully included in its training set. On the second set, this model is only able to find 133 active chemicals, compared to 161 using our developed models. In [81], support vector classification (SVC) models were generated using 4324 compounds screened for hERG channel inhibition in a thallium flux assay in qHTS format with the averaged area under the receiver operator characteristics curve (AUC-ROC) of 0.93 for the tested compounds. The external validation for the generated models was performed using a set of 66 drug molecules that exhibited an AUC-ROC of 0.86. The majority of these studies were inclined towards drug discovery and/or lead optimization projects and included chemicals with more drug-like, drug derivate structures. Here we developed a model with a broader chemical space diversity, including drug-like structures as well other environmental chemicals such as pesticides, biocides, surfactants, etc.

To validate the outcome of the hERG channel assay in thallium ion flux assay format, we compared several known hERG inhibitors for their activity on the hERG channel in thallium flux and whole-cell patch-clamp collected from literature sources. For the functional screening of hERG channels, the patch-clamp method is traditionally used and considered a gold standard. However, patch-clamp assay and other assays are low-throughput. The thallium flux assay provides vastly improved throughput for functional measurement of hERG activity. Unlike other ion channels that interact only with ligands of specific structural classes, the hERG potassium ion channel can be altered or modulated by a broad spectrum of structurally diverse compounds. Therefore, a functional assay of hERG channels is the ultimate methodology for examining the hERG activity of compounds. We found that the activities of many hERG channel inhibitors in this thallium flux assay are consistent with those obtained in automated patch-clamp experiments, albeit shifted towards lower potencies. For instance, under our assay conditions, dofetilide and cisapride, two structurally distinct hERG inhibitors, had IC_50_ values of 0.139 and 0.325 µM, respectively, in contrast to reported IC_50_ values in standard patch-clamp assays of 0.015 and 0.085 µM, respectively [51]. Several reports have suggested varying degrees of potency shift depending on the assay platform and the given compound tested for hERG activity [51,52,53]. It is well established that surrogate ions have a remarkable difference in permeabilities relative to physiological ions. This could lead to a right shift of affinity for many known hERG inhibitors [53]. Further advancement of the thallium-based flux assay could result in a better correlation with electrophysiology. Thus, thallium flux assay is proposed as an effective and alternative method for large-scale compound screens to assess compound activity on the hERG channel in vitro.

## 5. Conclusions 

An essential goal of the Tox21 collaboration is the generation of a robust, reliable dataset to rank chemicals for more comprehensive but lower throughput toxicological studies and to generate reliable computational models. Using a multiplexed qHTS-based assay strategy, we profiled > 7600 unique environmental, industrial, and drug-like compounds for their ability to modulate hERG channel activity. Predicting changes in hERG activity is a critical first cardiotoxicity screen, because chemicals that directly or indirectly alter hERG channel activity may affect cardiac action potential, cause QT prolongation, and lead to arrhythmia and/or the more fatal Torsade de pointes condition. The combination of evaluating each compound in a 15-point concentration-response, in triplicate, provided a robust screening dataset, which in turn allowed us to identify > 500 compounds that inhibited hERG channel activity based on thallium flux in U2OS cells. Additionally, we characterized chemical structural features that were frequently related to an alteration in hERG channel activity and constructed several computational models to predict new chemical structures capable of disrupting hERG activity. This work may serve as the foundation for a tiered approach for selecting compounds for more costly, lower throughput mechanistic studies.

## Figures and Tables

**Table 1:** Assay results summary: number of active antagonist chemicals sequentially selected in each step.

	Filtering Step					% of Active Chemicals
Filtering Step Applied Sequentially	Initial Outcome	Curve Rank (−9 to −5)	Efficacy (>30%)	Manual Activity Curve Check	Chemical Standardization	
Count of active chemicals	896	655	655	647	549	7.78%

**Table 2:** Performance of QSAR classification models based on the Tox21 dataset. Each model building process was repeated 5 times, each with distinct data splitting. The mean and standard deviation of each performance criterion are reported: Q, accuracy; Qb, balanced accuracy; Sp, specificity; Se, sensitivity; and MCC, Matthew coefficient correlation.

**10-Fold Cross-Validation (for five Undersampled Training Set, *n* = 2023)**					
	**Q**	**Qb**	**Sp**	**Se**	**MCC**
**CART**	0.88 (+/−0.005)	0.757 (+/−0.011)	0.948 (+/−0.003)	0.566 (+/−0.018)	0.561 (+/−0.019)
**NN**	0.868 (+/−0.008)	0.75 (+/−0.058)	0.933 (+/−0.013)	0.566 (+/−0.103)	0.526 (+/−0.057)
**DNN**	0.88 (+/−0.005)	0.824 (+/−0.017)	0.927 (+/−0.007)	0.721 (+/−0.039)	0.659 (+/−0.021)
**SVM-linear**	0.896 (+/−0.004)	0.803 (+/−0.008)	0.947 (+/−0.004)	0.658 (+/−0.011)	0.629 (+/−0.013)
**SVM-radial**	0.913 (+/−0.004)	0.82 (+/−0.009)	0.964 (+/−0.002)	0.676 (+/−0.016)	0.685 (+/−0.016)
**SVM-sigmoid**	0.89 (+/−0.002)	0.758 (+/−0.007)	0.962 (+/−0.002)	0.553 (+/−0.012)	0.587 (+/−0.009)
**RF**	0.907 (+/−0.004)	0.795 (+/−0.006)	0.969 (+/−0.003)	0.621 (+/−0.009)	0.657 (+/−0.014)
**LDA**	0.895 (+/−0.002)	0.805 (+/−0.004)	0.944 (+/−0.002)	0.666 (+/−0.005)	0.629 (+/−0.008)

**Fitting (for five undersampled training set, *n* = 2023)**					
	**Q**	**Qb**	**Sp**	**Se**	**MCC**
**CART**	0.914 (+/−0.002)	0.86 (+/−0.011)	0.961 (+/−0.005)	0.759 (+/−0.017)	0.751 (+/−0.005)
**NN**	0.895 (+/−0.01)	0.852 (+/−0.029)	0.931 (+/−0.016)	0.773 (+/−0.041)	0.704 (+/−0.028)
**DNN**	0.983 (+/−0.005)	0.975 (+/−0.005)	0.927 (+/−0.007)	0.962 (+/−0.014)	0.951 (+/−0.014)
**SVM-linear**	0.921 (+/−0.004)	0.881 (+/−0.008)	0.955 (+/−0.004)	0.806 (+/−0.012)	0.773 (+/−0.011)
**SVM-radial**	0.972 (+/−0.008)	0.958 (+/−0.015)	0.983 (+/−0.002)	0.933 (+/−0.028)	0.92 (+/−0.023)
**SVM-sigmoid**	0.884 (+/−0.004)	0.805 (+/−0.008)	0.952 (+/−0.005)	0.658 (+/−0.011)	0.657 (+/−0.011)
**RF**	0.997(+/−0.002)	0.996 (+/−0.004)	0.998 (+/−0.001)	0.993 (+/−0.006)	0.99 (+/−0.005)
**LDA**	0.902 (+/−0.005)	0.849 (+/−0.006)	0.948 (+/−0.005)	0.749 (+/−0.006)	0.718 (+/−0.013)

**External validation (test set, *n* = 1072)**					
	**Q**	**Qb**	**Sp**	**Se**	**MCC**
**CART**	0.898 (+/−0.005)	0.775 (+/−0.016)	0.92 (+/−0.006)	0.629 (+/−0.026)	0.447 (+/−0.016)
**NN**	0.893 (+/−0.008)	0.791 (+/−0.019)	0.911 (+/−0.009)	0.671 (+/−0.028)	0.456 (+/−0.021)
**DNN**	0.913 (+/−0.007)	0.812 (+/−0.027)	0.931 (+/−0.006)	0.693 (+/−0.052)	0.517 (+/−0.041)
**SVM-linear**	0.906 (+/−0.005)	0.802 (+/−0.012)	0.925 (+/−0.006)	0.678 (+/−0.017)	0.492 (+/−0.014)
**SVM-radial**	0.929 (+/−0.004)	0.818 (+/−0.011)	0.948 (+/−0.003)	0.688 (+/−0.018)	0.563 (+/−0.02)
**SVM-sigmoid**	0.92 (+/−0.004)	0.784 (+/−0.005)	0.944 (+/−0.004)	0.624 (+/−0.005)	0.505 (+/−0.012)
**RF**	0.928 (+/−0.004)	0.814 (+/−0.021)	0.948 (+/−0.005)	0.68 (+/−0.037)	0.557 (+/−0.019)
**LDA**	0.909 (+/−0.007)	0.795 (+/−0.015)	0.93 (+/−0.006)	0.659 (+/−0.024)	0.491 (+/−0.028)

**Table 3:** Performance of generated QSAR classification models built using the enriched set. Q, accuracy; Qb, balanced accuracy; Sp, specificity; Se, sensitivity; and MCC, Matthew coefficient correlation.

**10-Fold Cross-Validation (Full Training Set, *n* = 7064)**					
	**Q**	**Qb**	**Sp**	**Se**	**MCC**
**CART**	0.921	0.887	0.822	0.951	0.779
**NN**	0.890	0.826	0.707	0.947	0.684
**DNN**	0.941	0.917	0.962	0.873	0.836
**SVM-linear**	0.945	0.924	0.885	0.963	0.847
**SVM-radial**	0.953	0.932	0.893	0.972	0.870
**SVM-sigmoid**	0.939	0.916	0.873	0.959	0.831
**RF**	0.951	0.925	0.875	0.975	0.863
**LDA**	0.938	0.911	0.861	0.962	0.828

**Fitting (training set, *n* = 7064)**					
	**Q**	**Qb**	**Sp**	**Se**	**MCC**
**CART**	0.930	0.900	0.845	0.956	0.805
**NN**	0.935	0.922	0.897	0.947	0.826
**DNN**	0.930	0.984	0.994	0.974	0.971
**SVM-linear**	0.952	0.932	0.895	0.970	0.867
**SVM-radial**	0.981	0.970	0.951	0.990	0.946
**SVM-sigmoid**	0.941	0.920	0.881	0.960	0.838
**RF**	0.999	0.998	0.996	0.999	0.997
**LDA**	0.939	0.913	0.863	0.963	0.831

**External validation (test set, *n* = 1247)**					
	**Q**	**Qb**	**Sp**	**Se**	**MCC**
**CART**	0.929	0.904	0.858	0.951	0.804
**NN**	0.929	0.917	0.895	0.939	0.810
**DNN**	0.933	0.909	0.956	0.861	0.816
**SVM-linear**	0.949	0.926	0.882	0.970	0.857
**SVM-radial**	0.958	0.937	0.895	0.978	0.884
**SVM-sigmoid**	0.942	0.924	0.889	0.959	0.842
**RF**	0.949	0.926	0.882	0.970	0.857
**LDA**	0.937	0.906	0.848	0.964	0.823

**Table 4:** Details of the Top 10 descriptors from the best RF models, including average (M) of the descriptor values for each group of active and inactive chemicals, with an associated *p*-value significance (*** < 0.005). Student-test or Wilcoxon test (if descriptor distribution was not normal) were applied.

Descriptor	Description	M Active	M Inactive	*p*-Value
Physicochemical descriptors				
BP_pred	Boiling point prediction	358.27	279.15	***
LogKoc_pred	Log of soil adsorption coefficient of organic compounds. The ratio of the amount of chemical adsorbed per unit weight of organic carbon in the soil or sediment to the concentration of the chemical in solution at equilibrium.	3.56	2.53	***
MolLogP2	Crippen method to estimate log(P)^2^	22.12	8.76	***
MolLogP	Crippen method to estimate log(P)	4.41	2.19	***
MOE type				
SMR_VSA3	MOE-type descriptors using molecular refractivity contributions and surface area contributions	13.05	3.29	***
Topological				
BalabanJ	Balaban’s J index (J)	1.52	2.65	***
Charge descriptor				
QNss	Sum of squares of charges on N atoms	0.16	0.1	***
QNmin	Most negative charge on N atoms	−0.32	−0.16	***
Burden descriptors				
bcutm2	Highest eigenvalue 2 for burden matrix/weighted by atomic masses	3.89	3.59	***
bcutm3	Highest eigenvalue 3 for burden matrix/weighted by atomic masses	1.73	1.37	***
bcutm5	Highest eigenvalue 5 for burden matrix/weighted by atomic masses	3.15	2.52	***
bcutm4	Highest eigenvalue 4 for burden matrix/weighted by atomic masses	3.36	2.85	***
bcutm11	Highest eigenvalue 11 for burden matrix/weighted by atomic masses	1.73	1.37	***
Composition descriptor				
HeavyAtomCount	Count of heavy atom	25.14	16.18	***
ArBoundCount	Count of aromatic bonds	15.74	5.84	***

**Table 5:** Performance of the RF and DNN QSAR classification models on the external test sets. Consensus models built using the average probability of each active chemical.

	**PubChem (ID: AID588834) (135 Actives and 876 Inactives)**				
**TOX21 Model**	**Q**	**Qb**	**Sp**	**Se**	**MCC**
**DNN**	0.665	0.676	0.661	0.690	0.245
**RF**	0.920	0.793	0.966	0.619	0.631
**Consensus**	0.873	0.788	0.904	0.673	0.518
**TOX21-ChEMBL**					
**DNN**	0.693	0.703	0.690	0.717	0.286
**RF**	0.926	0.766	0.984	0.549	0.641
**Combined (RF + DNN)**	0.900	0.791	0.945	0.637	0.582
	**Lit-based hERG inhibitors (393 actives)**				
**TOX21 model**	**Q**		**TP**	**FN**	
**DNN**	0.41		161	231	
**RF**	0.40		157	235	
**Combined (RF + DNN)**	0.40		157	235	
**TOX21-ChEMBL**					
**DNN**	0.389		153	249	
**RF**	0.341		134	258	
**Combined (RF + DNN)**	0.341		134	258	

## Data Availability

All data generated or analyzed during this study are included in this article. Scripts used for this project are included in a GitHub repository available at https://github.com/ABorrel/cardiotox_hERG (latest accessed on 20 January 2022).

## Supplementary Materials

The following are available online at: https://www.mdpi.com/article/10.3390/biology11020209/s1, Figure S1: Correlation between pIC_50_ from the results using the NCATS assay with the pIC_50_ extracted on the same chemicals found in the ChEMBL library. Patch-clamp IC_50_ from ChEMBL are reported with a different color; Figure S2: Classification of active chemicals for the most populated chemical classes (from 80 classes). (A) Chemical counts for the 4950 chemicals classified from Tox21 chemicals library. (B) Top chemical classes with active chemicals for hERG; Figure S3: Scatter Plot (for a small subset of chemicals) between the pIC_50_ obtained in thallium flux assay from NCATS (yellow triangles) and patch-clamp assay from ChEMBL (blue circles); Figure S4: Correlation plot between the pIC_50_ on chemicals included in both the PubChem set (AID588834) and the Tox21 chemical library. Line in red shows the perfect correlation. The correlation between the two sets is reported in the figure and is equal to 0.86; Table S1: Parameters and hyperparameters screened for each machine learning developed. For the models built using the Tox21 library, an under-sampling was applied, and several combinations of parameters are reported; Table S2: performance of the SVM classification models on the external test sets. Supplementary data material File S1–S7 have been provided containing following information. Supplementary Material File S1: A .pdf file, including concentration-response curves for most active chemicals (IC50 < 1 µM) in blue and for the chemicals removed due to suspicious CR curve, in red and in green; Supplementary Material File S2: A .csv file containing all 7871 chemicals screened using the Tox21 chemical library for each of them with the −logIC50 and affinity classification (0: inactive and 1: active) are reported. The NA in the activity column (−log10(IC50)) stands for inconclusive chemicals; Supplementary Material File S3: A .csv file containing the descriptor table for the 7180 QSAR-ready structures of Tox21 chemical library screened; Supplementary Material File S4: A .csv file containing the activity (µM) of the enriched dataset (Tox21 and ChEMBL) for 8311 chemicals used for the QSAR modeling. The NA in the activity column stands for inactive chemicals; Supplementary Material File S5: A .csv file containing the descriptor table for the 8311 QSAR ready structures for enriched dataset (Tox21 chemical library and ChEMBL); Supplementary Material File S6: A .pdf file containing the structure of active chemicals ranked by activity and class; Supplementary Material File S7: A .csv file containing the enriched chemotypes found in active chemicals.
