# Supplementary material for: High-Throughput Chemical Screening and Structure-Based Models to Predict hERG Inhibition
Source: Biology (Basel). 2022 Jan 28;11(2):209. doi: 10.3390/biology11020209 (PMC8869358; doi:10.3390/biology11020209)
Supplement: Supplementary file 1 [file biology-11-00209-s001.zip › biology-1517439-supplementary.pdf]

Supplementary Materials:

High-Throughput Screening based clustering and QSAR to predict hERG inhibition.

Table S1: Parameters and hyperparameters screened for each machine learning developed. For the models built using the Tox21 library an under-sampling was applied, and several combinations of parameters are reported.

**Machine learning**	**Grid optimization methods and/or parameters screened**	**Parameter selected**	
		**Tox21 set (under sampling)**	**Enriched set**
Classification and Regression Trees (CART)	*rpart.control* function	*minsplot*:1<br>*maxsplit*: 30	*minsplot*:1<br>*maxsplit*: 30
Neural Network (NN)	*nnet* method<br>maxit: 75<br>*decay*: [0.1, 0.5, 1]<br>*vsize*: [1, 2, 5]	*vsize*: 2, 1<br>*decay*: 1, 1	*vsize*: 1<br>*decay*: 1
Deep Neural Network (DNN)	*kernel_inirialization*: random normal<br>*optimizer*: adam<br>*activation*: [relu, selu]<br>*epochs*: [50, 100, 120]<br>*batch_size*: [32, 64, 128]<br>*dense_layer*: [3, 4, 5]<br>*dense_candidate*: [50, 25, 20, 10, 1]	*activation*: selu, relu, relu, relu, selu<br>*epochs*: 50<br>*batch_size*: 128, 128, 64, 64, 64<br>*dense_layer*: 5, 4, 5, 4, 3<br>*dense_candidate*: 50	*activation*: selu<br>*epochs*: 50<br>*batch_size*: 128<br>*dense_layer*: 5<br>*dense_candidate*: 50
Support Vector Machine (SVM)-linear	*vcost:* [0.5, 1, 2]<br>*vgamma:* [0.1, 0.01, 0.001]	*vcost:* 0.5, 2, 1<br>*vgamma:* 0.1, 0.1, 0.1	*vcost:* 0.5<br>*vgamma:* 0.1
SVM- radial	*vcost:* [0.5, 1, 2]<br>*vgamma:* [0.1, 0.01, 0.001]	*vcost:* 2, 1<br>*vgamma:* 0.01, 0.01	*vcost:* 2<br>*vgamma:* 0.01
SVM-sigmoid	*vcost:* [0.5, 1, 2]<br>*vgamma:* [0.1, 0.01, 0.001]	*vcost:* 2<br>*vgamma:* 0.001	*vcost:* 0.5<br>*vgamma:* 0.1
Random Forest (RF)	*vntree:* [10, 50, 100, 200, 500]<br>*vmtry:* [1, 2, 3, 4, 5, 10, 15, 20, 25, 30]	*ntree*: 200, 50, 200<br>*mtry*: 15, 25, 30	*ntree*: 500<br>*mtry*: 30
Linear Discriminant Analysis (LDA)	-	-	-

Table S2: performance of the SVM classification models on the external test sets.

	**PubChem (ID: AID588834) (135 actives and 876 inactives)**				
**NCATS model**	**Q**	**Qb**	**Sp**	**Se**	**MCC**
SVM-linear	0.782	0.514	0.878	0.150	0.029
SVM-radial	0.793	0.517	0.891	0.142	0.035
**NCATS-ChEMBL**					
SVM-linear	0.801	0.529	0.898	0.159	0.062
SVM-radial	0.809	0.503	0.918	0.100	0.008
	**hERG inhibitors (398 actives)**				
**NCATS model**	**Q**		**TP**	**FN**	
SVM-linear	0.394		147	241	
SVM-radial	0.392		156	241	
**NCATS-ChEMBL**					
SVM-linear	0.386		154	244	
SVM-radial	0.342		136	262	

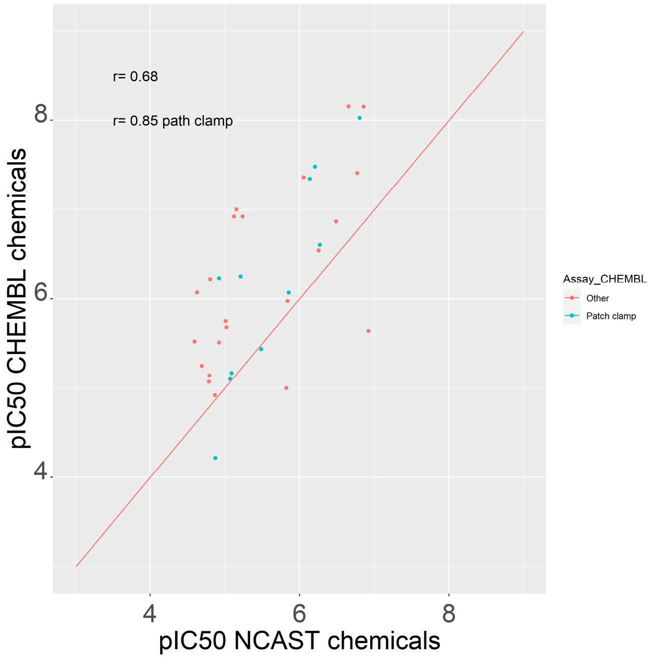

Figure S1: Correlation between pIC50 from the results using the NCATS assay with the pIC50 extracted on the same chemicals found in the ChEMBL library. Path-clamp IC50 from ChEMBL are reported with a different color.

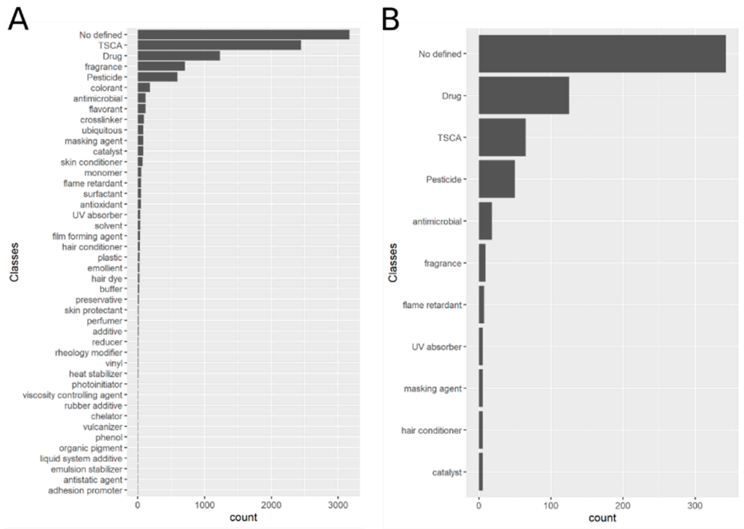

Figure S2: Classification of active chemicals for the most populated chemical classes (from 80 classes). (A) Chemical counts for the 4,950 chemicals classified from Tox21 chemicals library. (B) Top chemical classes with active chemicals for hERG.

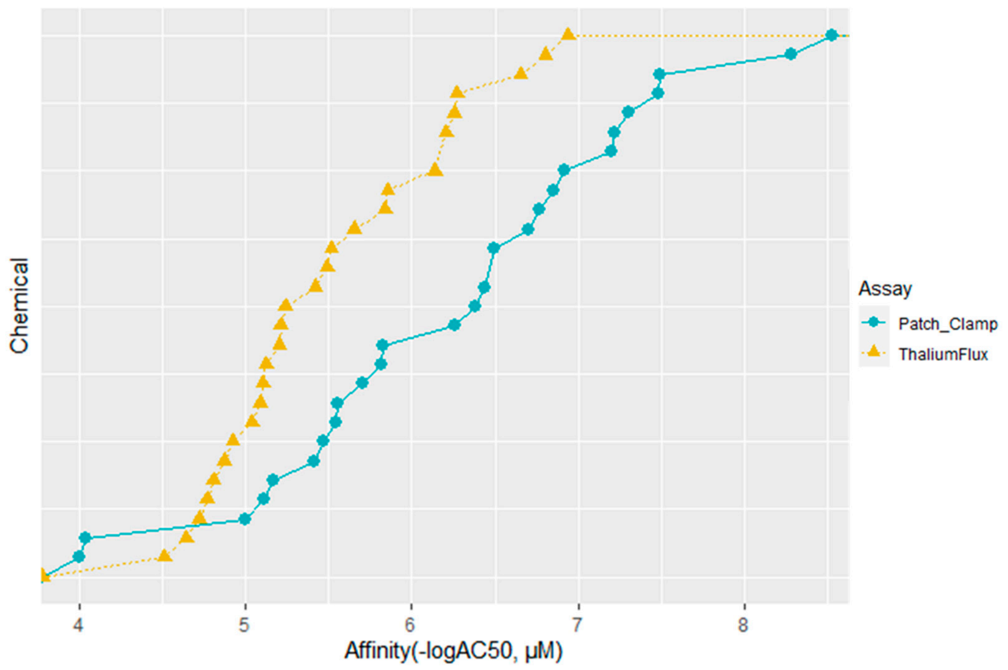

Figure S3: Scatter Plot (for a small subset of chemicals) between the pIC50 obtained in thallium flux assay from NCATS (yellow triangles) and Patch clamp assay from ChEMBL (blue circles).

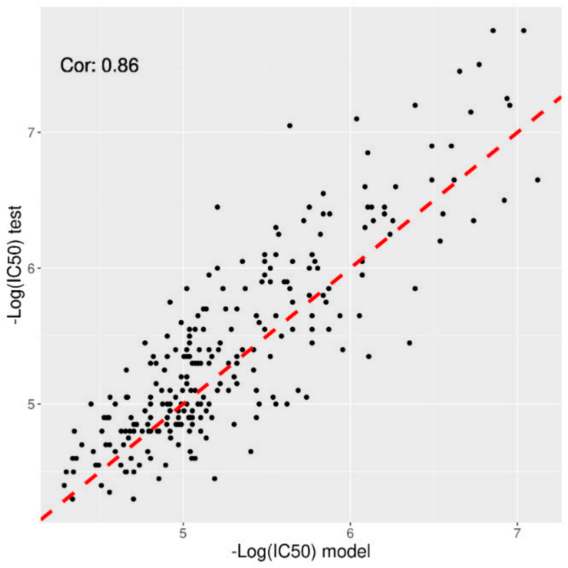

Figure S4: Correlation plot between the pIC50 on chemicals included in both the PubChem set (AID588834) and the Tox21 chemical library. Line in red shows the perfect correlation. The correlation between the two sets is reported on the figure and is equal to 0.86.
