# Supplementary material for: High-Throughput Chemical Screening and Structure-Based Models to Predict hERG Inhibition
Source: Biology (Basel). 2022 Jan 28;11(2):209. doi: 10.3390/biology11020209 (PMC8869358; doi:10.3390/biology11020209)
Supplement: Supplementary file 1 [file biology-11-00209-s001.zip › SupplementaryF.pdf]

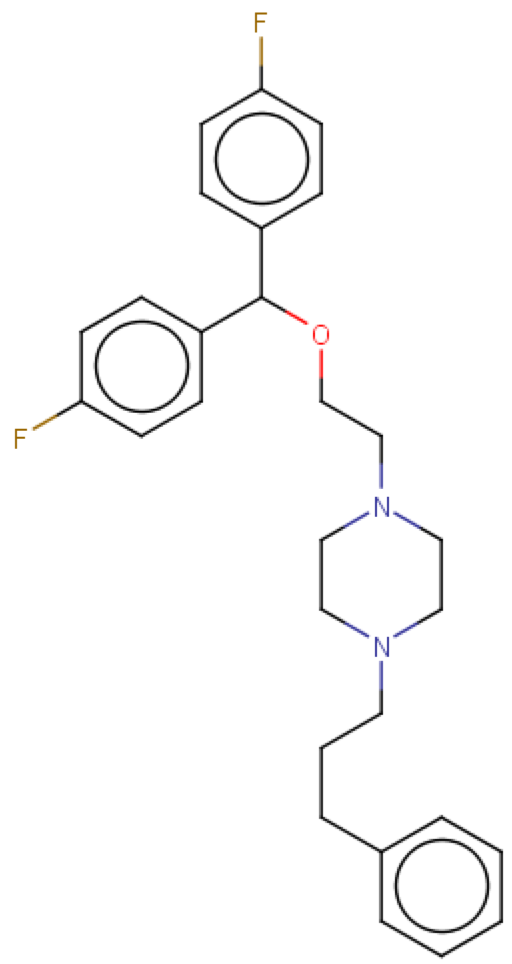

67469-78-7
Name: GBR 12909 dihydrochloride
pIC50: 7.12
Rank: 1
Classes: No defined

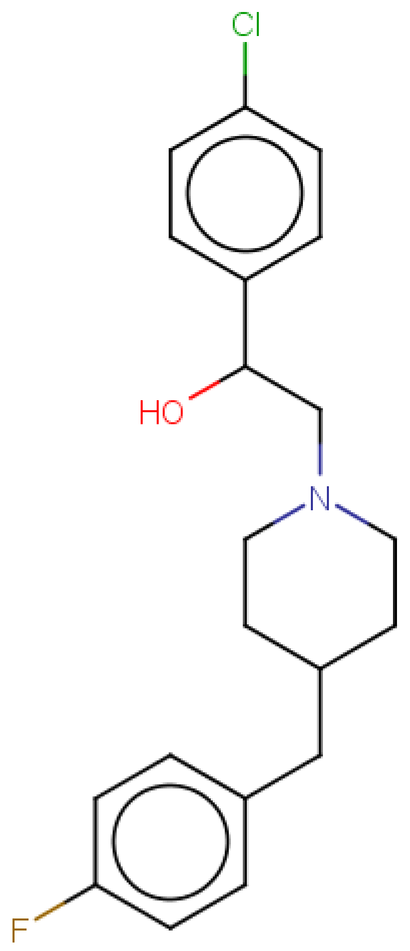

119431-25-3
Name: Eliprodil
pIC50: 7.04
Rank: 2
Classes: Drug

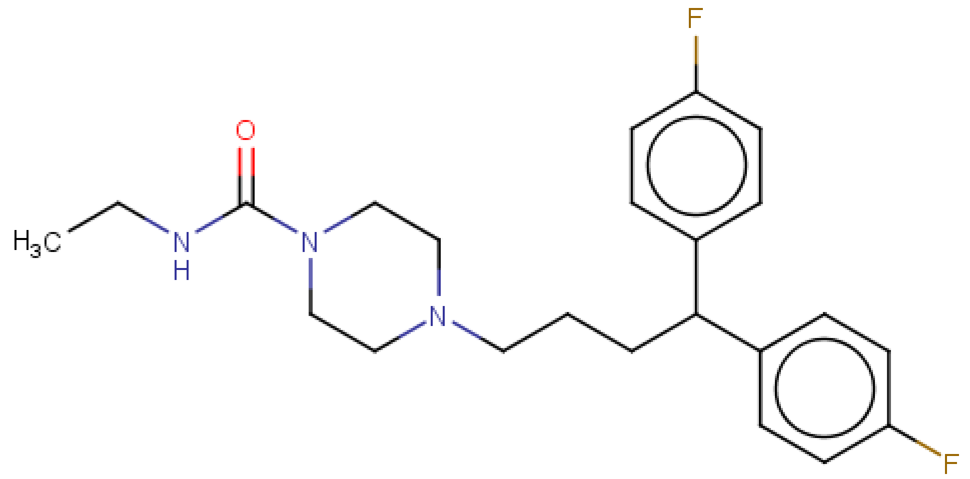

75529-73-6
Name: Amperozide hydrochloride
pIC50: 7.04
Rank: 3
Classes: No defined

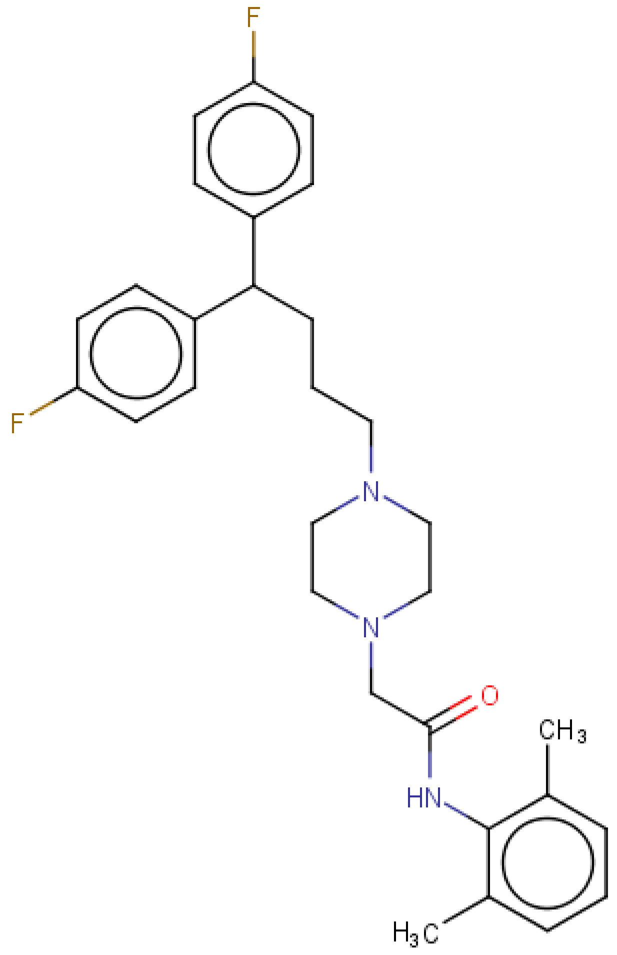

3416-26-0
Name: Lidoflazine
pIC50: 6.95
Rank: 4
Classes: No defined

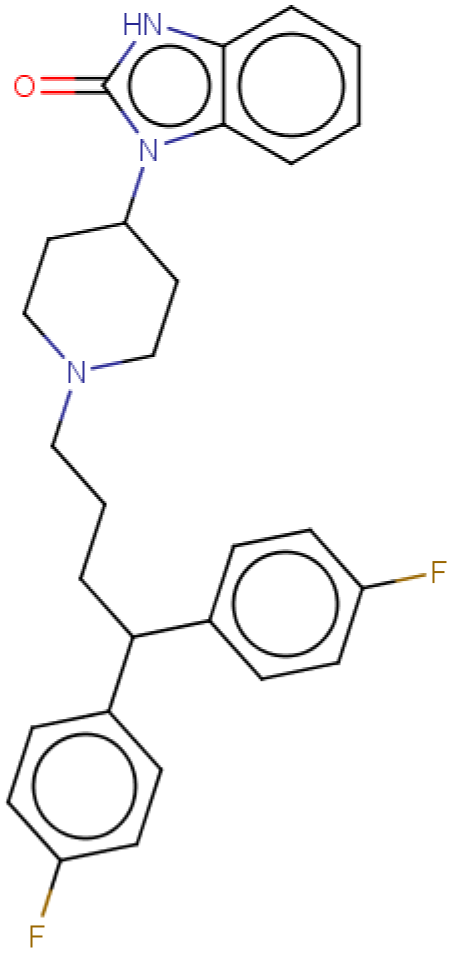

2062-78-4
Name: Pimozide
pIC50: 6.94
Rank: 5
Classes: Drug

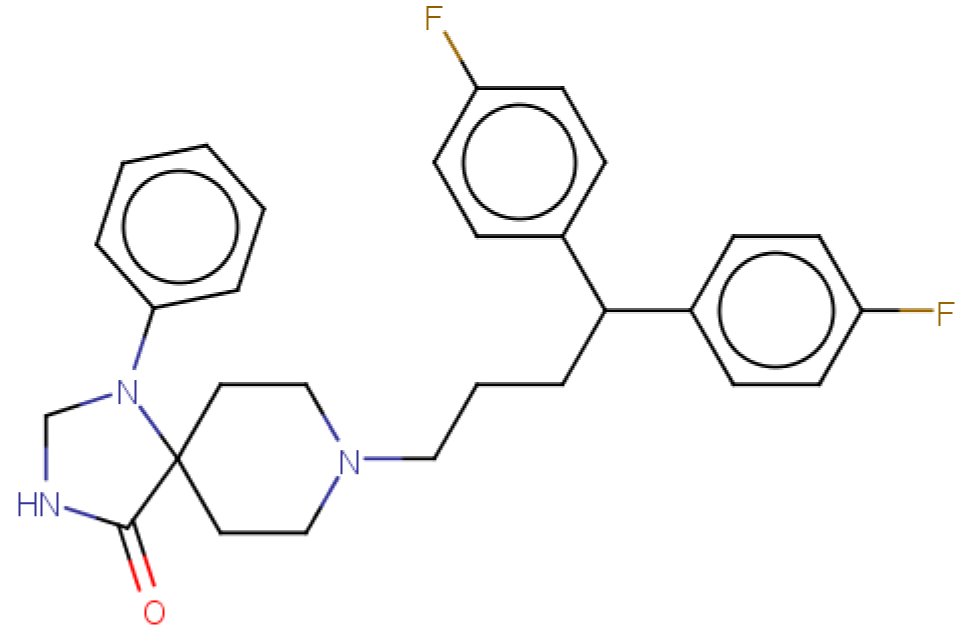

1841-19-6
Name: Fluspirilene
pIC50: 6.92
Rank: 6
Classes: Drug

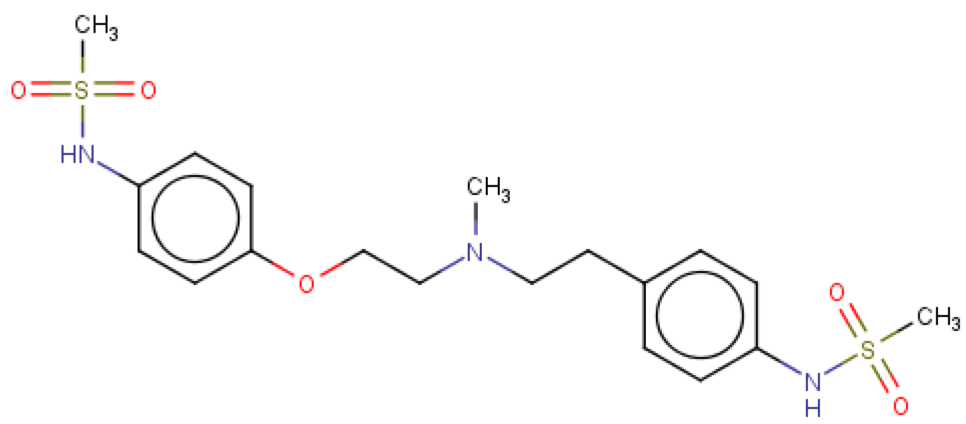

115256-11-6
Name: Dofetilide
pIC50: 6.85
Rank: 7
Classes: Drug

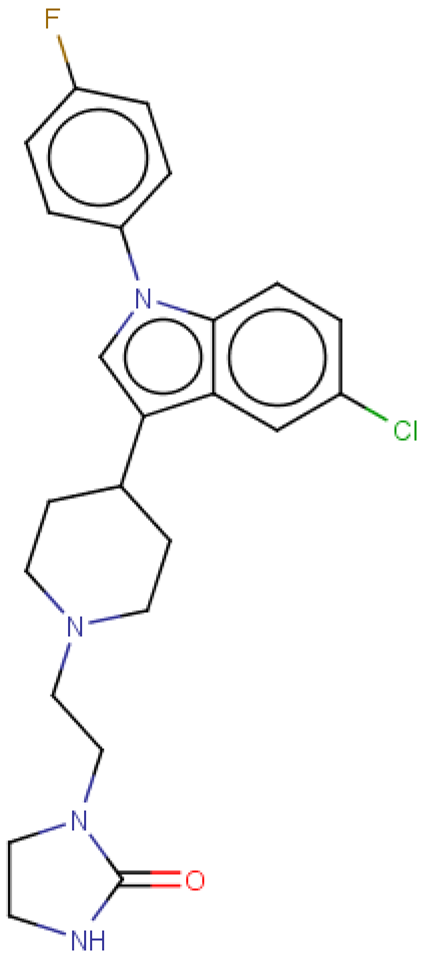

106516-24-9
Name: Sertindole
pIC50: 6.8
Rank: 8
Classes: Drug

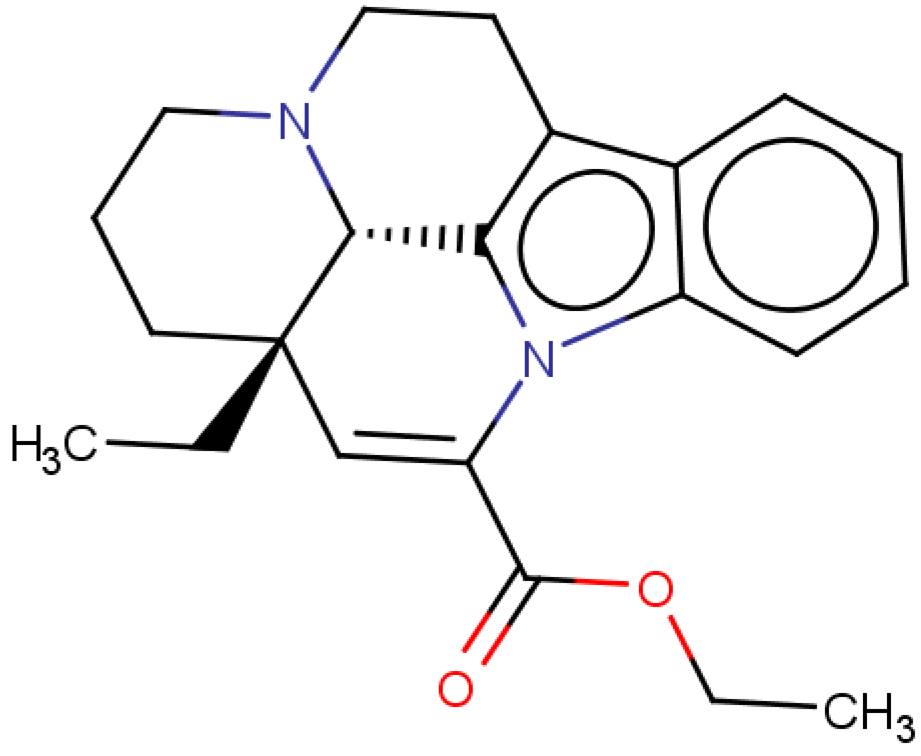

42971-09-5
Name: Vinpocetine
pIC50: 6.77
Rank: 9
Classes: Drug

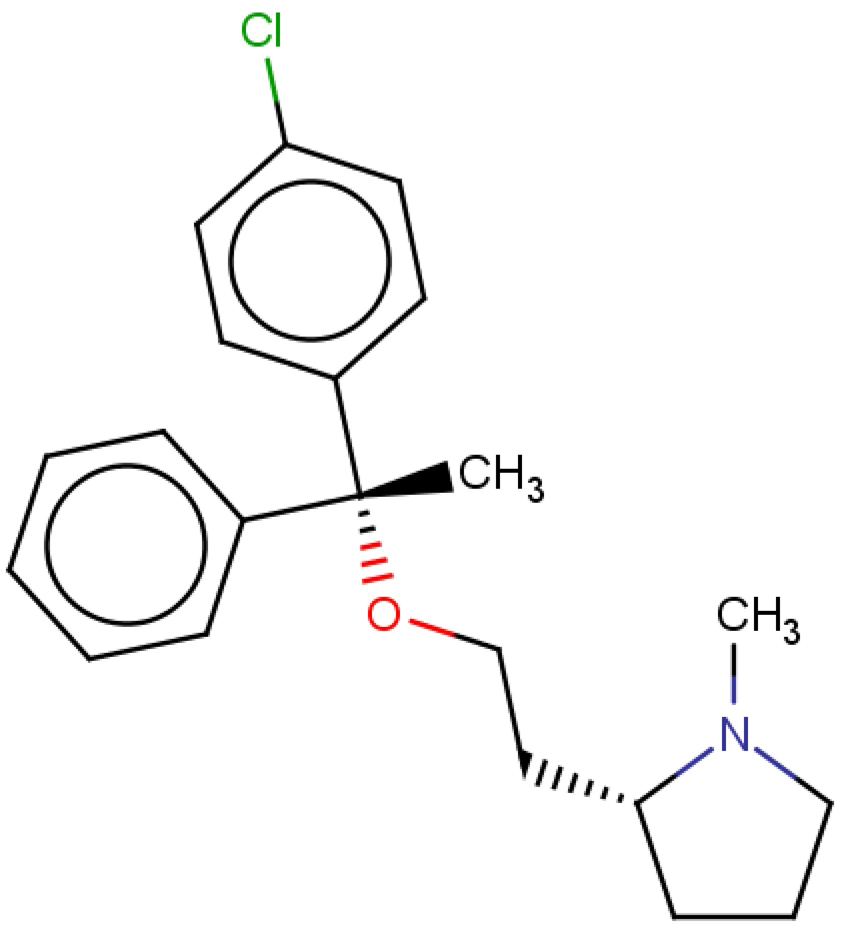

14976-57-9
Name: Clemastine fumarate
pIC50: 6.74
Rank: 10
Classes: No defined

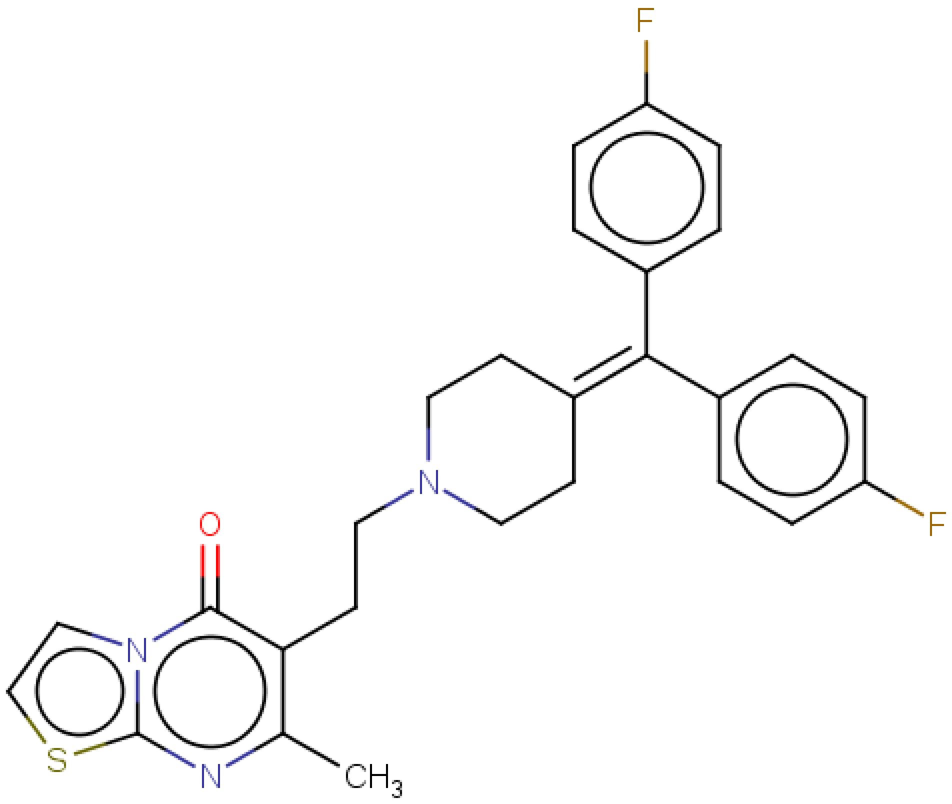

87051-43-2
Name: Ritanserin
pIC50: 6.72
Rank: 11
Classes: Drug

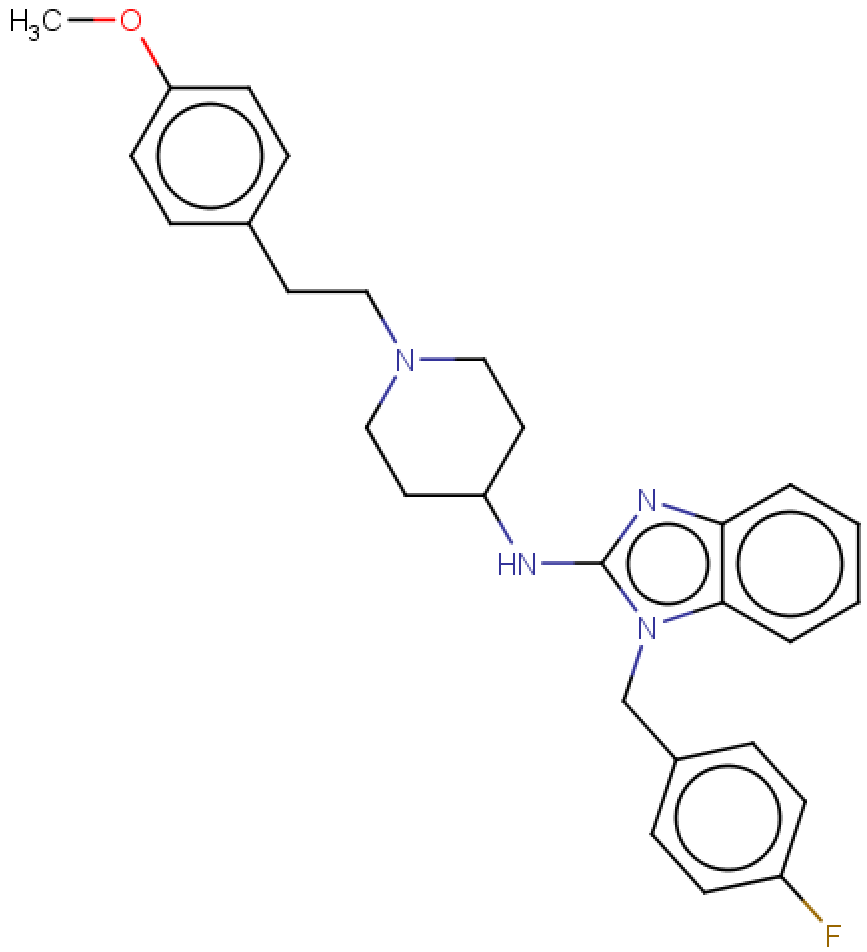

68844-77-9
Name: Astemizole
pIC50: 6.65
Rank: 12
Classes: Drug

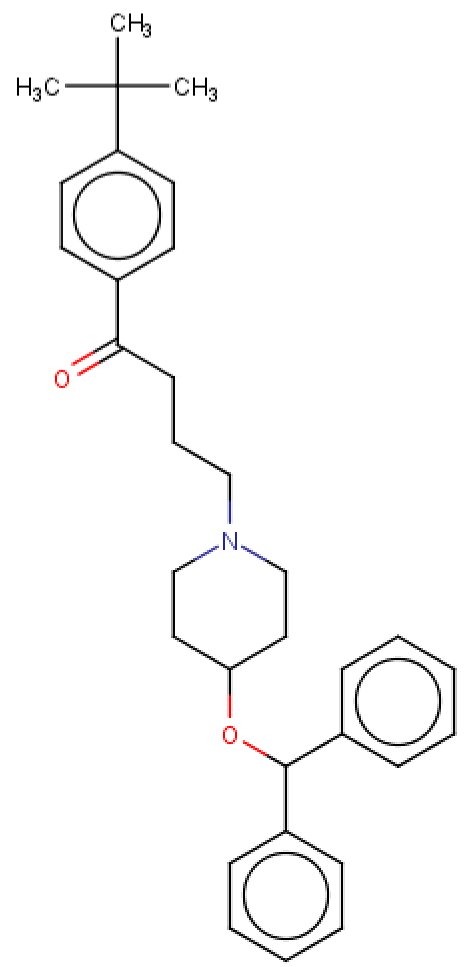

90729-43-4
Name: Ebastine
pIC50: 6.62
Rank: 13
Classes: Drug

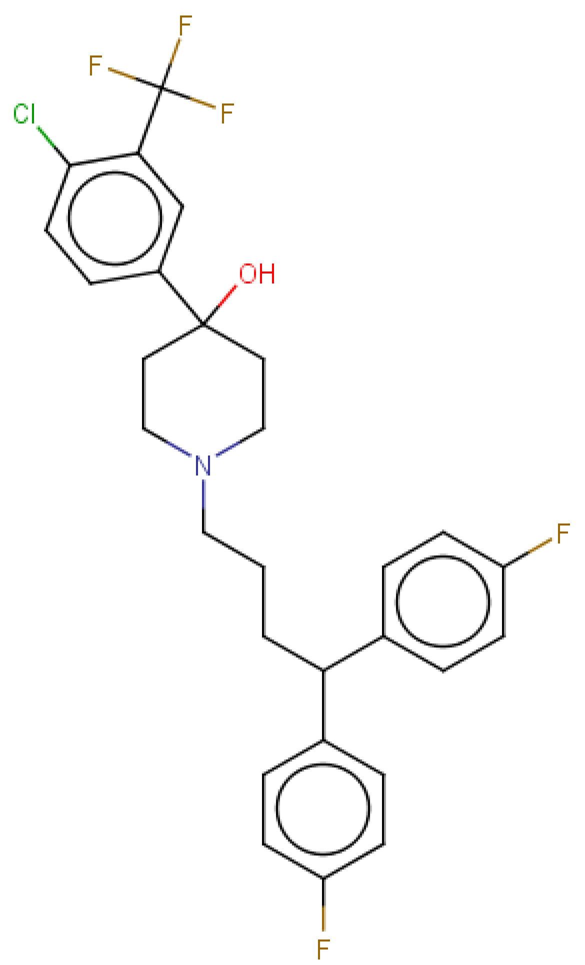

26864-56-2
Name: Penfluridol
pIC50: 6.62
Rank: 14
Classes: No defined

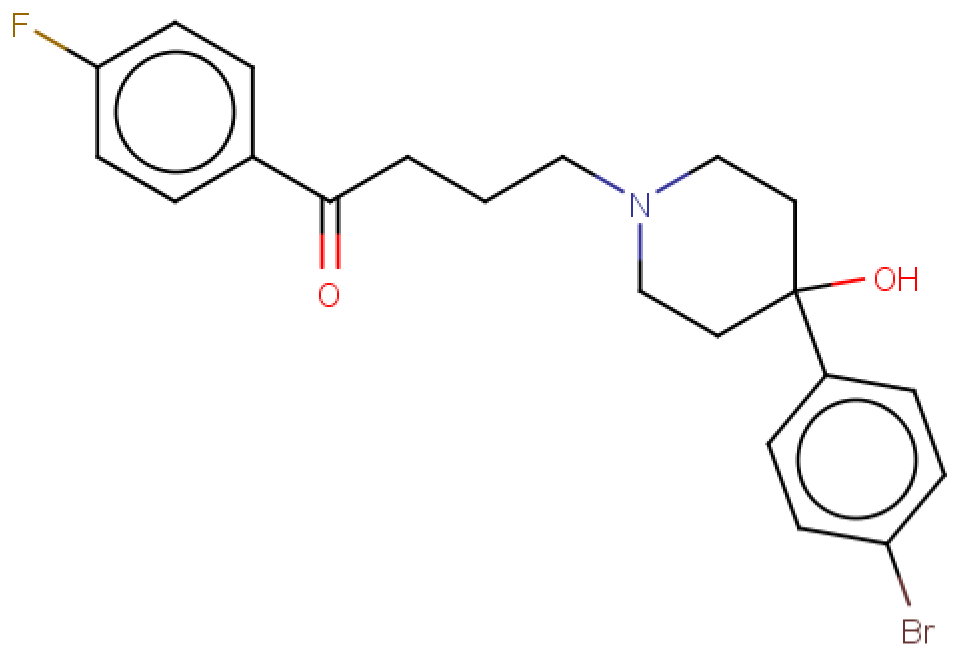

10457-90-6
Name: Bromperidol
pIC50: 6.6
Rank: 15
Classes: Drug

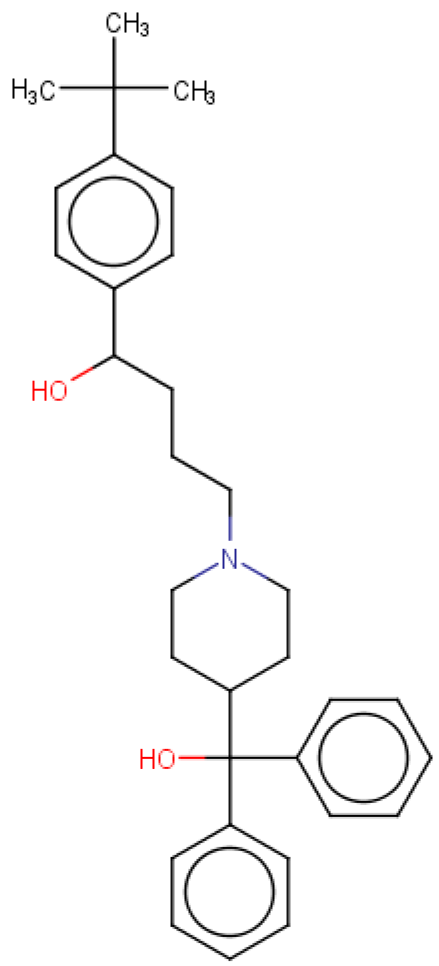

50679-08-8
Name: Terfenadine
pIC50: 6.55
Rank: 16
Classes: Drug

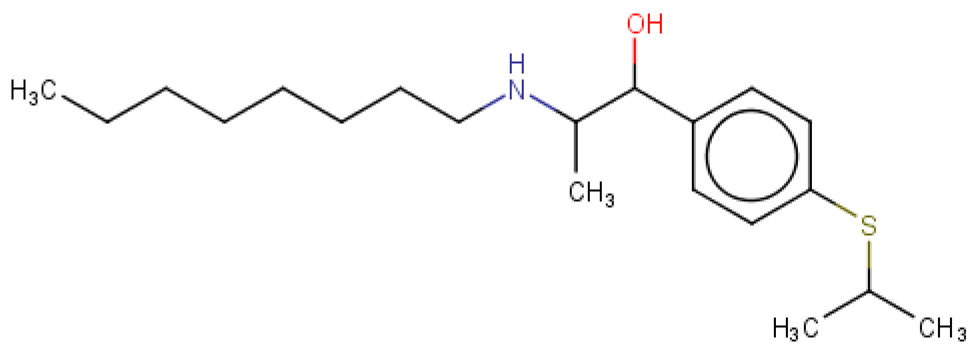

54767-75-8
Name: Suloctdil
pIC50: 6.54
Rank: 17
Classes: No defined

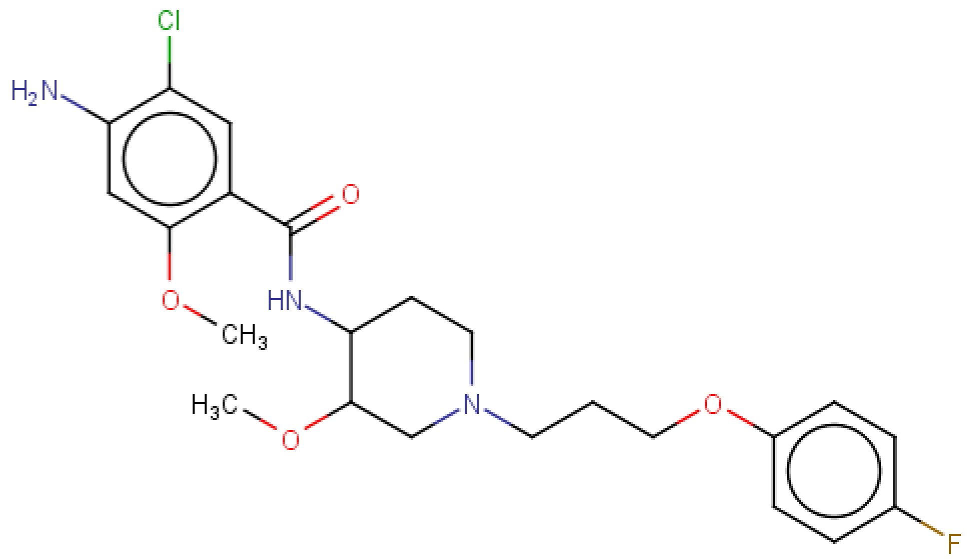

81098-60-4
Name: Cisapride
pIC50: 6.49
Rank: 18
Classes: Drug

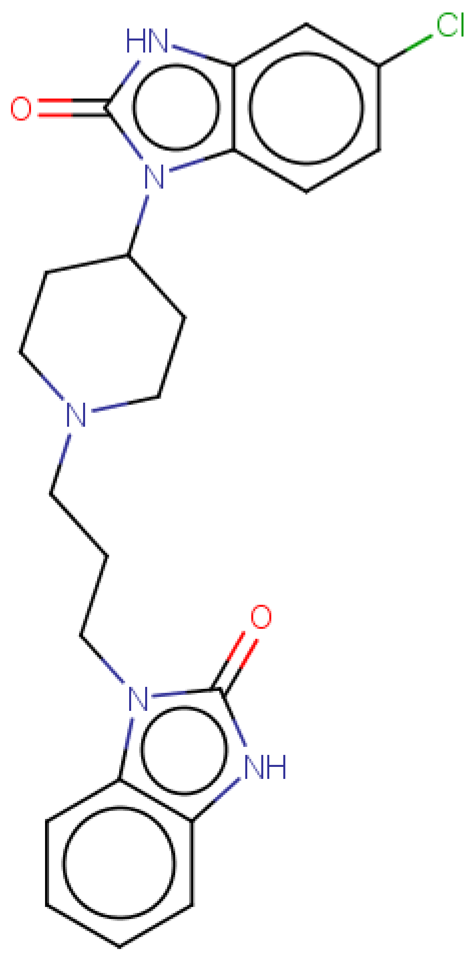

57808-66-9
Name: Domperidone
pIC50: 6.49
Rank: 19
Classes: Drug

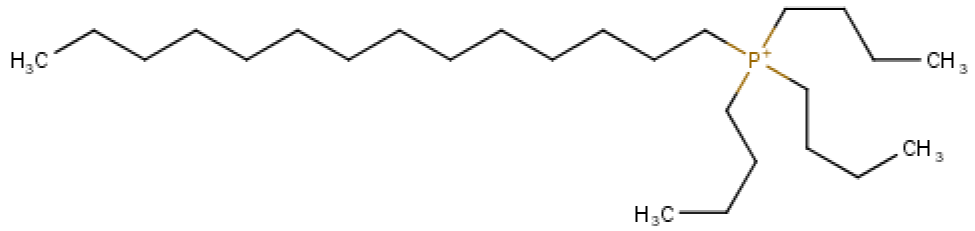

81741-28-8
Name: Tributyltetradecylphosphonium chloride
pIC50: 6.48
Rank: 20
Classes: catalyst

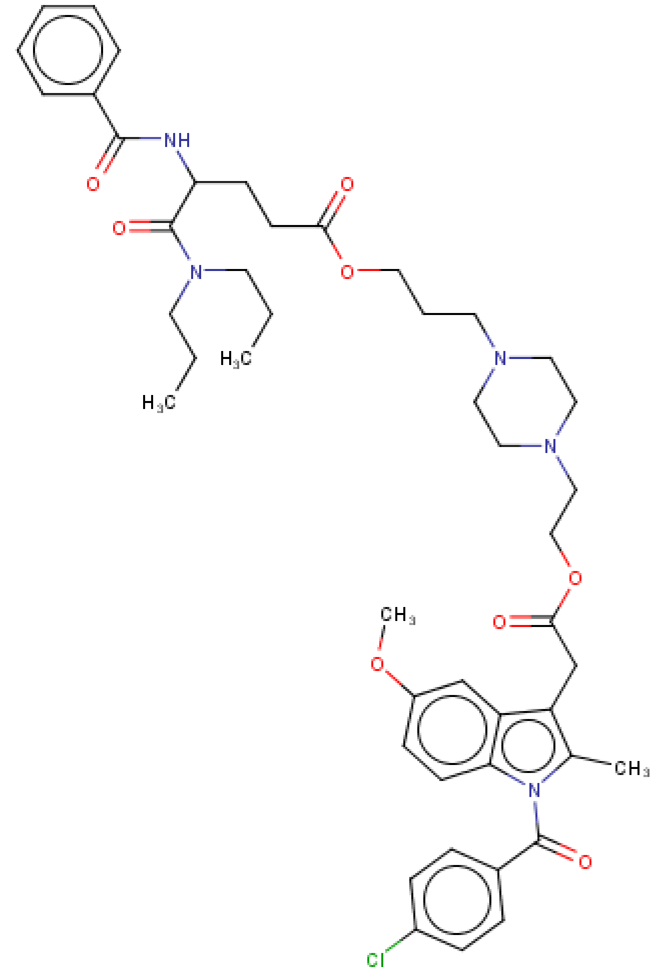

57132-53-3
Name: Proglumetacin
pIC50: 6.44
Rank: 21
Classes: No defined

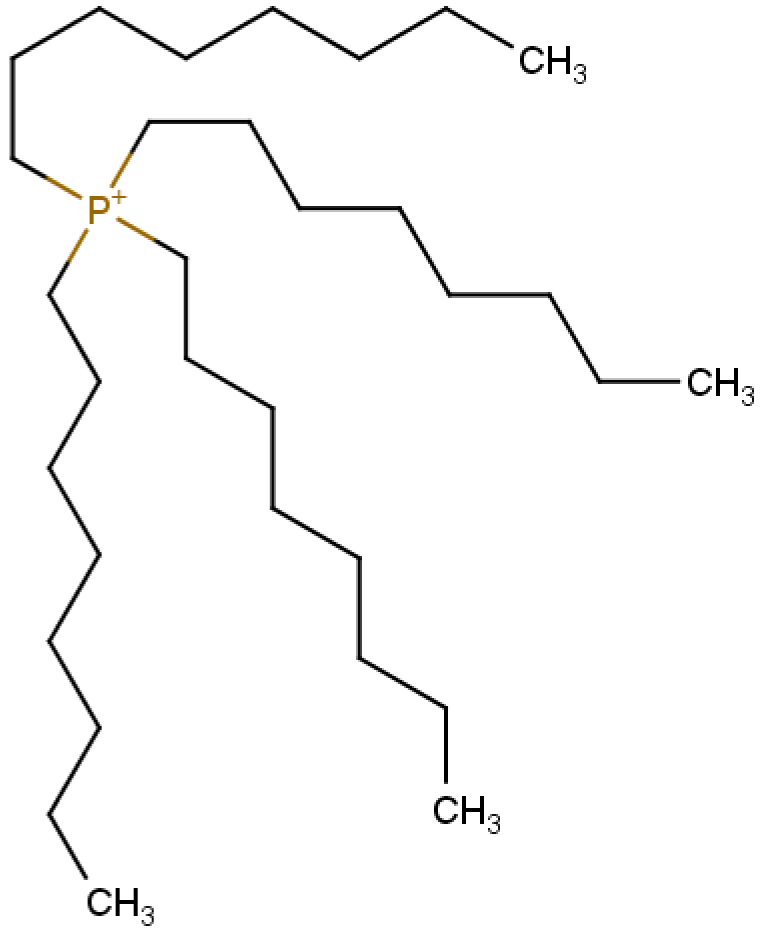

23906-97-0
Name: Tetraoctylphosphonium bromide
pIC50: 6.4
Rank: 22
Classes: No defined

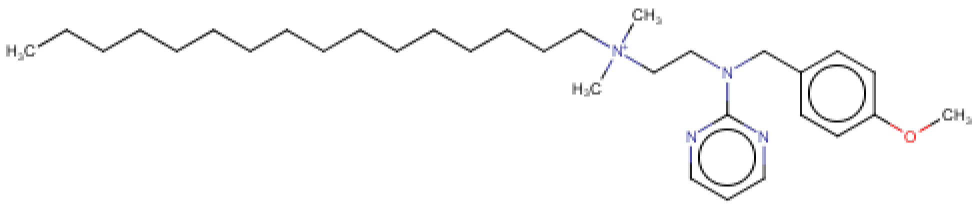

553-08-2
Name: Thonzonium bromide
pIC50: 6.39
Rank: 23
Classes: No defined

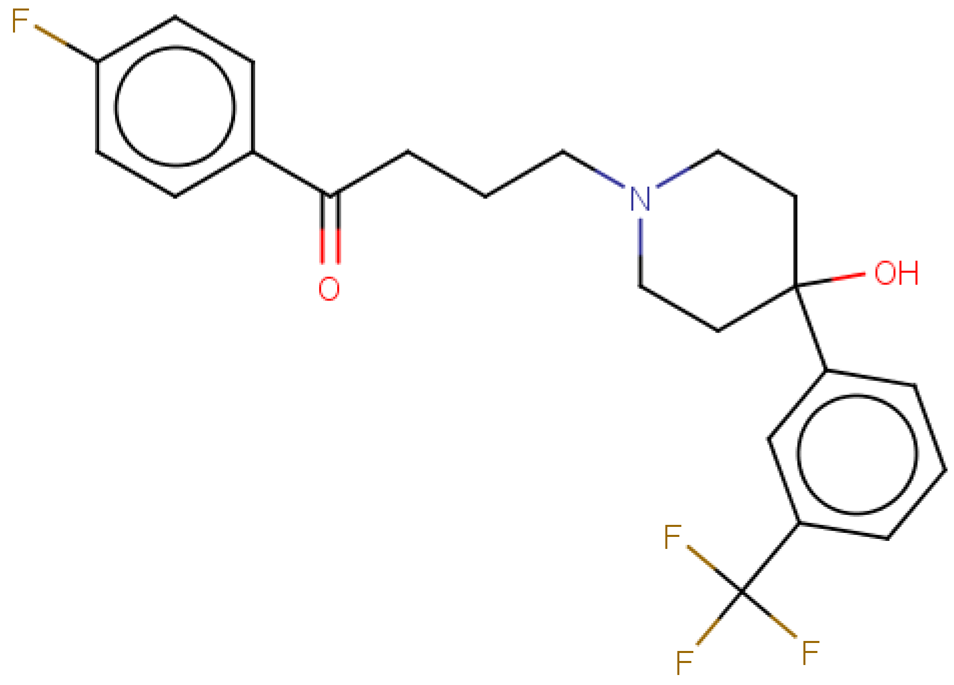

2062-77-3
Name: Trifluperidol hydrochloride
pIC50: 6.39
Rank: 24
Classes: No defined

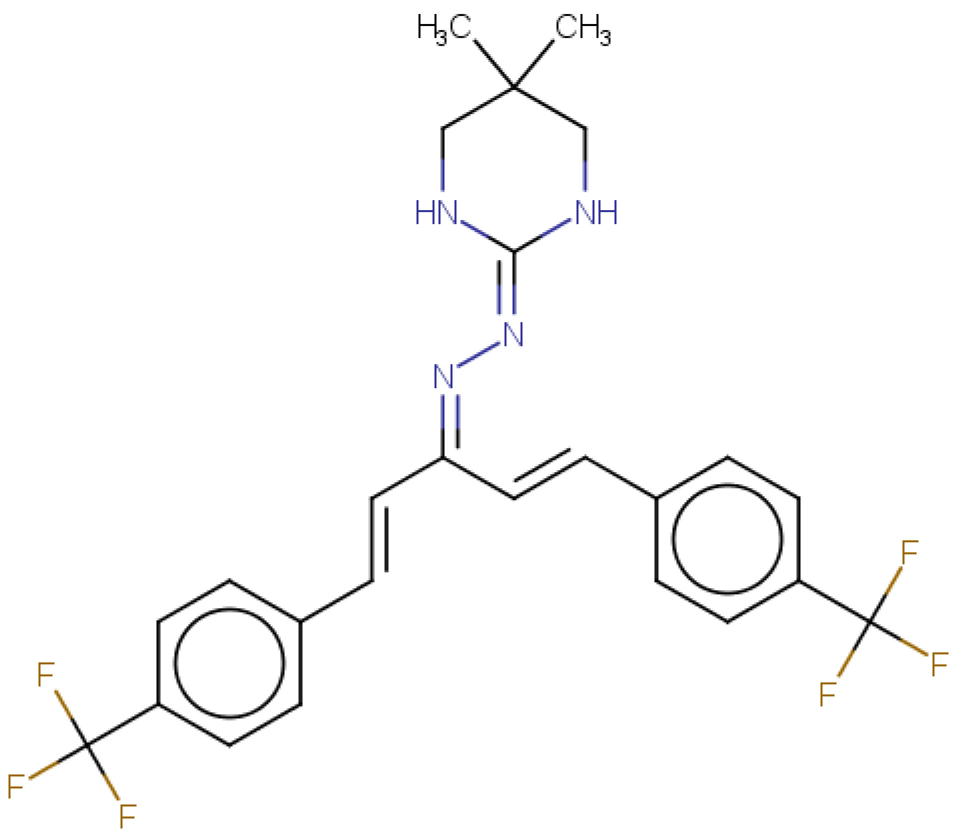

67485-29-4
Name: Hydramethylnon
pIC50: 6.36
Rank: 25
Classes: fragrance--Pesticide

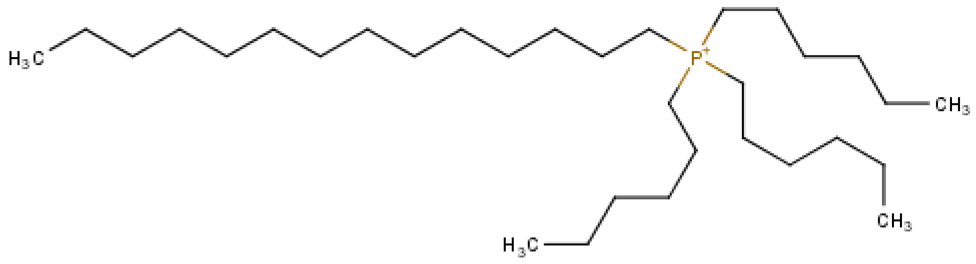

654057-97-3
Name: Trihexyltetradecylphosphonium bromide
pIC50: 6.36
Rank: 26
Classes: No defined

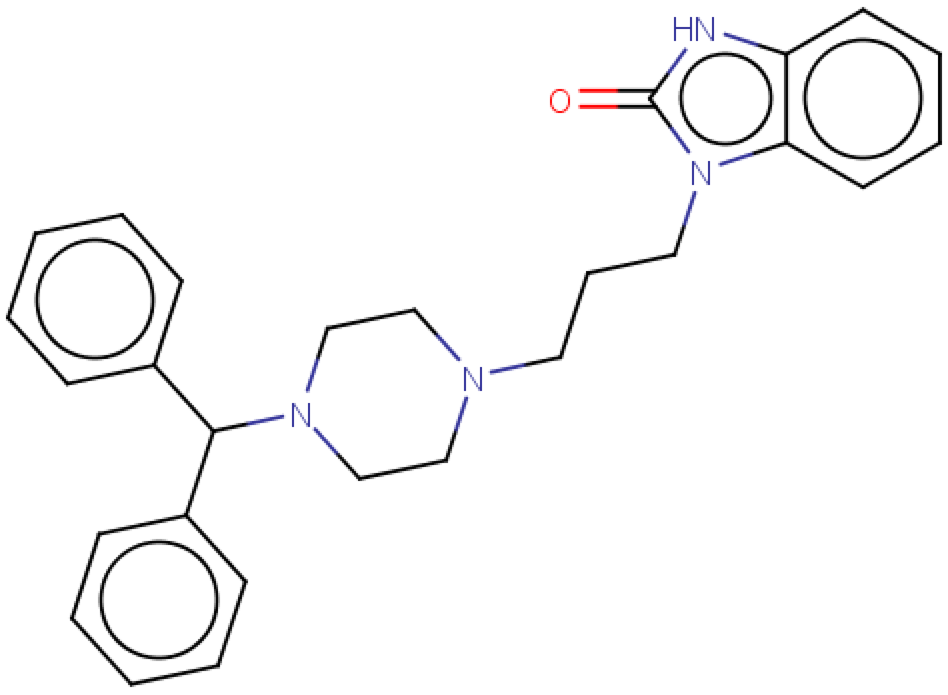

60607-34-3
Name: Oxatomide
pIC50: 6.35
Rank: 27
Classes: Drug

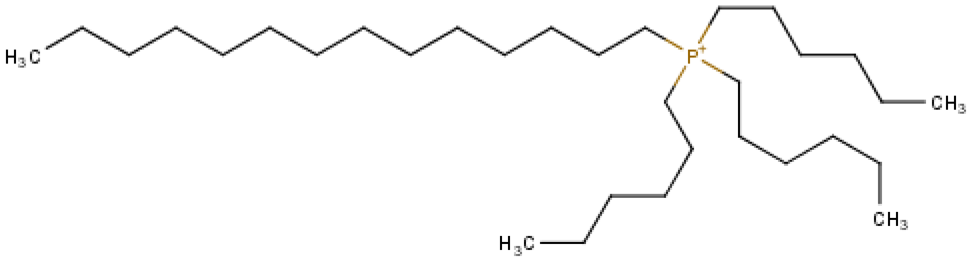

374683-44-0
Name: Trihexyltetradecylphosphonium hexafluorophosphate
pIC50: 6.33
Rank: 28
Classes: No defined

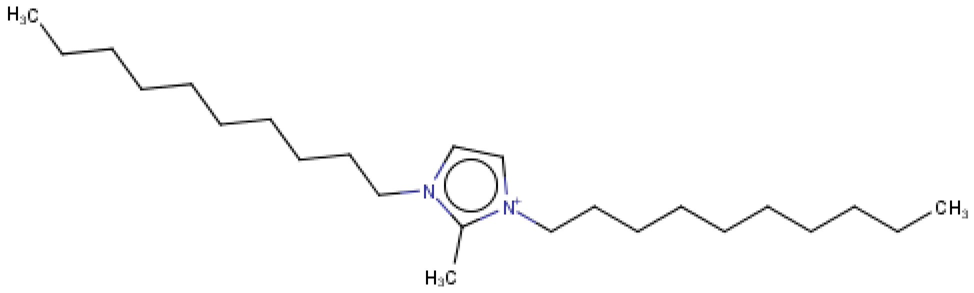

70862-65-6
Name: 1,3-Didecyl-2-methylimidazolium chloride
pIC50: 6.29
Rank: 29
Classes: No defined

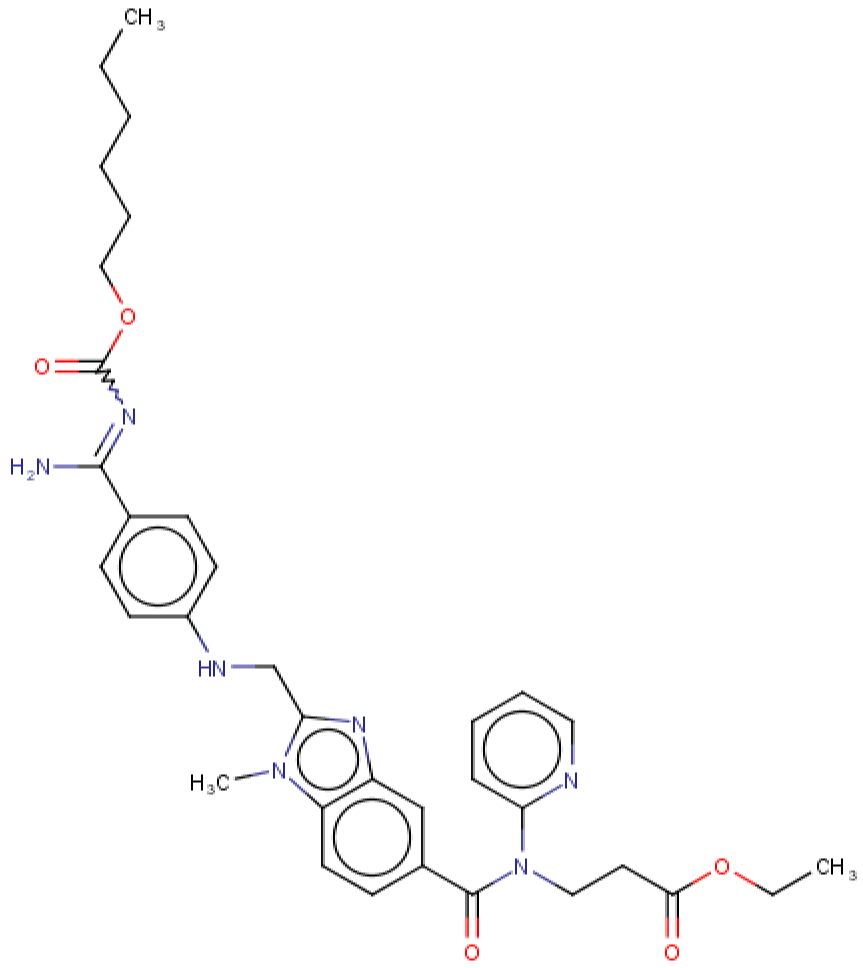

211915-06-9
Name: Dabigatran etexilate
pIC50: 6.29
Rank: 30
Classes: Drug

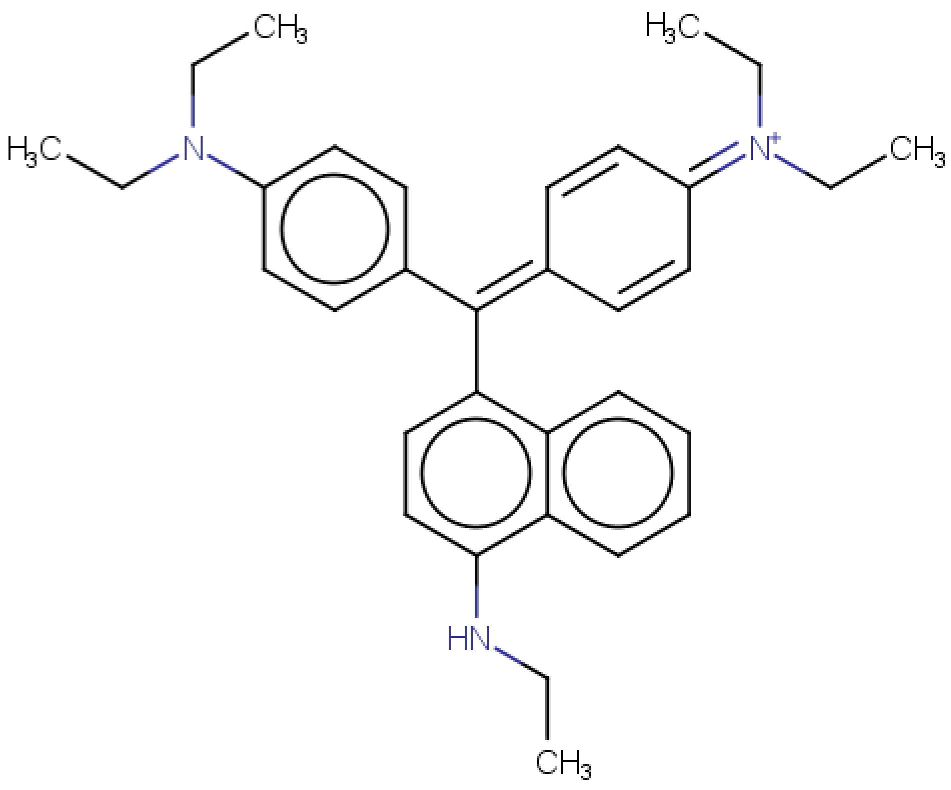

2390-60-5
Name: Basic Blue 7
pIC50: 6.28
Rank: 31
Classes: No defined

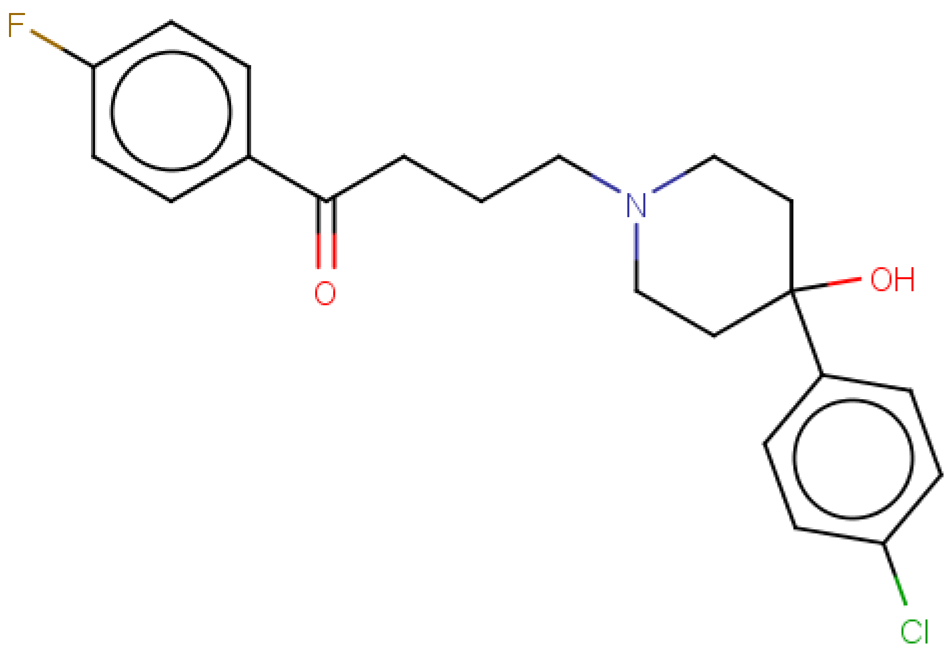

52-86-8
Name: Haloperidol
pIC50: 6.27
Rank: 32
Classes: Drug

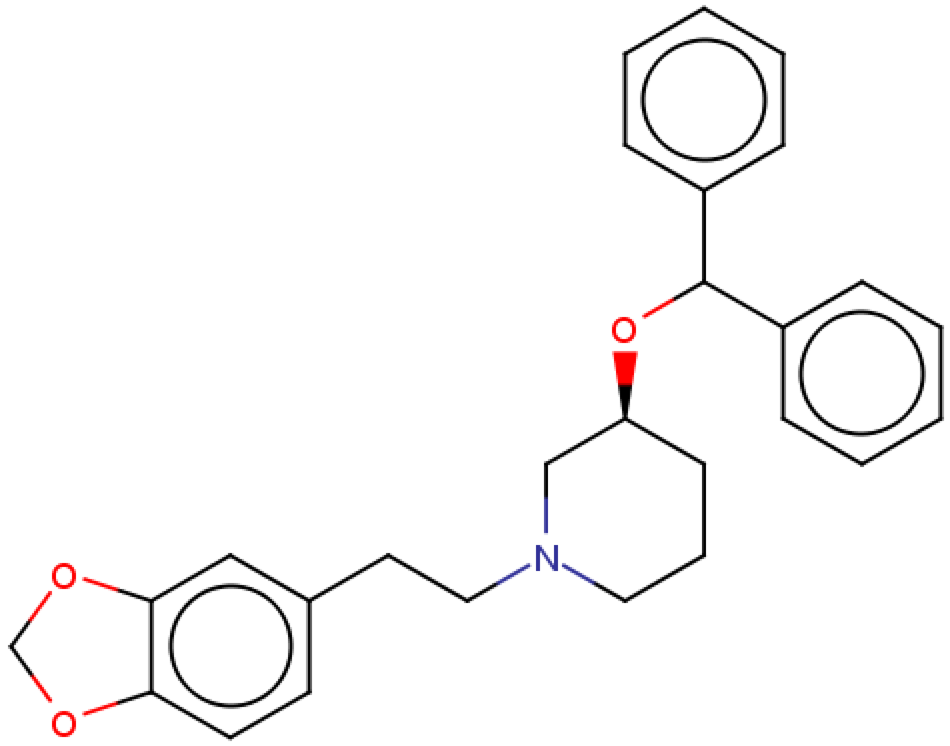

127308-98-9
Name: Zamifenacin fumarate
pIC50: 6.27
Rank: 33
Classes: No defined

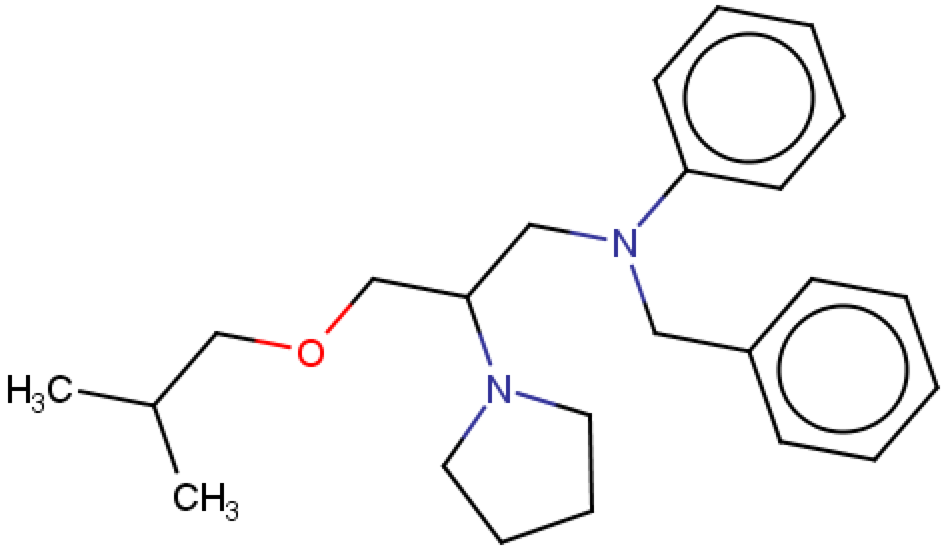

64706-54-3
Name: Bepridil
pIC50: 6.25
Rank: 34
Classes: Drug

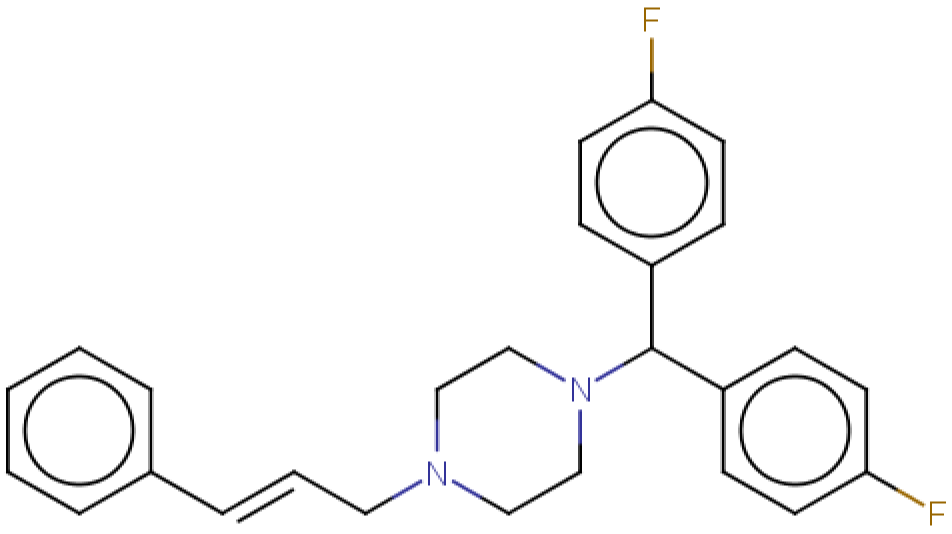

52468-60-7
Name: Flunarizine
pIC50: 6.25
Rank: 35
Classes: Drug

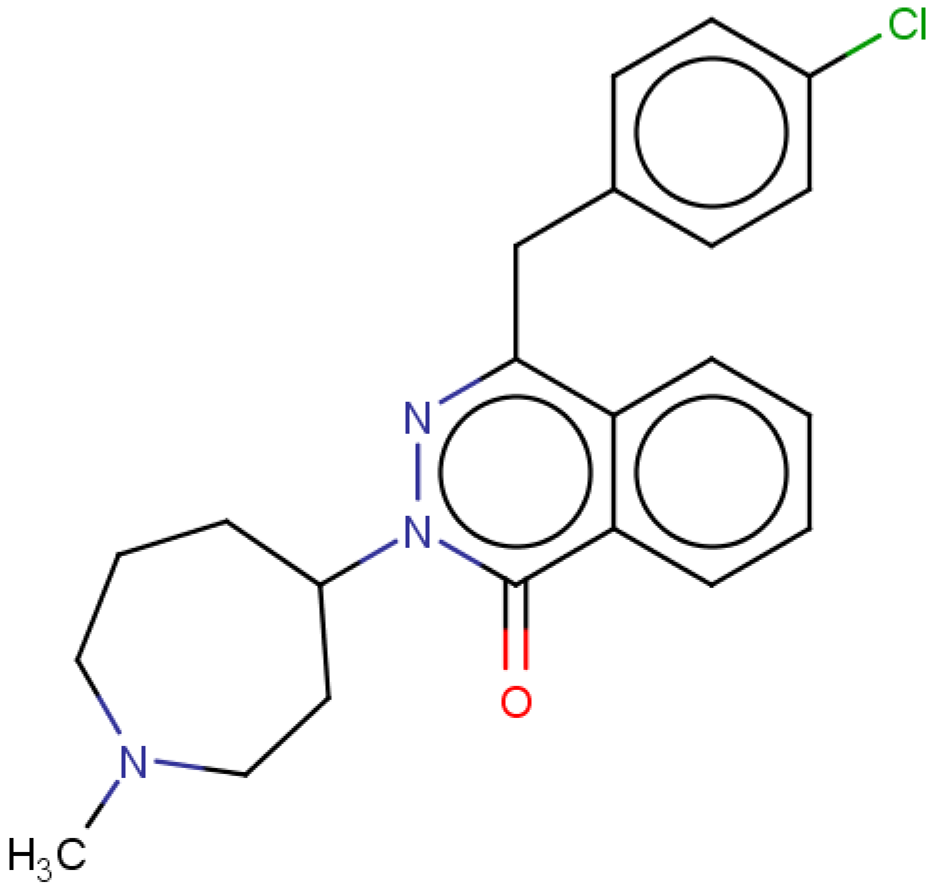

79307-93-0
Name: Azelastine hydrochloride
pIC50: 6.24
Rank: 36
Classes: No defined

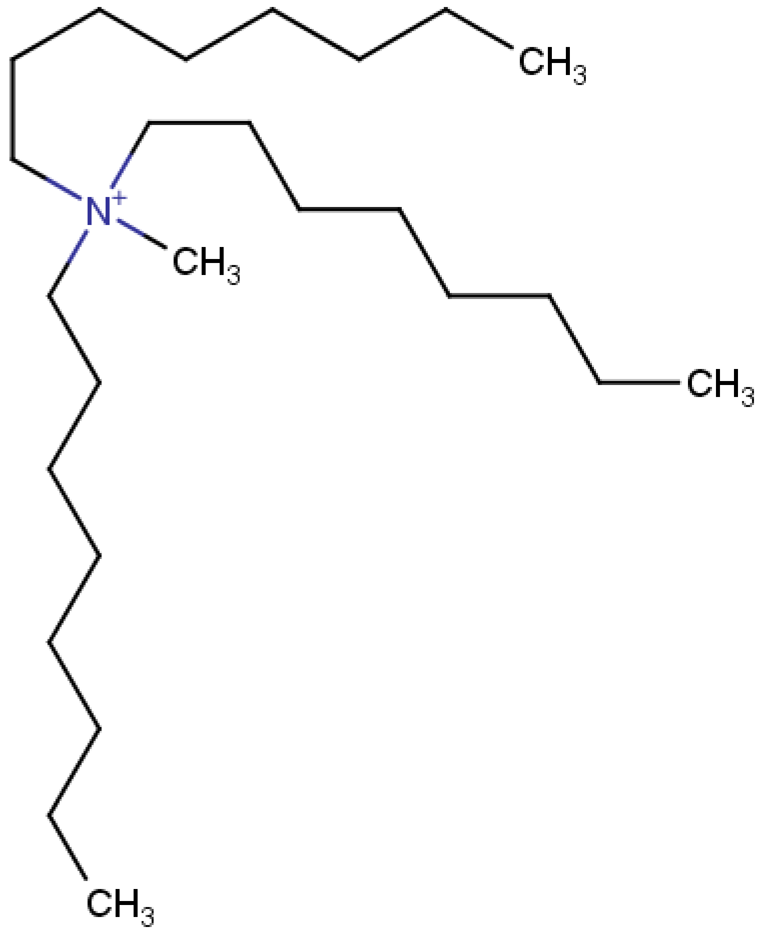

5137-55-3
Name: Methyltrioctylammonium chloride
pIC50: 6.21
Rank: 37
Classes: TSCA

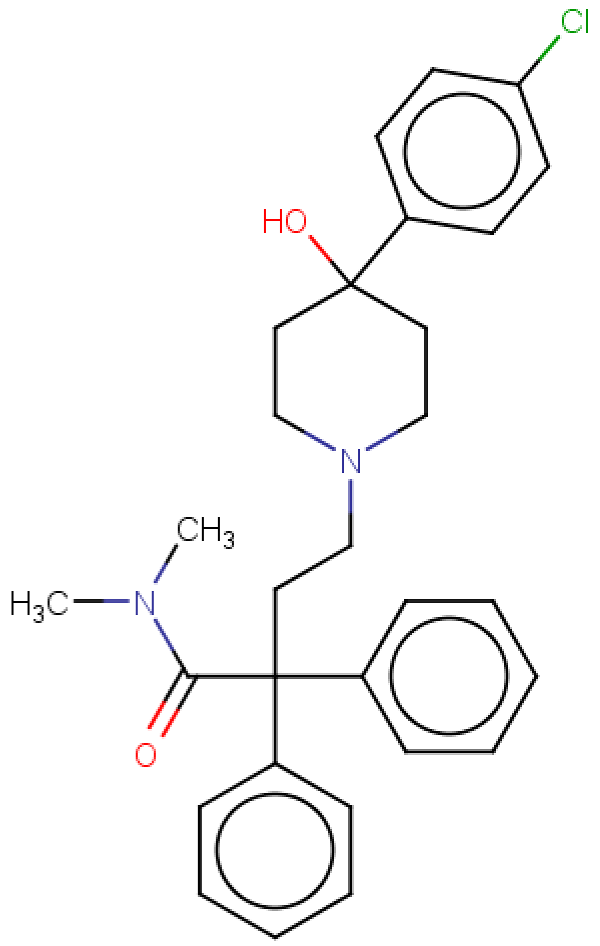

53179-11-6
Name: Loperamide
pIC50: 6.2
Rank: 38
Classes: Drug

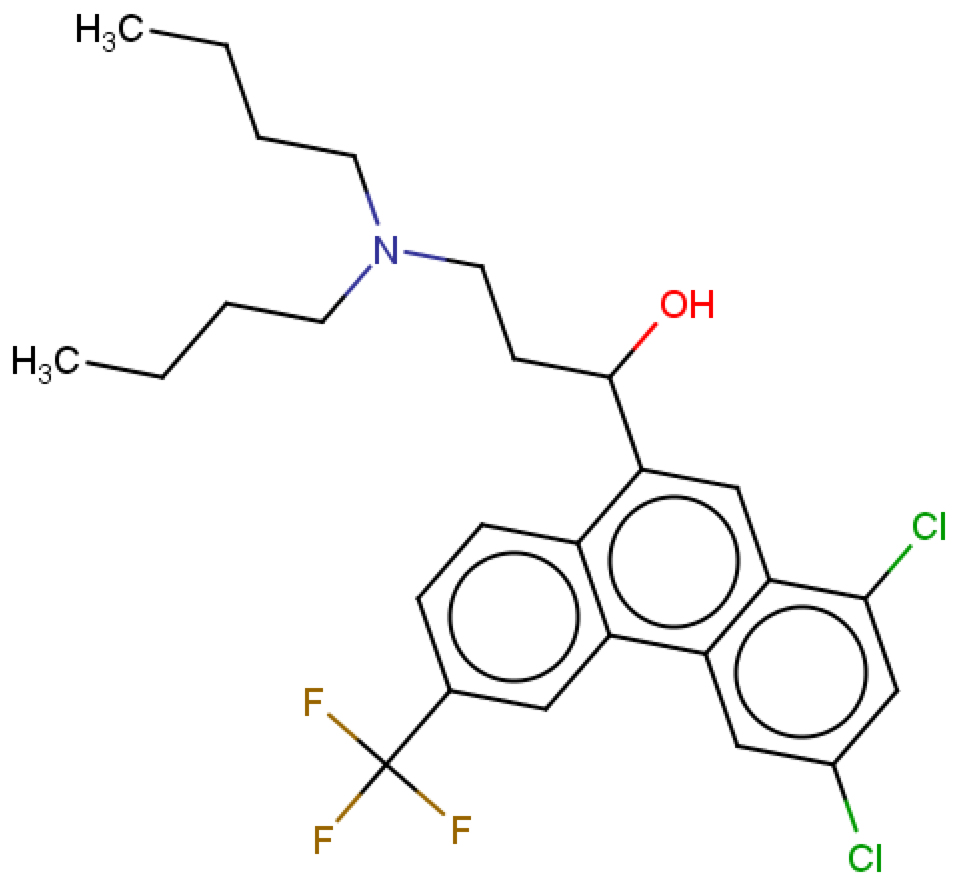

36167-63-2
Name: Halofantrine hydrochloride
pIC50: 6.2
Rank: 39
Classes: No defined

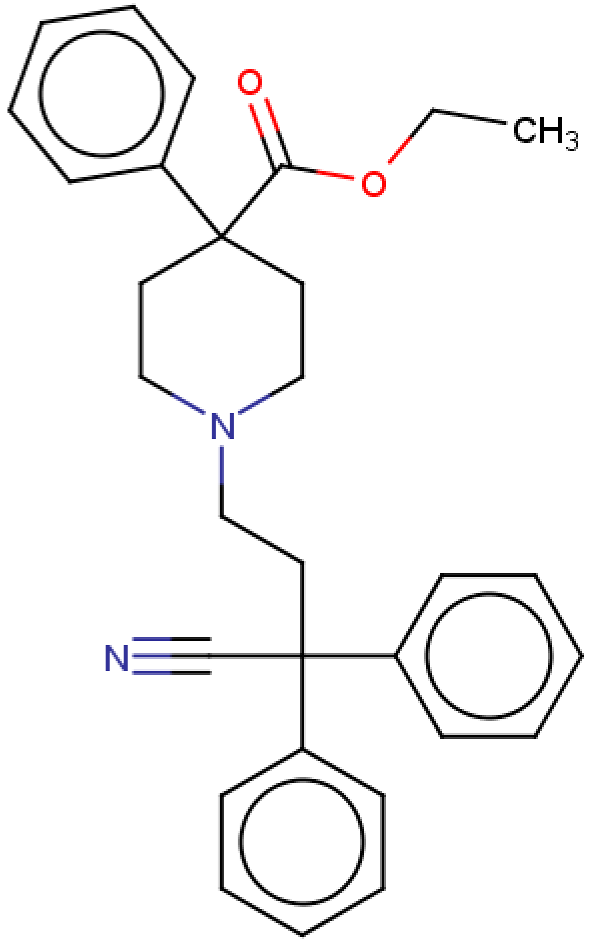

3810-80-8
Name: Diphenoxylate hydrochloride
pIC50: 6.2
Rank: 40
Classes: No defined

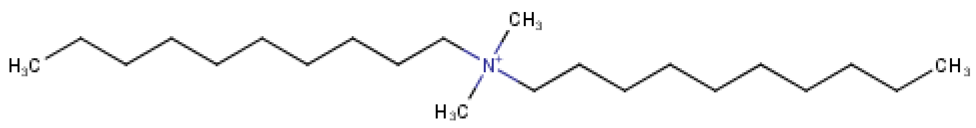

7173-51-5
Name: Didecyldimethylammonium chloride
pIC50: 6.19
Rank: 41
Classes: surfactant--NA--Pesticide--TSCA

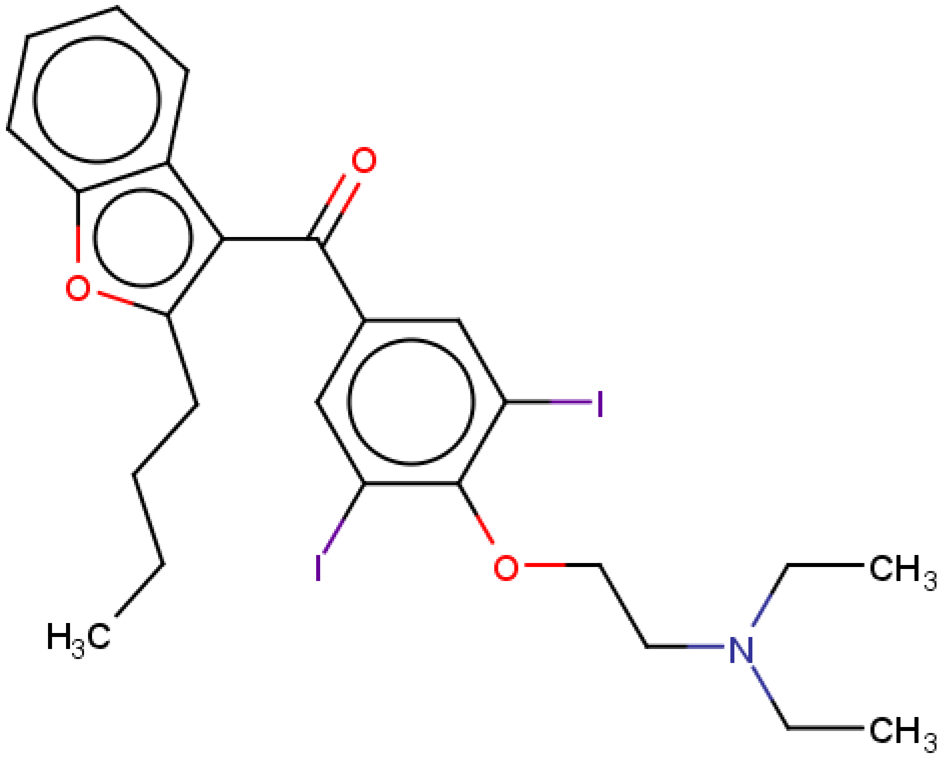

19774-82-4
Name: Amiodarone hydrochloride
pIC50: 6.19
Rank: 42
Classes: No defined

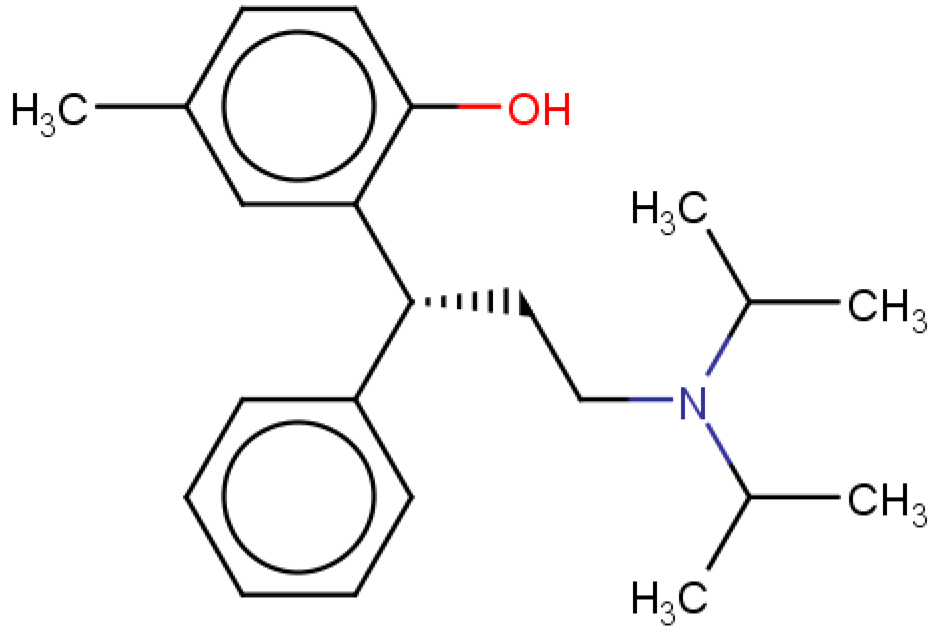

124937-51-5
Name: Tolterodine
pIC50: 6.14
Rank: 43
Classes: Drug

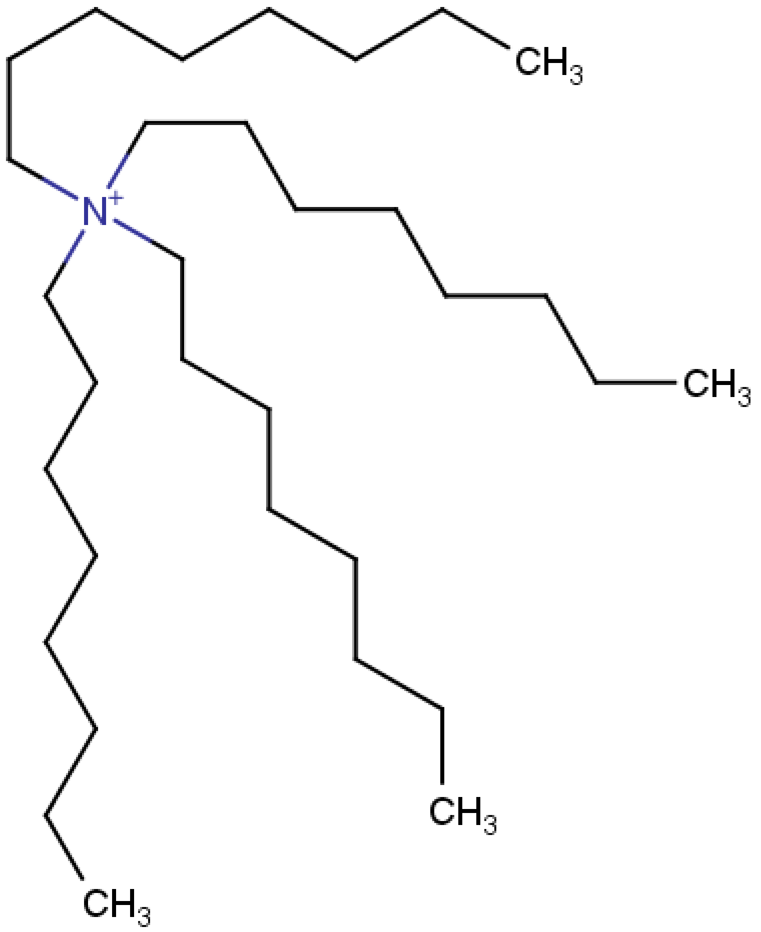

14866-33-2
Name: Tetra-N-octylammonium bromide
pIC50: 6.13
Rank: 44
Classes: No defined

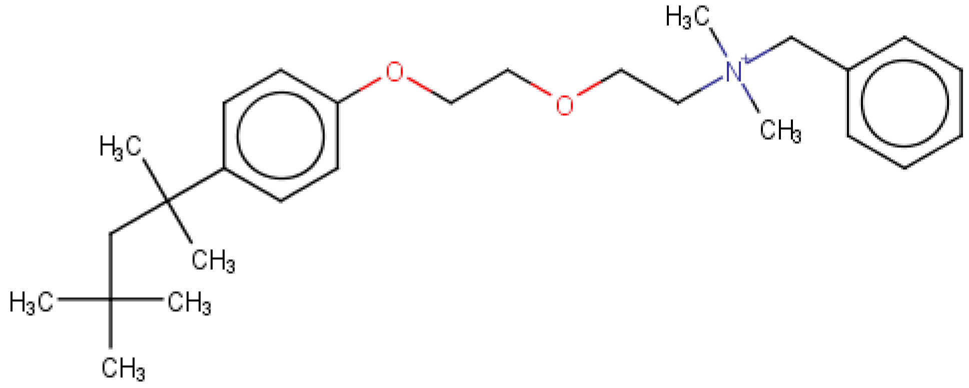

121-54-0
Name: Benzethonium chloride
pIC50: 6.11
Rank: 45
Classes: antimicrobial--NA--TSCA

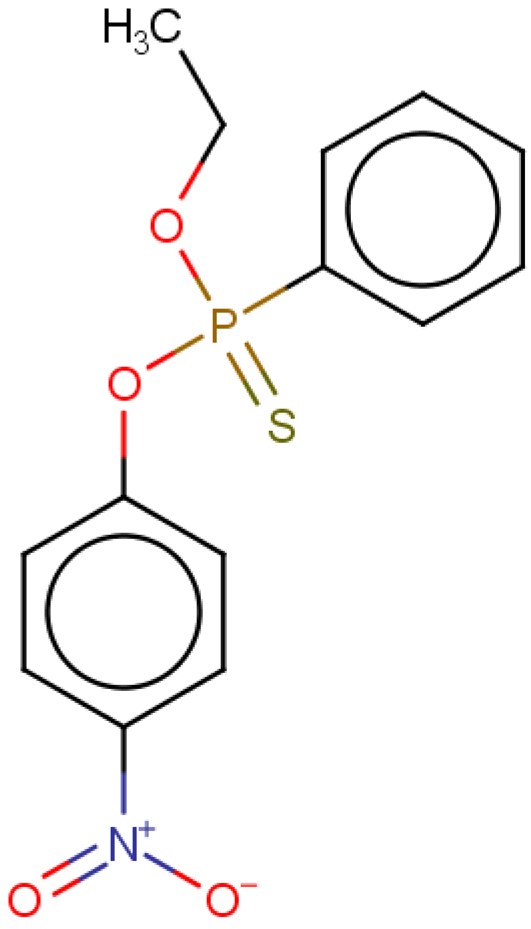

2104-64-5
Name: EPN
pIC50: 6.11
Rank: 46
Classes: No defined

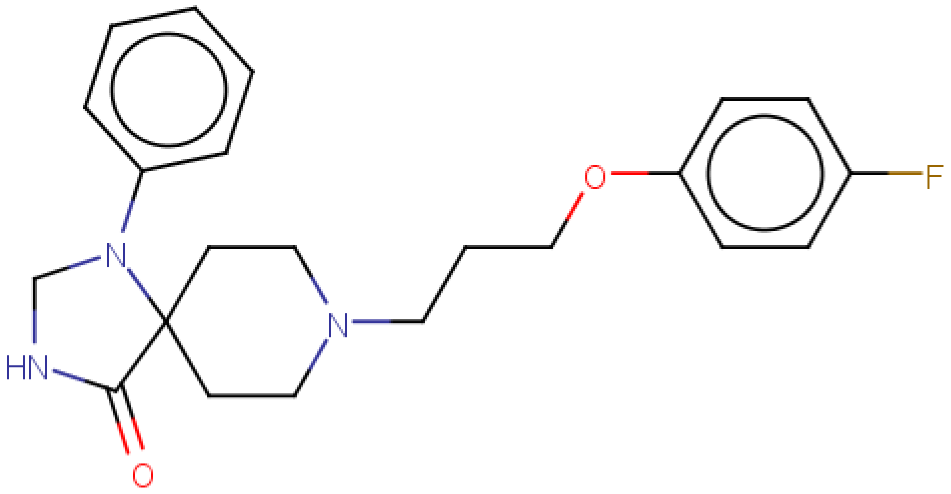

510-74-7
Name: AMI-193
pIC50: 6.1
Rank: 47
Classes: No defined

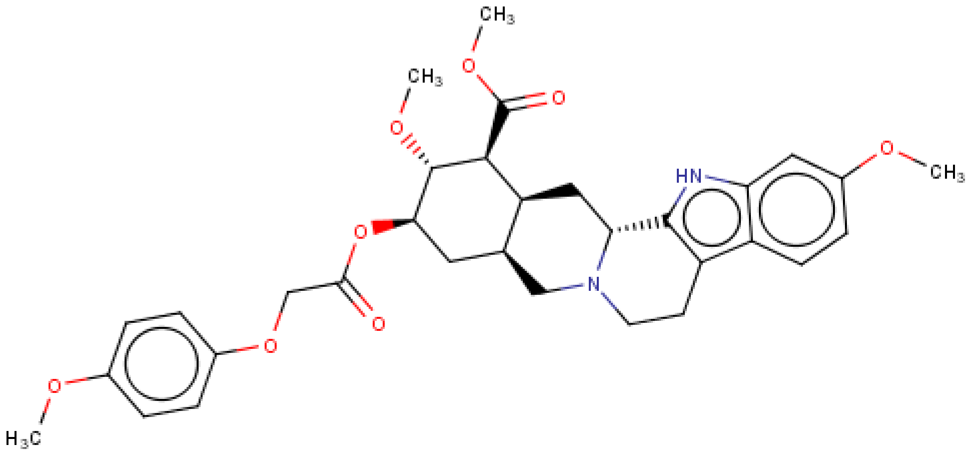

3735-85-1
Name: Mefeserpine
pIC50: 6.1
Rank: 48
Classes: No defined

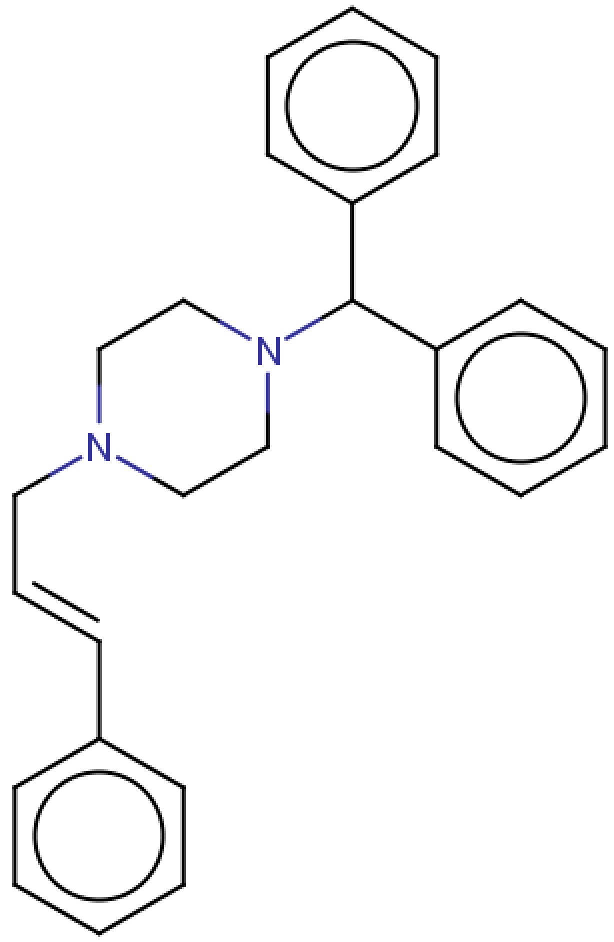

298-57-7
Name: Cinnarizine
pIC50: 6.09
Rank: 49
Classes: No defined

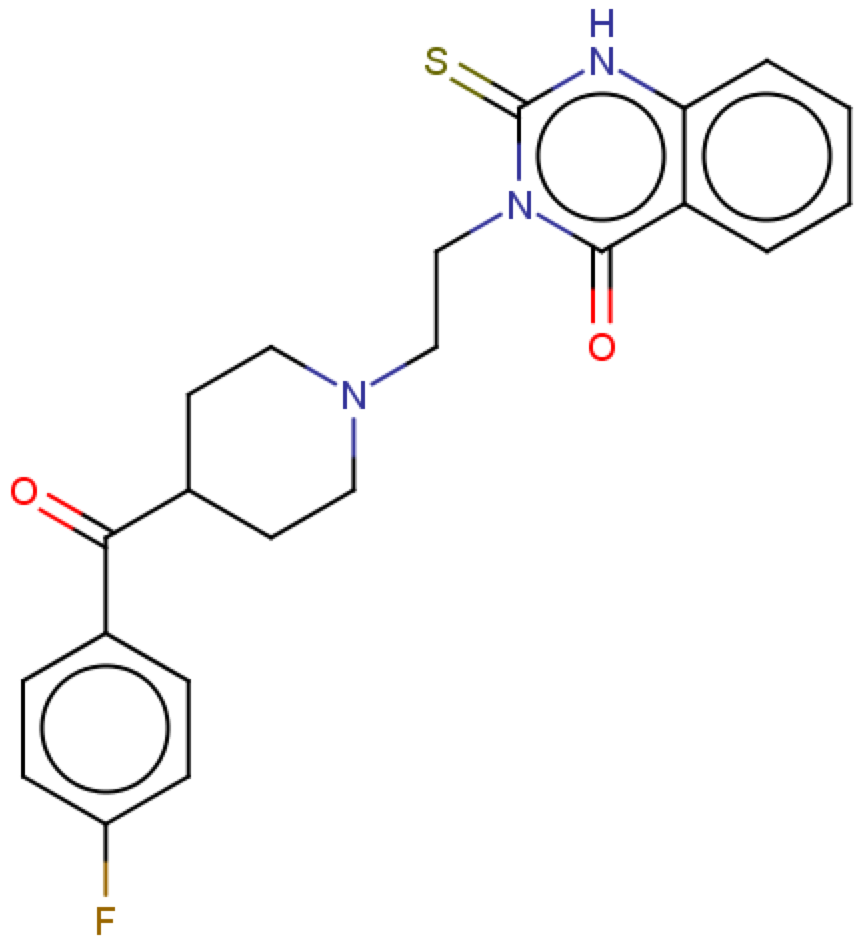

1135280-78-2
Name: Altanserin hydrochloride
pIC50: 6.09
Rank: 50
Classes: No defined

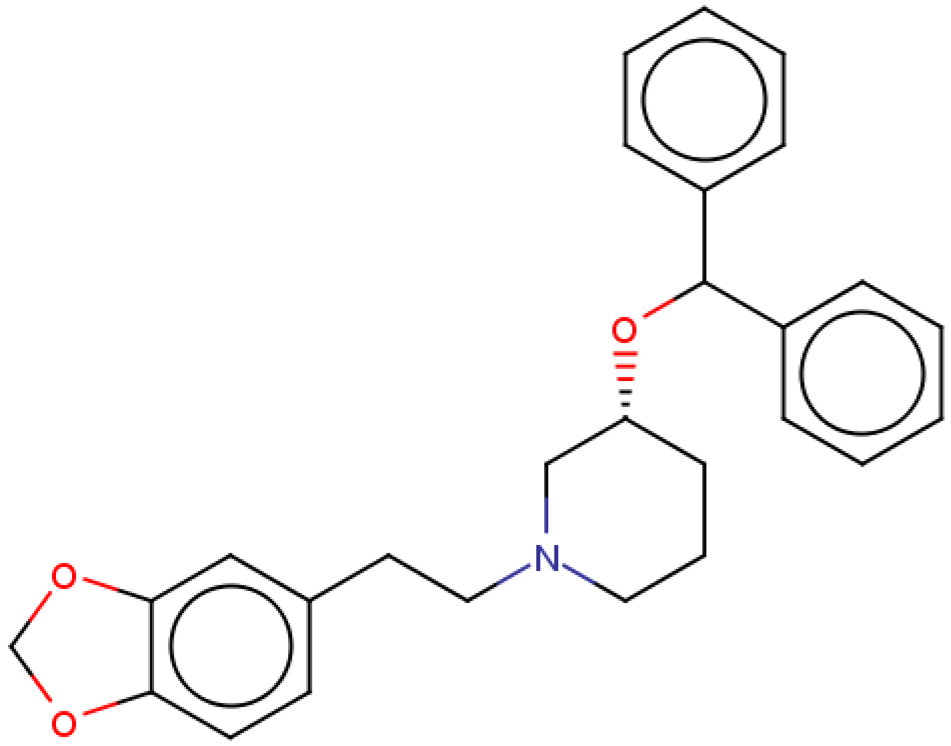

127308-82-1
Name: Zamifenacin
pIC50: 6.08
Rank: 51
Classes: No defined

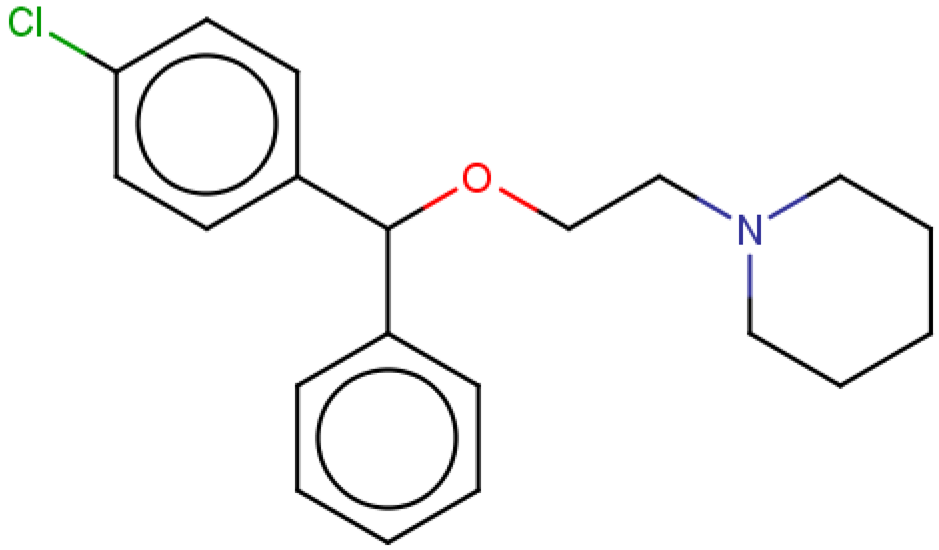

14984-68-0
Name: Cloperastine hydrochloride
pIC50: 6.07
Rank: 52
Classes: No defined

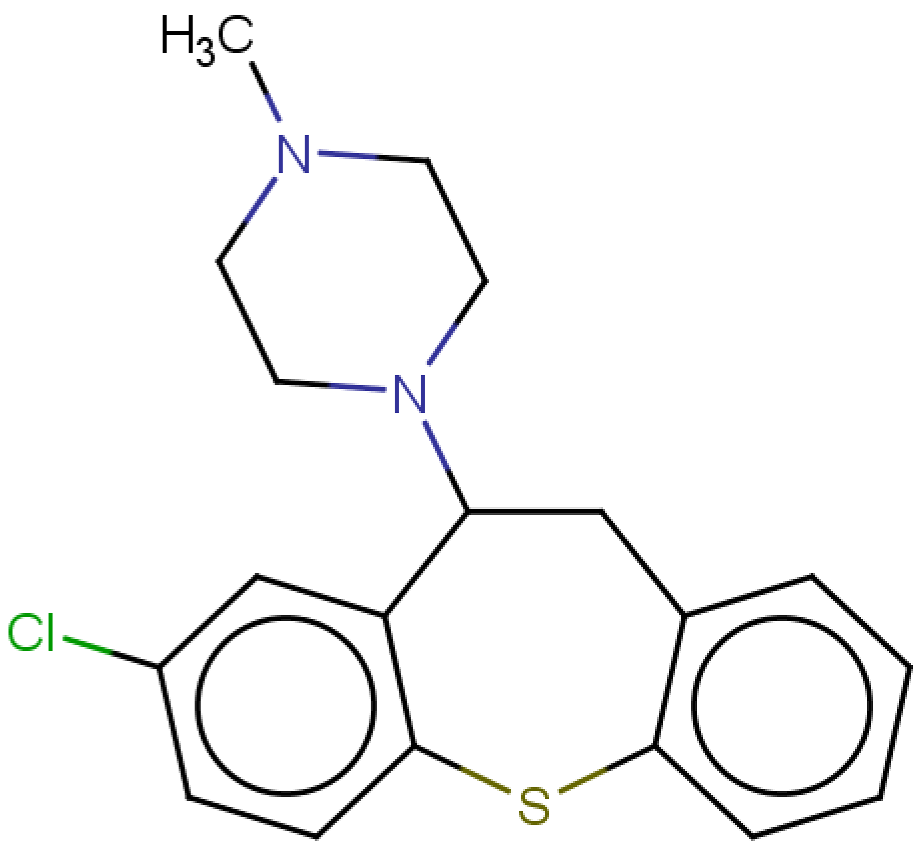

4789-68-8
Name: Octoclothepine maleate
pIC50: 6.07
Rank: 53
Classes: No defined

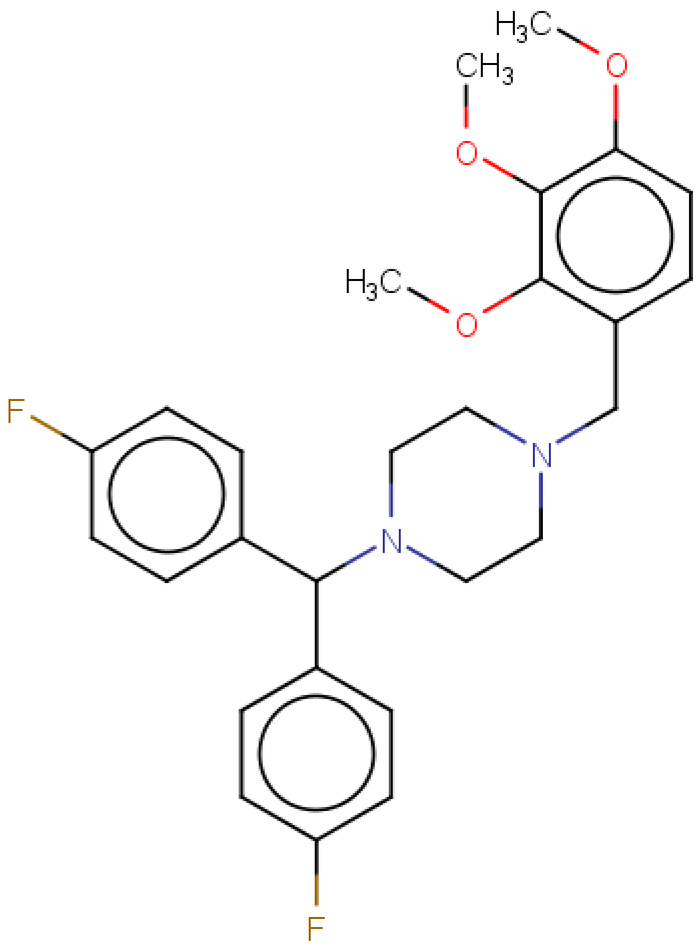

101477-54-7
Name: Lomerizine dihydrochloride
pIC50: 6.07
Rank: 54
Classes: No defined

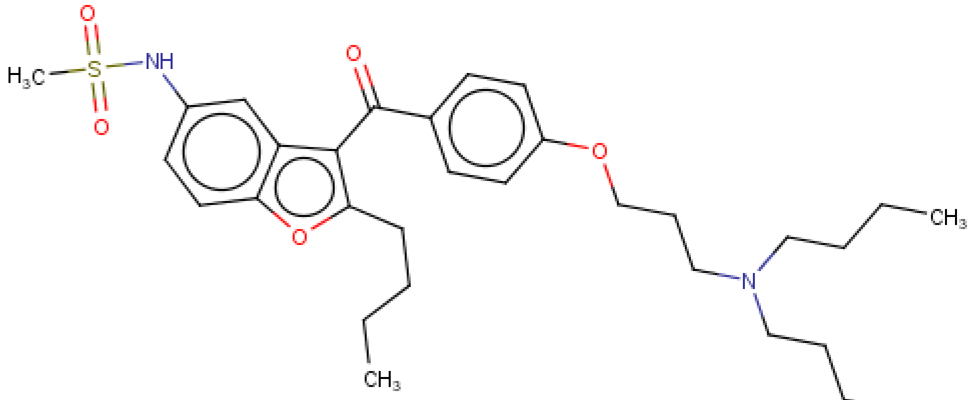

141626-36-0
Name: Dronedarone
pIC50: 6.07
Rank: 55
Classes: Drug

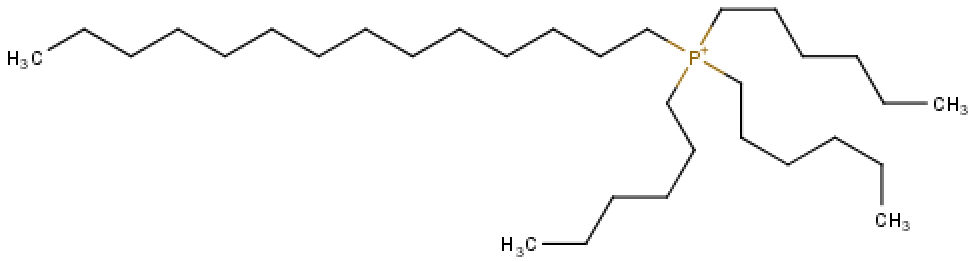

258864-54-9
Name: Trihexyltetradecylphosphonium chloride
pIC50: 6.06
Rank: 56
Classes: No defined

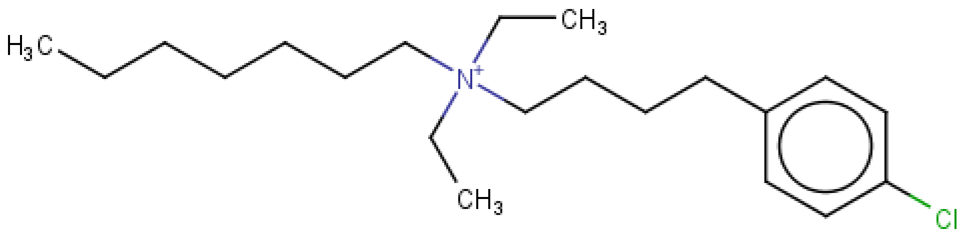

92953-10-1
Name: Clofilium tosylate
pIC50: 6.05
Rank: 57
Classes: No defined

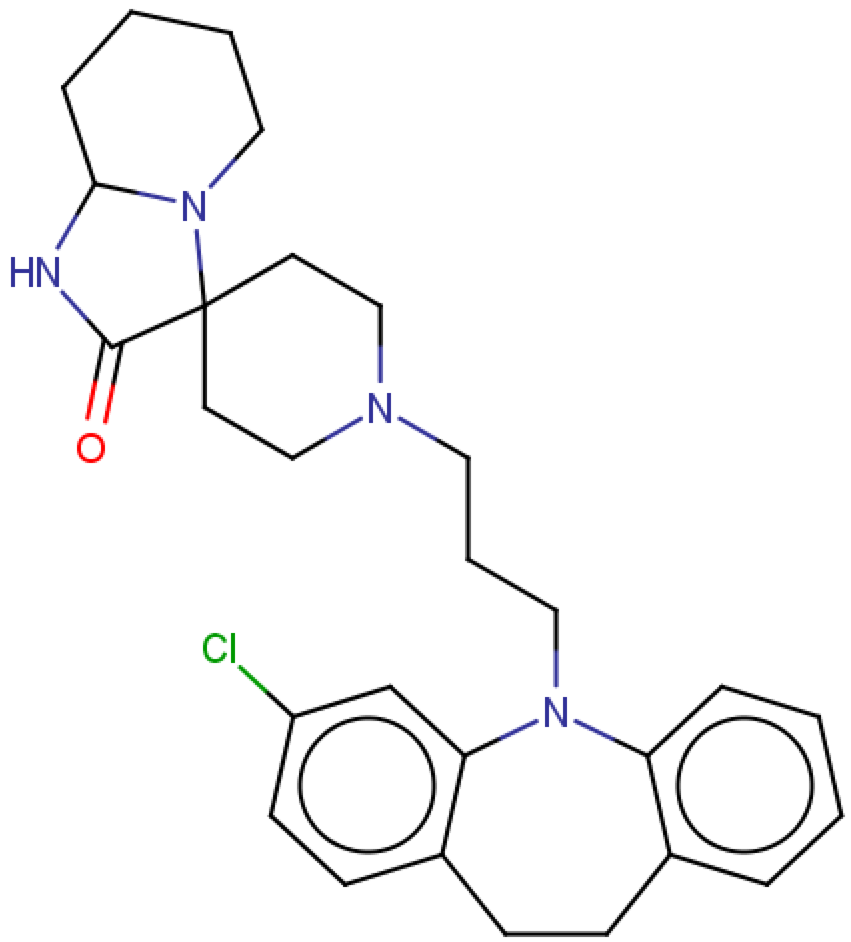

89419-40-9
Name: Mosapramine
pIC50: 6.05
Rank: 58
Classes: No defined

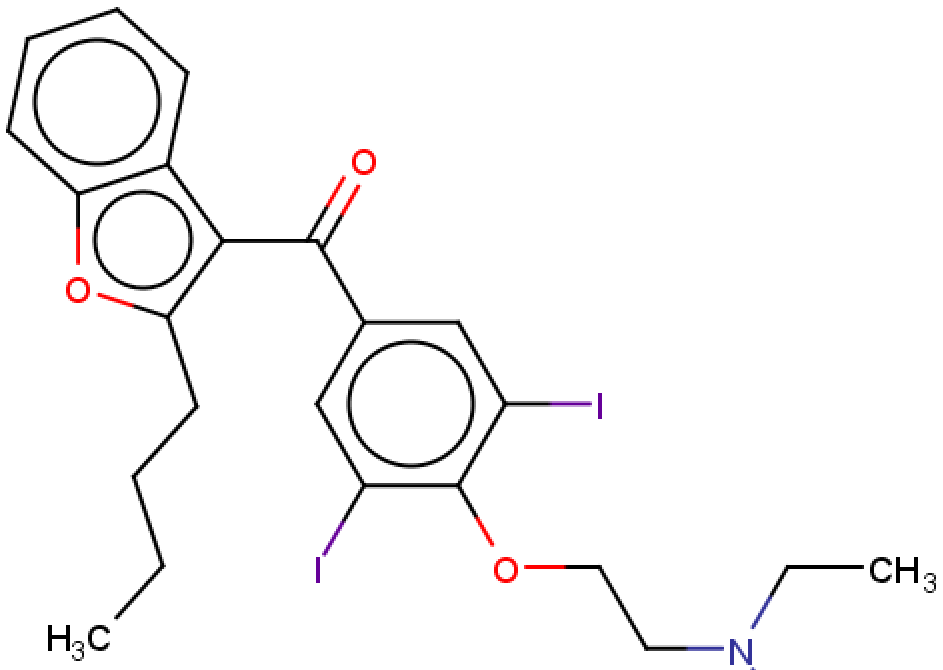

1951-25-3
Name: Amiodarone
pIC50: 6.04
Rank: 59
Classes: Drug

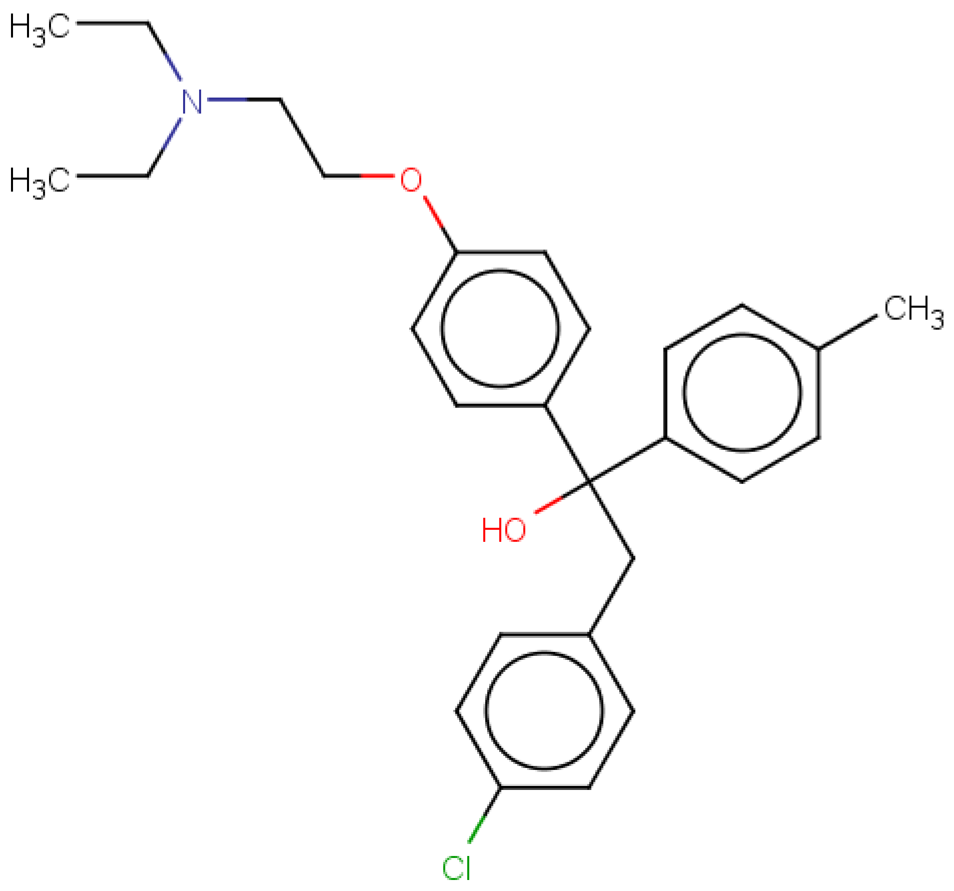

78-41-1
Name: Triparanol
pIC50: 5.95
Rank: 60
Classes: No defined

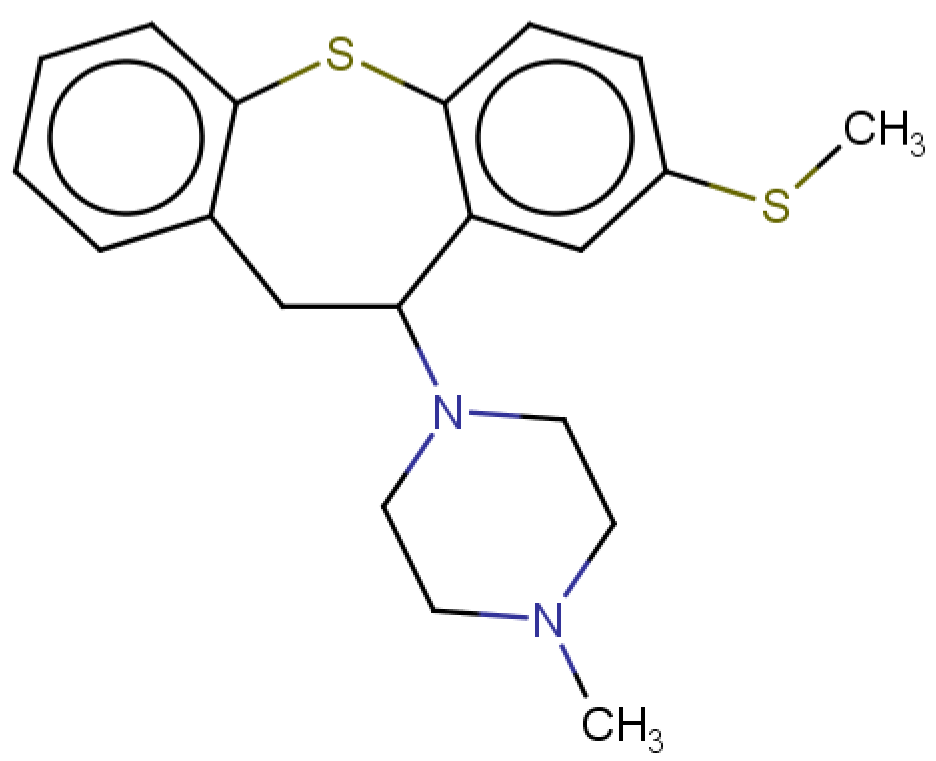

20229-30-5
Name: Metitepine
pIC50: 5.94
Rank: 61
Classes: No defined

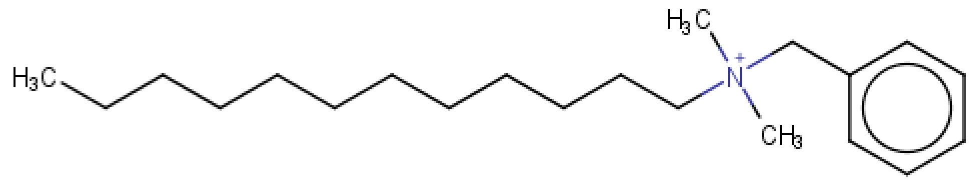

139-07-1
Name: Benzyldimethyldodecylammonium chlo[illegible]
pIC50: 5.92
Rank: 62
Classes: hair conditioner--TSCA

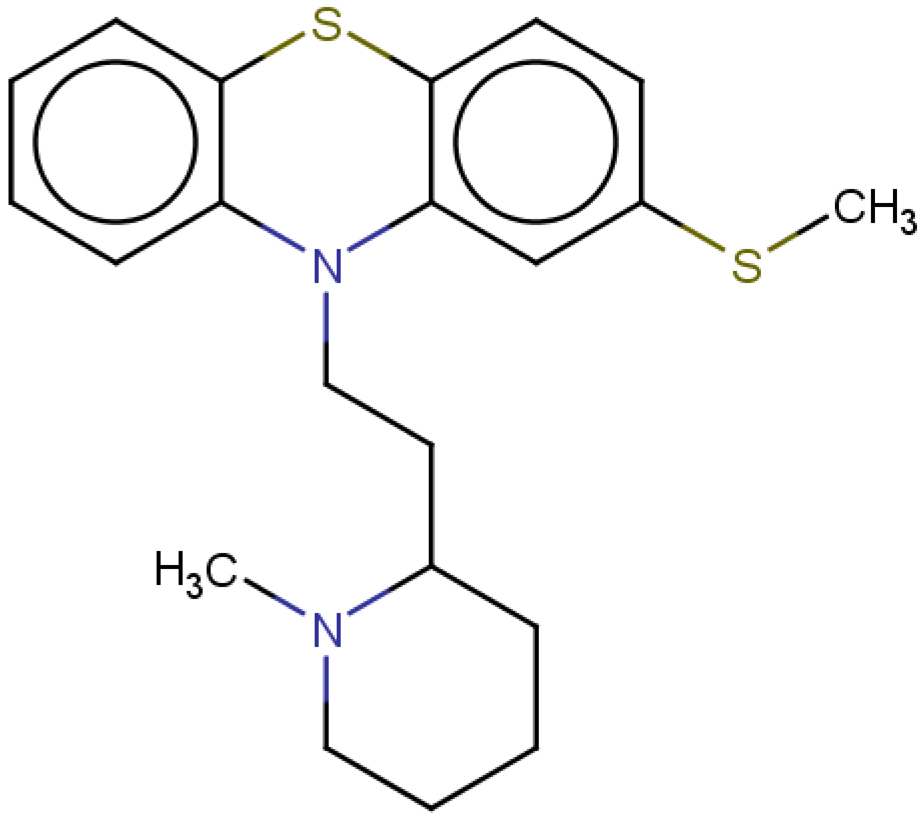

130-61-0
Name: Thioridazine hydrochloride
pIC50: 5.91
Rank: 63
Classes: No defined

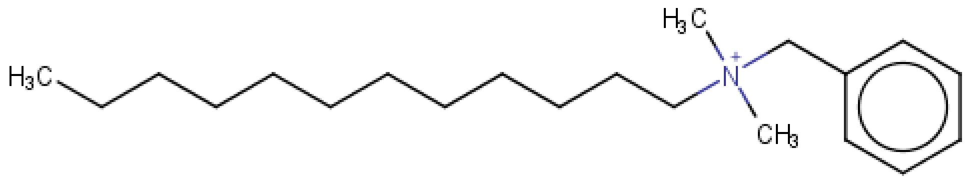

7281-04-1
Name: Benzyldodecyldimethylammonium bro[illegible]
pIC50: 5.9
Rank: 64
Classes: hair conditioner

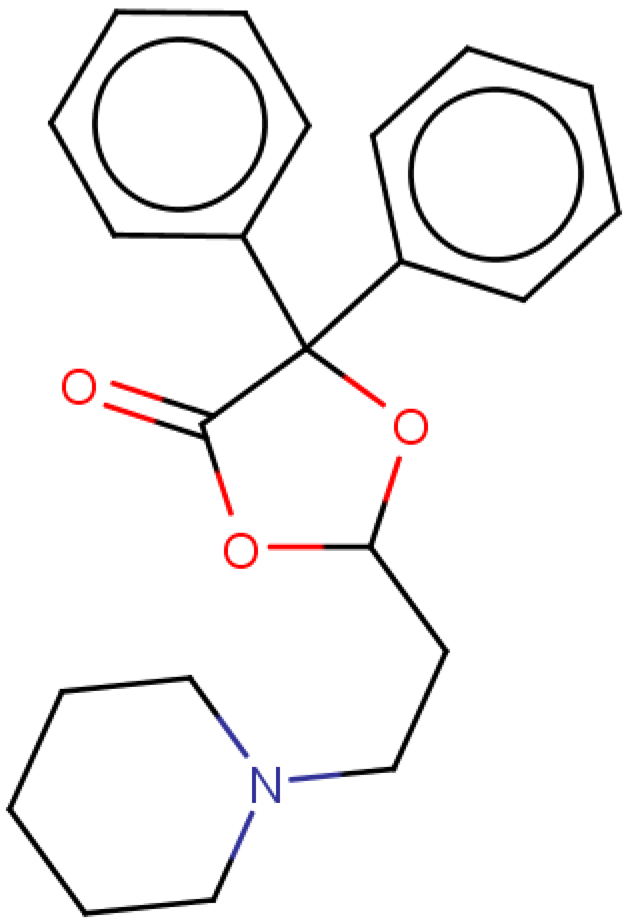

18174-58-8
Name: Pipoxolan hydrochloride
pIC50: 5.9
Rank: 65
Classes: No defined

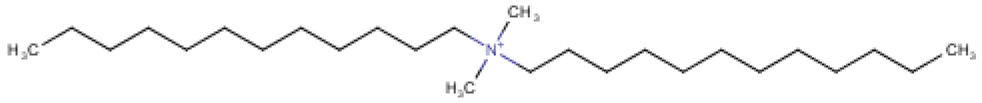

3282-73-3
Name: Didodecyldimethylammonium bromide
pIC50: 5.89
Rank: 66
Classes: TSCA

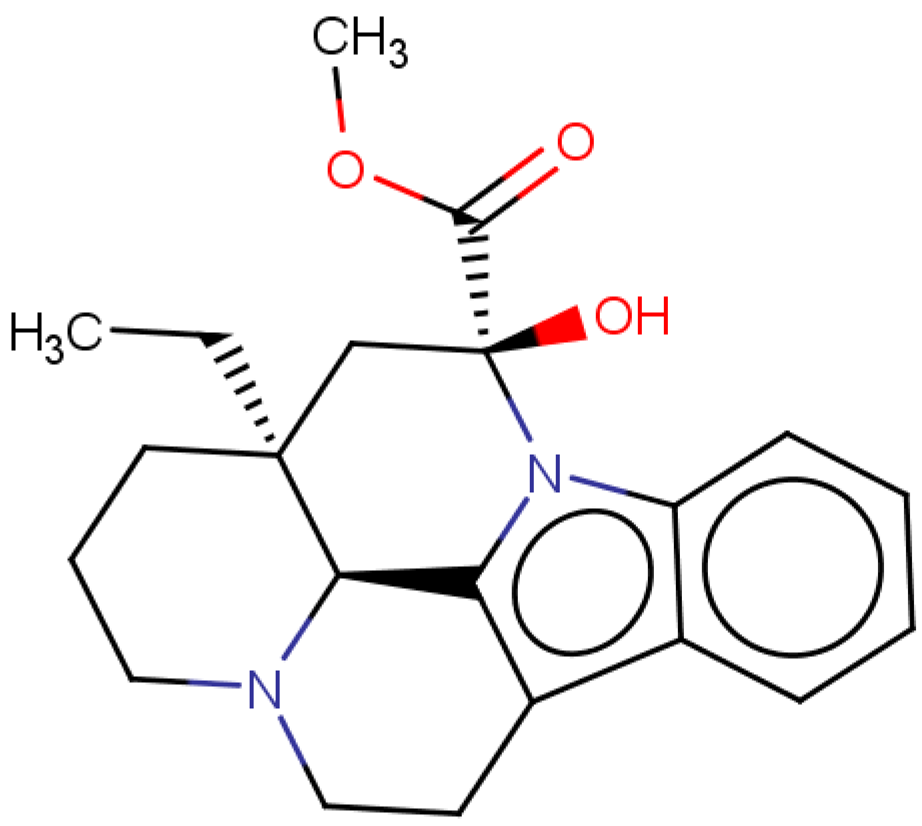

1617-90-9
Name: Vincamine
pIC50: 5.88
Rank: 67
Classes: No defined

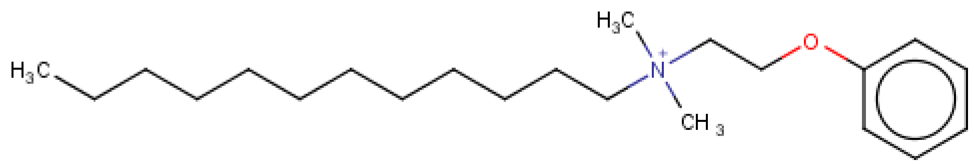

538-71-6
Name: Domiphen bromide
pIC50: 5.87
Rank: 68
Classes: antimicrobial--TSCA

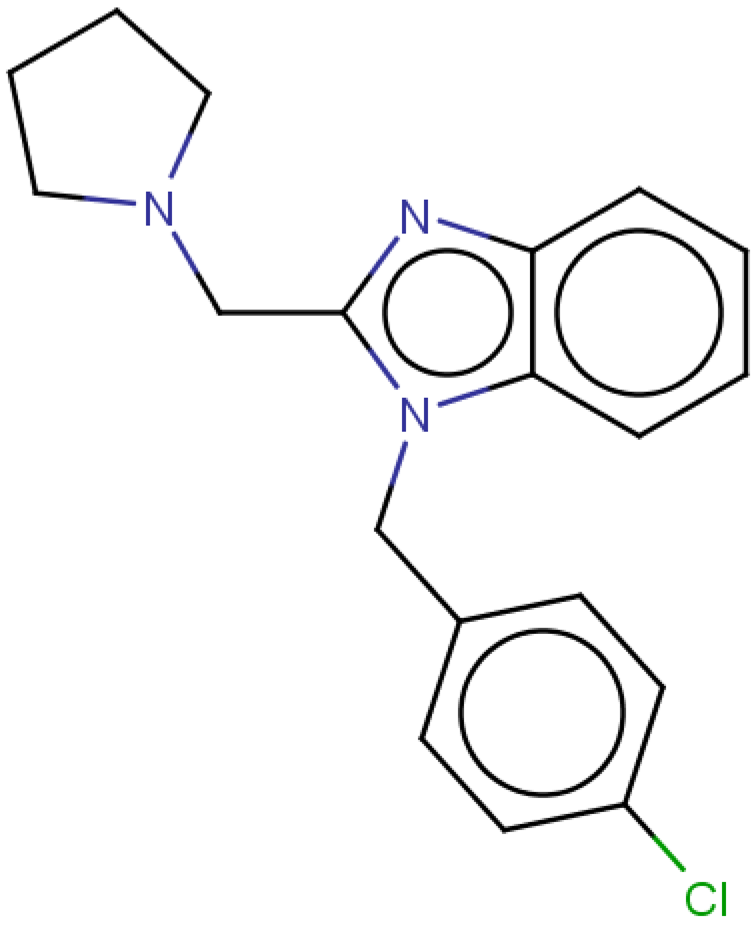

1163-36-6
Name: Clemizole hydrochloride
pIC50: 5.87
Rank: 69
Classes: No defined

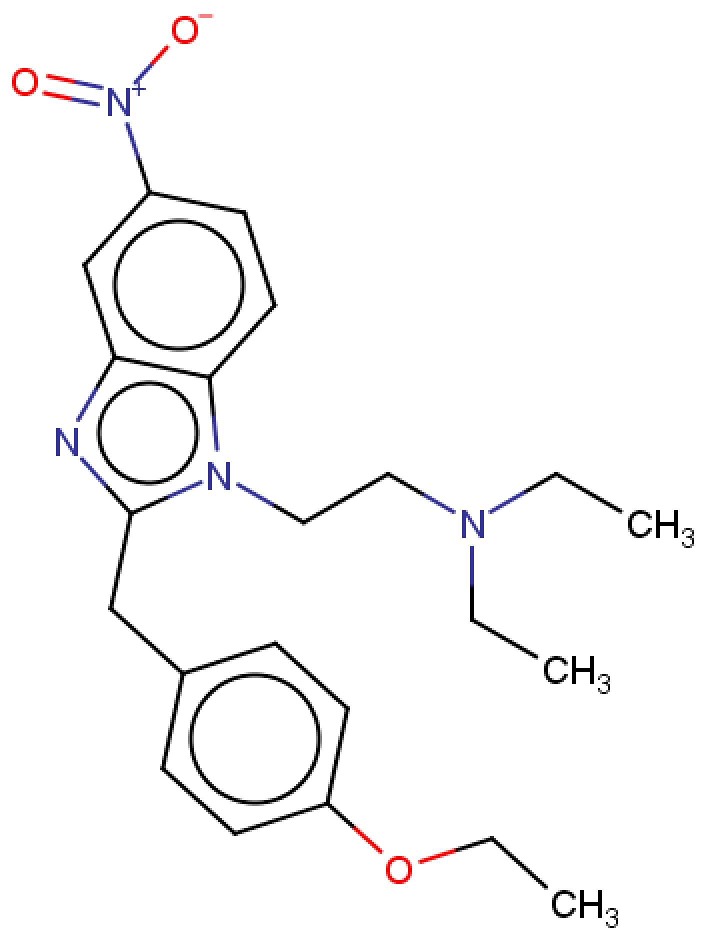

911-65-9
Name: Etonitazene
pIC50: 5.86
Rank: 70
Classes: Drug

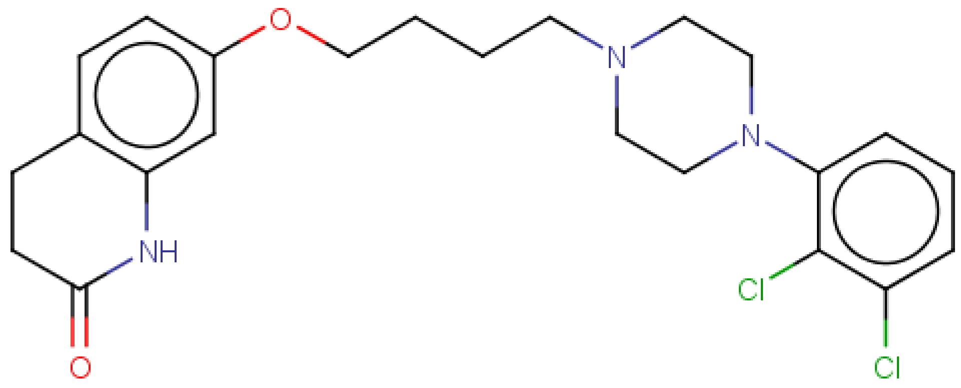

129722-12-9
Name: Aripiprazole
pIC50: 5.85
Rank: 71
Classes: Drug

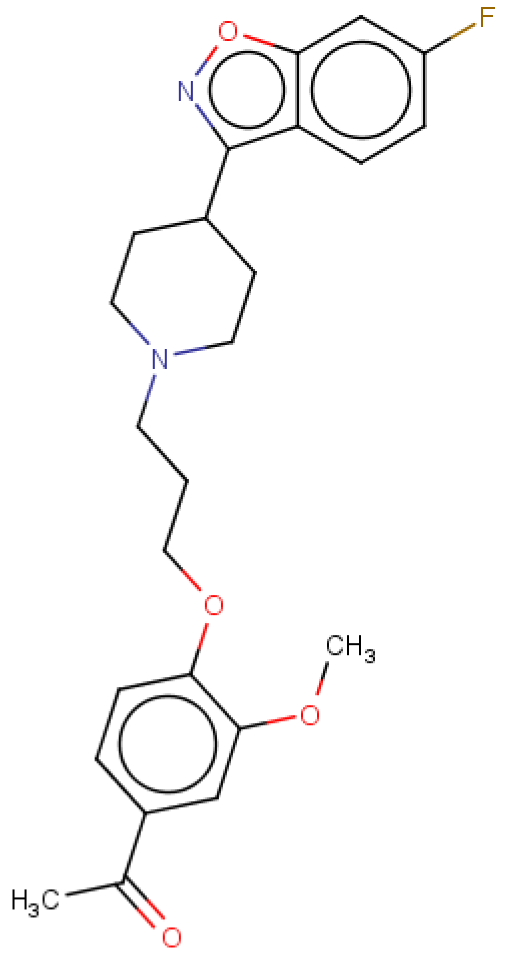

133454-47-4
Name: Iloperidone
pIC50: 5.85
Rank: 72
Classes: Drug

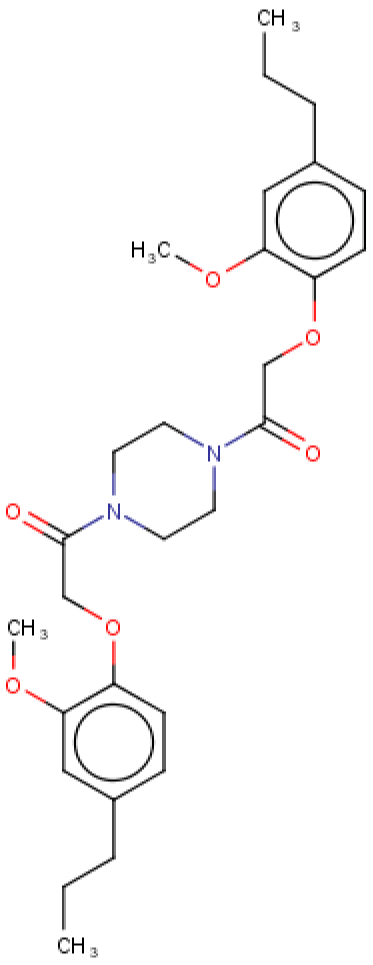

154-82-5
Name: Simetride
pIC50: 5.85
Rank: 73
Classes: No defined

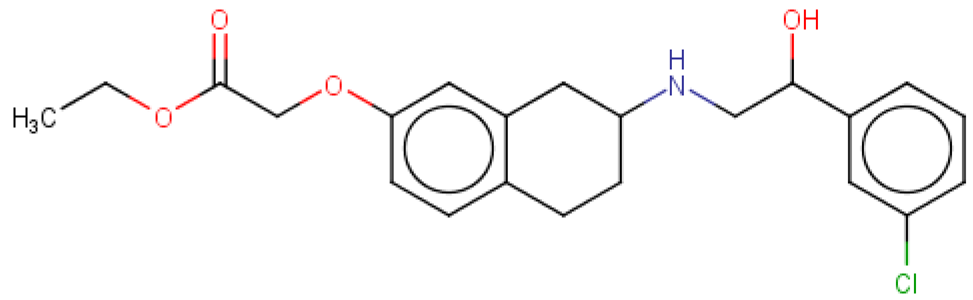

929601-09-2
Name: SR58611
pIC50: 5.84
Rank: 74
Classes: No defined

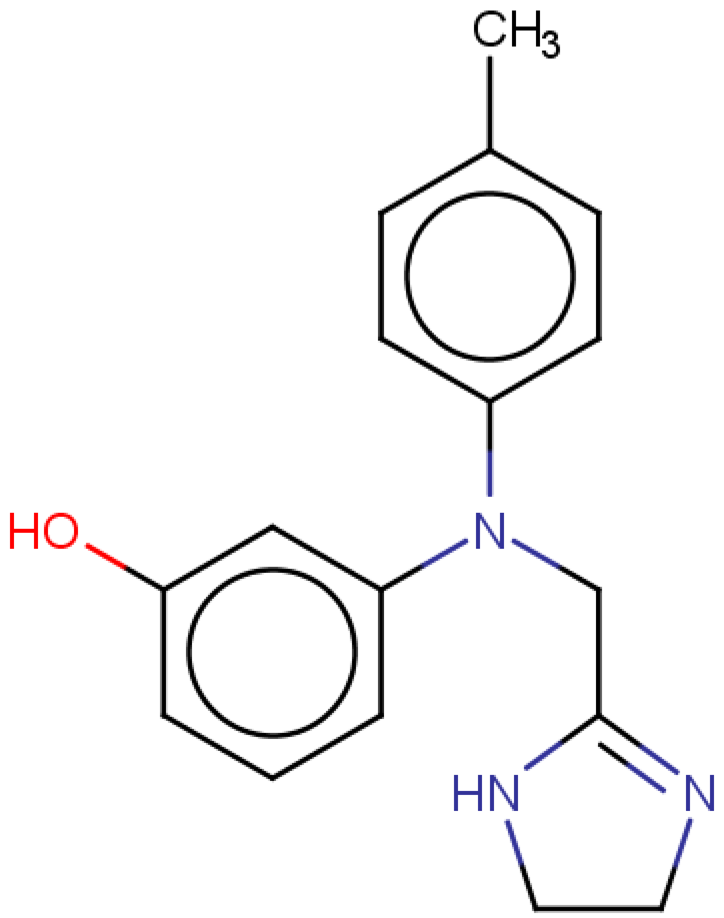

50-60-2
Name: Phentolamine
pIC50: 5.84
Rank: 75
Classes: Drug

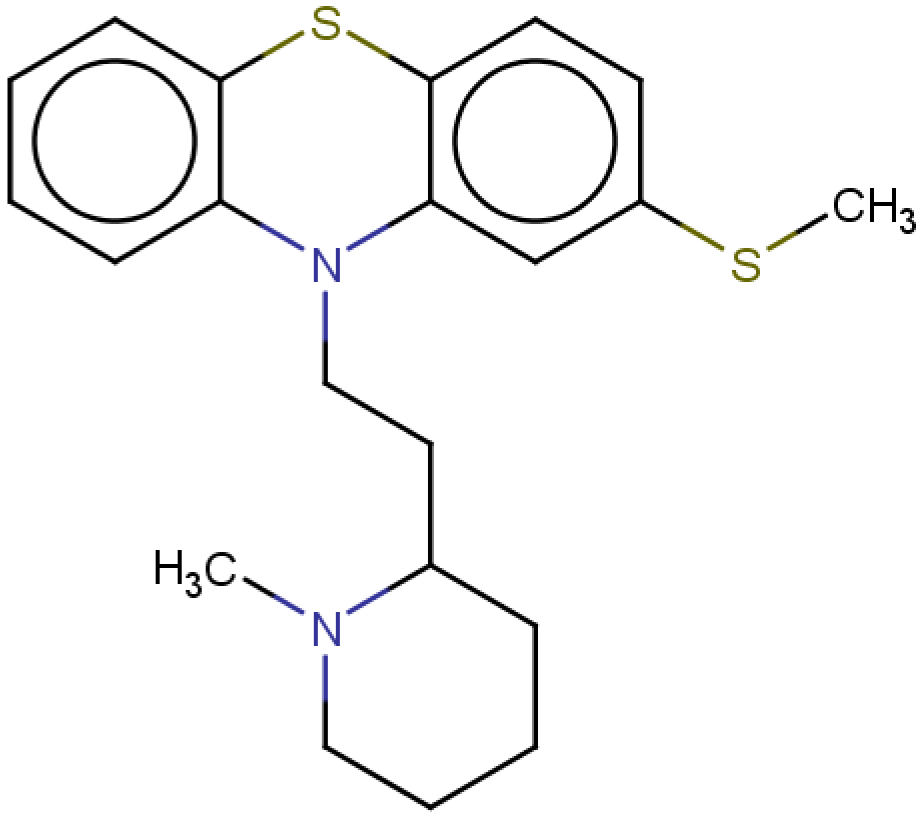

50-52-2
Name: Thioridazine
pIC50: 5.84
Rank: 76
Classes: Drug

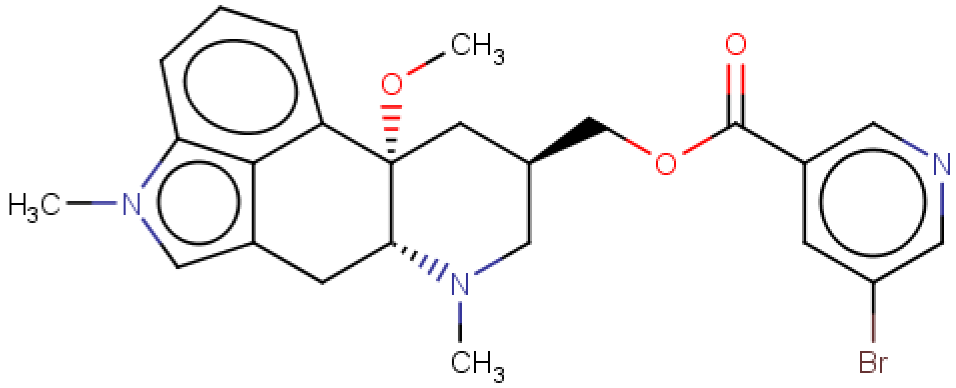

27848-84-6
Name: Nicergoline
pIC50: 5.84
Rank: 77
Classes: Drug

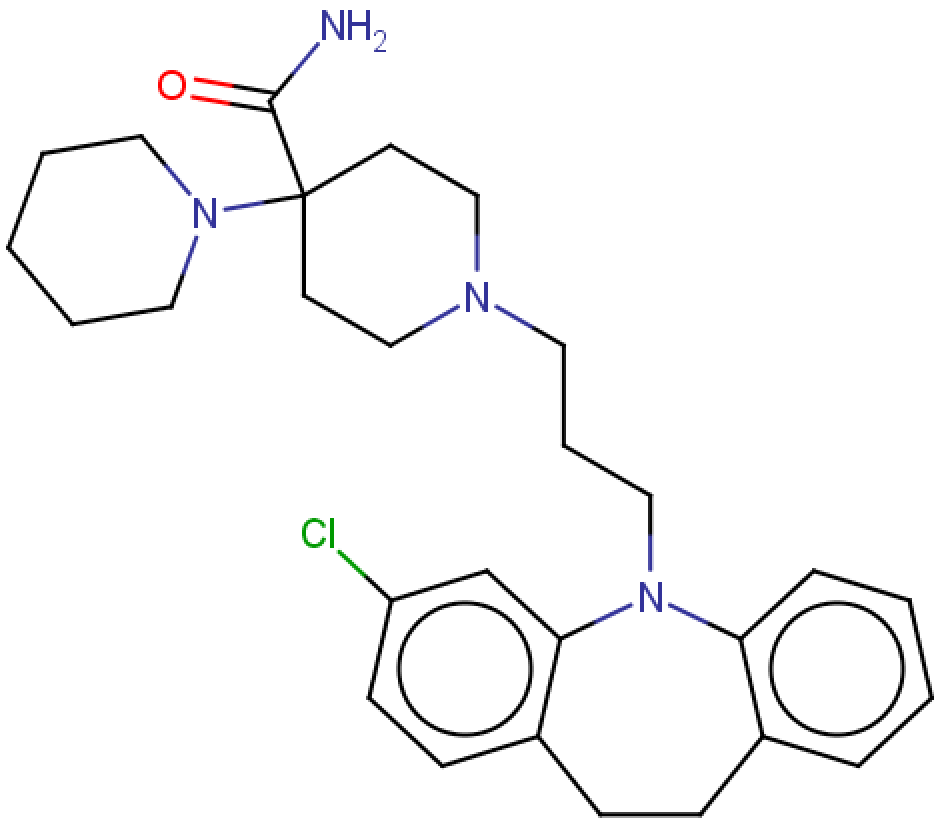

47739-98-0
Name: Clocapramine
pIC50: 5.84
Rank: 78
Classes: Drug

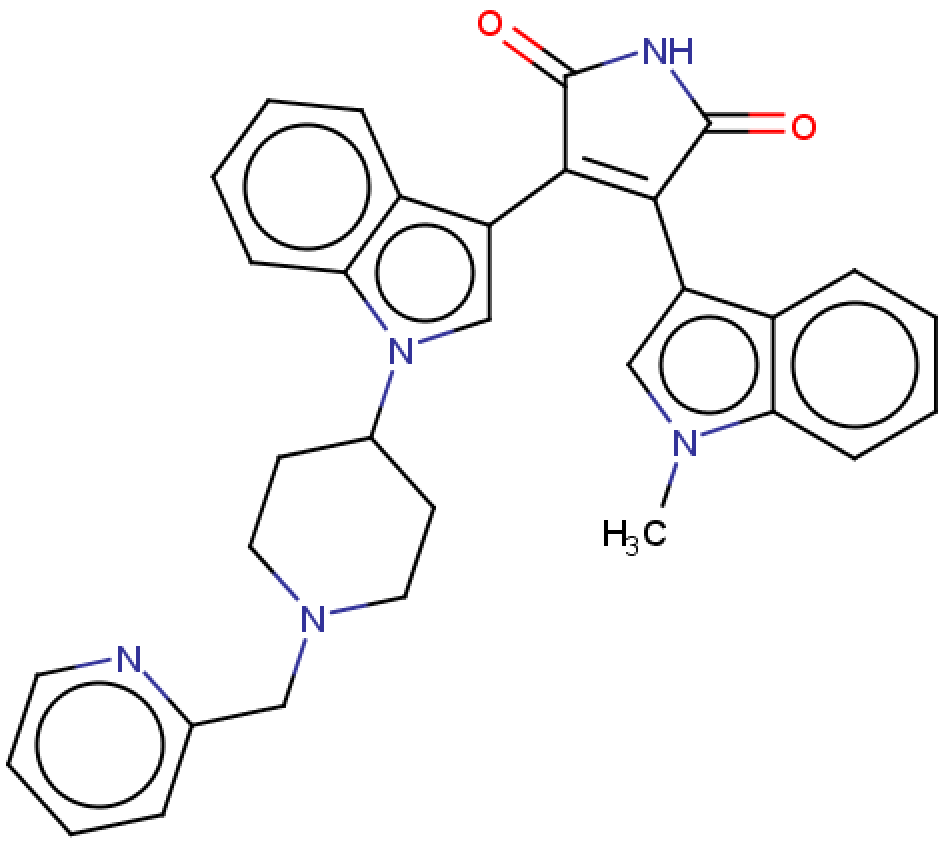

170364-57-5
Name: Enzastaurin
pIC50: 5.82
Rank: 79
Classes: No defined

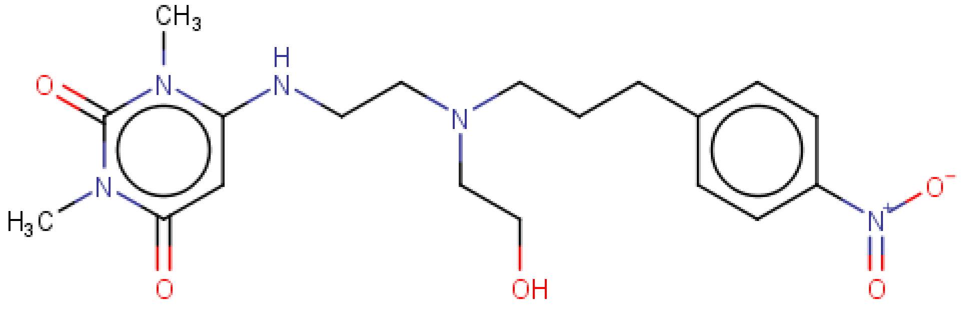

130636-43-0
Name: Nifekalant hydrochloride
pIC50: 5.82
Rank: 80
Classes: No defined

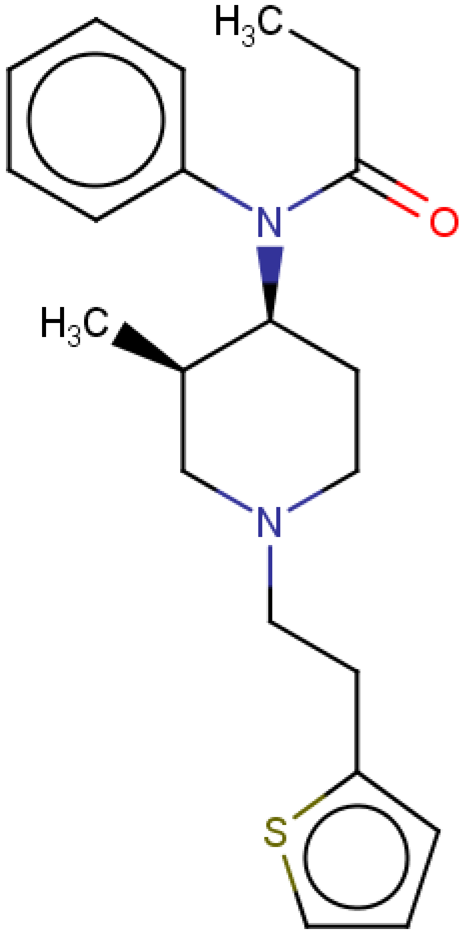

131690-45-4
Name: cis-N-(1-(2-(2-Thienyl)ethyl)-3-methyl-4-

pIC50: 5.81
Rank: 81
Classes: No defined

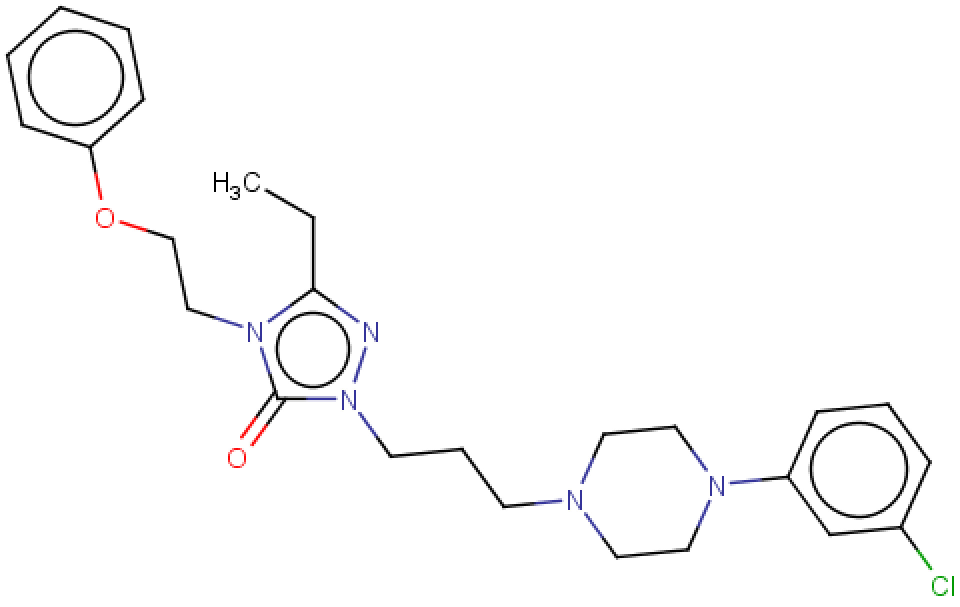

82752-99-6
Name: Nefazodone hydrochloride
pIC50: 5.8
Rank: 82
Classes: No defined

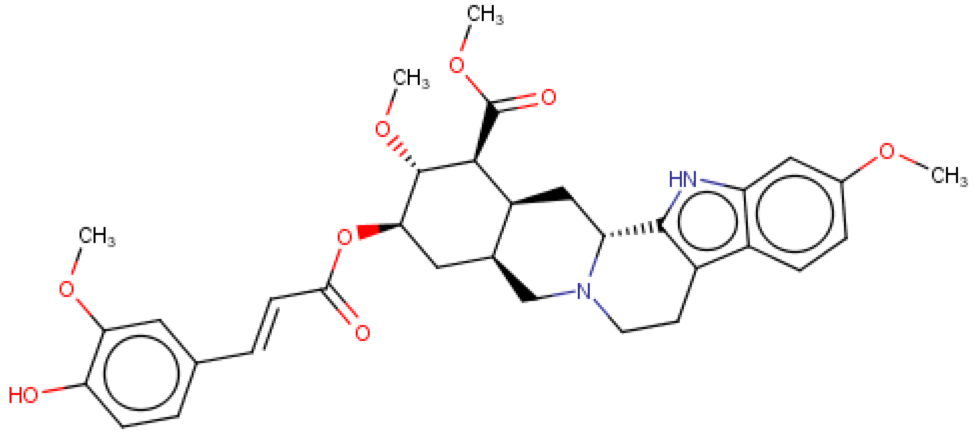

73573-42-9
Name: Rescimetol
pIC50: 5.8
Rank: 83
Classes: No defined

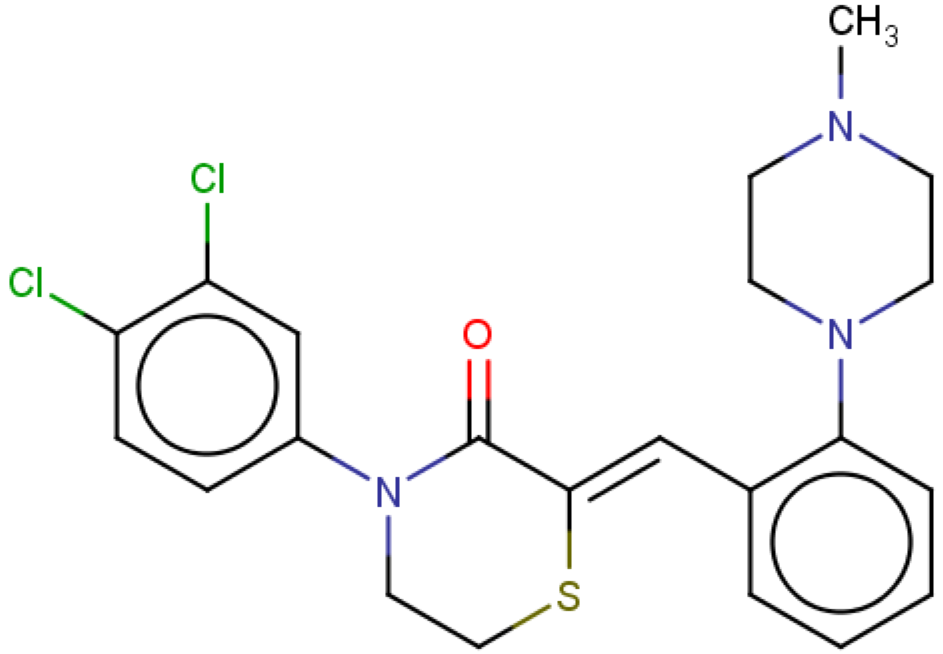

361343-19-3
Name: Elzasonan
pIC50: 5.79
Rank: 84
Classes: No defined

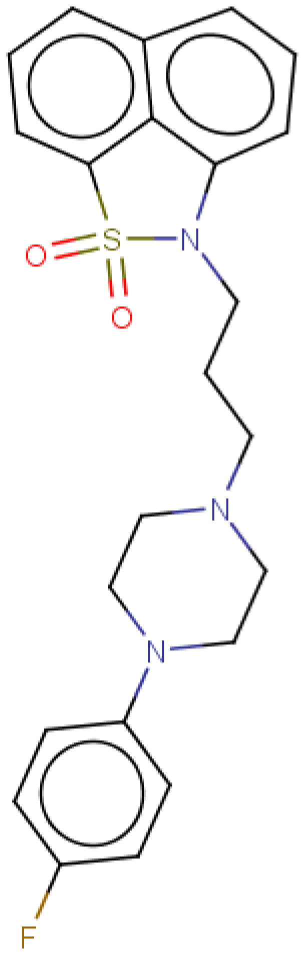

127625-29-0
Name: Fananserin
pIC50: 5.79
Rank: 85
Classes: No defined

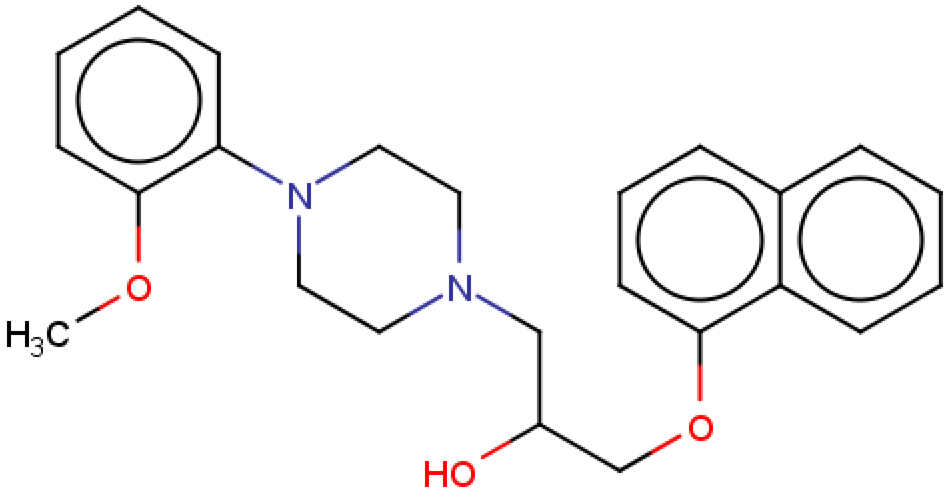

57149-07-2
Name: Naftopidil
pIC50: 5.77
Rank: 86
Classes: Drug

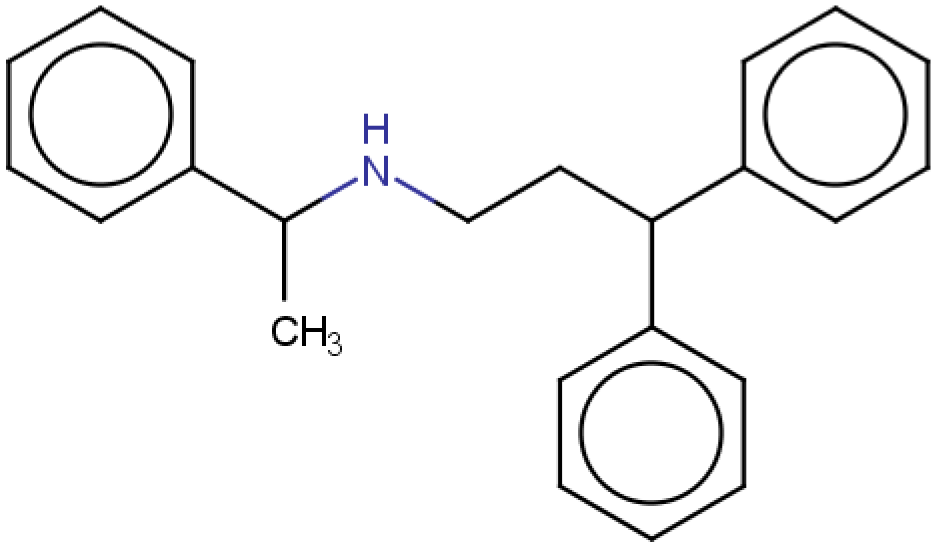

13636-18-5
Name: Fendiline hydrochloride
pIC50: 5.77
Rank: 87
Classes: No defined

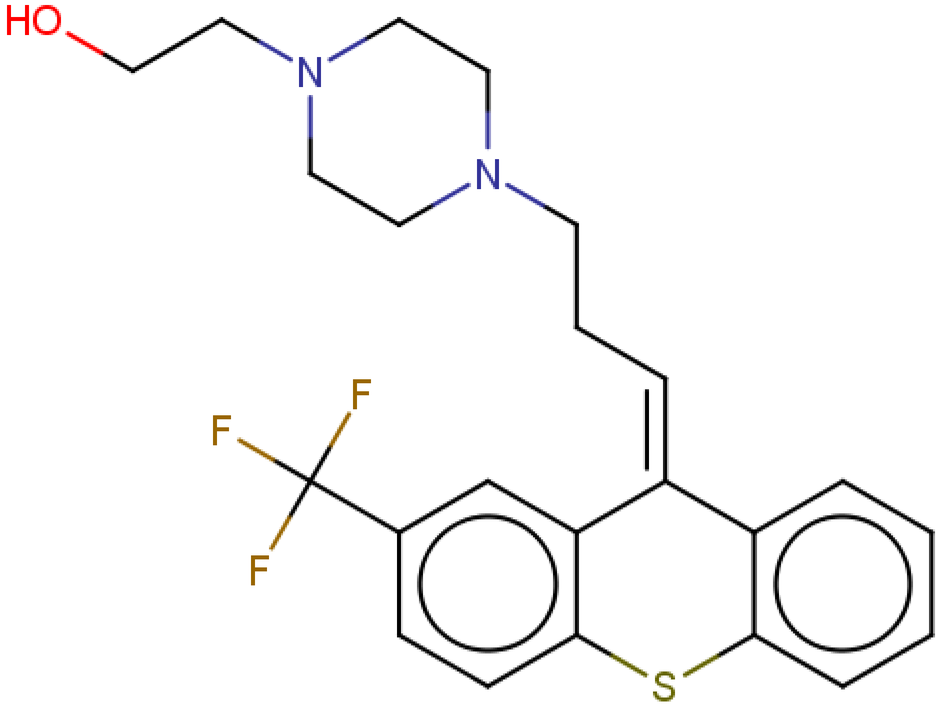

53772-82-0
Name: cis-Flupentixol
pIC50: 5.77
Rank: 88
Classes: Drug

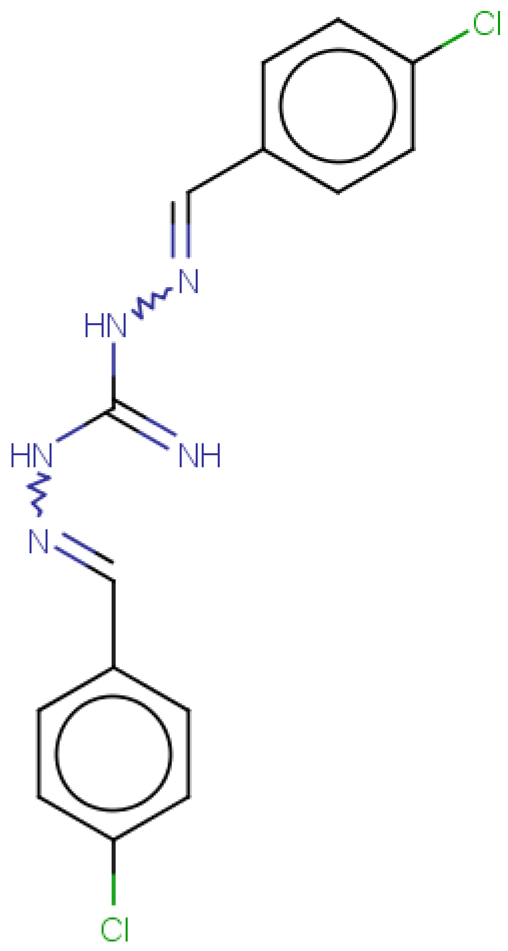

25875-51-8
Name: Robenidine
pIC50: 5.77
Rank: 89
Classes: No defined

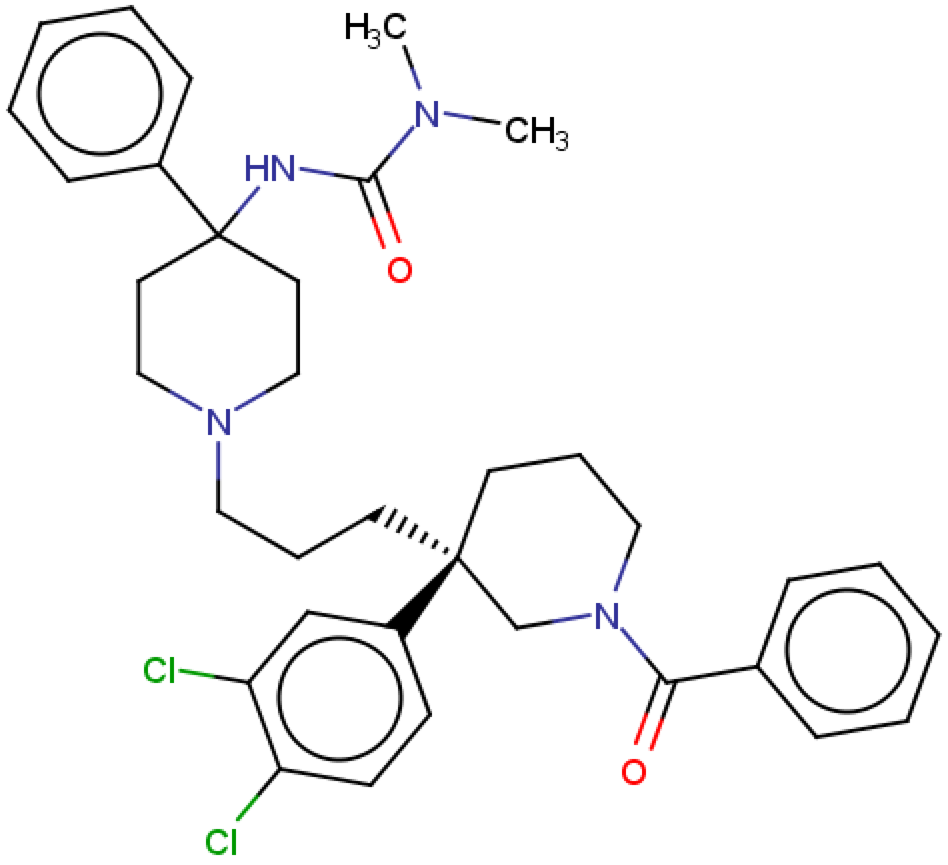

264618-44-2
Name: SSR146977
pIC50: 5.77
Rank: 90
Classes: No defined

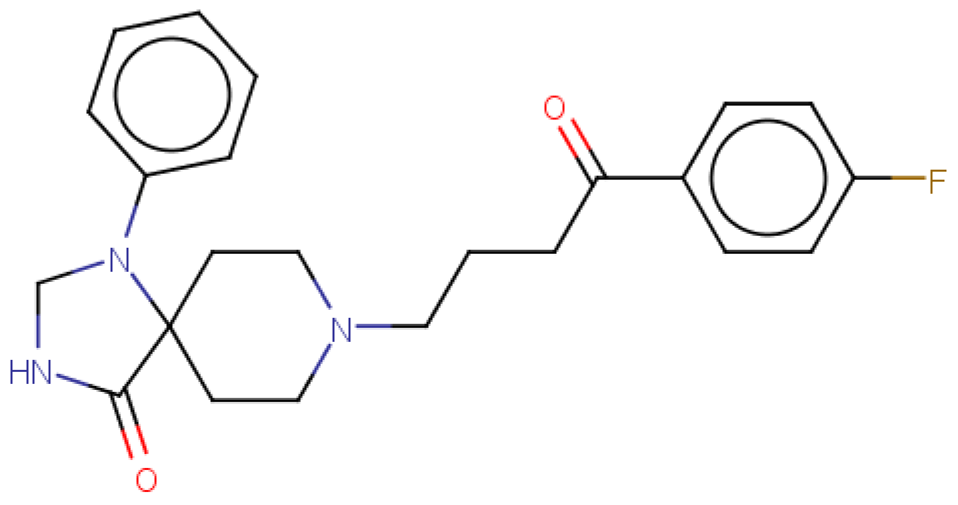

749-02-0
Name: Spiperone
pIC50: 5.75
Rank: 91
Classes: No defined

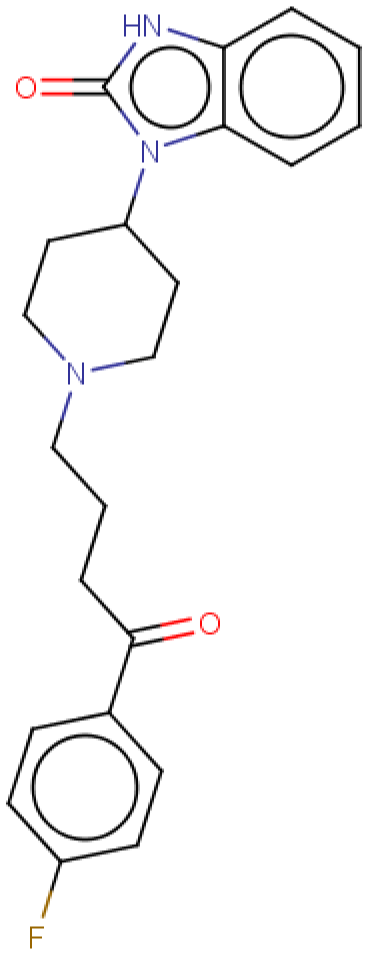

2062-84-2
Name: Benperidol
pIC50: 5.75
Rank: 92
Classes: Drug

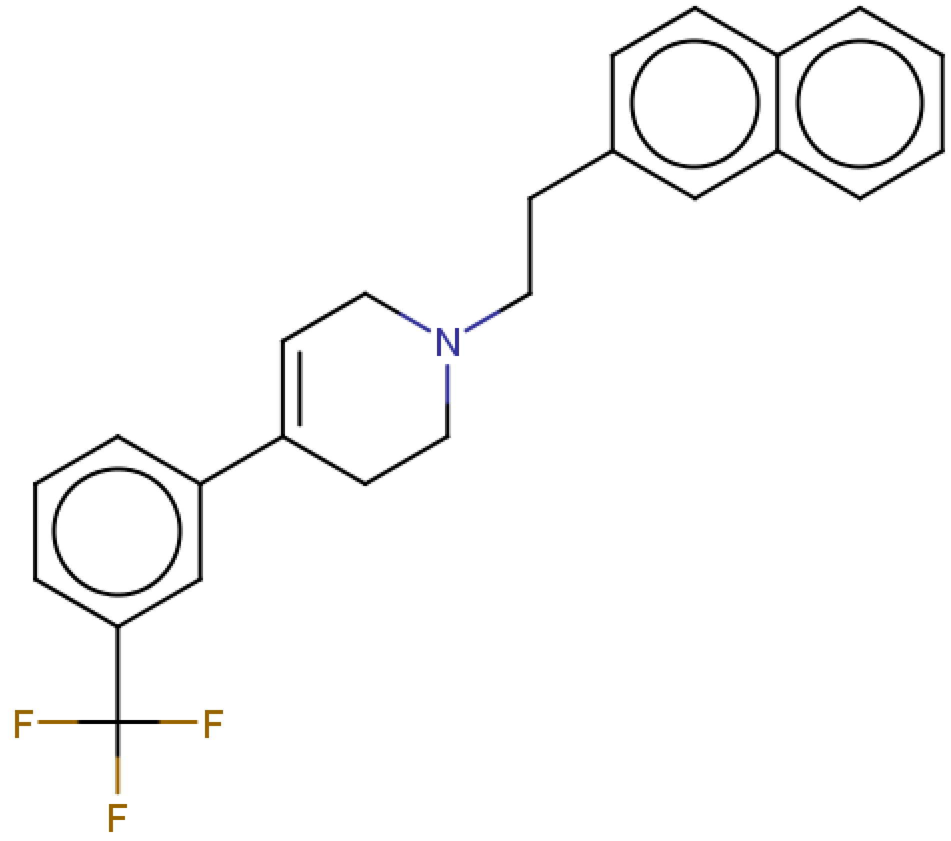

90494-79-4
Name: Xaliproden hydrochloride
pIC50: 5.75
Rank: 93
Classes: No defined

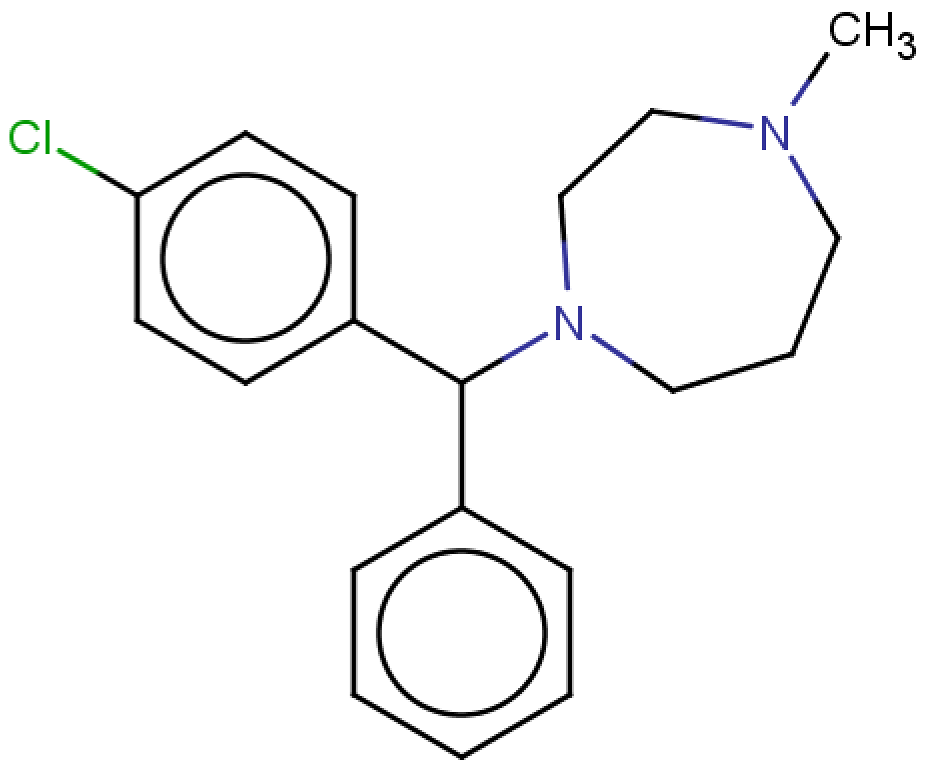

848-53-3
Name: Homochlorcyclizine
pIC50: 5.74
Rank: 94
Classes: No defined

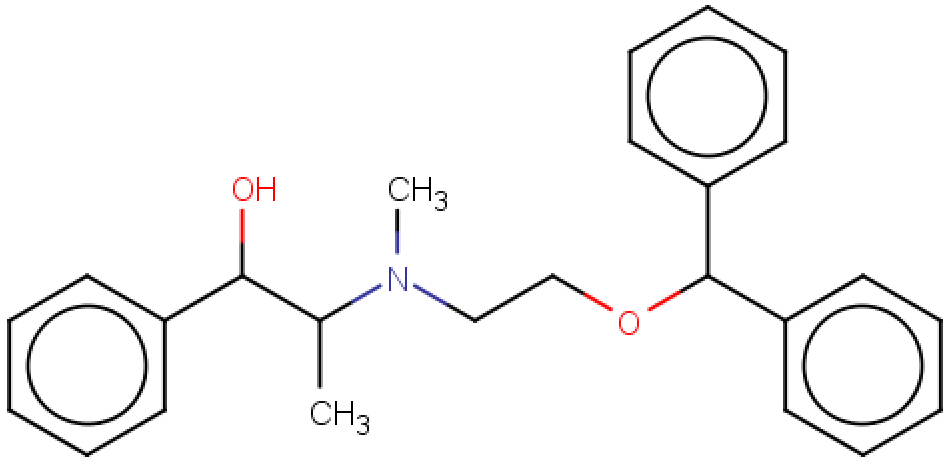

14587-50-9
Name: Difeterol
pIC50: 5.74
Rank: 95
Classes: No defined

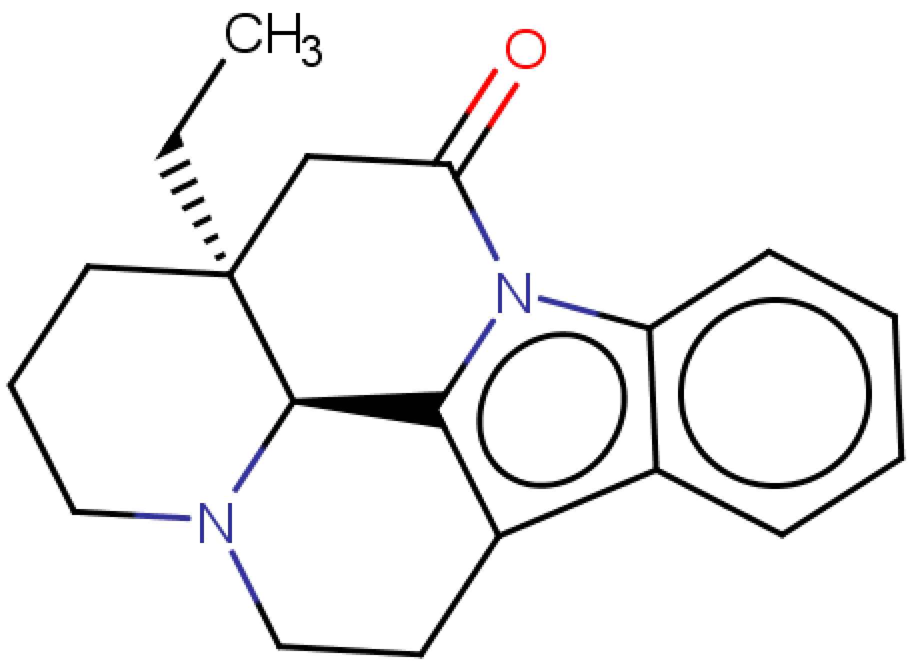

4880-88-0
Name: (-)-Eburnamonine
pIC50: 5.72
Rank: 96
Classes: No defined

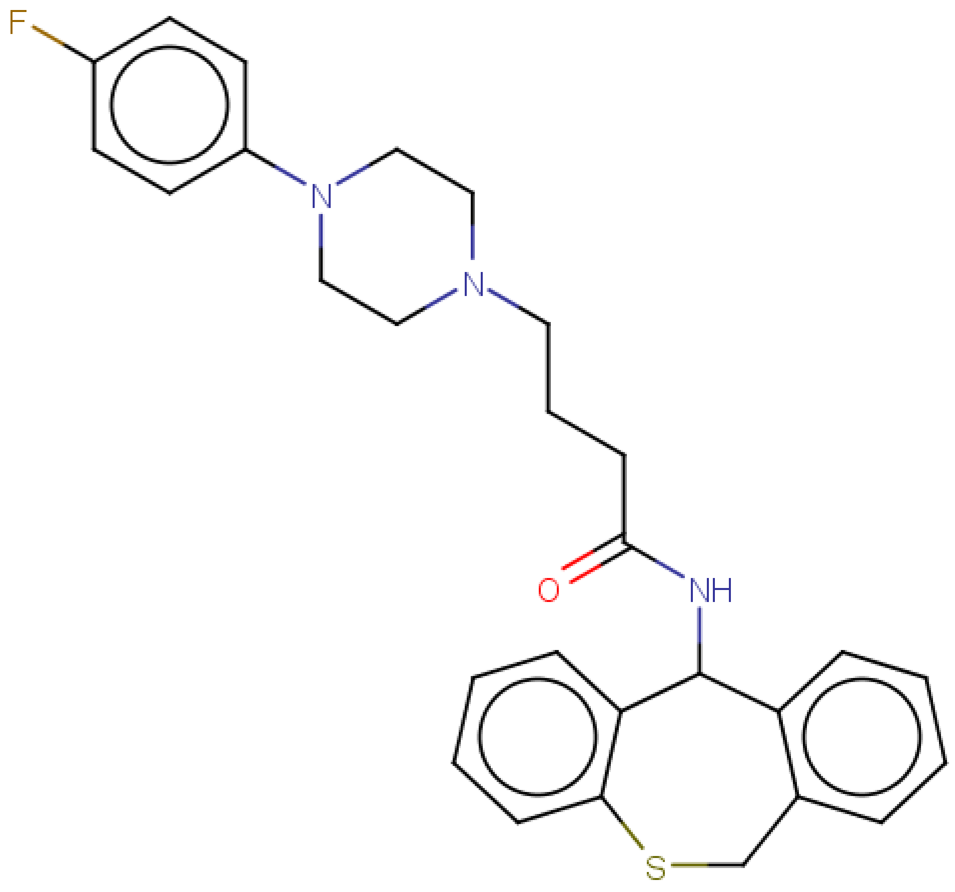

103377-41-9
Name: Monatepil
pIC50: 5.7
Rank: 97
Classes: No defined

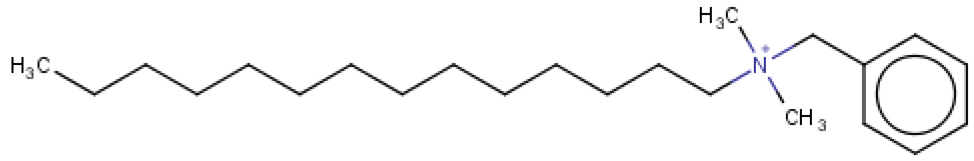

147228-81-7
Name: Benzyldimethyltetradecylammonium chloride
pIC50: 5.69
Rank: 98
Classes: No defined

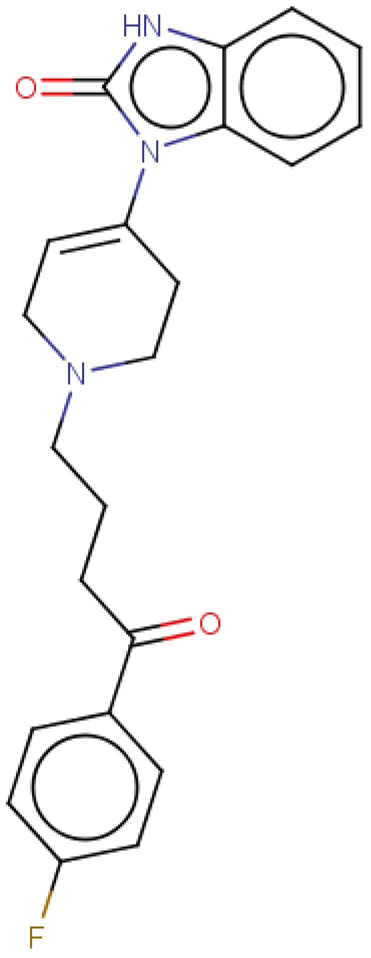

548-73-2
Name: Droperidol
pIC50: 5.65
Rank: 99
Classes: Drug

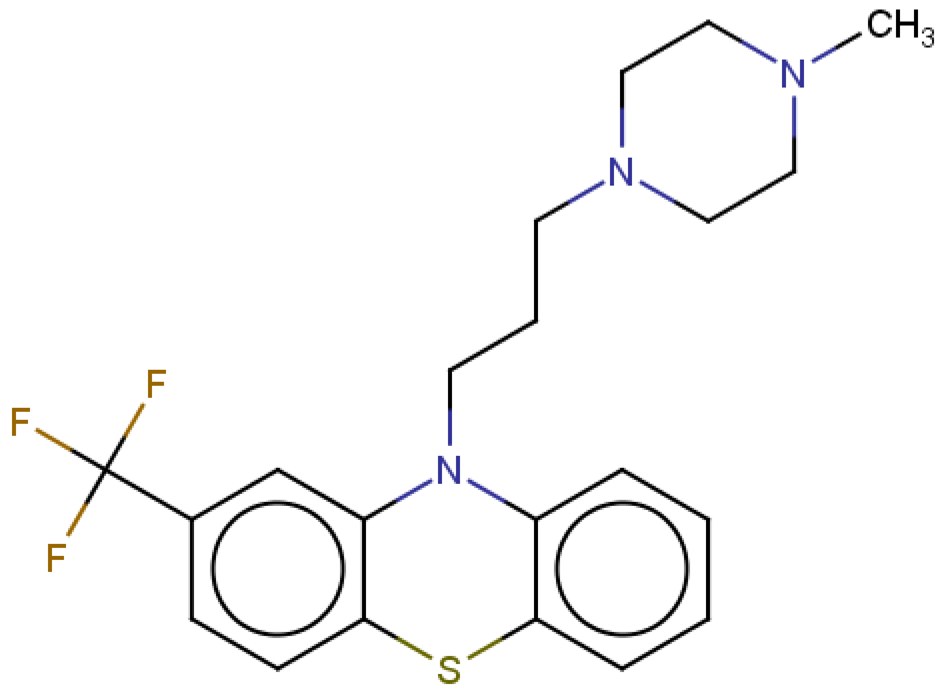

440-17-5
Name: Fluoperazine dihydrochloride
pIC50: 5.65
Rank: 100
Classes: No defined

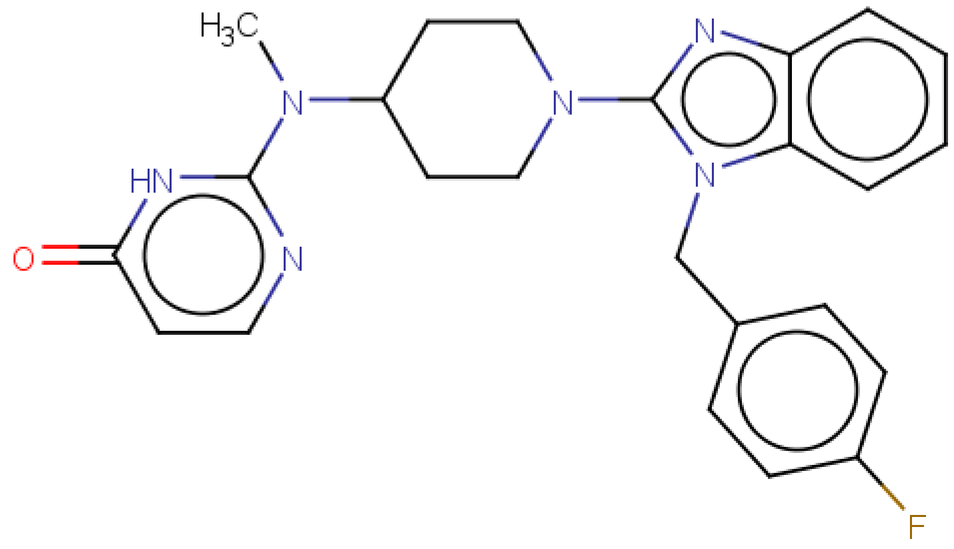

108612-45-9
Name: Mizolastine
pIC50: 5.65
Rank: 101
Classes: Drug

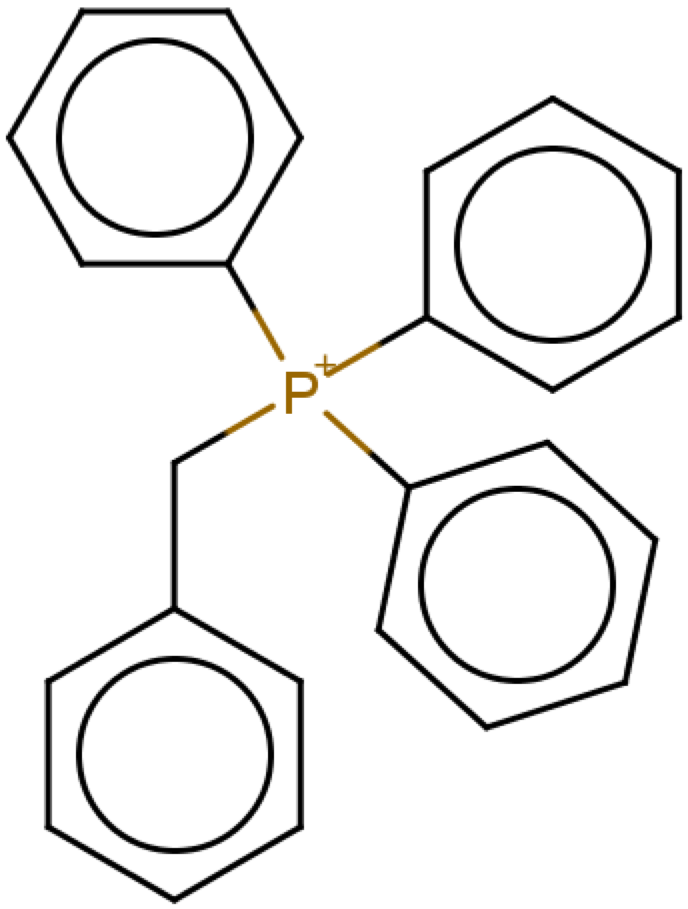

1100-88-5
Name: Benzyltriphenylphosphonium chloride
pIC50: 5.64
Rank: 102
Classes: No defined

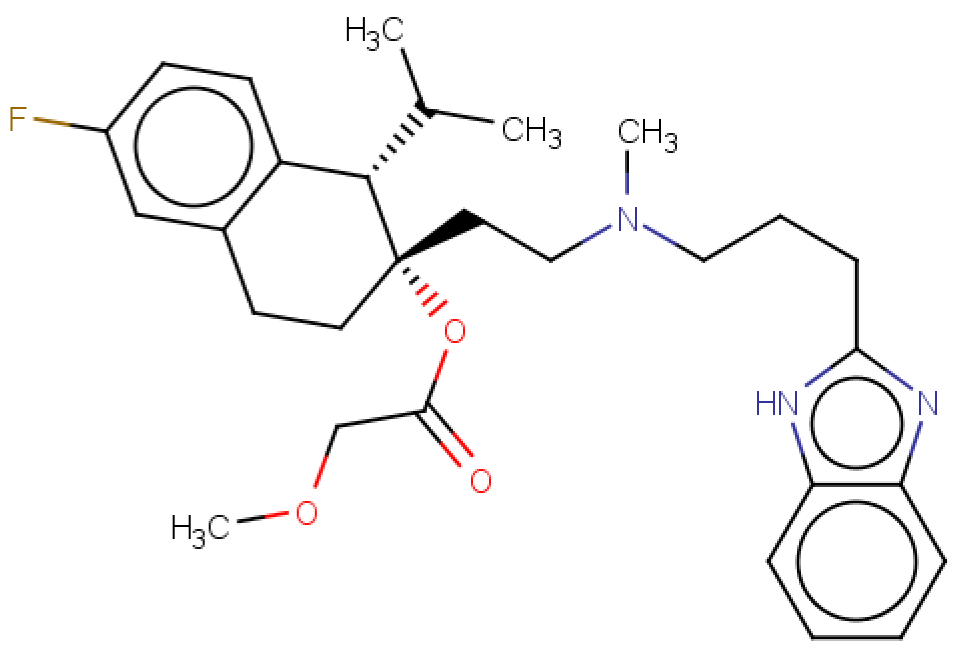

116666-63-8
Name: Mibefradil dihydrochloride
pIC50: 5.64
Rank: 103
Classes: No defined

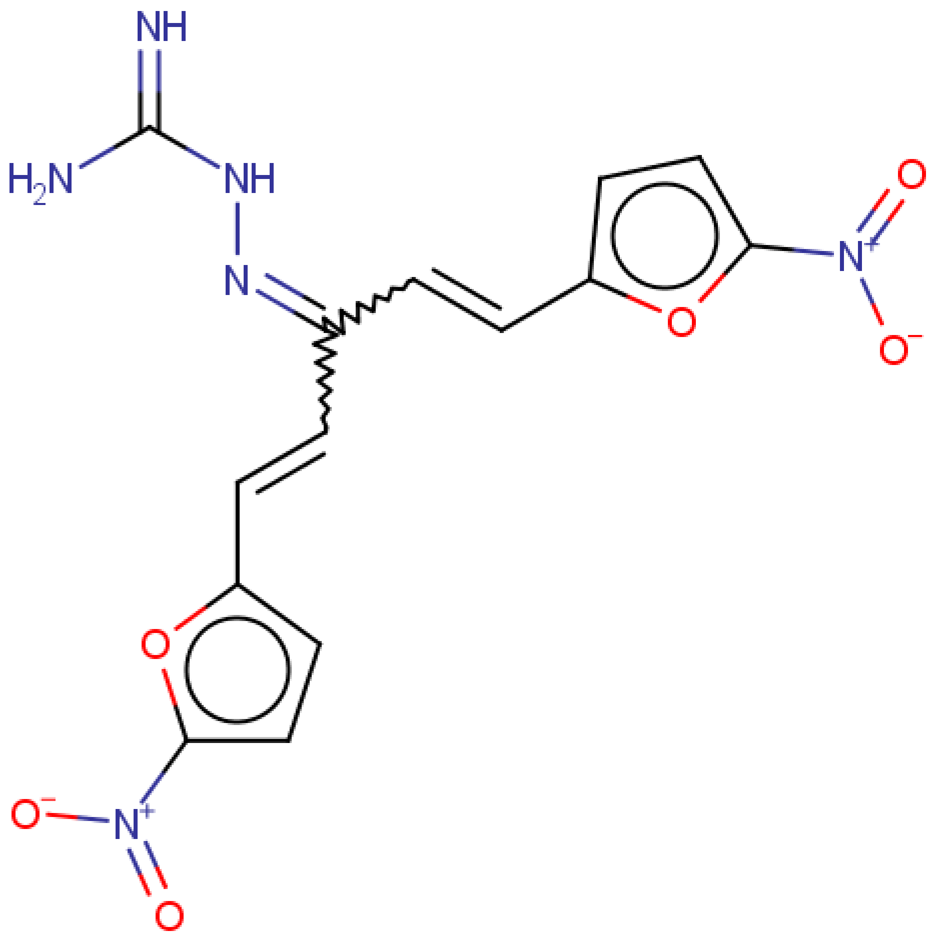

804-36-4
Name: Nitrovin
pIC50: 5.64
Rank: 104
Classes: No defined

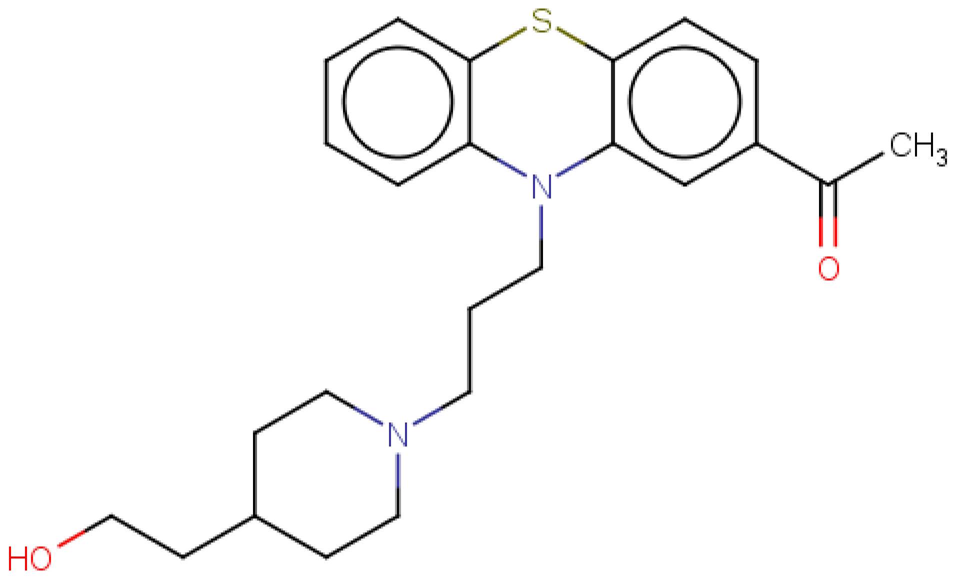

3819-00-9
Name: Piperacetazine
pIC50: 5.62
Rank: 105
Classes: No defined

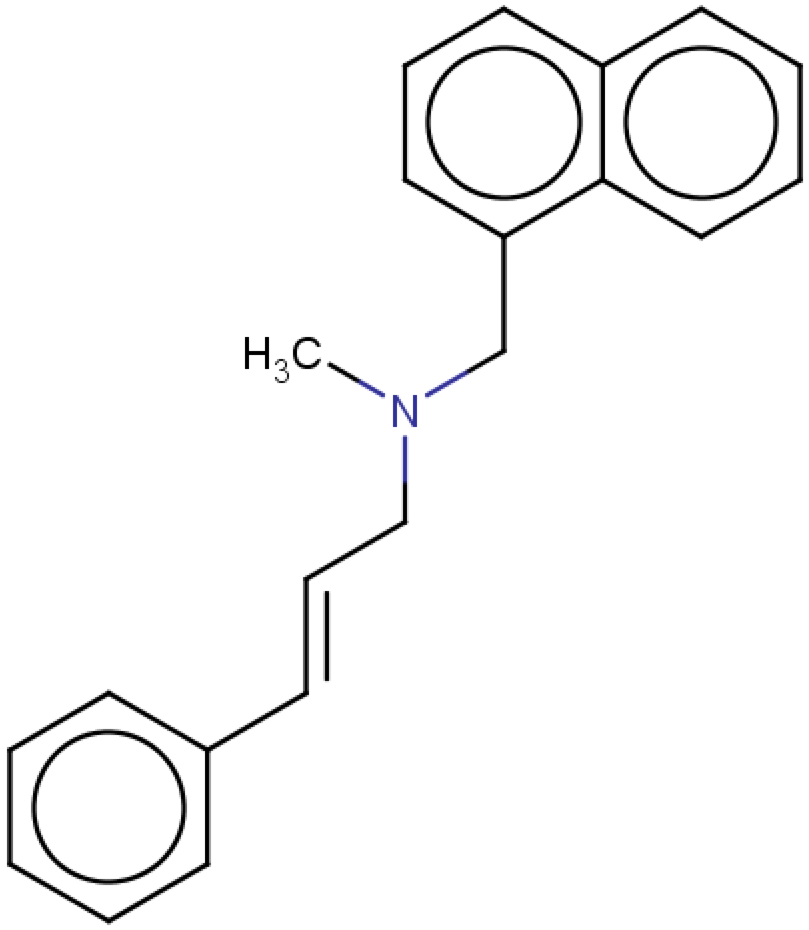

65473-14-5
Name: Naftifine hydrochloride
pIC50: 5.62
Rank: 106
Classes: No defined

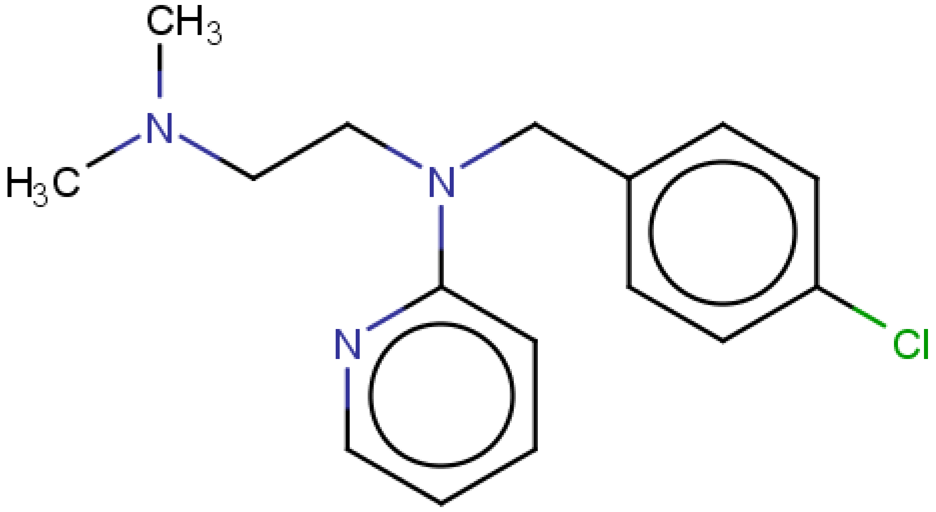

6170-42-9
Name: Chloropyramine hydrochloride
pIC50: 5.62
Rank: 107
Classes: No defined

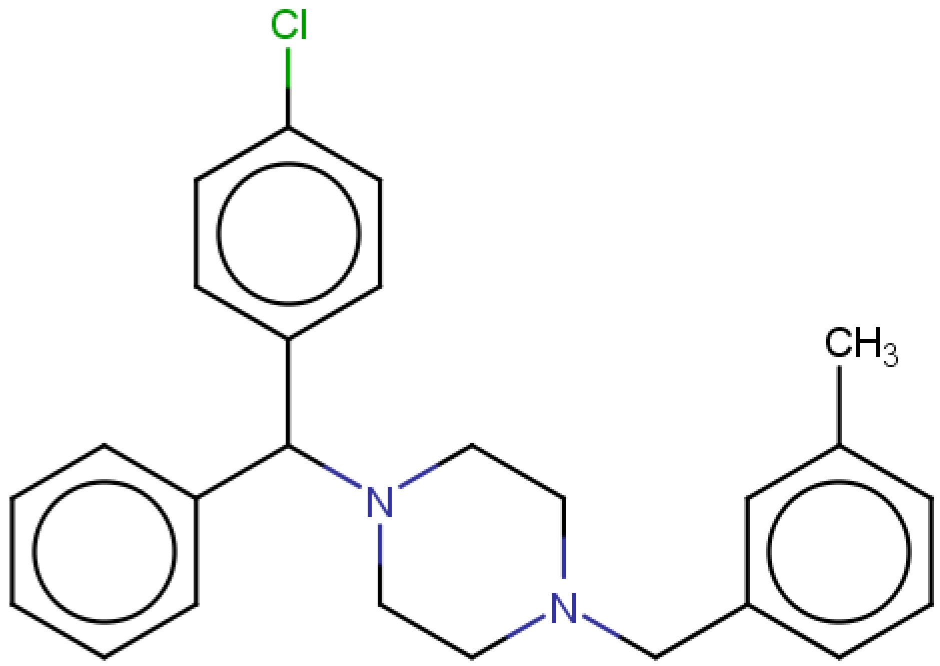

1104-22-9
Name: Meclozine dihydrochloride
pIC50: 5.6
Rank: 108
Classes: No defined

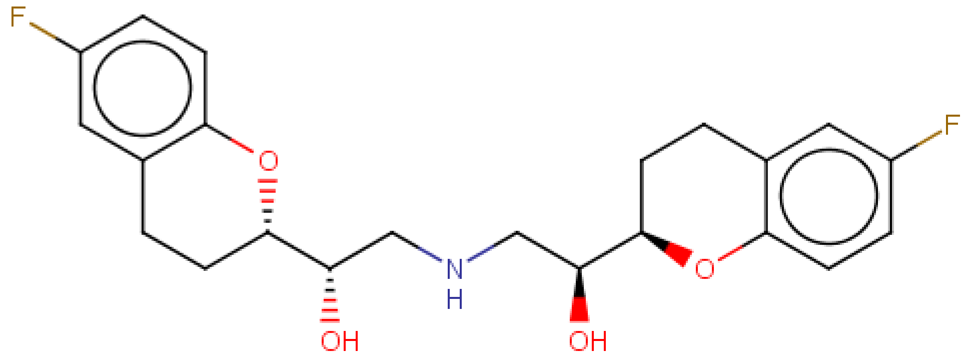

169293-50-9
Name: rac Nebivolol hydrochloride
pIC50: 5.6
Rank: 109
Classes: No defined

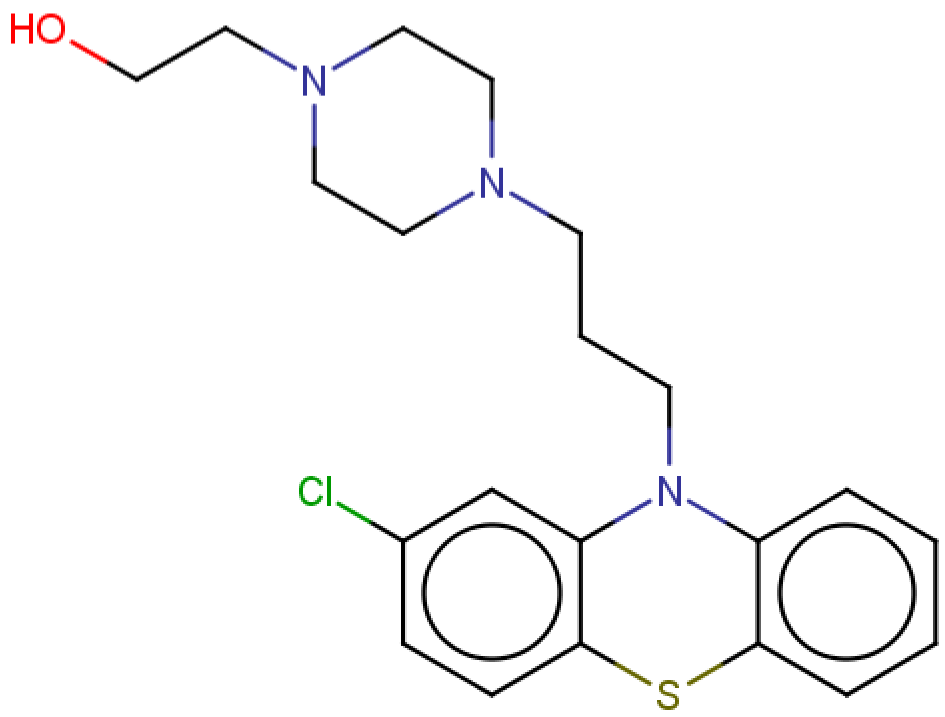

58-39-9
Name: Perphenazine
pIC50: 5.59
Rank: 110
Classes: Drug

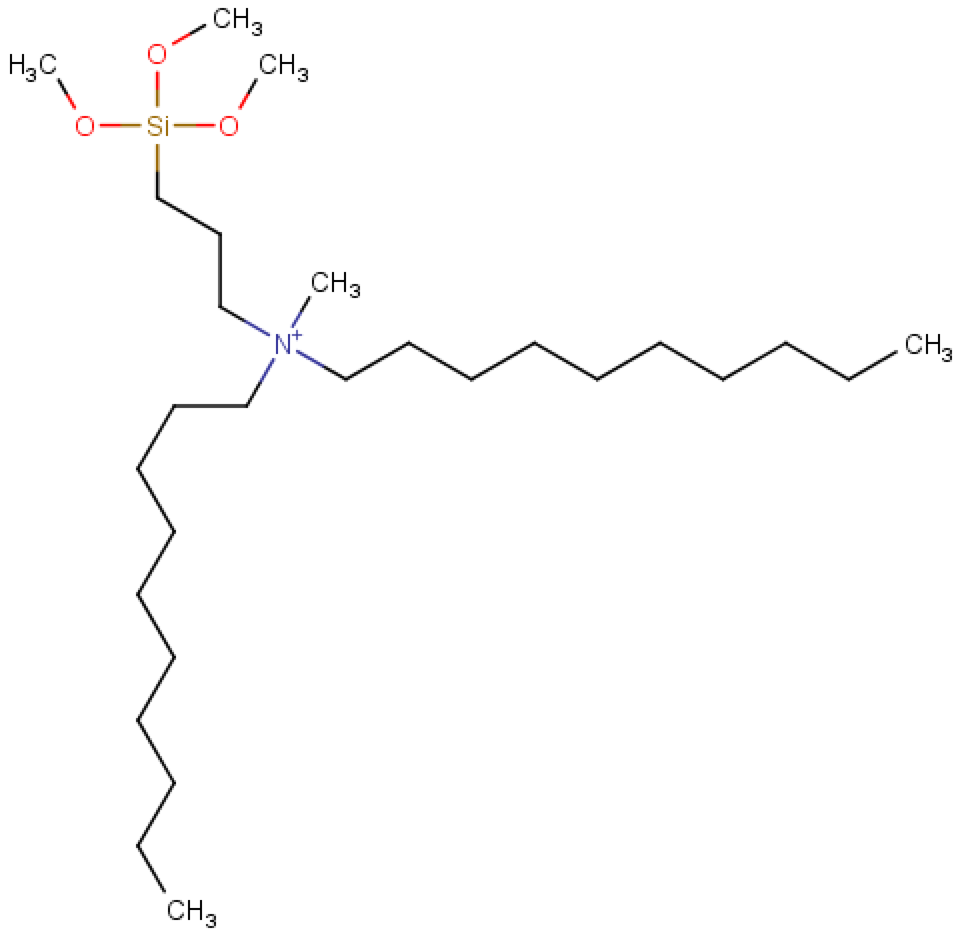

68959-20-6
Name: Disiquonium chloride
pIC50: 5.58
Rank: 111
Classes: antimicrobial

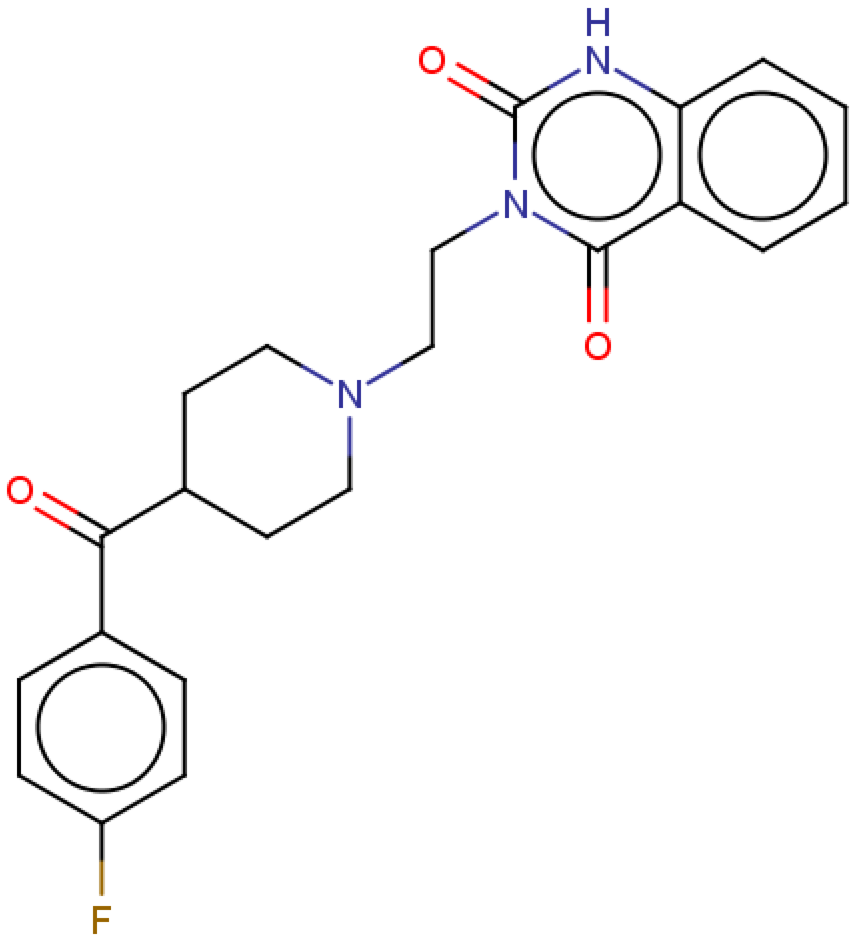

74050-98-9
Name: Ketanserin
pIC50: 5.57
Rank: 112
Classes: Drug

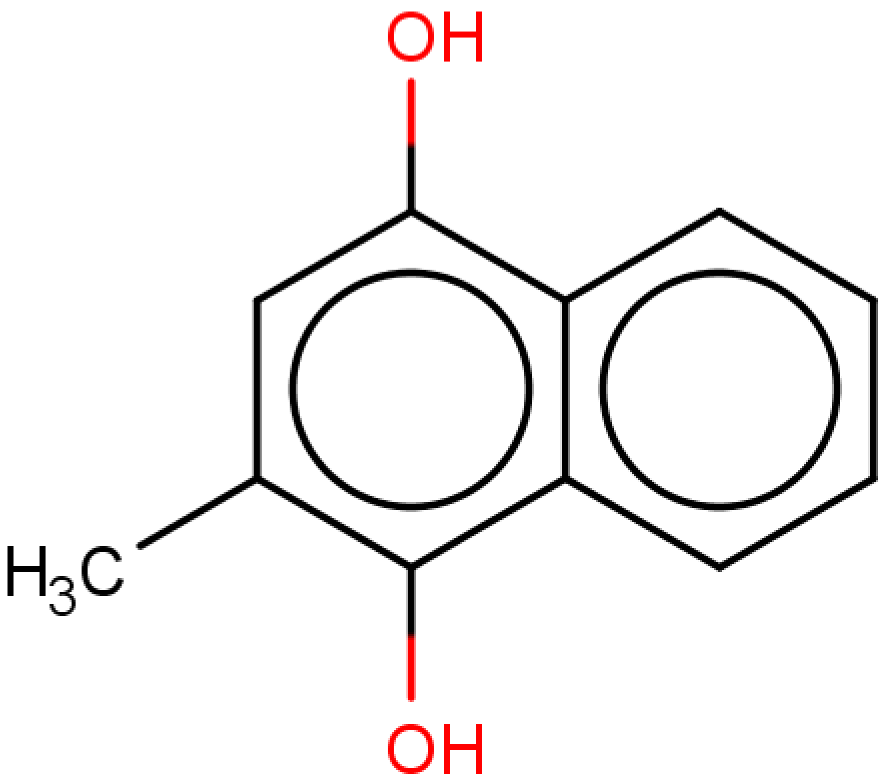

481-85-6
Name: Menadiol
pIC50: 5.57
Rank: 113
Classes: No defined

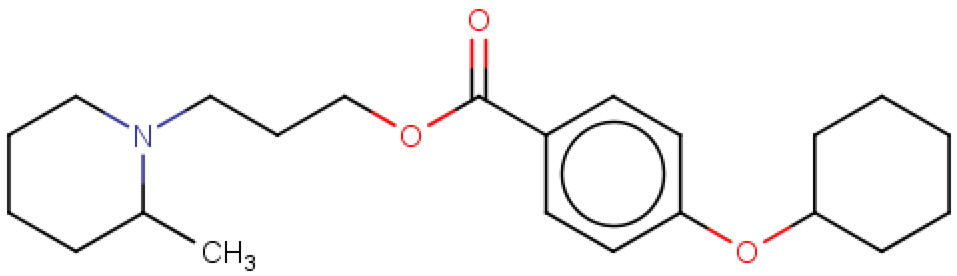

139-62-8
Name: Cyclomethycaine
pIC50: 5.57
Rank: 114
Classes: No defined

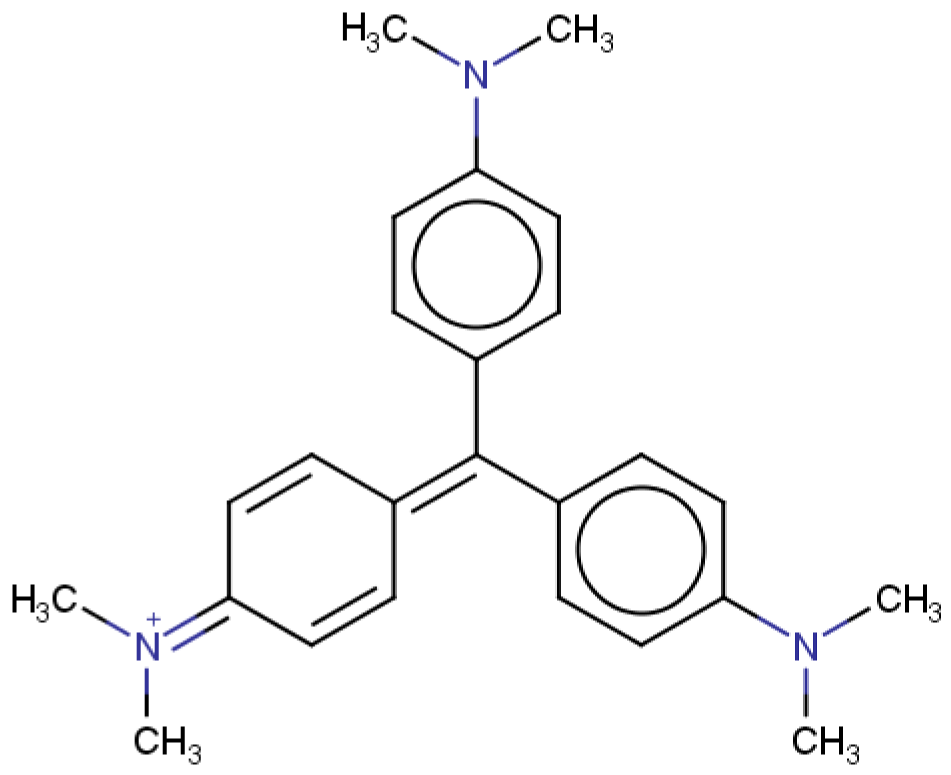

548-62-9
Name: Gentian Violet
pIC50: 5.55
Rank: 115
Classes: TSCA

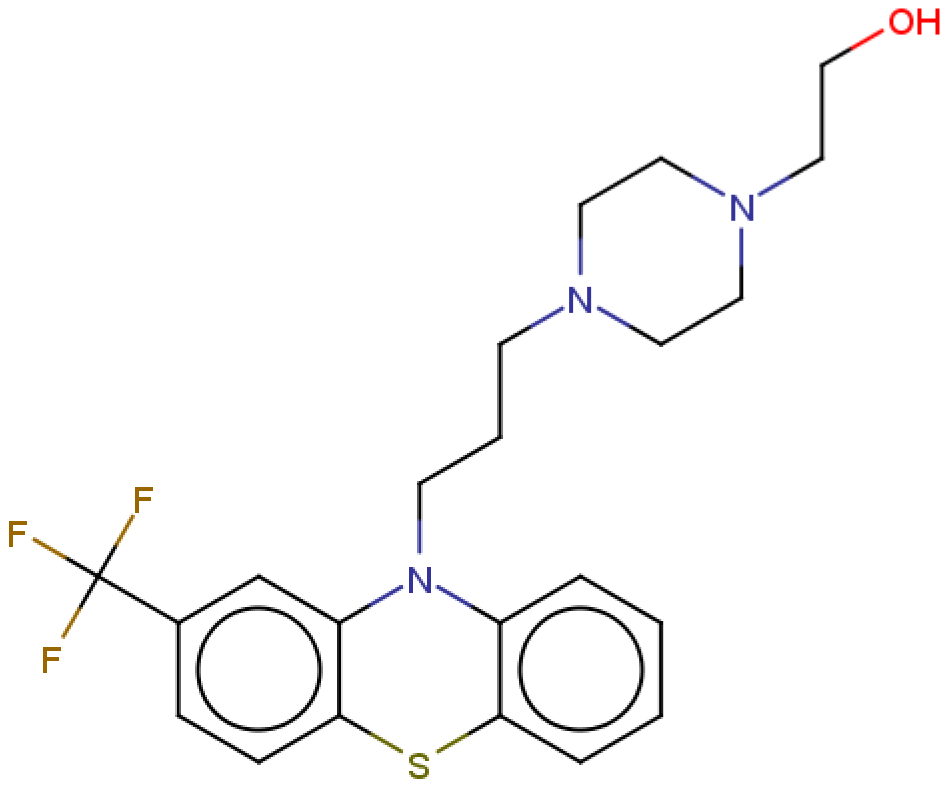

69-23-8
Name: Fluphenazine
pIC50: 5.55
Rank: 116
Classes: Drug

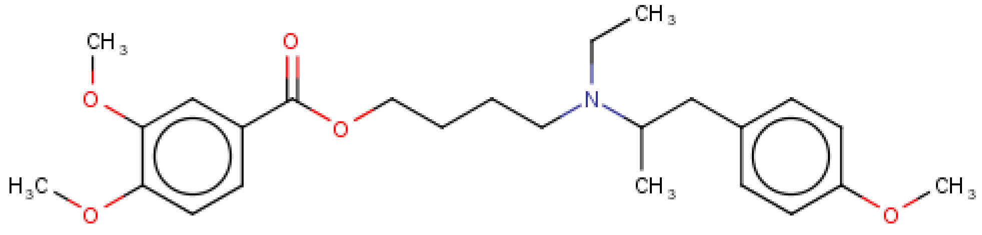

2753-45-9
Name: Mebeverine hydrochloride
pIC50: 5.55
Rank: 117
Classes: No defined

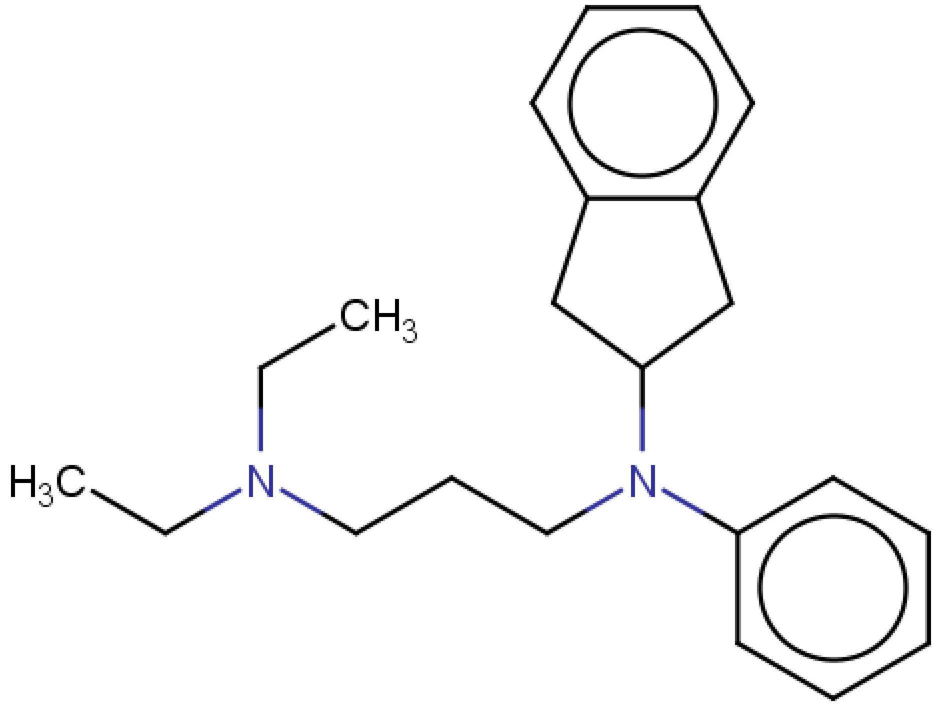

33237-74-0
Name: Aprindine hydrochloride
pIC50: 5.55
Rank: 118
Classes: No defined

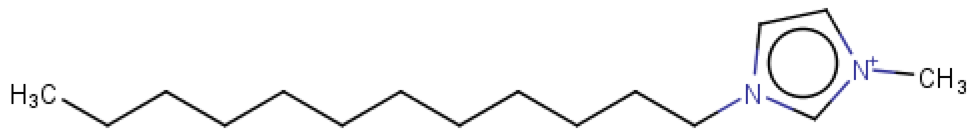

114569-84-5
Name: 1-Dodecyl-3-methylimidazolium chloride
pIC50: 5.54
Rank: 119
Classes: No defined

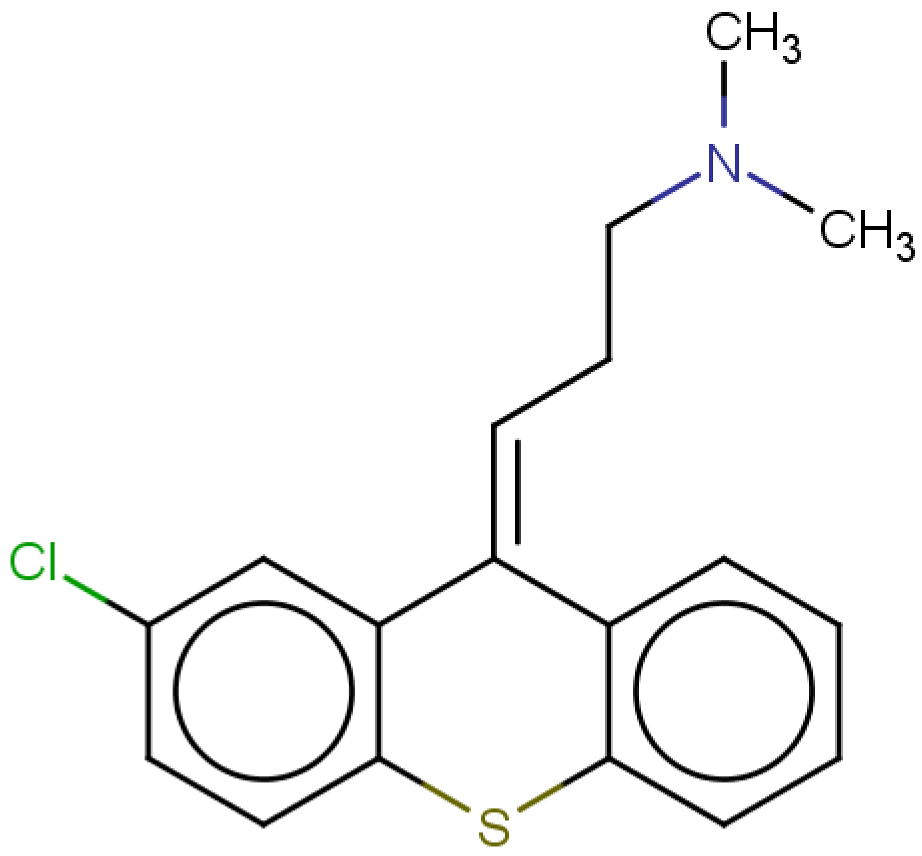

113-59-7
Name: Chlorprothixene
pIC50: 5.54
Rank: 120
Classes: Drug

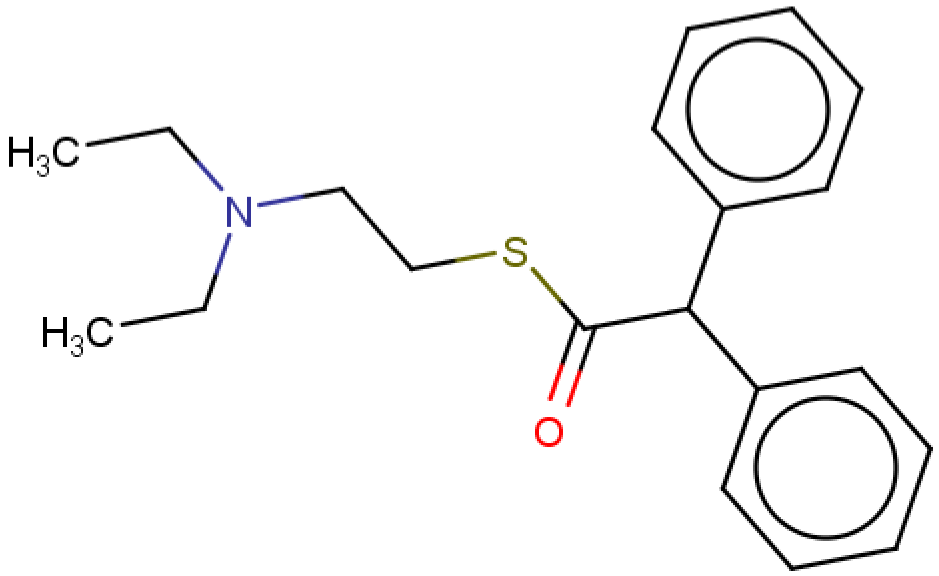

82-99-5
Name: Thiphenamil
pIC50: 5.52
Rank: 121
Classes: No defined

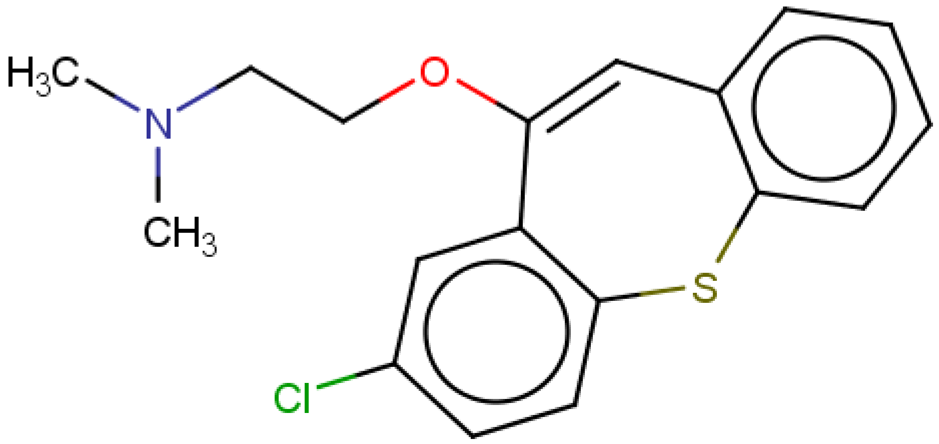

26615-21-4
Name: Zotepine
pIC50: 5.52
Rank: 122
Classes: Drug

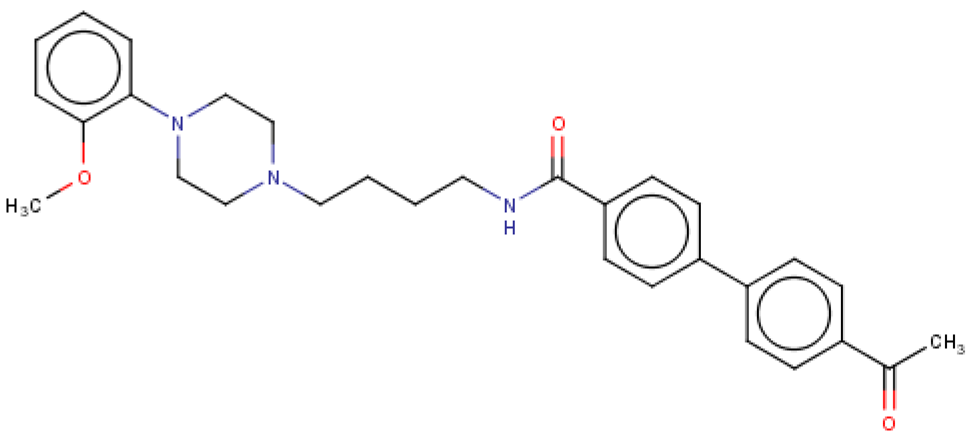

162408-66-4
Name: GR 103691
pIC50: 5.52
Rank: 123
Classes: No defined

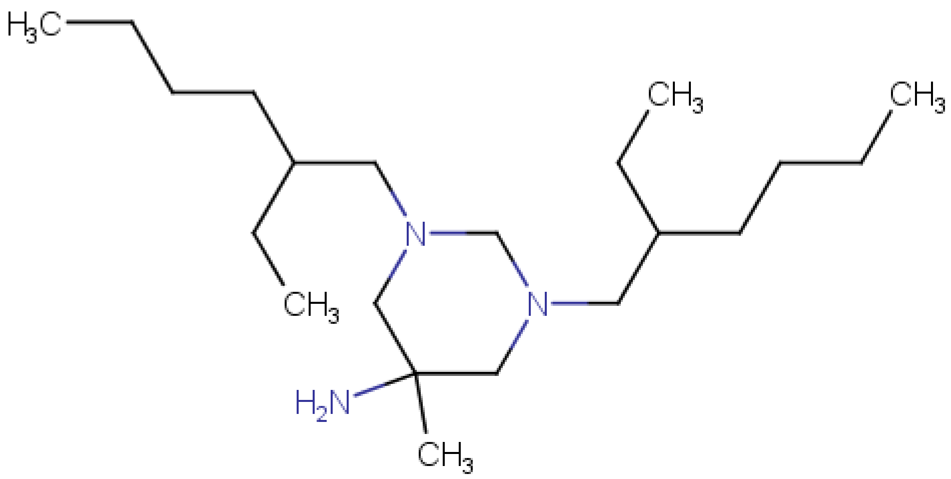

141-94-6
Name: Hexetidine
pIC50: 5.52
Rank: 124
Classes: solvent--Drug

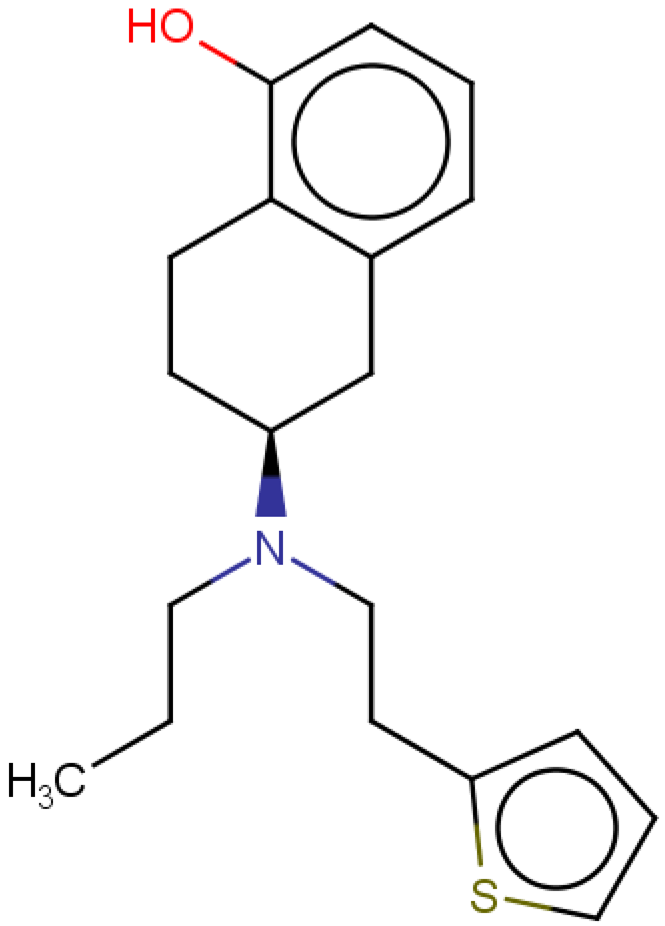

99755-59-6
Name: Rotigotine
pIC50: 5.52
Rank: 125
Classes: Drug

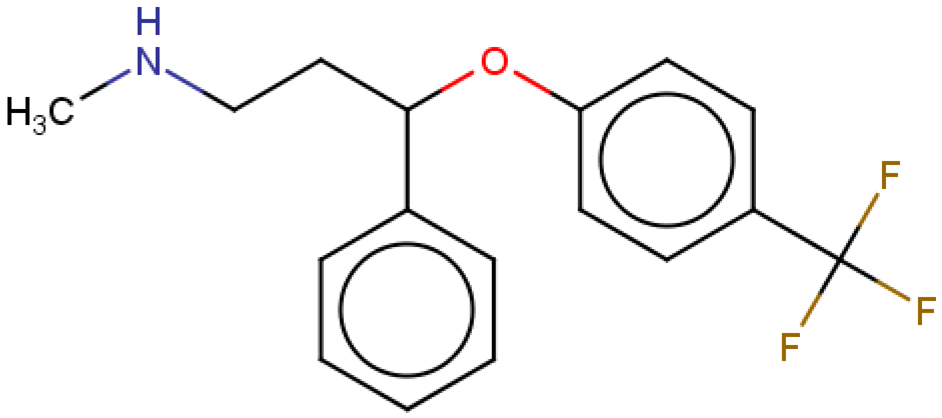

56296-78-7
Name: Fluoxetine hydrochloride
pIC50: 5.52
Rank: 126
Classes: No defined

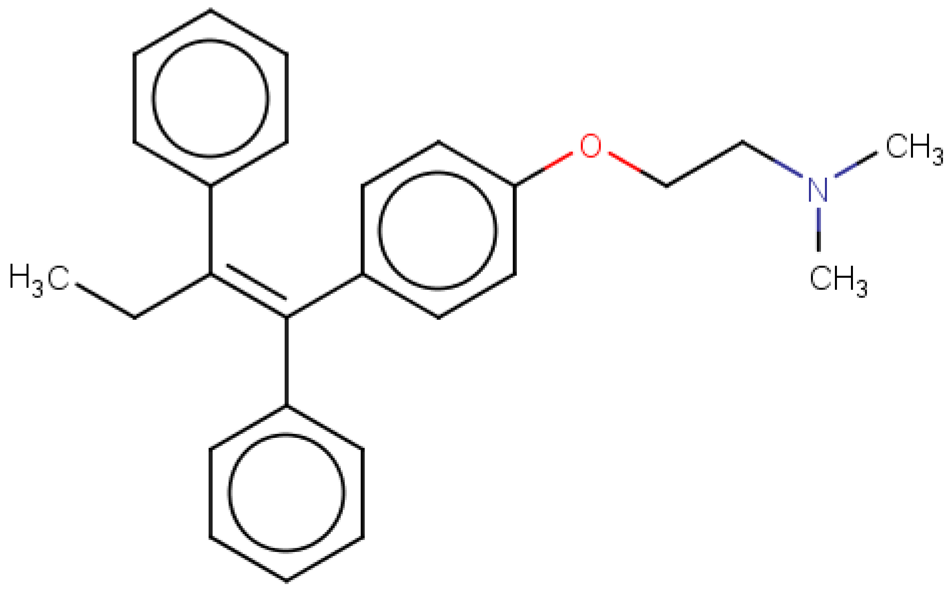

10540-29-1
Name: Tamoxifen
pIC50: 5.51
Rank: 127
Classes: Drug

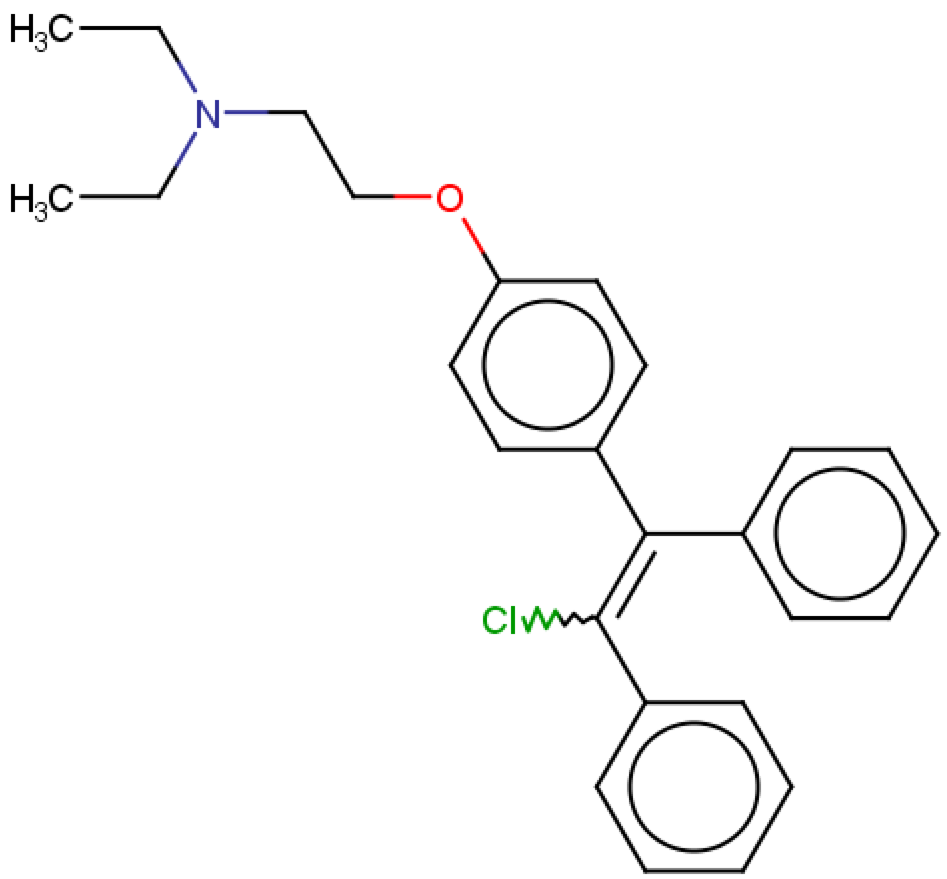

911-45-5
Name: Clomiphene
pIC50: 5.5
Rank: 128
Classes: Drug

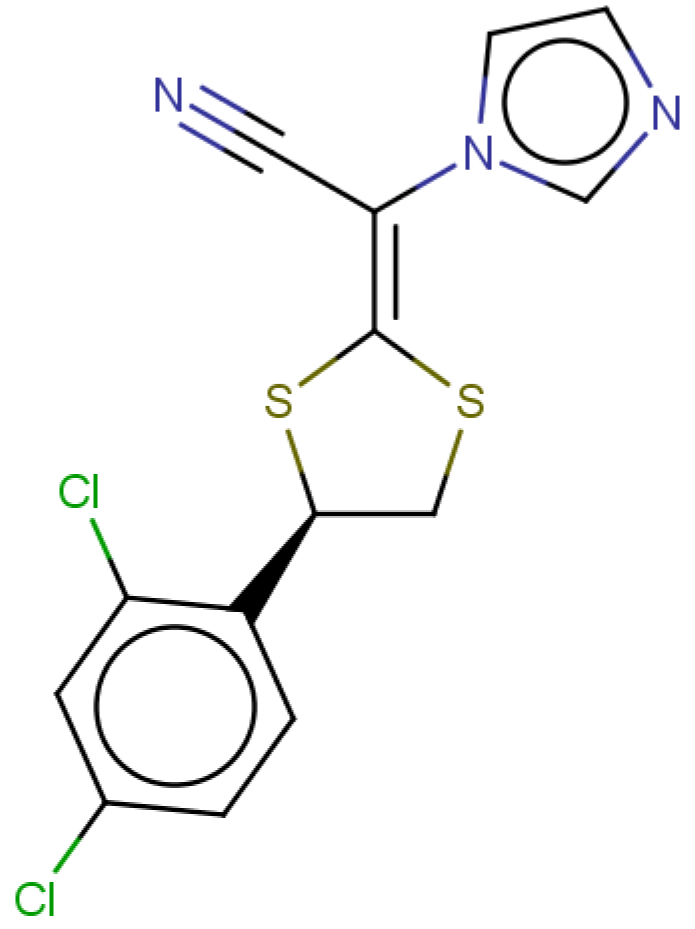

187164-19-8
Name: Luliconazole
pIC50: 5.5
Rank: 129
Classes: Drug

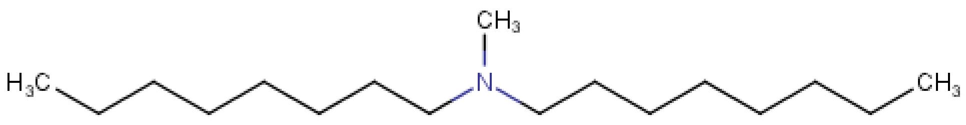

4455-26-9
Name: N-Methyldioctylamine
pIC50: 5.49
Rank: 130
Classes: TSCA

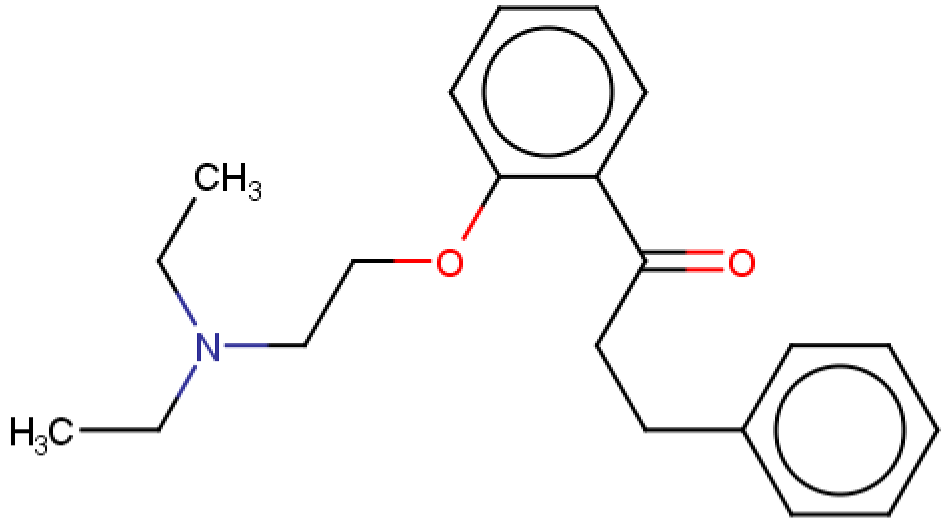

90-54-0
Name: Etafenone
pIC50: 5.49
Rank: 131
Classes: No defined

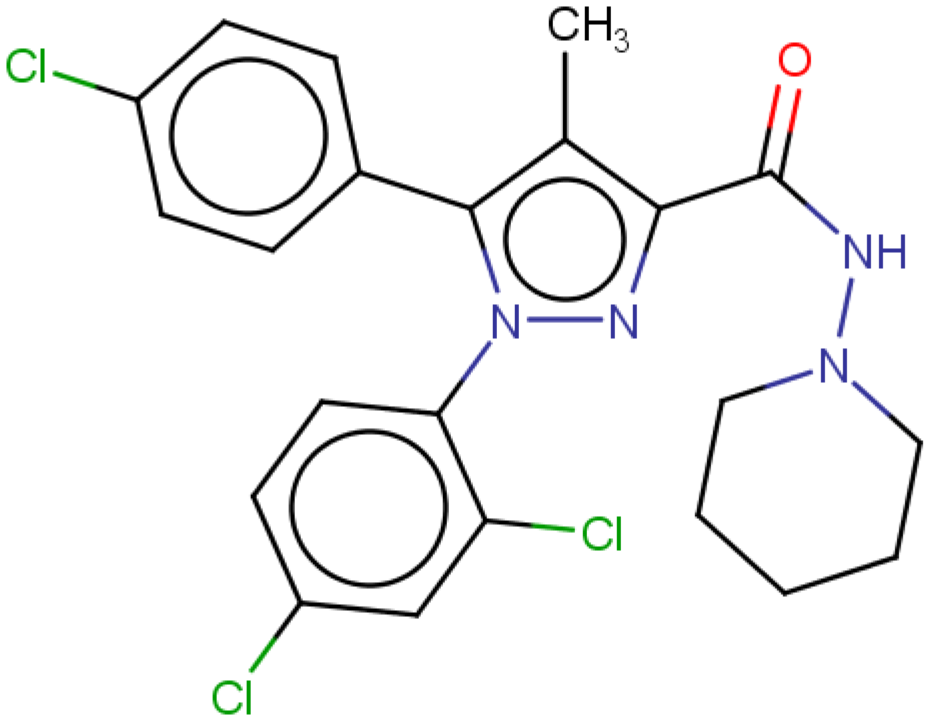

168273-06-1
Name: Rimonabant
pIC50: 5.49
Rank: 132
Classes: Drug

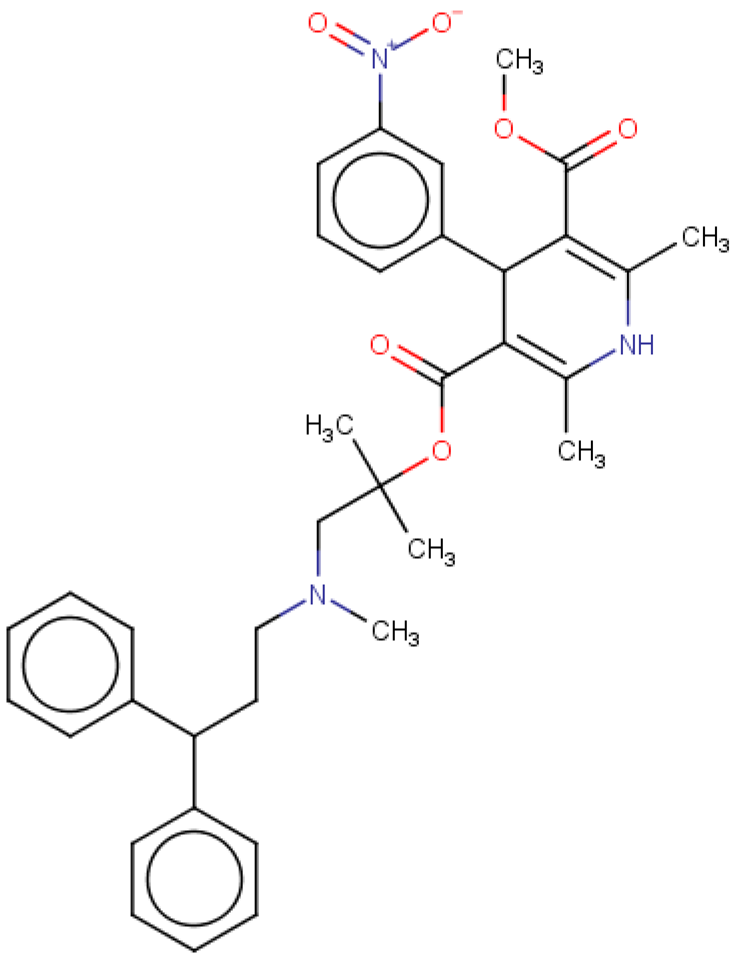

132866-11-6
Name: Lercanidipine hydrochloride
pIC50: 5.49
Rank: 133
Classes: No defined

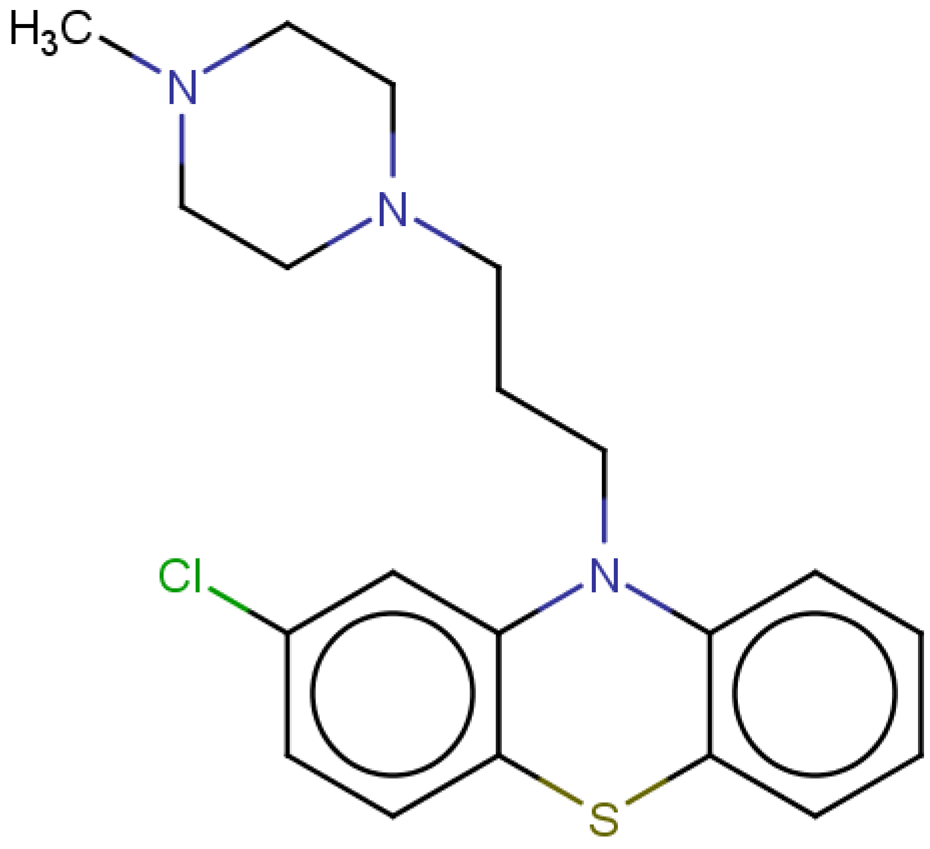

84-02-6
Name: Prochlorperazine dimaleate
pIC50: 5.49
Rank: 134
Classes: No defined

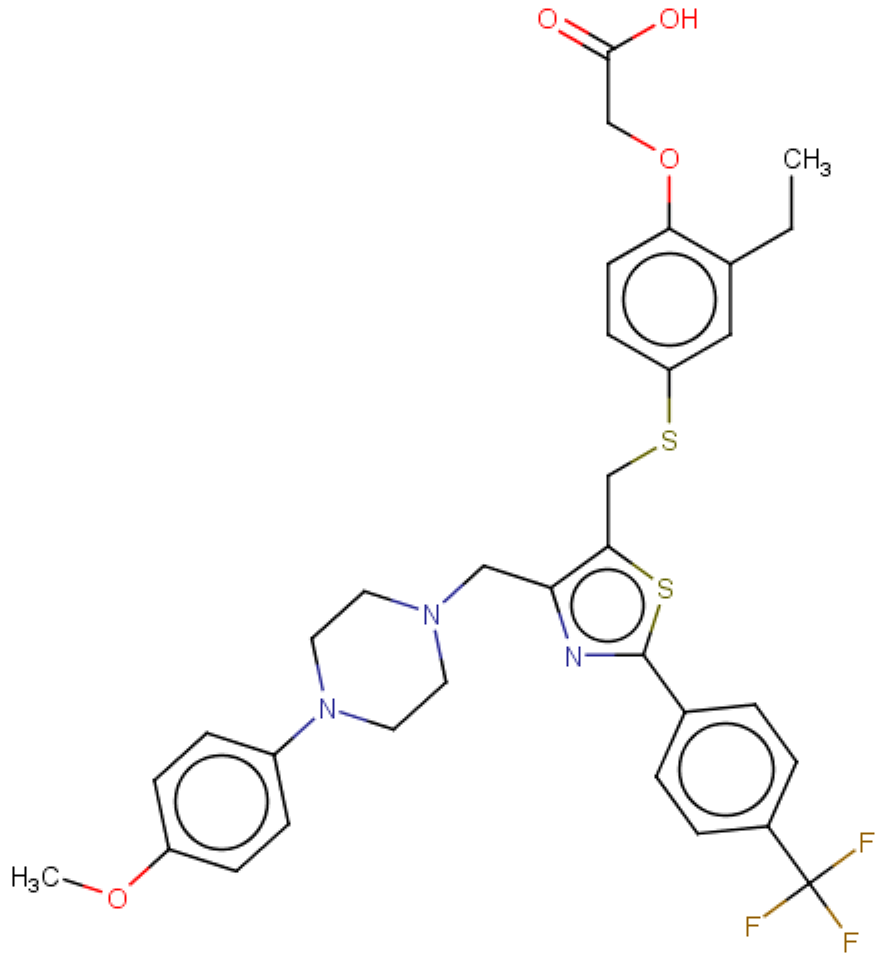

444610-91-7
Name: PharmaGSID_47315
pIC50: 5.48
Rank: 135
Classes: No defined

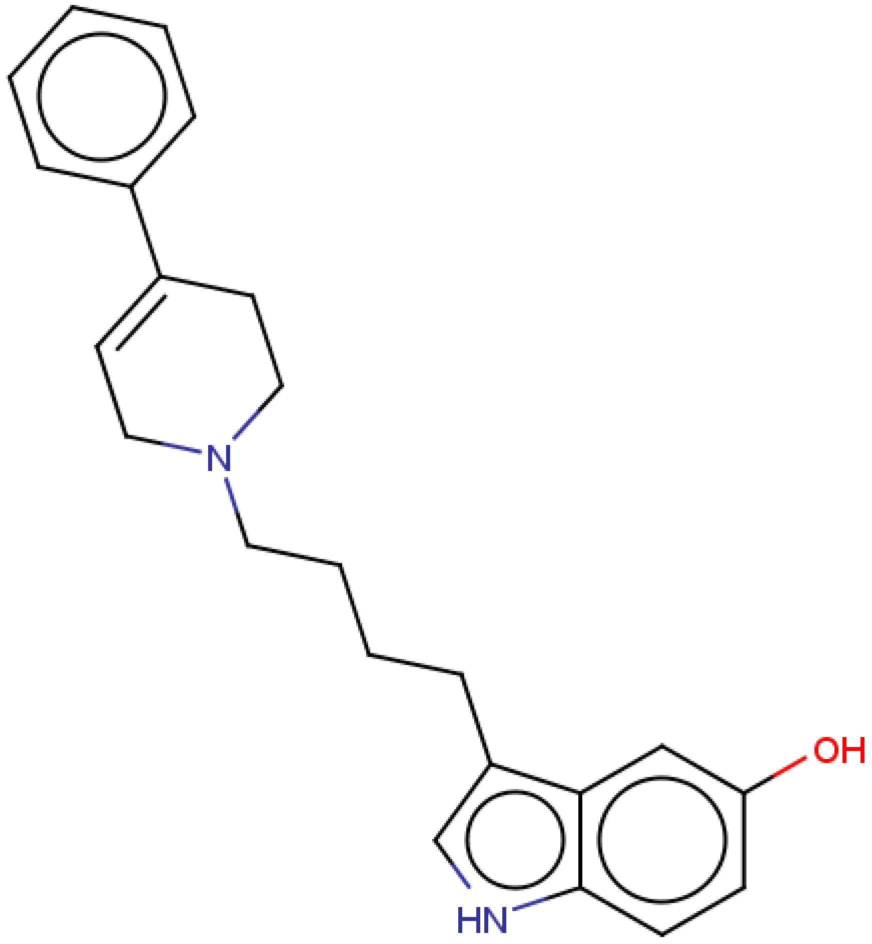

112192-04-8
Name: Roxindole
pIC50: 5.47
Rank: 136
Classes: No defined

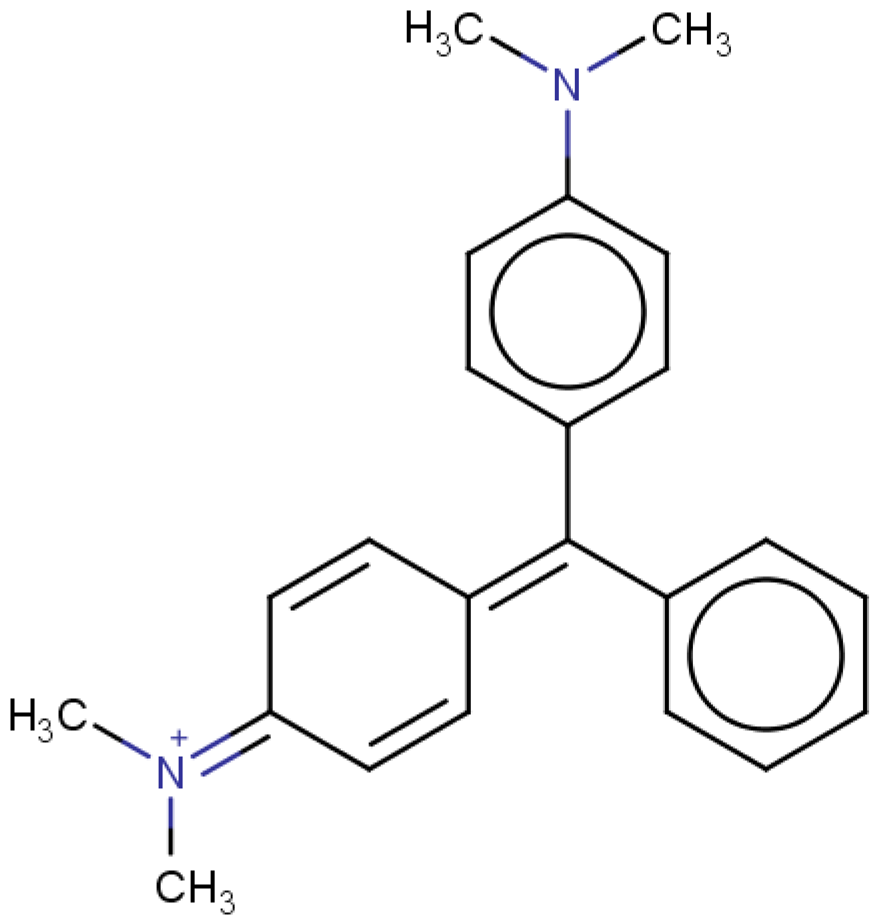

2437-29-8
Name: Malachite green oxalate
pIC50: 5.45
Rank: 137
Classes: No defined

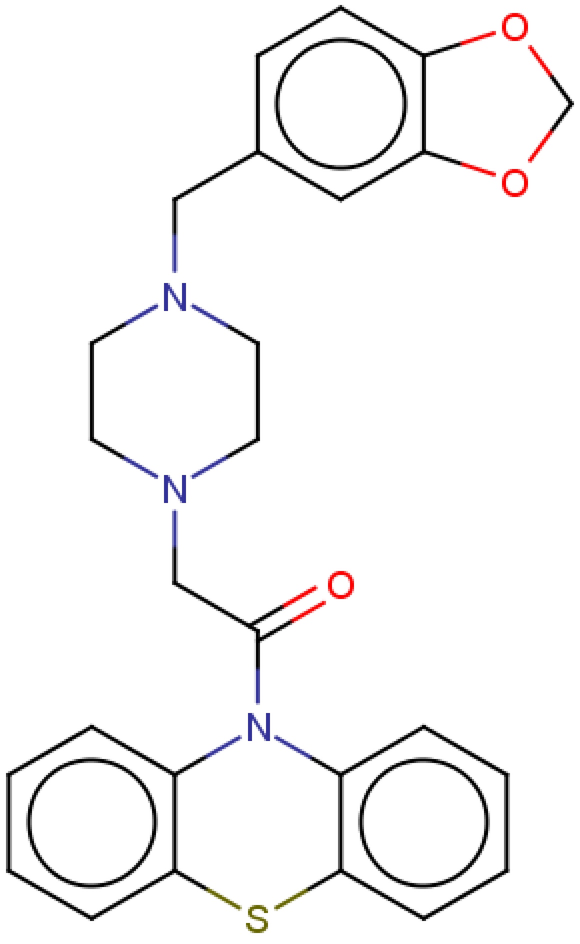

37561-27-6
Name: Fenoverine
pIC50: 5.45
Rank: 138
Classes: Drug

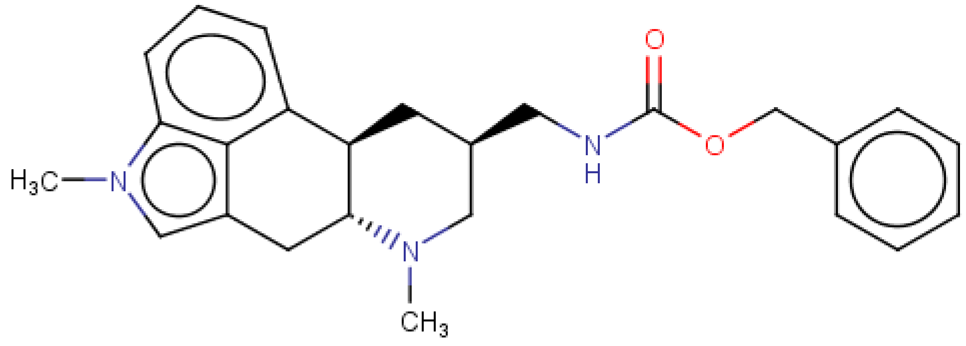

17692-51-2
Name: Metergoline
pIC50: 5.44
Rank: 139
Classes: No defined

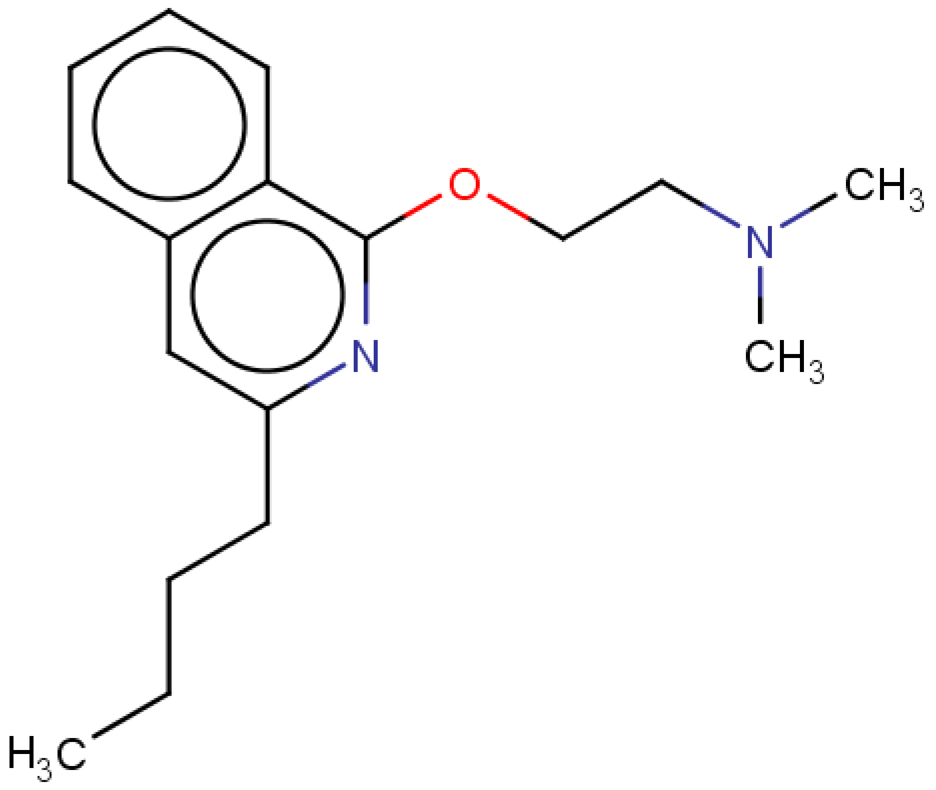

2773-92-4
Name: Dimethisoquin hydrochloride
pIC50: 5.44
Rank: 140
Classes: No defined

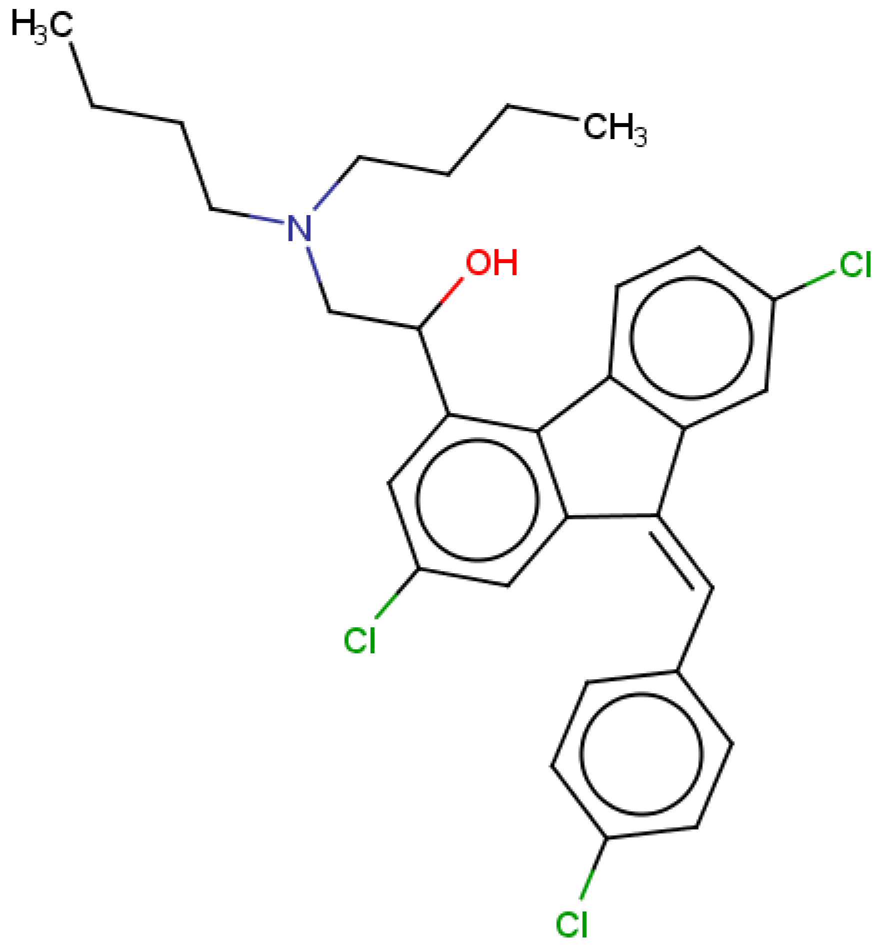

82186-77-4
Name: Lumefantrine
pIC50: 5.44
Rank: 141
Classes: Drug

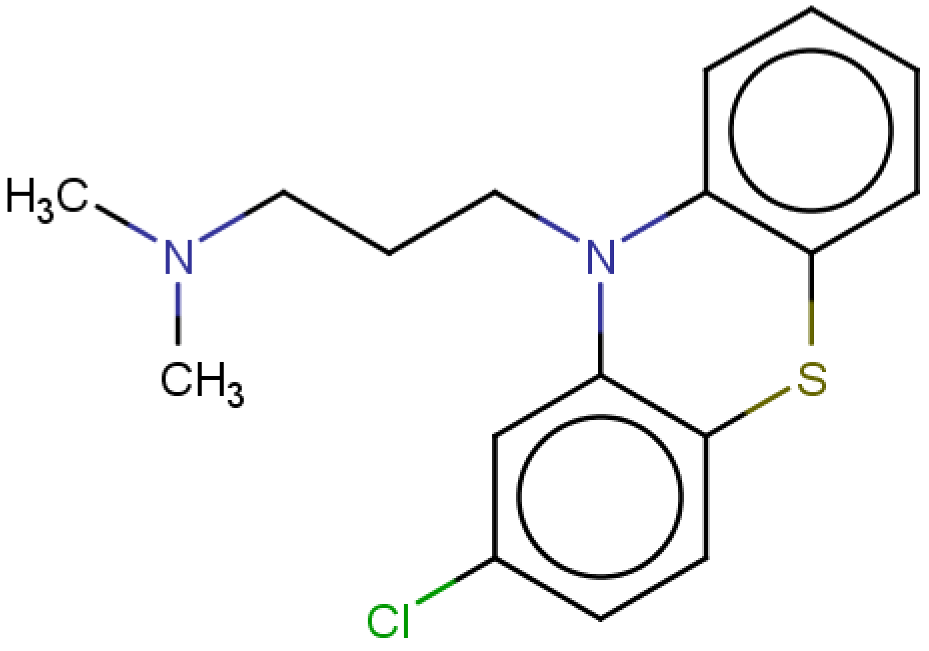

50-53-3
Name: Chlorpromazine
pIC50: 5.42
Rank: 142
Classes: Drug

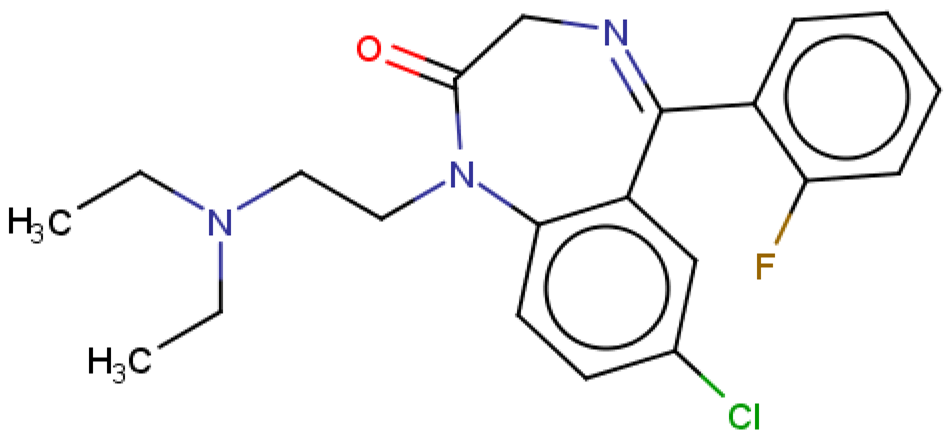

17617-23-1
Name: Flurazepam
pIC50: 5.42
Rank: 143
Classes: Drug

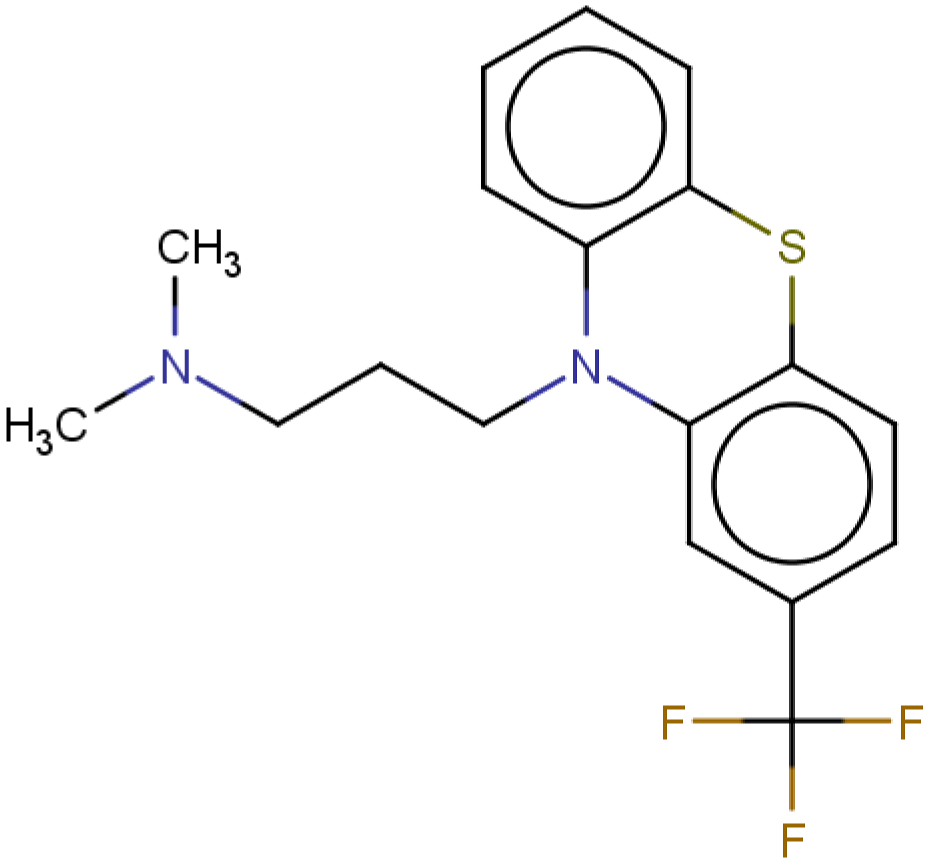

1098-60-8
Name: Triflupromazine hydrochloride
pIC50: 5.42
Rank: 144
Classes: No defined

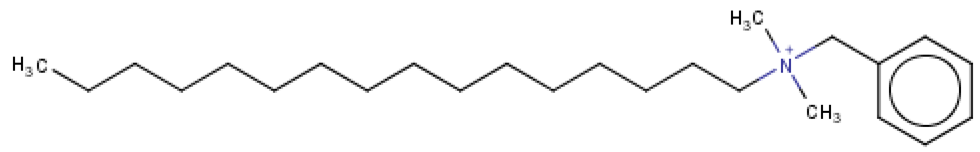

122-18-9
Name: Benzylhexadecyldimethylammonium chloride
pIC50: 5.4
Rank: 145
Classes: antimicrobial--TSCA

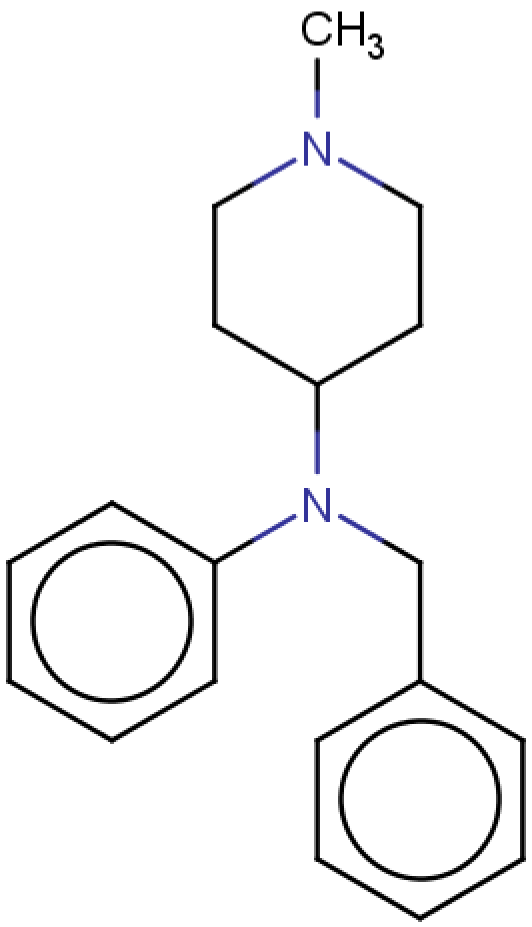

4945-47-5
Name: Bamipine
pIC50: 5.4
Rank: 146
Classes: No defined

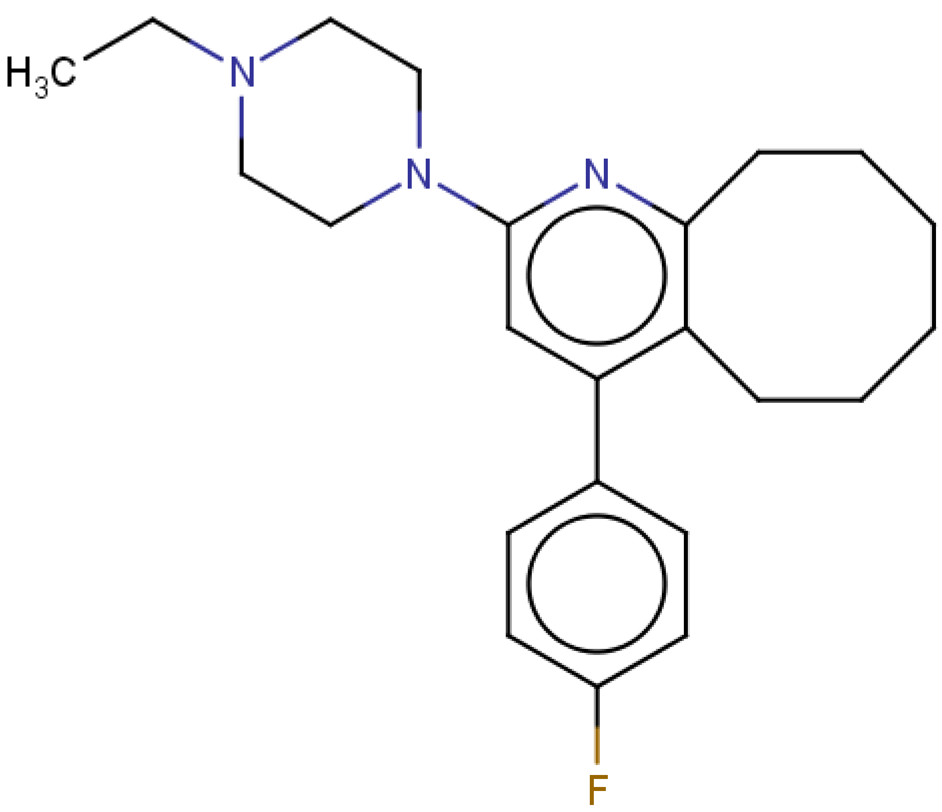

132810-10-7
Name: Blonanserin
pIC50: 5.4
Rank: 147
Classes: Drug

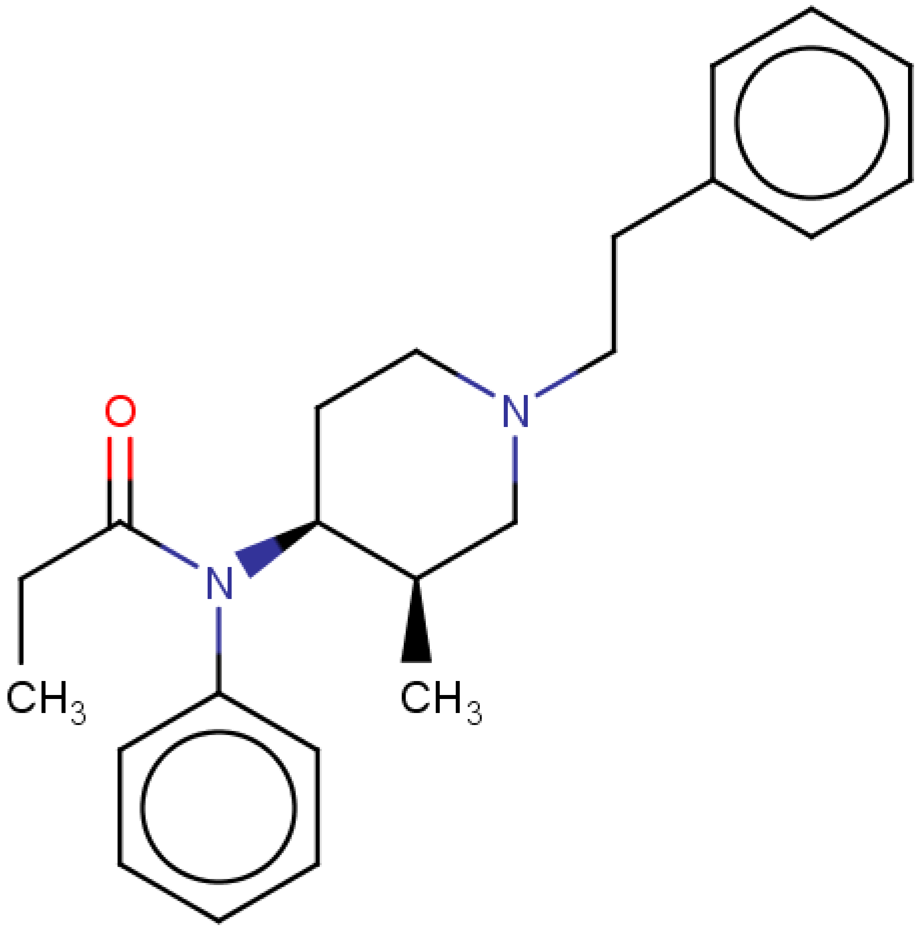

78995-18-3
Name: Dl-cis-3-Methylfentanyl hydrochloride
pIC50: 5.39
Rank: 148
Classes: No defined

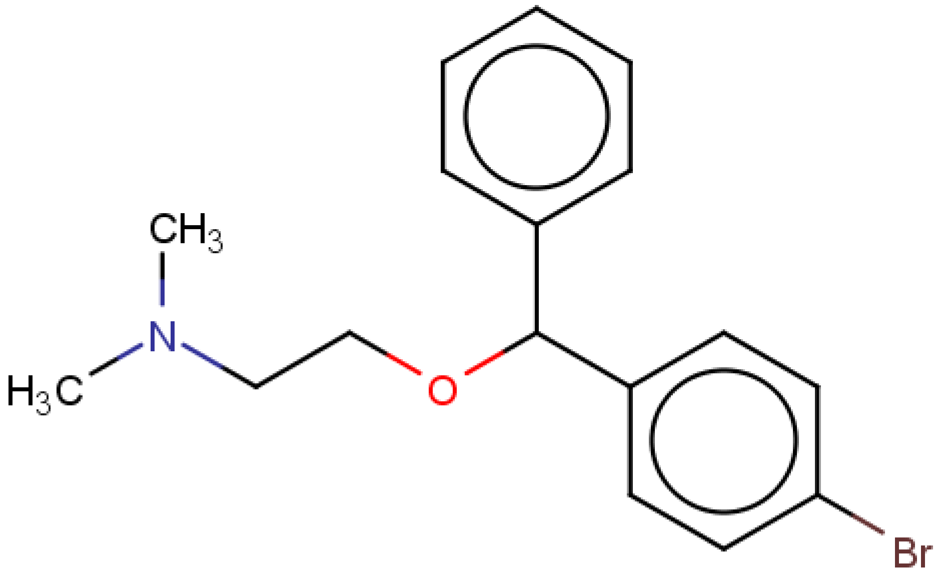

118-23-0
Name: Bromodiphenhydramine
pIC50: 5.39
Rank: 149
Classes: Drug

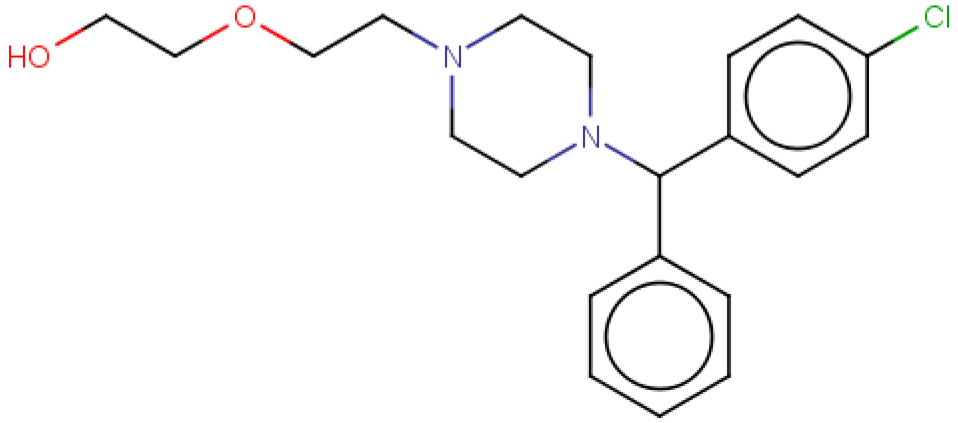

1244-76-4
Name: Hydroxyzine hydrochloride
pIC50: 5.38
Rank: 150
Classes: No defined

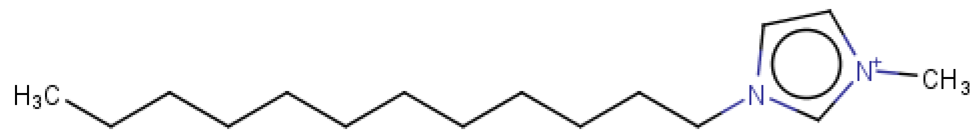

219947-93-0
Name: 1-Dodecyl-3-methylimidazolium hexafluorophosphate
pIC50: 5.38
Rank: 151
Classes: No defined

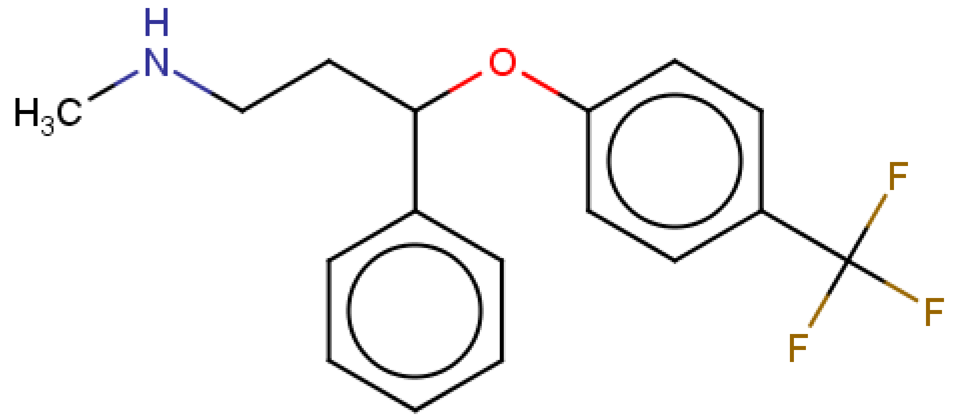

54910-89-3
Name: Fluoxetine
pIC50: 5.37
Rank: 152
Classes: Drug

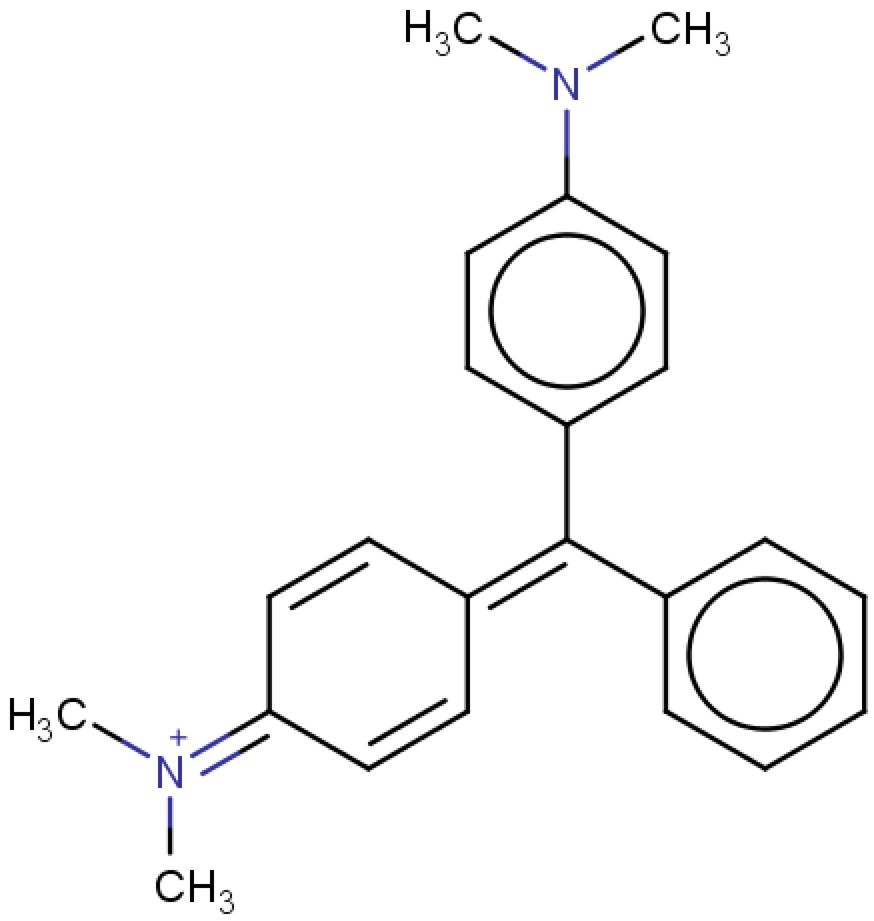

569-64-2
Name: Malachite green
pIC50: 5.37
Rank: 153
Classes: TSCA

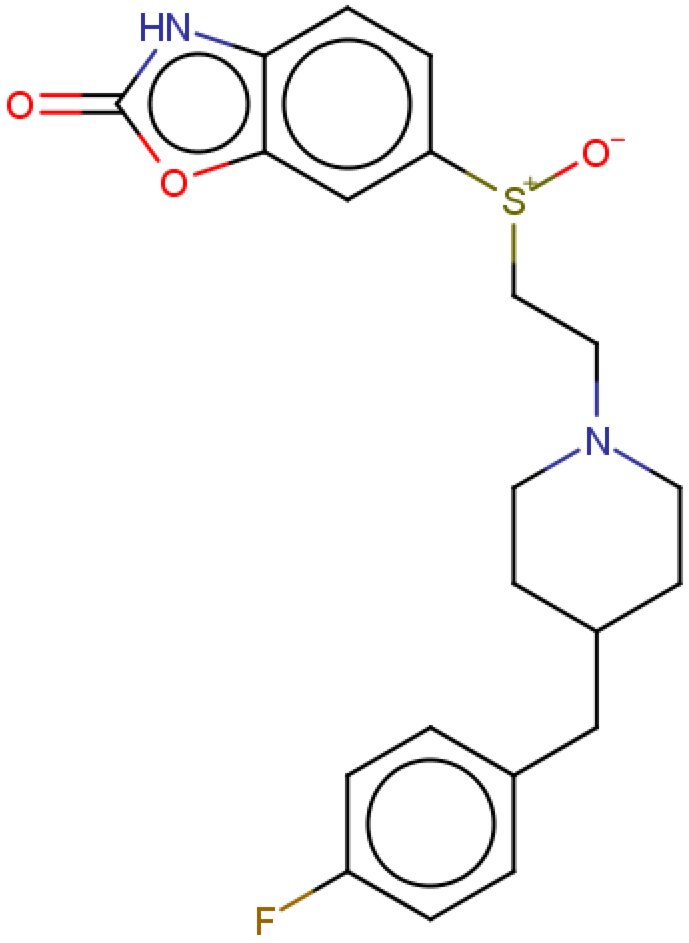

253450-09-8
Name: Besonprodil
pIC50: 5.36
Rank: 154
Classes: No defined

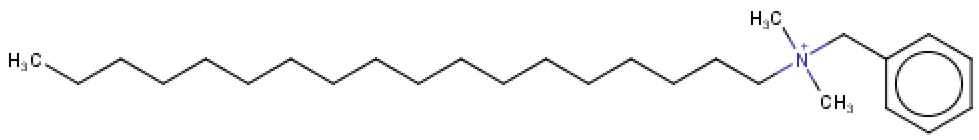

122-19-0
Name: N N-Dimethyl-N-benzyl-N-octadecylammonium chloride
pIC50: 5.36
Rank: 155
Classes: surfactant--TSCA

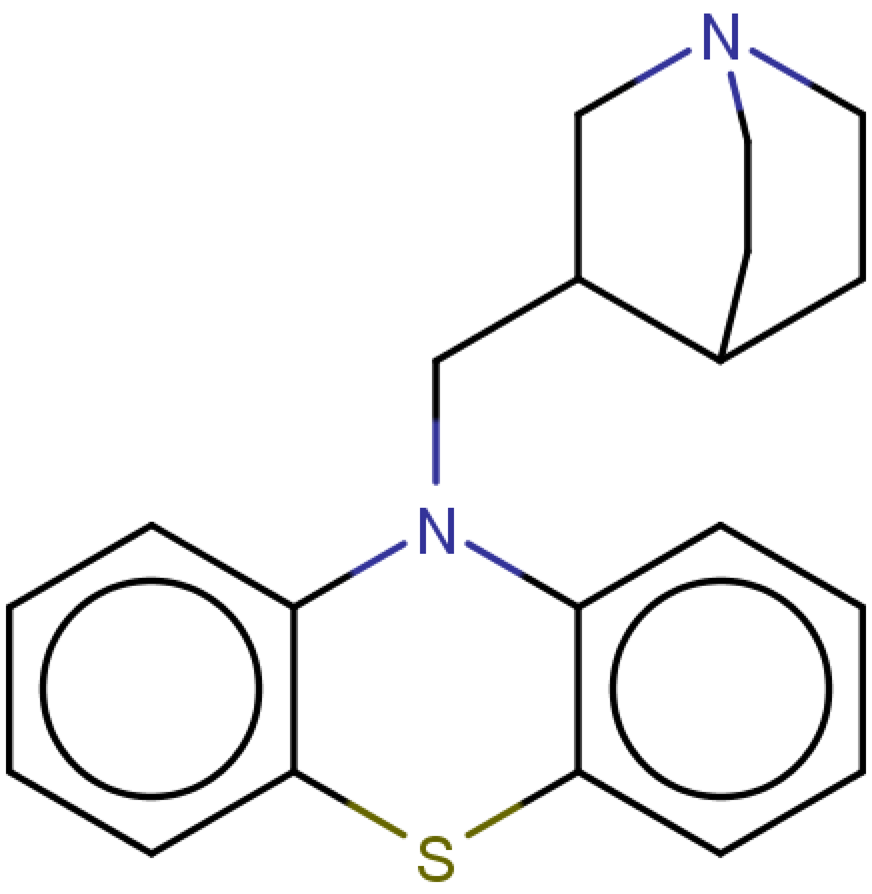

29216-28-2
Name: Mequitazine
pIC50: 5.35
Rank: 156
Classes: Drug

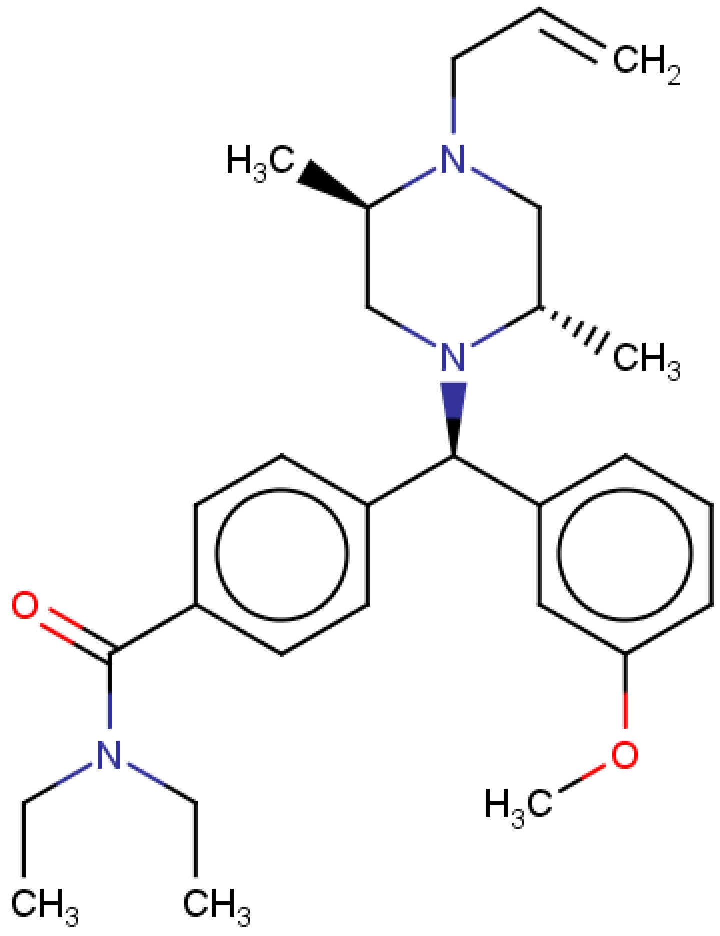

156727-74-1
Name: SNC 80
pIC50: 5.35
Rank: 157
Classes: No defined

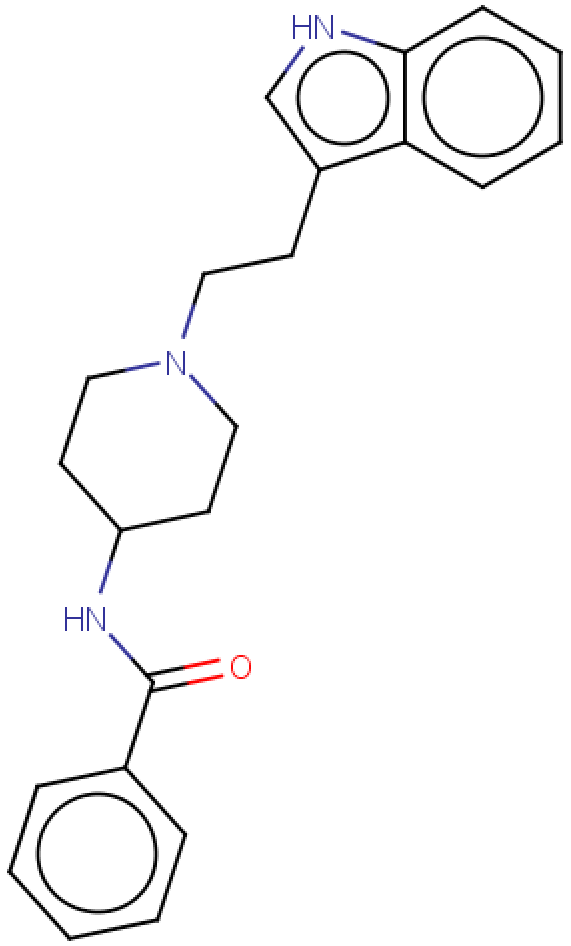

38821-52-2
Name: Indoramin hydrochloride
pIC50: 5.35
Rank: 158
Classes: No defined

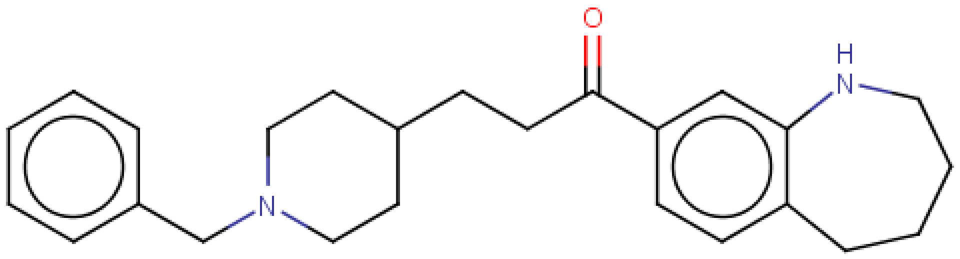

263248-42-6
Name: Zanapezil fumerate
pIC50: 5.34
Rank: 159
Classes: No defined

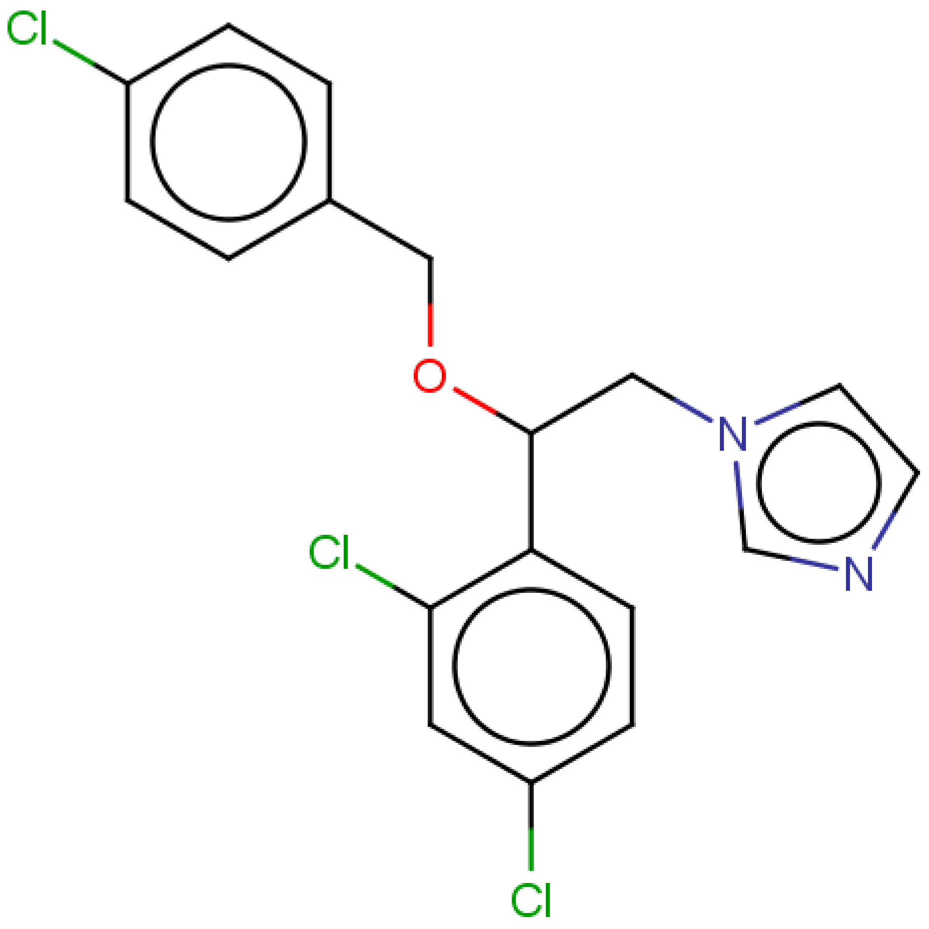

24169-02-6
Name: Econazole nitrate
pIC50: 5.32
Rank: 160
Classes: No defined

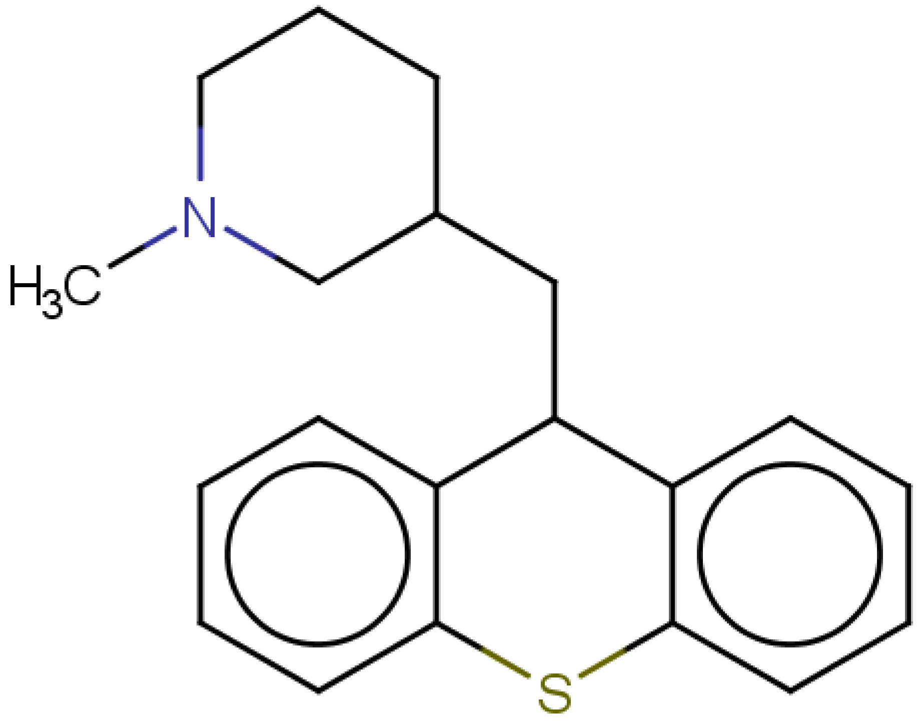

1553-34-0
Name: Metixene hydrochloride
pIC50: 5.32
Rank: 161
Classes: No defined

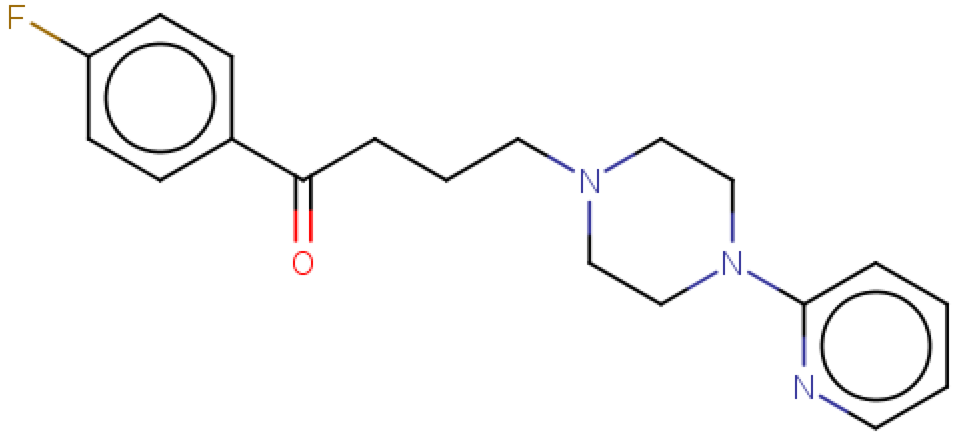

1649-18-9
Name: Azaperone
pIC50: 5.32
Rank: 162
Classes: No defined

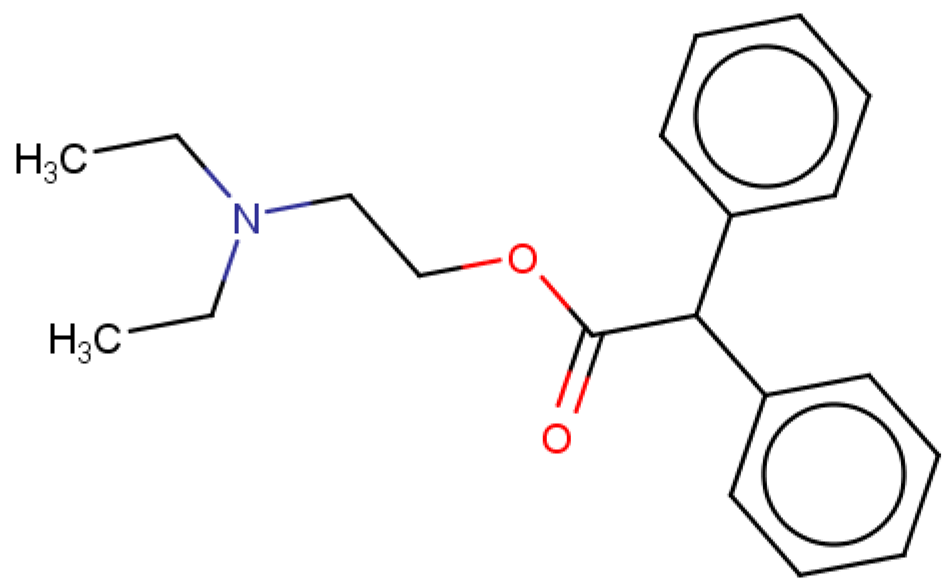

50-42-0
Name: Adiphenine hydrochloride
pIC50: 5.32
Rank: 163
Classes: No defined

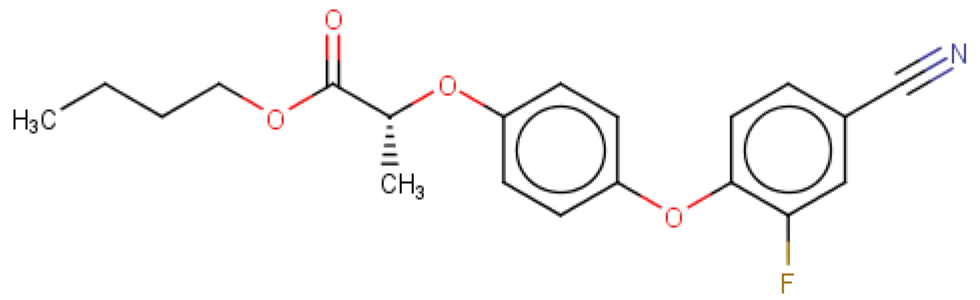

122008-85-9
Name: Cyhalofop-butyl
pIC50: 5.31
Rank: 164
Classes: Pesticide

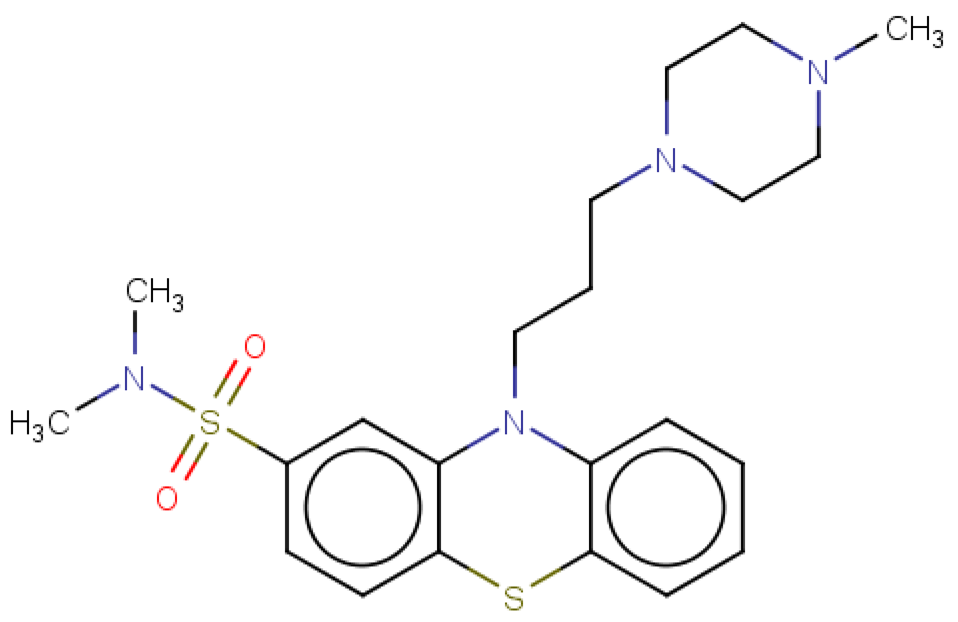

2347-80-0
Name: Thioproperazine dimethanesulfonate
pIC50: 5.3
Rank: 165
Classes: No defined

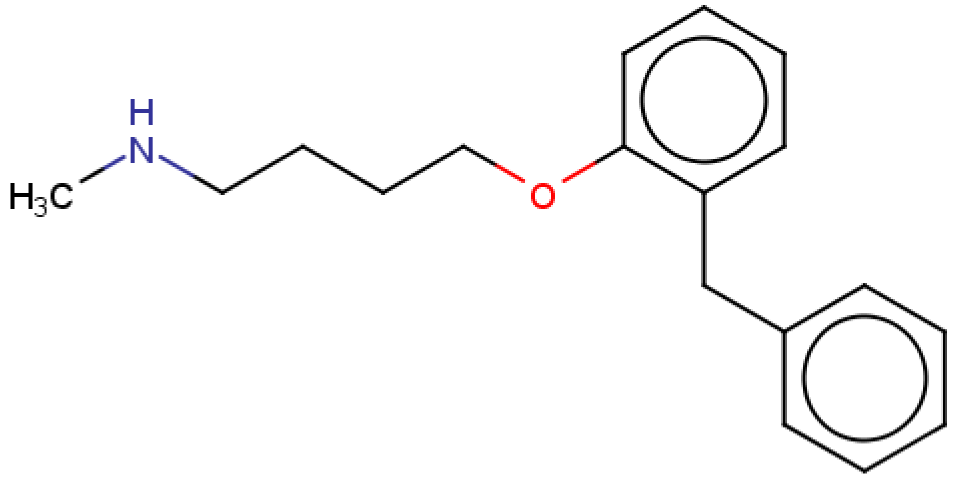

90293-01-9
Name: Bifemelane
pIC50: 5.3
Rank: 166
Classes: No defined

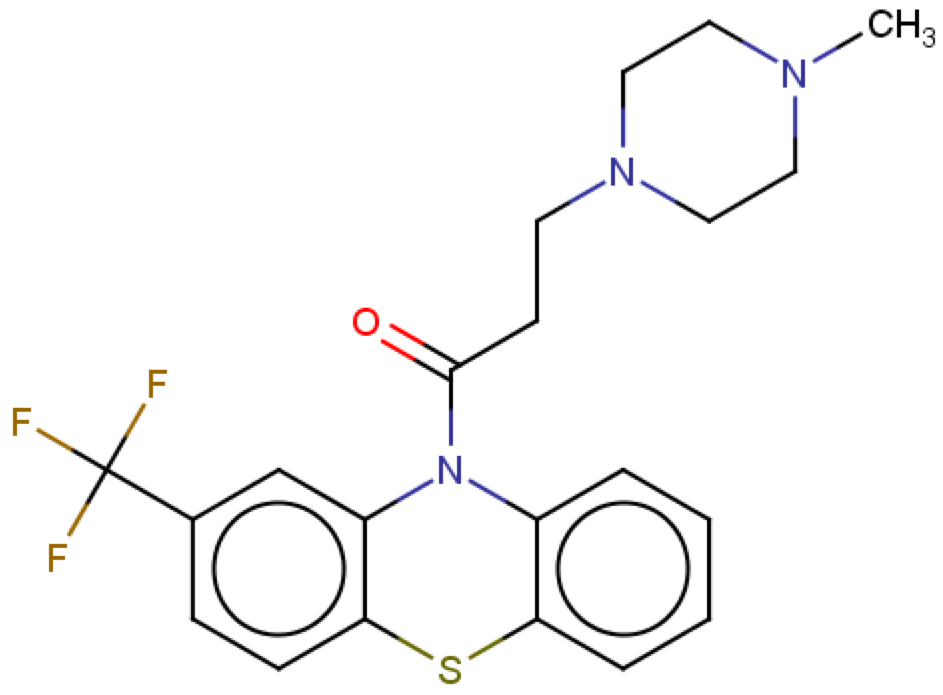

33414-30-1
Name: Ftormetazine
pIC50: 5.3
Rank: 167
Classes: No defined

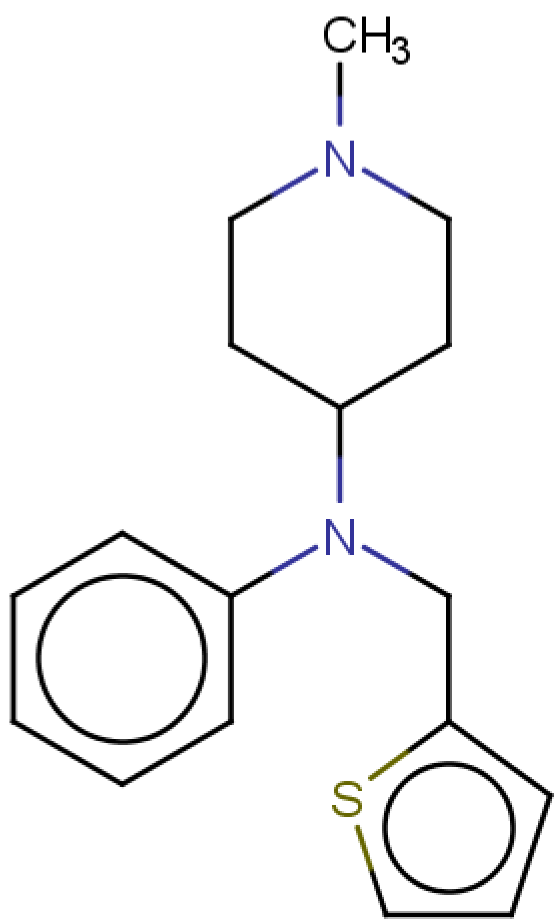

86-12-4
Name: Thenalidine
pIC50: 5.3
Rank: 168
Classes: Drug

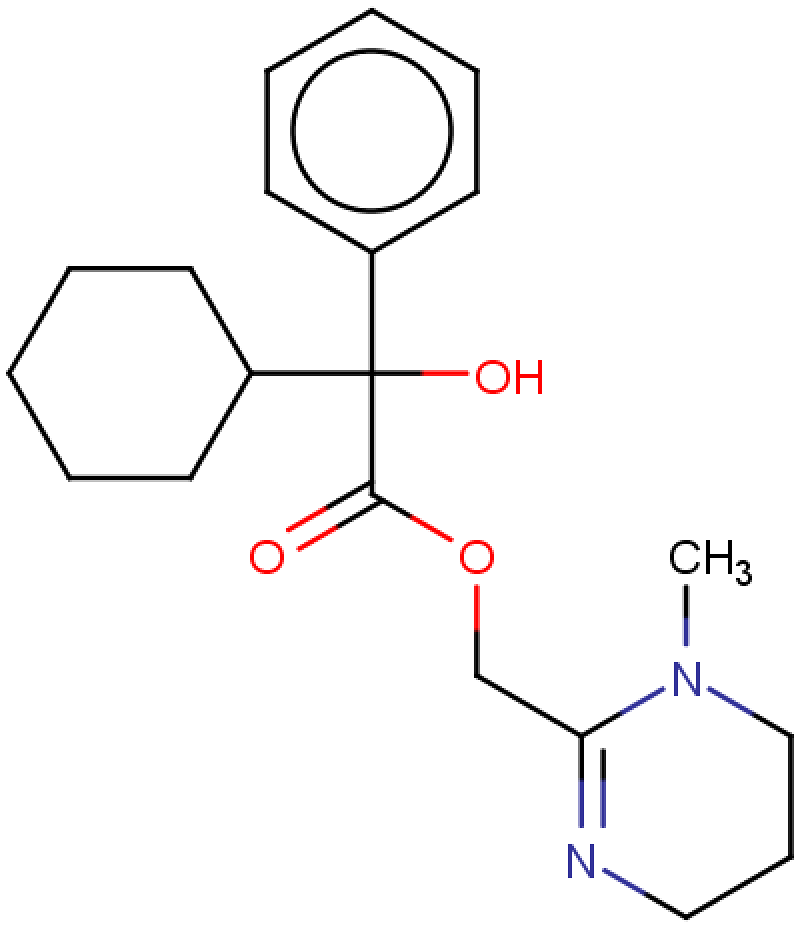

125-52-0
Name: Oxyphencyclimine hydrochloride
pIC50: 5.29
Rank: 169
Classes: No defined

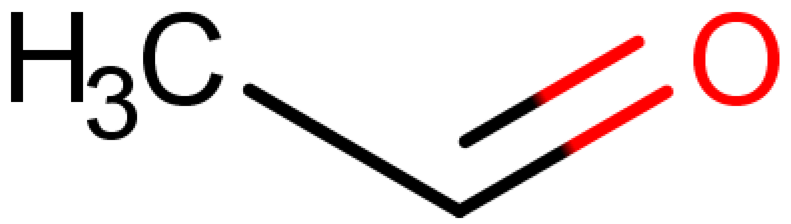

75-07-0
Name: Acetaldehyde
pIC50: 5.27
Rank: 170
Classes: colorant--TSCA

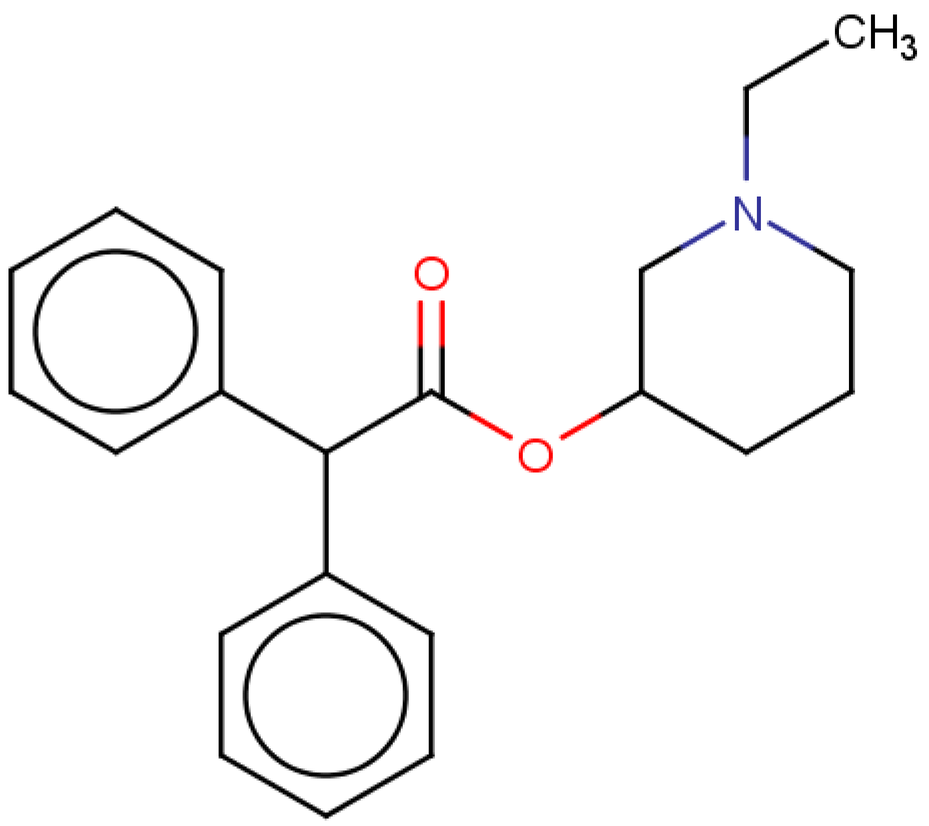

129-77-1
Name: Piperidolate hydrochloride
pIC50: 5.27
Rank: 171
Classes: No defined

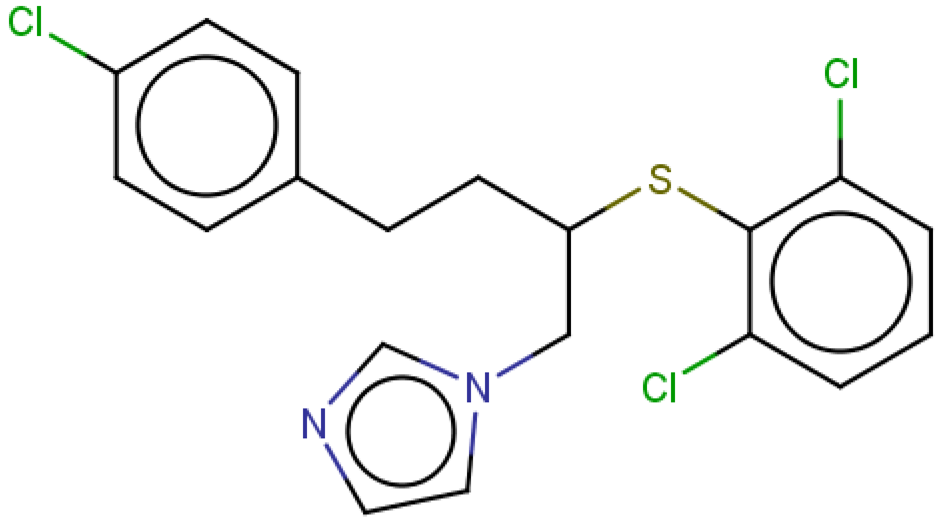

64872-77-1
Name: Butoconazole nitrate
pIC50: 5.27
Rank: 172
Classes: No defined

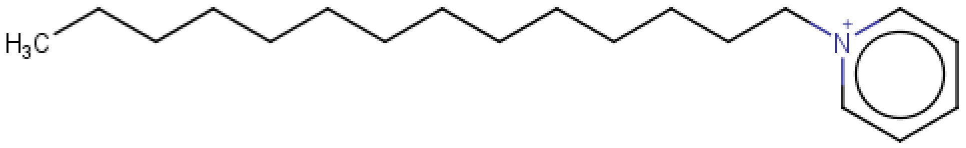

1155-74-4
Name: Tetradecylpyridinium bromide
pIC50: 5.26
Rank: 173
Classes: No defined

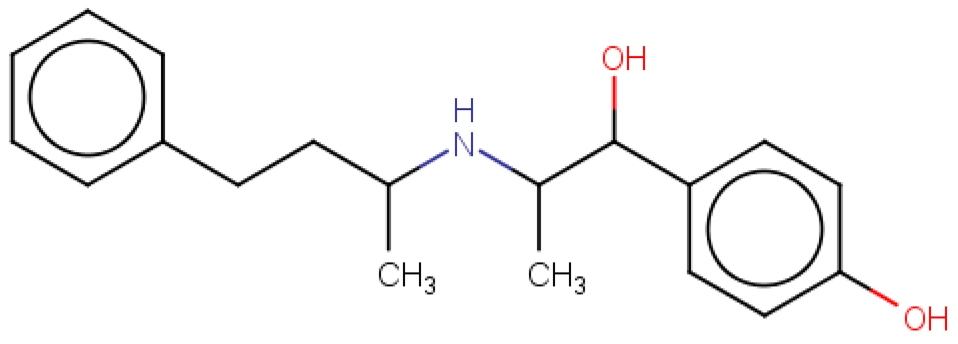

447-41-6
Name: Nylidrin
pIC50: 5.25
Rank: 174
Classes: No defined

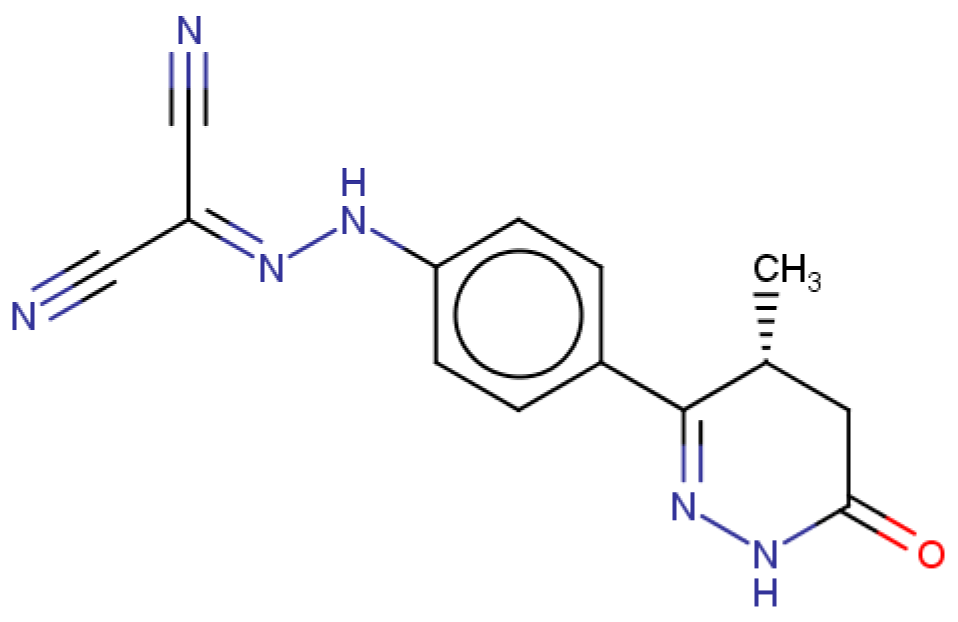

141505-33-1
Name: Levosimendan
pIC50: 5.25
Rank: 175
Classes: Drug

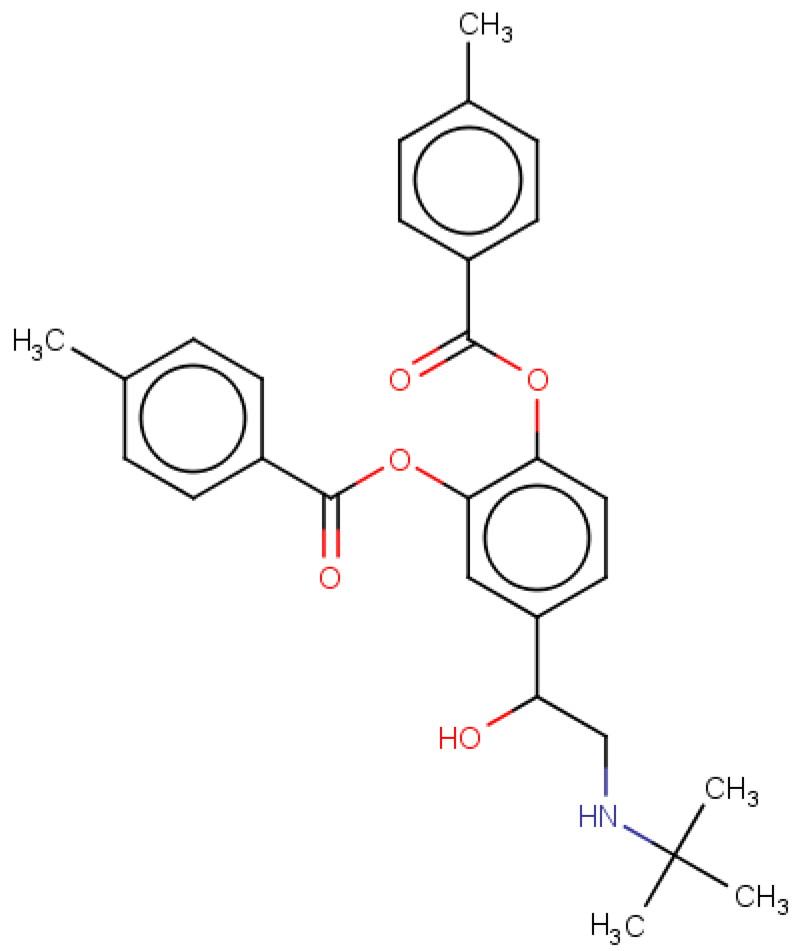

30392-40-6
Name: Bitolterol
pIC50: 5.24
Rank: 176
Classes: Drug

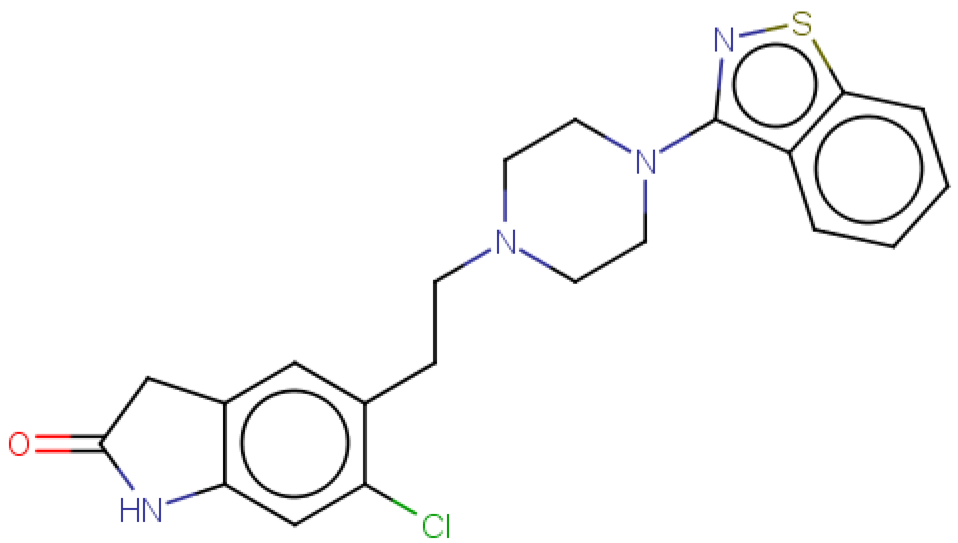

146939-27-7
Name: Ziprasidone
pIC50: 5.24
Rank: 177
Classes: Drug

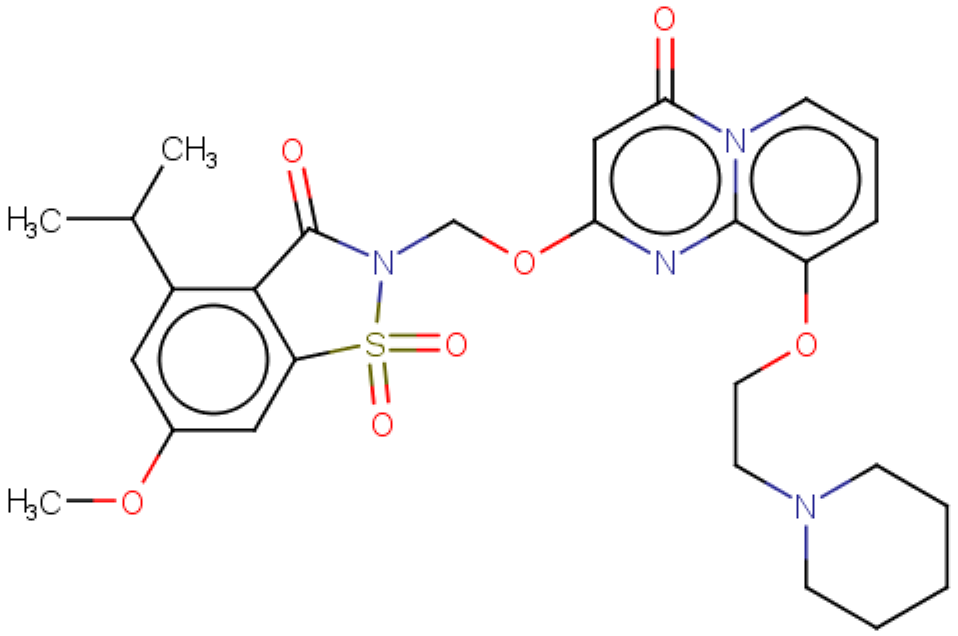

344930-95-6
Name: SSR69071
pIC50: 5.23
Rank: 178
Classes: No defined

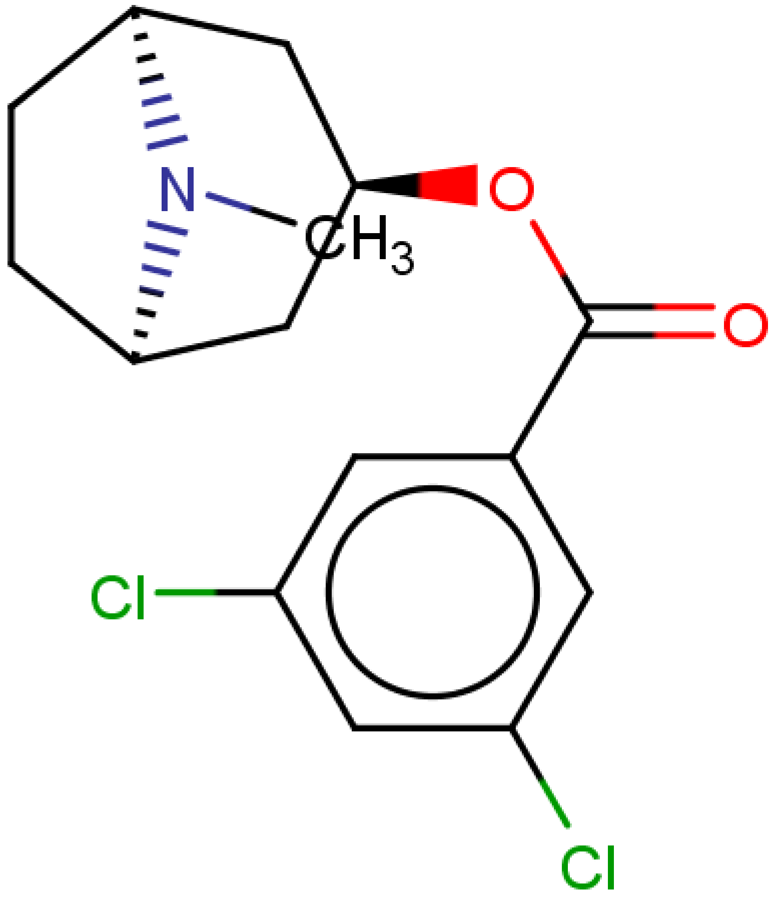

40796-97-2
Name: MDL 72222
pIC50: 5.22
Rank: 179
Classes: No defined

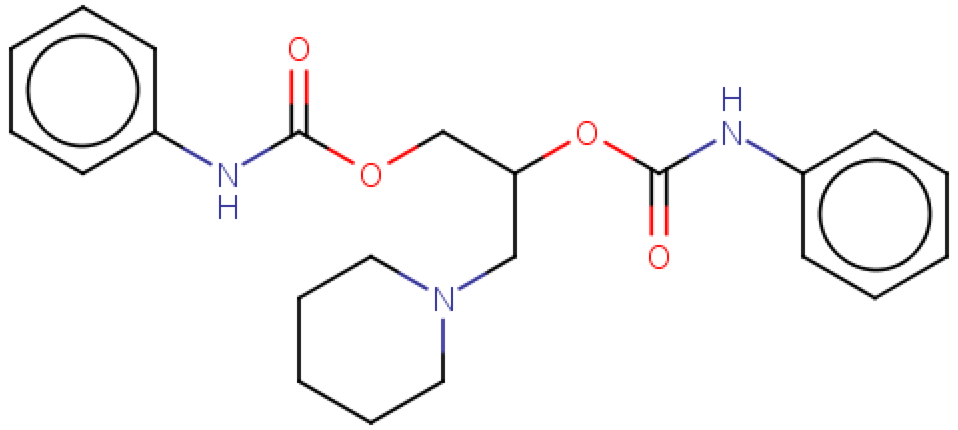

537-12-2
Name: Diperodon hydrochloride
pIC50: 5.22
Rank: 180
Classes: No defined

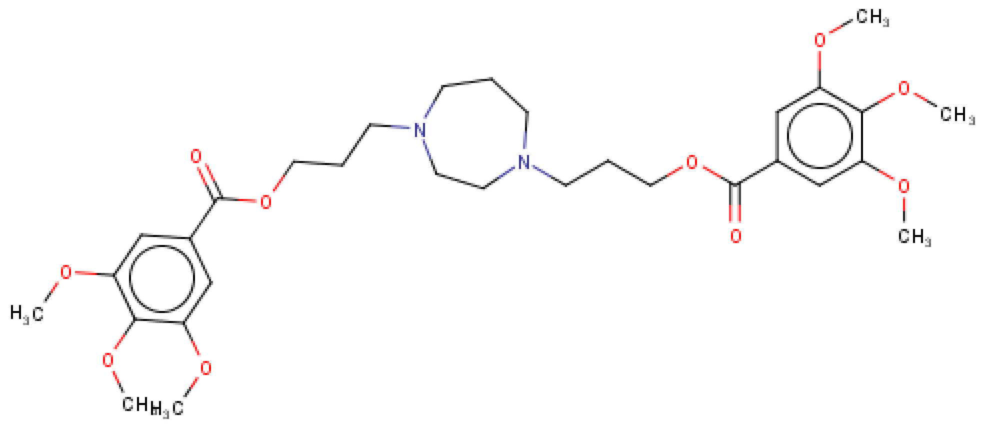

35898-87-4
Name: Dilazep
pIC50: 5.22
Rank: 181
Classes: No defined

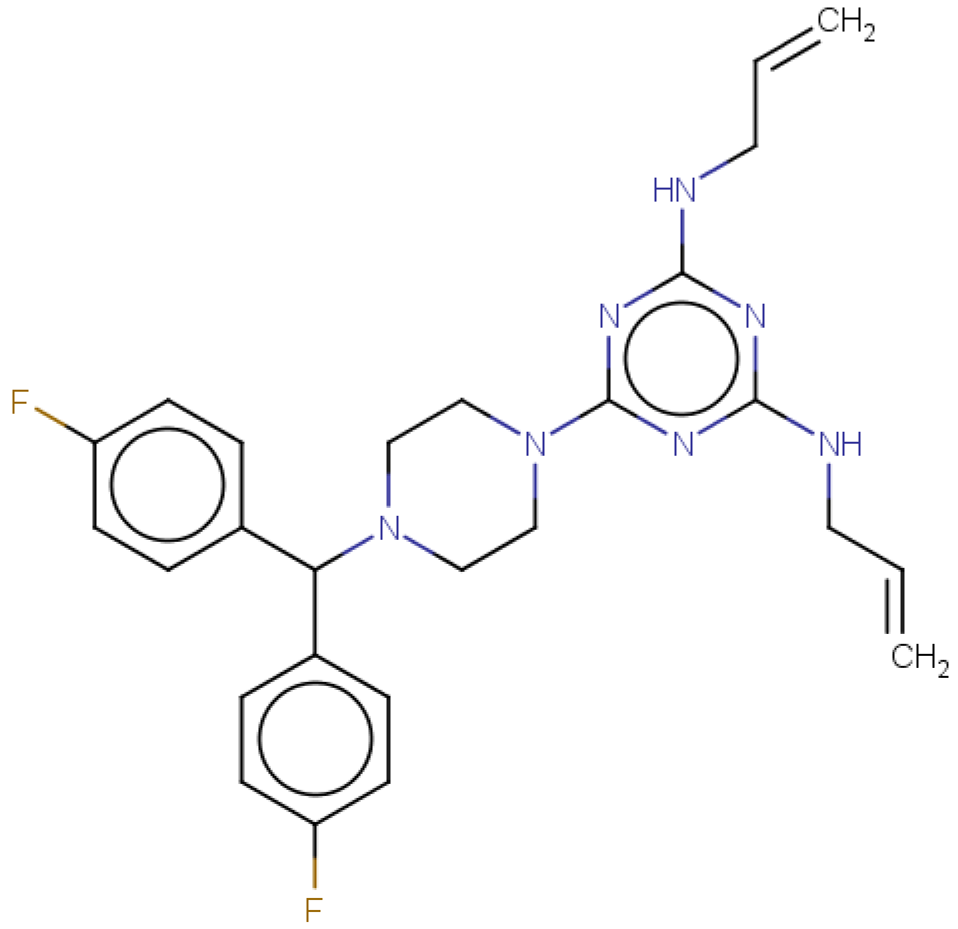

29608-49-9
Name: Almitrine dimethanesulfonate
pIC50: 5.22
Rank: 182
Classes: No defined

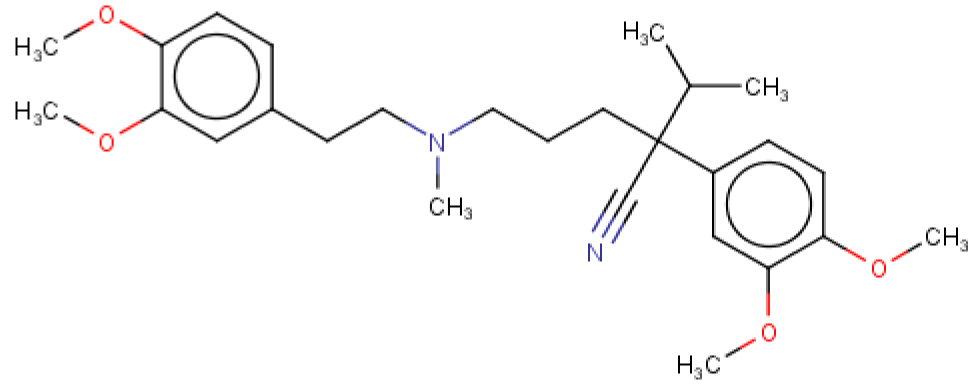

152-11-4
Name: Verapamil hydrochloride
pIC50: 5.21
Rank: 183
Classes: No defined

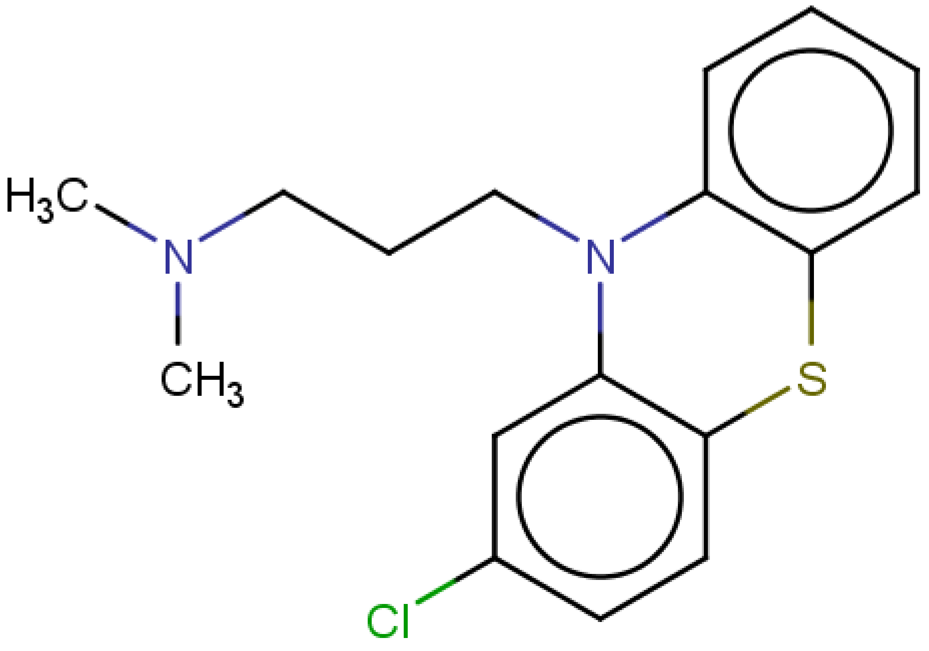

69-09-0
Name: Chlorpromazine hydrochloride
pIC50: 5.21
Rank: 184
Classes: TSCA

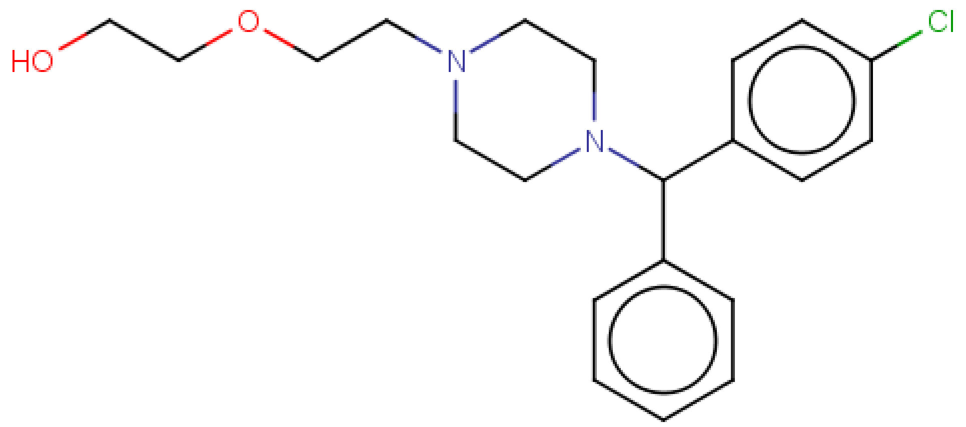

68-88-2
Name: Hydroxyzine
pIC50: 5.2
Rank: 185
Classes: Drug

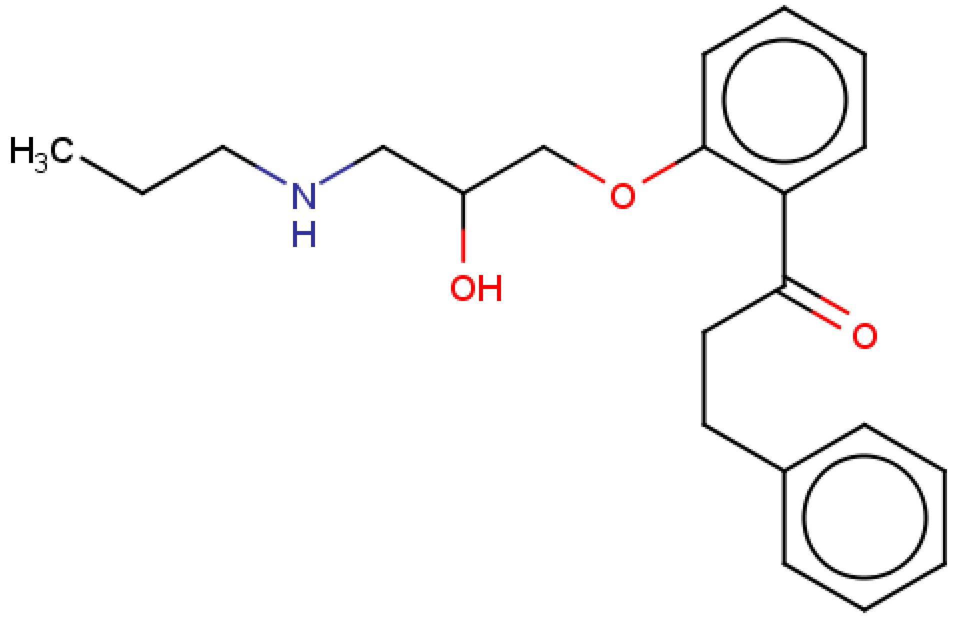

54063-53-5
Name: Propafenone
pIC50: 5.2
Rank: 186
Classes: Drug

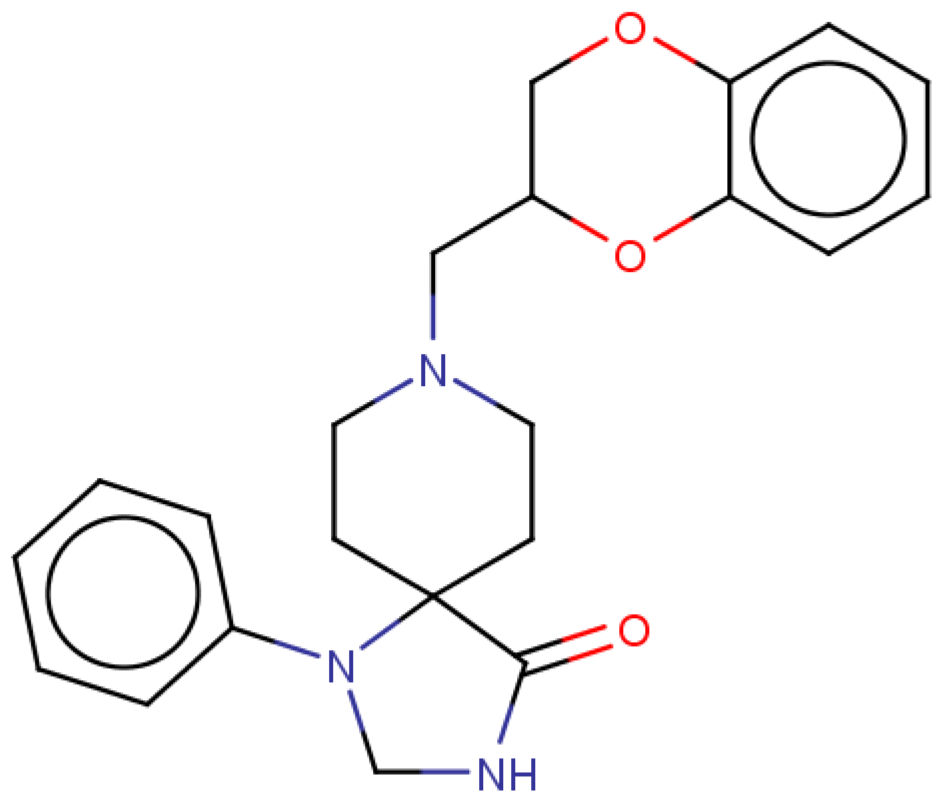

1054-88-2
Name: Spiroxatrine
pIC50: 5.2
Rank: 187
Classes: No defined

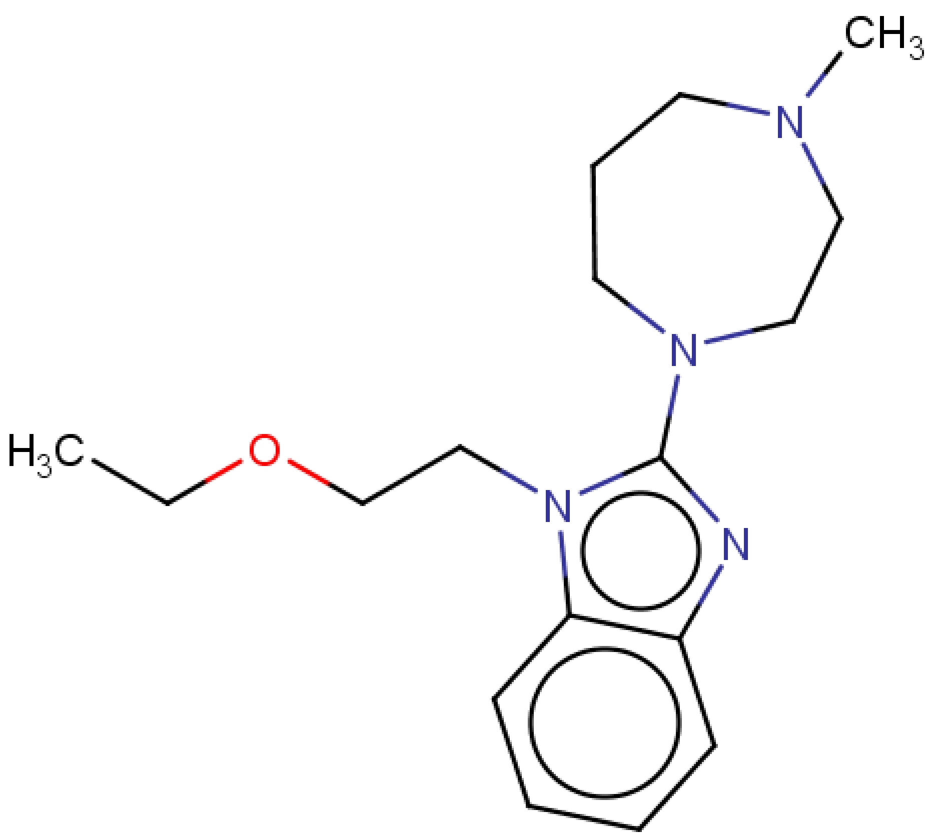

87233-62-3
Name: Emedastine difumarate
pIC50: 5.2
Rank: 188
Classes: No defined

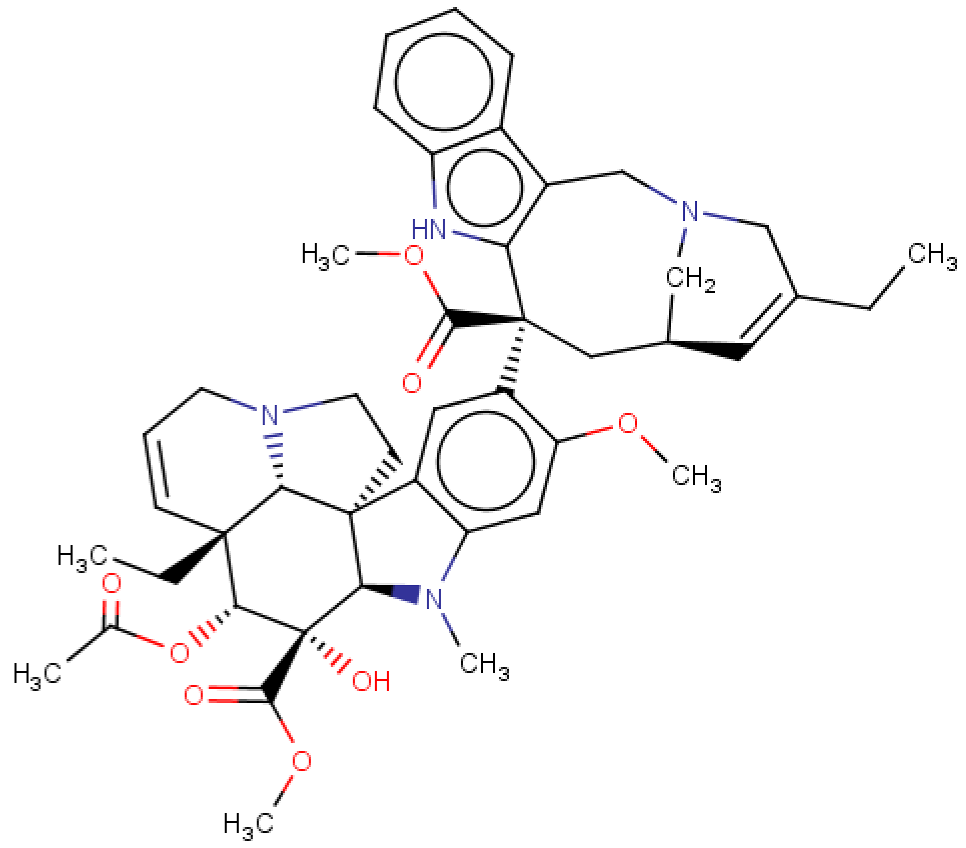

125317-39-7
Name: Vinorelbine tartrate
pIC50: 5.19
Rank: 189
Classes: No defined

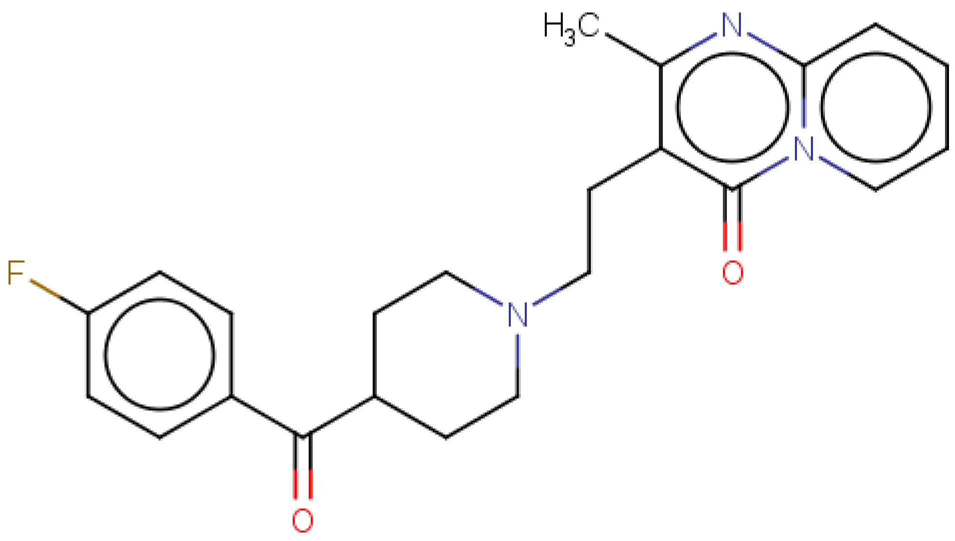

75444-65-4
Name: Pirenperone
pIC50: 5.17
Rank: 190
Classes: No defined

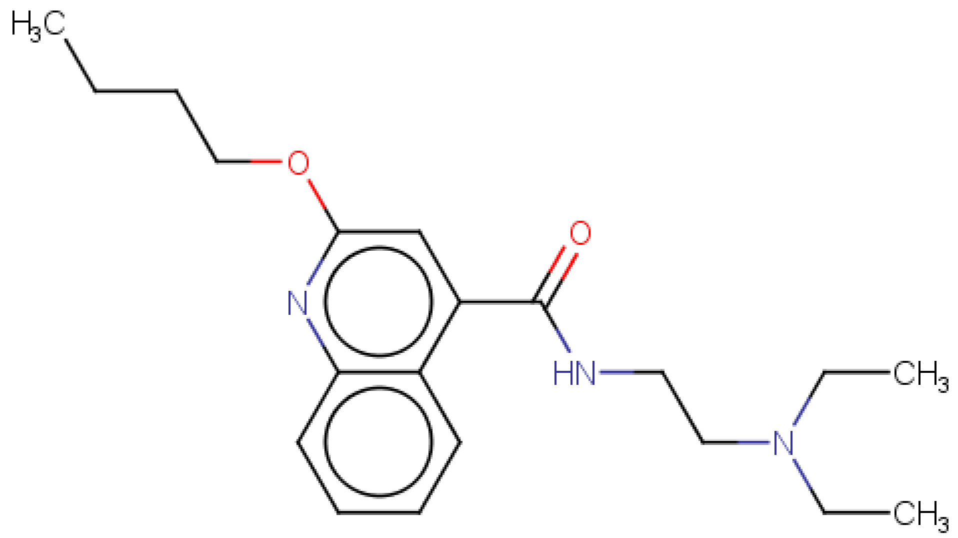

85-79-0
Name: Dibucaine
pIC50: 5.17
Rank: 191
Classes: Drug

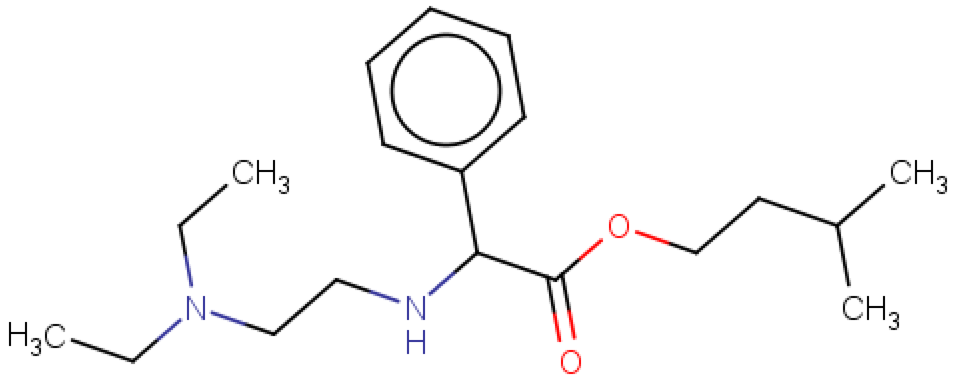

54-30-8
Name: Camylofin
pIC50: 5.17
Rank: 192
Classes: No defined

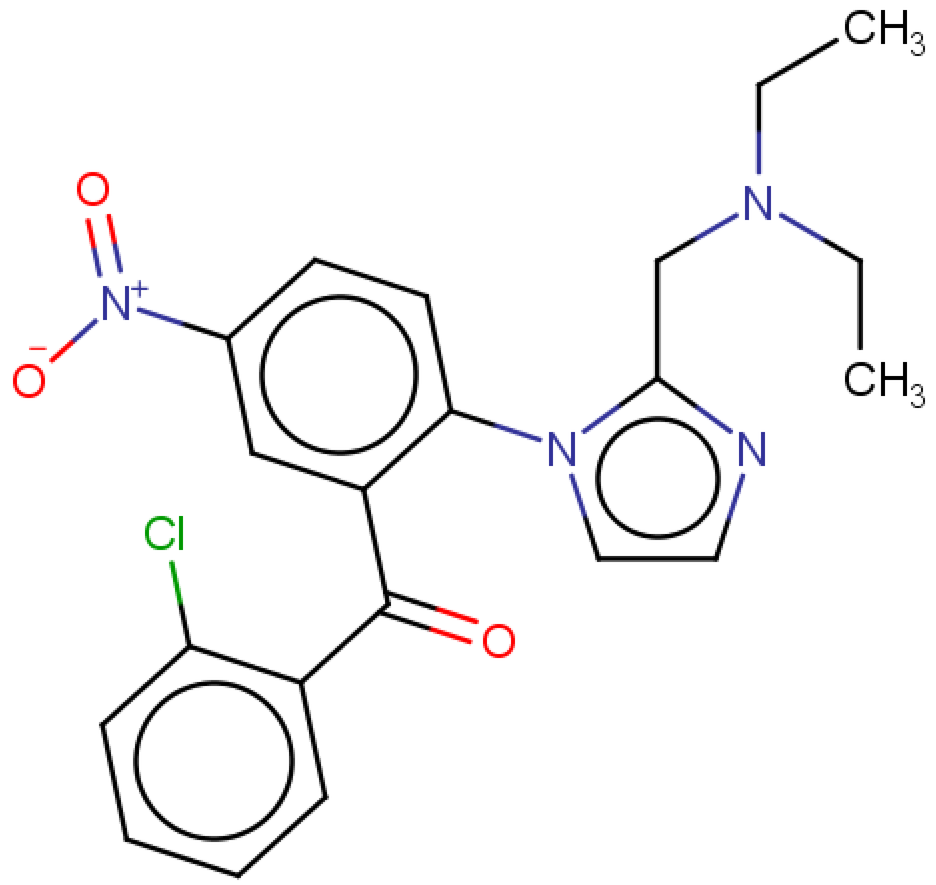

54533-86-7
Name: Nizofenone
pIC50: 5.17
Rank: 193
Classes: No defined

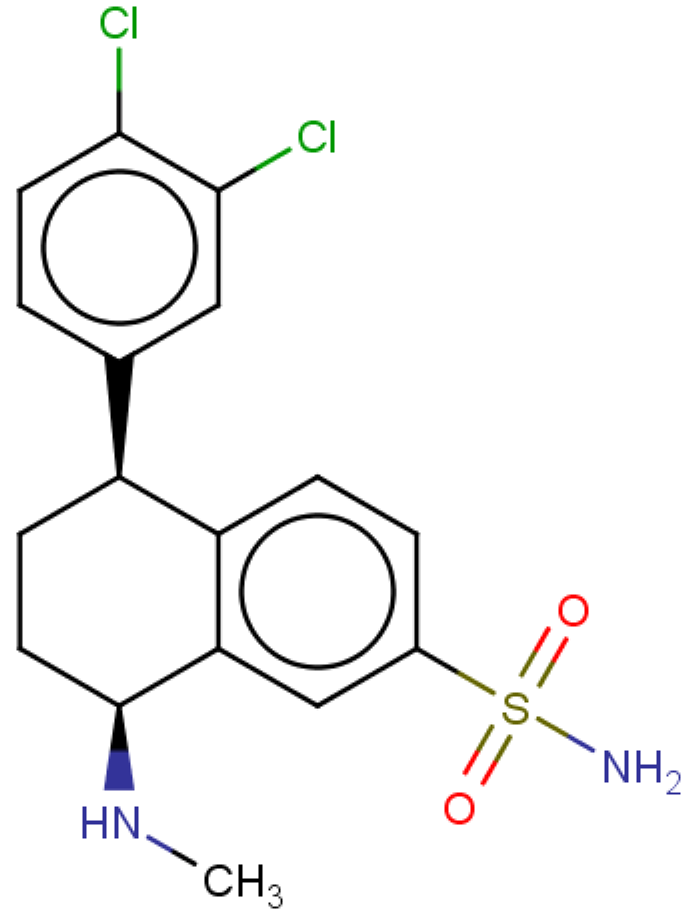

291305-06-1
Name: UK-373911
pIC50: 5.16
Rank: 194
Classes: No defined

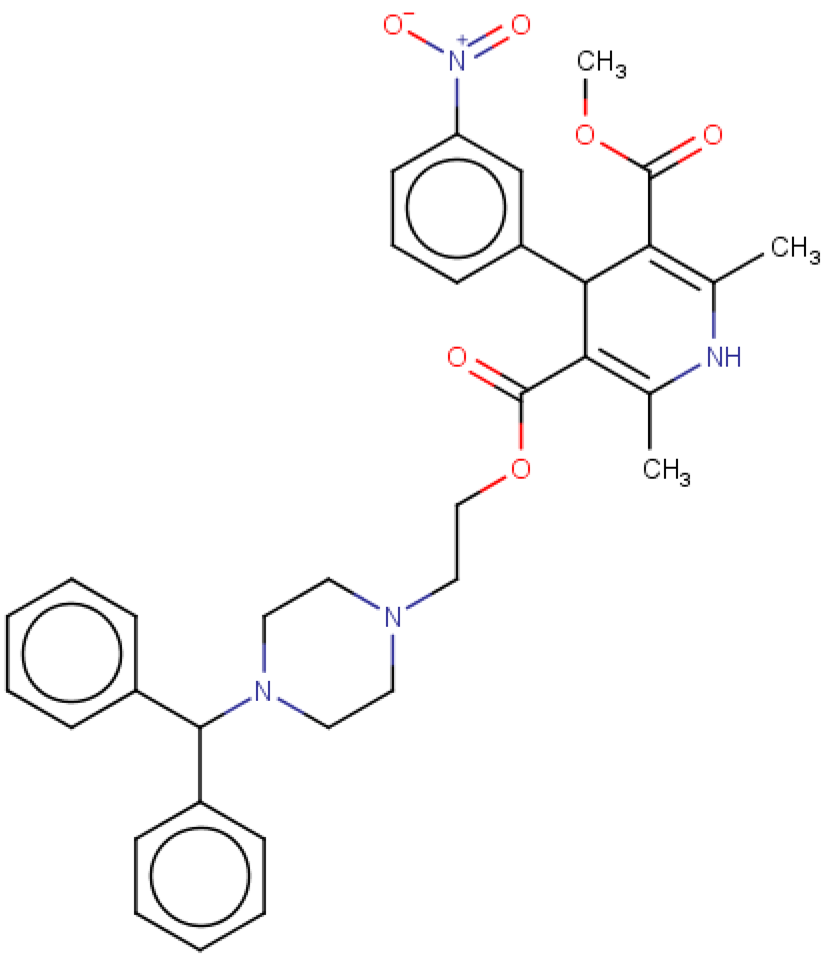

89226-75-5
Name: Manidipine dihydrochloride
pIC50: 5.15
Rank: 195
Classes: No defined

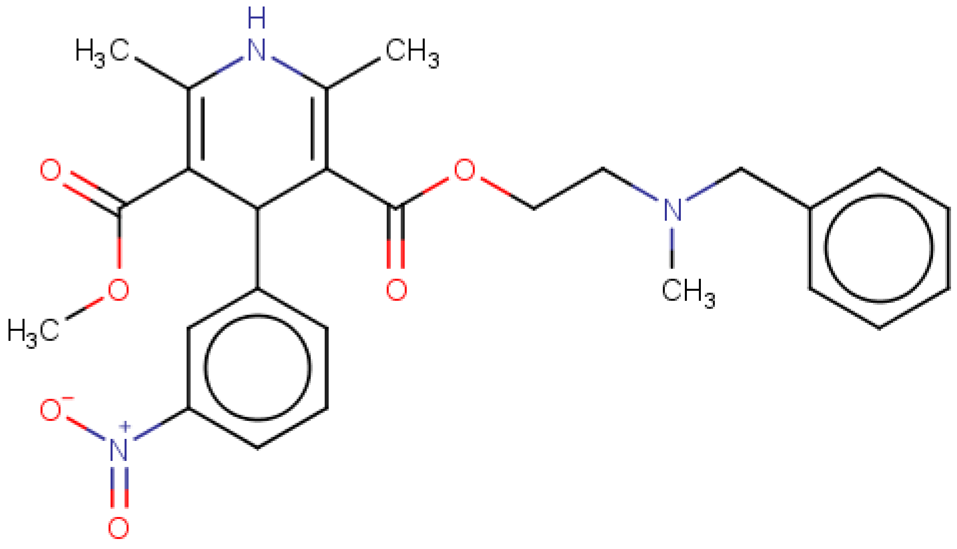

55985-32-5
Name: Nicardipine
pIC50: 5.15
Rank: 196
Classes: Drug

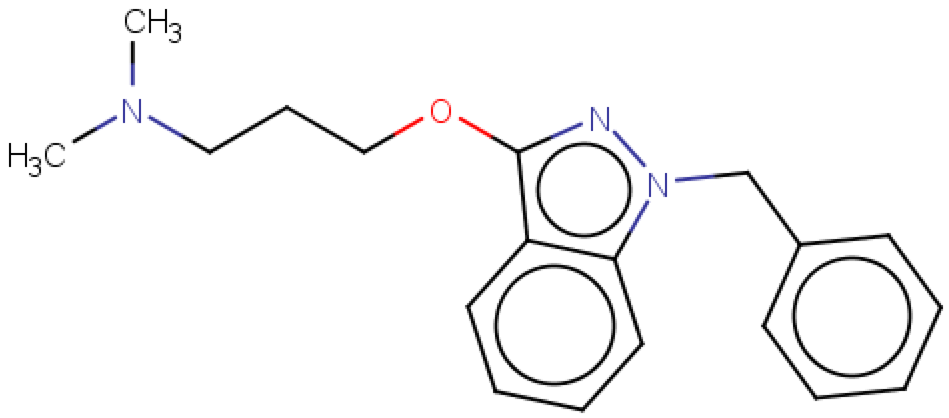

132-69-4
Name: Benzydamine hydrochloride
pIC50: 5.15
Rank: 197
Classes: No defined

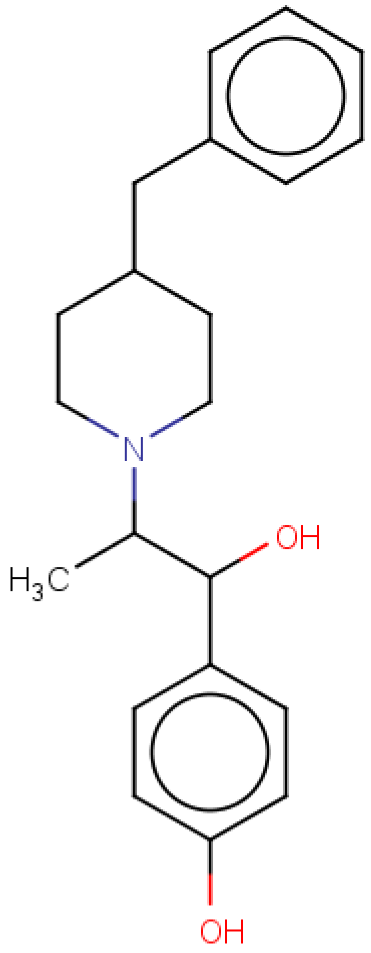

23210-56-2
Name: Ifenprodil
pIC50: 5.15
Rank: 198
Classes: Drug

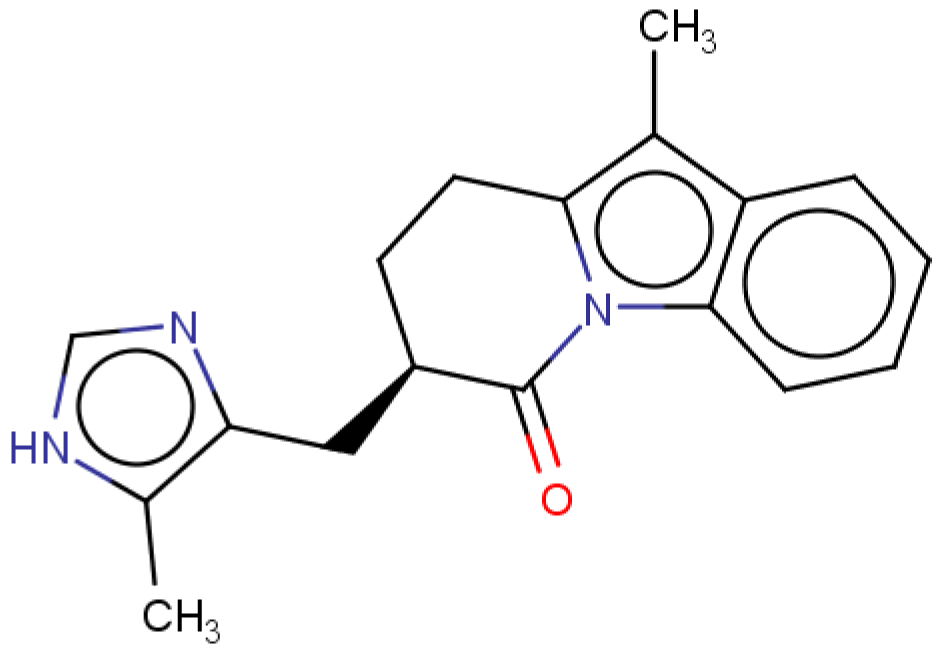

129299-90-7
Name: Fabesetron hydrochloride
pIC50: 5.15
Rank: 199
Classes: No defined

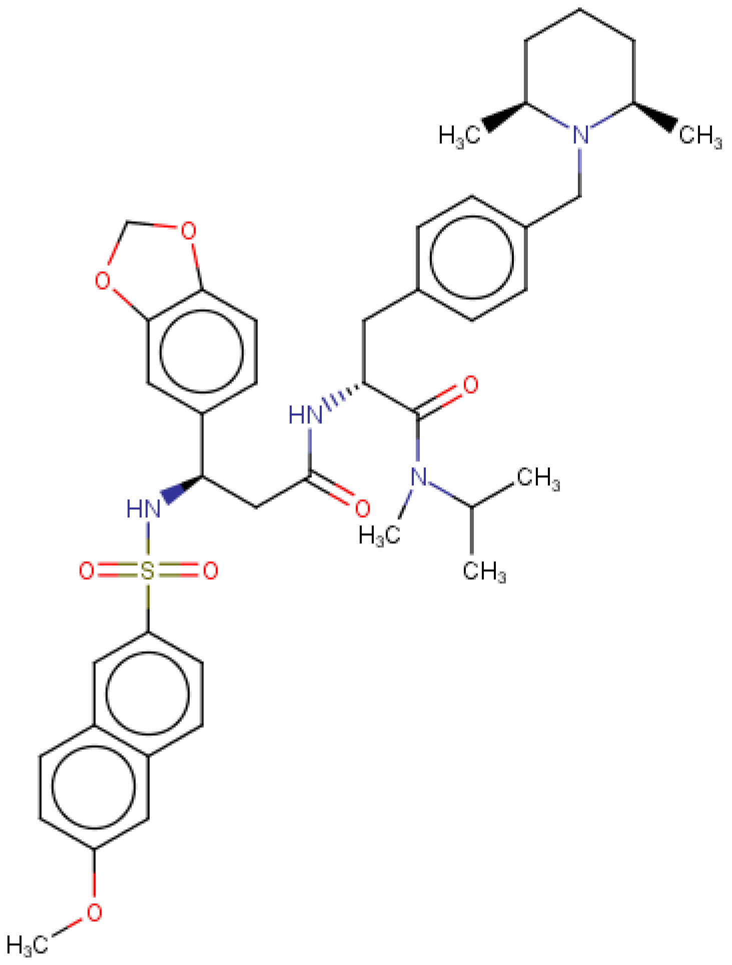

464930-42-5
Name: SSR 240612
pIC50: 5.15
Rank: 200
Classes: No defined

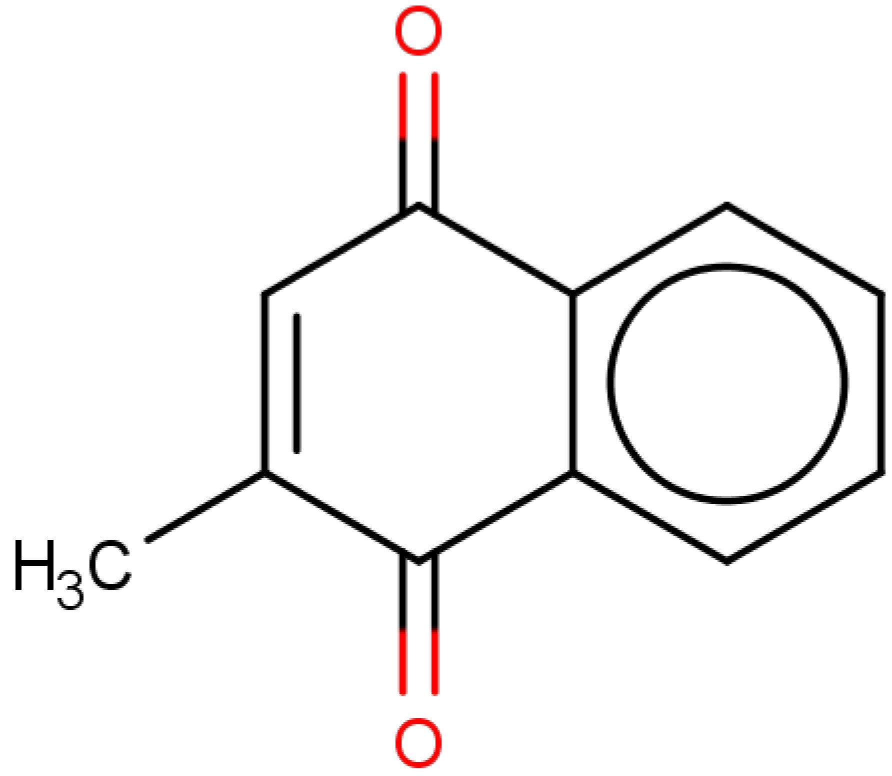

58-27-5
Name: Menadione
pIC50: 5.14
Rank: 201
Classes: masking agent--Drug--TSCA

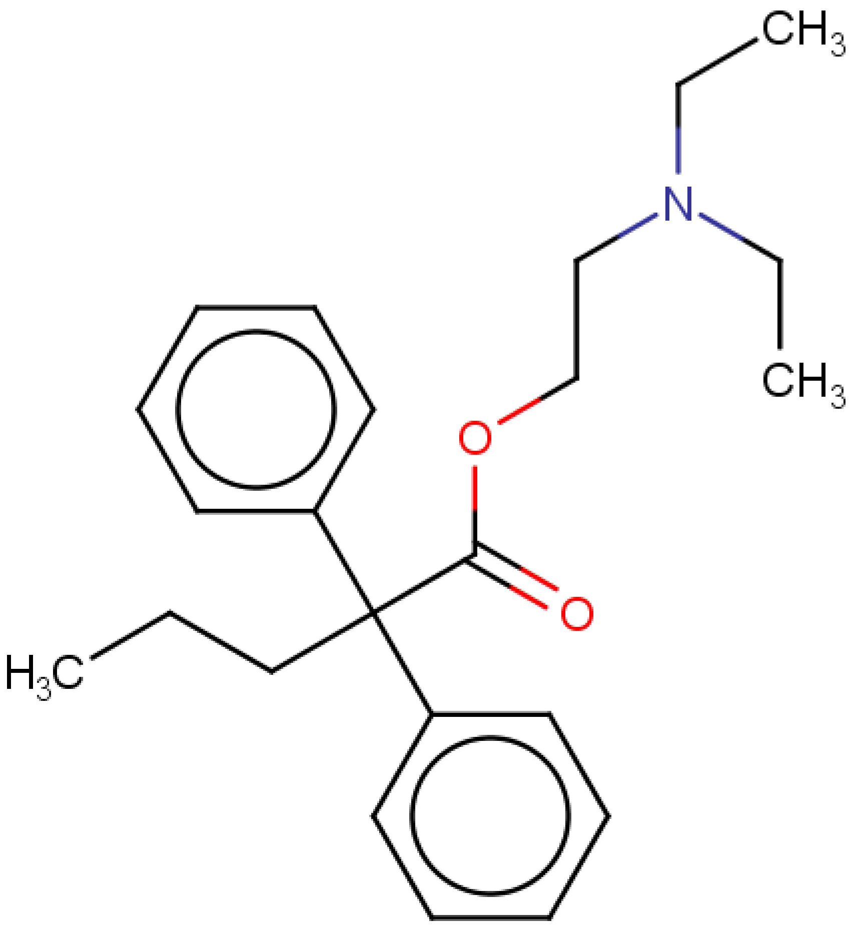

62-68-0
Name: Proadifen hydrochloride
pIC50: 5.14
Rank: 202
Classes: No defined

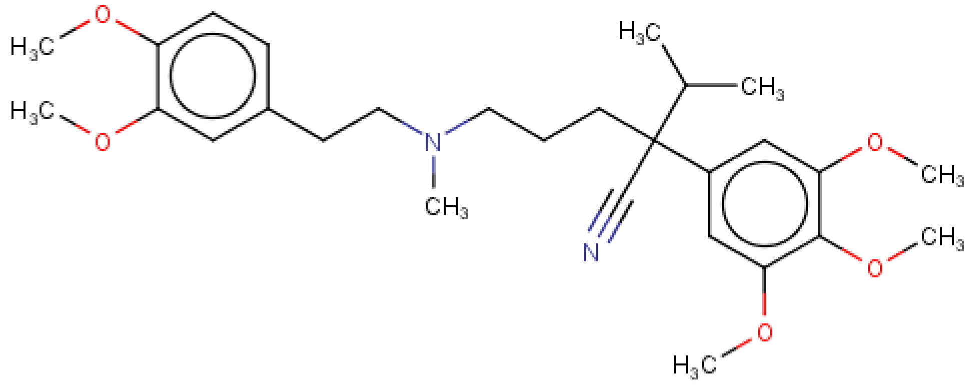

16662-46-7
Name: Gallopamil hydrochloride
pIC50: 5.14
Rank: 203
Classes: No defined

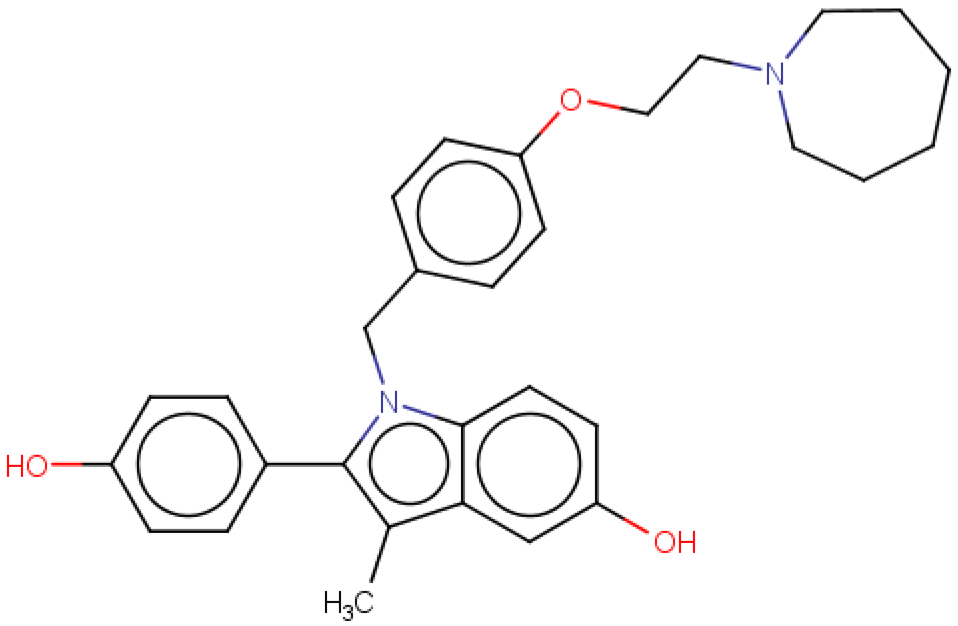

198481-33-3
Name: Bazedoxifene acetate
pIC50: 5.14
Rank: 204
Classes: No defined

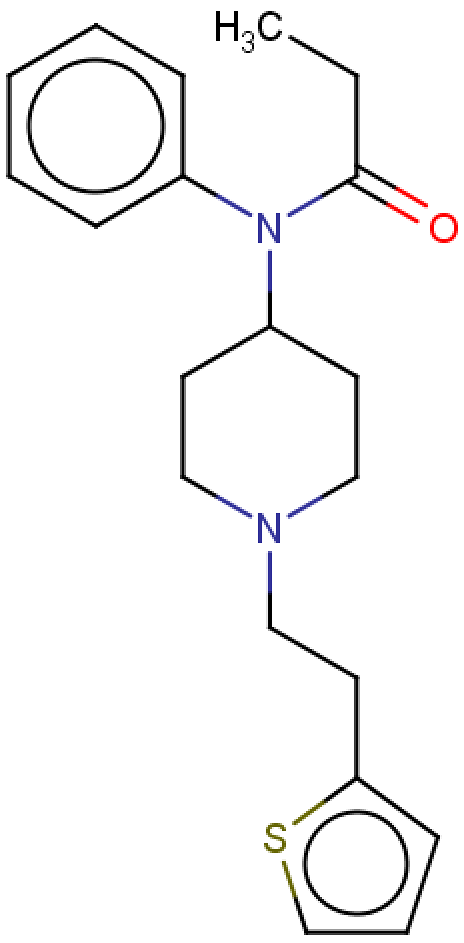

79278-88-9
Name: N-Phenyl-N-[1-[2-(2-thienyl)ethyl]-4-piperidinyl]propanamide hydrochloride (1:1)
pIC50: 5.12
Rank: 205
Classes: No defined

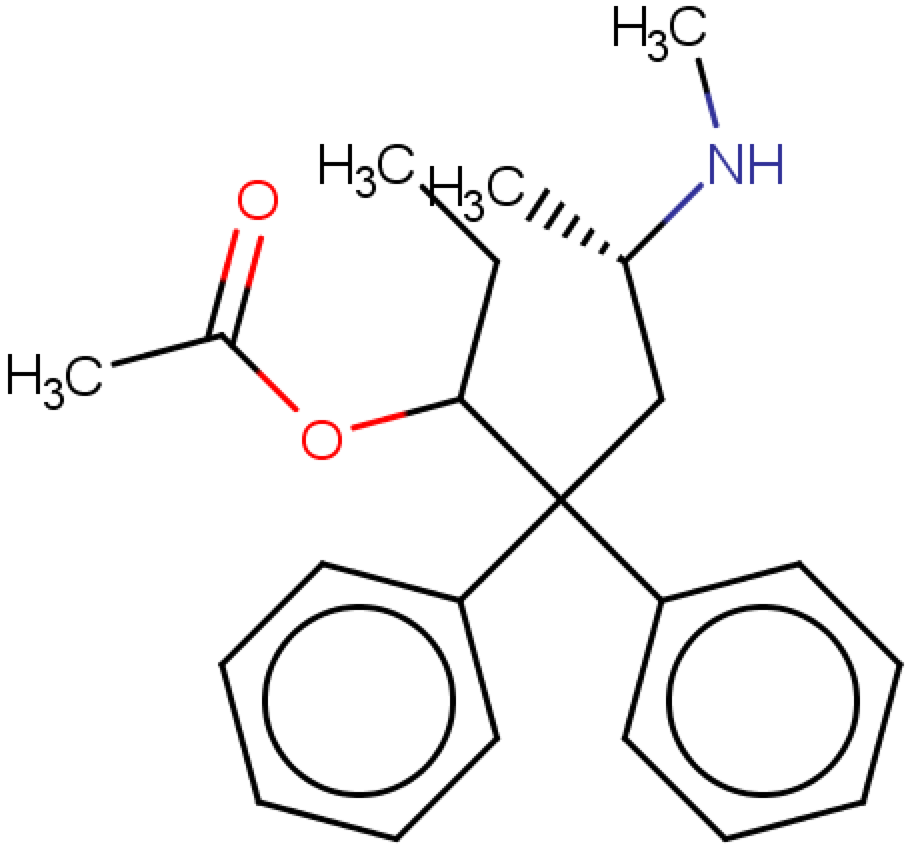

55096-75-8
Name: L-alpha-Noracetylmethadol hydrochloride
pIC50: 5.12
Rank: 206
Classes: No defined

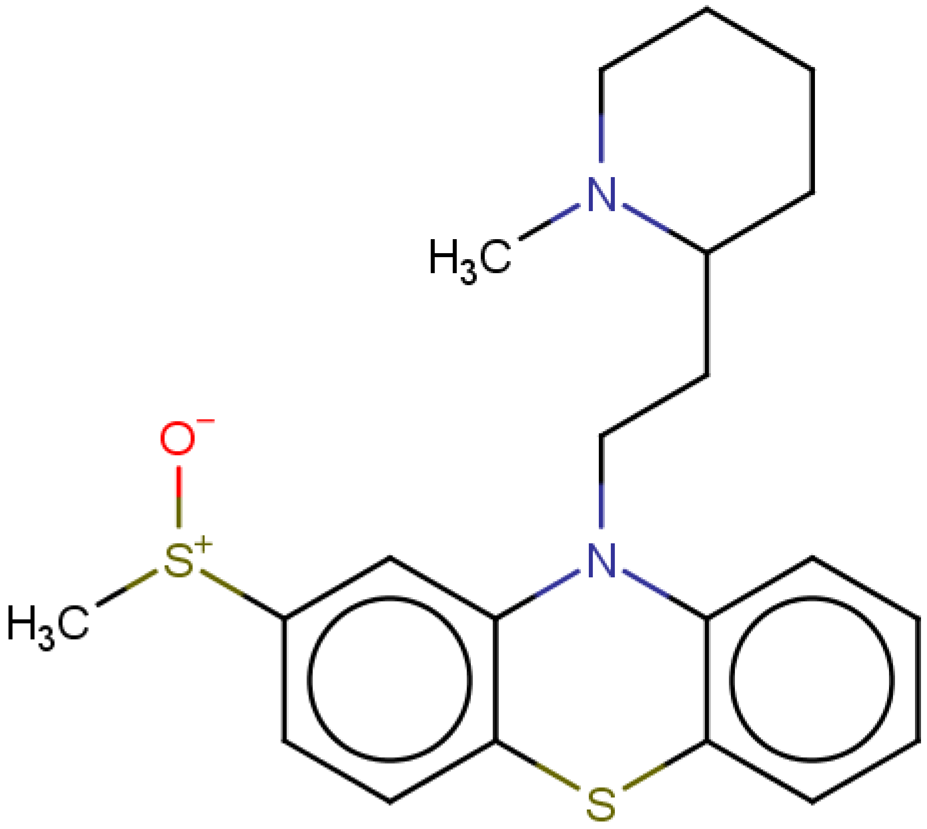

5588-33-0
Name: Mesoridazine
pIC50: 5.12
Rank: 207
Classes: Drug

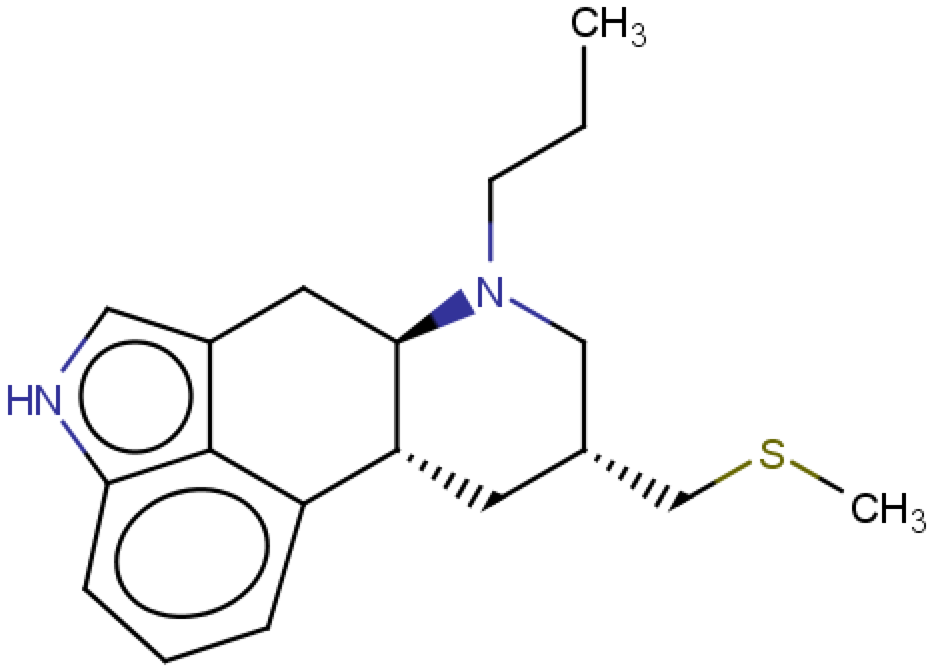

66104-23-2
Name: Pergolide methanesulfonate
pIC50: 5.12
Rank: 208
Classes: No defined

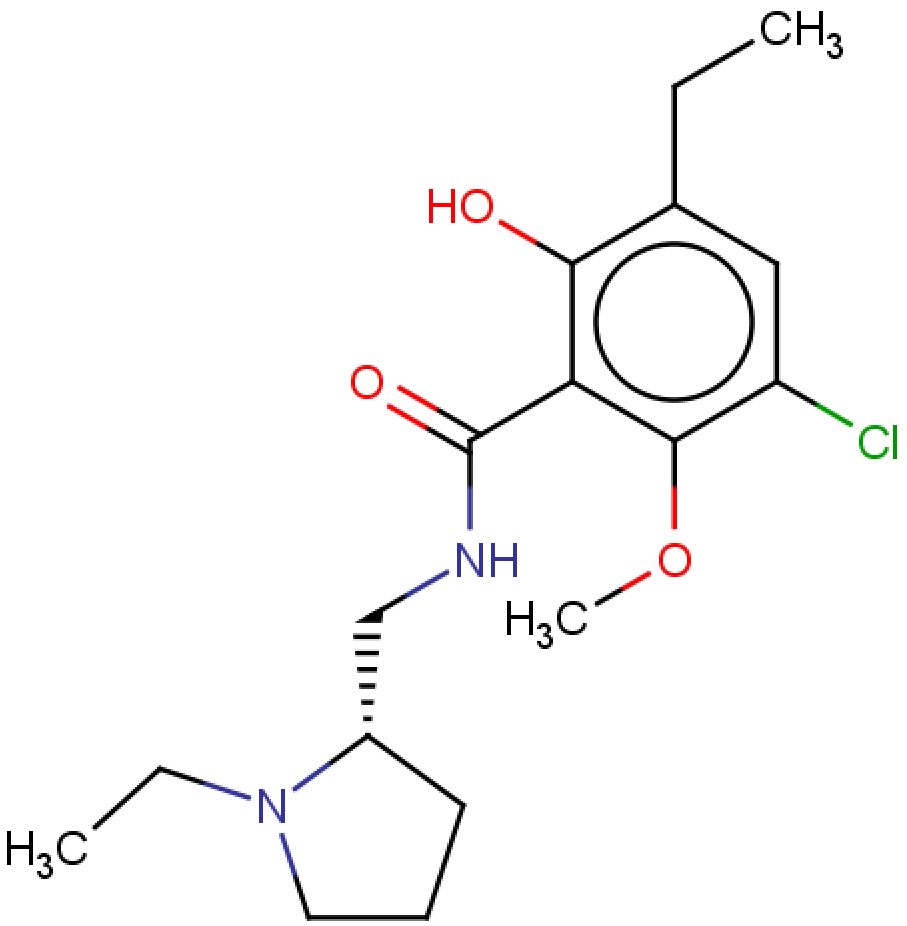

97612-24-3
Name: Eticlopride hydrochloride
pIC50: 5.12
Rank: 209
Classes: No defined

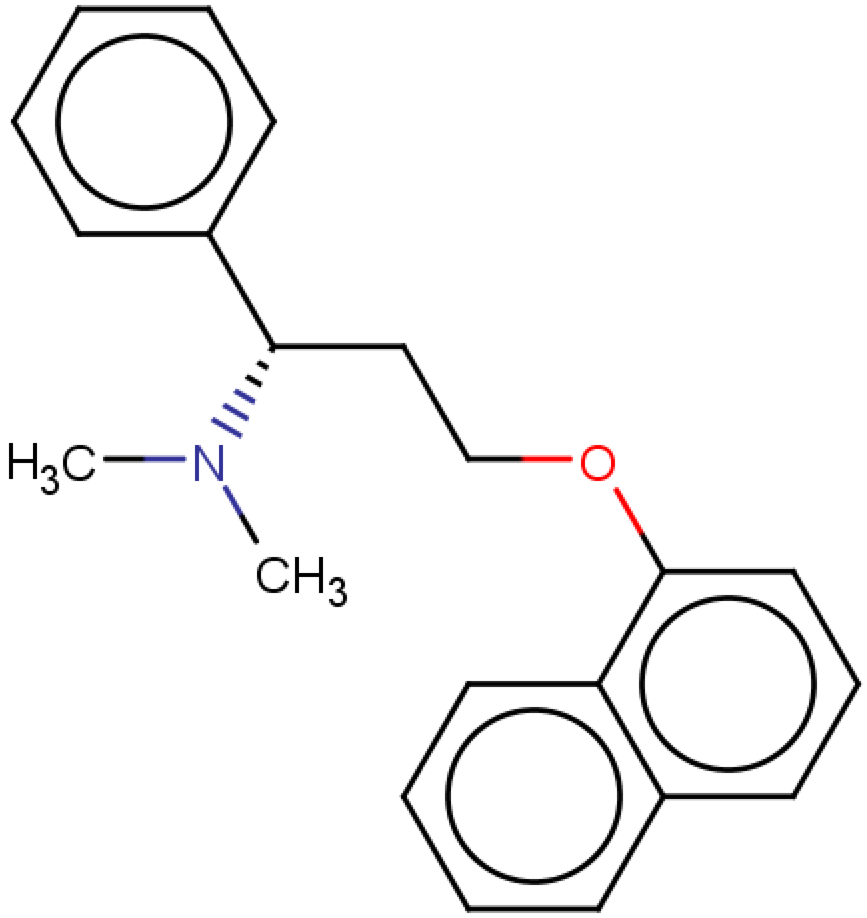

119356-77-3
Name: Dapoxetine
pIC50: 5.12
Rank: 210
Classes: Drug

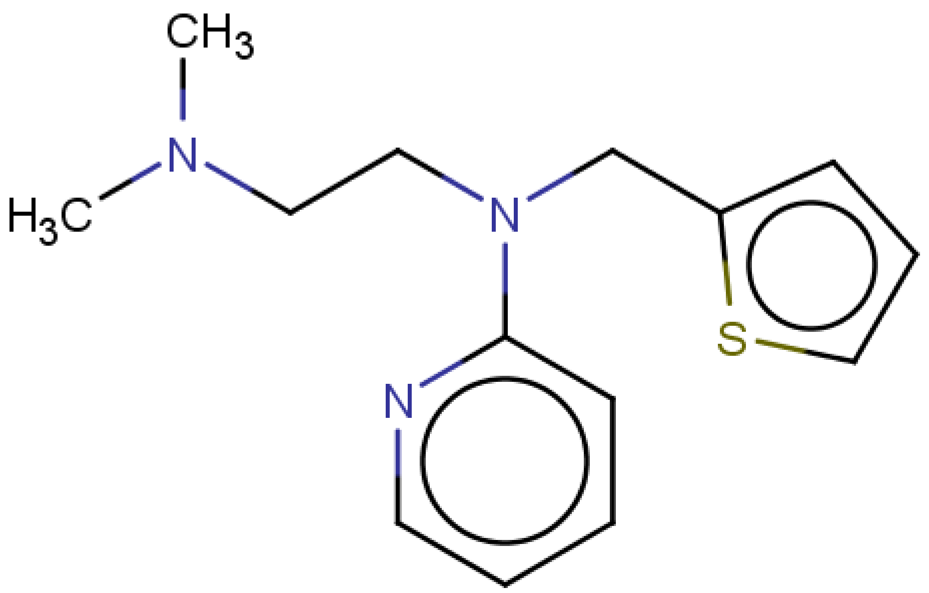

33032-12-1
Name: Methapyrilene fumarate
pIC50: 5.11
Rank: 211
Classes: No defined

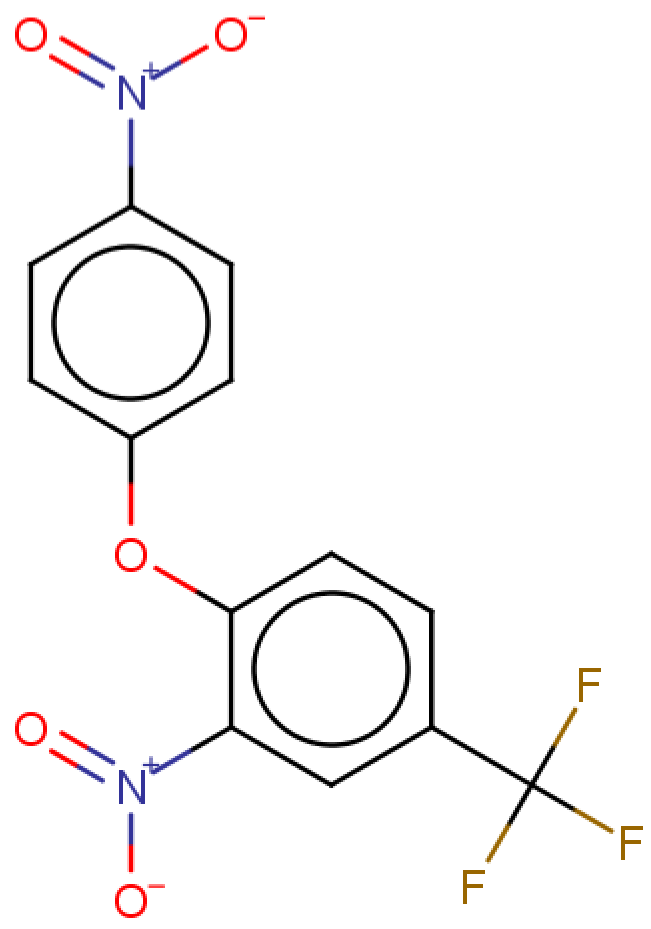

15457-05-3
Name: Fluorodifen
pIC50: 5.11
Rank: 212
Classes: No defined

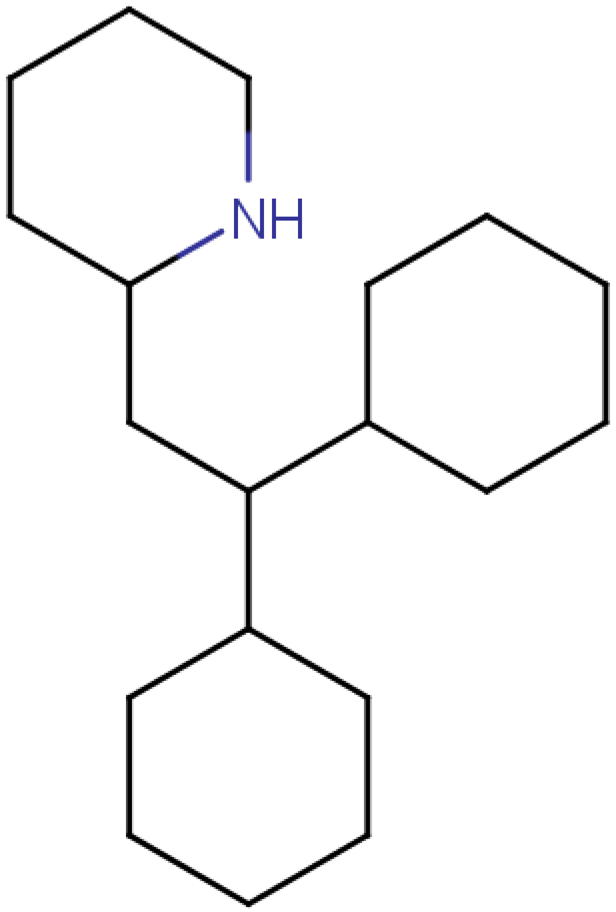

6724-53-4
Name: Perhexiline maleate
pIC50: 5.1
Rank: 213
Classes: No defined

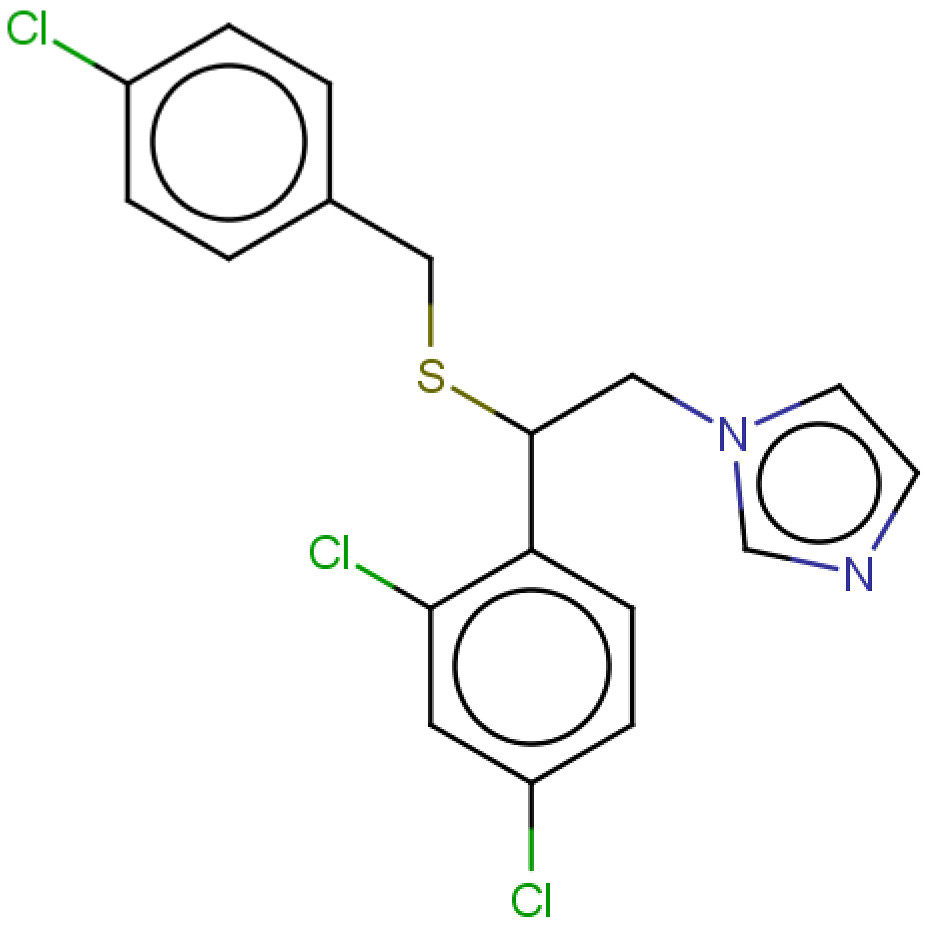

61318-91-0
Name: Sulconazole nitrate
pIC50: 5.1
Rank: 214
Classes: No defined

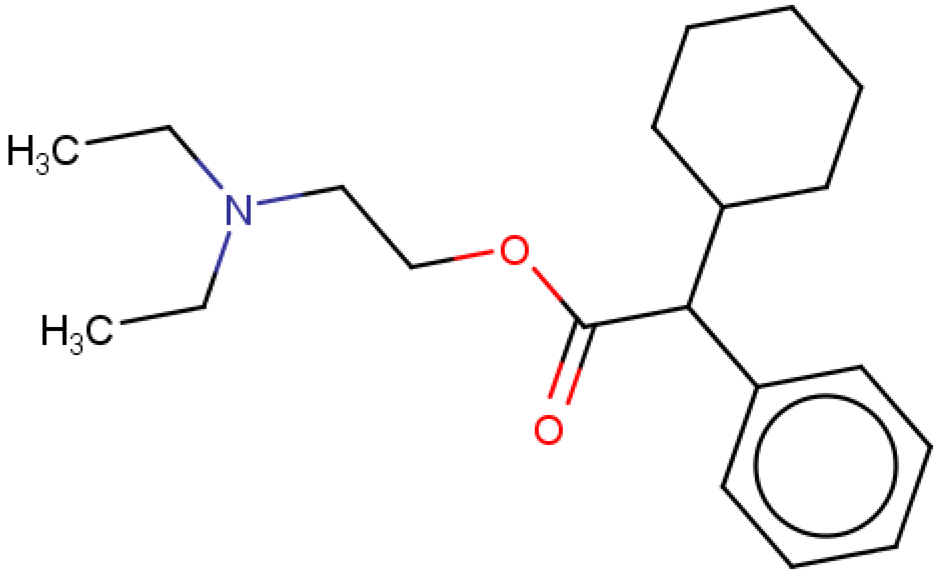

548-66-3
Name: Drofenine hydrochloride
pIC50: 5.1
Rank: 215
Classes: No defined

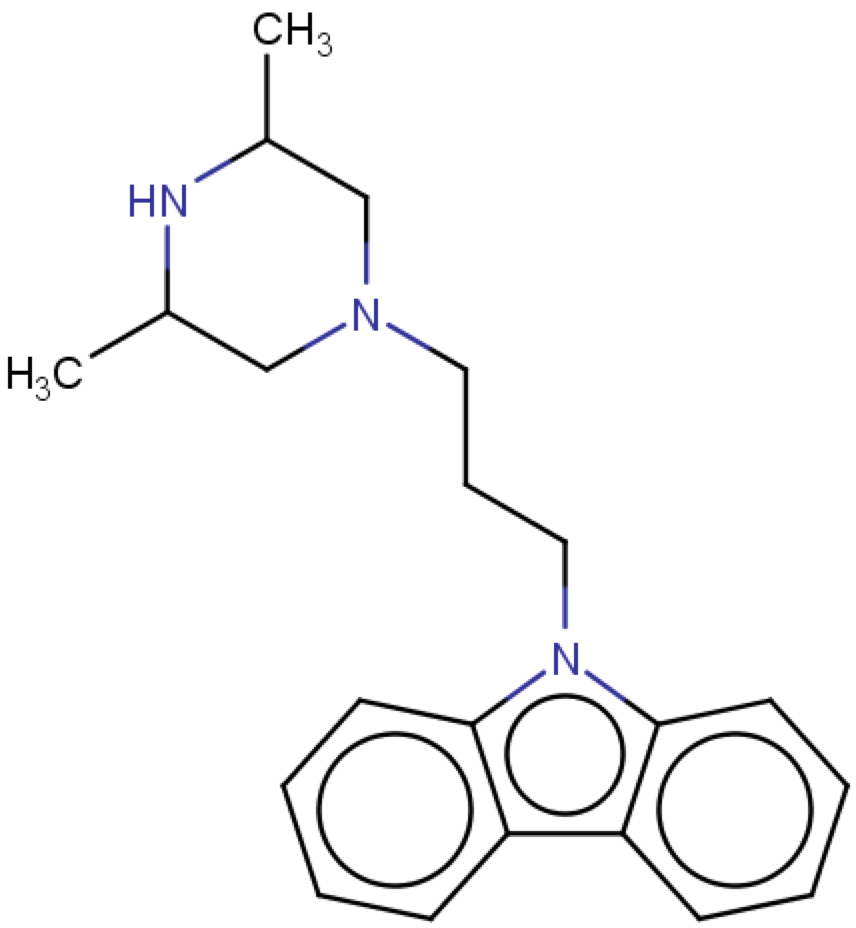

75859-03-9
Name: Rimcazole dihydrochloride
pIC50: 5.1
Rank: 216
Classes: No defined

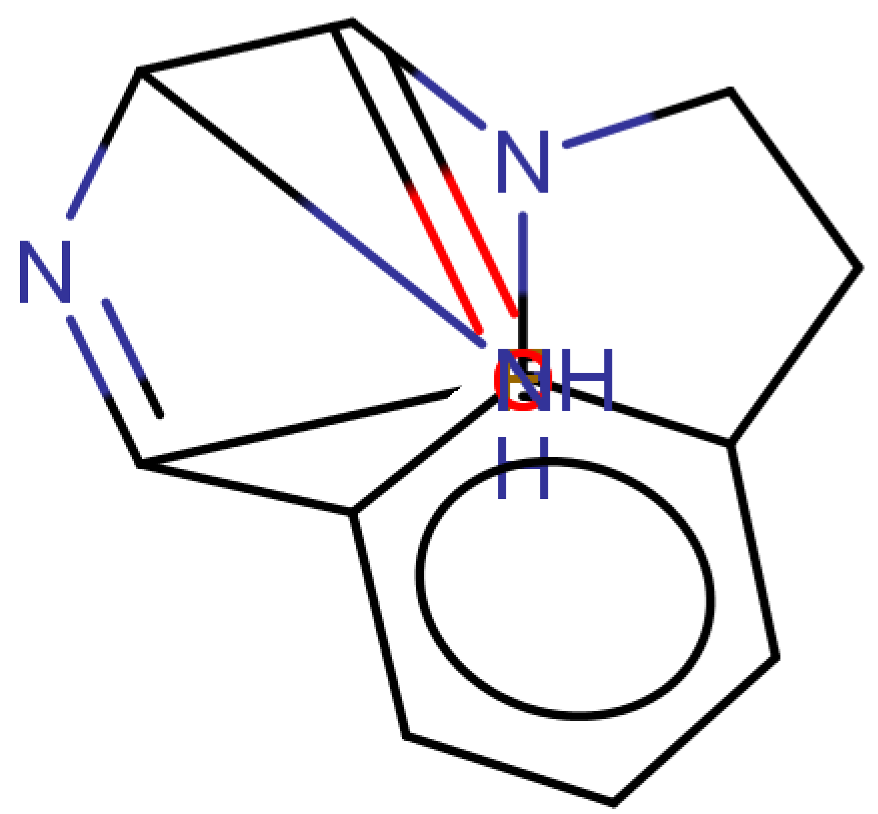

150408-73-4
Name: Pranazepide
pIC50: 5.1
Rank: 217
Classes: No defined

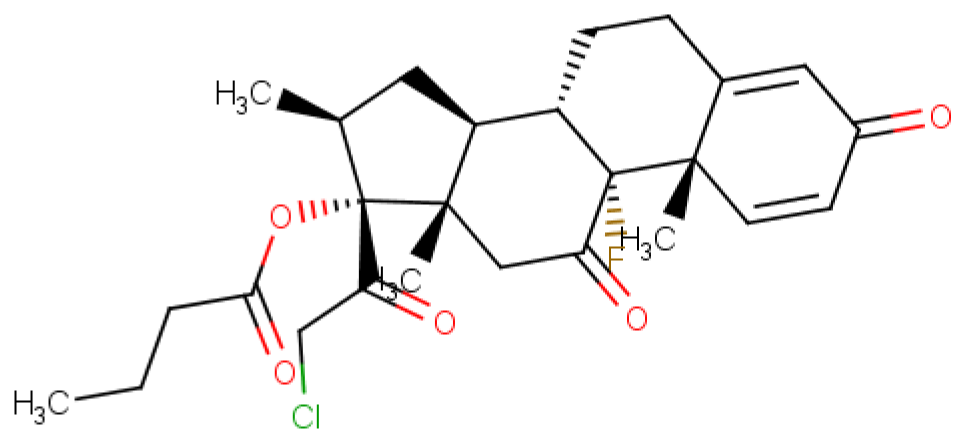

25122-57-0
Name: Clobetasone butyrate
pIC50: 5.1
Rank: 218
Classes: No defined

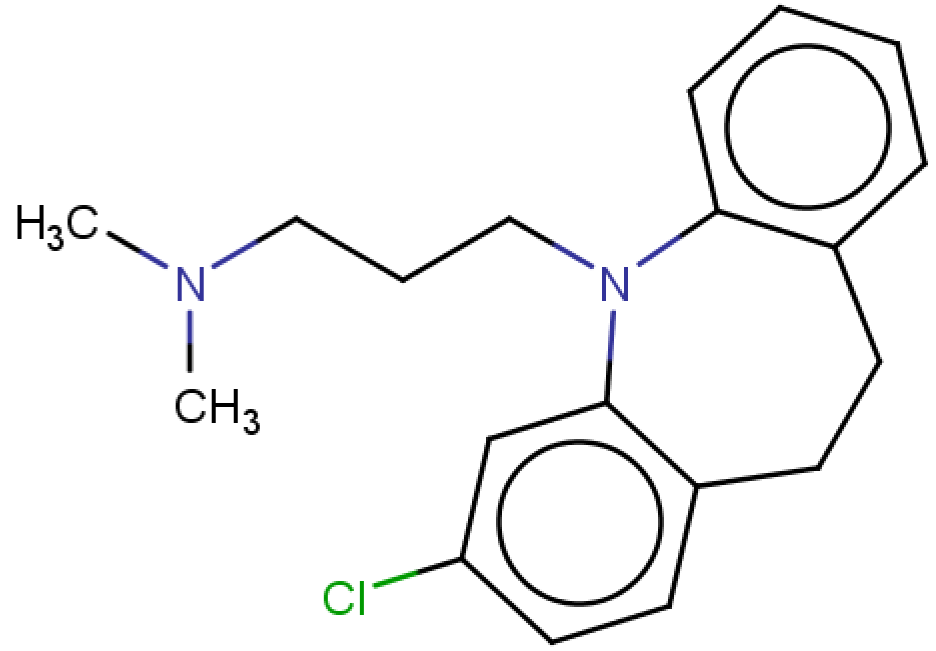

303-49-1
Name: Clomipramine
pIC50: 5.09
Rank: 219
Classes: Drug

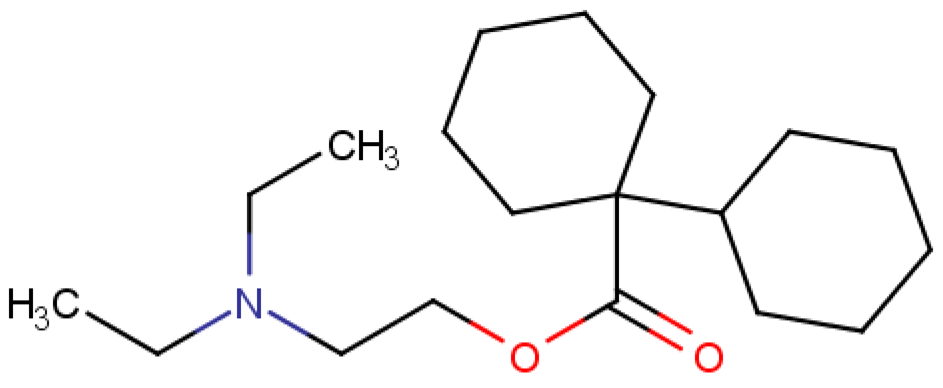

77-19-0
Name: Dicyclomine
pIC50: 5.09
Rank: 220
Classes: Drug

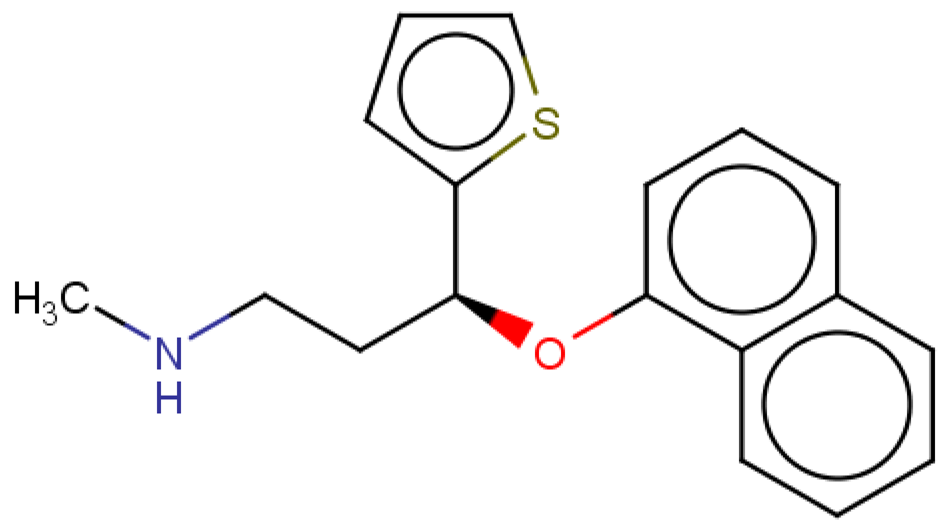

136434-34-9
Name: Duloxetine hydrochloride
pIC50: 5.09
Rank: 221
Classes: No defined

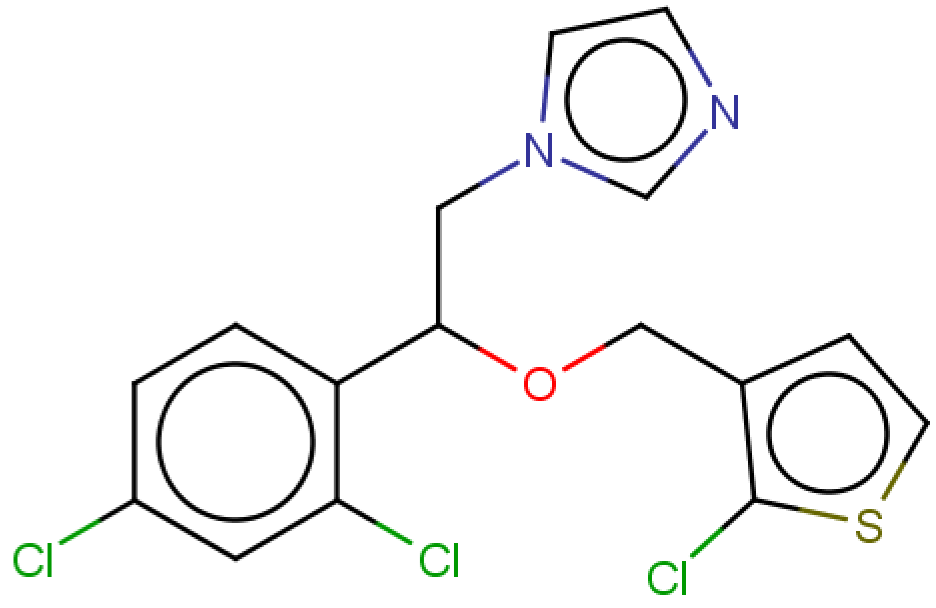

65899-73-2
Name: Tioconazole
pIC50: 5.09
Rank: 222
Classes: Drug

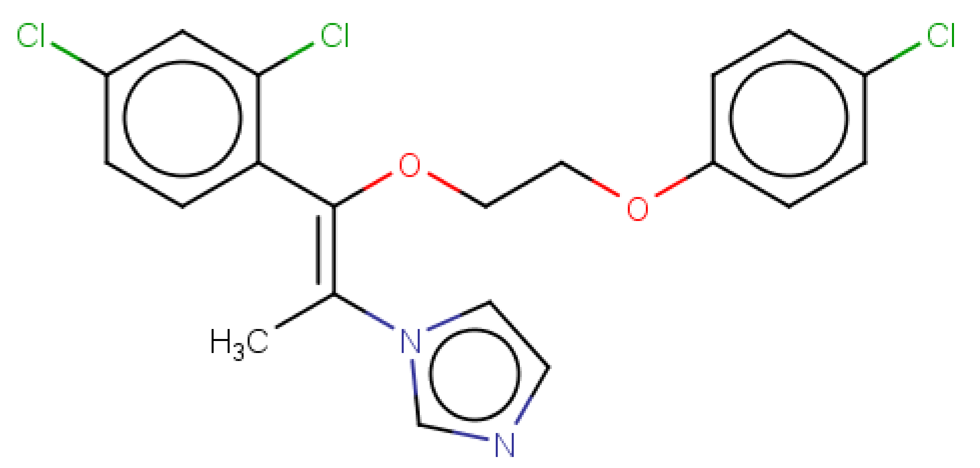

74512-12-2
Name: Omoconazole
pIC50: 5.09
Rank: 223
Classes: No defined

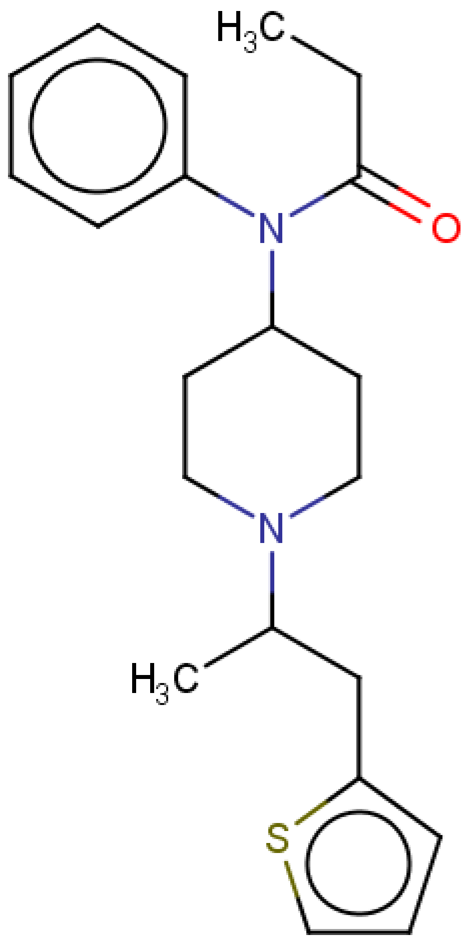

117332-94-2
Name: N-[1-[1-Methyl-2-(2-thienyl)ethyl]-4-pipe[illegible]hloride
pIC50: 5.09
Rank: 224
Classes: No defined

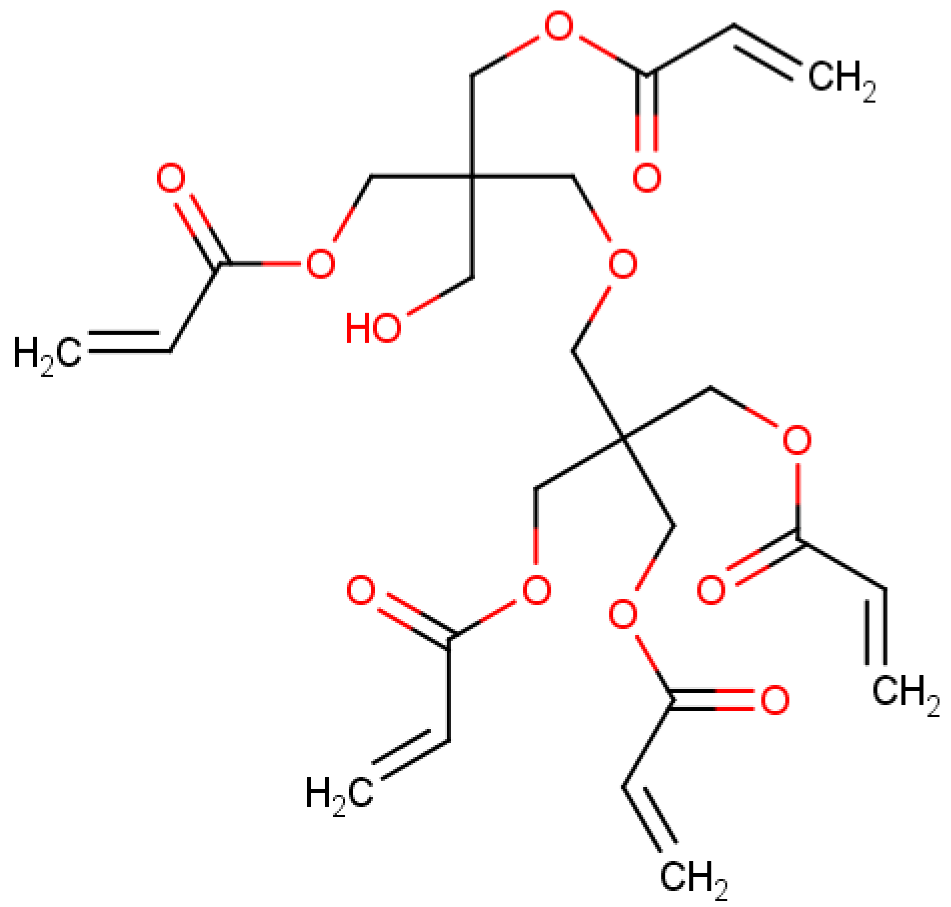

60506-81-2
Name: Dipentaerythritol pentaacrylate
pIC50: 5.08
Rank: 225
Classes: monomer--TSCA

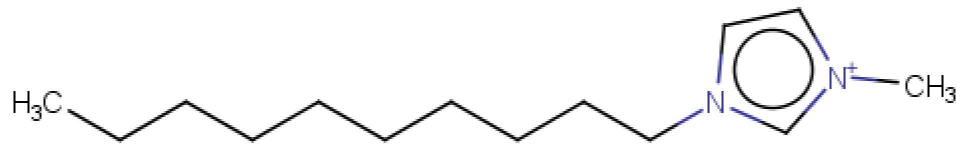

362043-46-7
Name: 1-Decyl-3-methylimidazolium hexafluoro[illegible]
pIC50: 5.08
Rank: 226
Classes: No defined

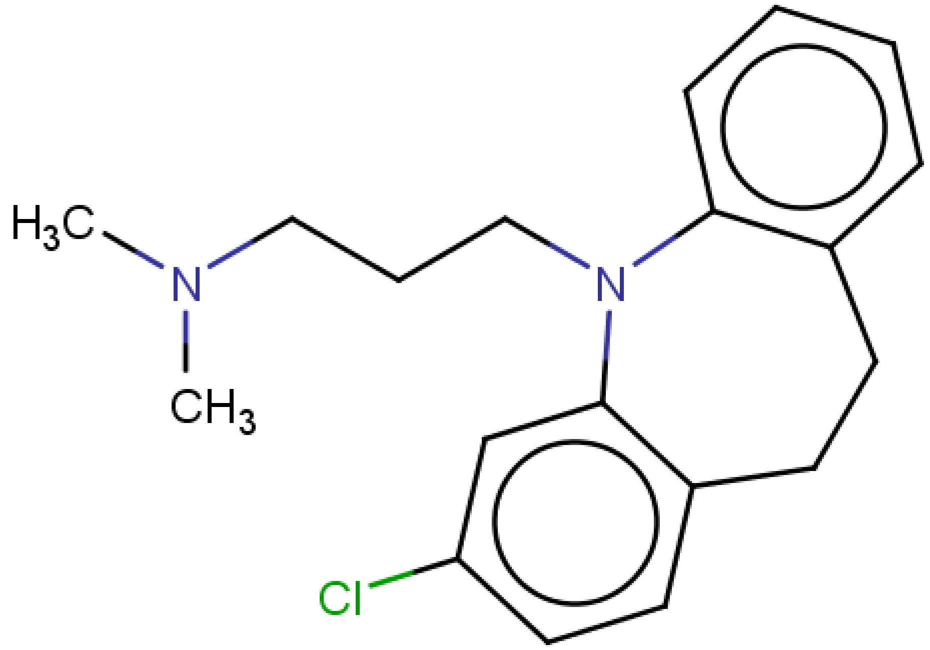

17321-77-6
Name: Clomipramine hydrochloride
pIC50: 5.08
Rank: 227
Classes: No defined

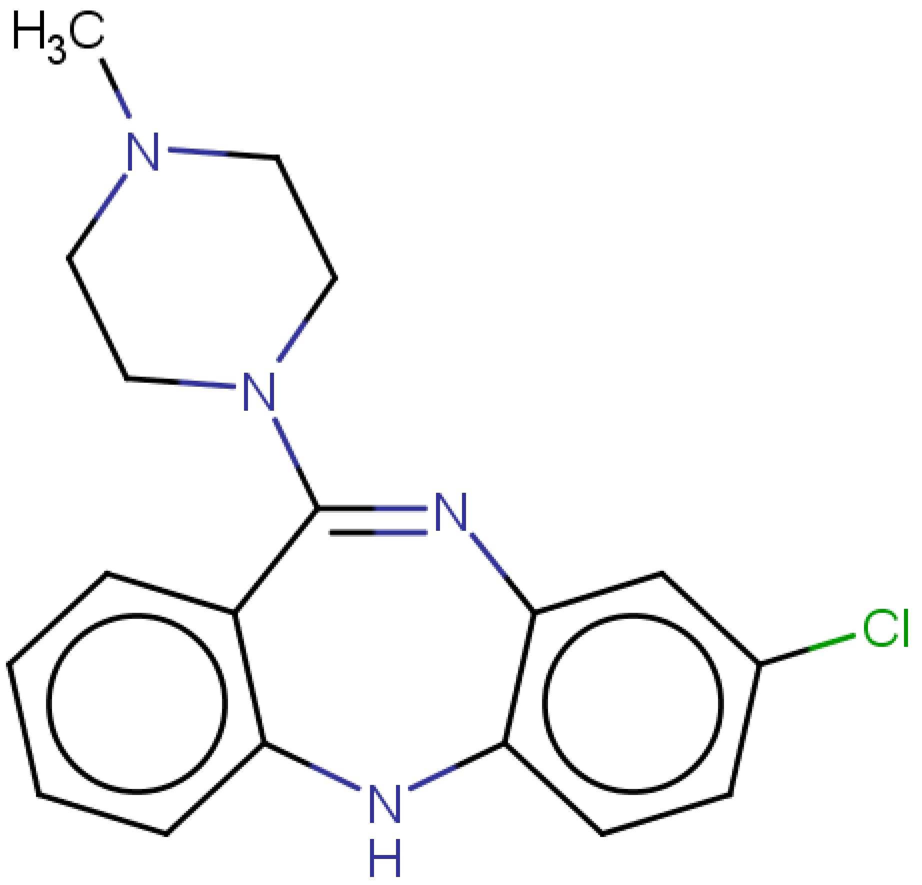

5786-21-0
Name: Clozapine
pIC50: 5.07
Rank: 228
Classes: Drug

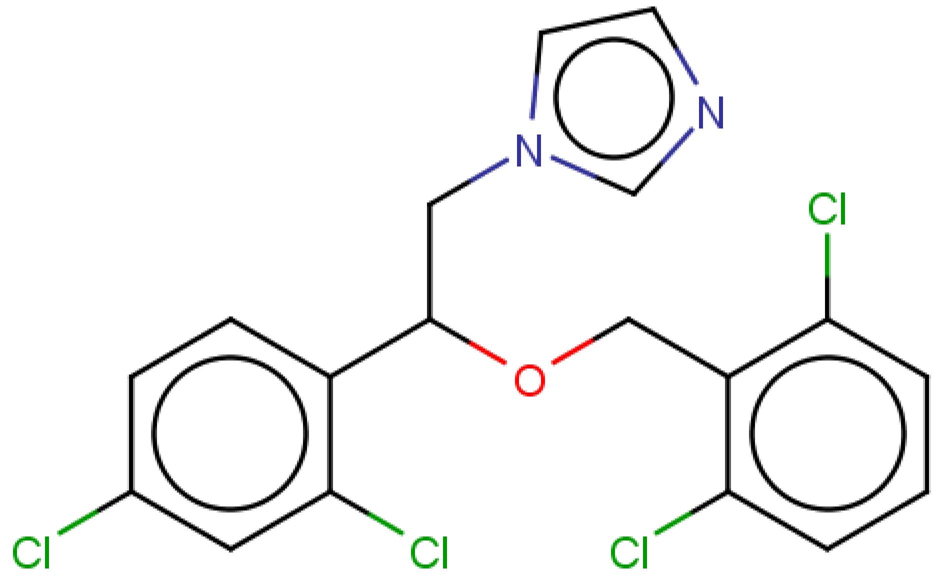

27523-40-6
Name: Isoconazole
pIC50: 5.07
Rank: 229
Classes: Drug

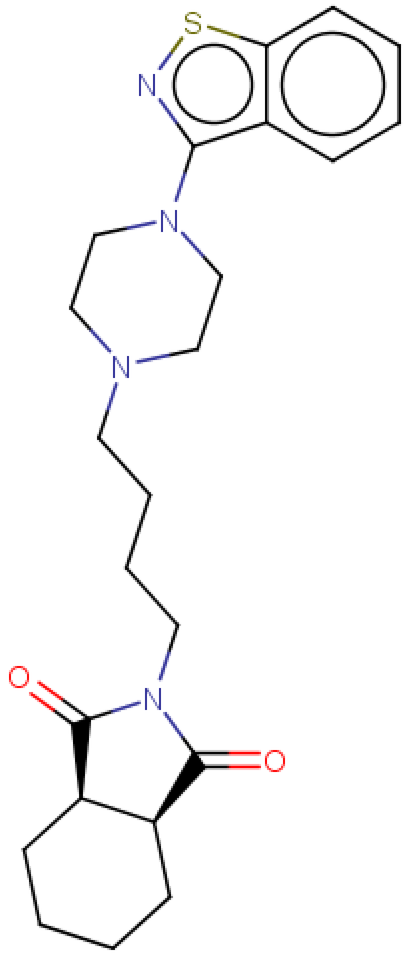

129273-38-7
Name: Perospirone hydrochloride
pIC50: 5.07
Rank: 230
Classes: No defined

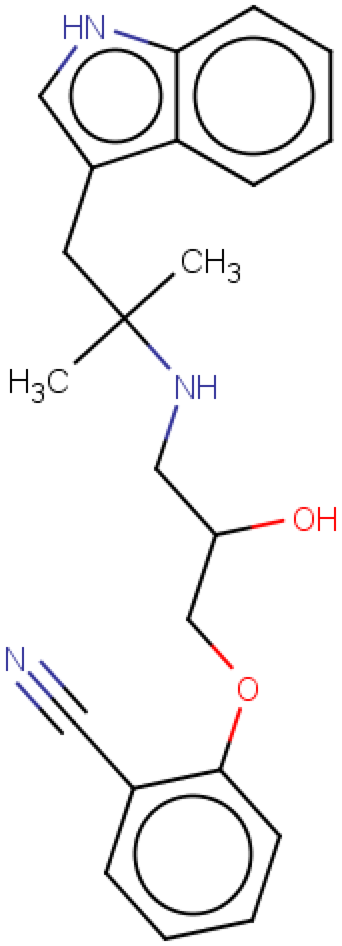

71119-11-4
Name: Bucindolol
pIC50: 5.07
Rank: 231
Classes: Drug

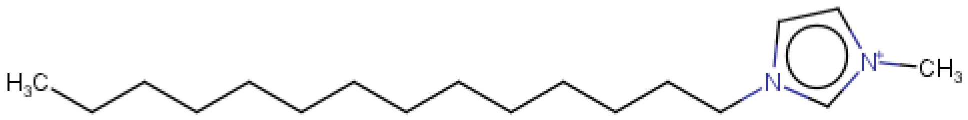

171058-21-2
Name: 1-Methyl-3-tetradecylimidazolium chloride
pIC50: 5.06
Rank: 232
Classes: No defined

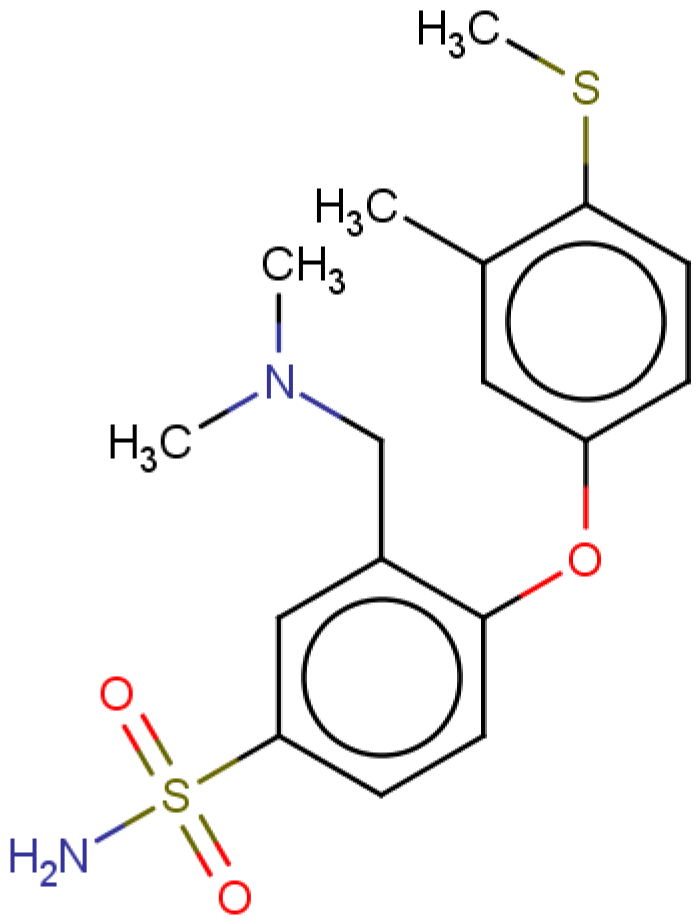

402910-27-4
Name: UK-416244
pIC50: 5.06
Rank: 233
Classes: No defined

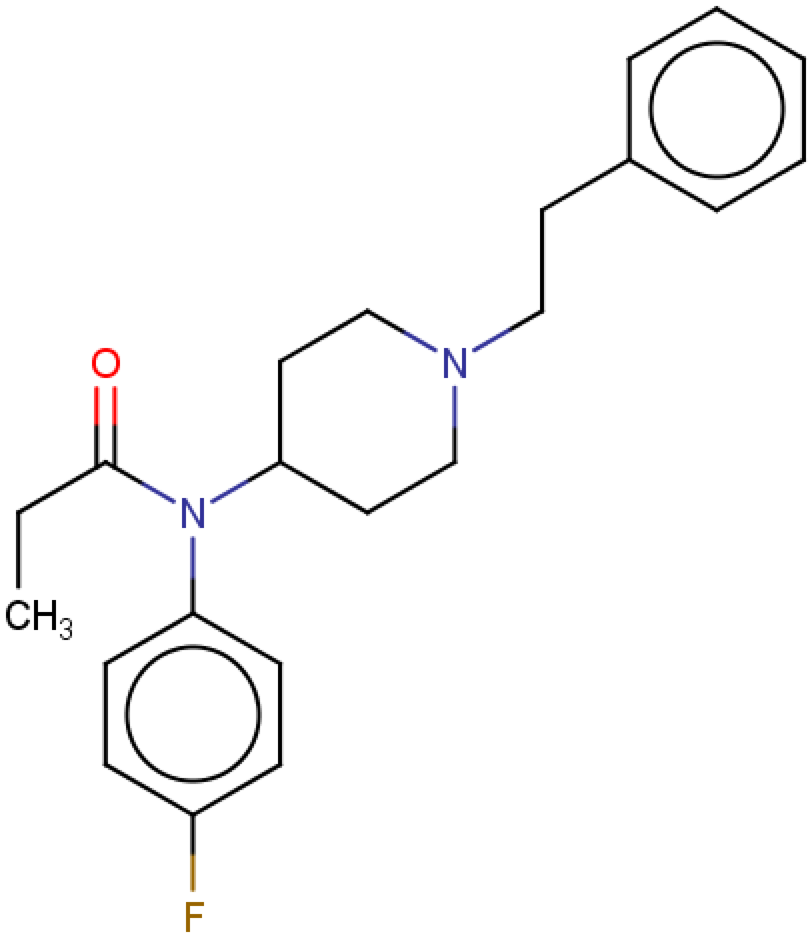

90736-23-5
Name: N-(4-Fluorophenyl)-N-[1-(2-phenylethyl
pIC50: 5.06
Rank: 234
Classes: Drug

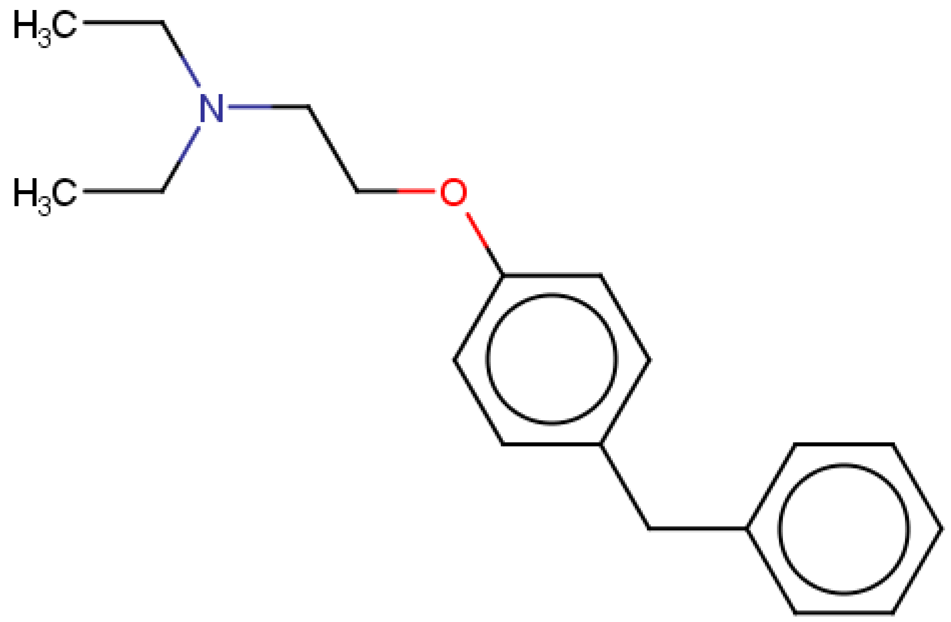

98774-23-3
Name: Tesmilifene
pIC50: 5.05
Rank: 235
Classes: Drug

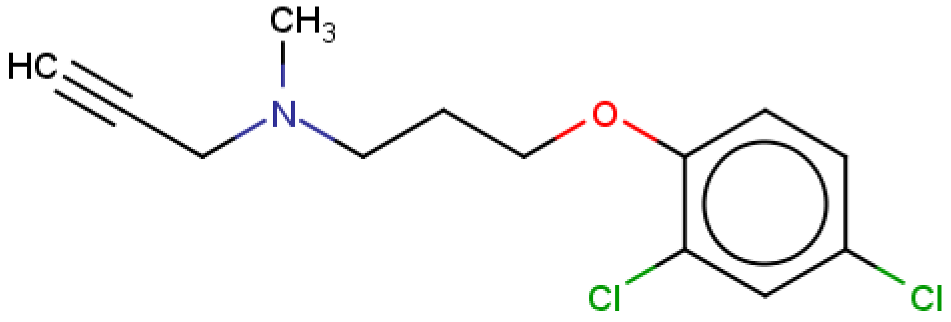

17780-75-5
Name: Clorgyline hydrochloride
pIC50: 5.05
Rank: 236
Classes: No defined

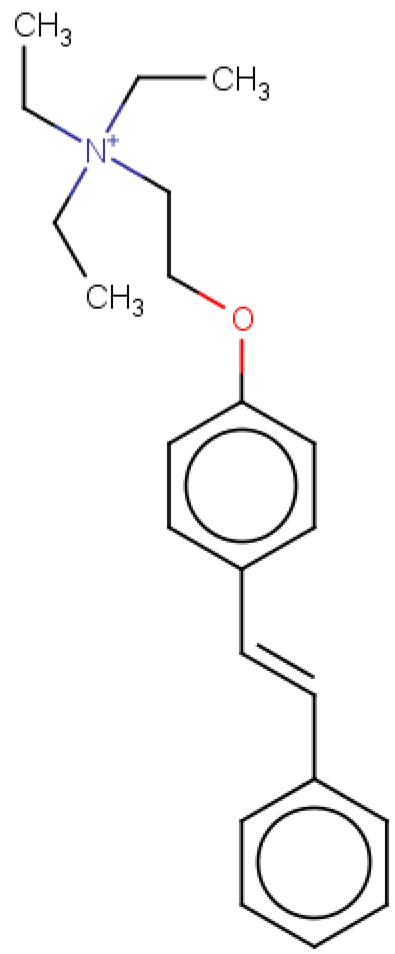

77257-42-2
Name: Stilonium iodide
pIC50: 5.05
Rank: 237
Classes: No defined

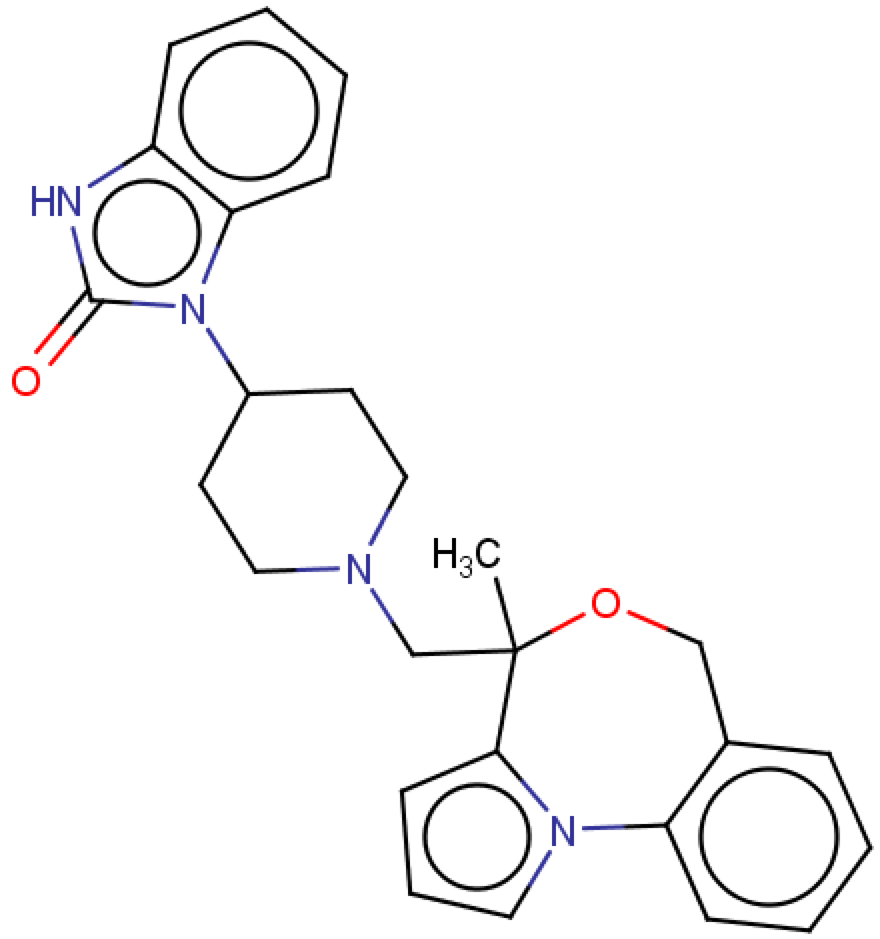

109826-26-8
Name: Zaldaride
pIC50: 5.05
Rank: 238
Classes: No defined

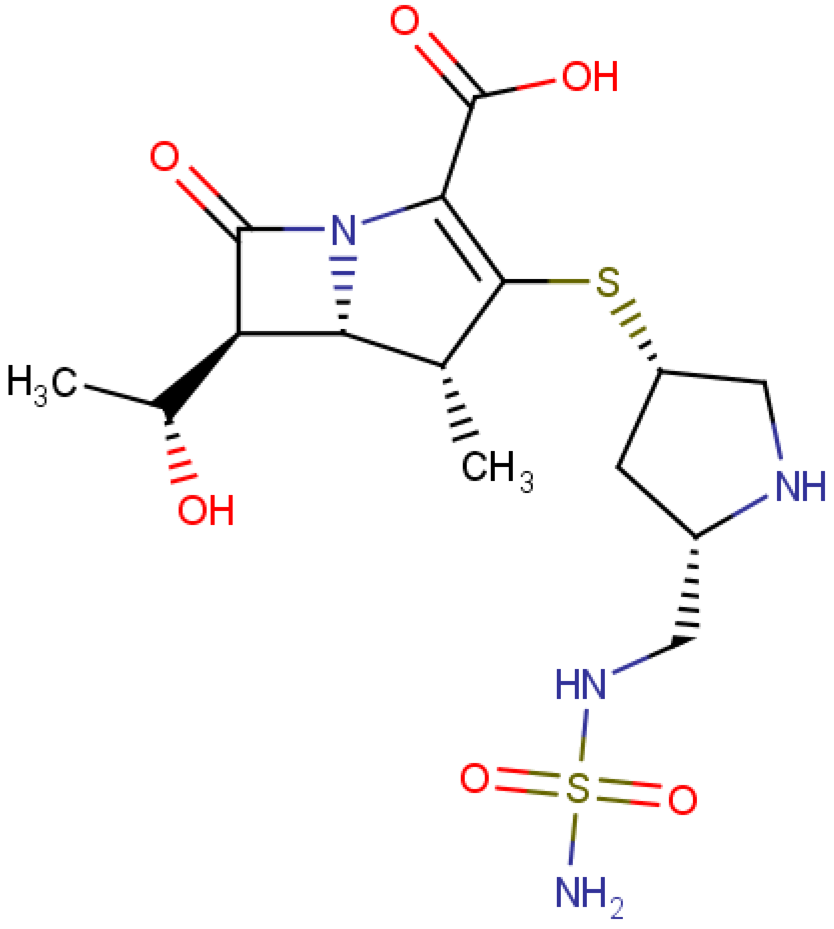

148016-81-3
Name: Doripenem
pIC50: 5.05
Rank: 239
Classes: Drug

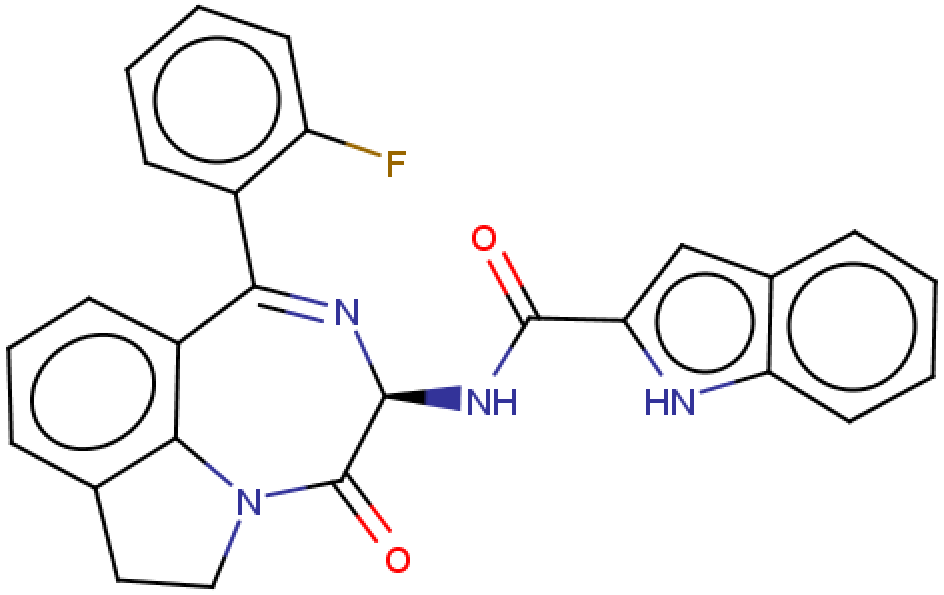

167820-10-2
Name: N-[(3R)-1-(2-Fluorophenyl)-4-oxo-3 4 6
pIC50: 5.05
Rank: 240
Classes: No defined

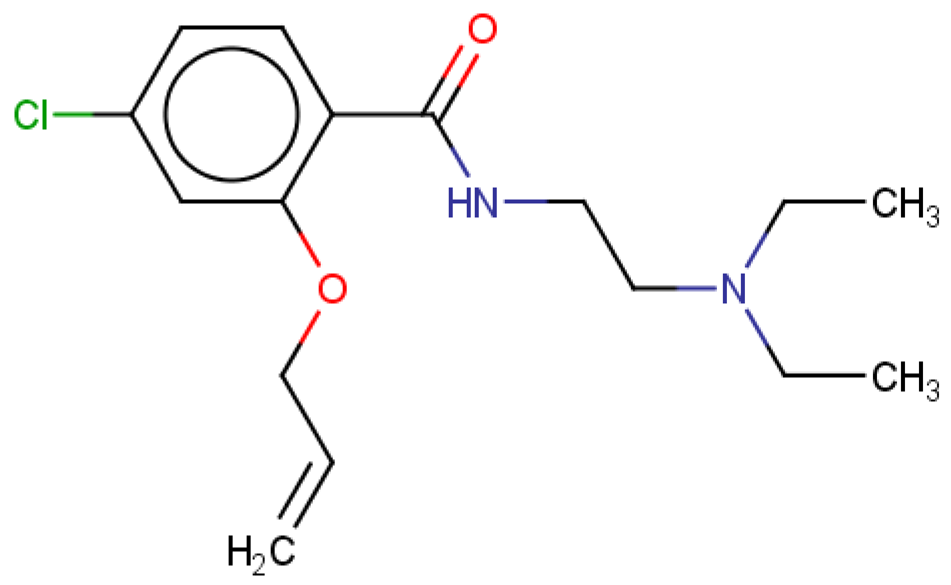

5486-77-1
Name: Alloclamide
pIC50: 5.05
Rank: 241
Classes: No defined

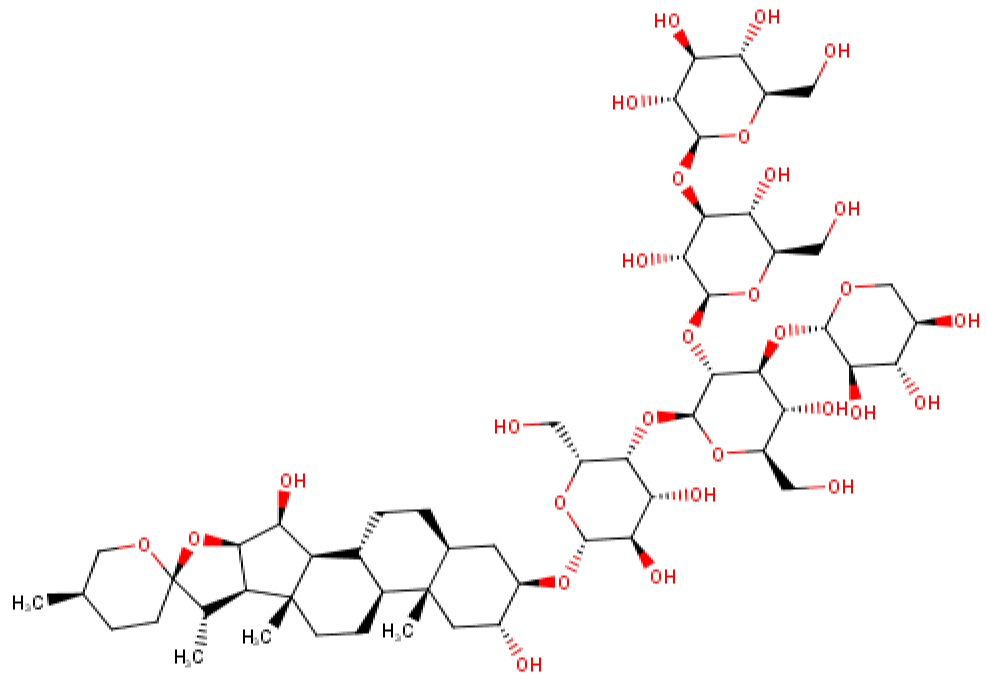

11024-24-1
Name: Digitonin
pIC50: 5.05
Rank: 242
Classes: No defined

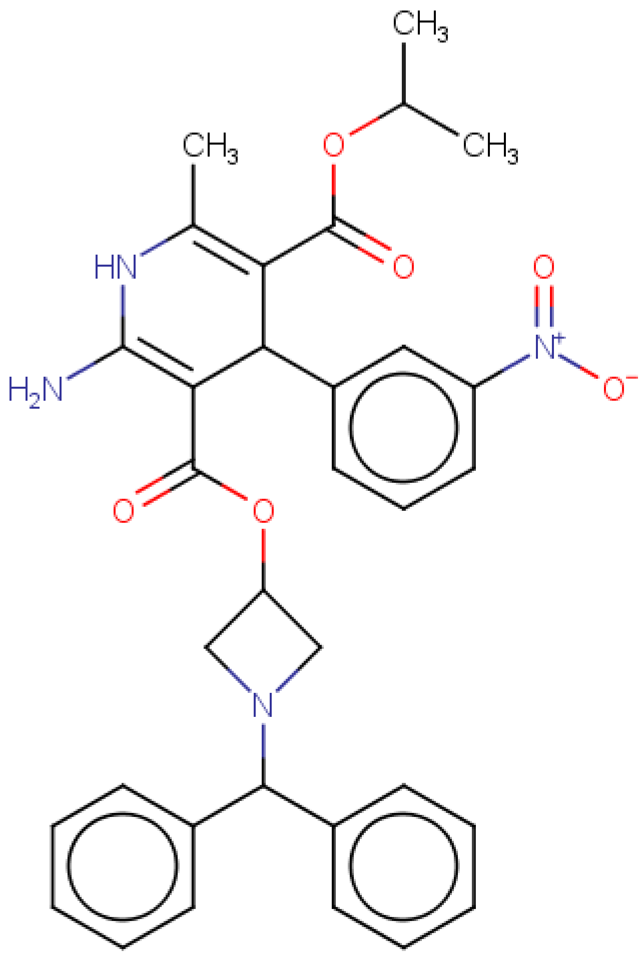

123524-52-7
Name: Azelnidipine
pIC50: 5.05
Rank: 243
Classes: Drug

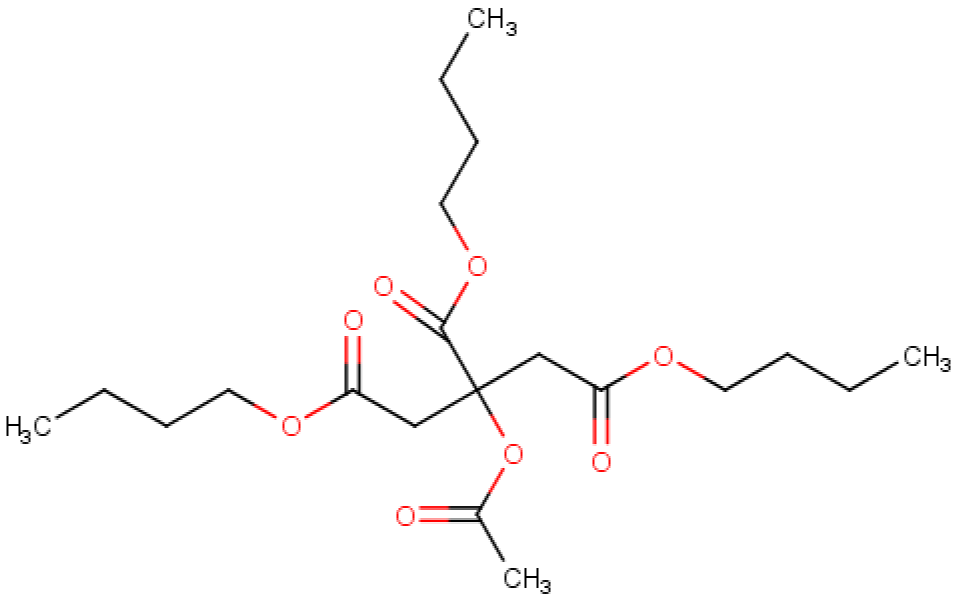

77-90-7
Name: Acetyl tributyl citrate
pIC50: 5.05
Rank: 244
Classes: fragrance--Pesticide--TSCA

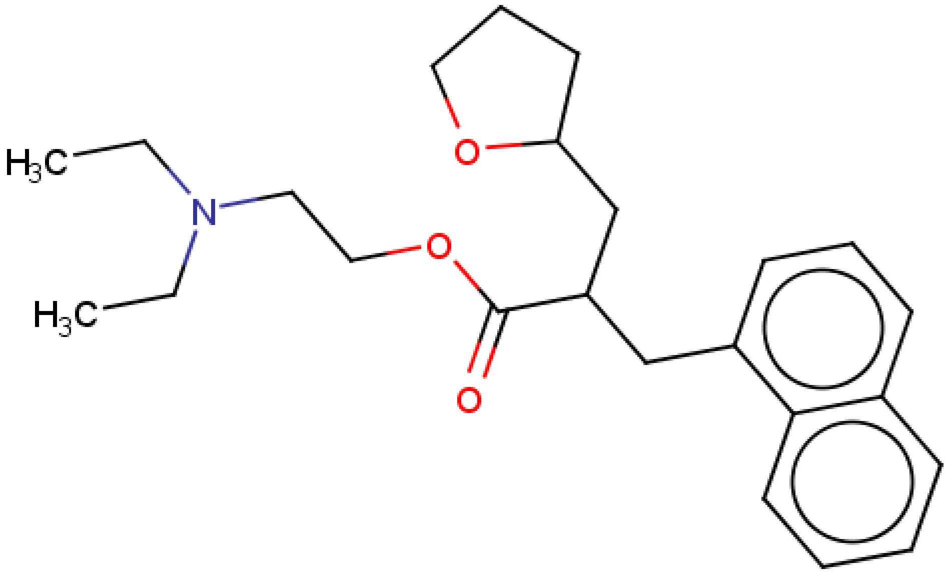

3200-06-4
Name: Nafronyl oxalate
pIC50: 5.05
Rank: 245
Classes: No defined

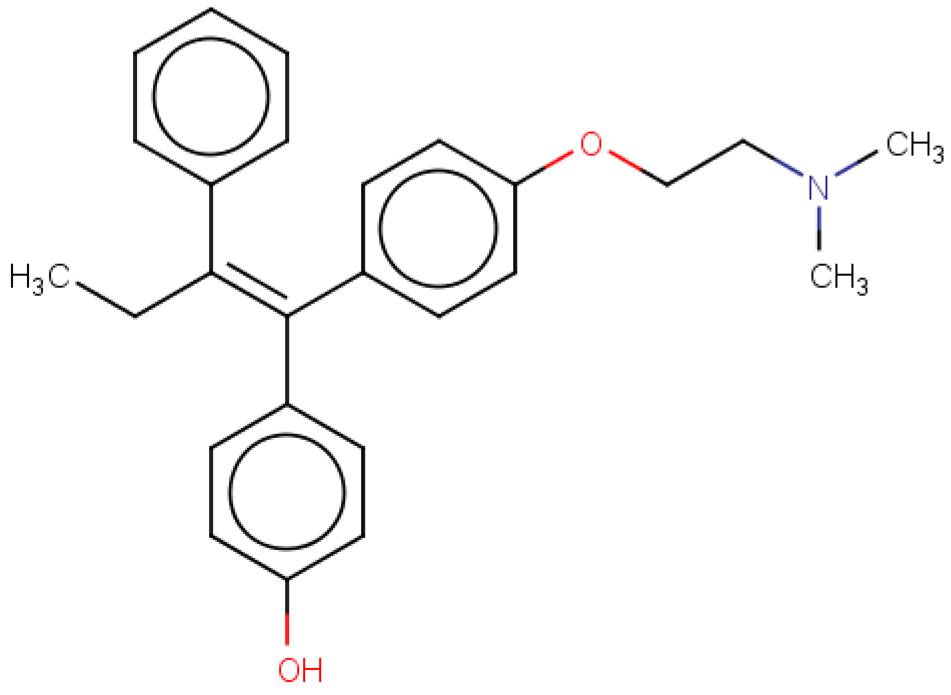

68047-06-3
Name: (Z)-4-Hydroxytamoxifen
pIC50: 5.04
Rank: 246
Classes: Drug

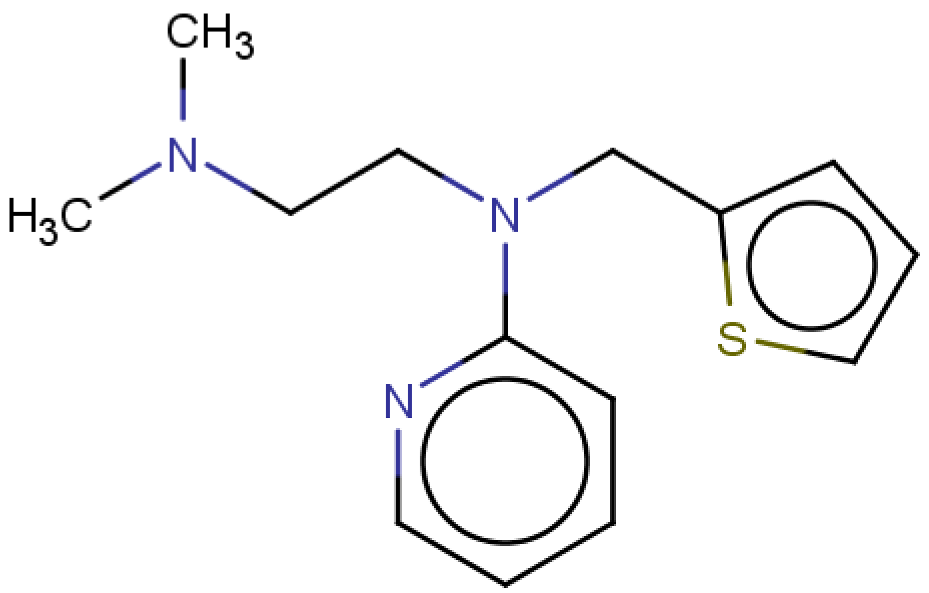

135-23-9
Name: Methapyrilene hydrochloride
pIC50: 5.04
Rank: 247
Classes: Drug

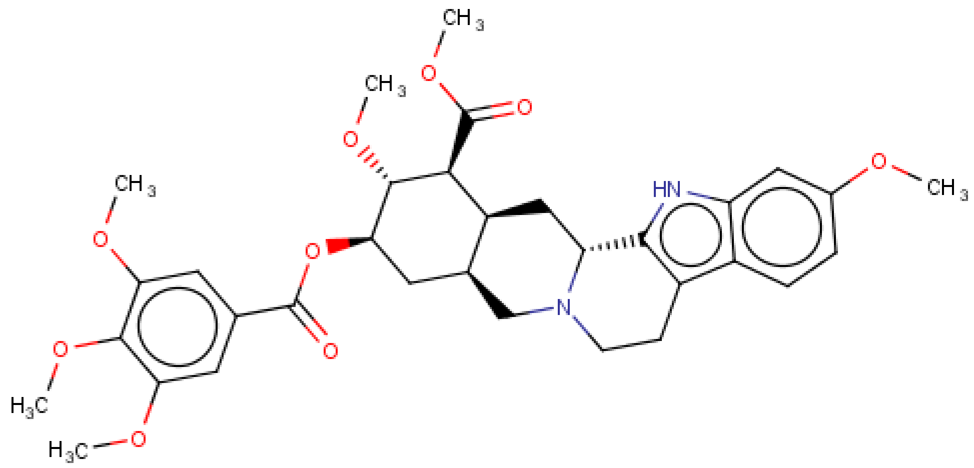

50-55-5
Name: Reserpine
pIC50: 5.04
Rank: 248
Classes: Drug

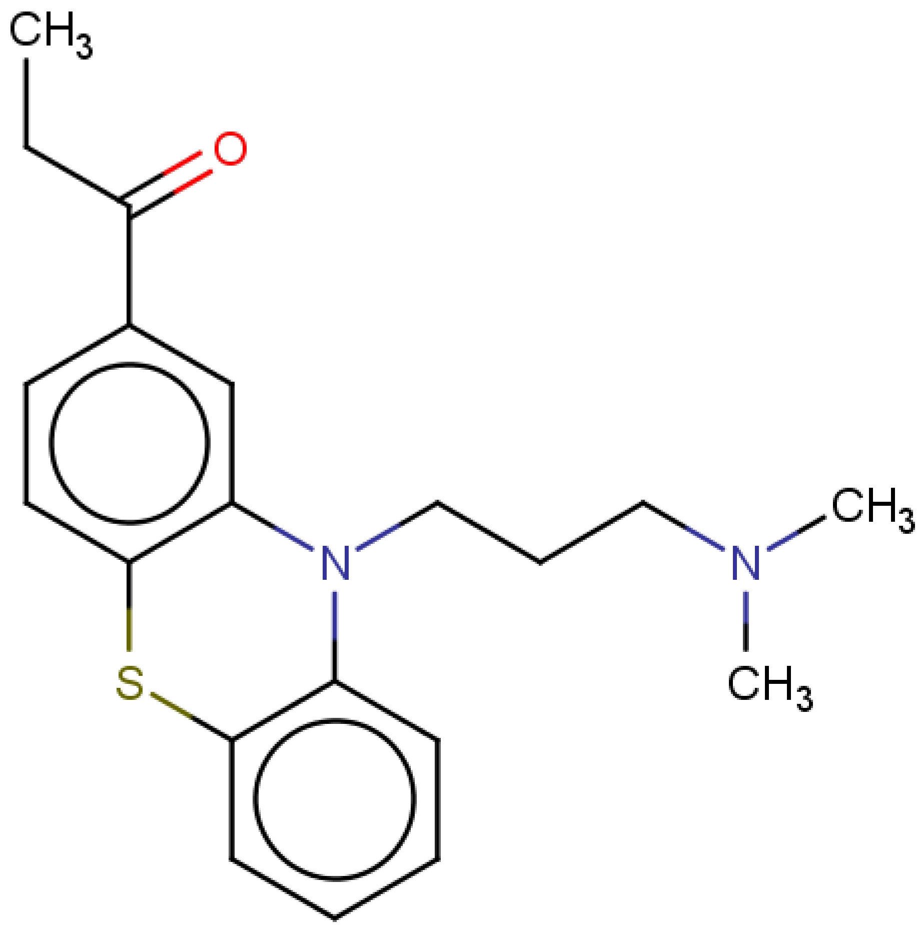

7681-67-6
Name: Propionylpromazine hydrochloride
pIC50: 5.04
Rank: 249
Classes: No defined

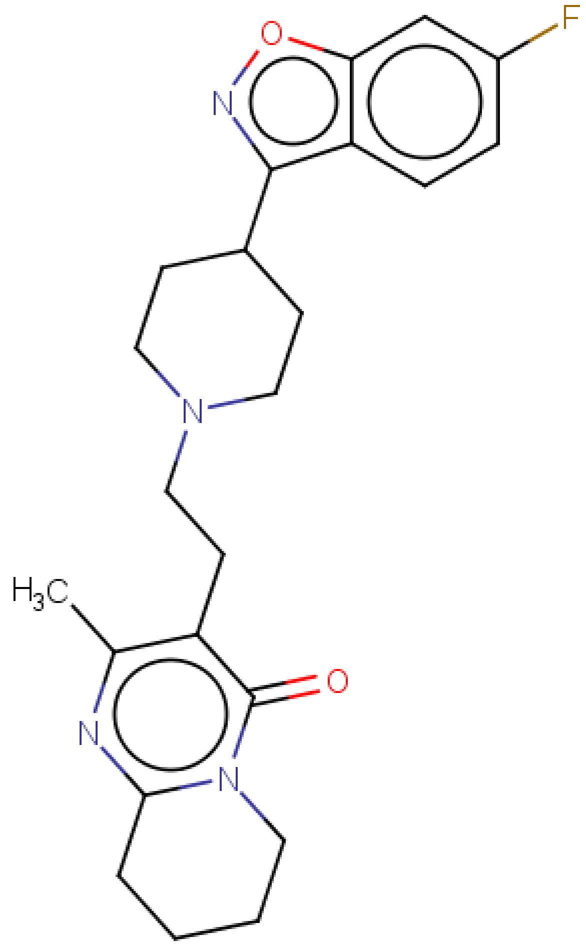

106266-06-2
Name: Risperidone
pIC50: 5.04
Rank: 250
Classes: Drug

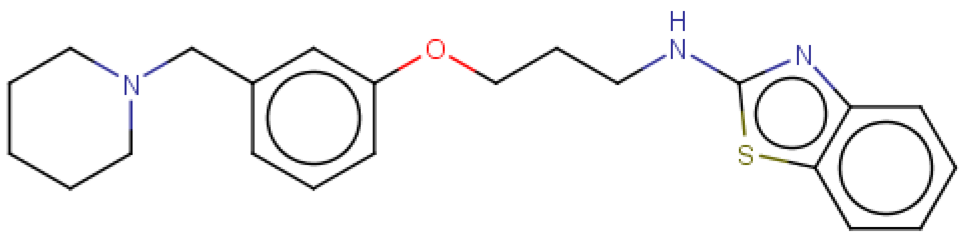

104076-39-3
Name: Zolantidine dimaleate
pIC50: 5.04
Rank: 251
Classes: No defined

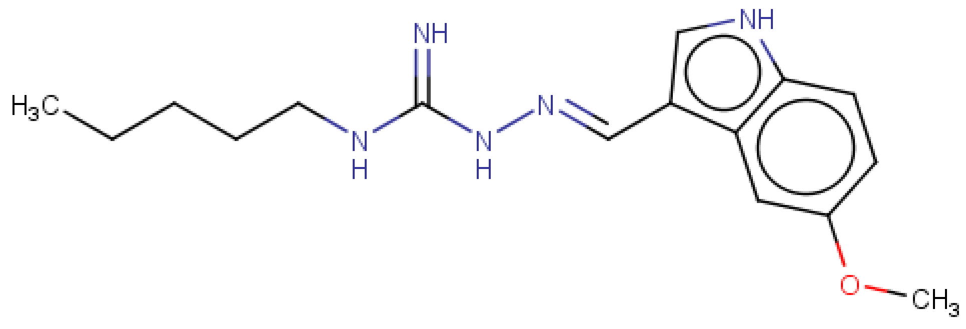

145158-71-0
Name: Tegaserod
pIC50: 5.04
Rank: 252
Classes: Drug

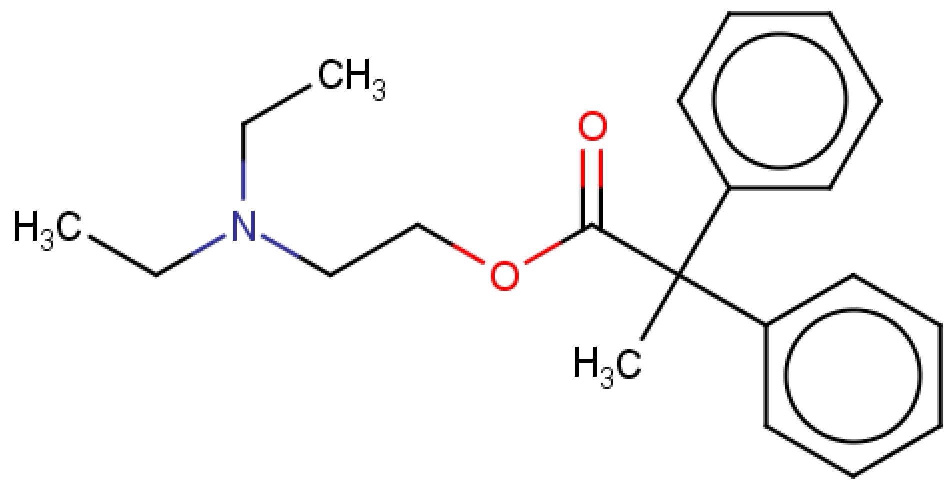

3563-01-7
Name: Aprofene
pIC50: 5.04
Rank: 253
Classes: No defined

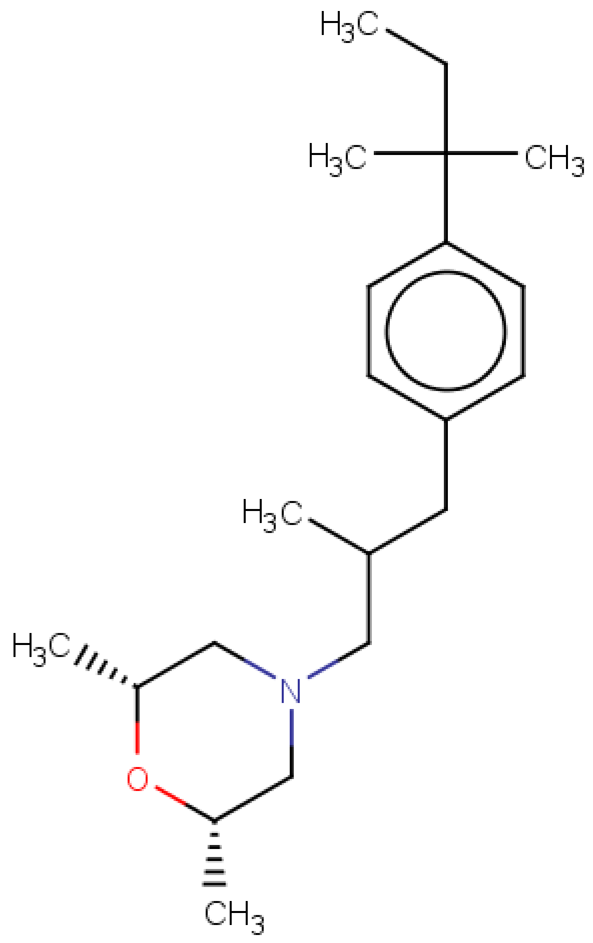

78613-35-1
Name: Amorolfine
pIC50: 5.04
Rank: 254
Classes: No defined

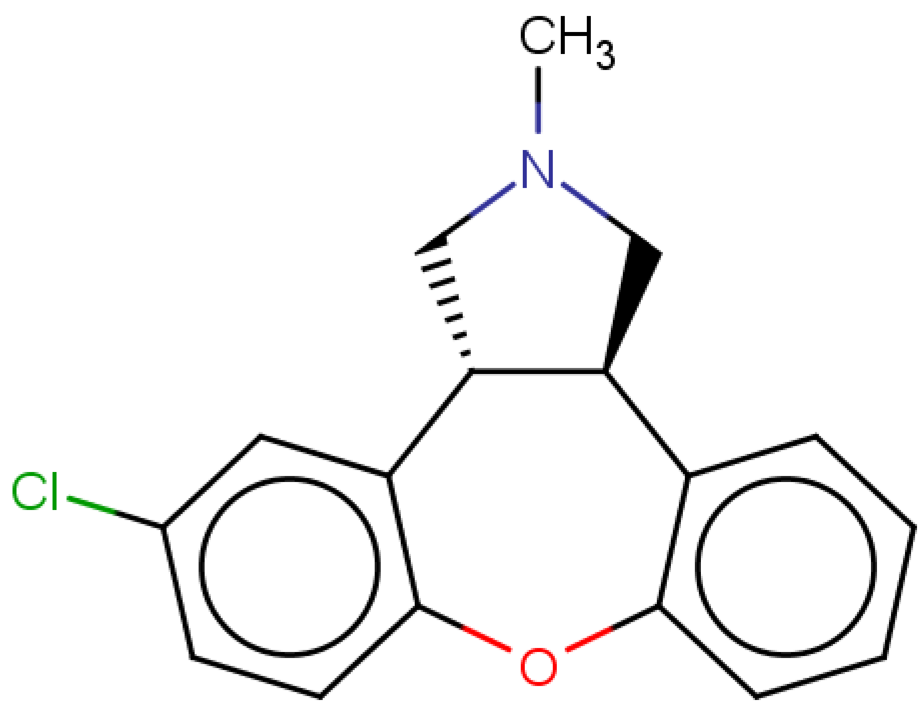

65576-45-6
Name: Asenapine
pIC50: 5.04
Rank: 255
Classes: Drug

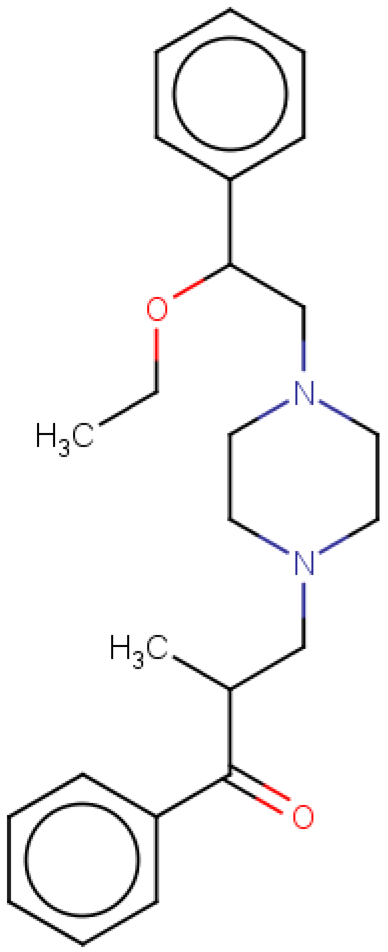

10402-53-6
Name: Eprazinone dihydrochloride
pIC50: 5.03
Rank: 256
Classes: No defined

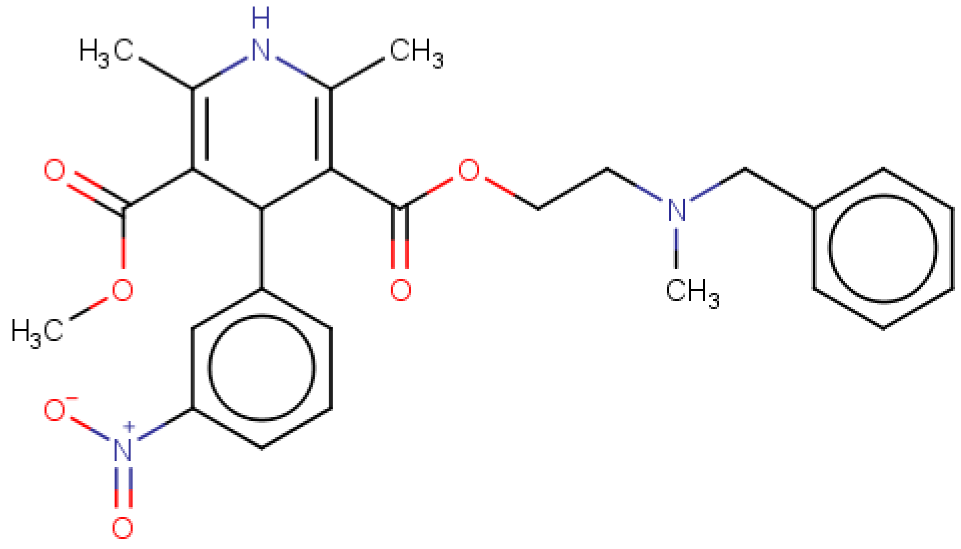

54527-84-3
Name: Nicardipine hydrochloride
pIC50: 5.03
Rank: 257
Classes: No defined

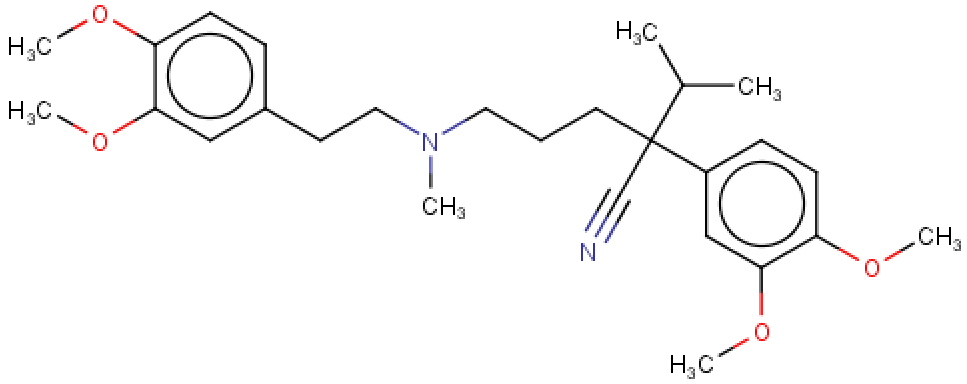

52-53-9
Name: Verapamil
pIC50: 5.02
Rank: 258
Classes: Drug

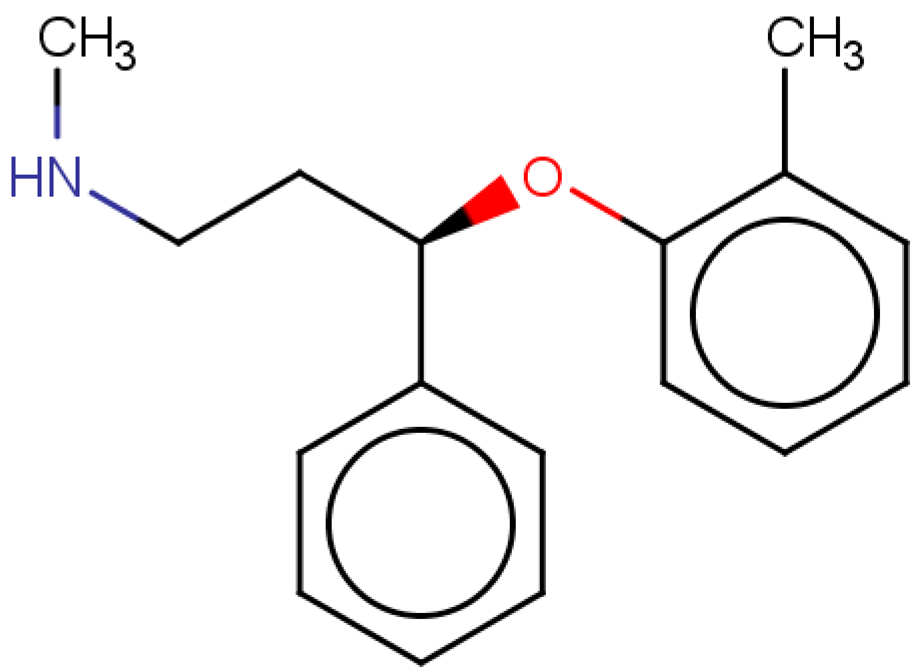

82248-59-7
Name: Atomoxetine hydrochloride
pIC50: 5.02
Rank: 259
Classes: No defined

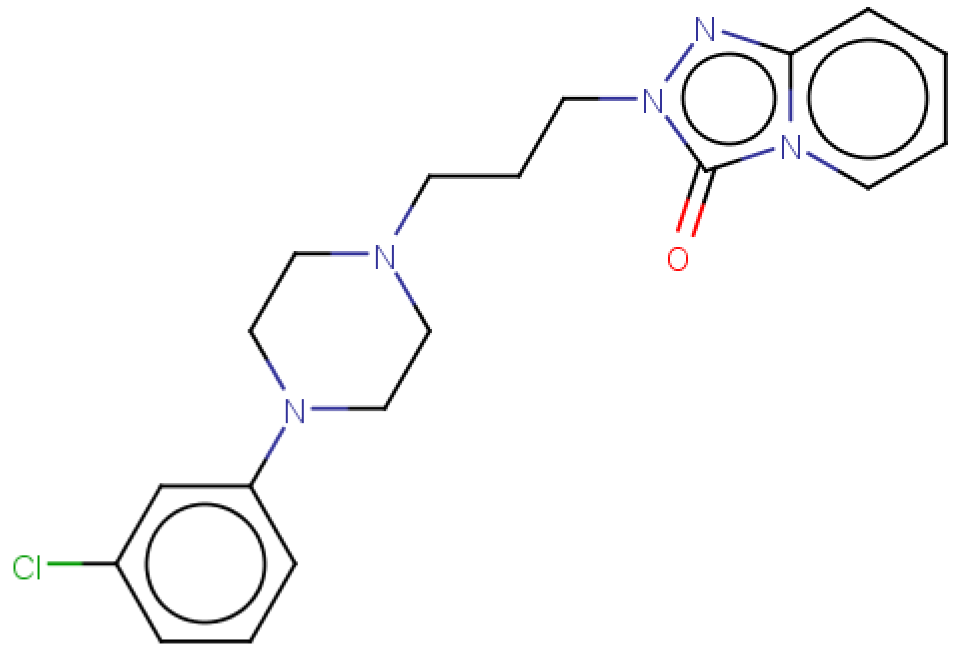

25332-39-2
Name: Trazodone hydrochloride
pIC50: 5.02
Rank: 260
Classes: No defined

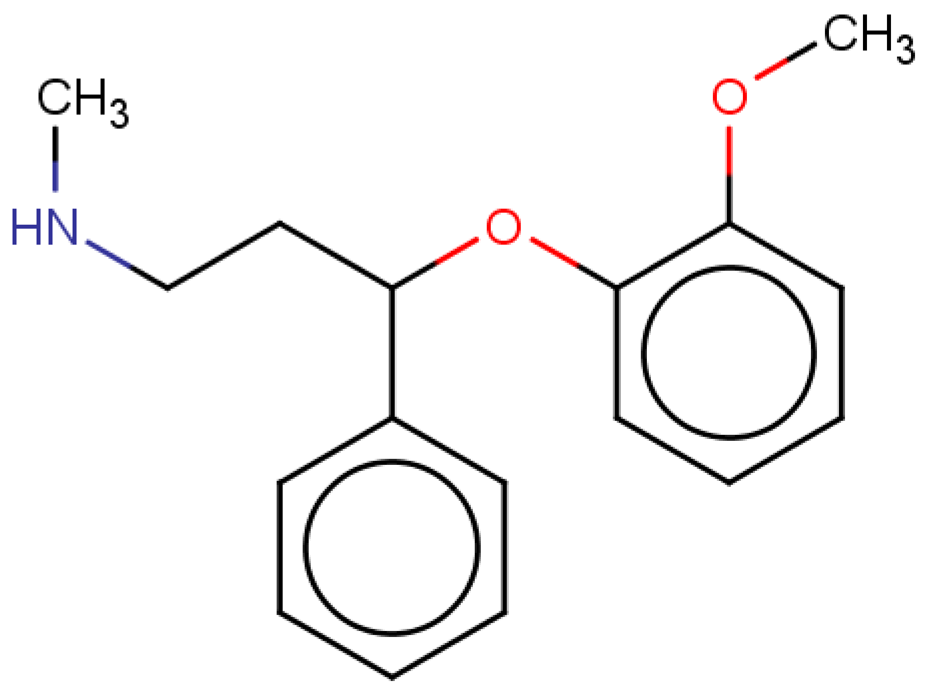

53179-07-0
Name: Nisoxetine
pIC50: 5.02
Rank: 261
Classes: Drug

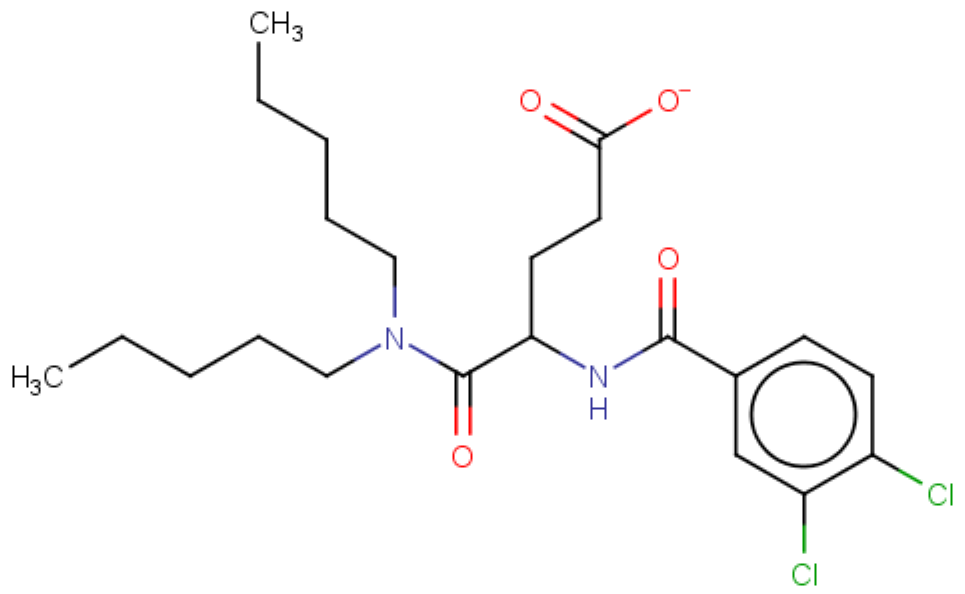

1021868-76-7
Name: Lorglumide sodium
pIC50: 5.02
Rank: 262
Classes: No defined

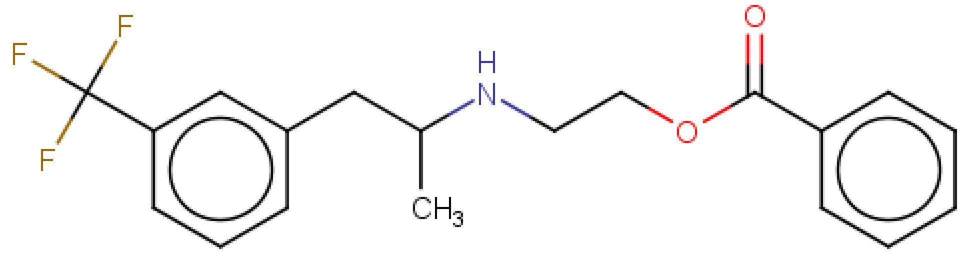

23642-66-2
Name: Benfluorex hydrochloride
pIC50: 5.02
Rank: 263
Classes: No defined

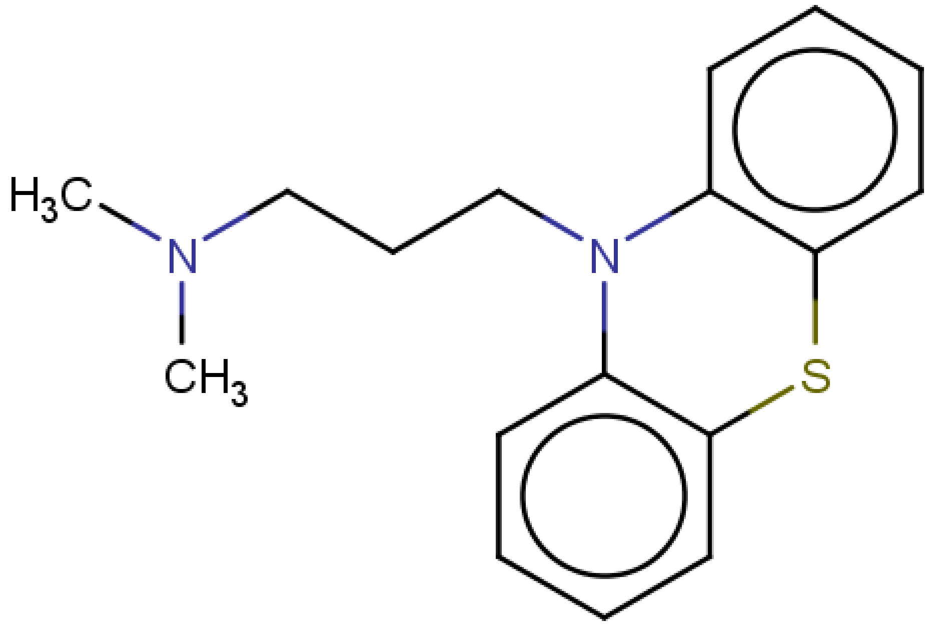

53-60-1
Name: Promazine hydrochloride
pIC50: 5.02
Rank: 264
Classes: No defined

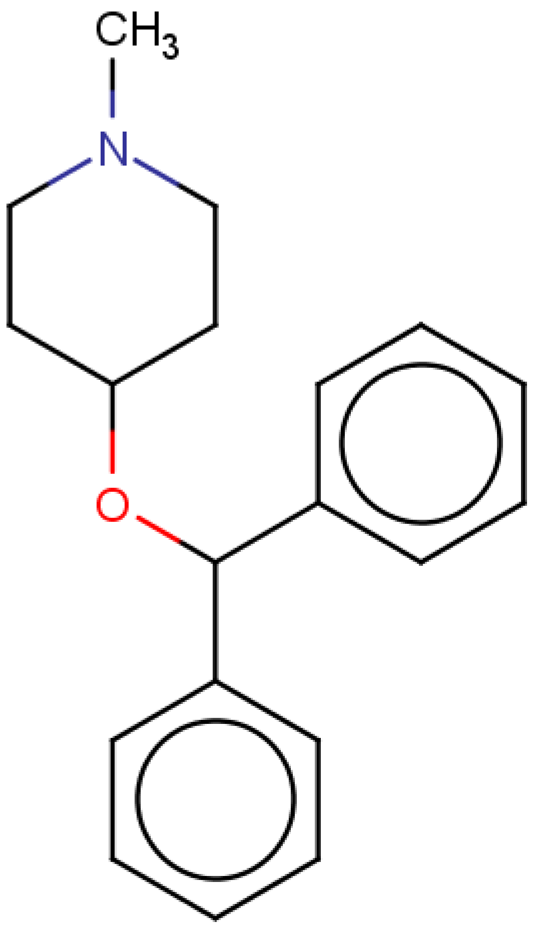

132-18-3
Name: Diphenylpyraline hydrochloride
pIC50: 5.02
Rank: 265
Classes: No defined

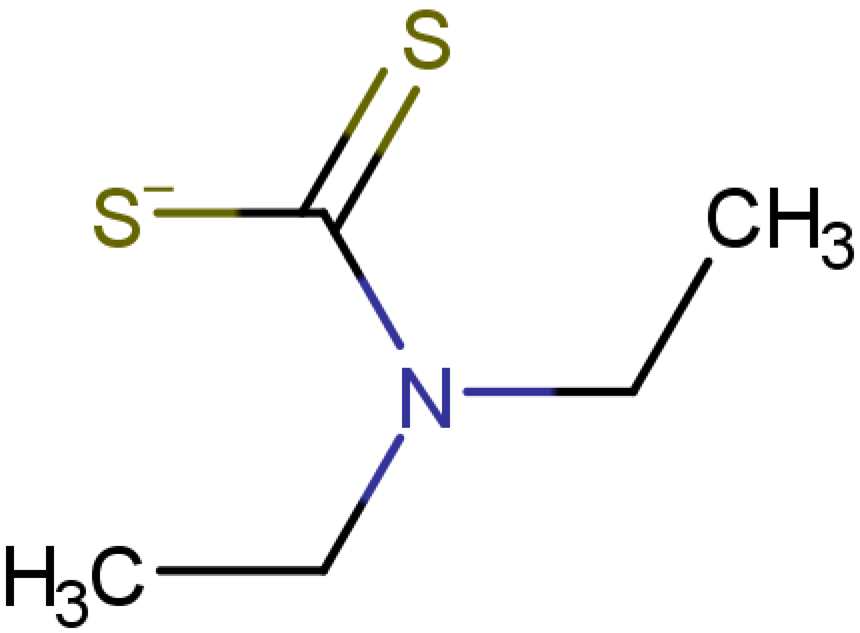

14324-55-1
Name: Zinc diethyldithiocarbamate
pIC50: 5.01
Rank: 266
Classes: catalyst

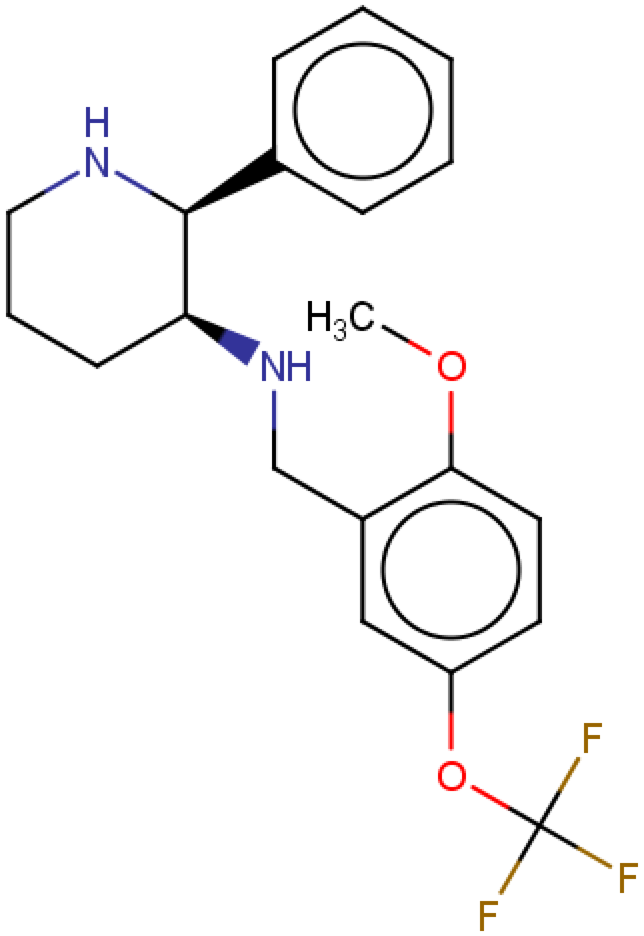

145742-28-5
Name: CP-122721
pIC50: 5.01
Rank: 267
Classes: Drug

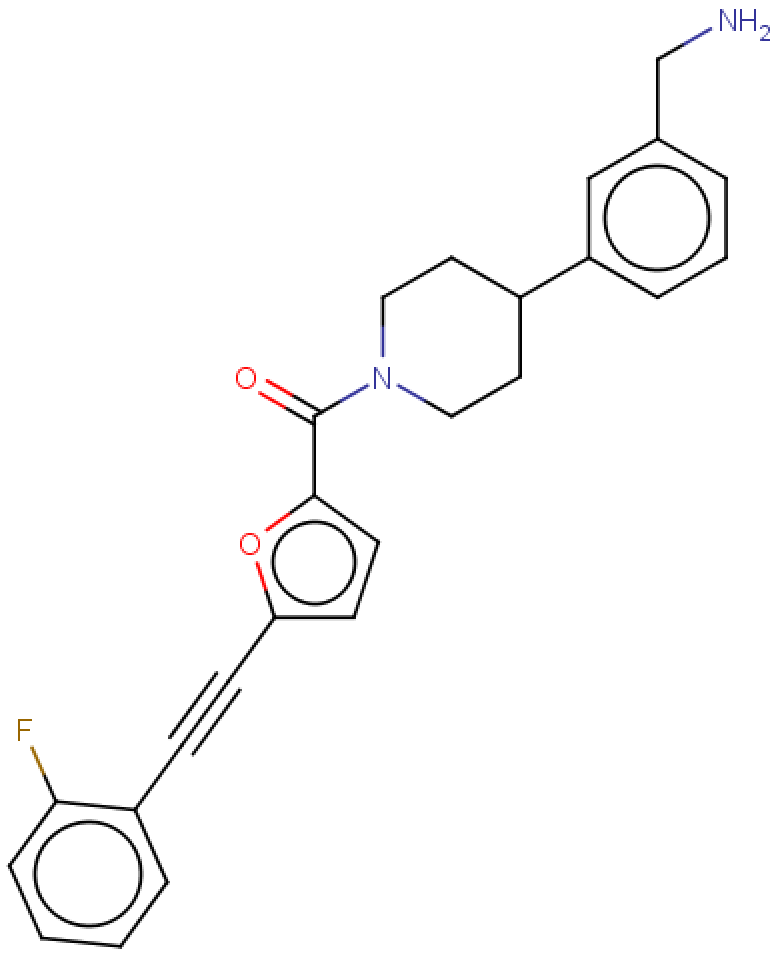

725228-45-5
Name: AVE5638
pIC50: 5.01
Rank: 268
Classes: No defined

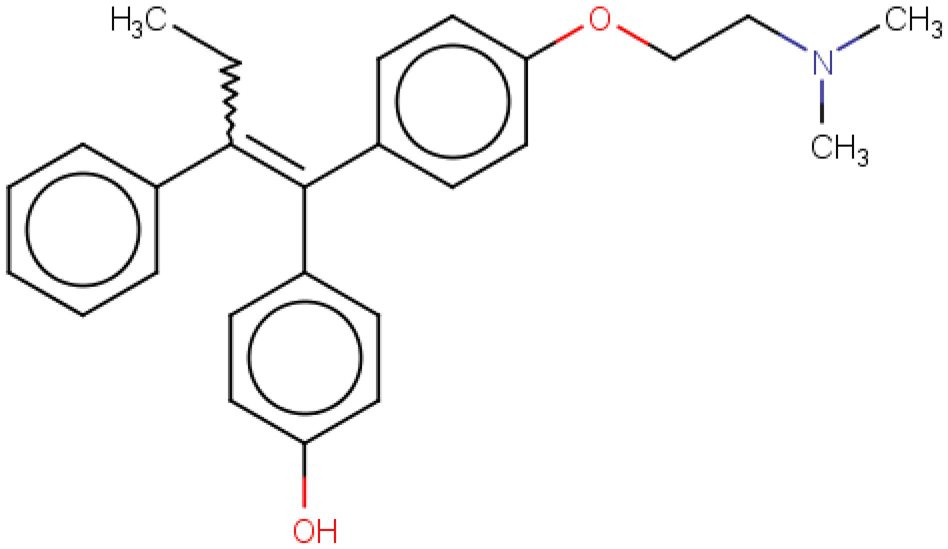

68392-35-8
Name: 4-Hydroxytamoxifen
pIC50: 5.01
Rank: 269
Classes: No defined

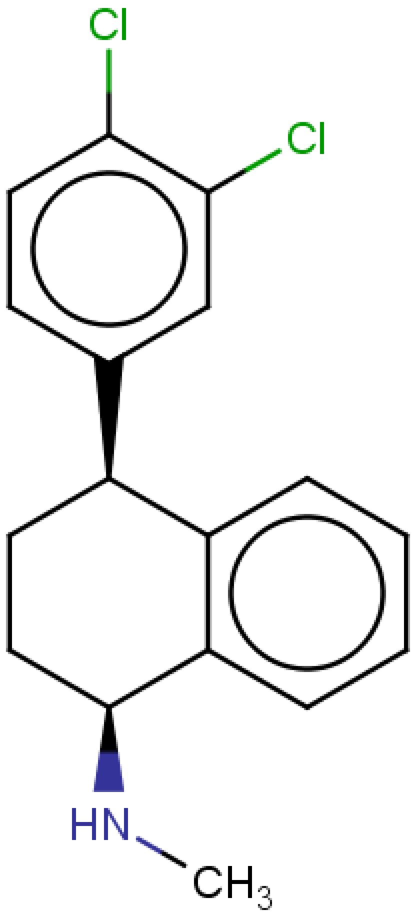

79559-97-0
Name: Sertraline hydrochloride
pIC50: 5.0
Rank: 270
Classes: No defined

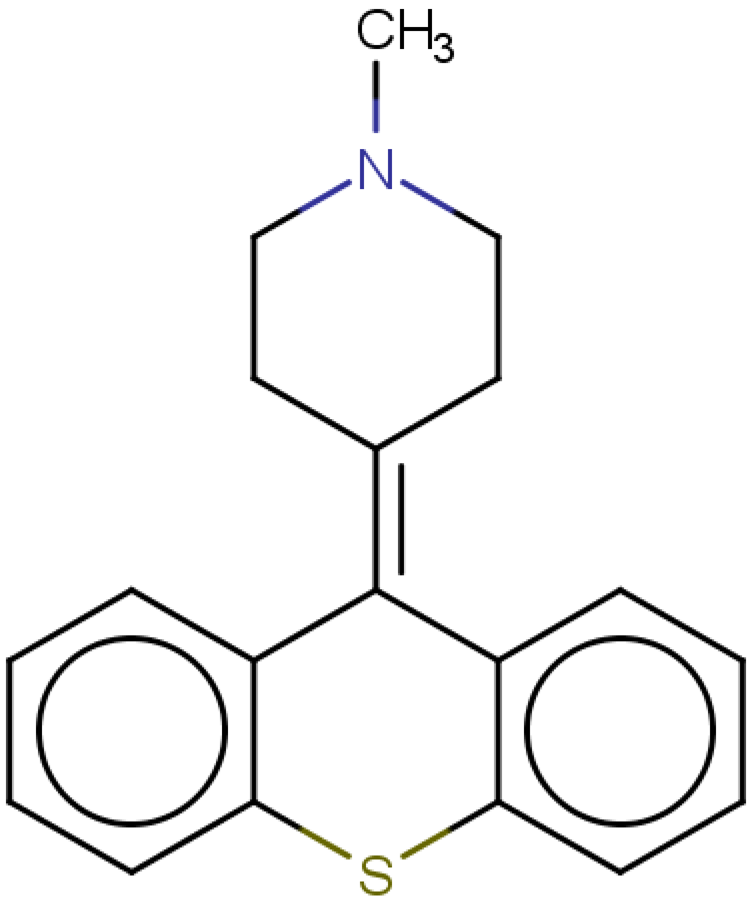

13187-06-9
Name: Pimethixene maleate
pIC50: 5.0
Rank: 271
Classes: No defined

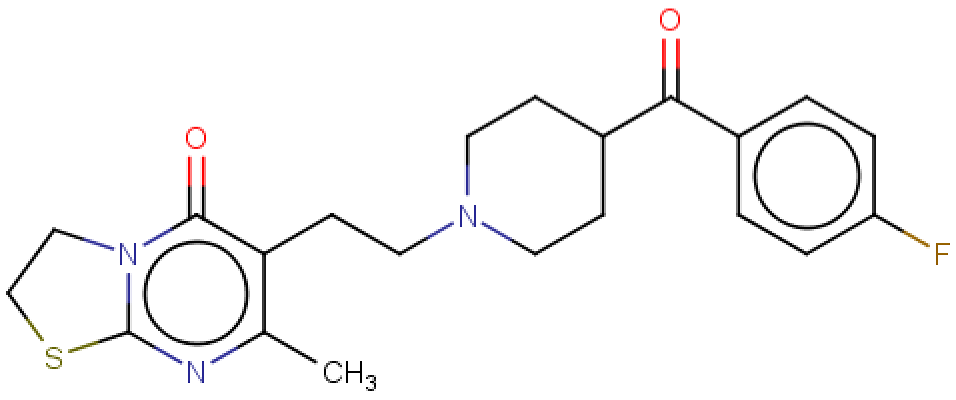

86487-64-1
Name: Setoperone
pIC50: 5.0
Rank: 272
Classes: No defined

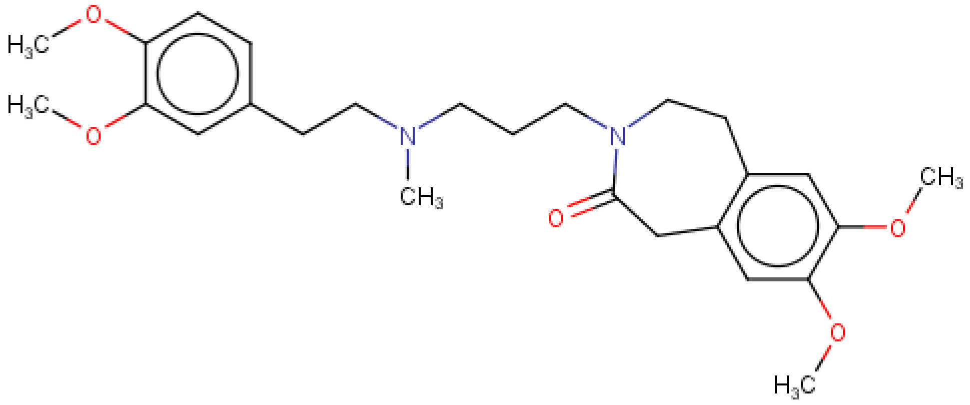

91940-87-3
Name: Zatebradine hydrochloride
pIC50: 5.0
Rank: 273
Classes: No defined

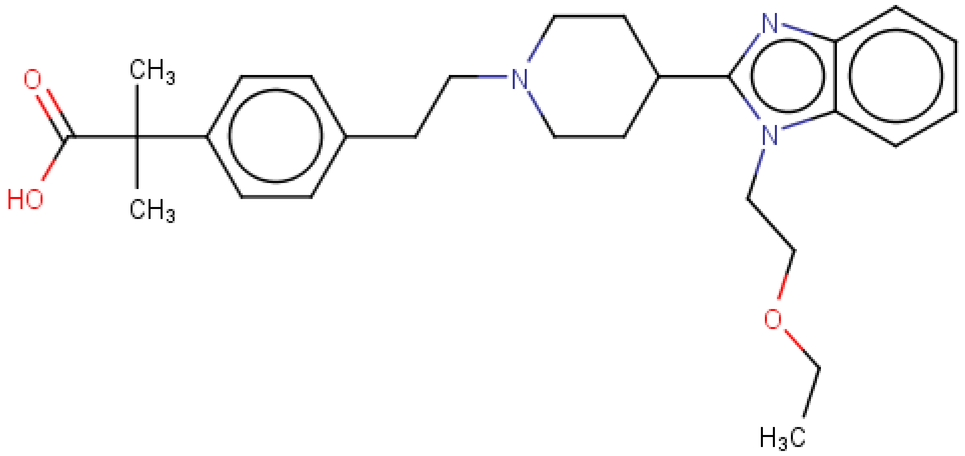

202189-78-4
Name: Bilastine
pIC50: 5.0
Rank: 274
Classes: Drug

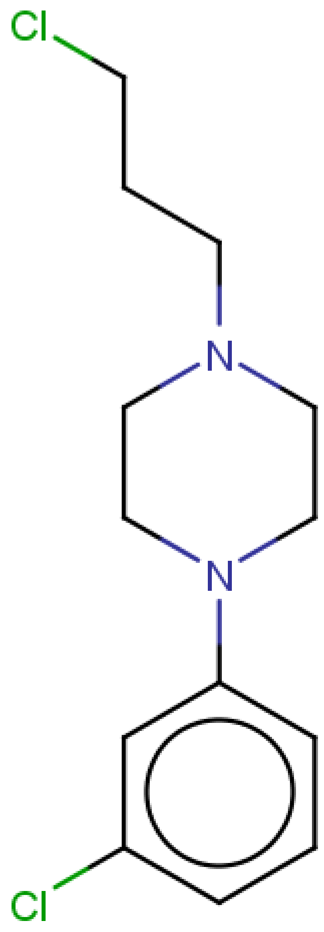

52605-52-4
Name: 1-(3-Chlorophenyl)-4-(3-chloropropyl)piperazine hydrochloride
pIC50: 5.0
Rank: 275
Classes: No defined

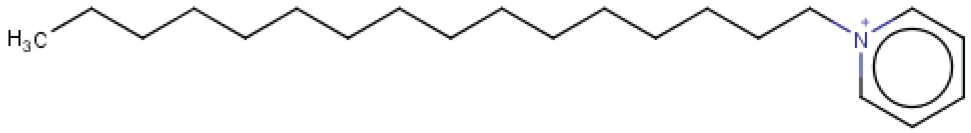

123-03-5
Name: Cetylpyridinium chloride
pIC50: 4.99
Rank: 276
Classes: antimicrobial--NA--TSCA

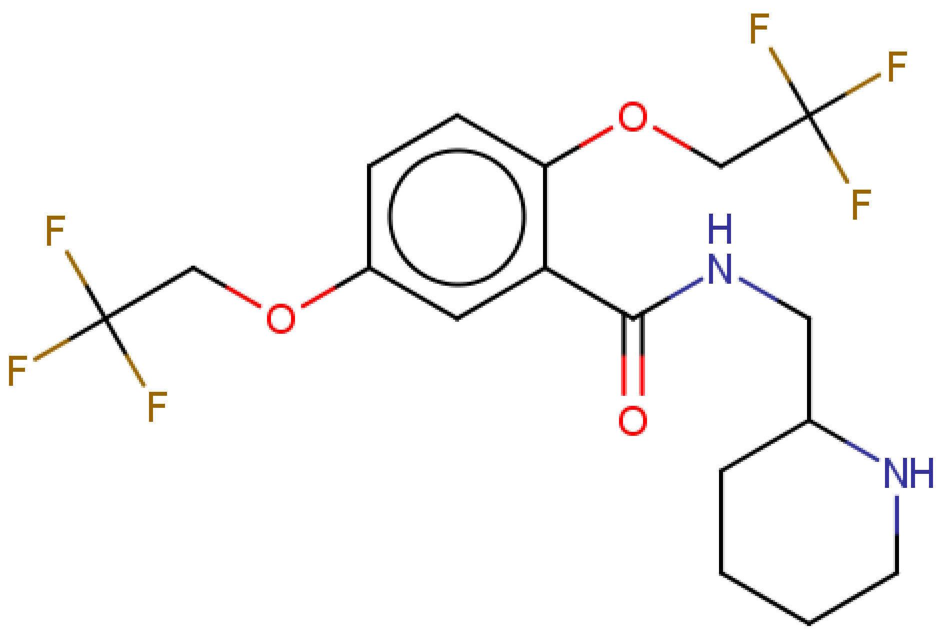

54143-55-4
Name: Flecainide
pIC50: 4.99
Rank: 277
Classes: Drug

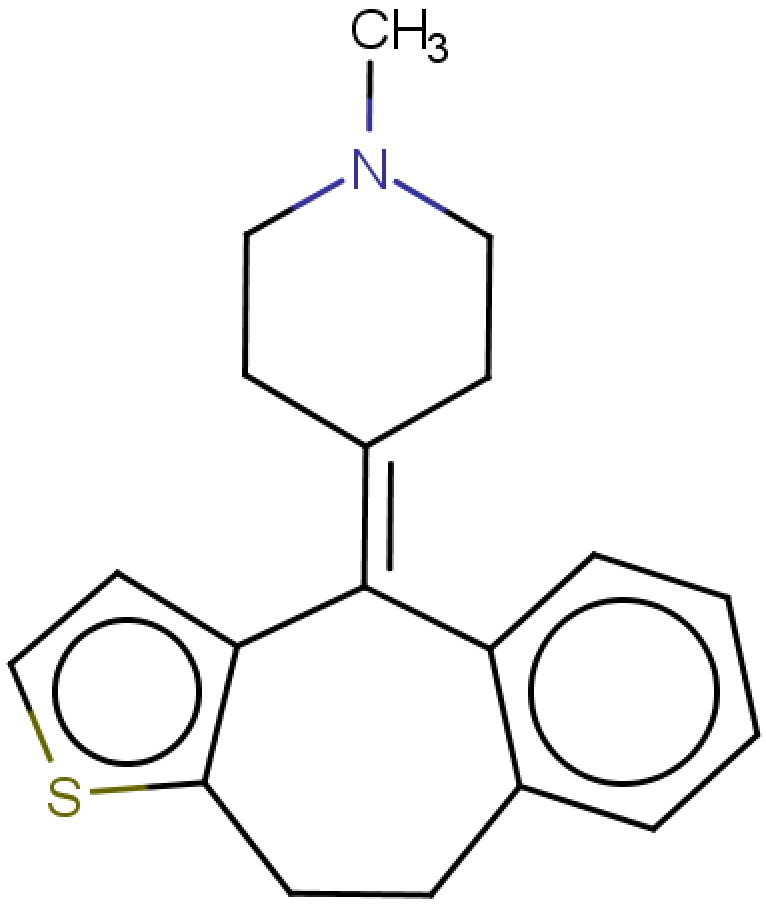

15574-96-6
Name: Pizotyline
pIC50: 4.99
Rank: 278
Classes: No defined

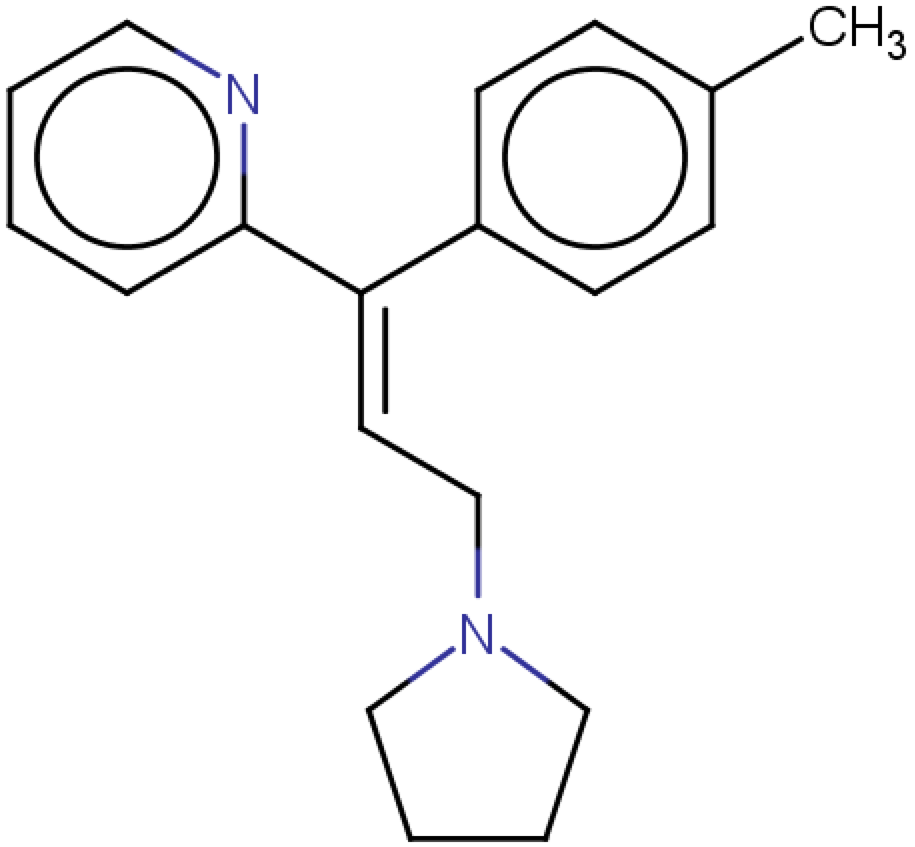

486-12-4
Name: Triprolidine
pIC50: 4.99
Rank: 279
Classes: Drug

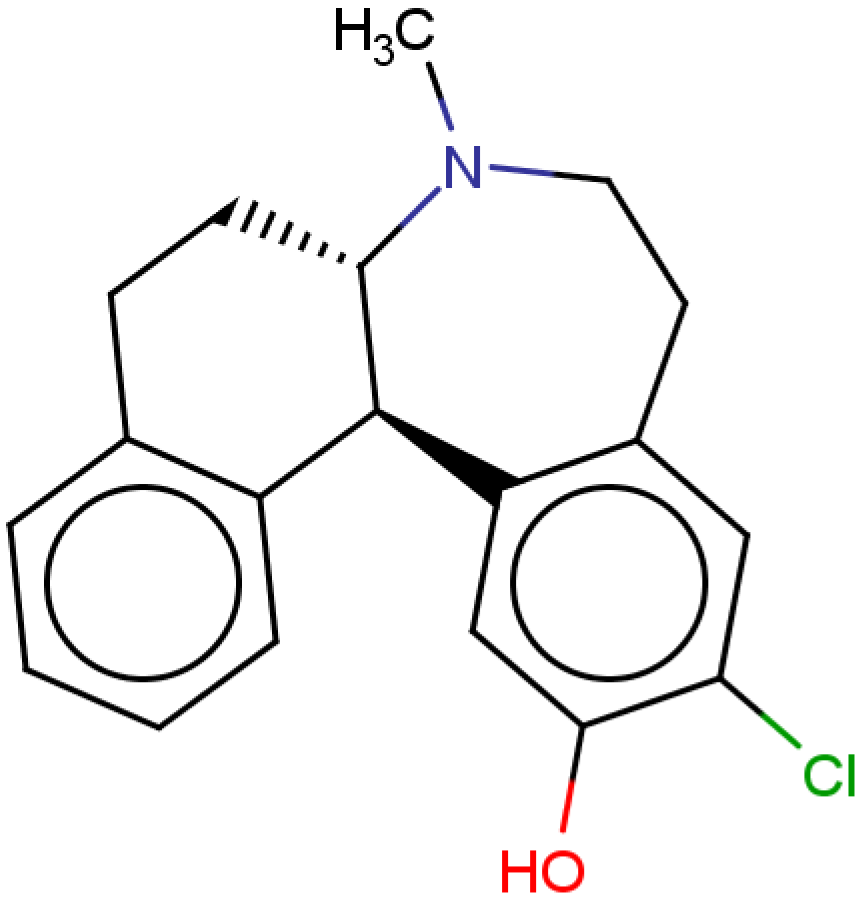

112108-01-7
Name: Ecopipam
pIC50: 4.99
Rank: 280
Classes: Drug

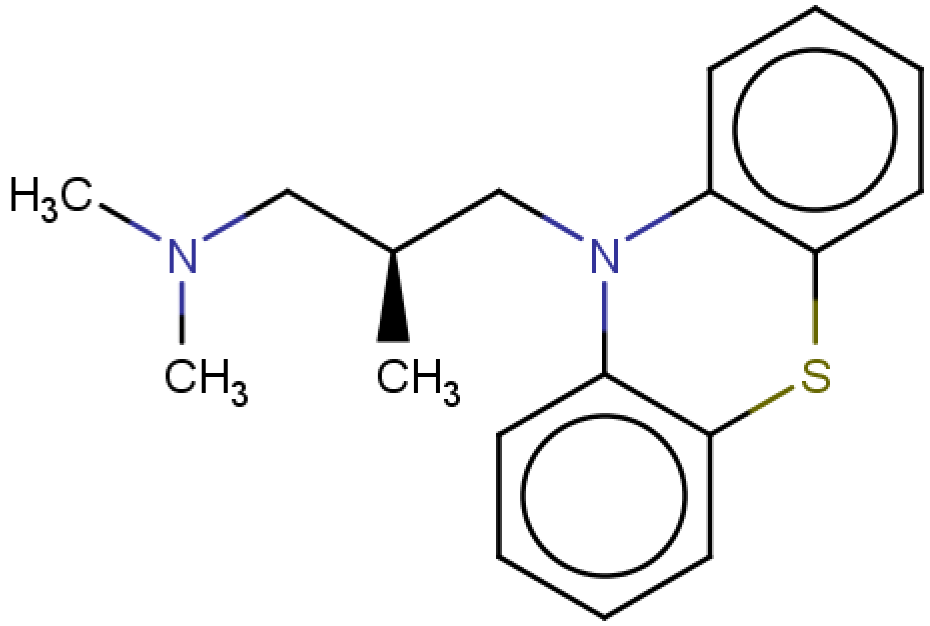

4330-99-8
Name: Trimeprazine tartrate
pIC50: 4.99
Rank: 281
Classes: No defined

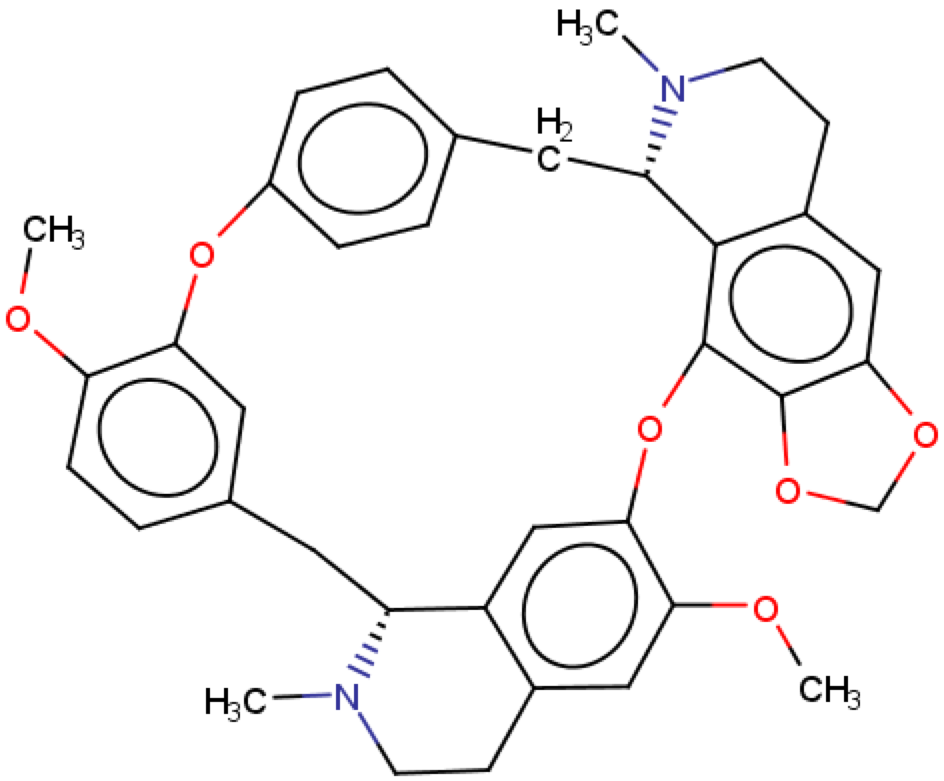

481-49-2
Name: Cepharanthine
pIC50: 4.99
Rank: 282
Classes: No defined

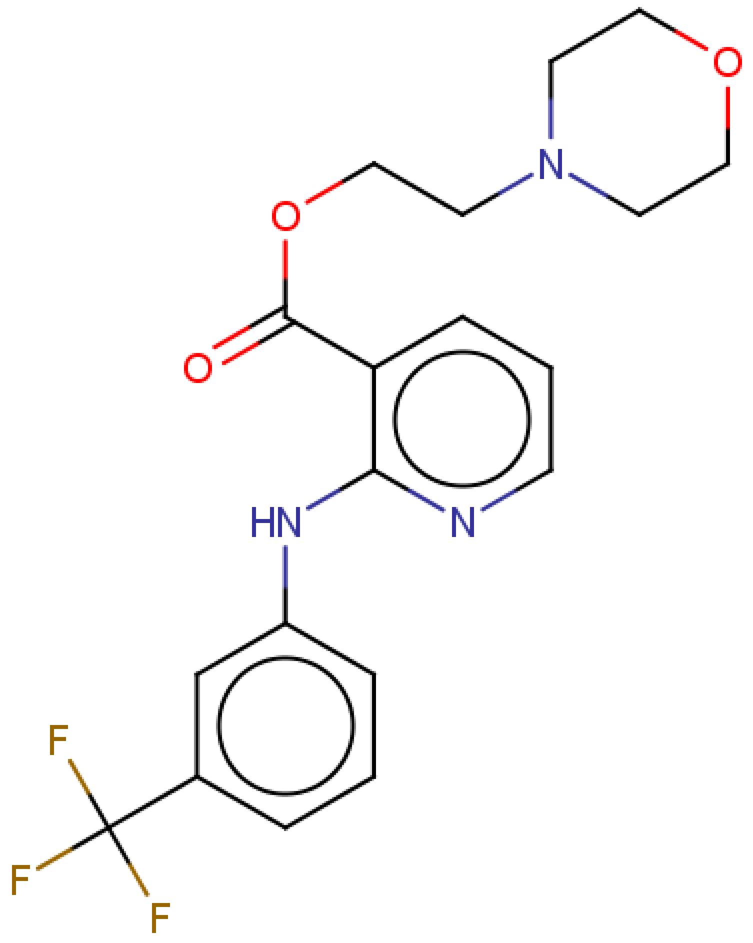

65847-85-0
Name: Morniflumate
pIC50: 4.99
Rank: 283
Classes: Drug

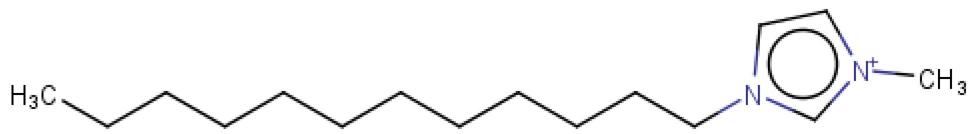

81995-09-7
Name: 1-Dodecyl-3-methylimidazolium iodide
pIC50: 4.97
Rank: 284
Classes: No defined

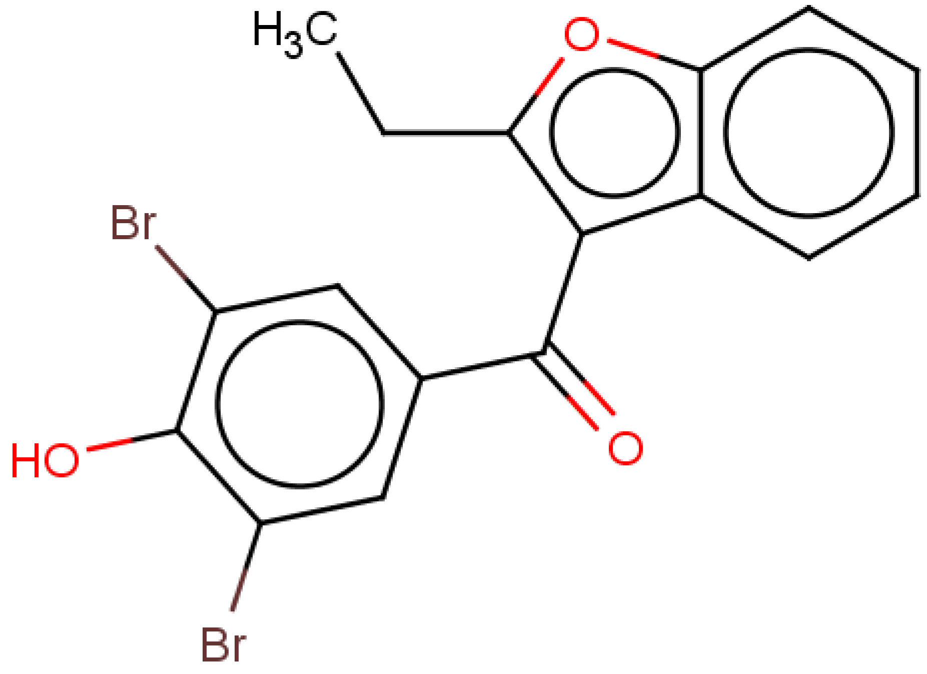

3562-84-3
Name: Benzbromarone
pIC50: 4.97
Rank: 285
Classes: Drug

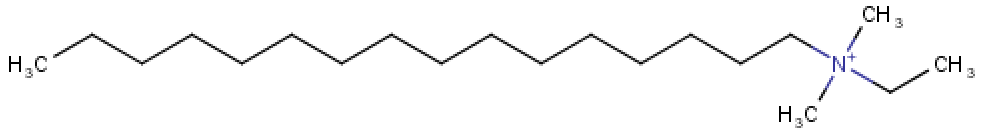

124-03-8
Name: Ethylhexadecyldimethylammonium bromide
pIC50: 4.97
Rank: 286
Classes: antimicrobial

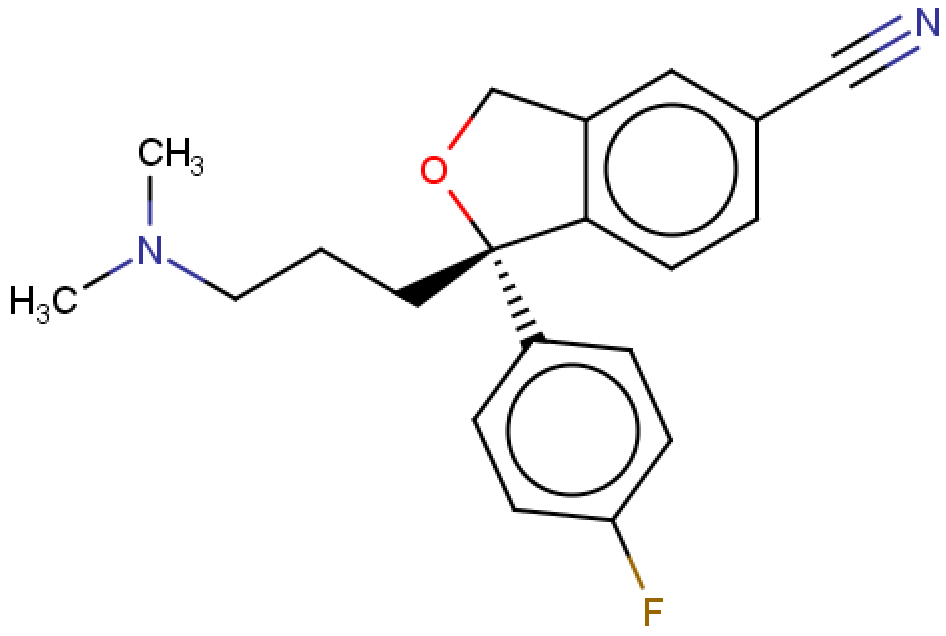

219861-08-2
Name: Escitalopram oxalate
pIC50: 4.97
Rank: 287
Classes: No defined

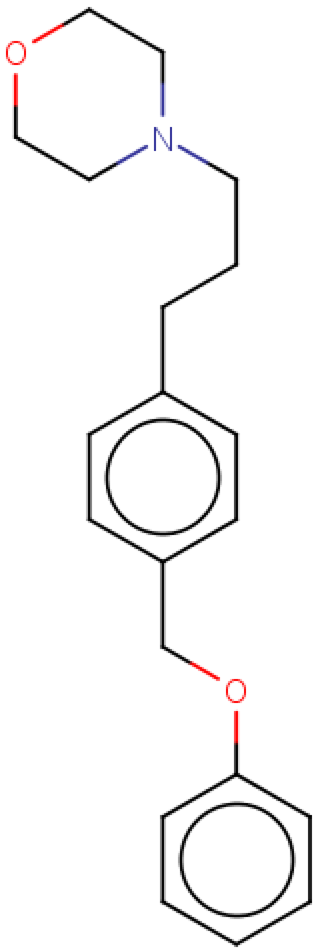

17692-39-6
Name: Fomocaine
pIC50: 4.97
Rank: 288
Classes: No defined

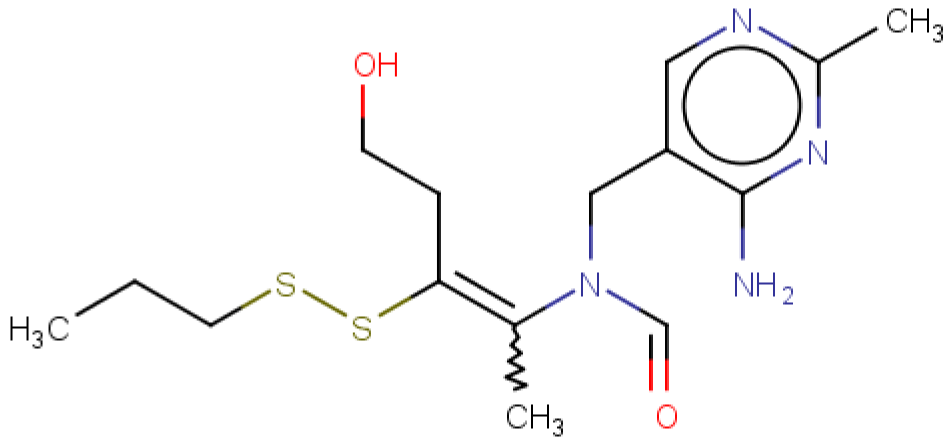

59-58-5
Name: Prosultiamine
pIC50: 4.97
Rank: 289
Classes: No defined

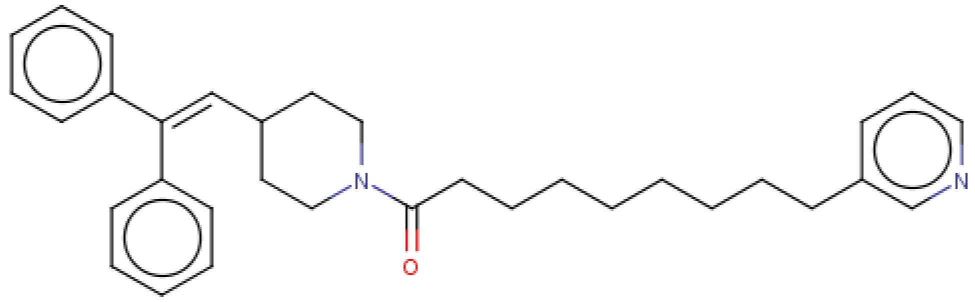

107071-66-9
Name: Ro 23-7637
pIC50: 4.96
Rank: 290
Classes: No defined

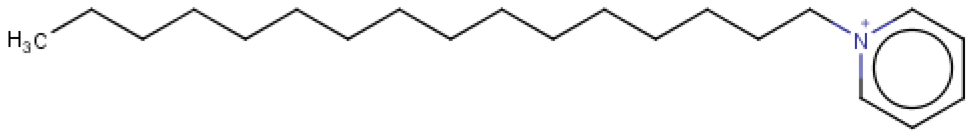

140-72-7
Name: Cetylpyridinium bromide
pIC50: 4.96
Rank: 291
Classes: TSCA

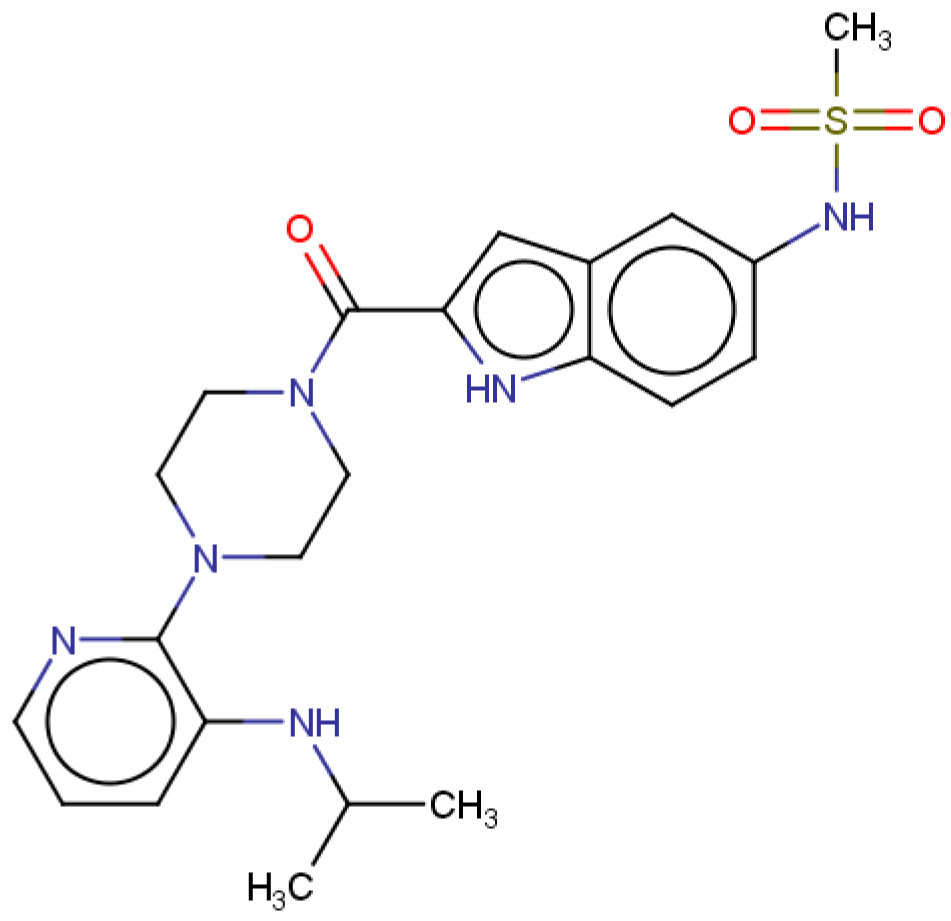

136817-59-9
Name: Delavirdine
pIC50: 4.95
Rank: 292
Classes: Drug

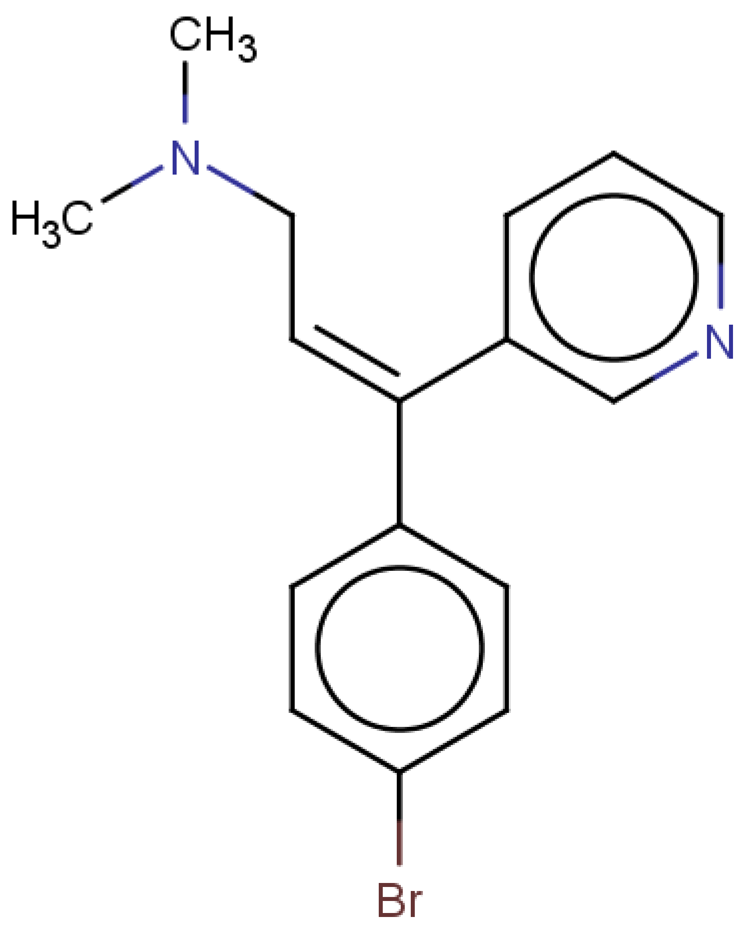

61129-30-4
Name: Zimeldine dihydrochloride hydrate
pIC50: 4.95
Rank: 293
Classes: No defined

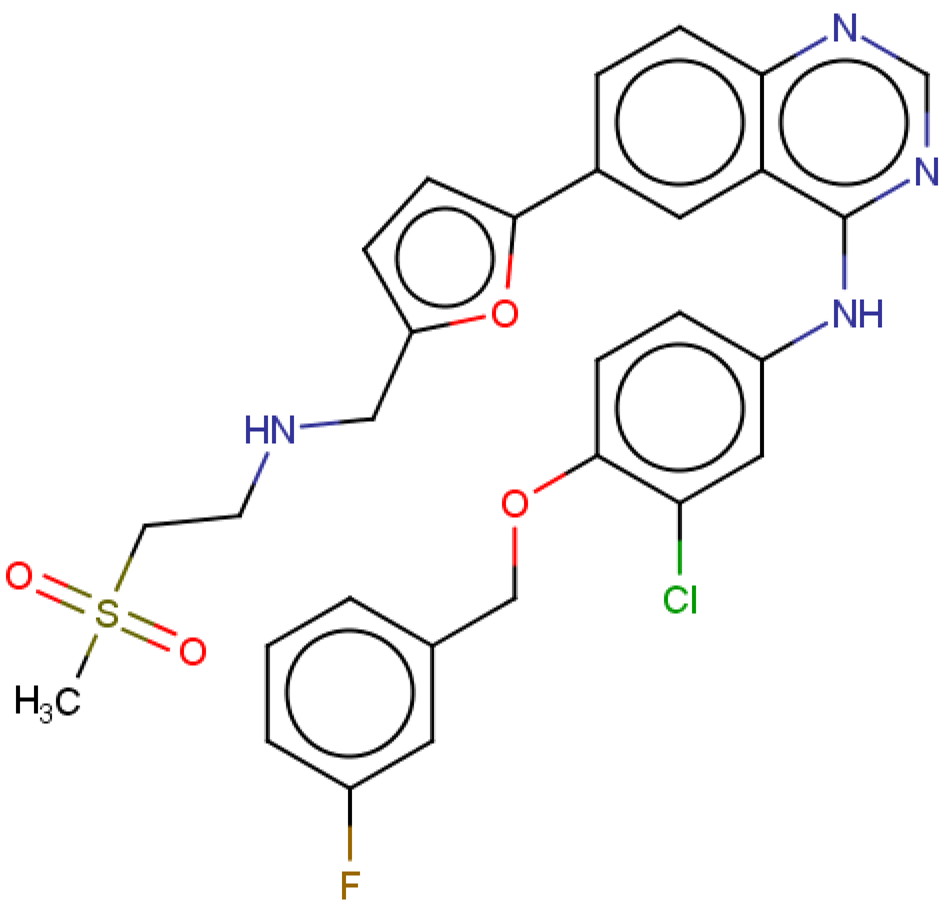

231277-92-2
Name: Lapatinib
pIC50: 4.94
Rank: 294
Classes: Drug

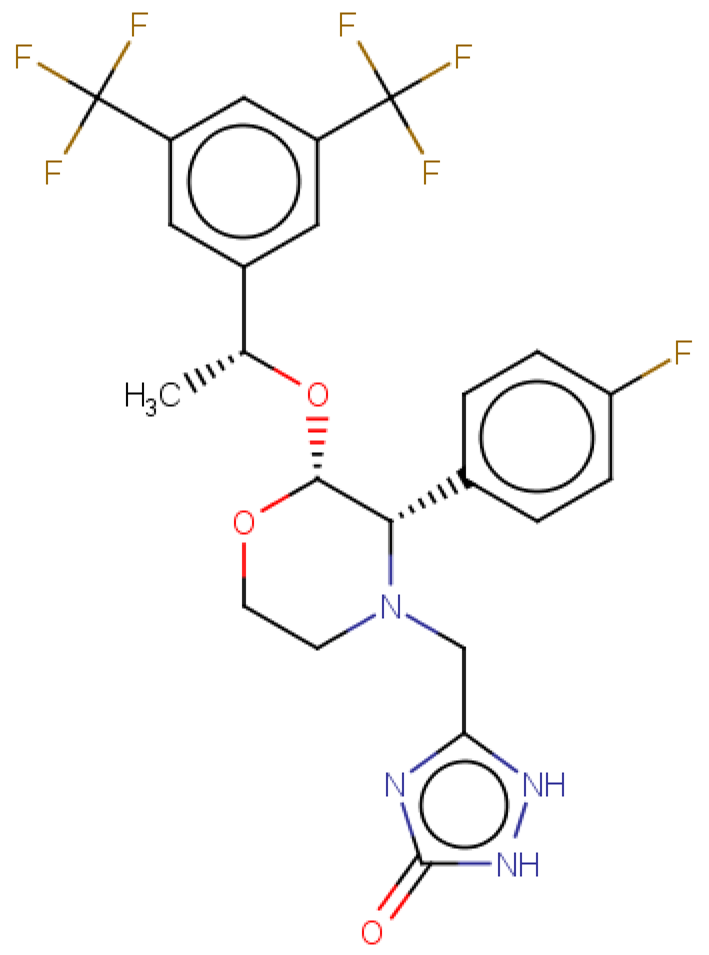

170729-80-3
Name: Aprepitant
pIC50: 4.94
Rank: 295
Classes: Drug

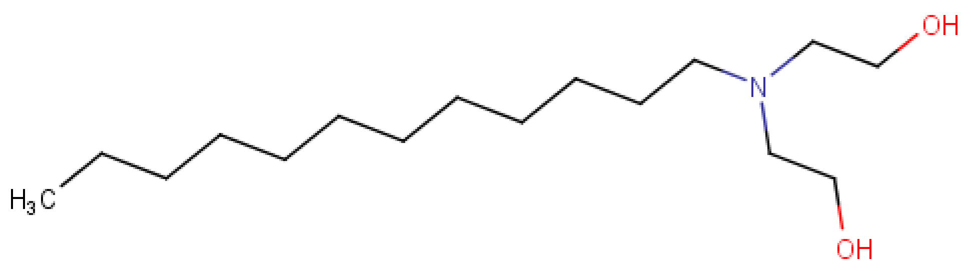

1541-67-9
Name: Lauryldiethanolamine
pIC50: 4.93
Rank: 296
Classes: hair conditioner--TSCA

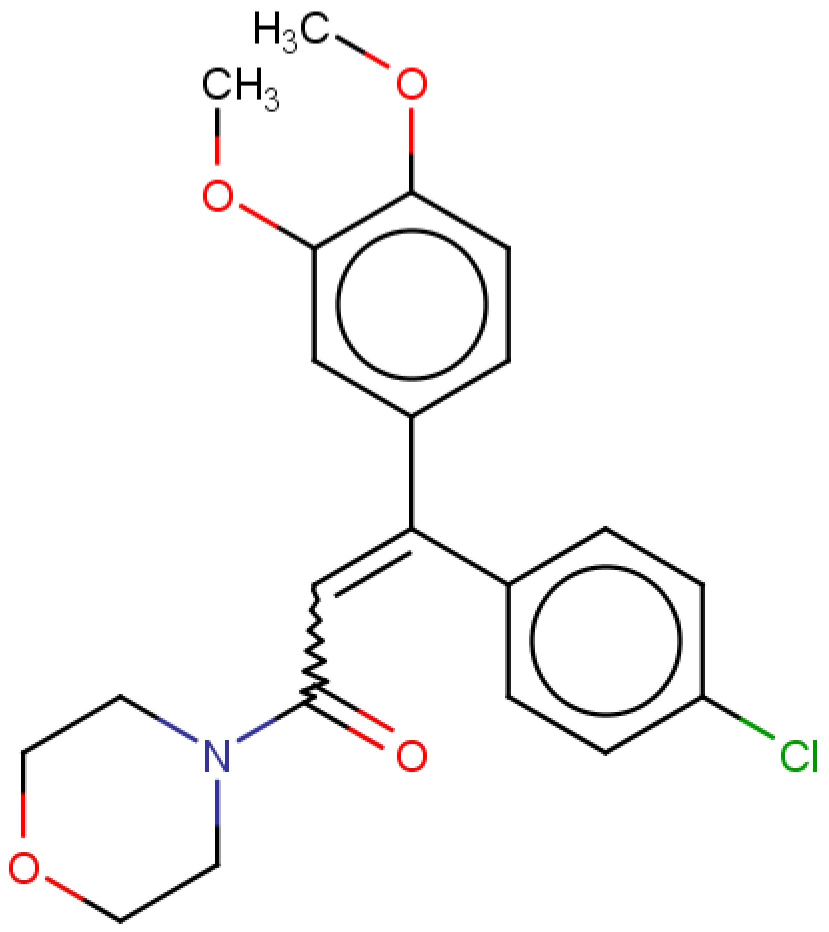

110488-70-5
Name: Dimethomorph
pIC50: 4.93
Rank: 297
Classes: Pesticide

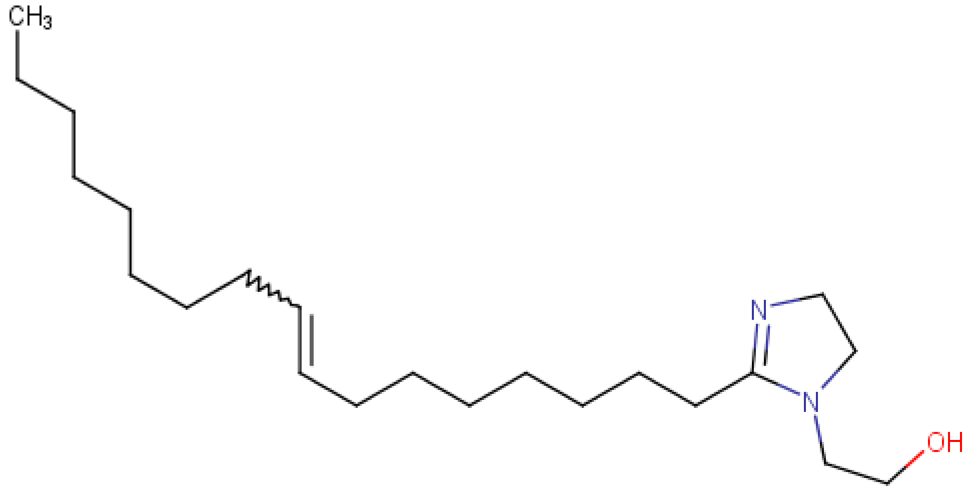

95-38-5
Name: 2-(8-Heptadecenyl)-2-imidazoline-1-ethanol
pIC50: 4.93
Rank: 298
Classes: TSCA

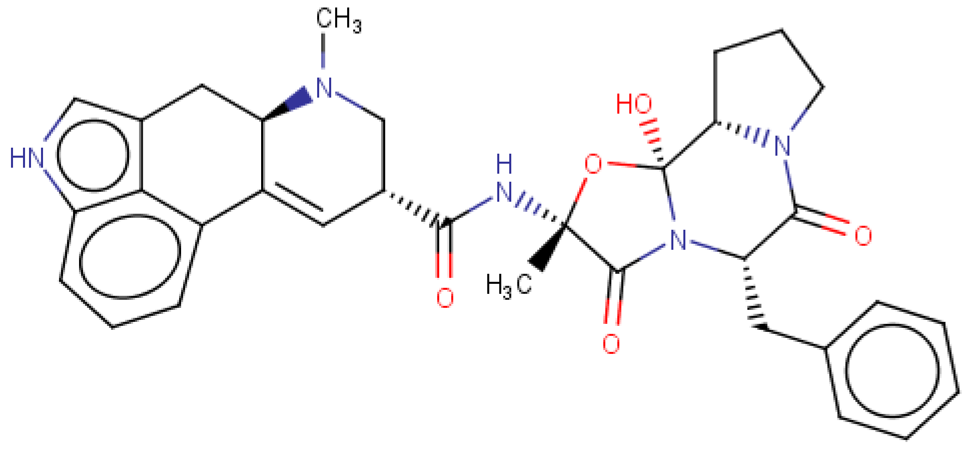

379-79-3
Name: Ergotamine D-tartrate
pIC50: 4.92
Rank: 299
Classes: No defined

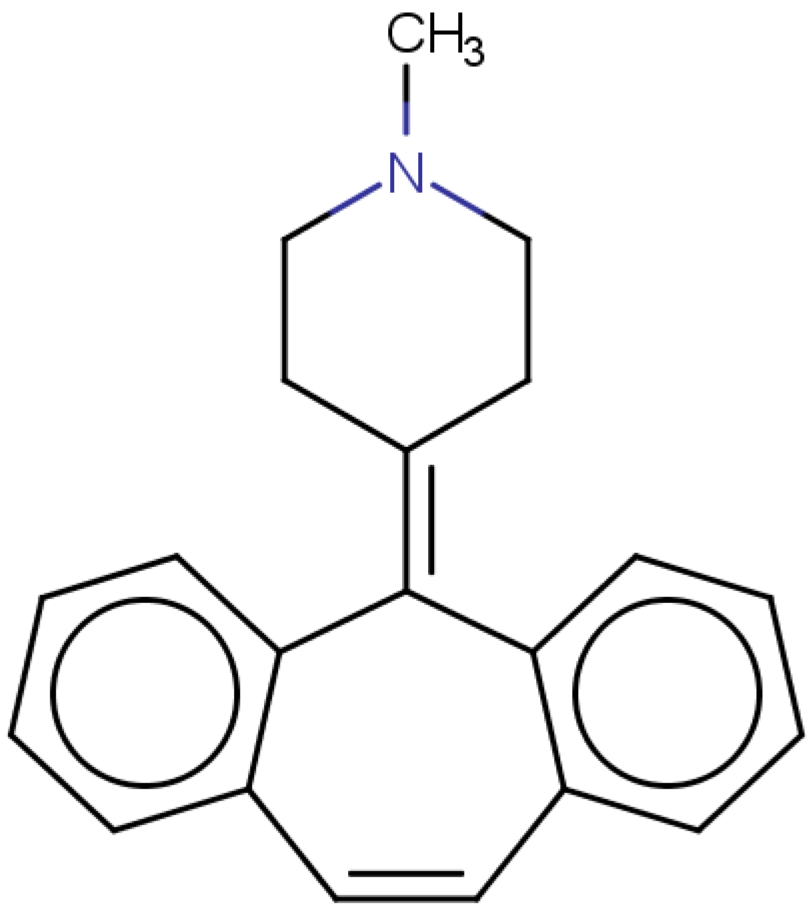

129-03-3
Name: Cyproheptadine
pIC50: 4.92
Rank: 300
Classes: Drug

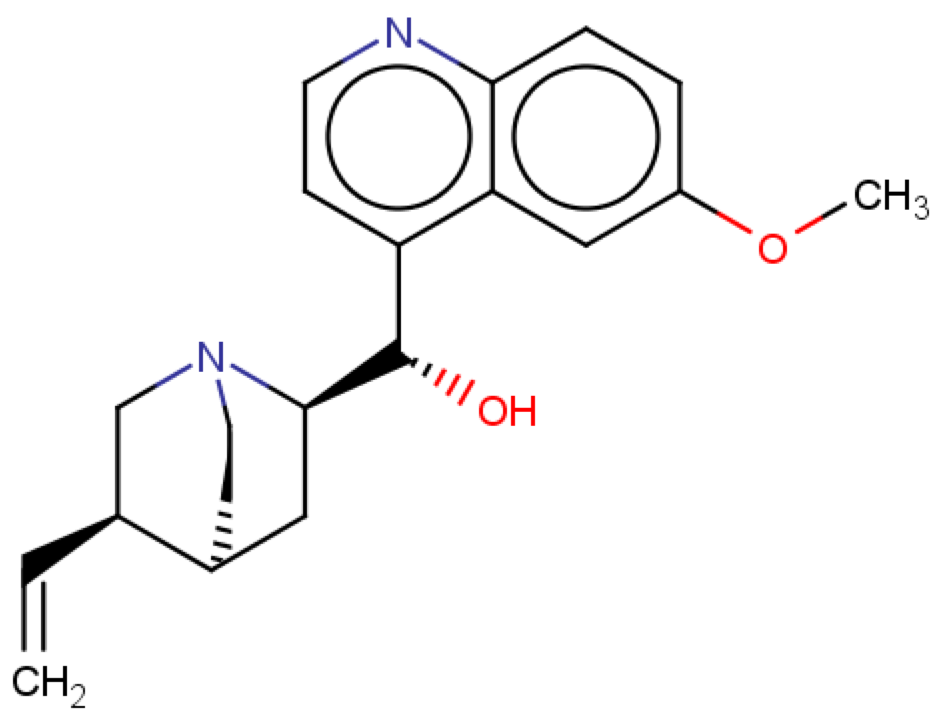

56-54-2
Name: Quinidine
pIC50: 4.92
Rank: 301
Classes: Drug

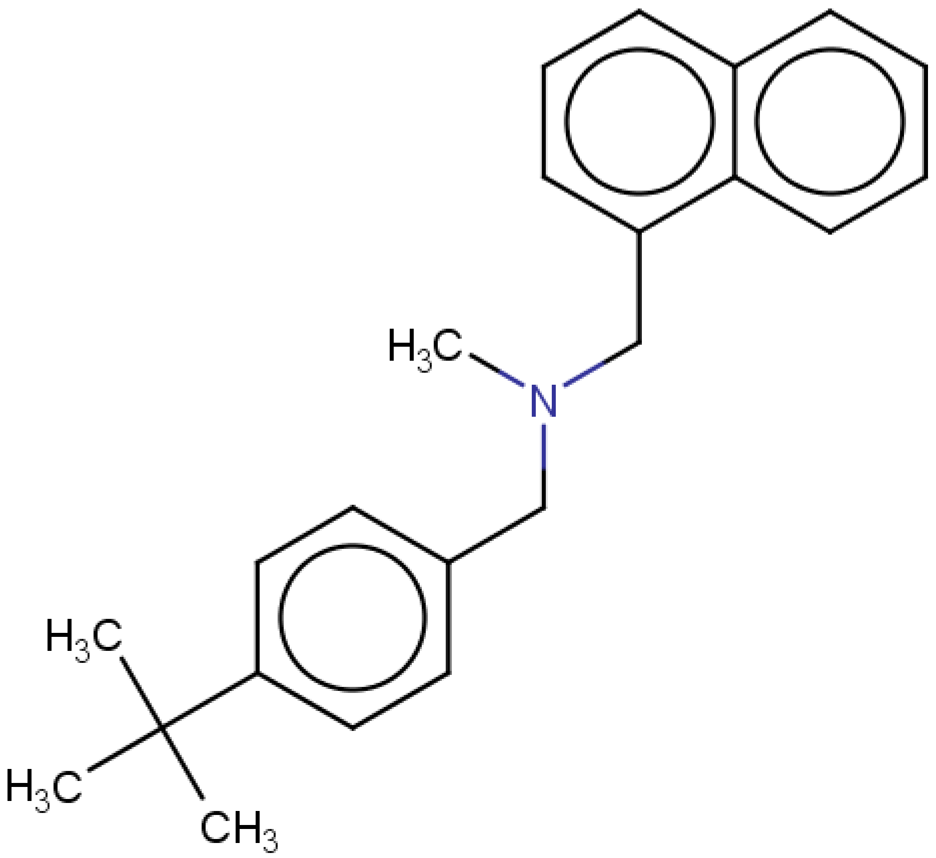

101828-21-1
Name: Butenafine
pIC50: 4.92
Rank: 302
Classes: Drug

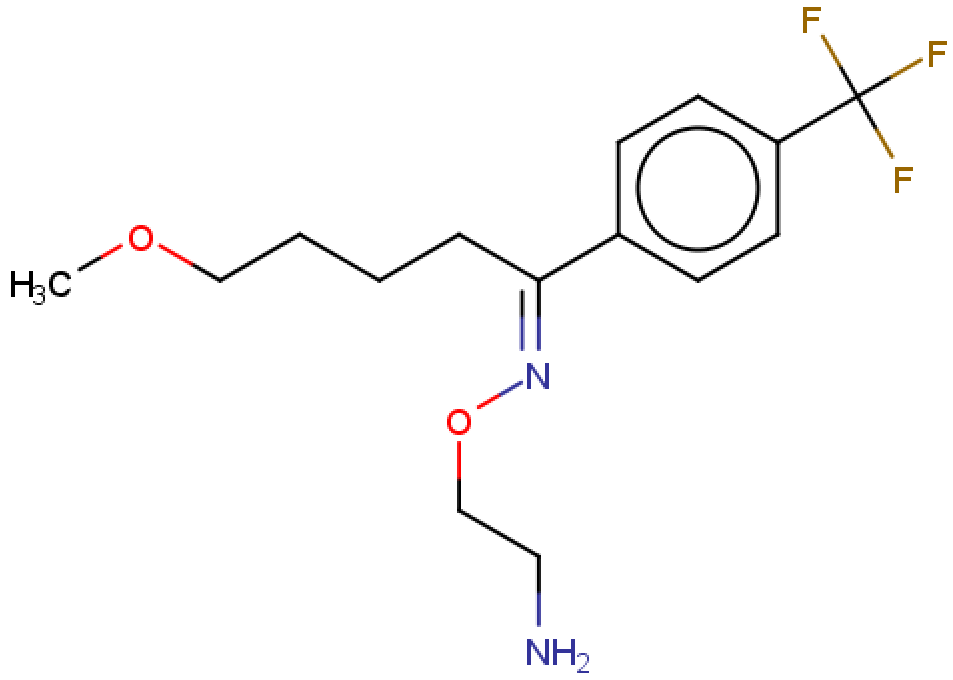

54739-18-3
Name: Fluvoxamine
pIC50: 4.92
Rank: 303
Classes: No defined

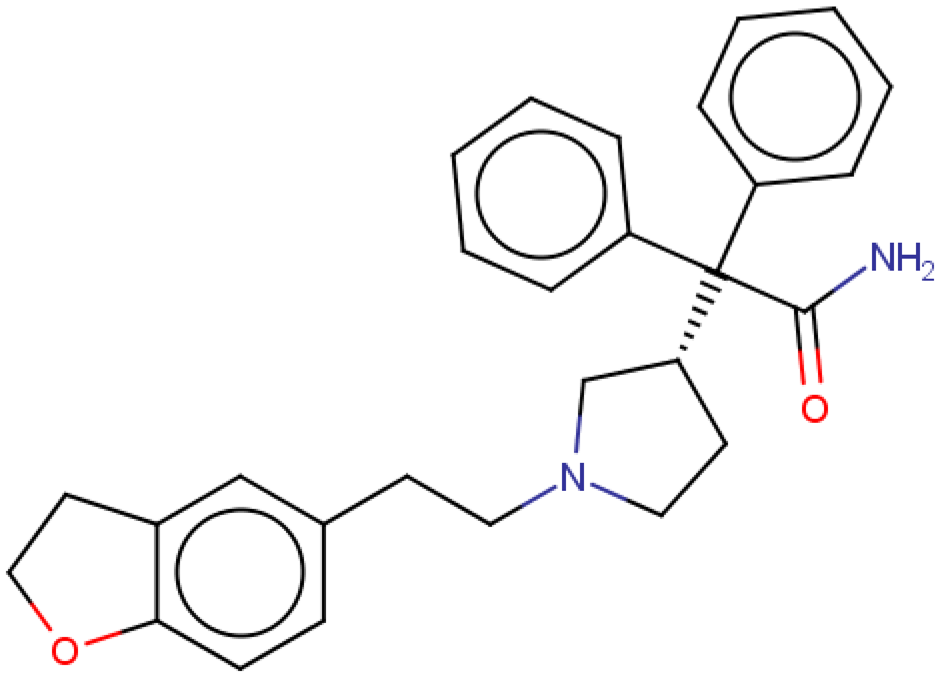

133099-07-7
Name: Darifenacin hydrobromide
pIC50: 4.92
Rank: 304
Classes: No defined

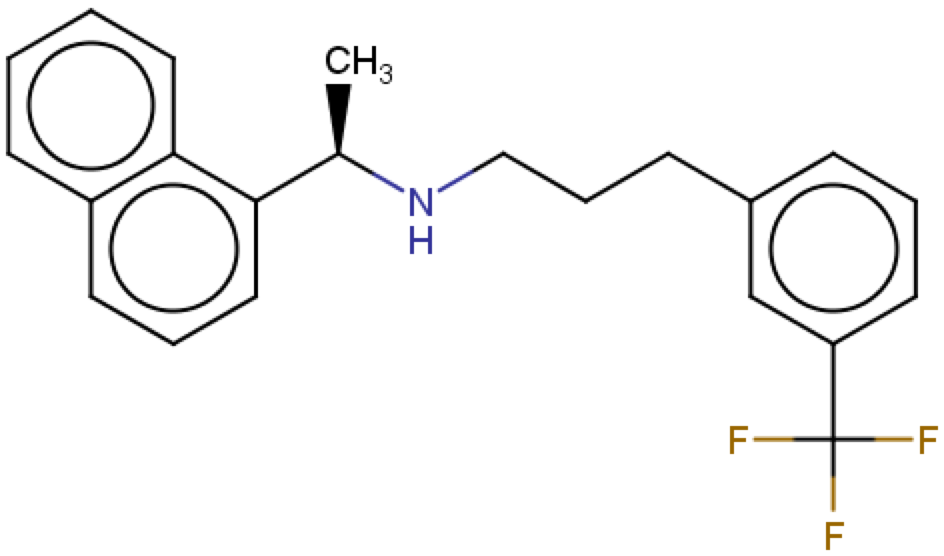

364782-34-3
Name: Cinacalcet hydrochloride
pIC50: 4.92
Rank: 305
Classes: No defined

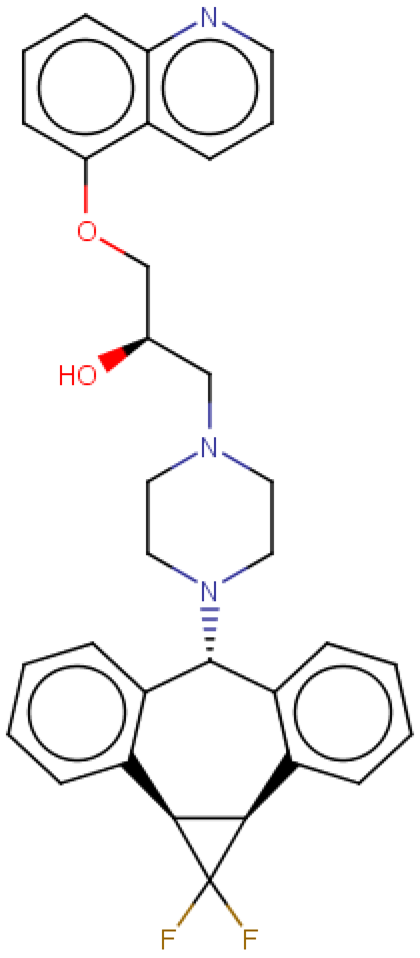

167465-36-3
Name: LY335979 trihydrochloride
pIC50: 4.92
Rank: 306
Classes: No defined

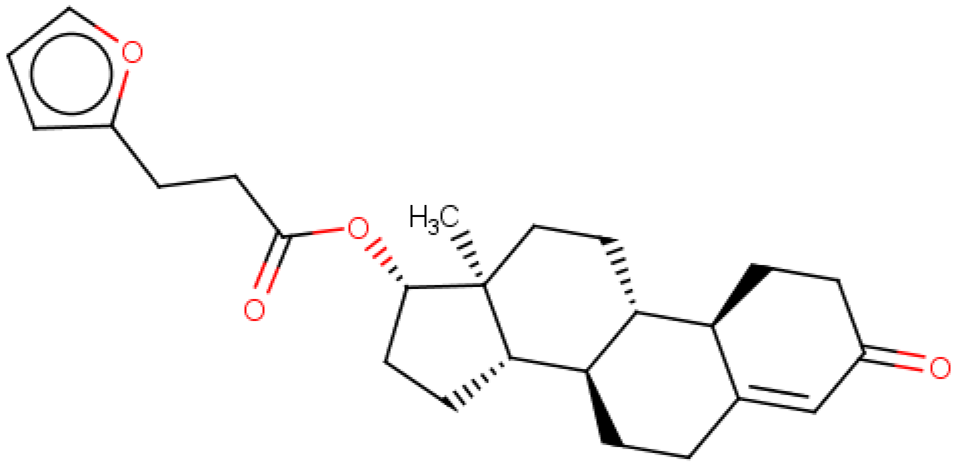

7642-64-0
Name: Nandrolone furylpropionate
pIC50: 4.92
Rank: 307
Classes: No defined

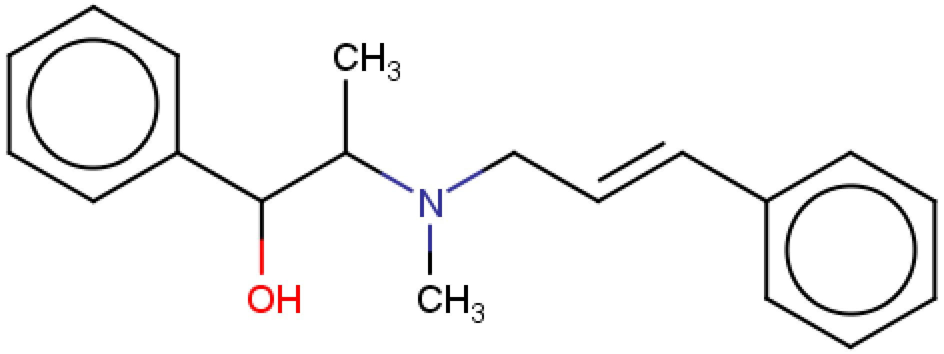

90-86-8
Name: Cinnamedrine
pIC50: 4.92
Rank: 308
Classes: No defined

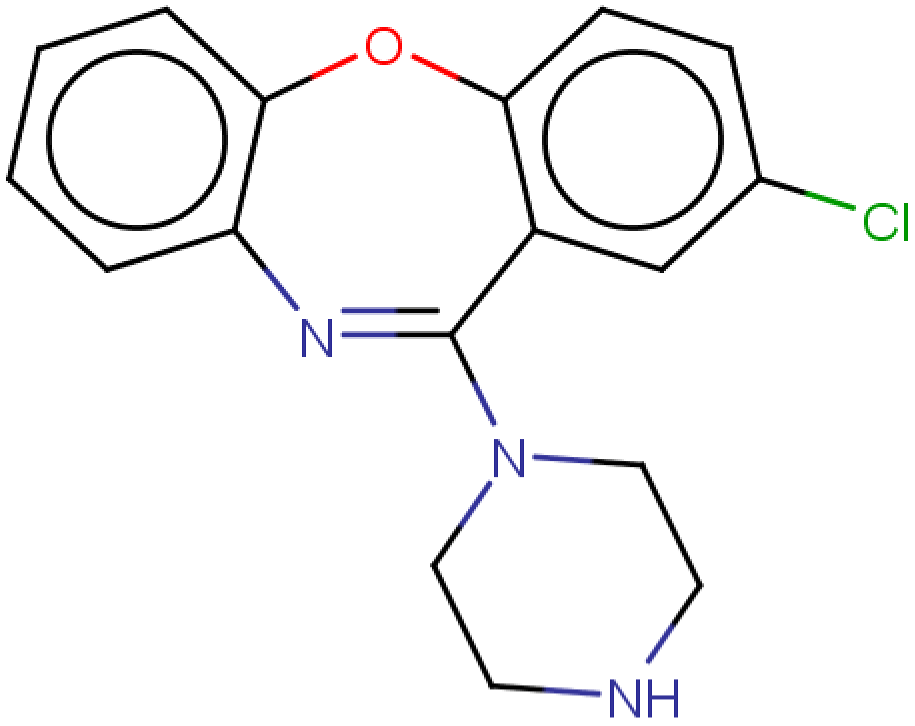

14028-44-5
Name: Amoxapine
pIC50: 4.9
Rank: 309
Classes: Drug

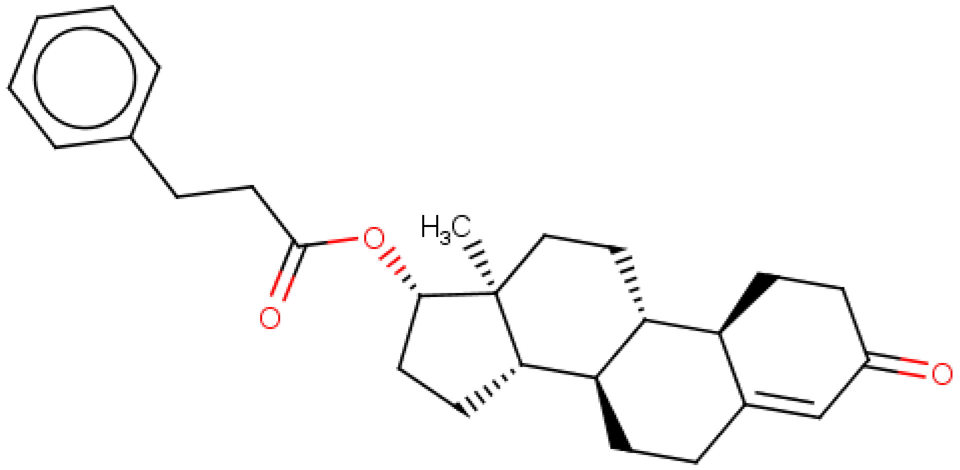

62-90-8
Name: Nandrolone phenpropionate
pIC50: 4.9
Rank: 310
Classes: Drug

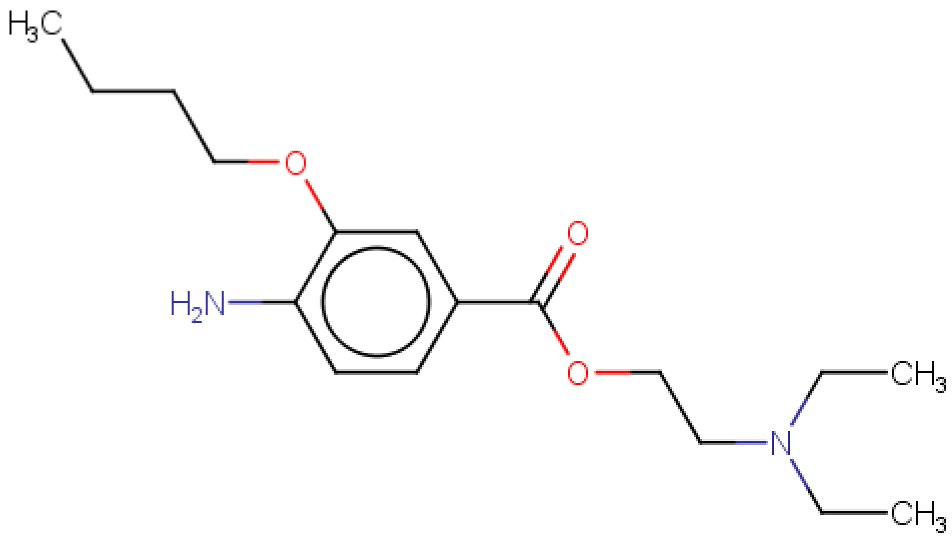

5987-82-6
Name: Benoxinate hydrochloride
pIC50: 4.9
Rank: 311
Classes: No defined

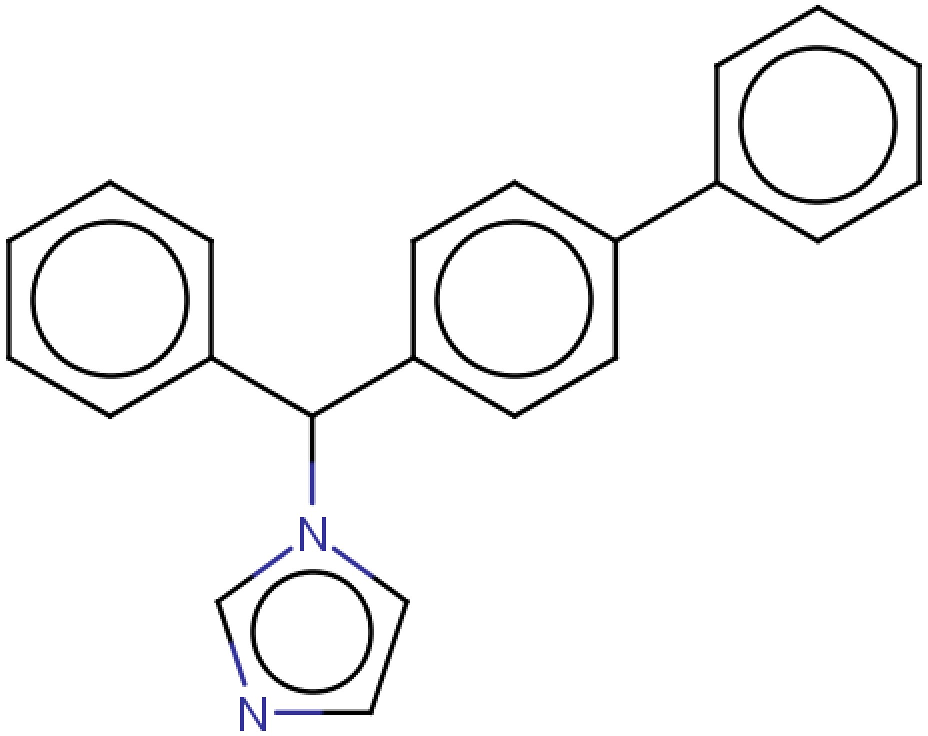

60628-96-8
Name: Bifonazole
pIC50: 4.9
Rank: 312
Classes: antimicrobial--Drug

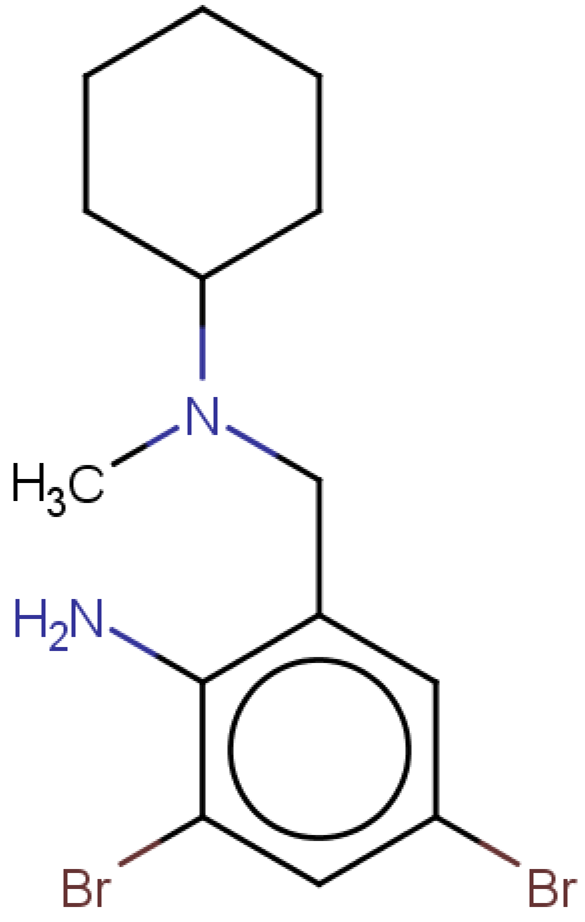

611-75-6
Name: Bromhexine hydrochloride
pIC50: 4.9
Rank: 313
Classes: No defined

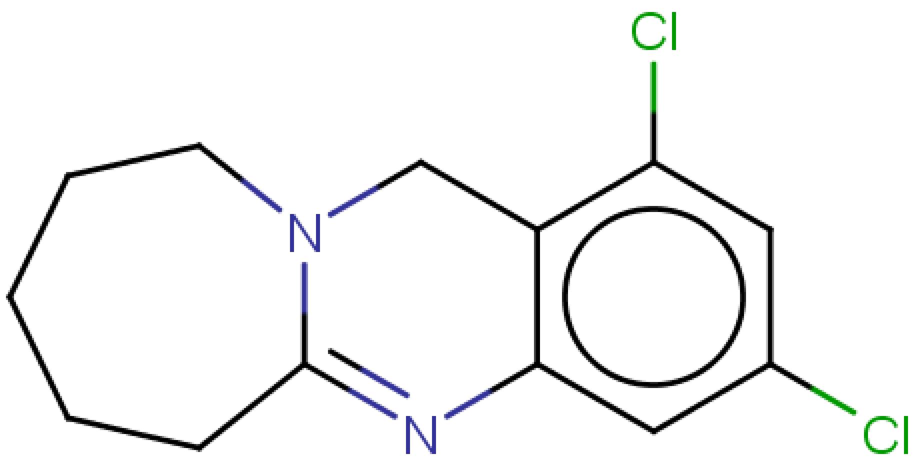

149062-75-9
Name: 1 3-Dichloro-6 7 8 9 10 12-hexahydroazepino[2 1-b]quinazoline hydrochloride (1:1)
pIC50: 4.9
Rank: 314
Classes: No defined

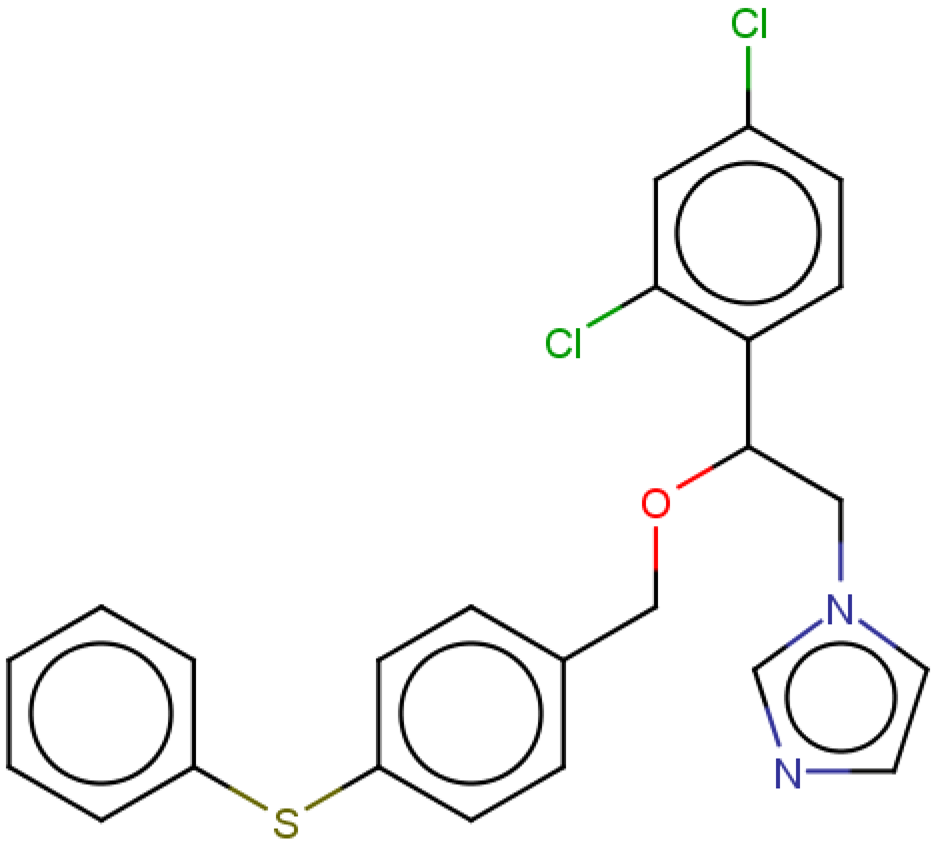

72479-26-6
Name: Fenticonazole
pIC50: 4.9
Rank: 315
Classes: No defined

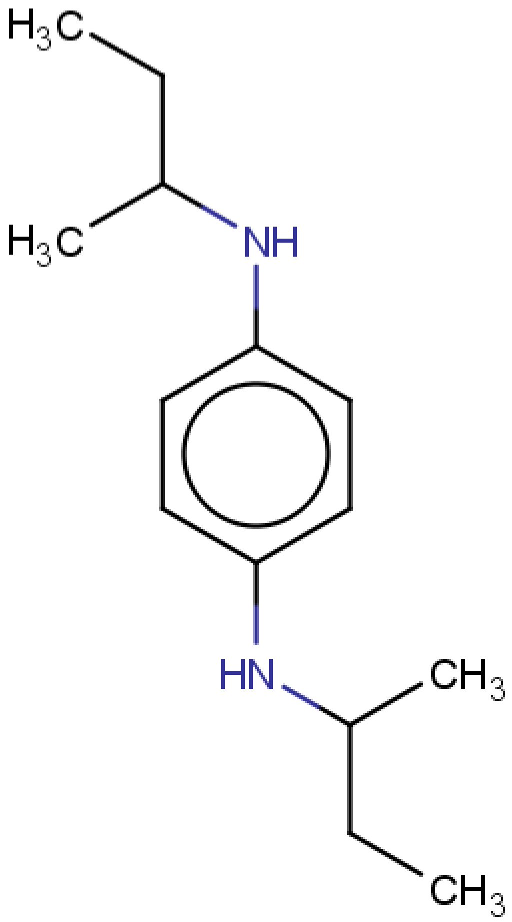

101-96-2
Name: N N'-Bis(1-methylpropyl)-1 4-benzenediamine
pIC50: 4.9
Rank: 316
Classes: No defined

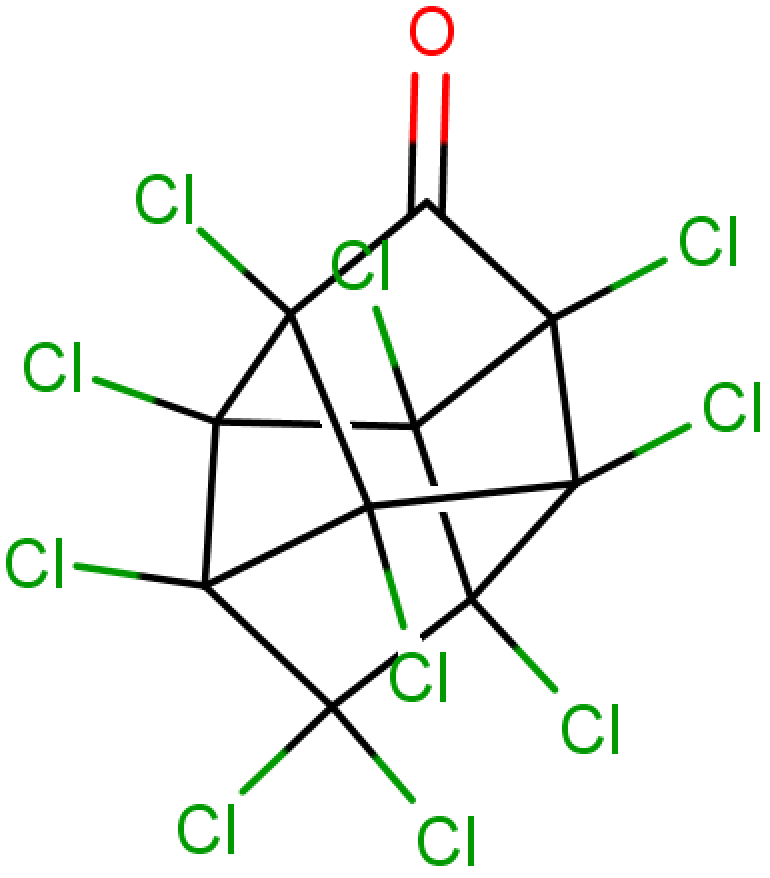

143-50-0
Name: Kepone
pIC50: 4.89
Rank: 317
Classes: No defined

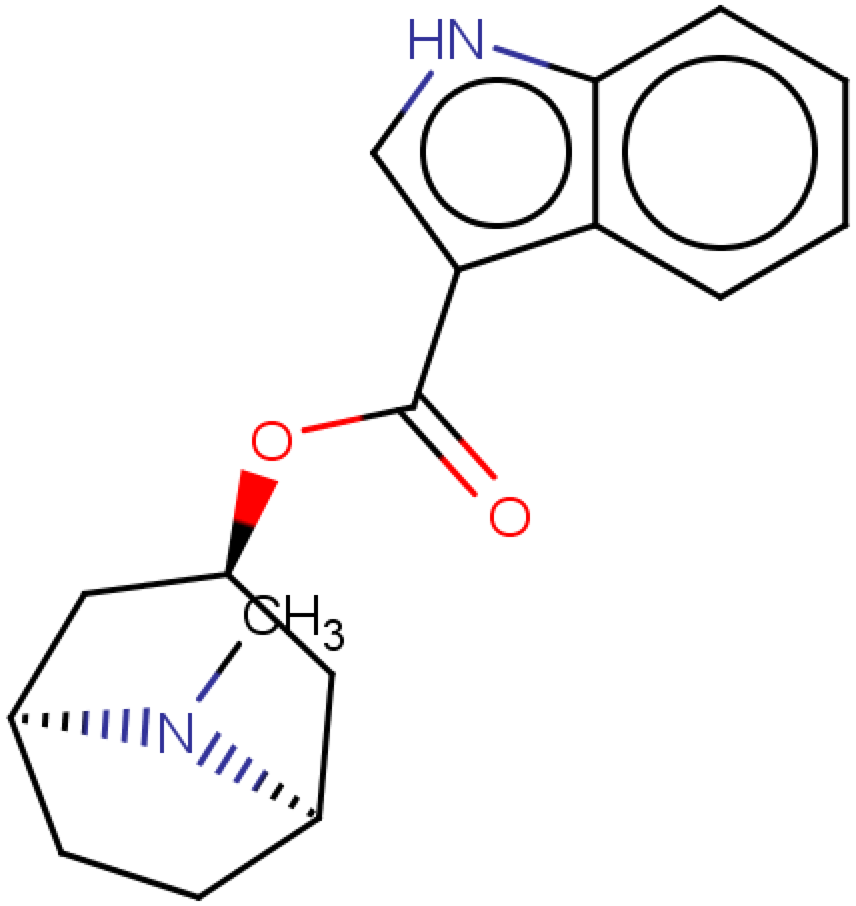

89565-68-4
Name: Tropisetron
pIC50: 4.89
Rank: 318
Classes: Drug

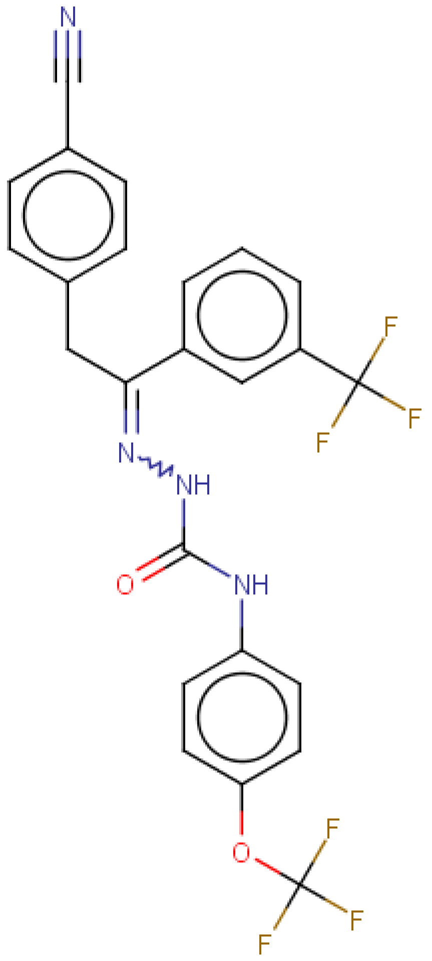

139968-49-3
Name: Metaflumizone
pIC50: 4.88
Rank: 319
Classes: Pesticide

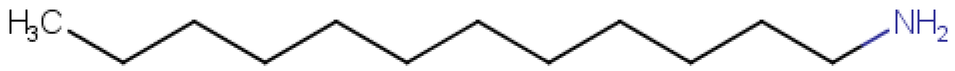

929-73-7
Name: Dodecylamine hydrochloride
pIC50: 4.88
Rank: 320
Classes: TSCA

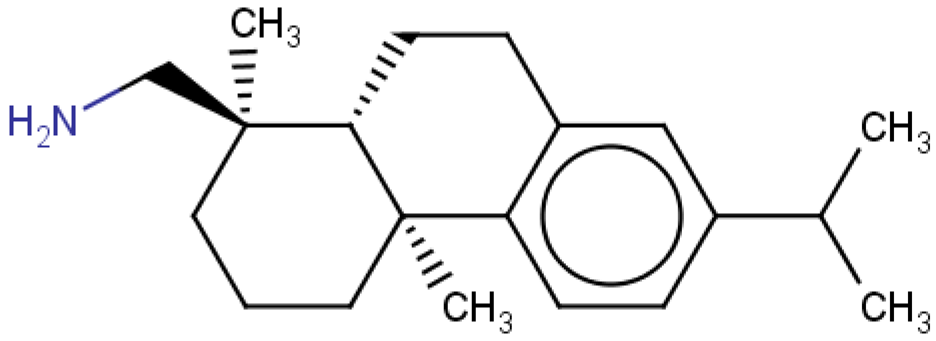

2026-24-6
Name: Dehydroabietylamine acetate
pIC50: 4.88
Rank: 321
Classes: No defined

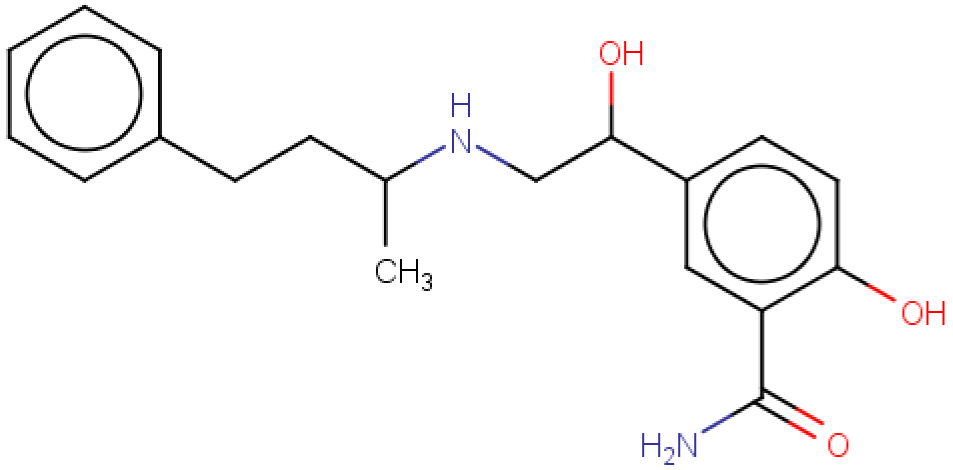

32780-64-6
Name: Labetalol hydrochloride
pIC50: 4.88
Rank: 322
Classes: No defined

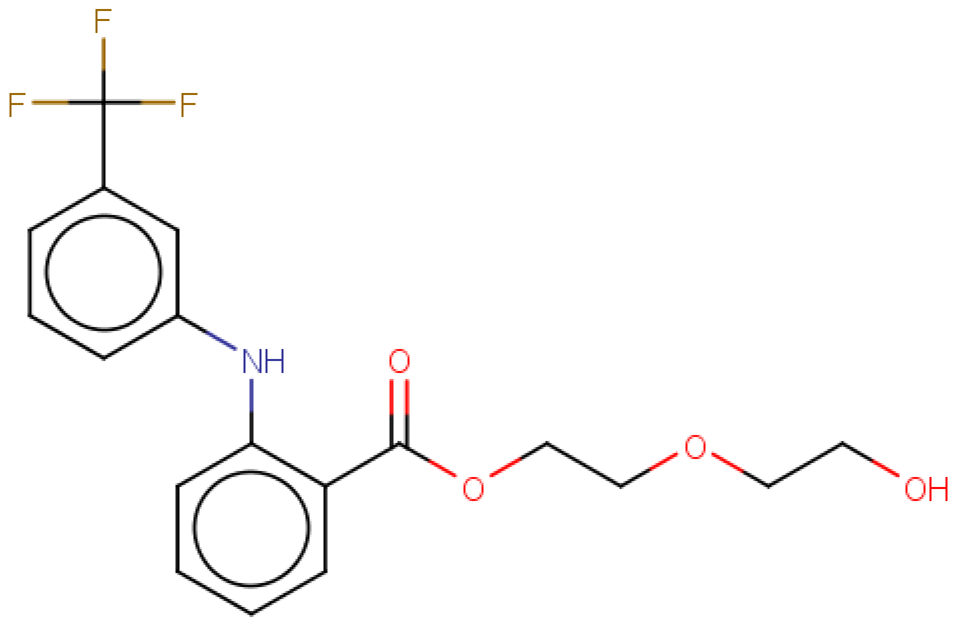

30544-47-9
Name: Etofenamate
pIC50: 4.87
Rank: 323
Classes: Drug

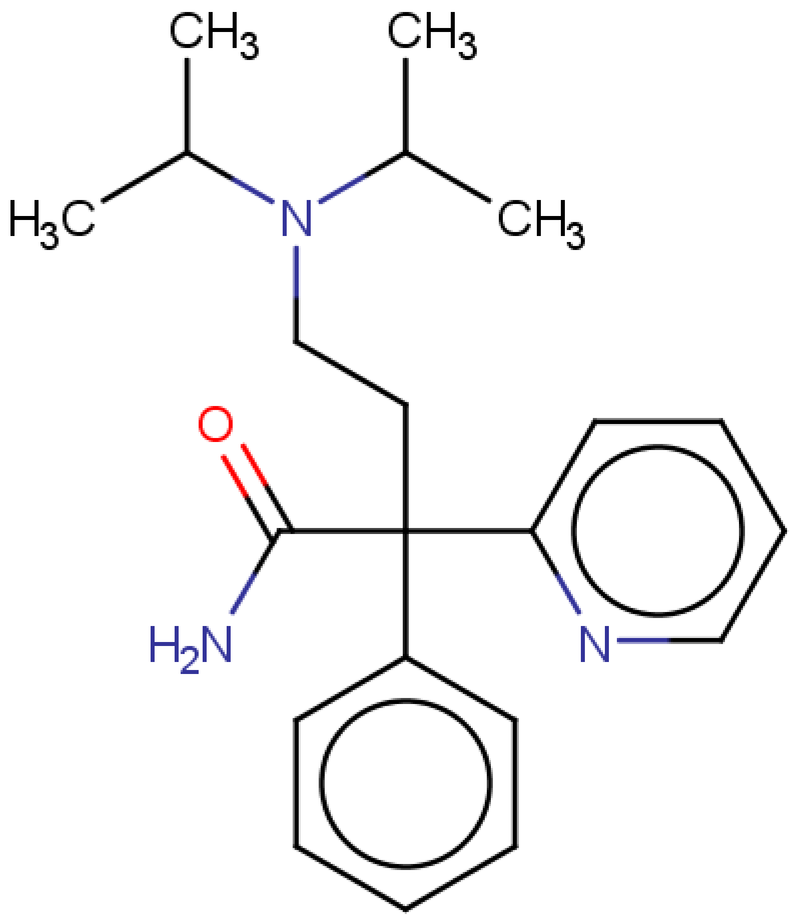

3737-09-5
Name: Disopyramide
pIC50: 4.87
Rank: 324
Classes: Drug

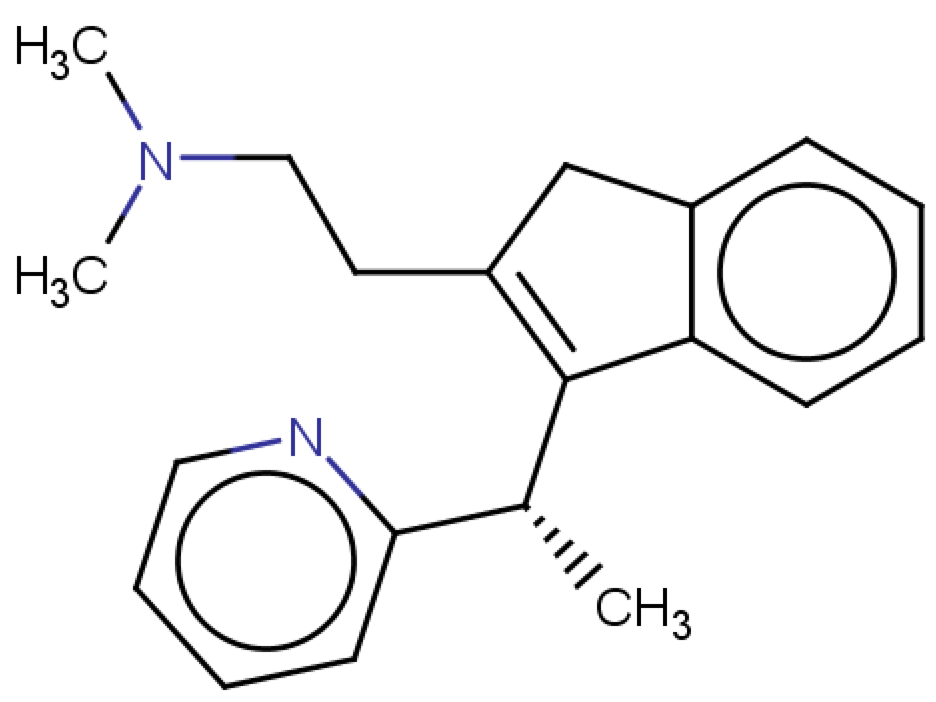

1217457-81-2
Name: (S)-(+)-Dimethindene maleate
pIC50: 4.87
Rank: 325
Classes: No defined

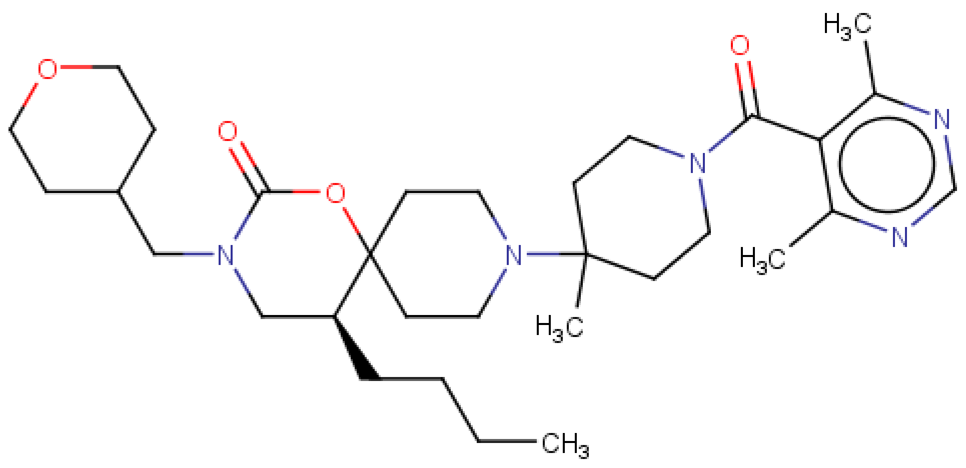

1191914-21-2
Name: PharmaGSID_48521
pIC50: 4.86
Rank: 326
Classes: No defined

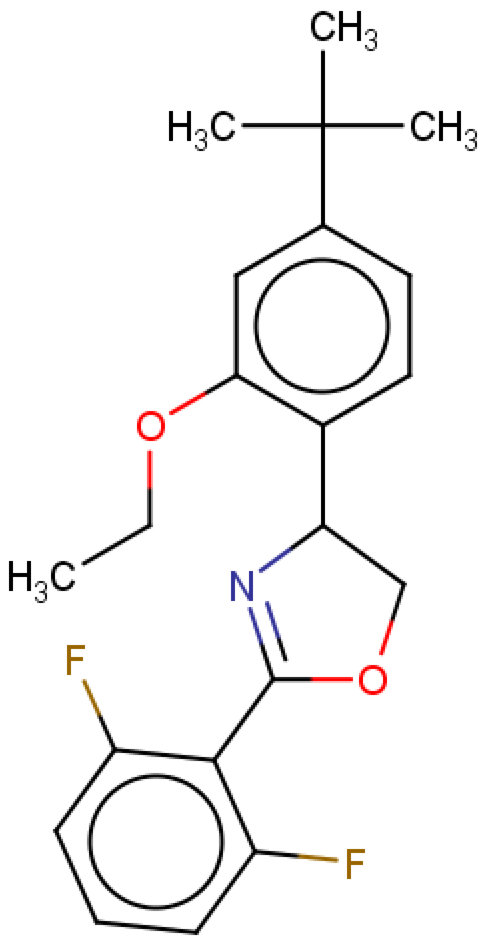

153233-91-1
Name: Etoxazole
pIC50: 4.86
Rank: 327
Classes: Pesticide

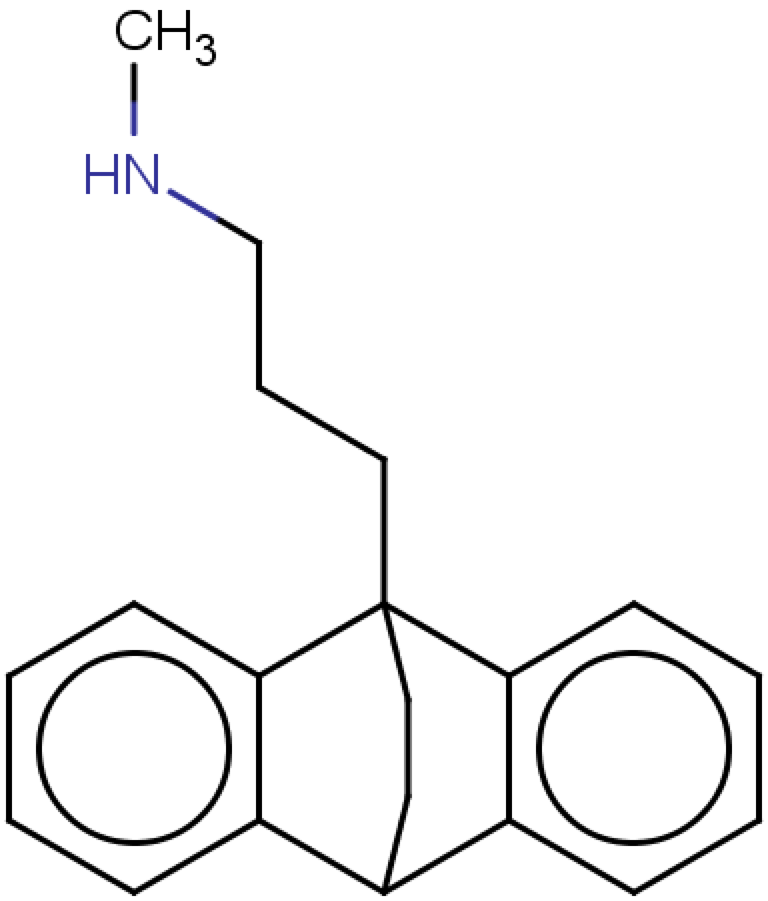

10262-69-8
Name: Maprotiline
pIC50: 4.85
Rank: 328
Classes: Drug

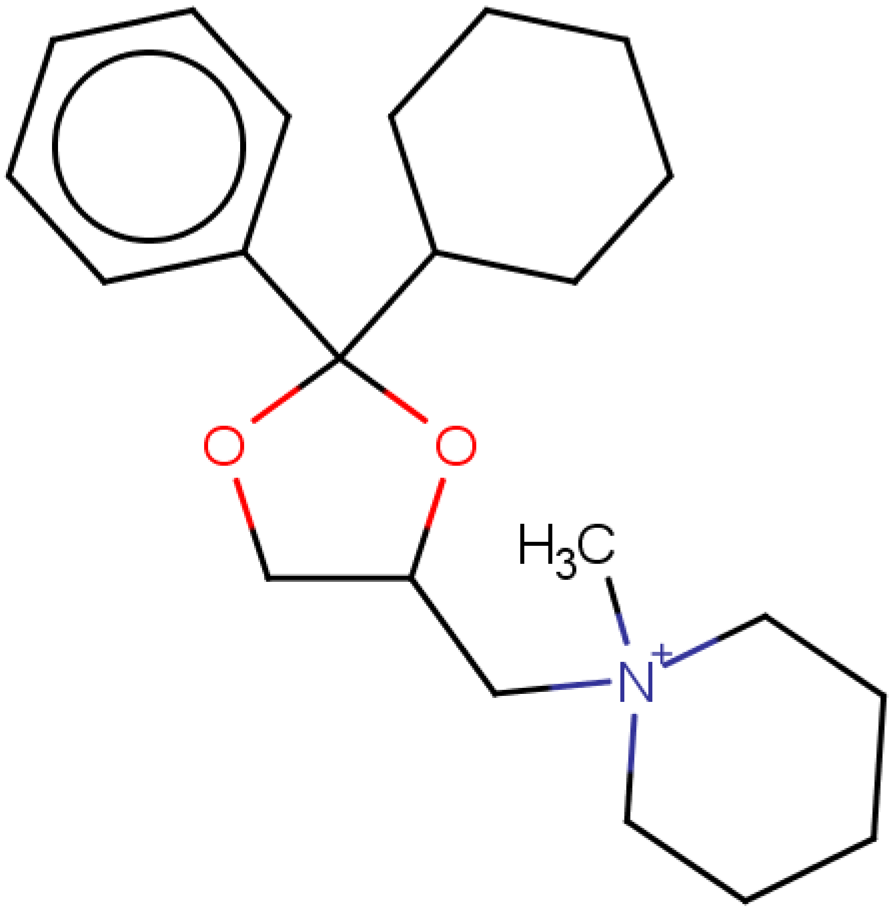

6577-41-9
Name: Oxapium iodide
pIC50: 4.85
Rank: 329
Classes: No defined

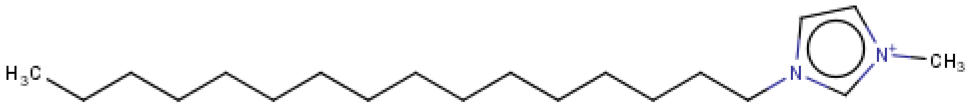

61546-01-8
Name: 1-Hexadecyl-3-methylimidazolium chlo
pIC50: 4.85
Rank: 330
Classes: No defined

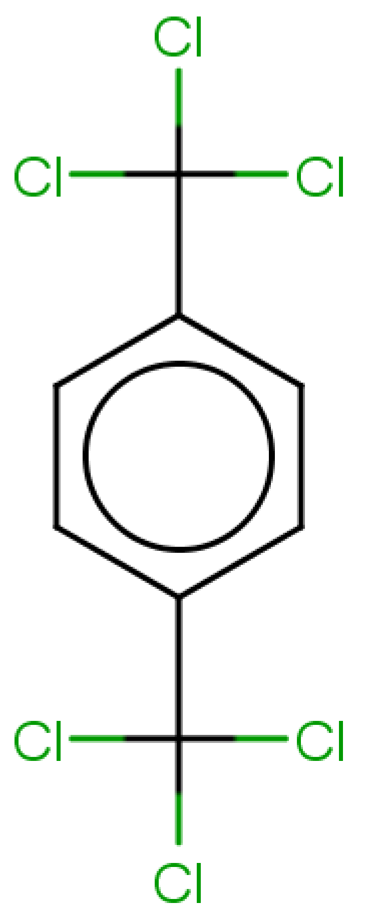

68-36-0
Name: 1 4-Bis(trichloromethyl)benzene
pIC50: 4.85
Rank: 331
Classes: No defined

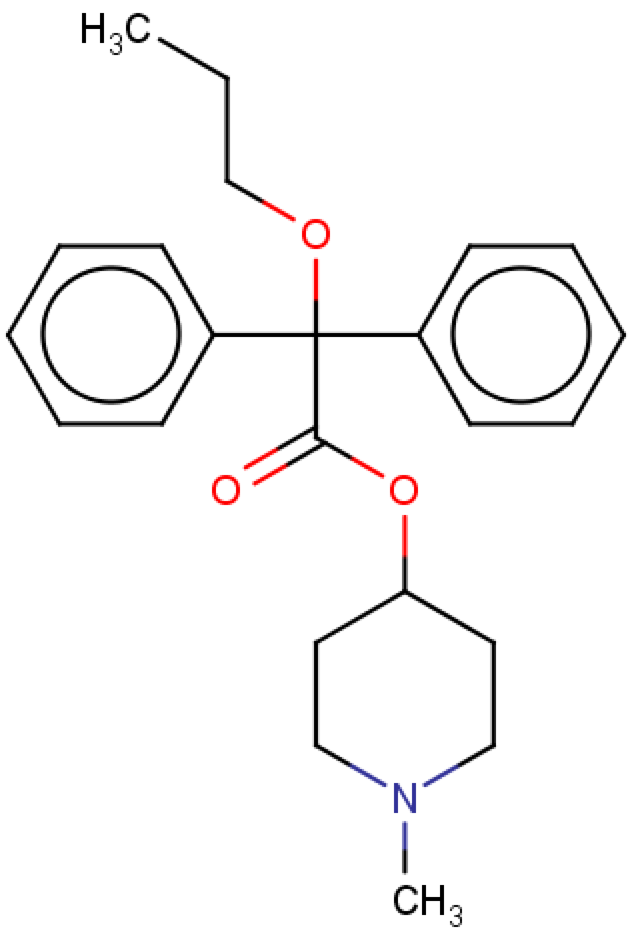

54556-98-8
Name: Propiverine hydrochloride
pIC50: 4.85
Rank: 332
Classes: No defined

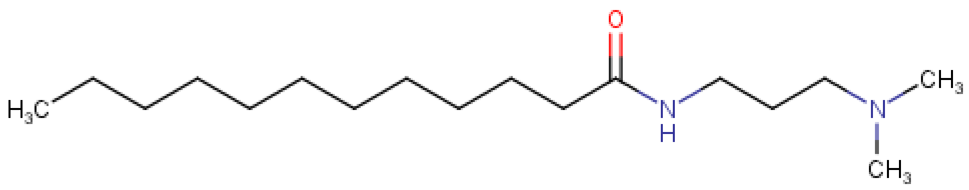

3179-80-4
Name: N-[3-(Dimethylamino)propyl]dodecana
pIC50: 4.84
Rank: 333
Classes: antistatic agent--Pesticide--TSCA

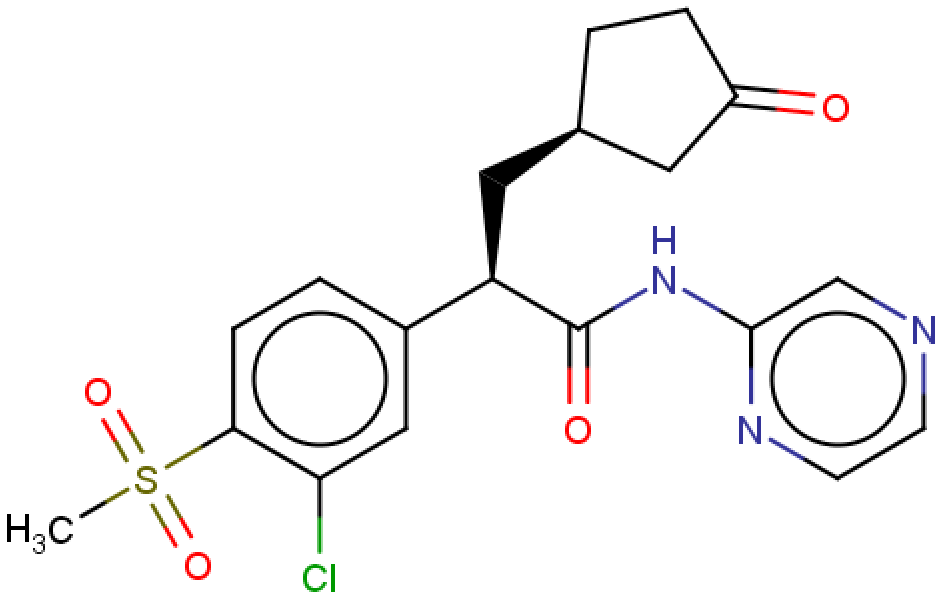

625114-41-2
Name: Piragliatin
pIC50: 4.84
Rank: 334
Classes: No defined

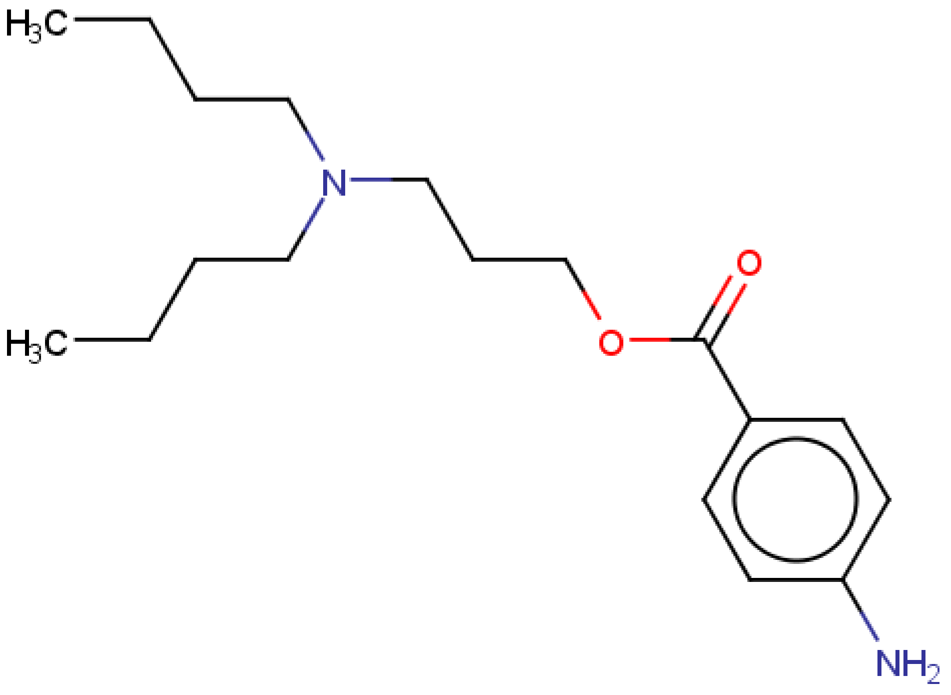

149-16-6
Name: Butacaine
pIC50: 4.84
Rank: 335
Classes: No defined

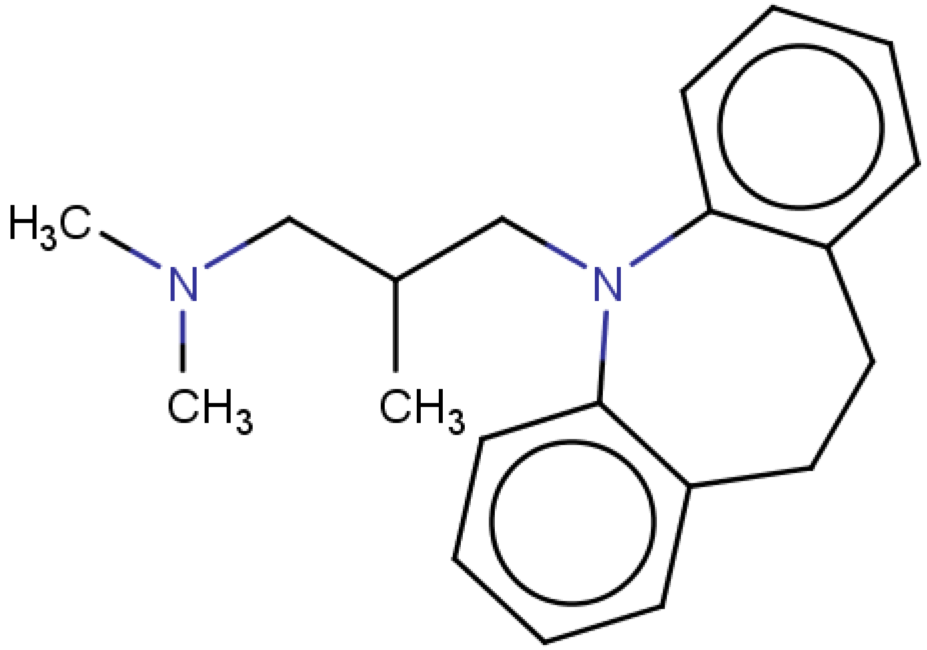

521-78-8
Name: Trimipramine maleate
pIC50: 4.84
Rank: 336
Classes: No defined

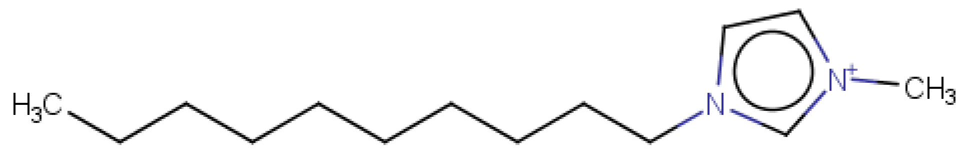

188589-32-4
Name: 1-Decyl-3-methylimidazolium bromide
pIC50: 4.83
Rank: 337
Classes: No defined

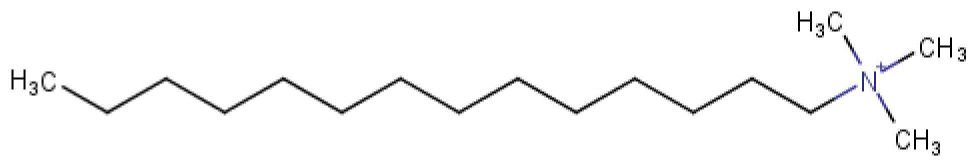

4574-04-3
Name: Myristyltrimethylammonium chlorideN,N,N-Trimethyltetradecan-1-aminium chloride
pIC50: 4.82
Rank: 338
Classes: Pesticide

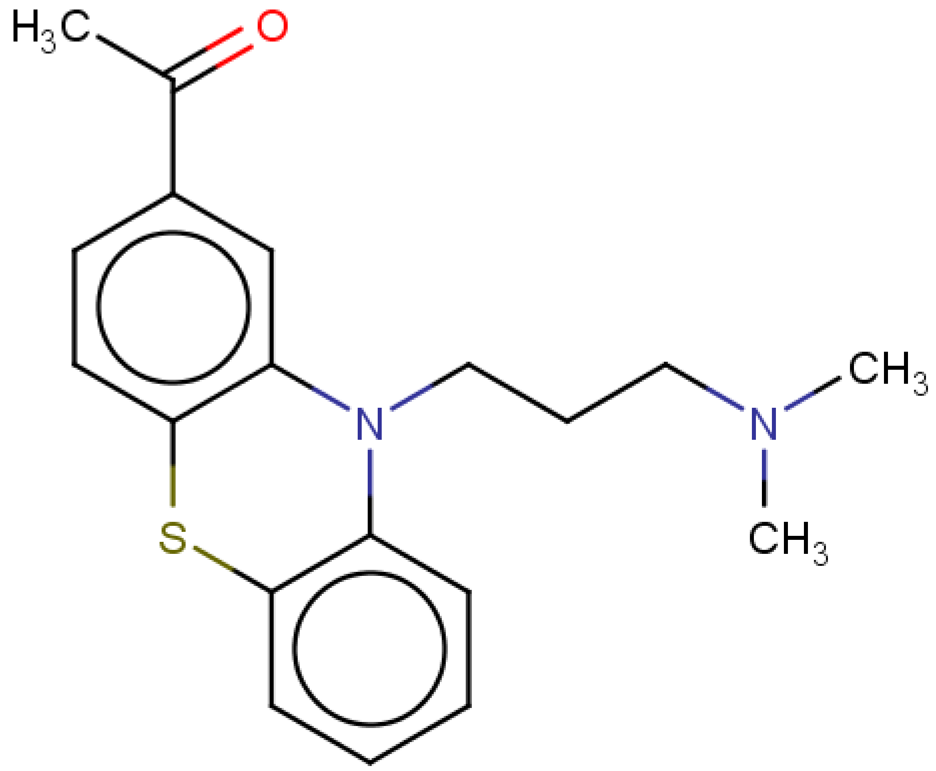

3598-37-6
Name: Acetylpromazine maleate
pIC50: 4.82
Rank: 339
Classes: No defined

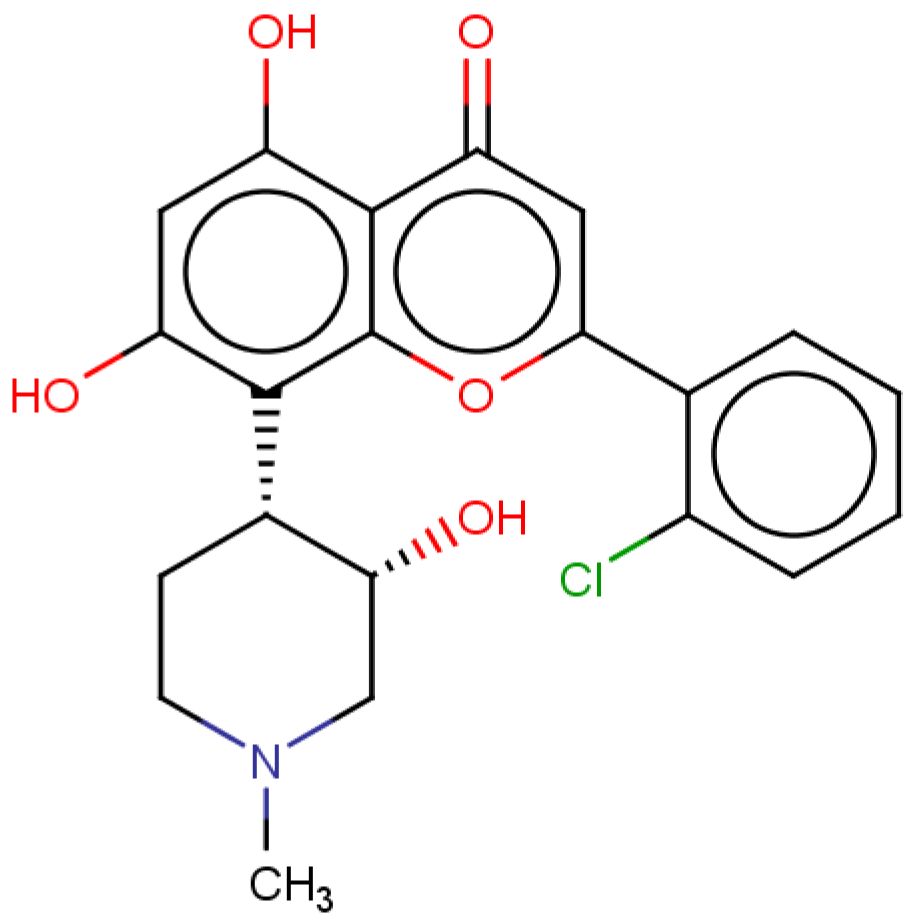

131740-09-5
Name: Flavopiridol hydrochloride
pIC50: 4.82
Rank: 340
Classes: No defined

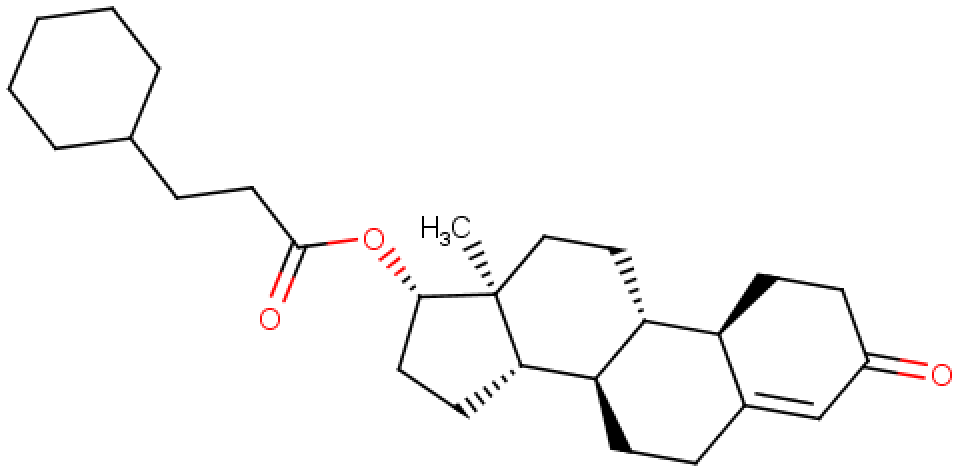

912-57-2
Name: Nandrolone cyclohexylpropionate
pIC50: 4.82
Rank: 341
Classes: No defined

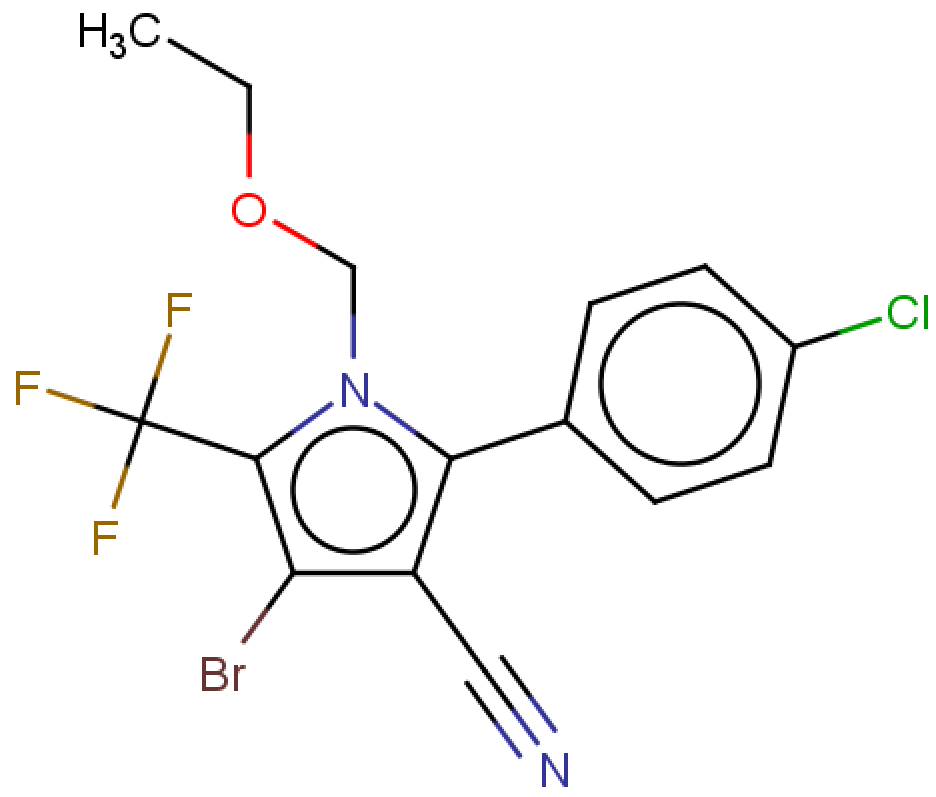

122453-73-0
Name: Chlorfenapyr
pIC50: 4.81
Rank: 342
Classes: Pesticide

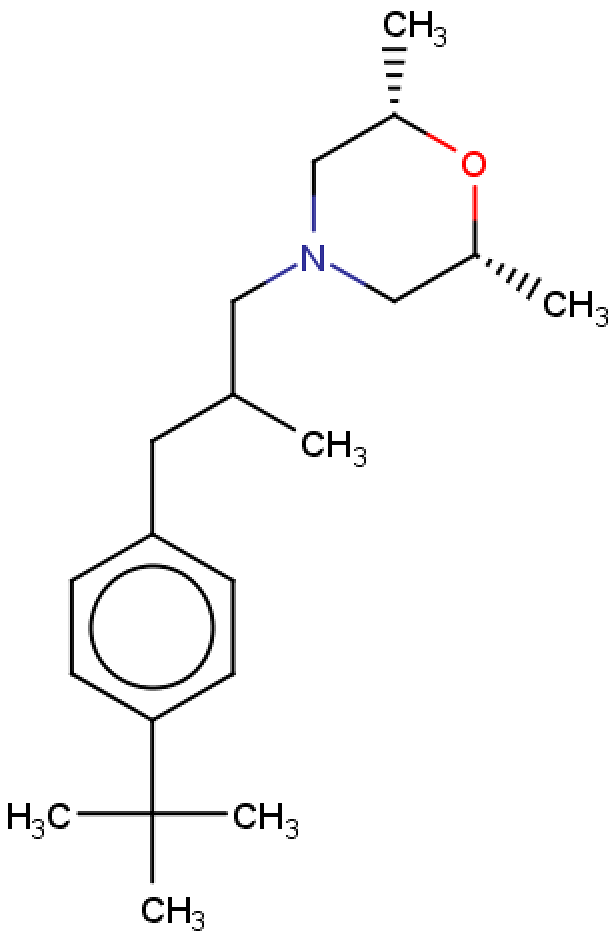

67564-91-4
Name: (2R 6S)-Fenpropimorph
pIC50: 4.81
Rank: 343
Classes: antimicrobial

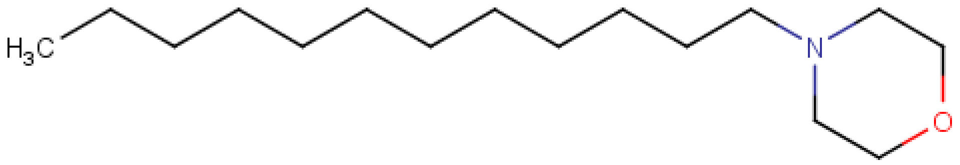

1541-81-7
Name: 4-Dodecylmorpholine
pIC50: 4.81
Rank: 344
Classes: No defined

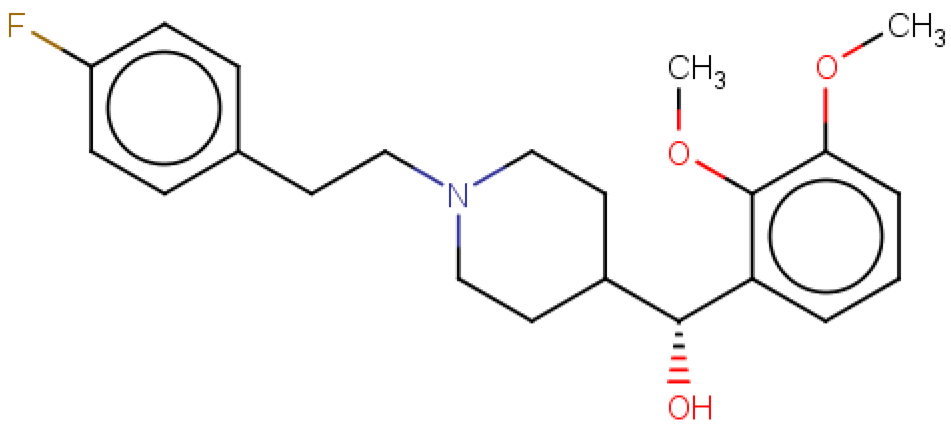

139290-65-6
Name: Volinanserin
pIC50: 4.81
Rank: 345
Classes: No defined

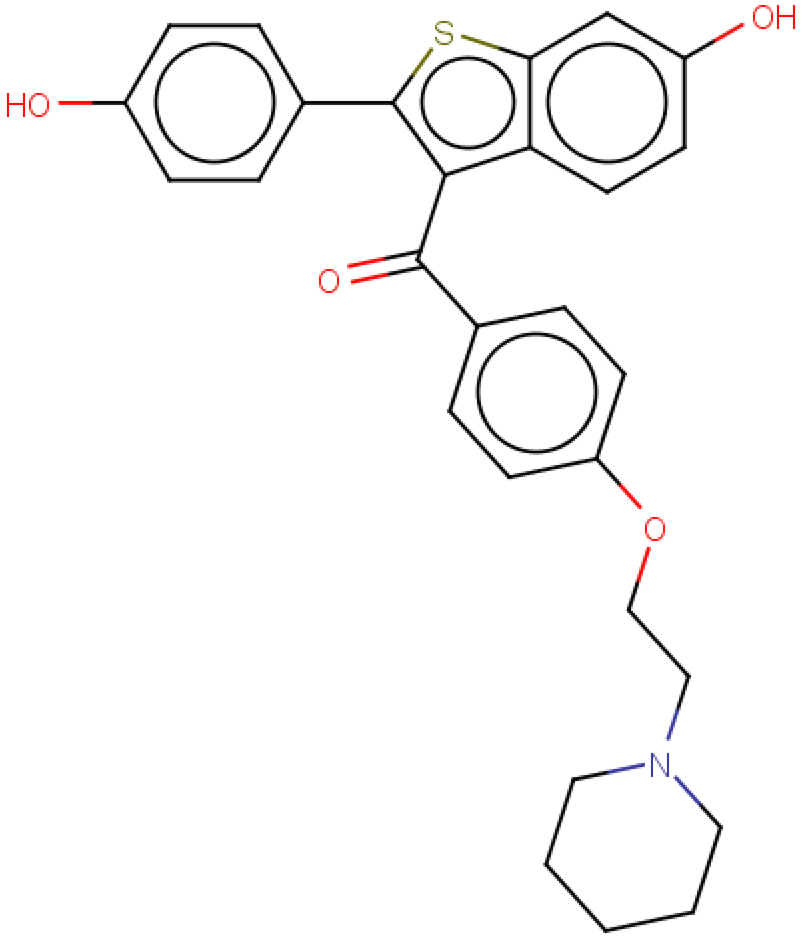

84449-90-1
Name: Raloxifene
pIC50: 4.81
Rank: 346
Classes: Drug

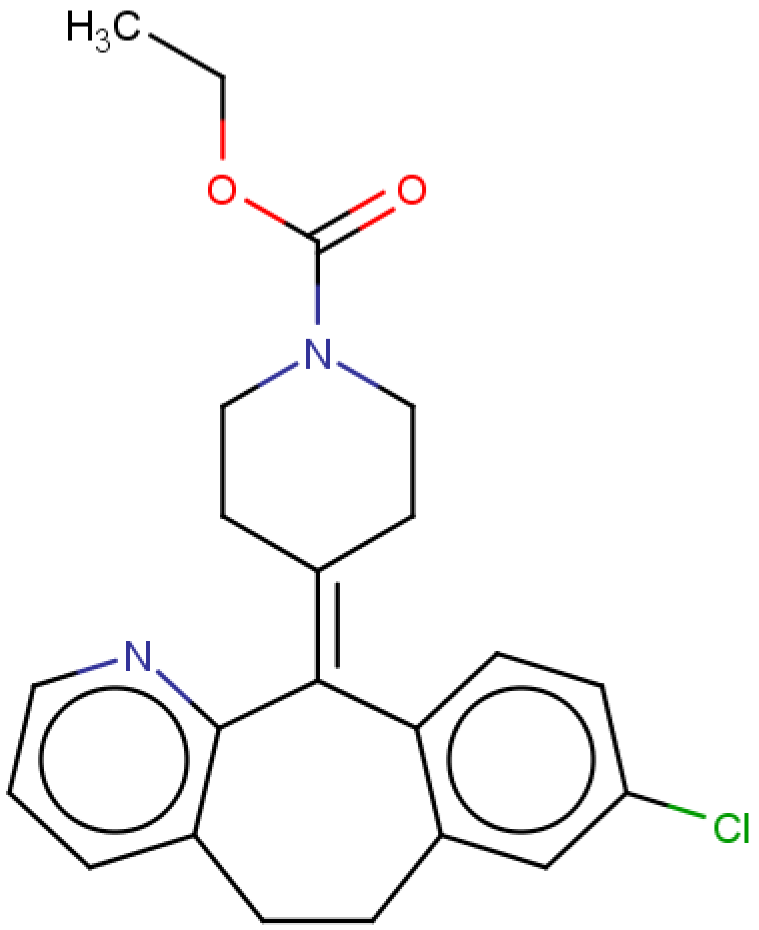

79794-75-5
Name: Loratadine
pIC50: 4.8
Rank: 347
Classes: Drug

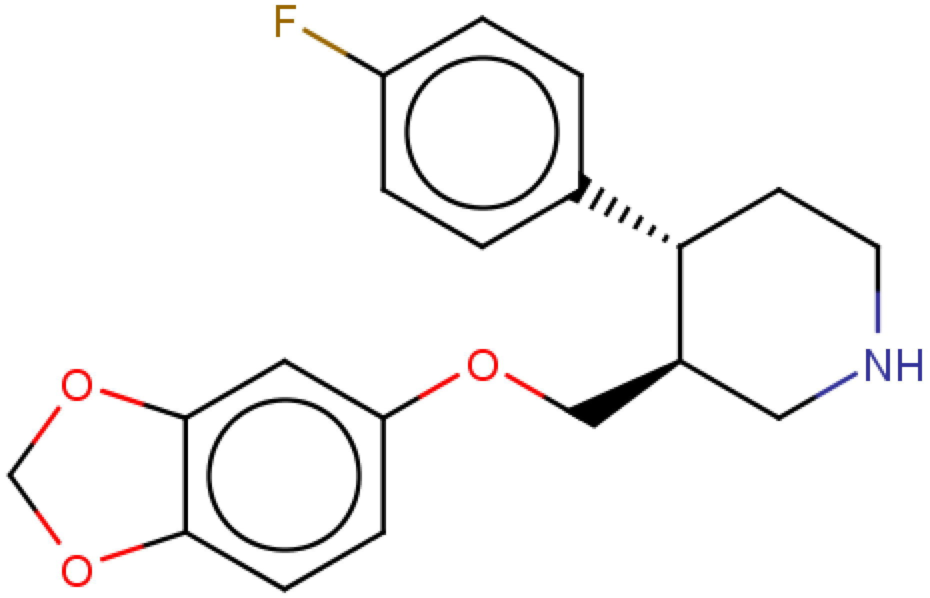

61869-08-7
Name: Paroxetine
pIC50: 4.8
Rank: 348
Classes: Drug

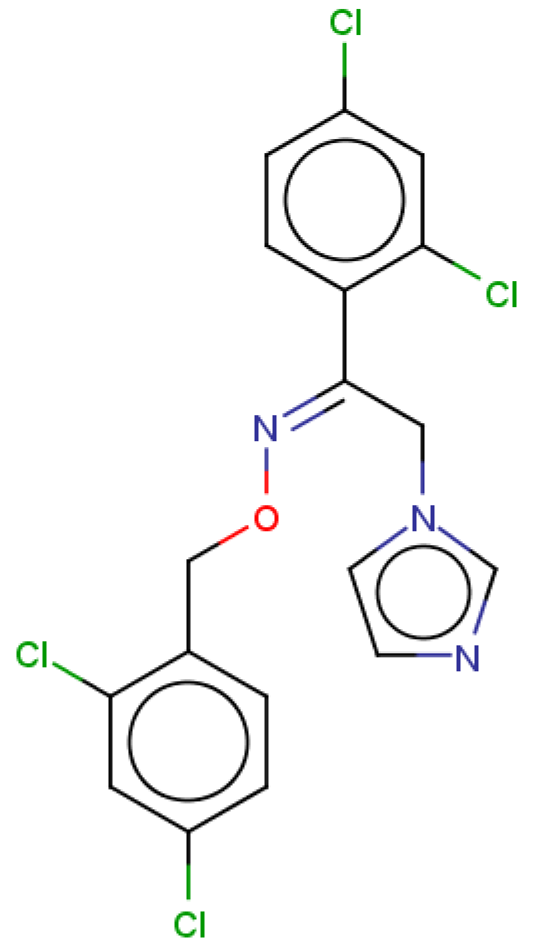

64211-46-7
Name: Oxiconazole nitrate
pIC50: 4.8
Rank: 349
Classes: No defined

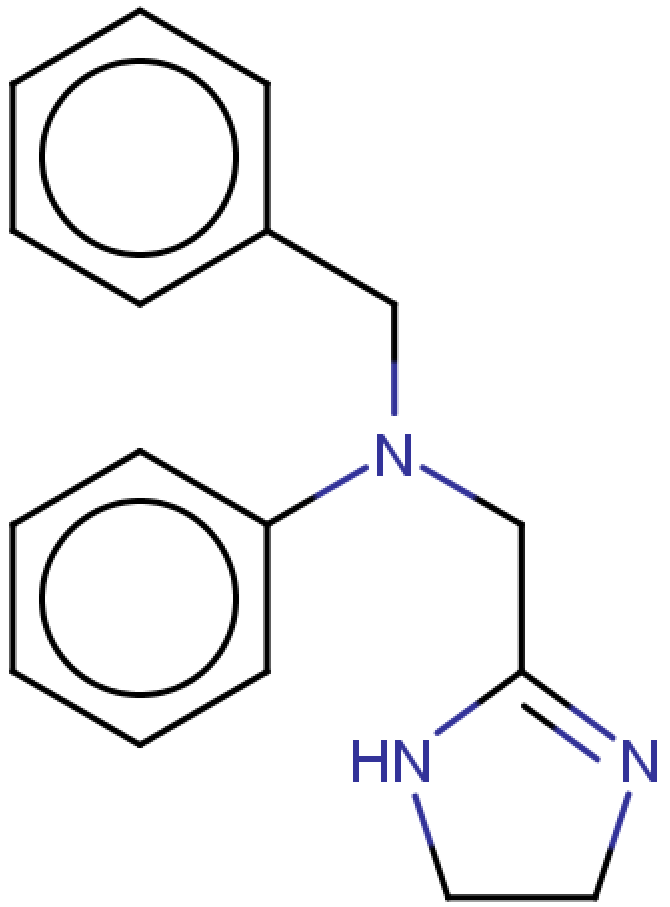

2508-72-7
Name: Antazoline hydrochloride
pIC50: 4.8
Rank: 350
Classes: No defined

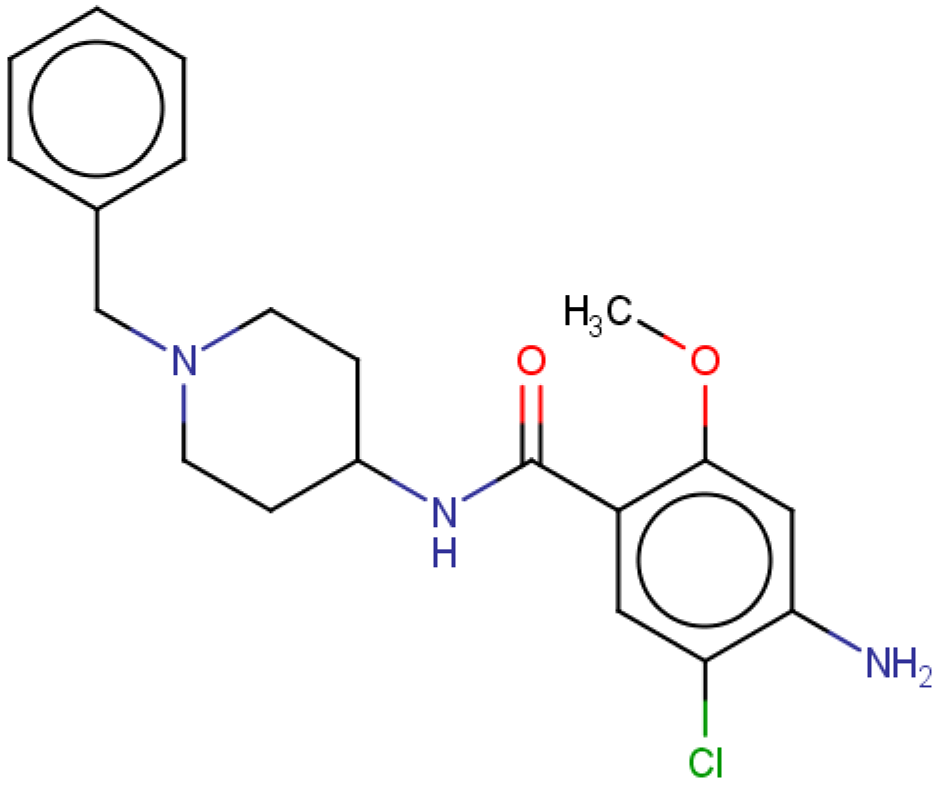

84370-95-6
Name: Clebopride maleate
pIC50: 4.8
Rank: 351
Classes: No defined

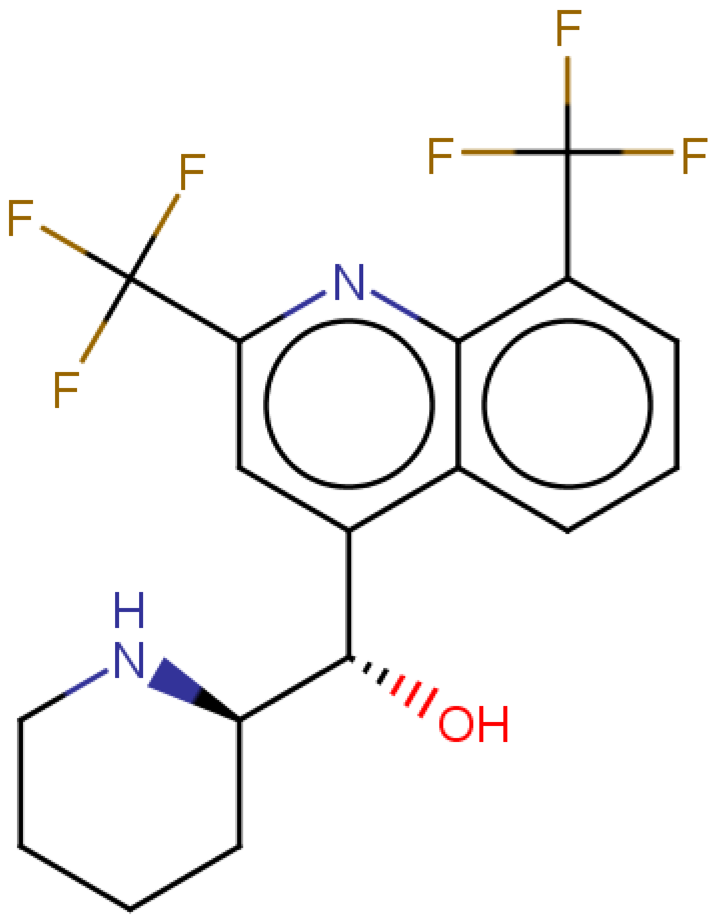

51773-92-3
Name: Mefloquine hydrochloride
pIC50: 4.8
Rank: 352
Classes: No defined

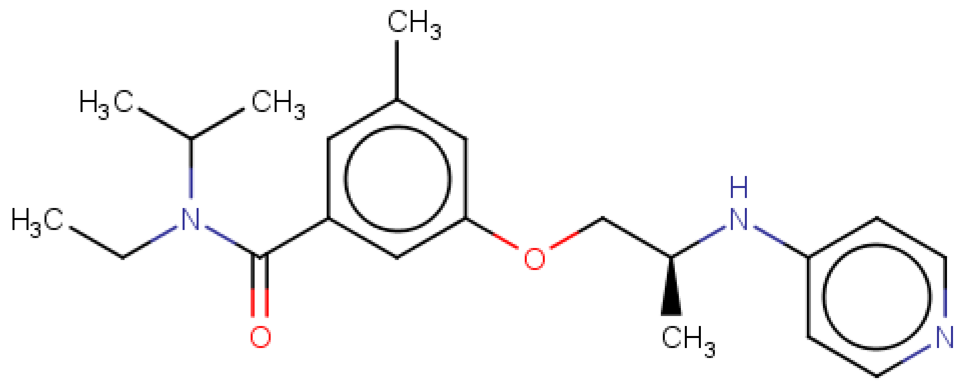

263553-33-9
Name: GW473178E methyl benzene sulphonic acid
pIC50: 4.8
Rank: 353
Classes: No defined

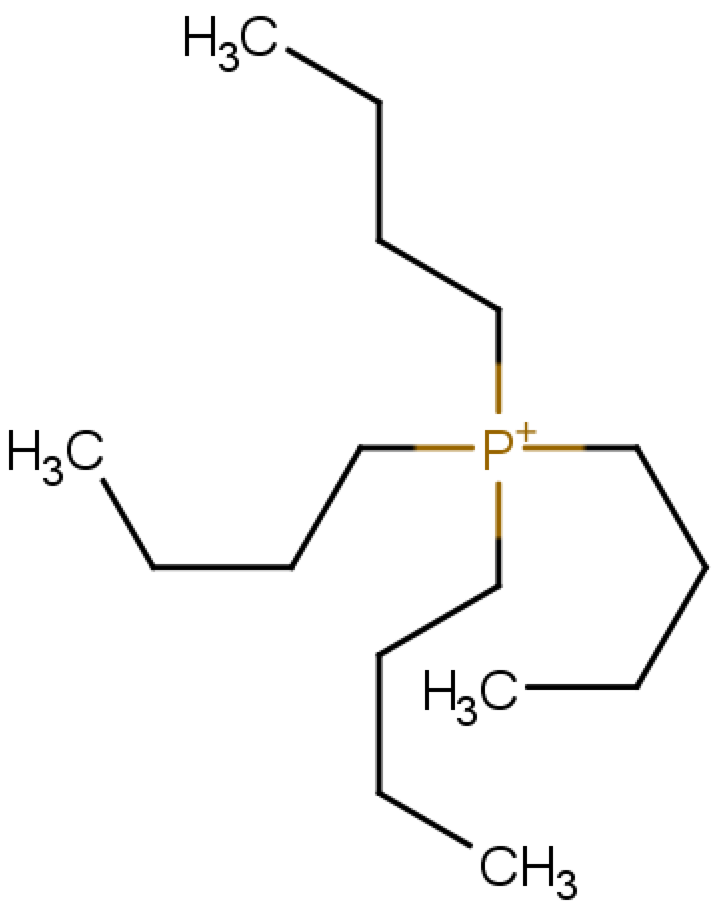

3115-68-2
Name: Tetrabutylphosphonium bromide
pIC50: 4.79
Rank: 354
Classes: catalyst--TSCA

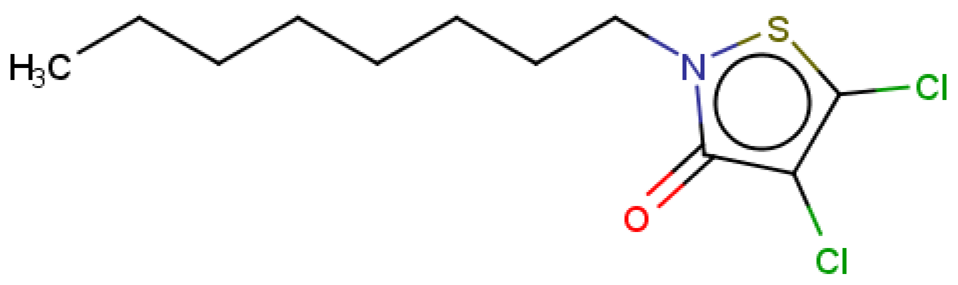

64359-81-5
Name: 4 5-Dichloro-2-octyl-3(2H)-isothiazolone
pIC50: 4.79
Rank: 355
Classes: ubiquitous

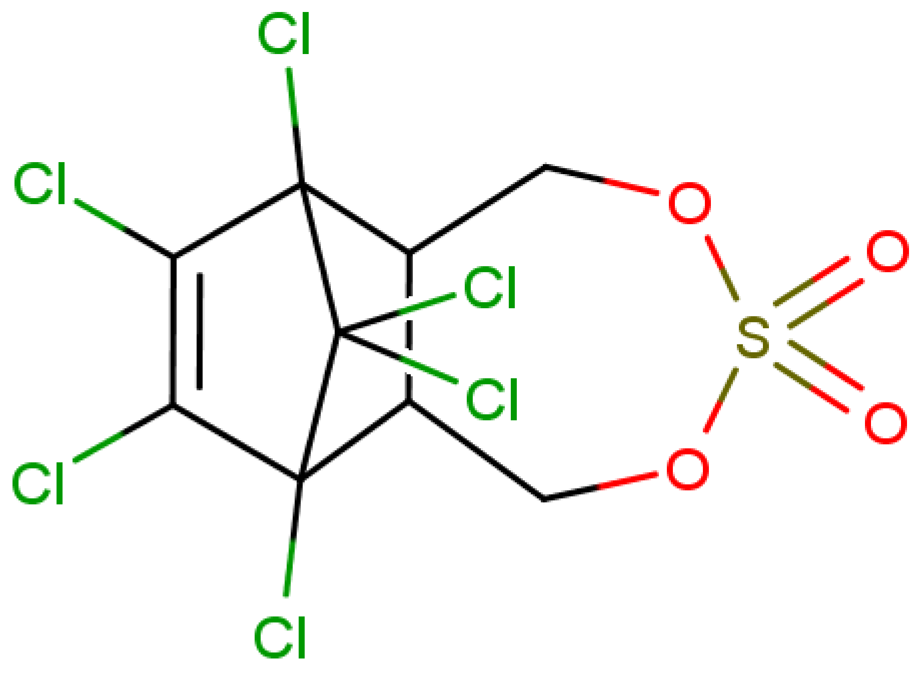

1031-07-8
Name: Endosulfan sulfate
pIC50: 4.79
Rank: 356
Classes: No defined

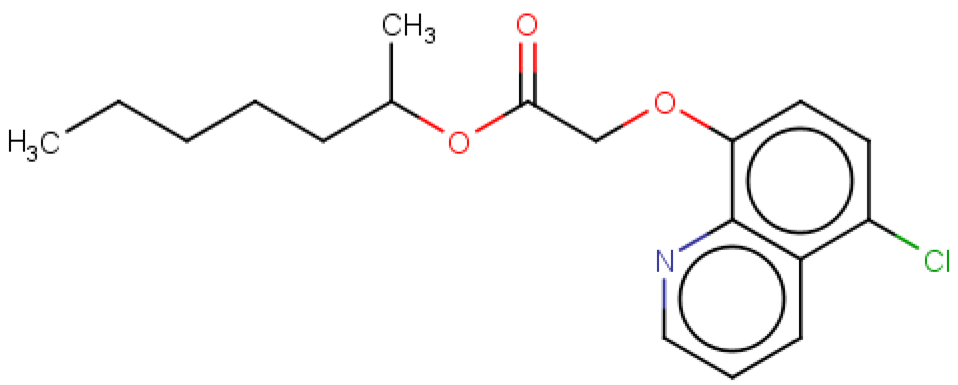

99607-70-2
Name: Cloquintocet-mexyl
pIC50: 4.79
Rank: 357
Classes: Pesticide--TSCA

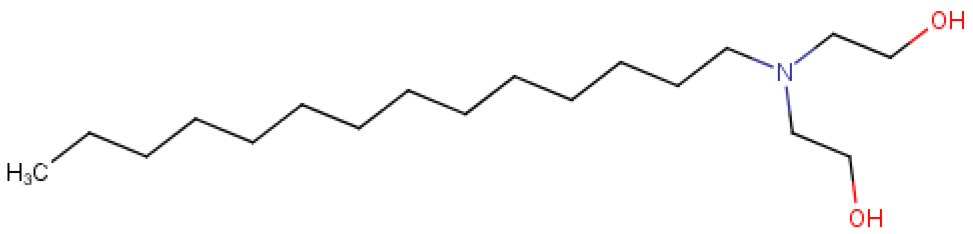

18924-66-8
Name: 2 2'-(Tetradecylimino)diethanol
pIC50: 4.79
Rank: 358
Classes: No defined

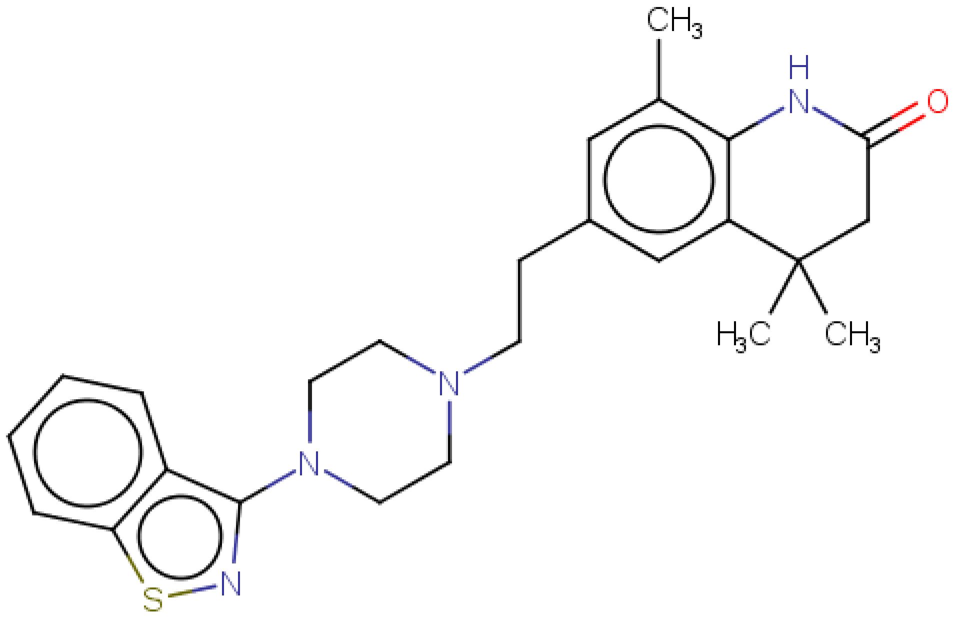

676116-04-4
Name: PD 0343701
pIC50: 4.79
Rank: 359
Classes: No defined

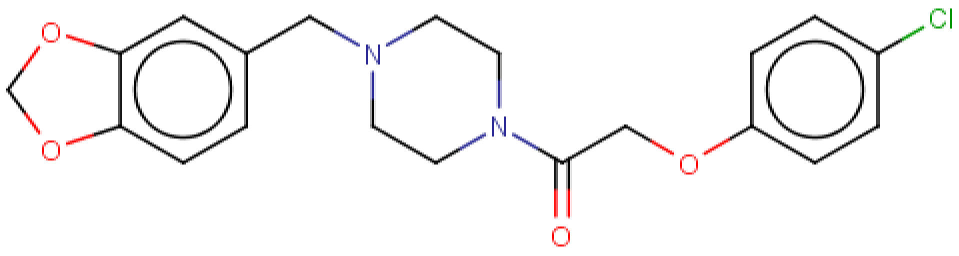

34161-23-4
Name: Fipexide hydrochloride
pIC50: 4.79
Rank: 360
Classes: No defined

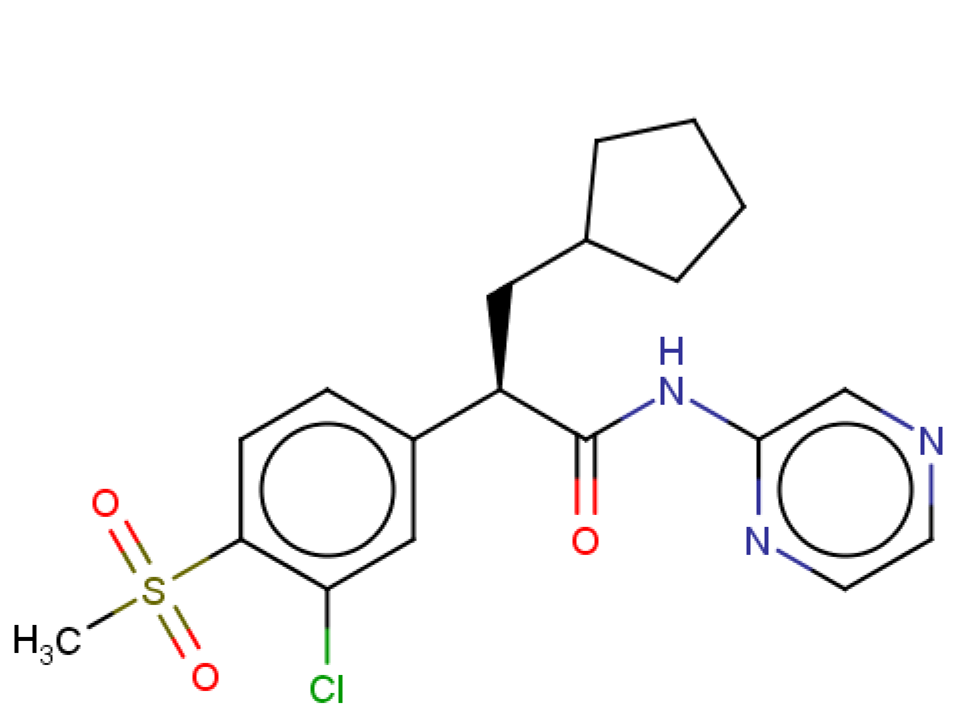

588941-45-1
Name: PharmaGSID_48506
pIC50: 4.79
Rank: 361
Classes: No defined

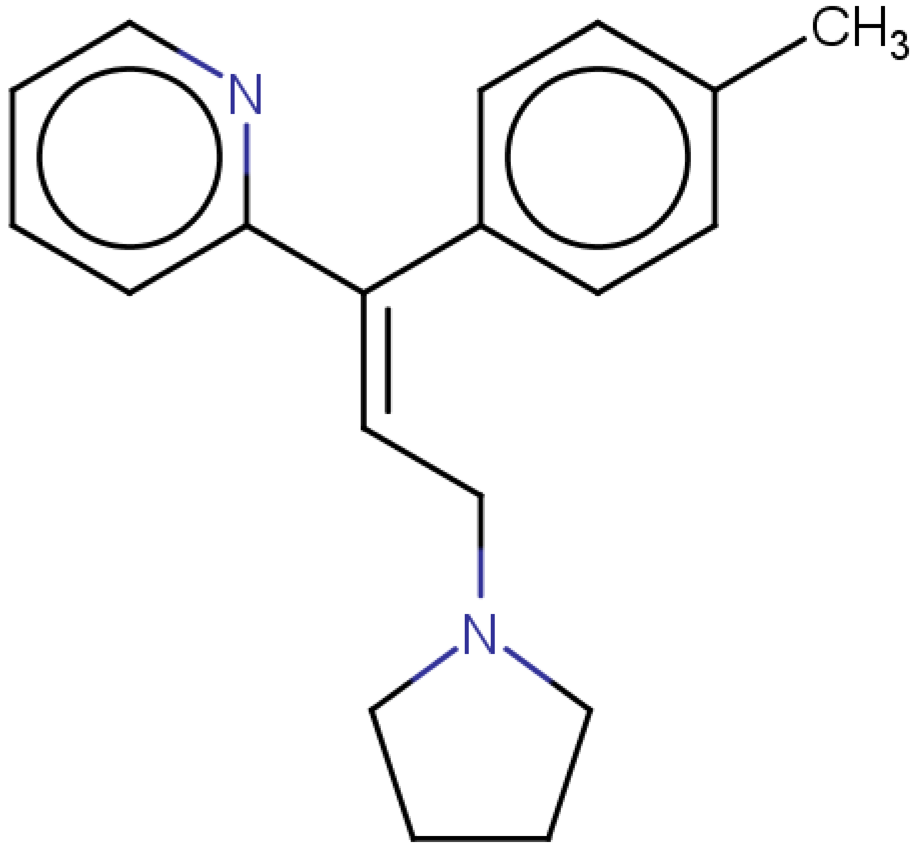

6138-79-0
Name: Triprolidine hydrochloride monohydrate
pIC50: 4.79
Rank: 362
Classes: No defined

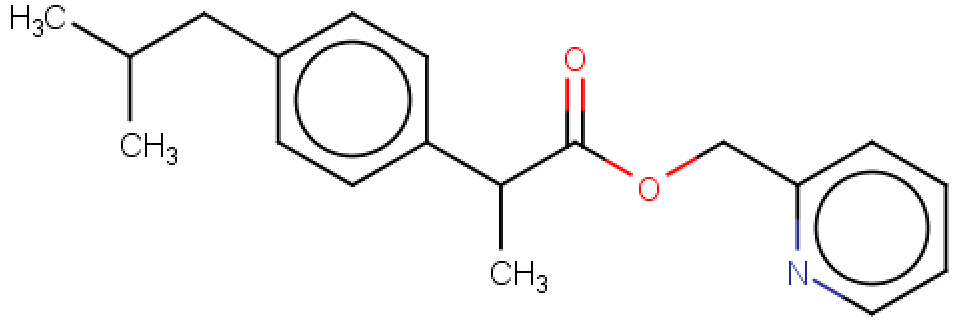

64622-45-3
Name: Ibuprofen piconol
pIC50: 4.79
Rank: 363
Classes: No defined

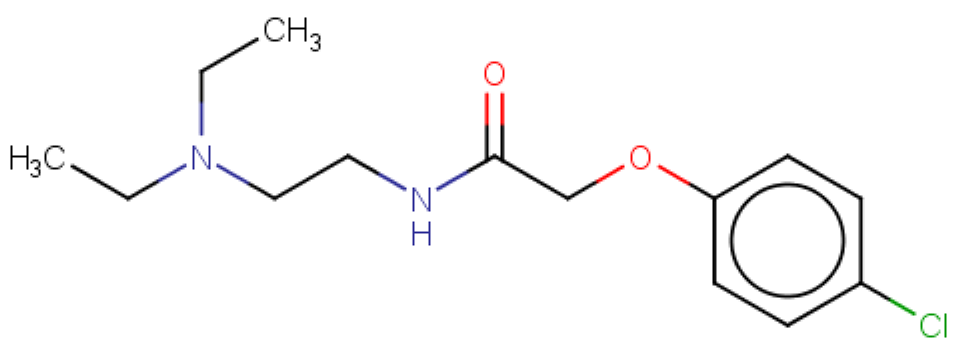

1223-36-5
Name: Clofexamide
pIC50: 4.79
Rank: 364
Classes: No defined

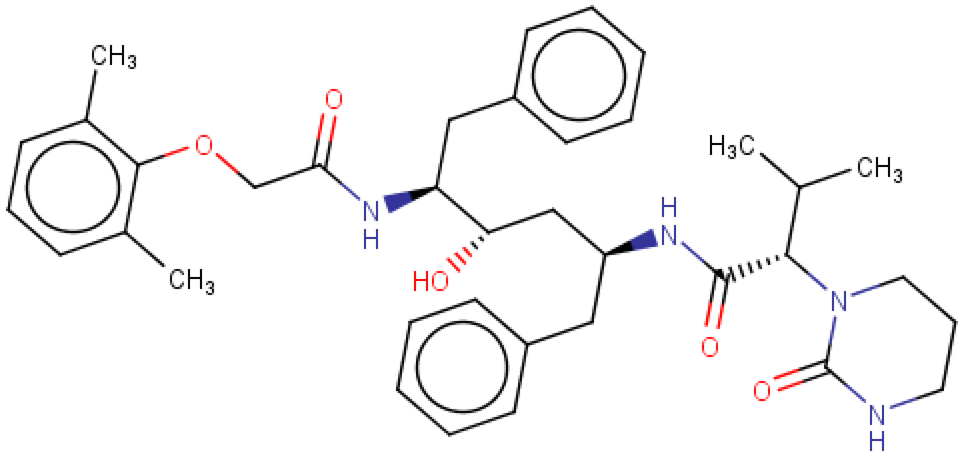

192725-17-0
Name: Lopinavir
pIC50: 4.79
Rank: 365
Classes: Drug

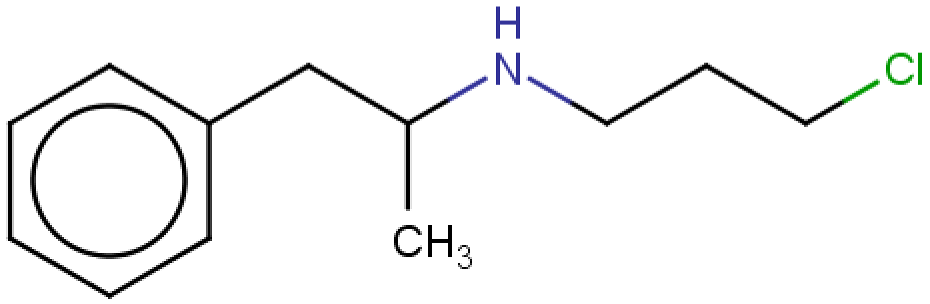

17243-57-1
Name: Mefenorex
pIC50: 4.79
Rank: 366
Classes: No defined

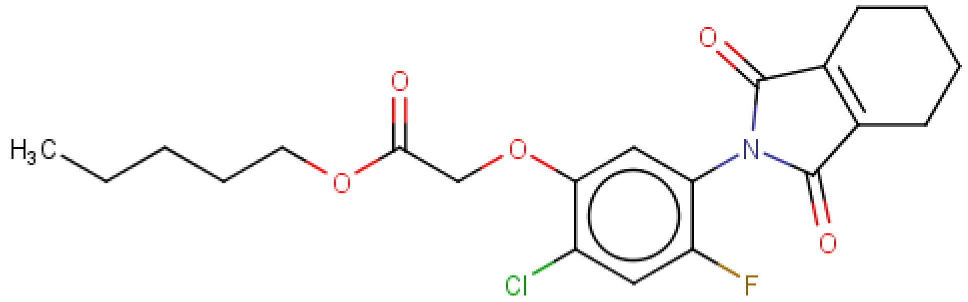

87546-18-7
Name: Flumiclorac-pentyl
pIC50: 4.79
Rank: 367
Classes: Pesticide

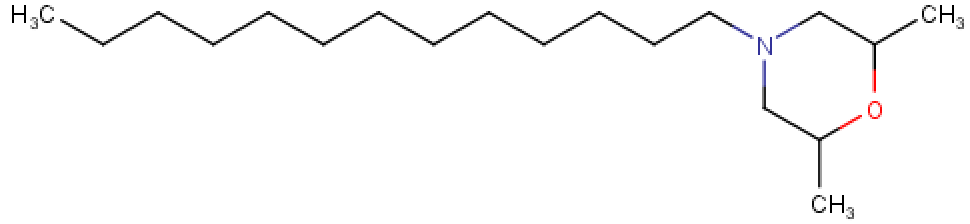

24602-86-6
Name: Tridemorph
pIC50: 4.79
Rank: 368
Classes: Pesticide

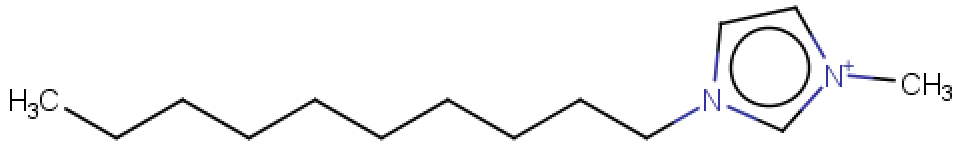

171058-18-7
Name: 1-Decyl-3-methylimidazolium chloride
pIC50: 4.78
Rank: 369
Classes: No defined

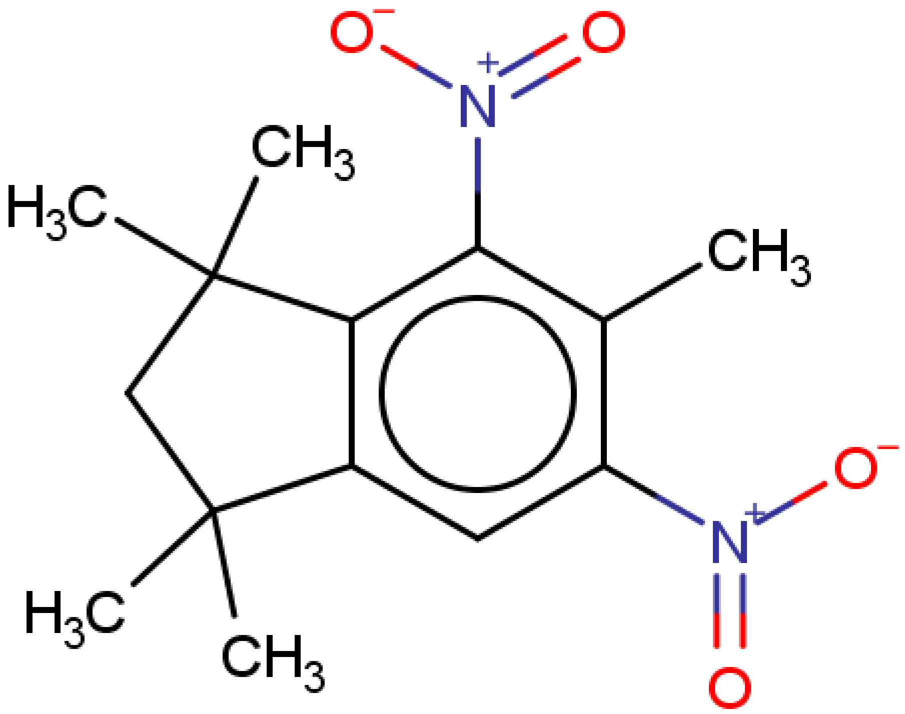

116-66-5
Name: 1 1 3 3 5-Pentamethyl-4 6-dinitro-2 3-dihydro-1H-indene
pIC50: 4.78
Rank: 370
Classes: TSCA

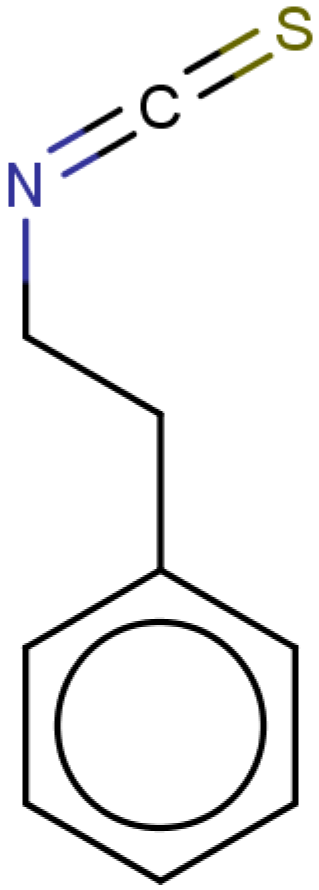

2257-09-2
Name: Phenethyl isothiocyanate
pIC50: 4.78
Rank: 371
Classes: fragrance--Drug

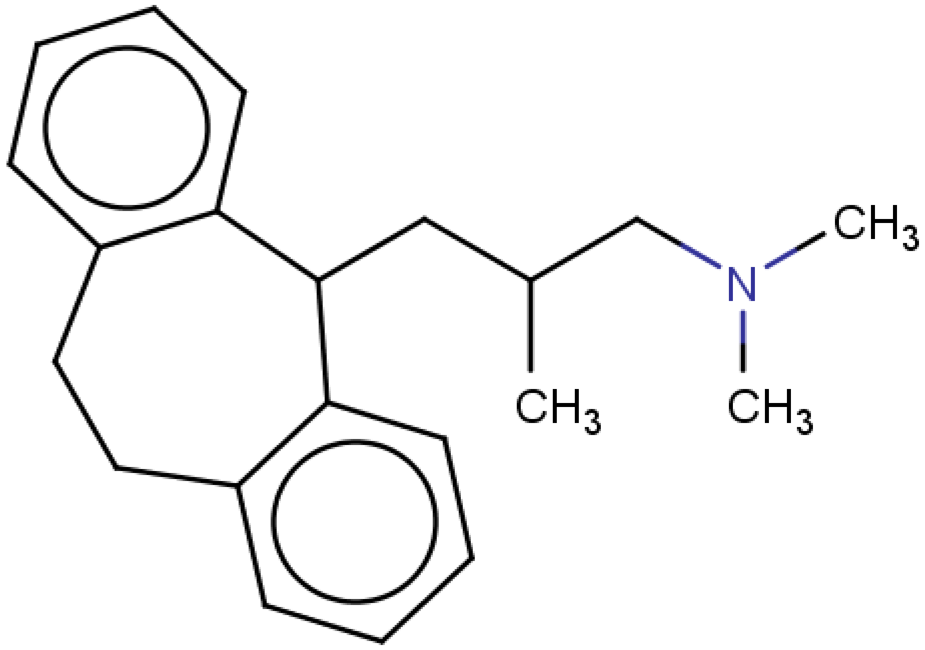

35941-65-2
Name: Butriptyline
pIC50: 4.77
Rank: 372
Classes: Drug

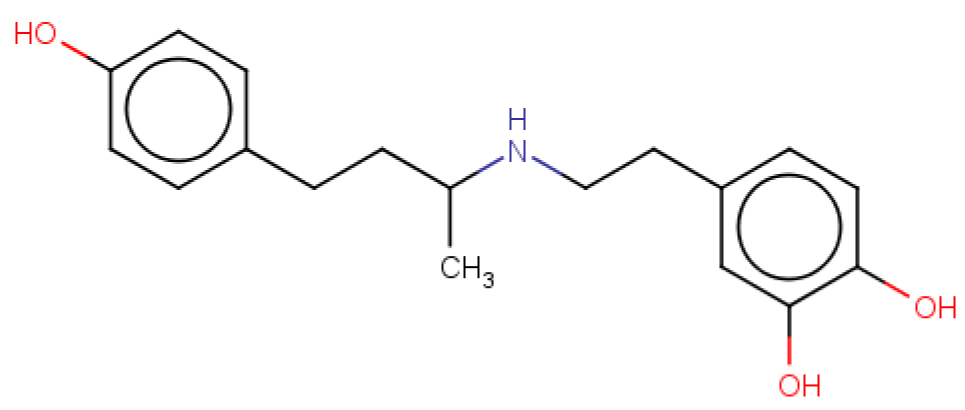

34368-04-2
Name: Dobutamine
pIC50: 4.77
Rank: 373
Classes: Drug

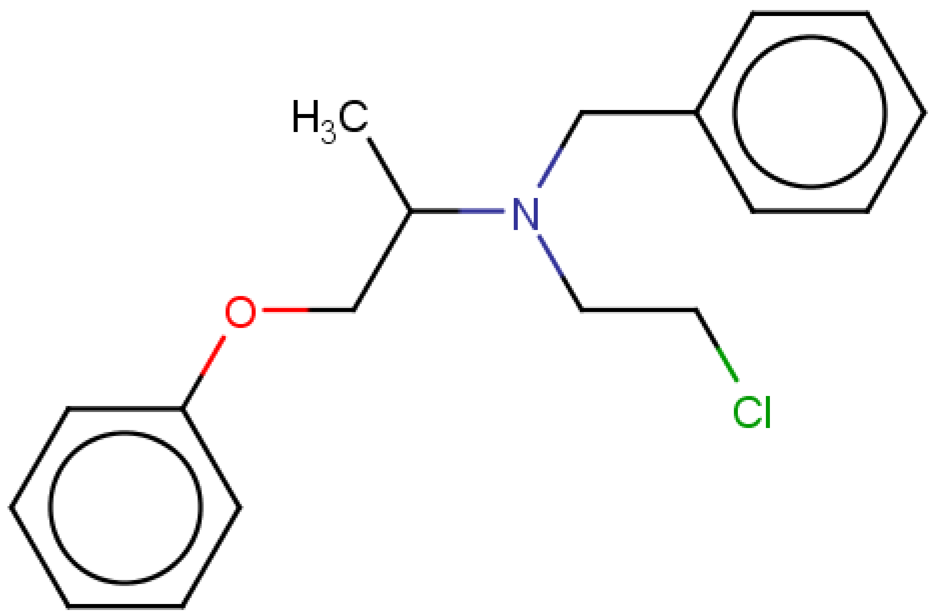

59-96-1
Name: Phenoxybenzamine
pIC50: 4.77
Rank: 374
Classes: Drug

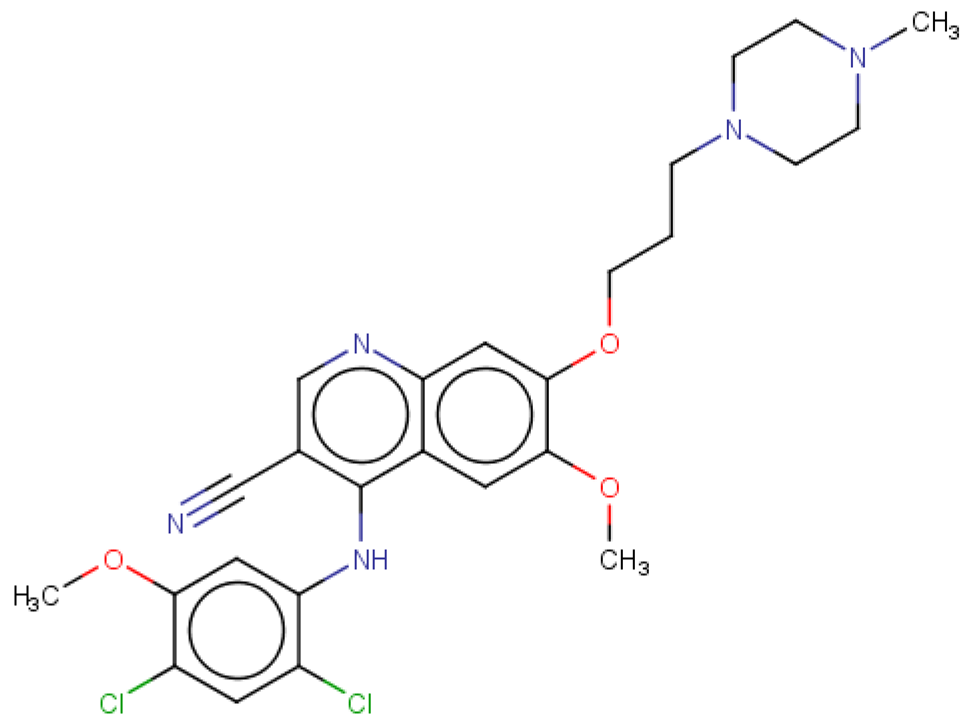

918639-10-8
Name: Bosutinib methanoate
pIC50: 4.77
Rank: 375
Classes: No defined

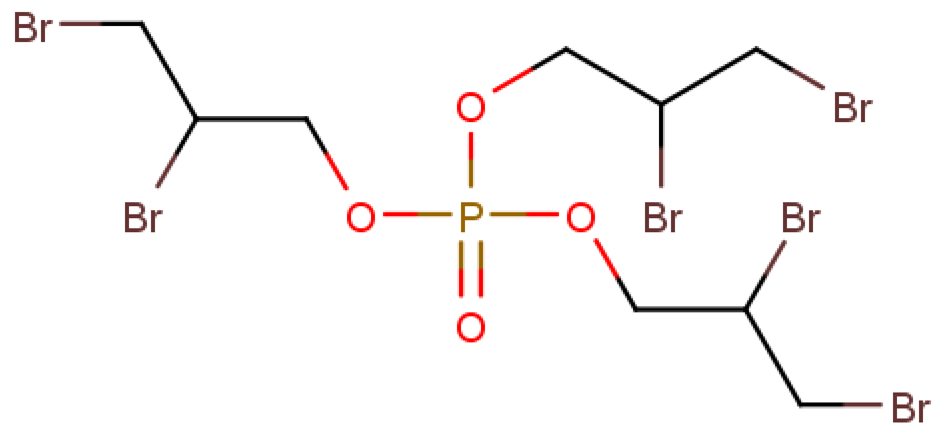

126-72-7
Name: Tris(2 3-dibromopropyl) phosphate
pIC50: 4.77
Rank: 376
Classes: TSCA

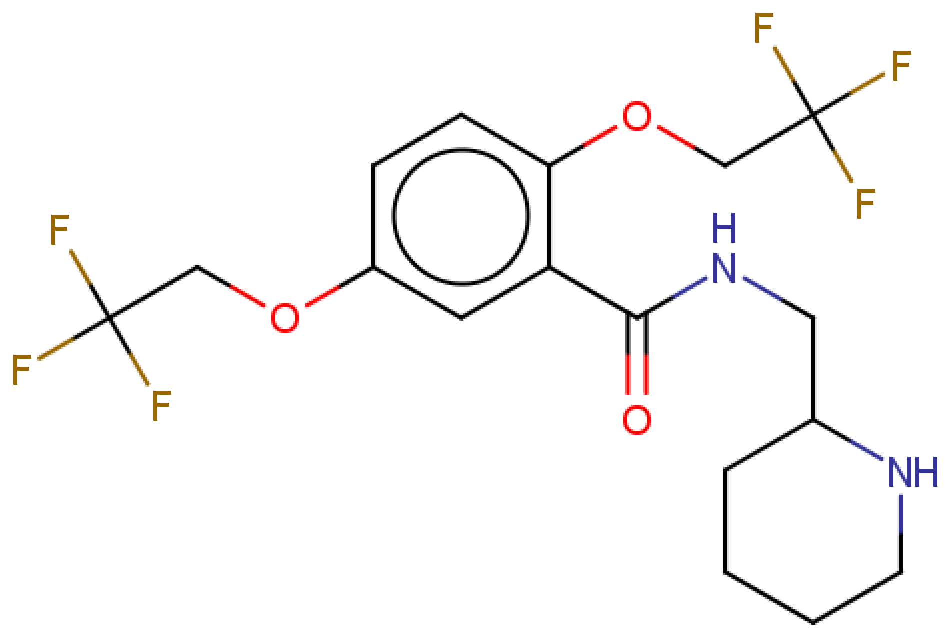

54143-56-5
Name: Flecainide acetate
pIC50: 4.77
Rank: 377
Classes: No defined

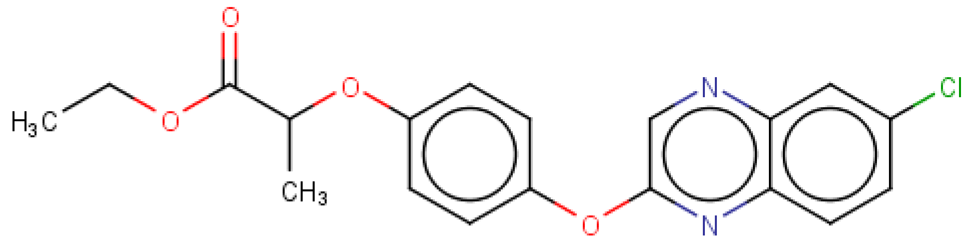

76578-14-8
Name: Quizalofop-ethyl
pIC50: 4.77
Rank: 378
Classes: No defined

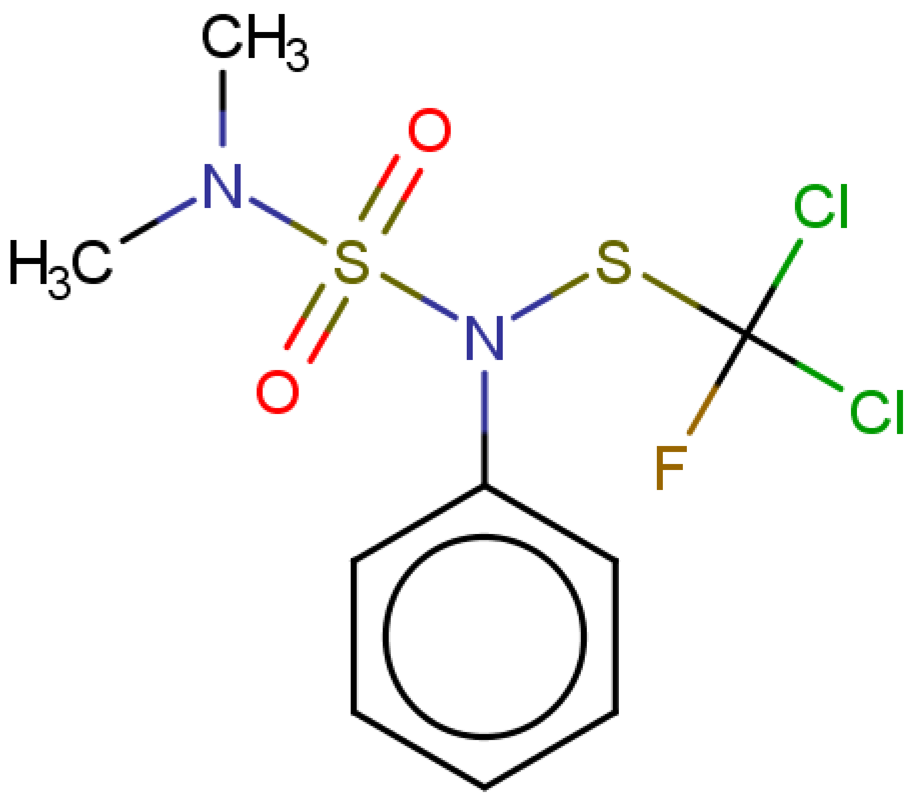

1085-98-9
Name: Dichlofluanid
pIC50: 4.76
Rank: 379
Classes: No defined

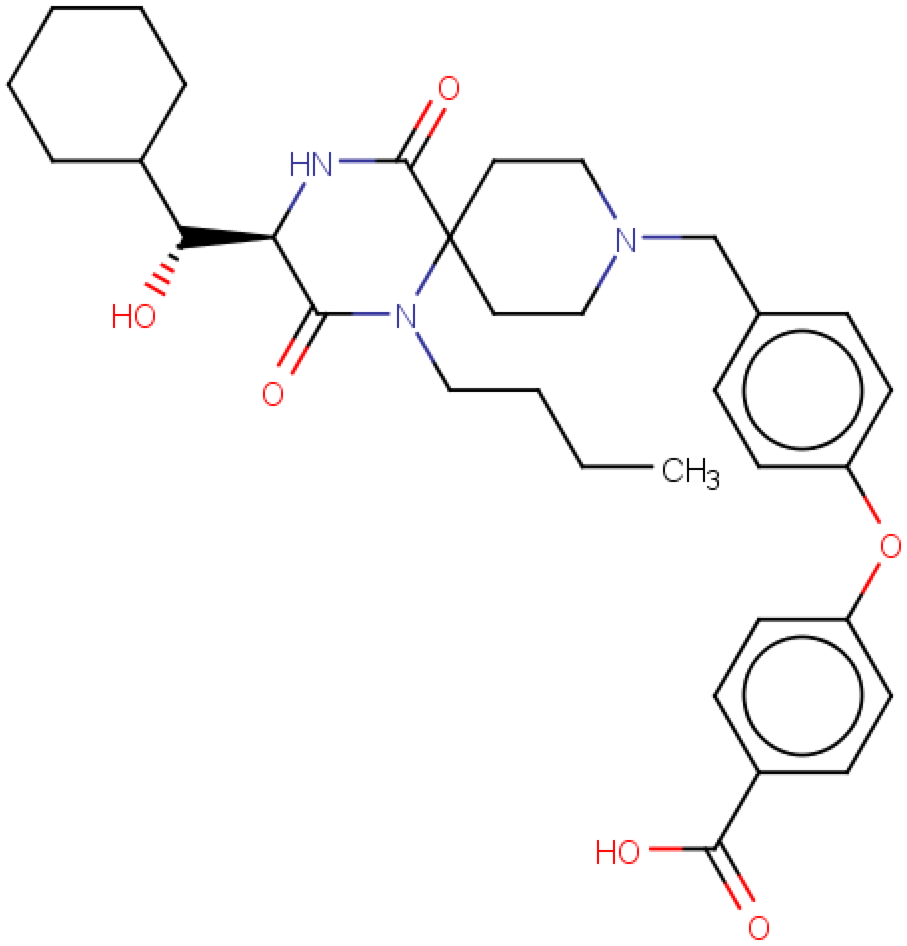

461023-63-2
Name: Aplaviroc hydrochloride
pIC50: 4.76
Rank: 380
Classes: No defined

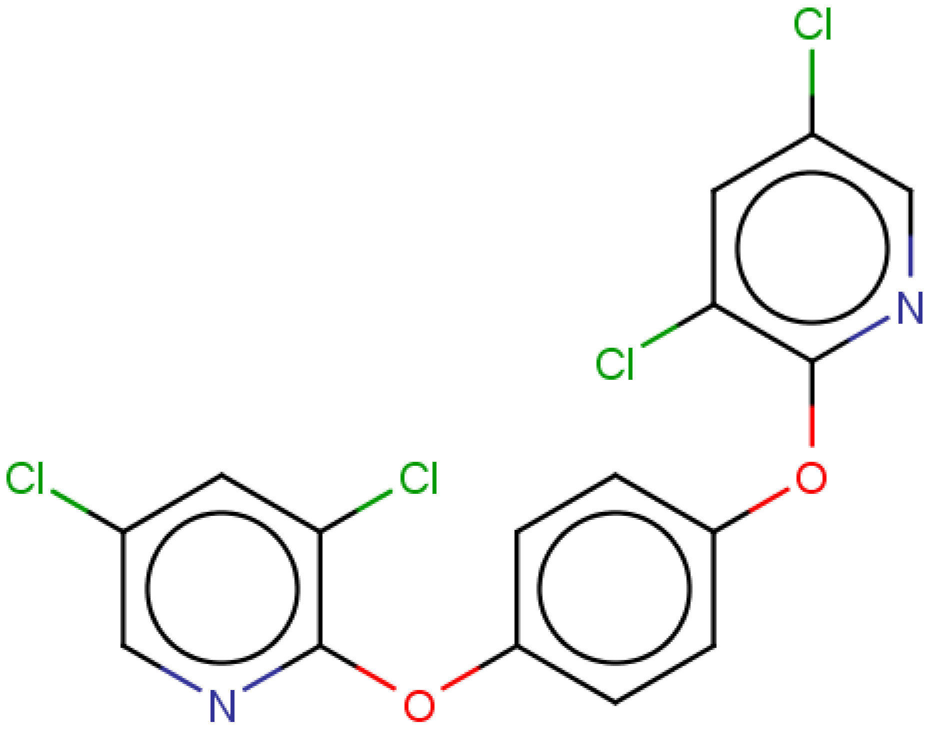

76150-91-9
Name: 1 4-Bis[2-(3 5-dichloropyridyloxy)]benz
pIC50: 4.76
Rank: 381
Classes: No defined

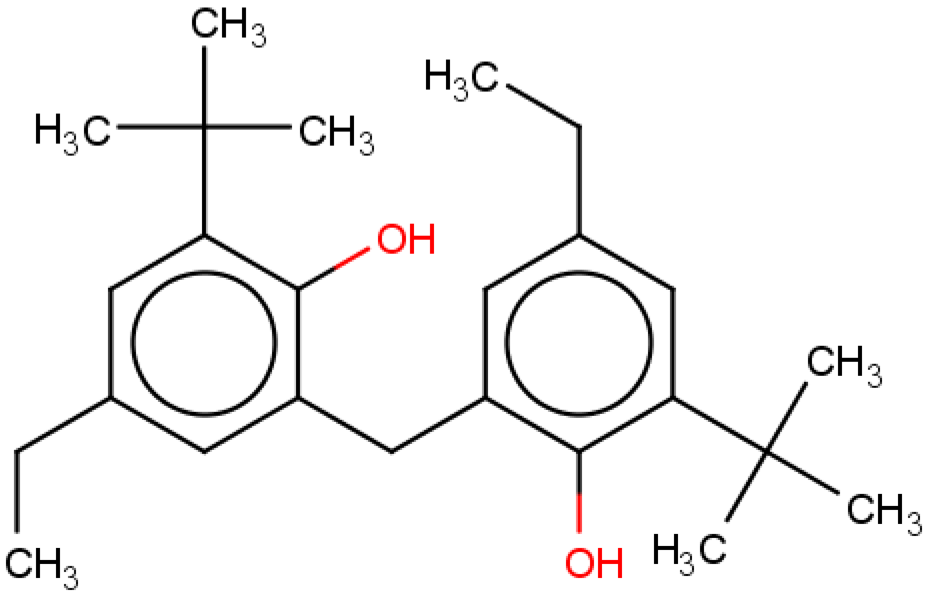

88-24-4
Name: 2 2'-Methylenebis(ethyl-6-tert-butylphenol)
pIC50: 4.76
Rank: 382
Classes: antioxidant

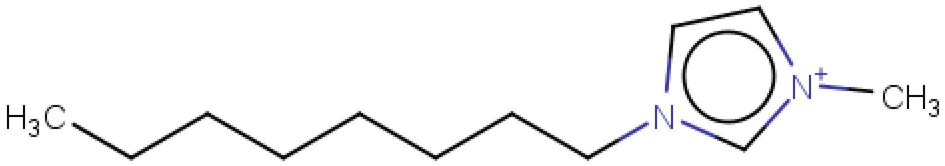

304680-36-2
Name: 1-Methyl-3-octylimidazolium hexafluorophosphate
pIC50: 4.76
Rank: 383
Classes: No defined

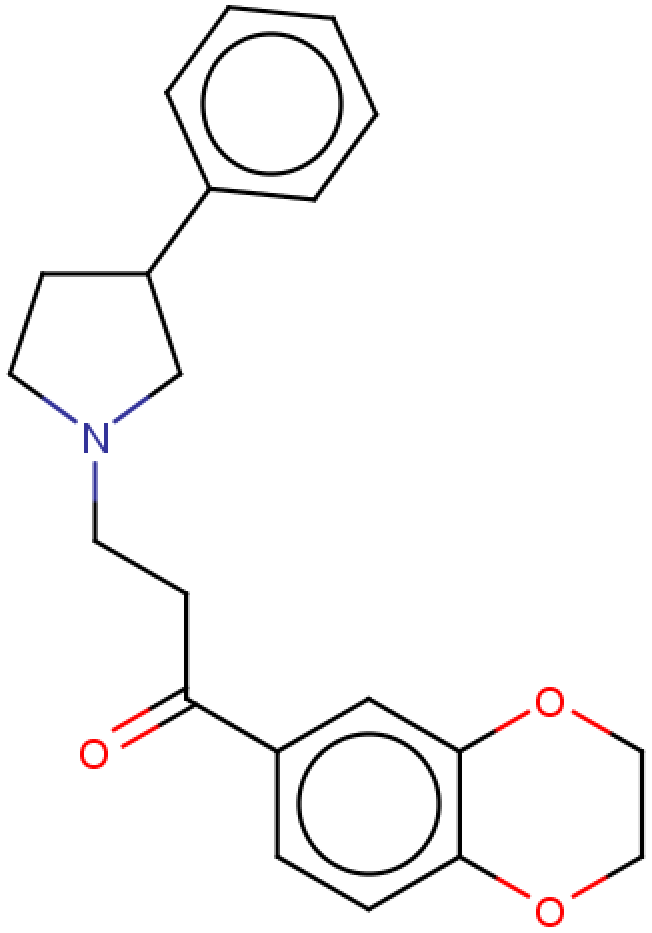

33743-96-3
Name: Proroxan
pIC50: 4.75
Rank: 384
Classes: No defined

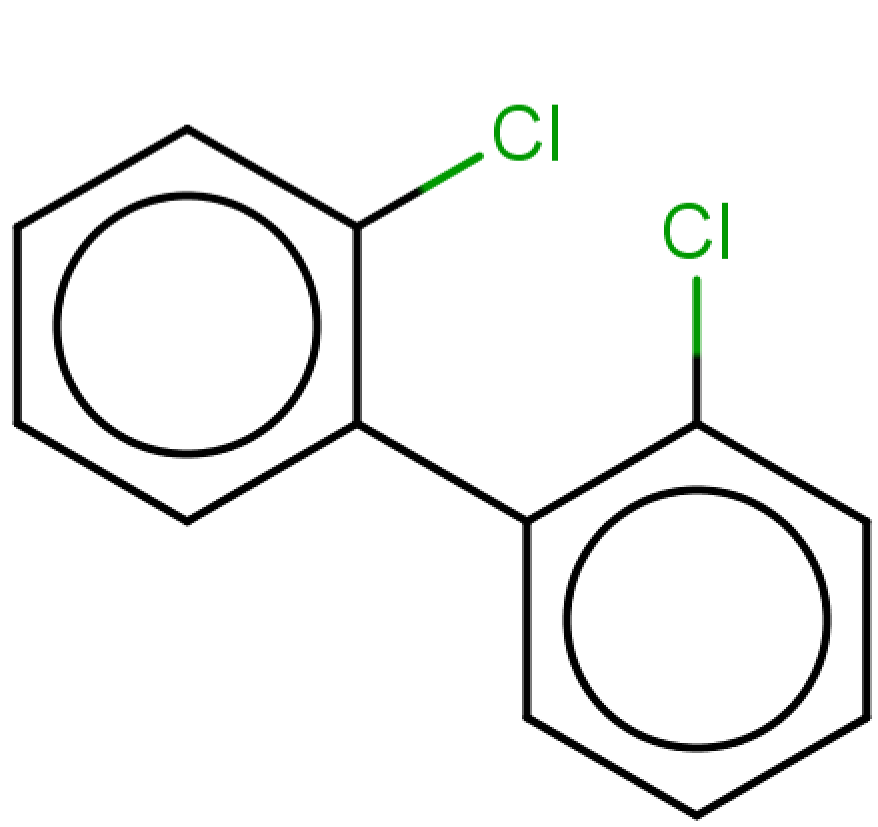

13029-08-8
Name: 2 2'-Dichlorobiphenyl
pIC50: 4.75
Rank: 385
Classes: No defined

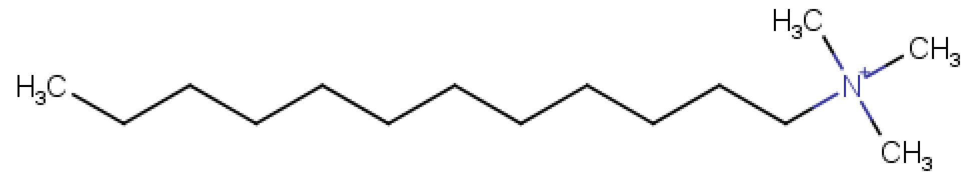

112-00-5
Name: Dodecyltrimethylammonium chloride
pIC50: 4.74
Rank: 386
Classes: hair conditioner--NA--Pesticide--TSCA

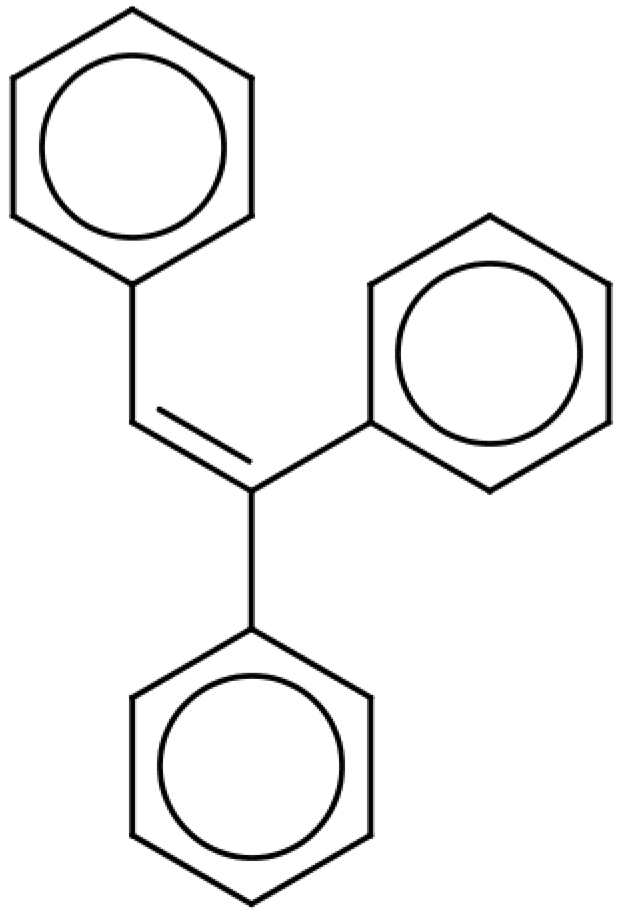

58-72-0
Name: Triphenylethylene
pIC50: 4.74
Rank: 387
Classes: No defined

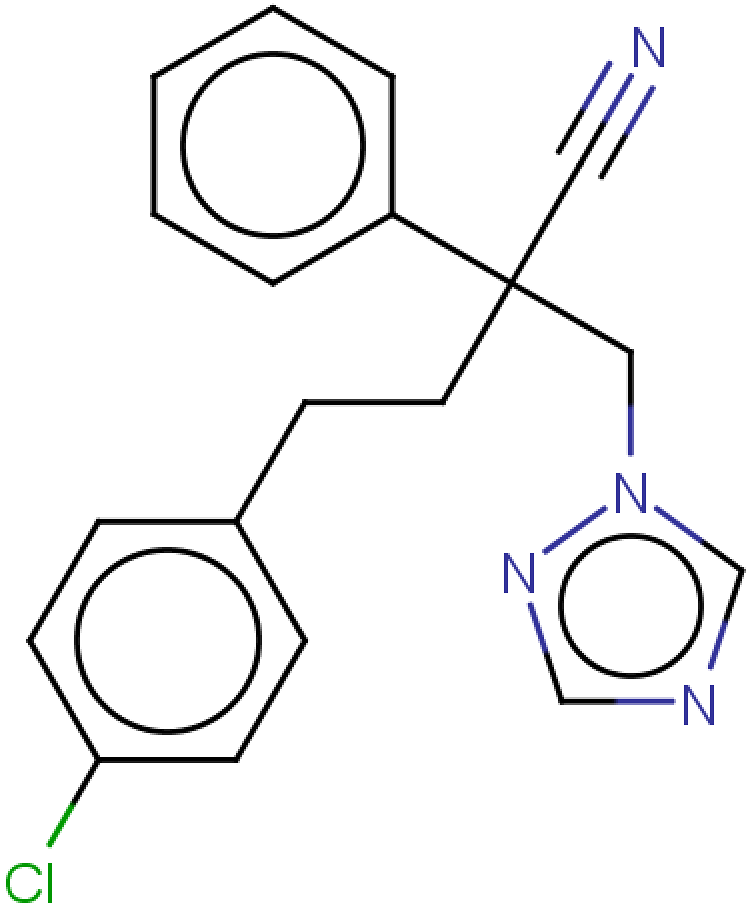

114369-43-6
Name: Fenbuconazole
pIC50: 4.74
Rank: 388
Classes: Pesticide

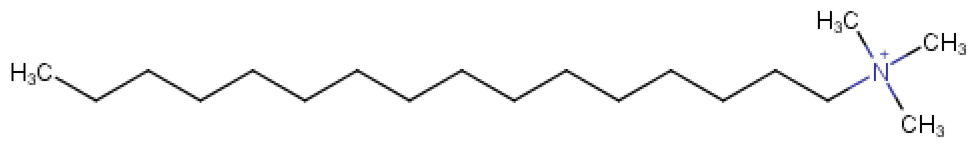

112-02-7
Name: Hexadecyl trimethyl ammonium chloride
pIC50: 4.74
Rank: 389
Classes: antimicrobial--NA--Pesticide--TSCA

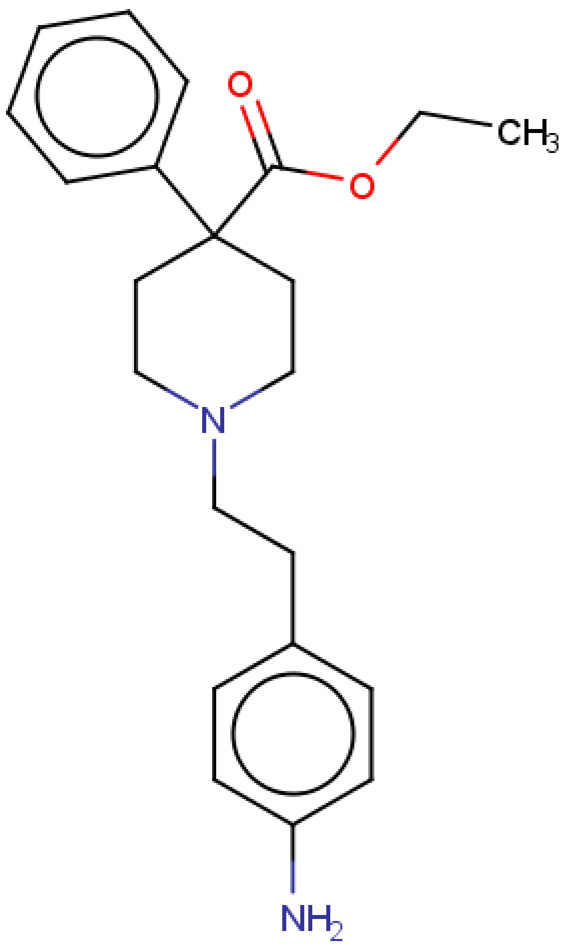

144-14-9
Name: Anileridine
pIC50: 4.74
Rank: 390
Classes: Drug

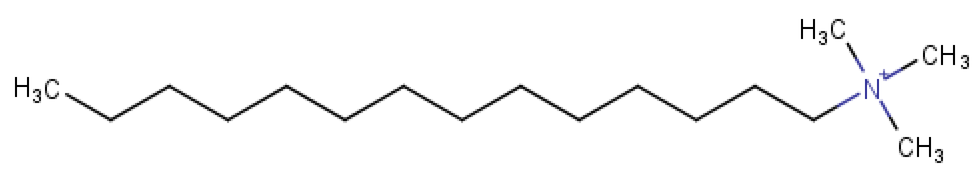

1119-97-7
Name: Tetradonium bromide
pIC50: 4.74
Rank: 391
Classes: preservative--TSCA

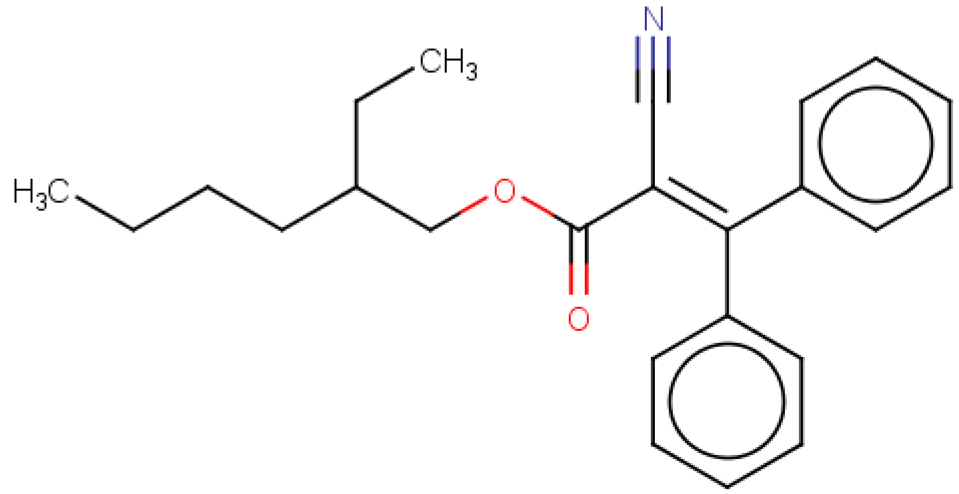

6197-30-4
Name: 2-Ethylhexyl-2-cyano-3 3-diphenylacrylate
pIC50: 4.73
Rank: 392
Classes: UV absorber--NA--TSCA

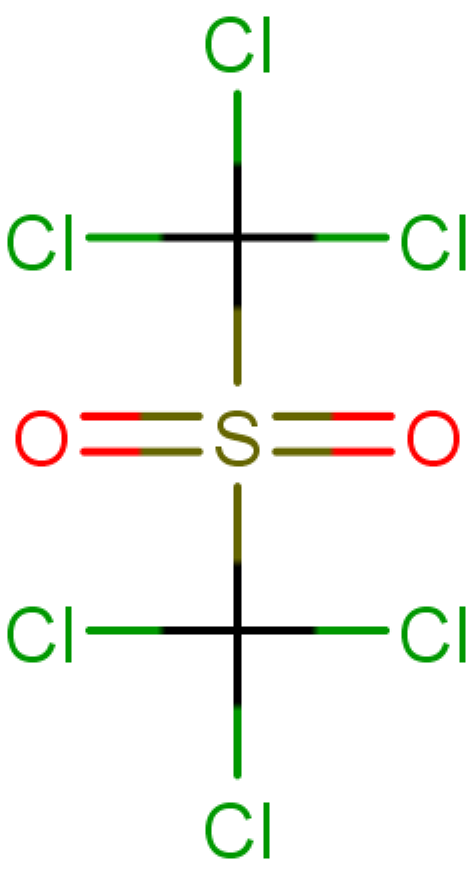

3064-70-8
Name: Bis(trichloromethyl)sulfone
pIC50: 4.73
Rank: 393
Classes: antimicrobial

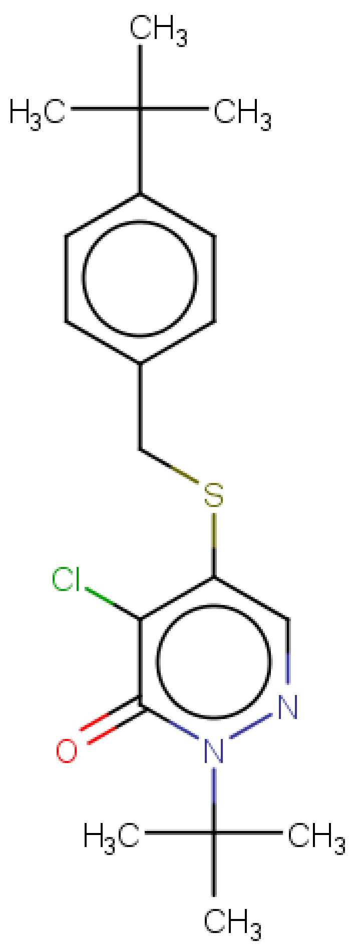

96489-71-3
Name: Pyridaben
pIC50: 4.73
Rank: 394
Classes: Pesticide

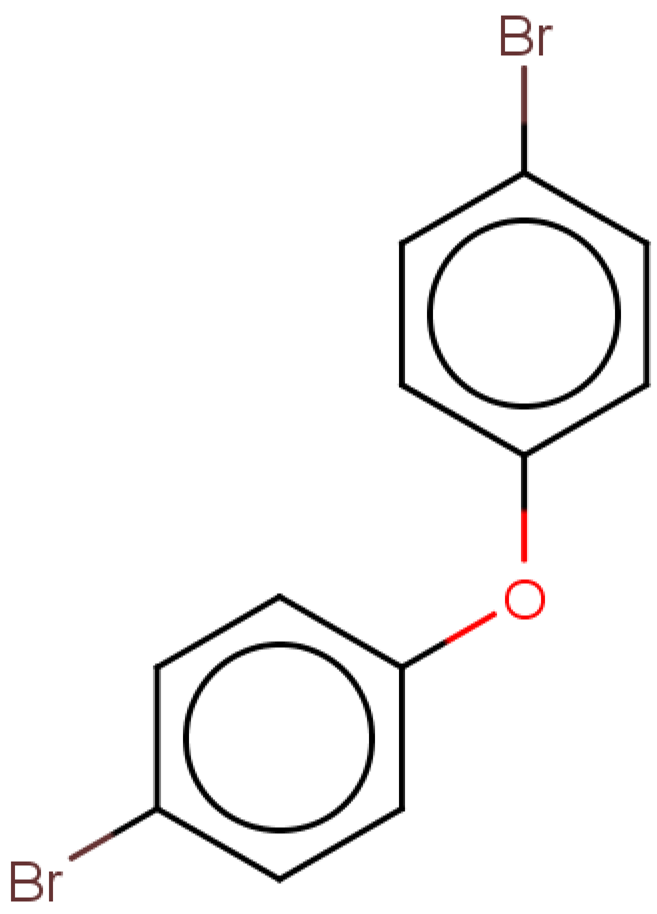

2050-47-7
Name: 4 4'-Dibromodiphenyl ether
pIC50: 4.73
Rank: 395
Classes: No defined

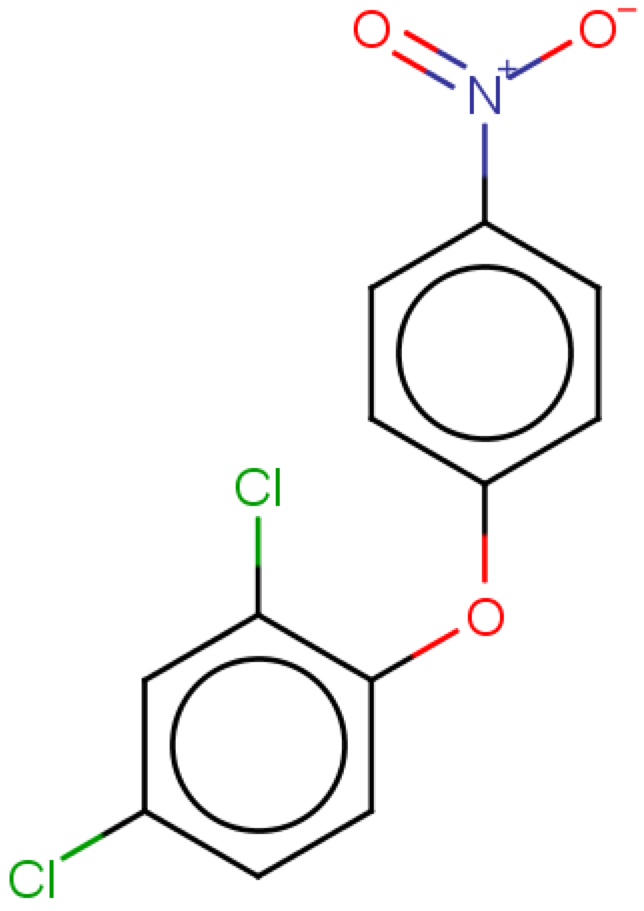

1836-75-5
Name: Nitrofen
pIC50: 4.72
Rank: 396
Classes: TSCA

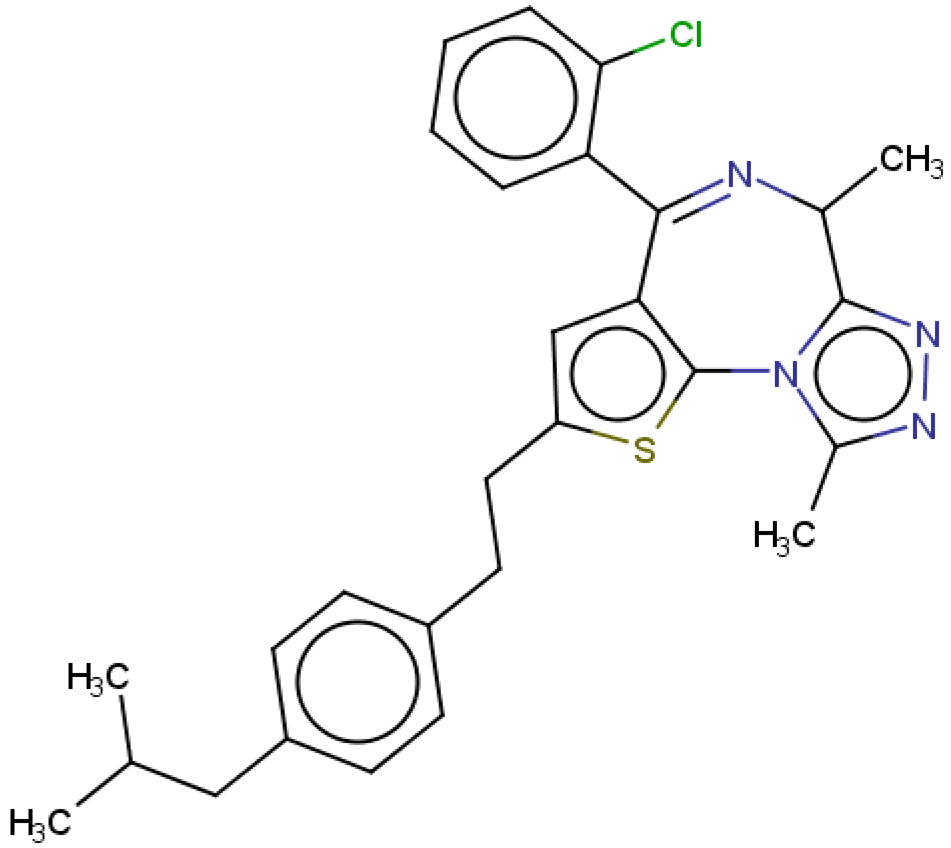

117279-73-9
Name: Israpafant
pIC50: 4.72
Rank: 397
Classes: No defined

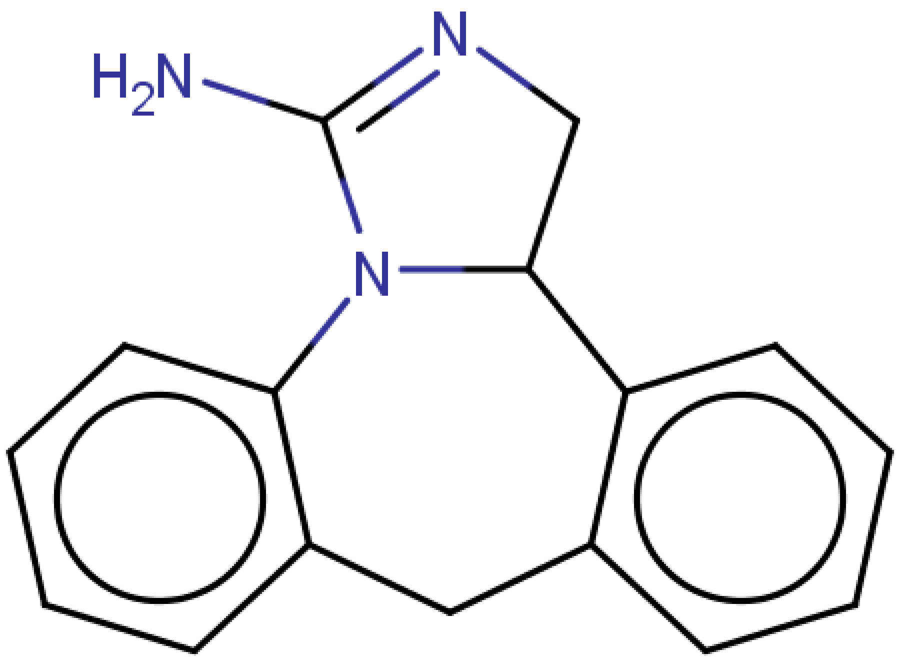

108929-04-0
Name: Epinastine hydrochloride
pIC50: 4.72
Rank: 398
Classes: No defined

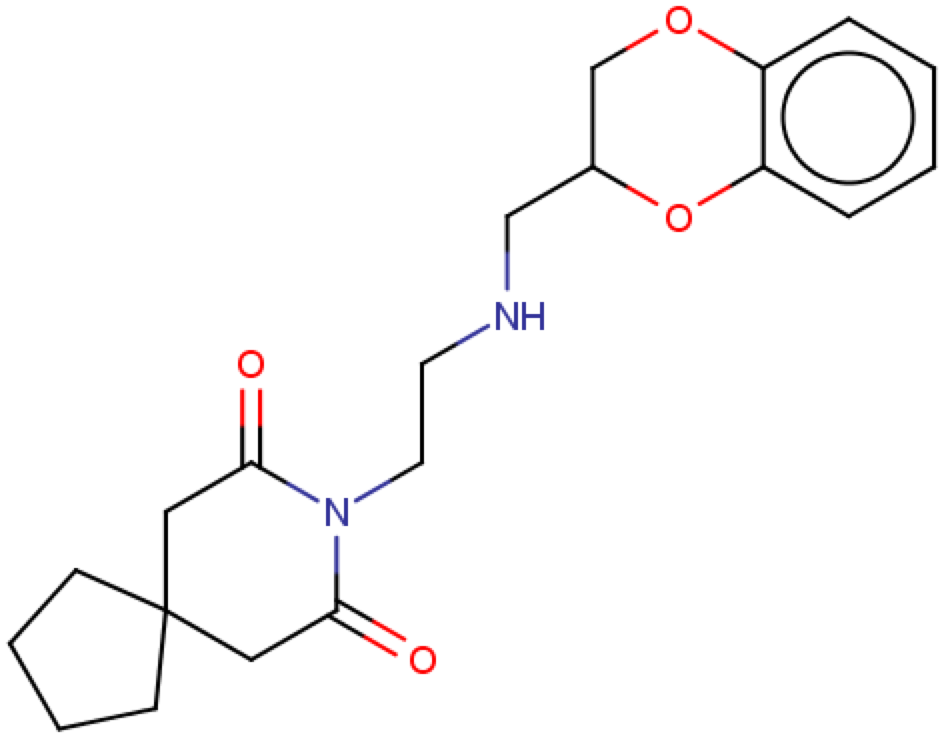

124756-23-6
Name: Binospirone mesylate
pIC50: 4.72
Rank: 399
Classes: No defined

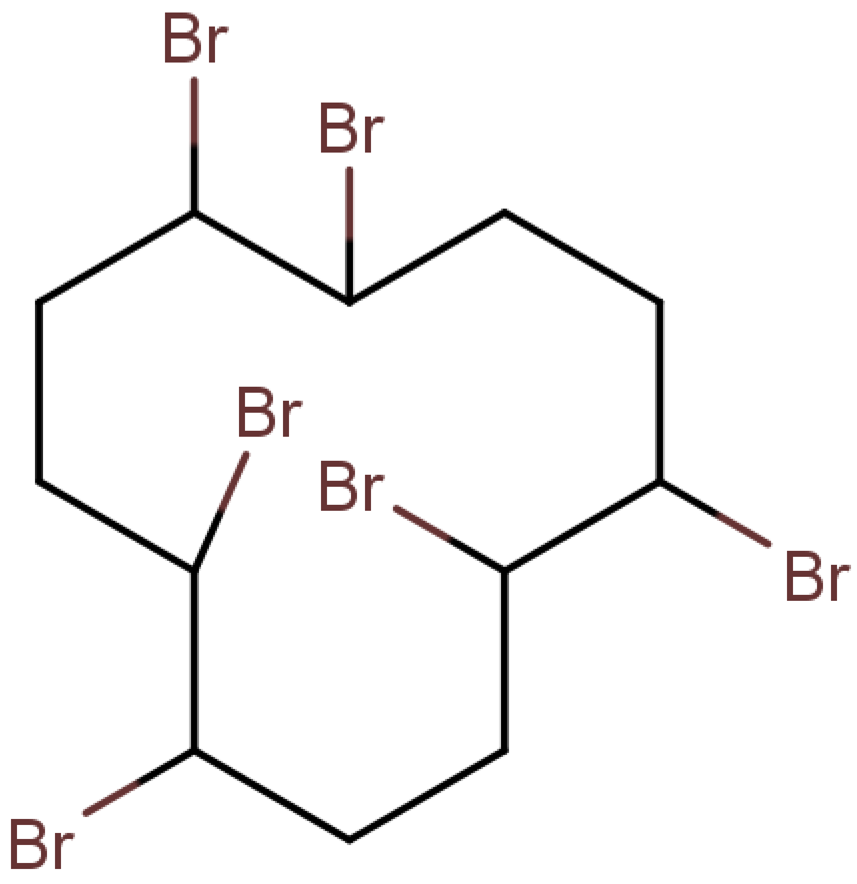

3194-55-6
Name: 1 2 5 6 9 10-Hexabromocyclododecane
pIC50: 4.72
Rank: 400
Classes: flame retardant--TSCA

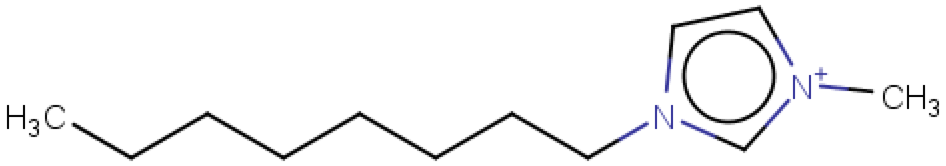

64697-40-1
Name: 1-Methyl-3-octylimidazolium chloride
pIC50: 4.71
Rank: 401
Classes: No defined

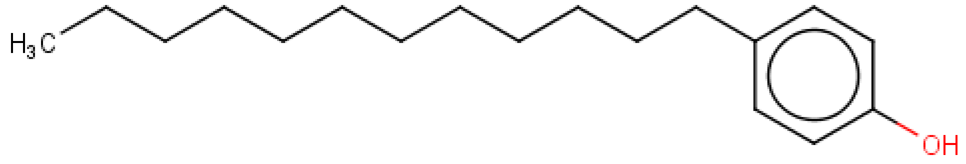

104-43-8
Name: 4-Dodecylphenol
pIC50: 4.71
Rank: 402
Classes: TSCA

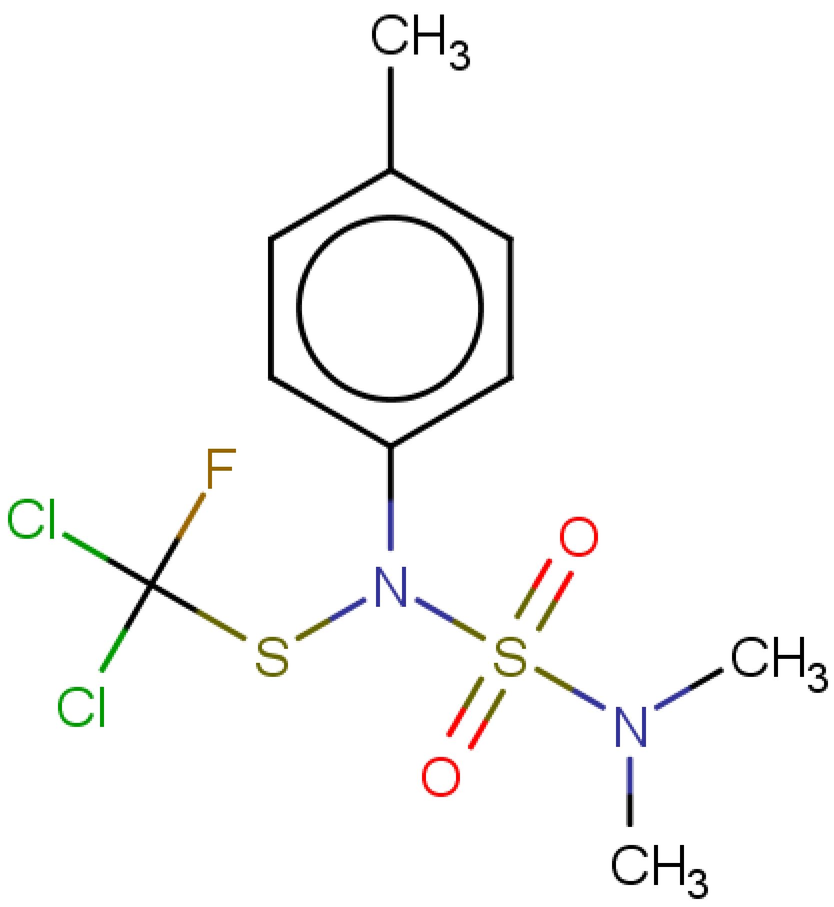

731-27-1
Name: Tolylfluanid
pIC50: 4.71
Rank: 403
Classes: No defined

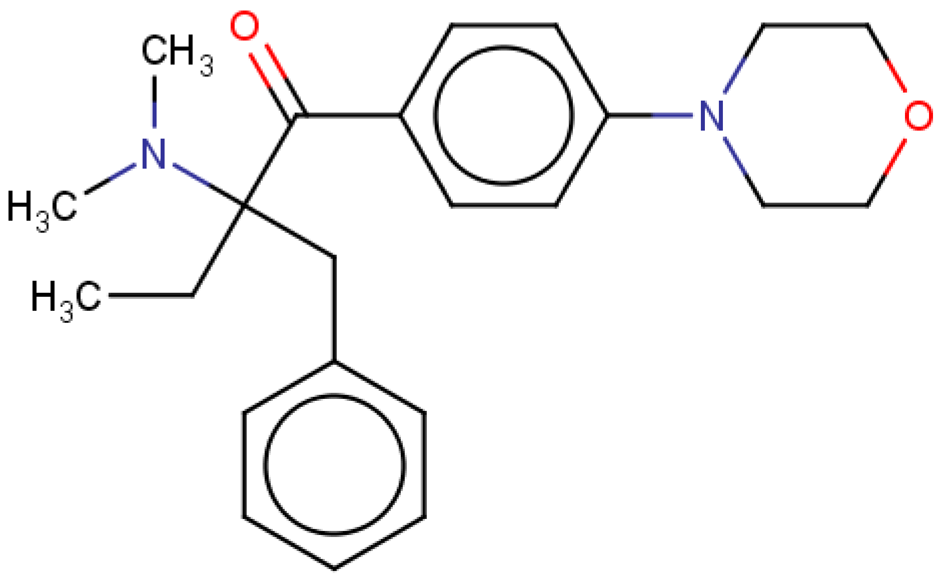

119313-12-1
Name: 2-Benzyl-2-(dimethylamino)-1-[4-(morp[illegible]
pIC50: 4.71
Rank: 404
Classes: UV absorber--TSCA

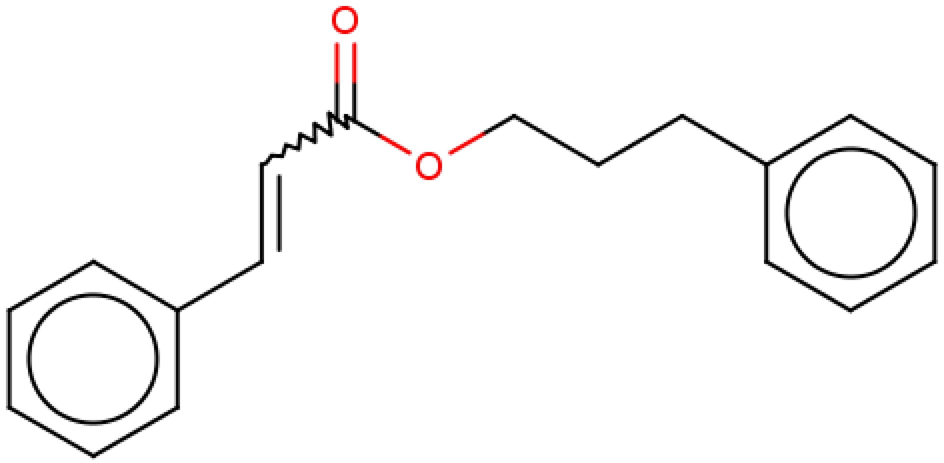

122-68-9
Name: 3-Phenylpropyl cinnamate
pIC50: 4.71
Rank: 405
Classes: fragrance

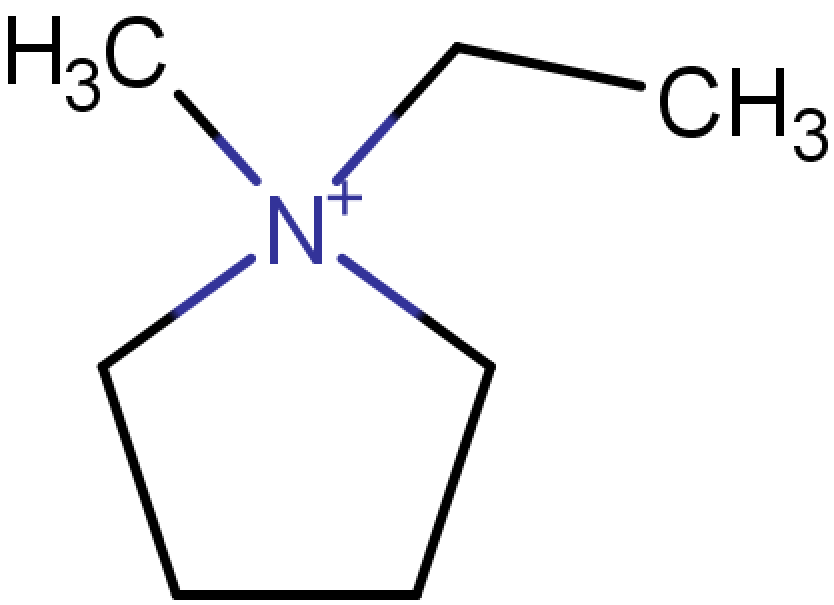

4186-68-9
Name: 1-Ethyl-1-methylpyrrolidinium iodide
pIC50: 4.71
Rank: 406
Classes: No defined

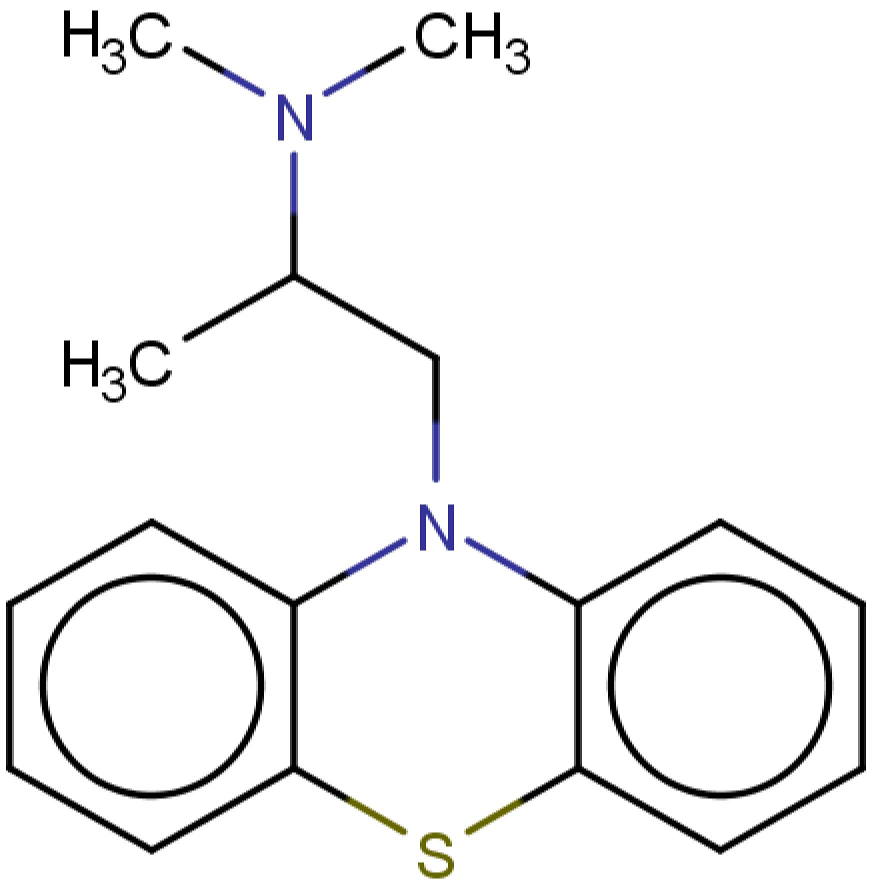

60-87-7
Name: Promethazine
pIC50: 4.7
Rank: 407
Classes: Drug

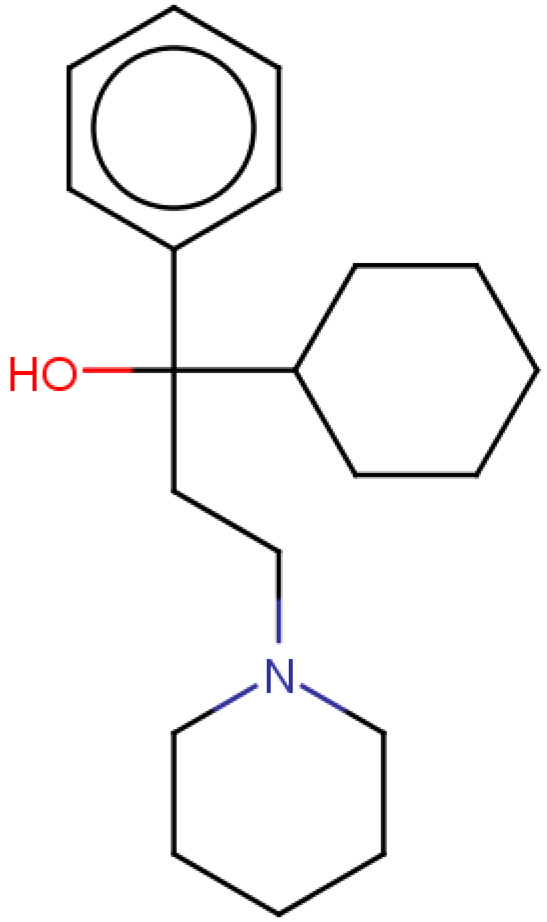

52-49-3
Name: Trihexyphenidyl hydrochloride
pIC50: 4.7
Rank: 408
Classes: No defined

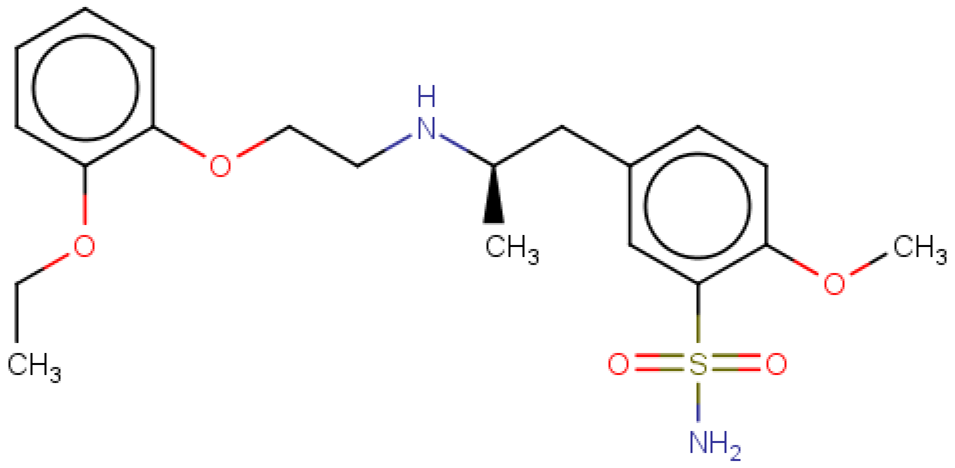

106463-17-6
Name: Tamsulosin hydrochloride
pIC50: 4.7
Rank: 409
Classes: No defined

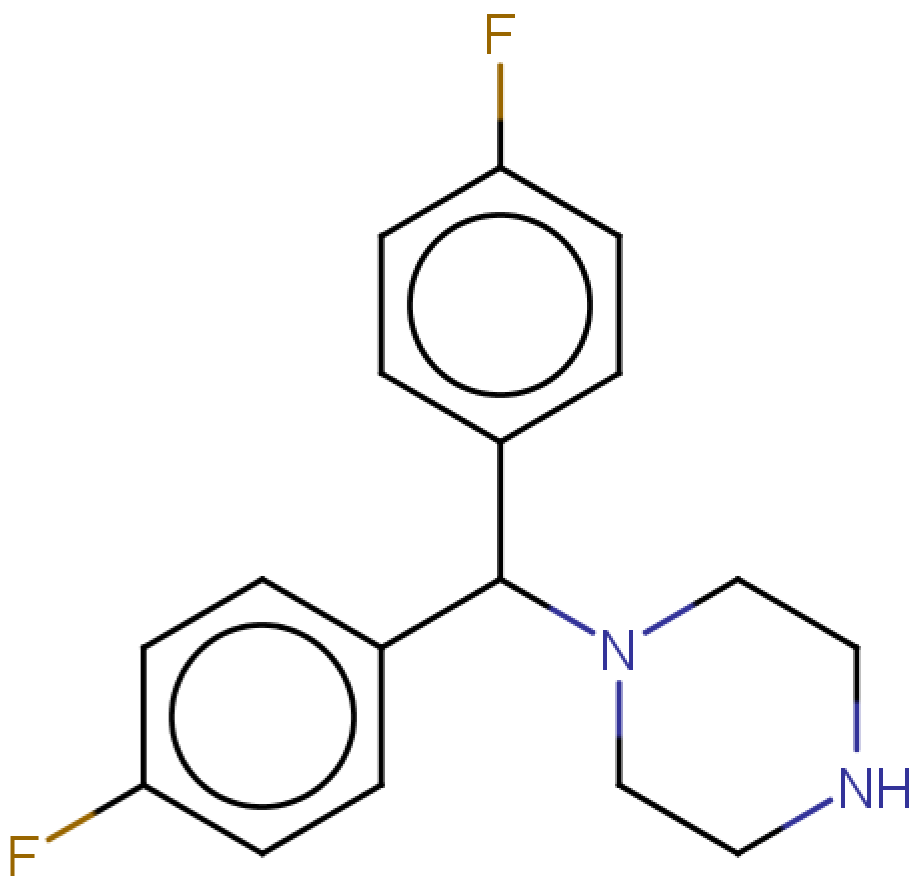

27469-60-9
Name: 1-(Bis(4-fluorophenyl)methyl)piperazine
pIC50: 4.7
Rank: 410
Classes: No defined

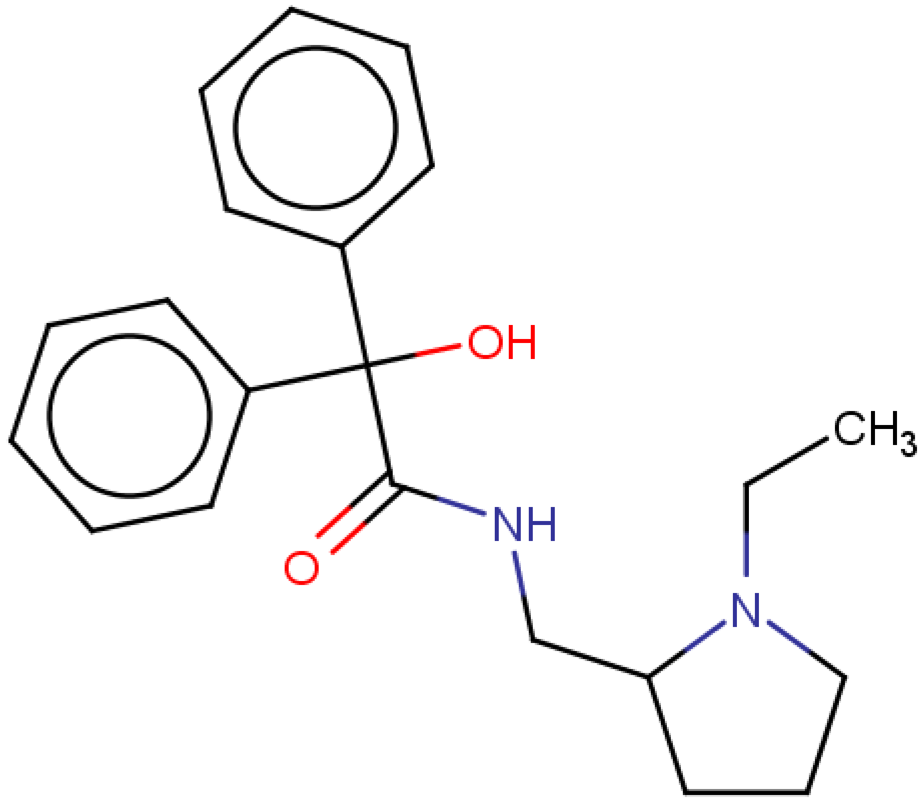

66304-03-8
Name: Epicainide
pIC50: 4.7
Rank: 411
Classes: No defined

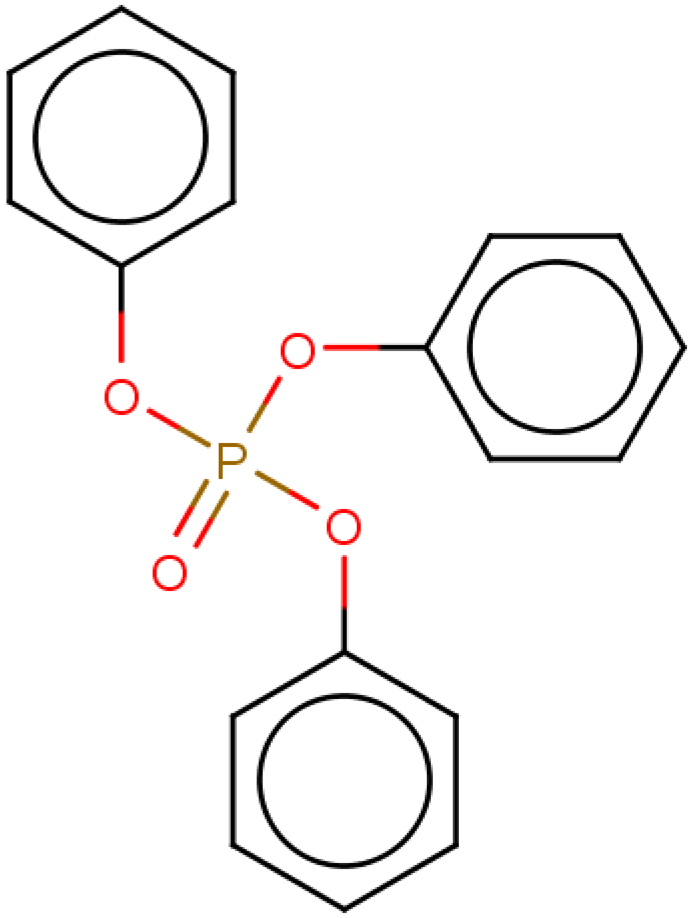

26444-49-5
Name: Cresyl diphenyl phosphate
pIC50: 4.7
Rank: 412
Classes: flame retardant

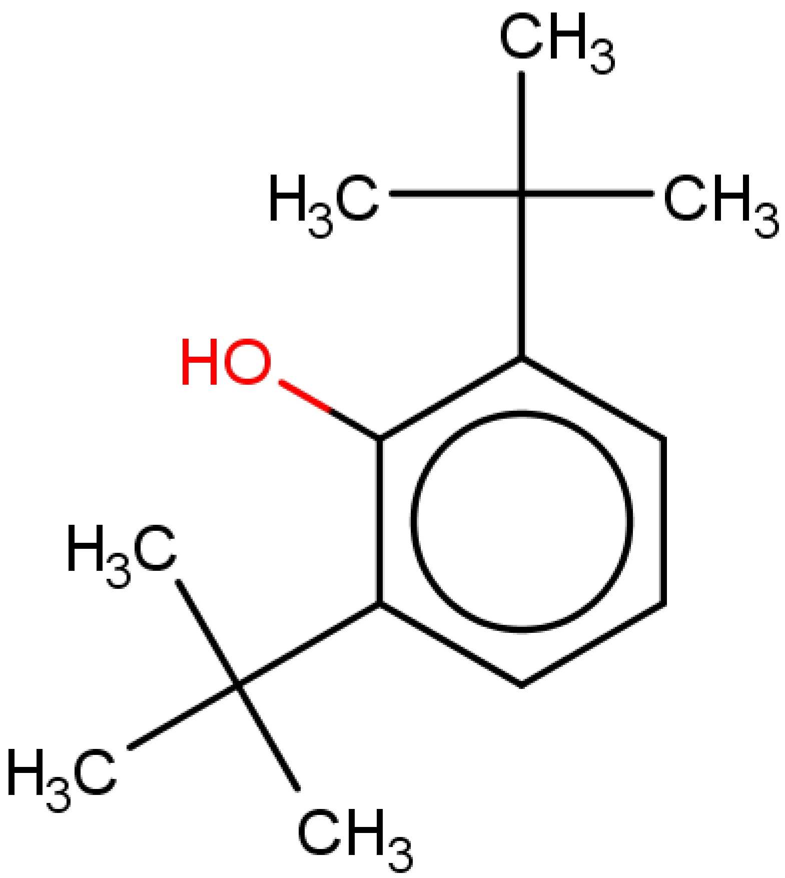

128-39-2
Name: 2 6-Di-tert-butylphenol
pIC50: 4.7
Rank: 413
Classes: fragrance--TSCA

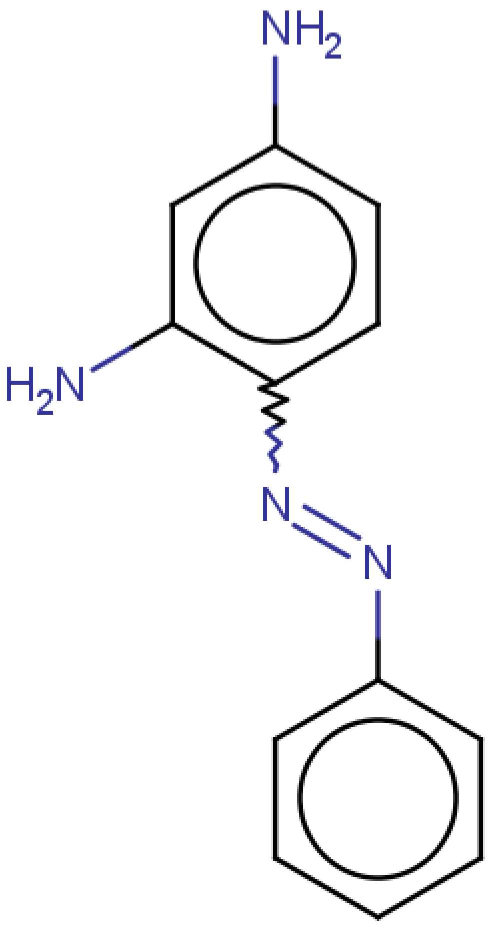

532-82-1
Name: C.I. Basic Orange 2
pIC50: 4.7
Rank: 414
Classes: No defined

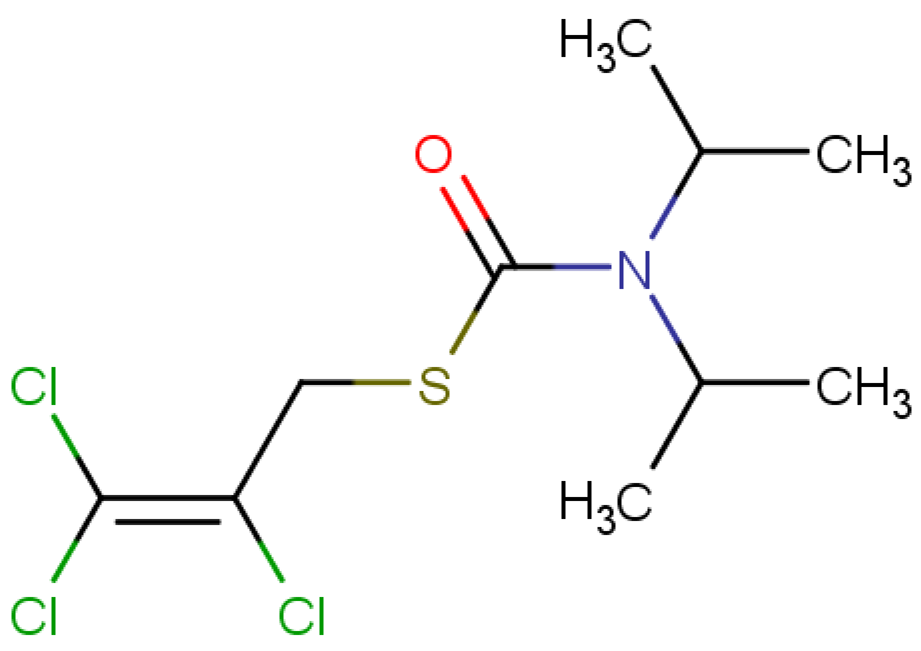

2303-17-5
Name: Tri-allate
pIC50: 4.7
Rank: 415
Classes: Pesticide

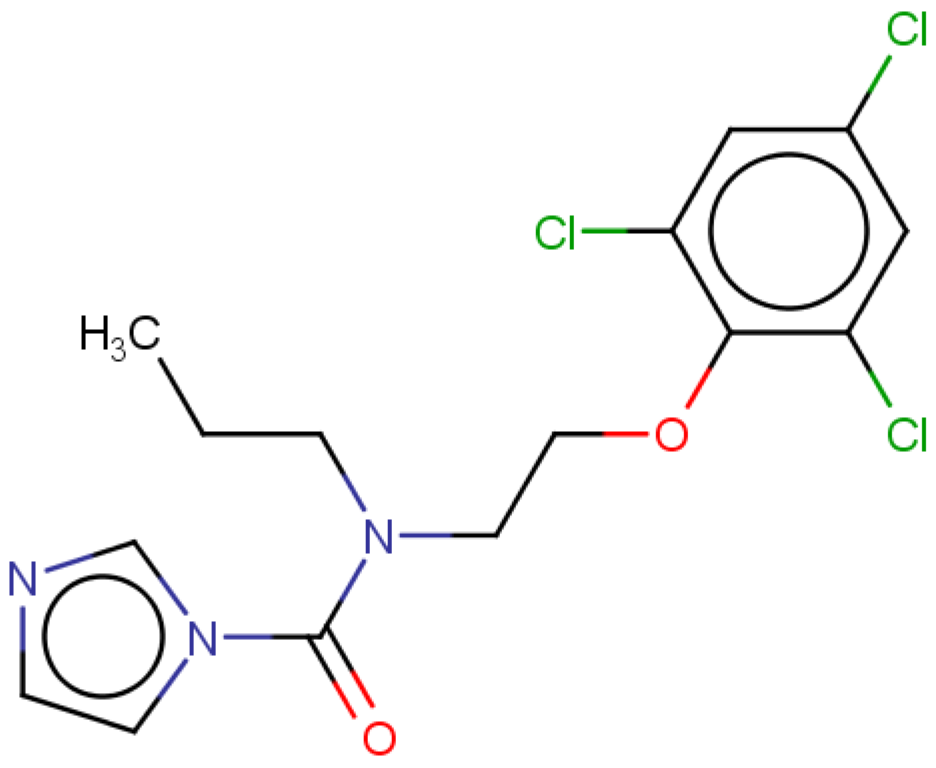

67747-09-5
Name: Prochloraz
pIC50: 4.69
Rank: 416
Classes: No defined

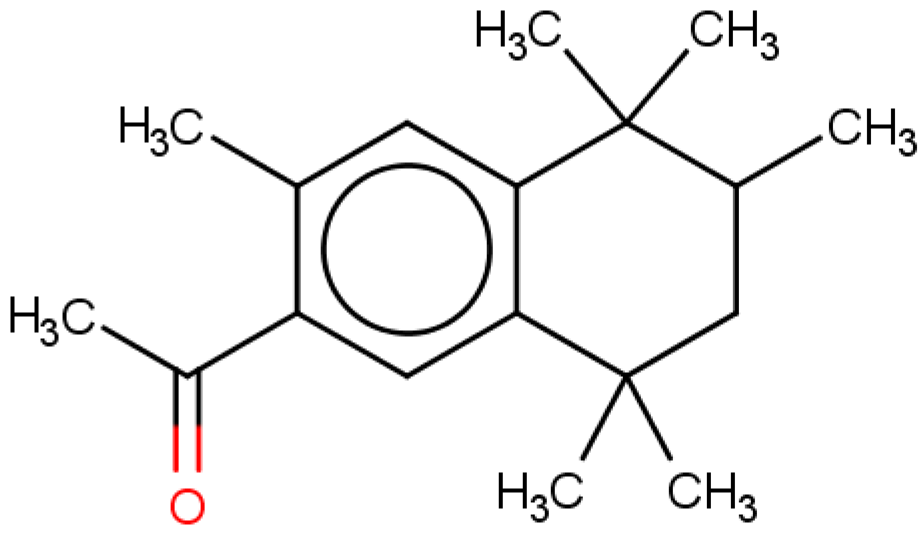

21145-77-7
Name: 6-Acetyl-1 1 2 4 4 7-hexamethyltetralin
pIC50: 4.69
Rank: 417
Classes: masking agent

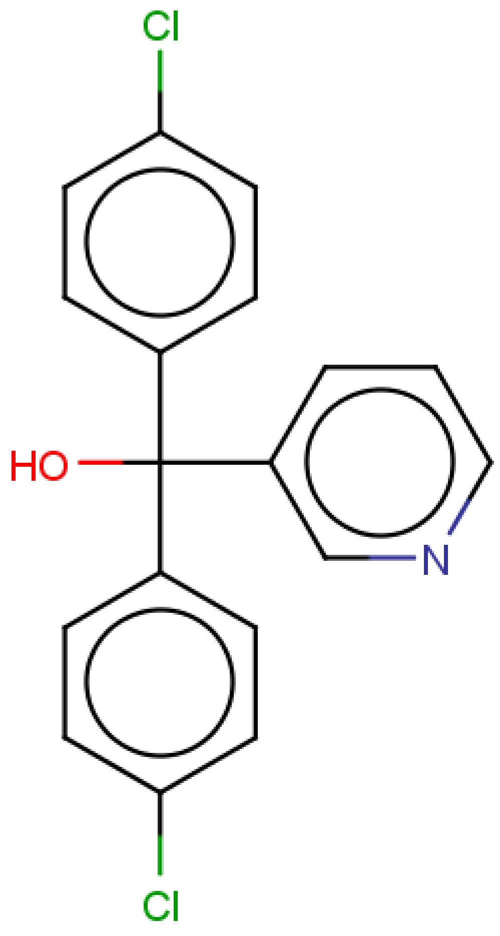

17781-31-6
Name: Parinol
pIC50: 4.69
Rank: 418
Classes: No defined

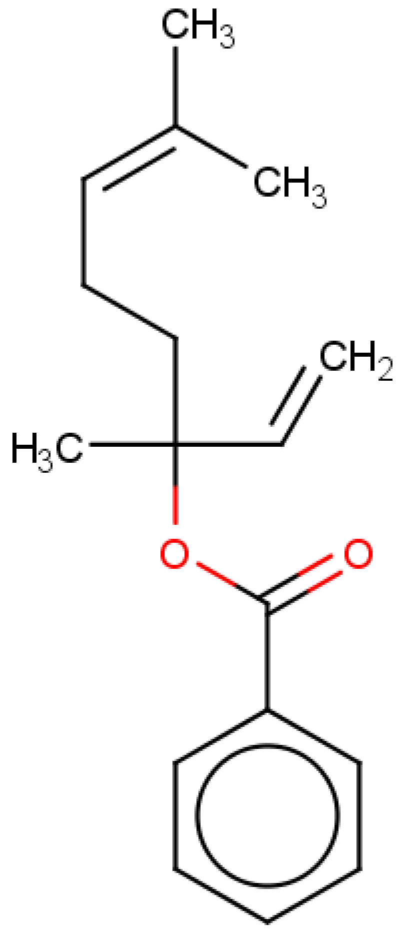

126-64-7
Name: Linalyl benzoate
pIC50: 4.69
Rank: 419
Classes: fragrance

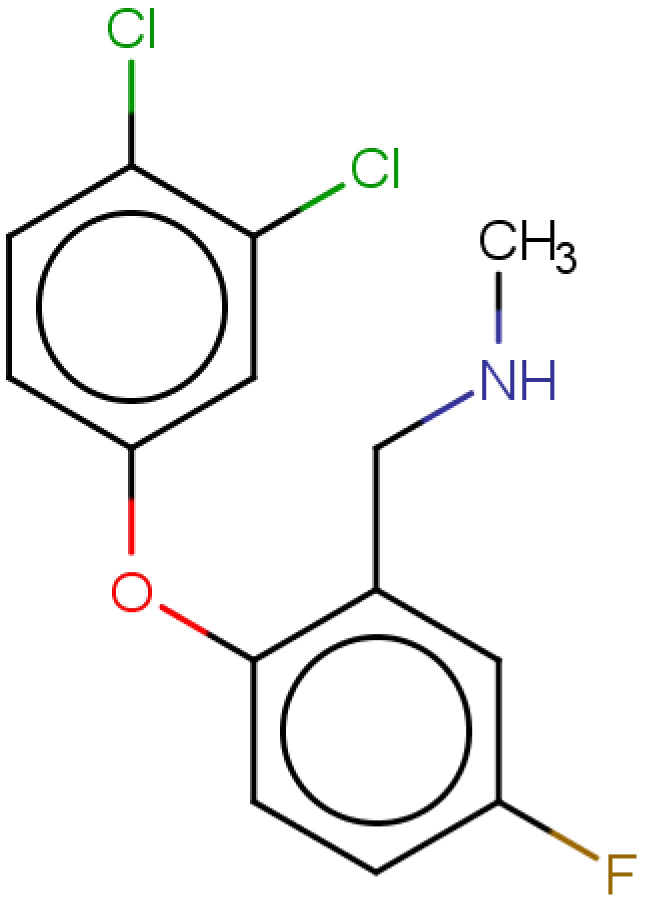

289716-94-5
Name: CP-607366
pIC50: 4.69
Rank: 420
Classes: No defined

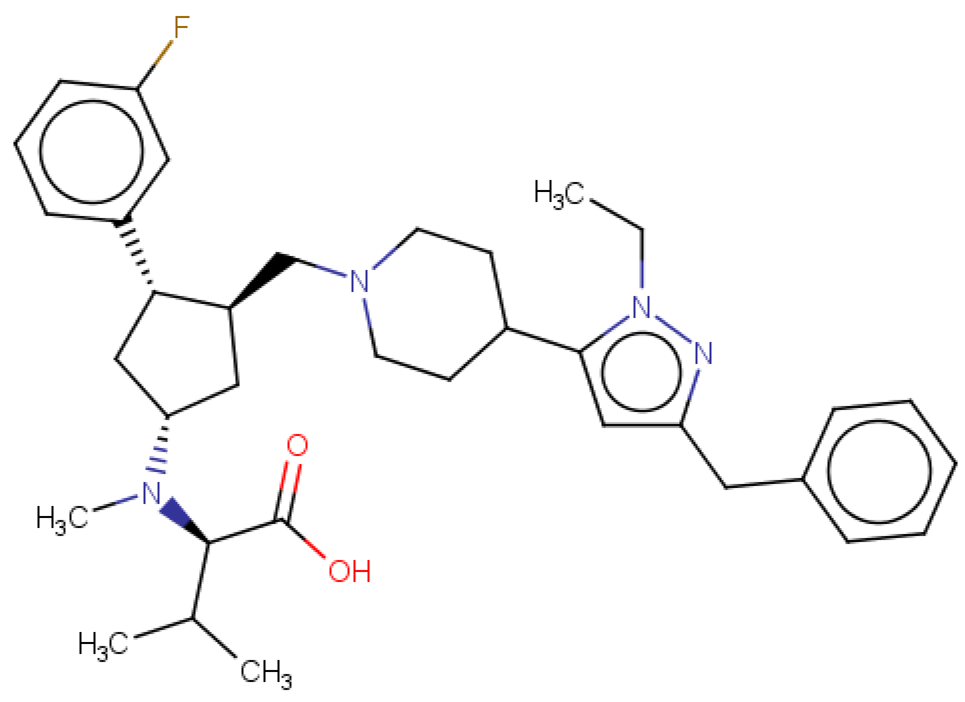

313994-79-5
Name: MK-578
pIC50: 4.69
Rank: 421
Classes: No defined

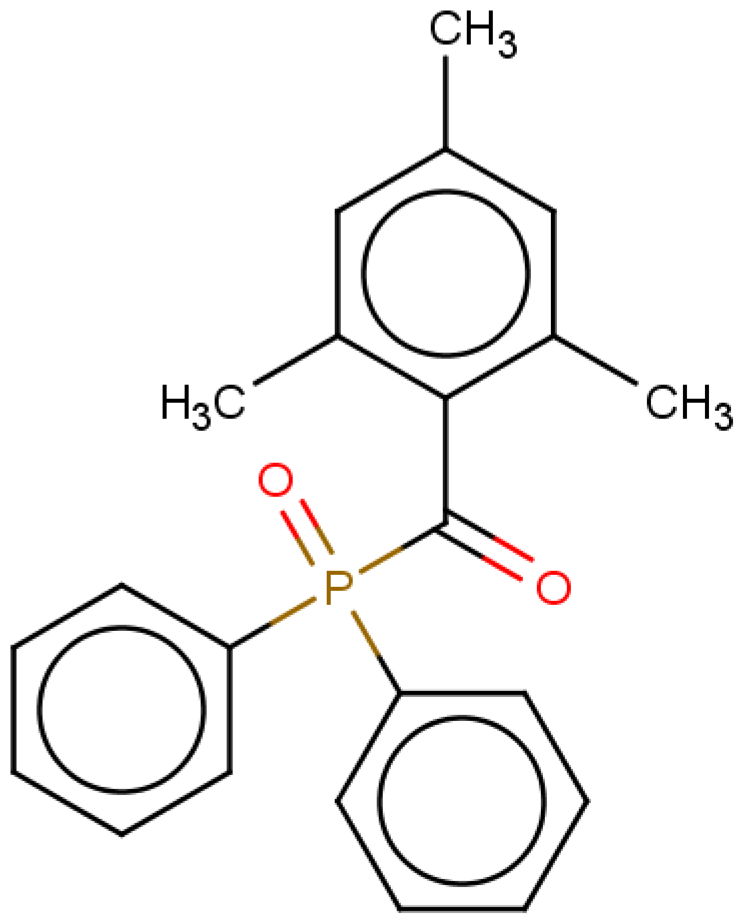

75980-60-8
Name: Diphenyl(2 4 6-trimethylbenzoyl)phosphine oxide
pIC50: 4.69
Rank: 422
Classes: crosslinker--TSCA

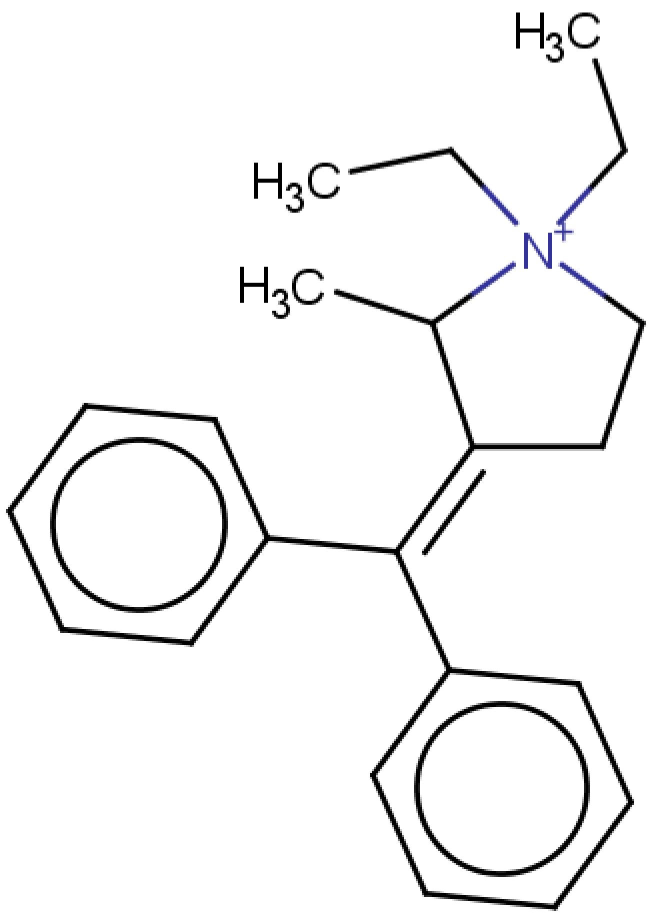

4630-95-9
Name: Prifinium bromide
pIC50: 4.69
Rank: 423
Classes: No defined

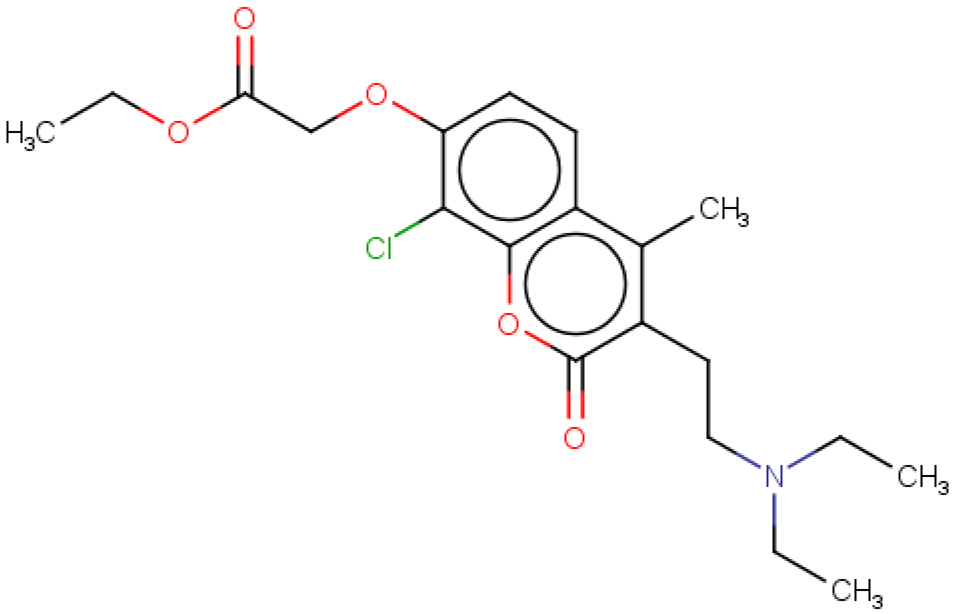

74697-28-2
Name: Cloricromen hydrochloride
pIC50: 4.69
Rank: 424
Classes: No defined

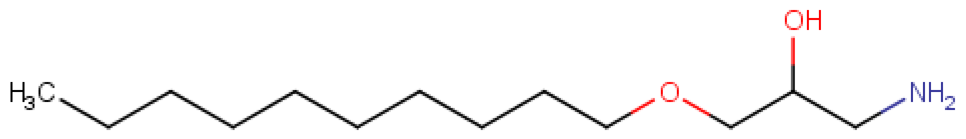

60812-23-9
Name: Decominol hydrochloride
pIC50: 4.69
Rank: 425
Classes: No defined

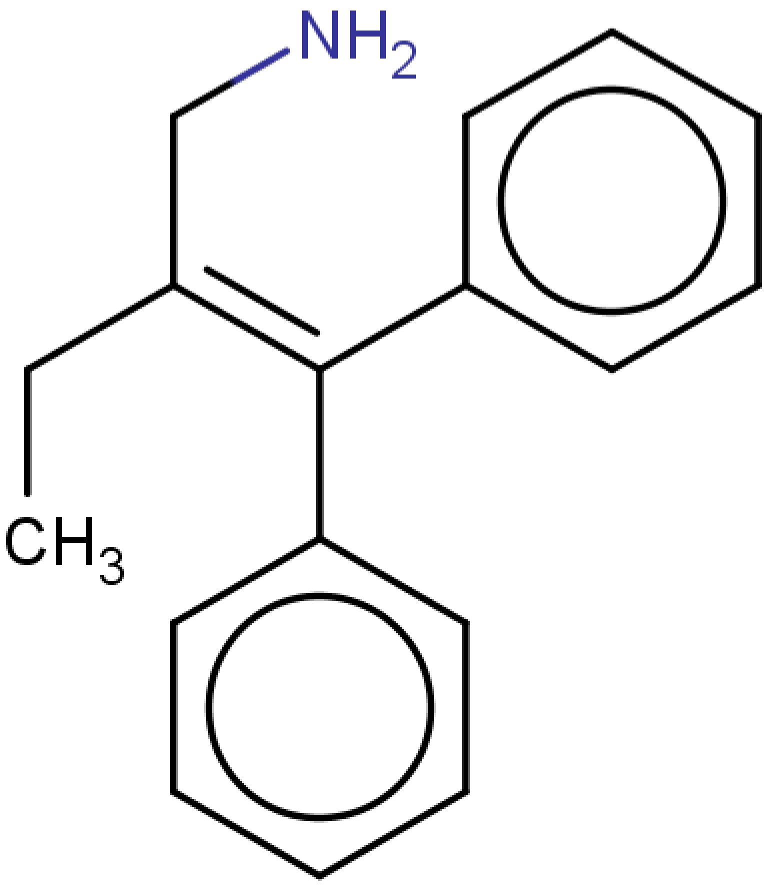

1146-95-8
Name: Etifelmine hydrochloride
pIC50: 4.69
Rank: 426
Classes: No defined

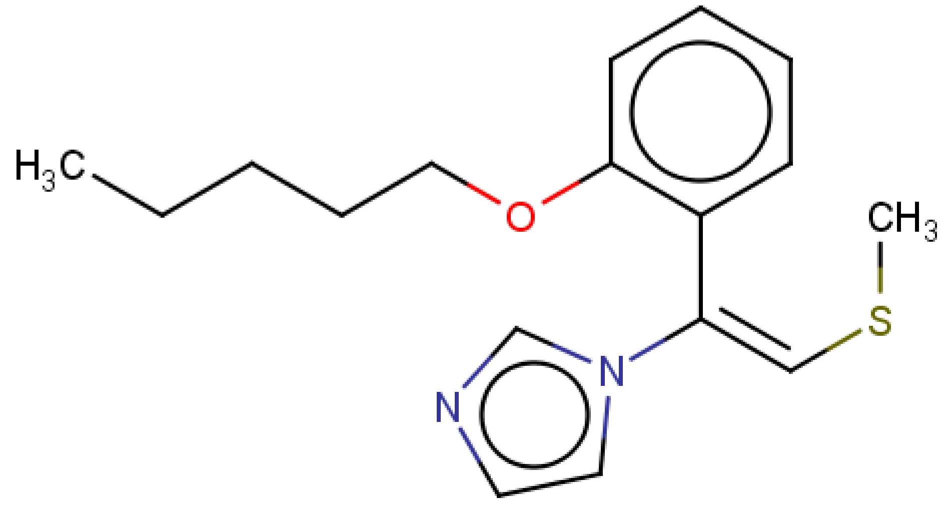

130726-68-0
Name: Neticonazole
pIC50: 4.69
Rank: 427
Classes: No defined

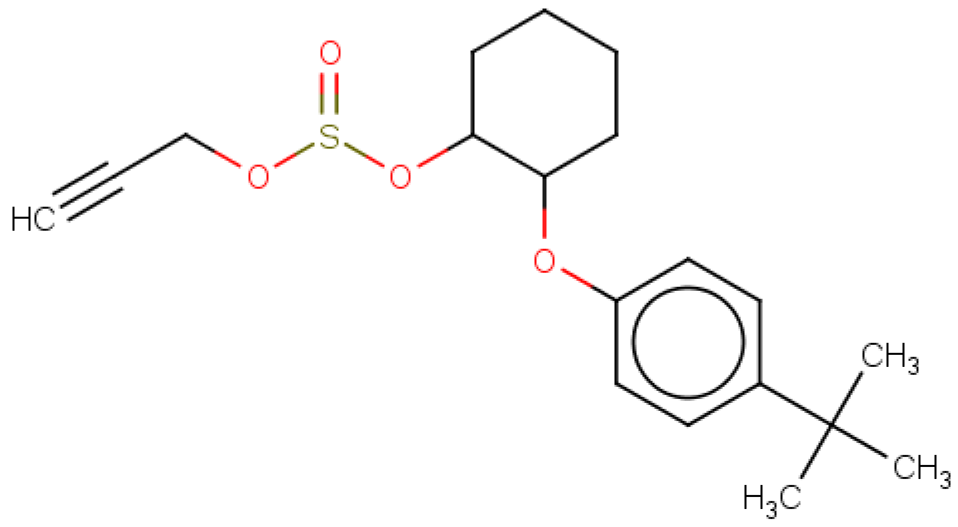

2312-35-8
Name: Propargite
pIC50: 4.68
Rank: 428
Classes: Pesticide

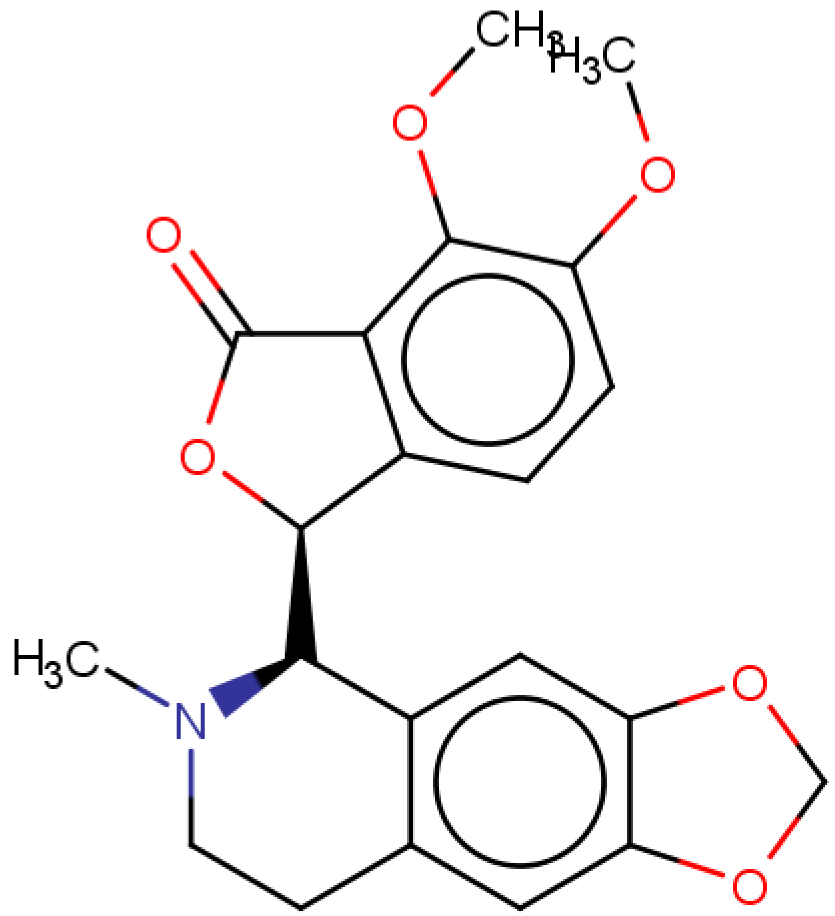

118-08-1
Name: Hydrastine
pIC50: 4.68
Rank: 429
Classes: No defined

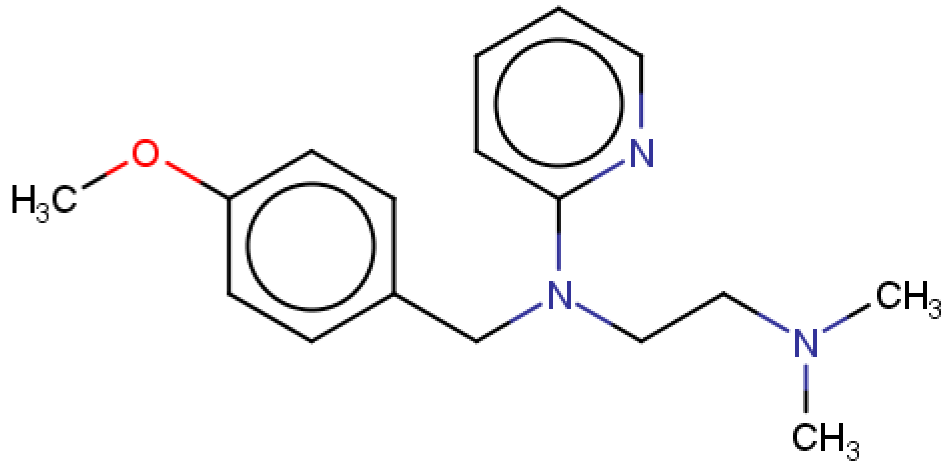

59-33-6
Name: Pyrilamine maleate
pIC50: 4.68
Rank: 430
Classes: No defined

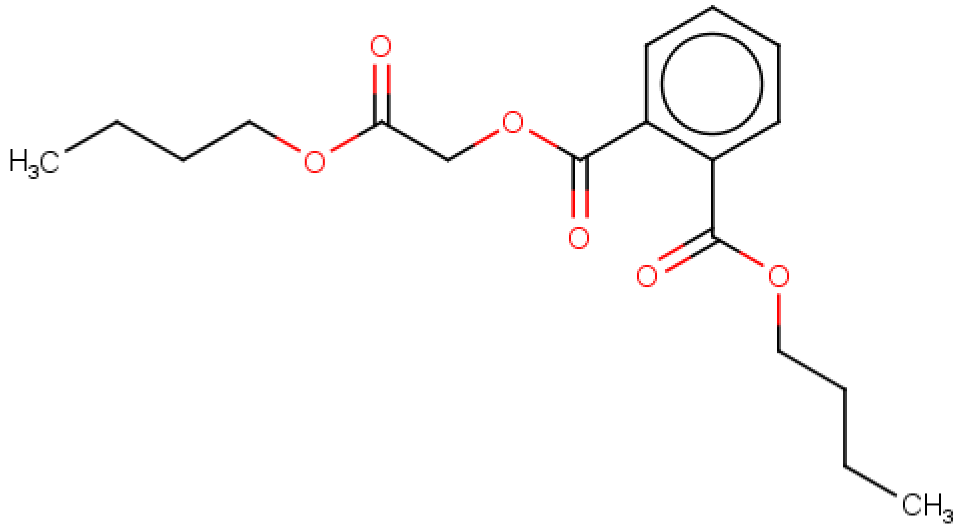

85-70-1
Name: Butylphthalyl butylglycolate
pIC50: 4.68
Rank: 431
Classes: film forming agent

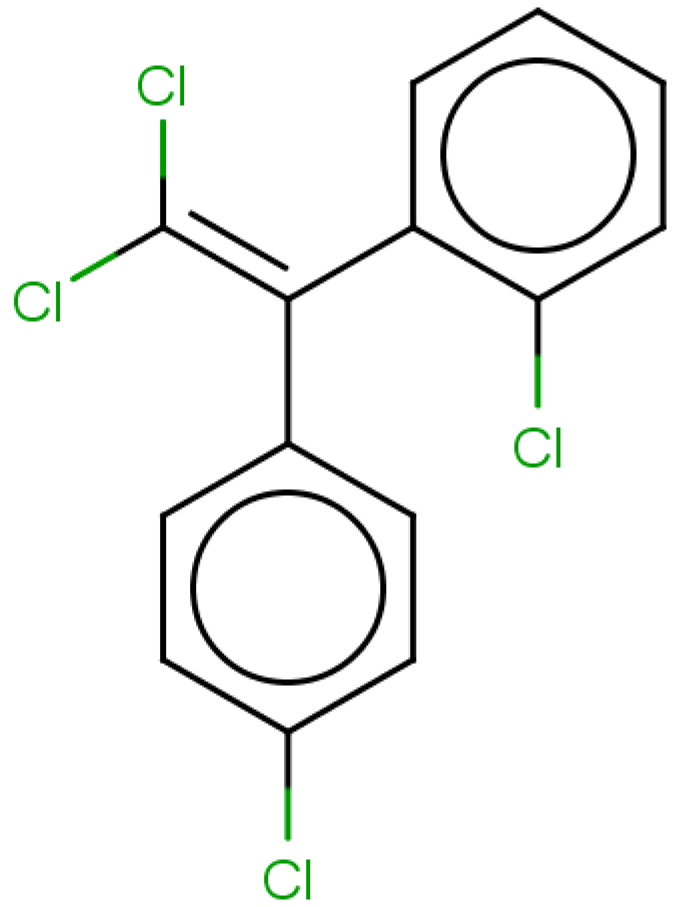

3424-82-6
Name: o p'-DDE
pIC50: 4.68
Rank: 432
Classes: No defined

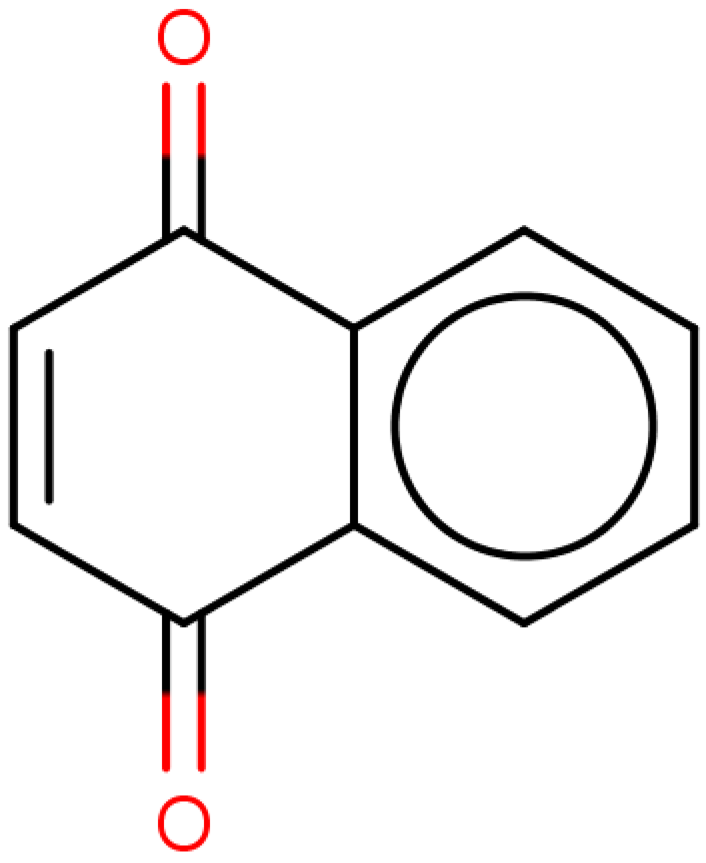

130-15-4
Name: 1 4-Naphthoquinone
pIC50: 4.68
Rank: 433
Classes: TSCA

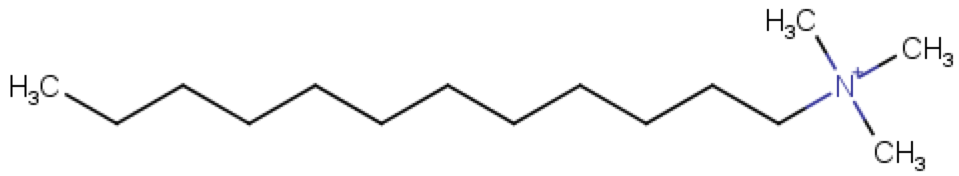

1119-94-4
Name: Dodecyltrimethylammonium bromide
pIC50: 4.68
Rank: 434
Classes: hair conditioner--TSCA

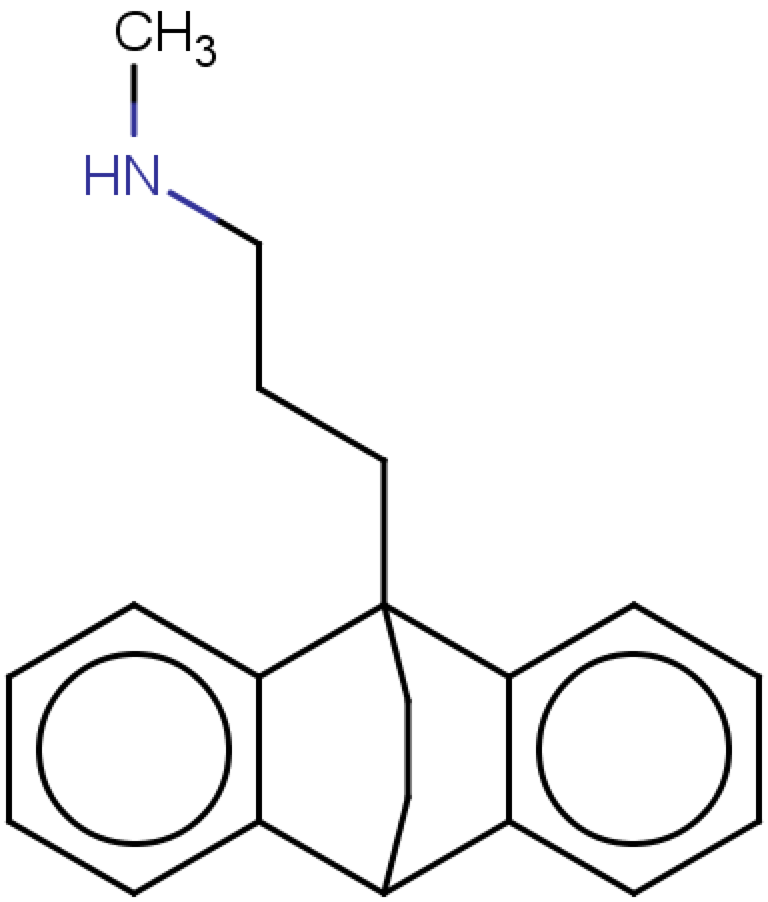

10347-81-6
Name: Maprotiline hydrochloride
pIC50: 4.68
Rank: 435
Classes: No defined

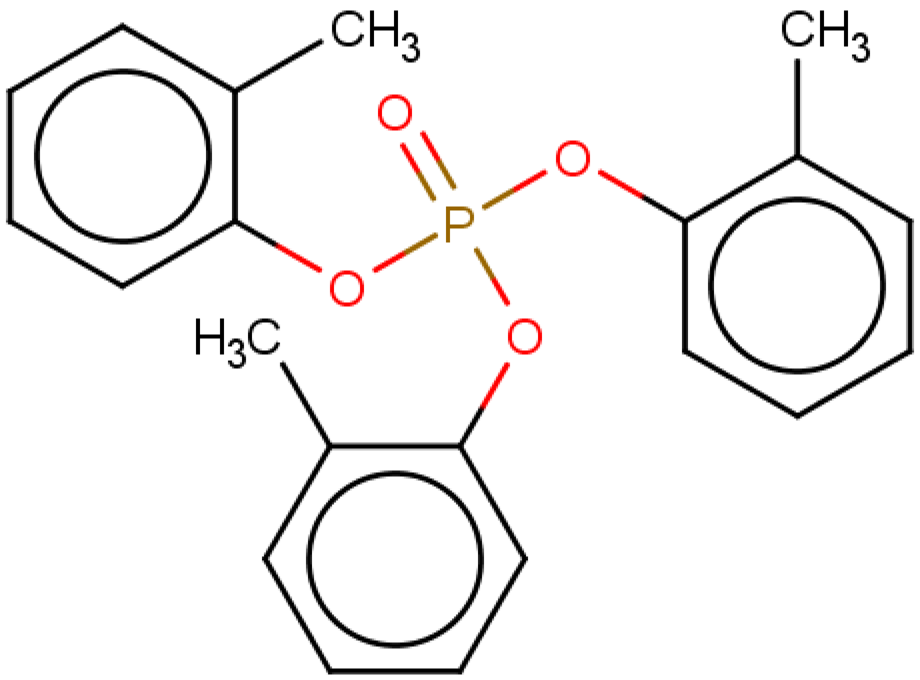

78-30-8
Name: Tri-o-cresyl phosphate
pIC50: 4.68
Rank: 436
Classes: TSCA

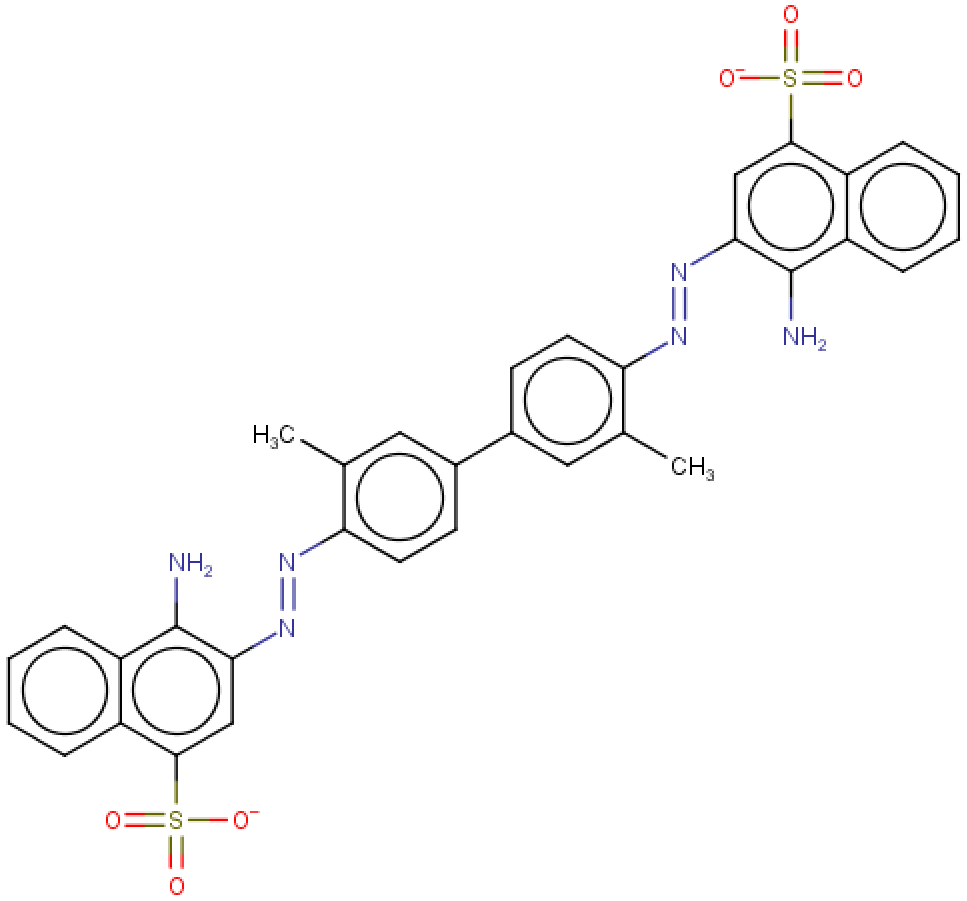

992-59-6
Name: C.I. Direct Red 2
pIC50: 4.67
Rank: 437
Classes: No defined

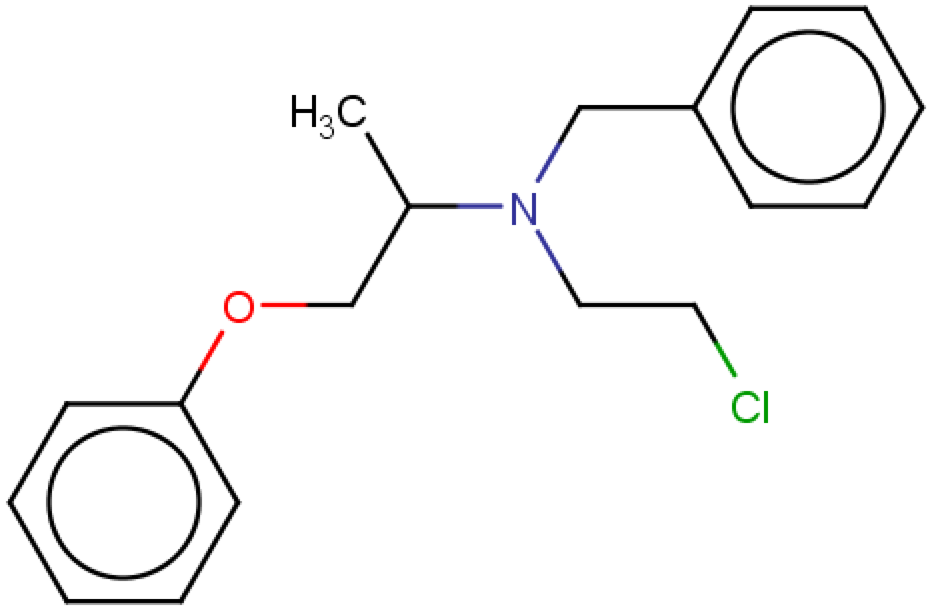

63-92-3
Name: Phenoxybenzamine hydrochloride
pIC50: 4.67
Rank: 438
Classes: No defined

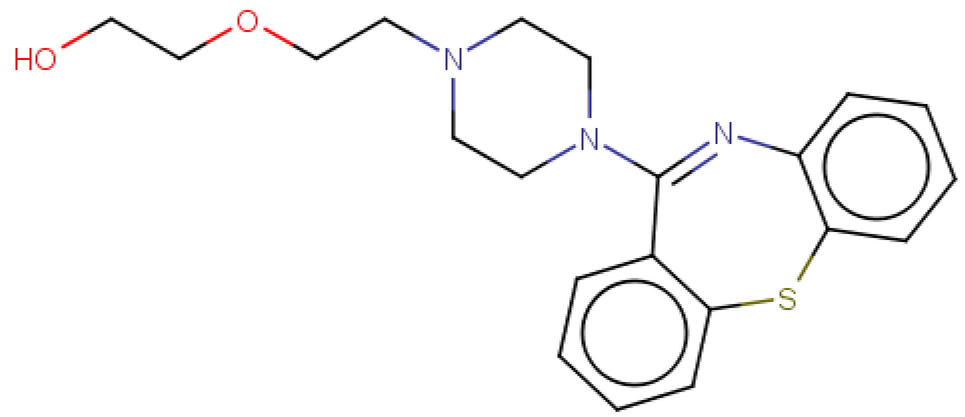

111974-72-2
Name: Quetiapine fumarate
pIC50: 4.67
Rank: 439
Classes: No defined

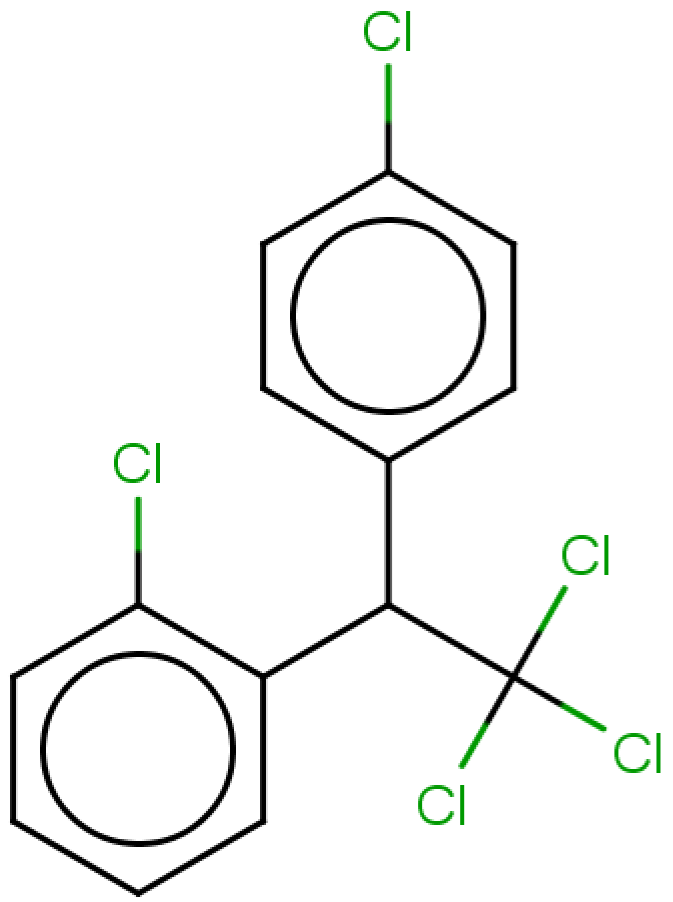

789-02-6
Name: o p'-DDT
pIC50: 4.67
Rank: 440
Classes: No defined

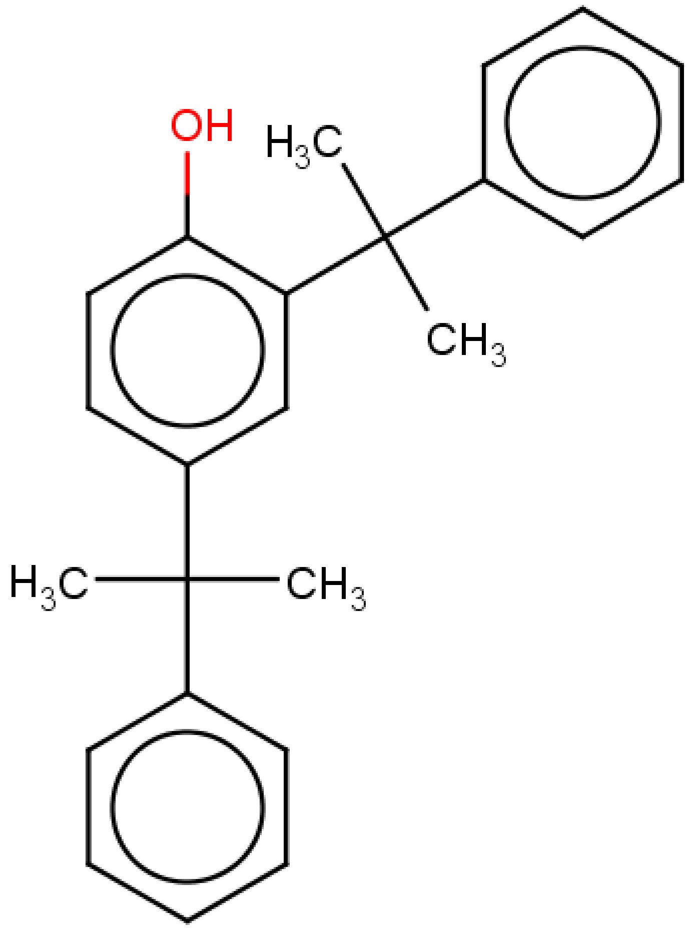

2772-45-4
Name: 2 4-Bis(1-methyl-1-phenylethyl)phenol
pIC50: 4.66
Rank: 441
Classes: TSCA

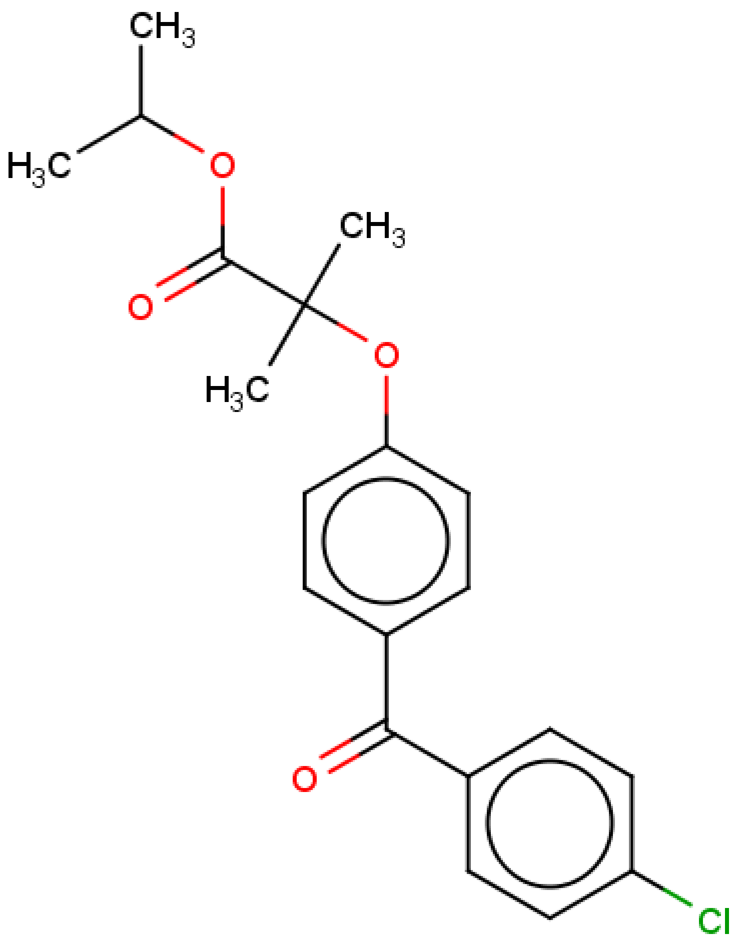

49562-28-9
Name: Fenofibrate
pIC50: 4.66
Rank: 442
Classes: Drug

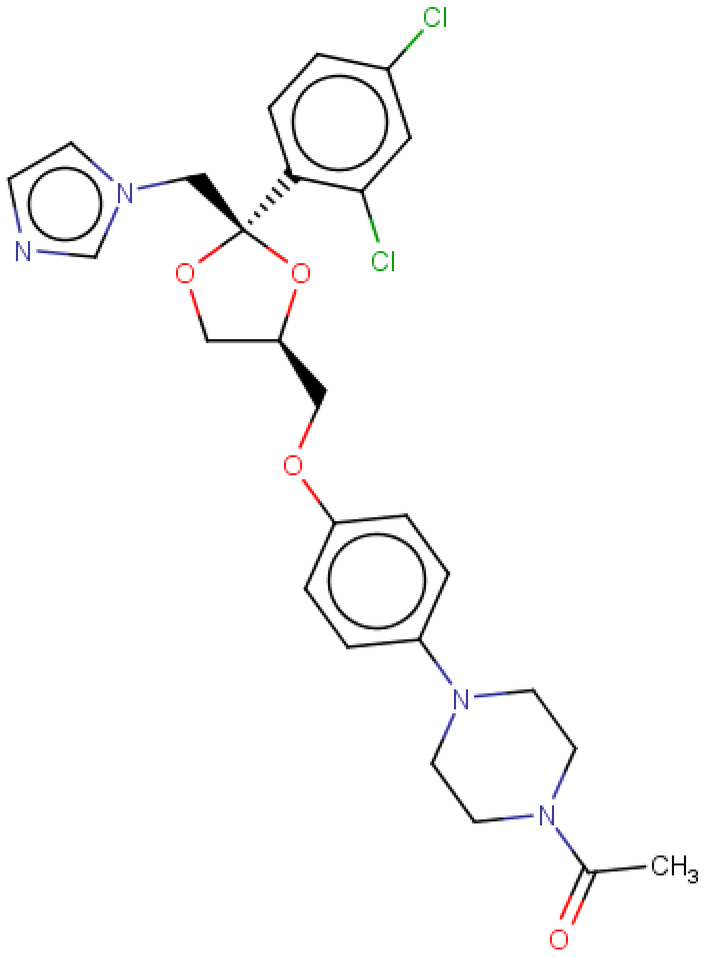

65277-42-1
Name: Ketoconazole
pIC50: 4.66
Rank: 443
Classes: No defined

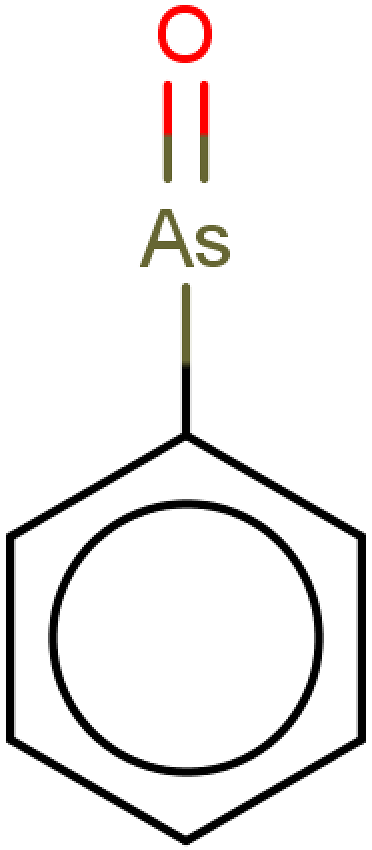

637-03-6
Name: Phenylarsine oxide
pIC50: 4.66
Rank: 444
Classes: No defined

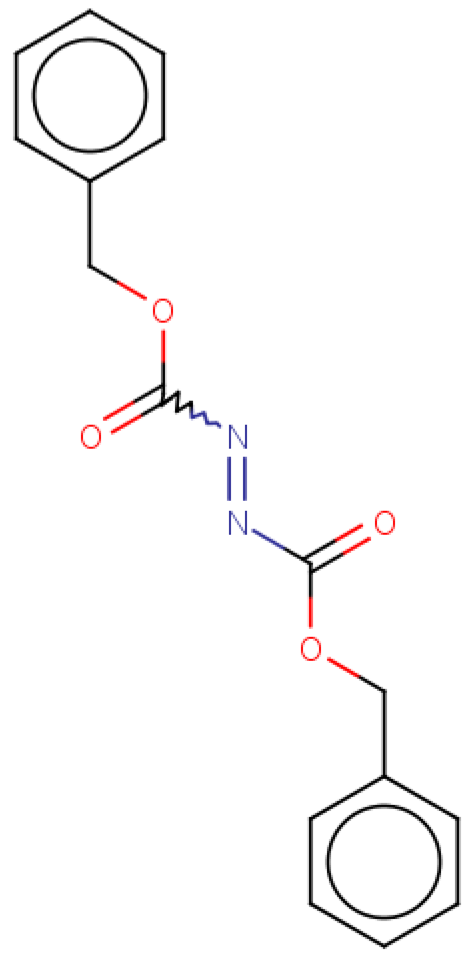

2449-05-0
Name: Dibenzyl diazene-1 2-dicarboxylate
pIC50: 4.66
Rank: 445
Classes: No defined

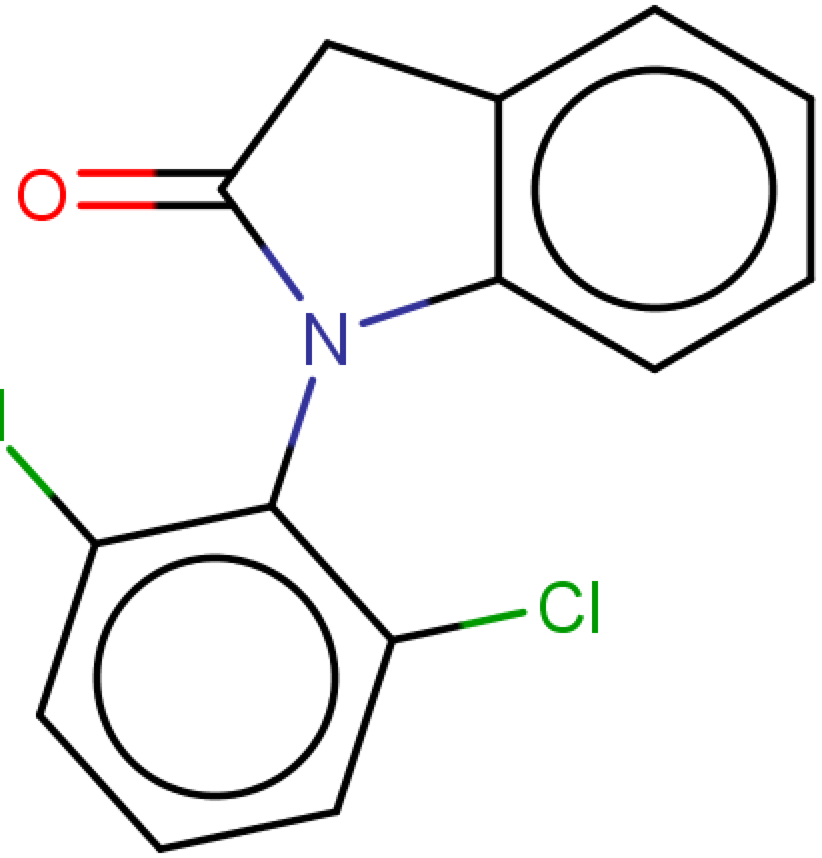

15362-40-0
Name: 1-(2 6-Dichlorophenyl)-2-indolinone
pIC50: 4.66
Rank: 446
Classes: No defined

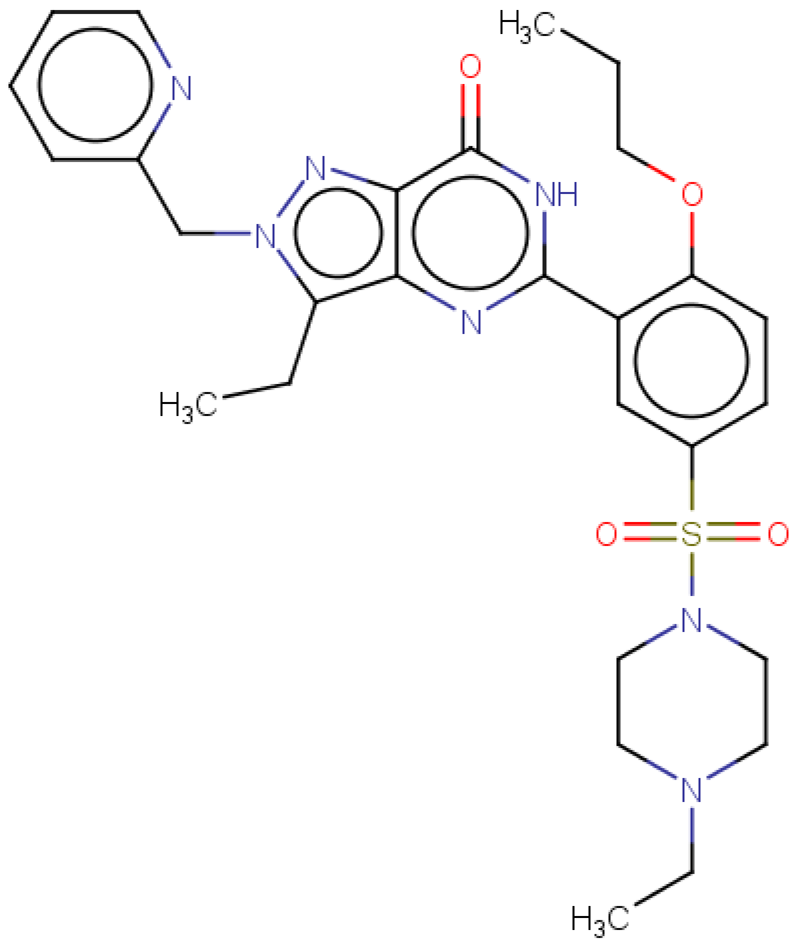

215297-27-1
Name: UK-343664
pIC50: 4.66
Rank: 447
Classes: No defined

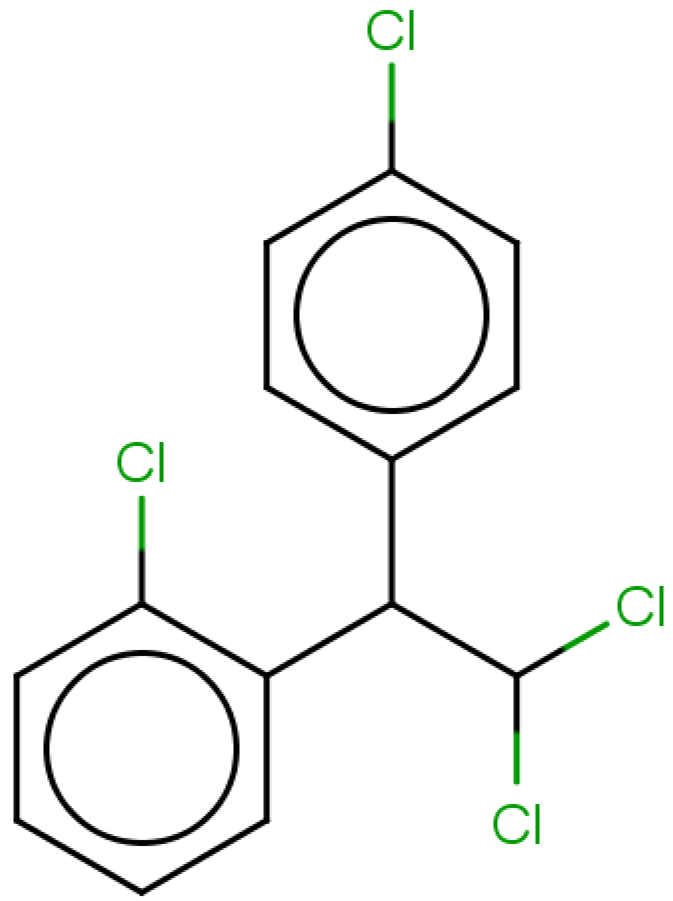

53-19-0
Name: o p'-DDD
pIC50: 4.66
Rank: 448
Classes: Drug

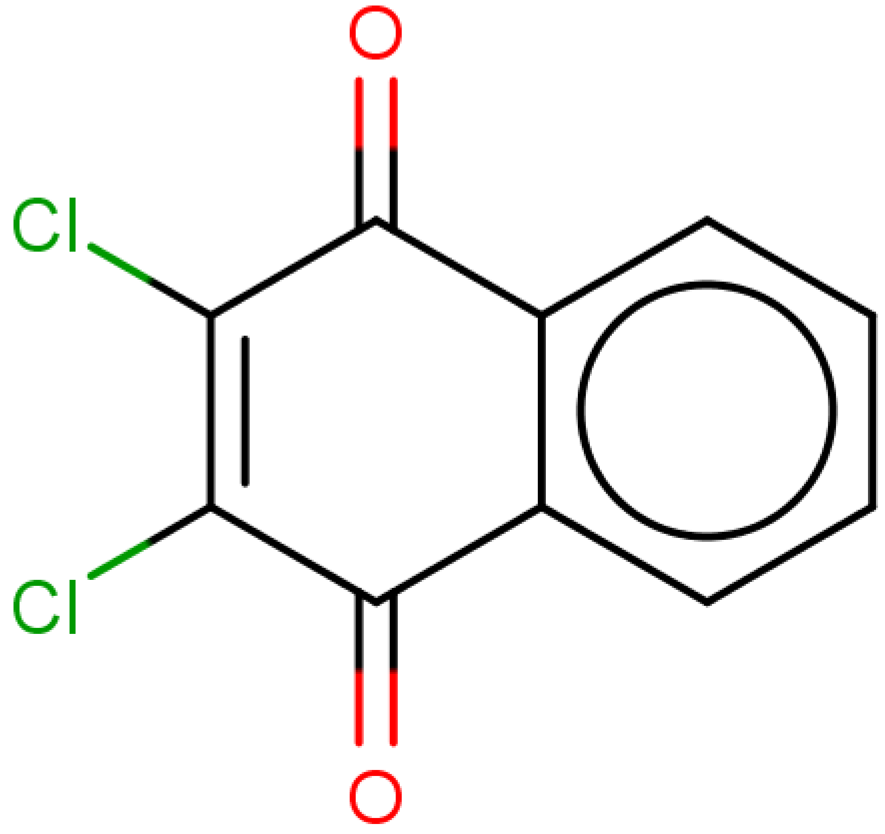

117-80-6
Name: Dichlone
pIC50: 4.66
Rank: 449
Classes: antimicrobial--TSCA

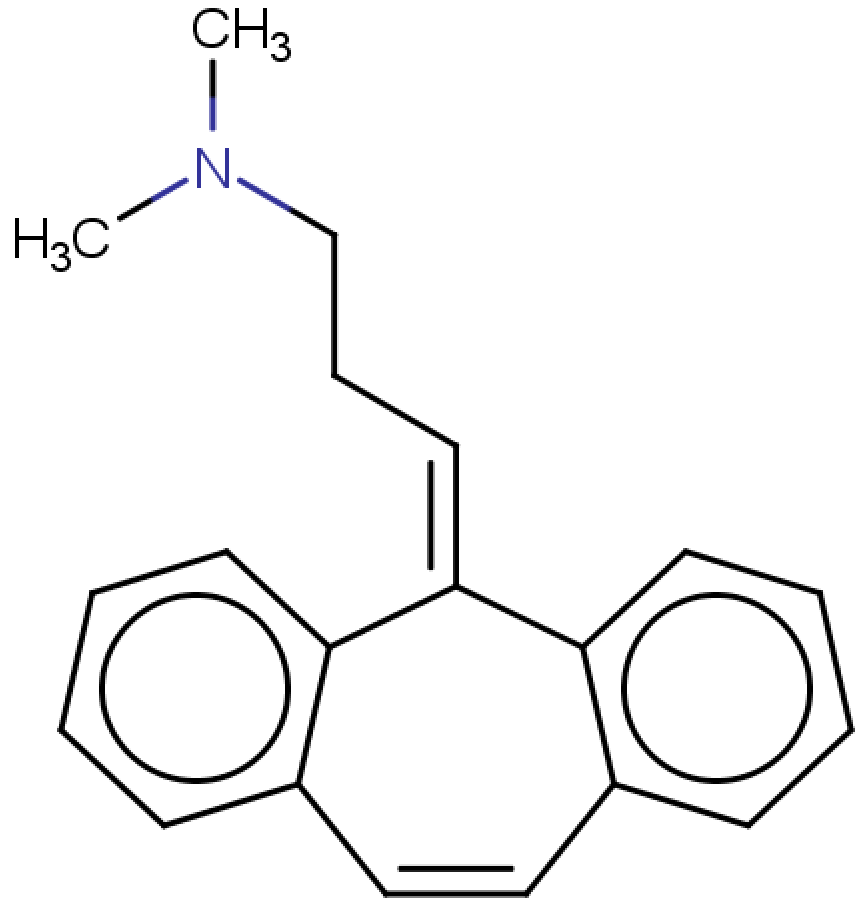

6202-23-9
Name: Cyclobenzaprine hydrochloride
pIC50: 4.65
Rank: 450
Classes: No defined

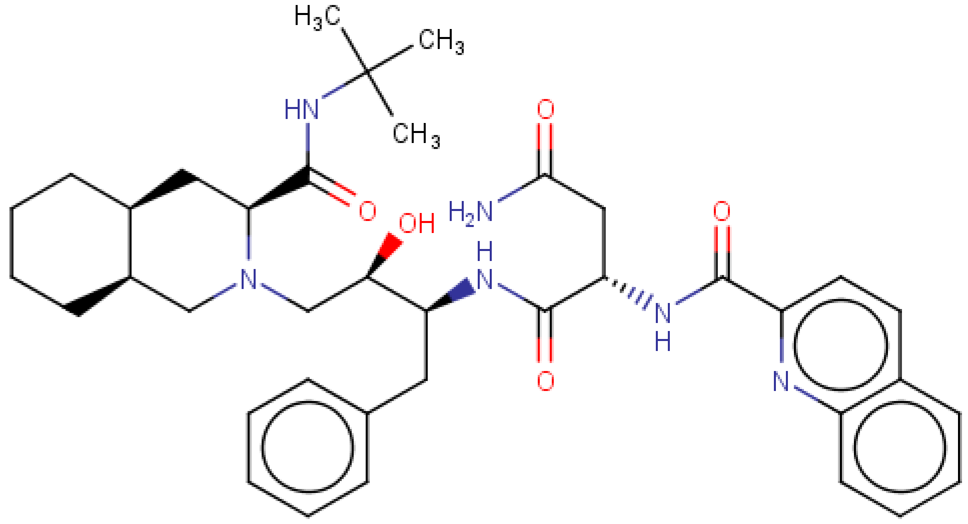

149845-06-7
Name: Saquinavir mesylate
pIC50: 4.65
Rank: 451
Classes: No defined

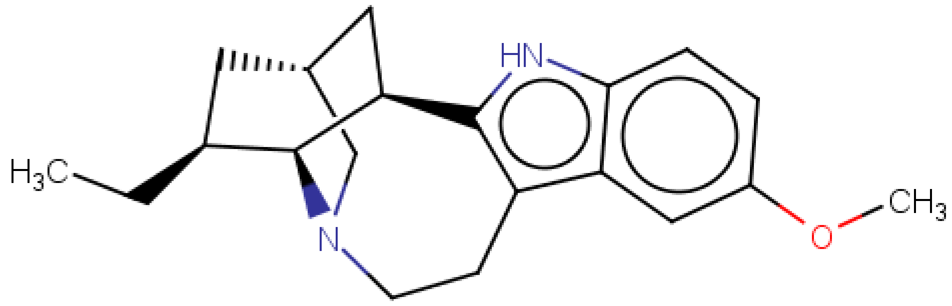

36415-61-9
Name: Ibogaine hydrochloride
pIC50: 4.65
Rank: 452
Classes: No defined

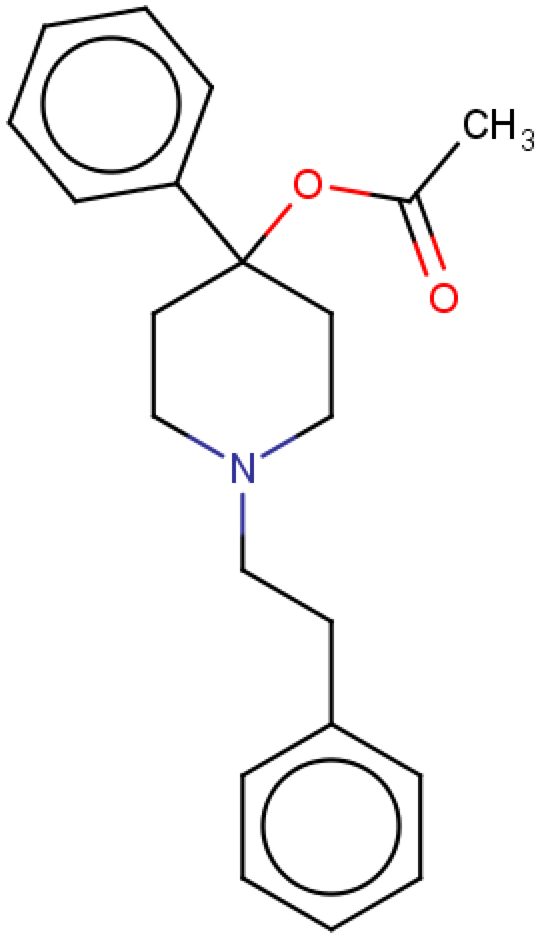

64-52-8
Name: 1-(2-Phenylethyl)-4-phenyl-4-acetoxypi[illegible]
pIC50: 4.65
Rank: 453
Classes: Drug

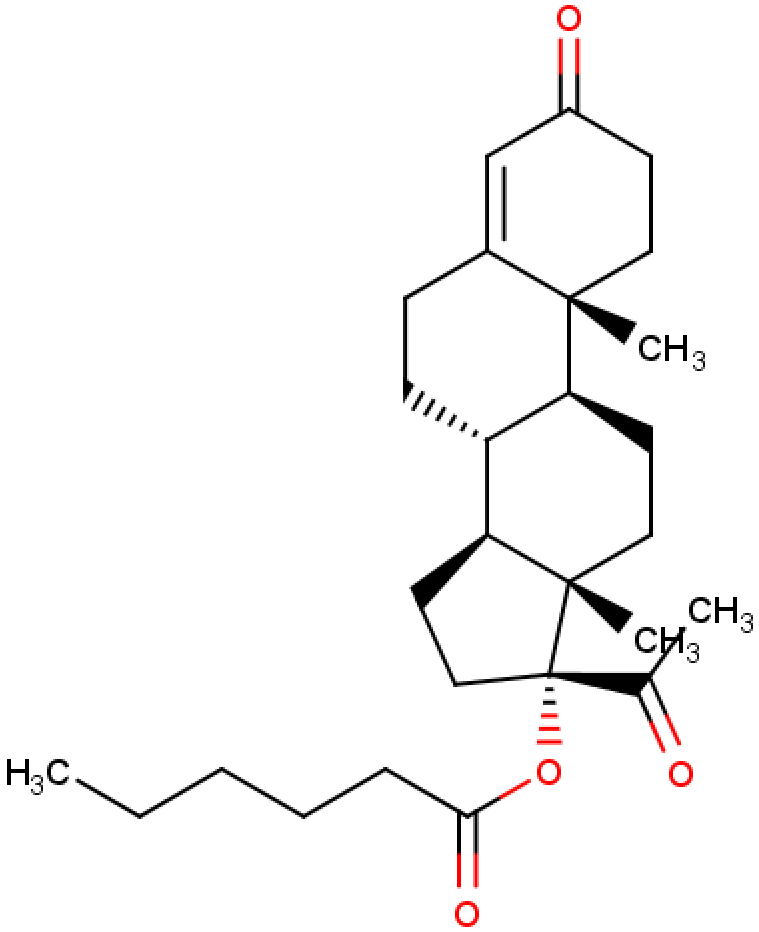

630-56-8
Name: Hydroxyprogesterone caproate
pIC50: 4.65
Rank: 454
Classes: Drug

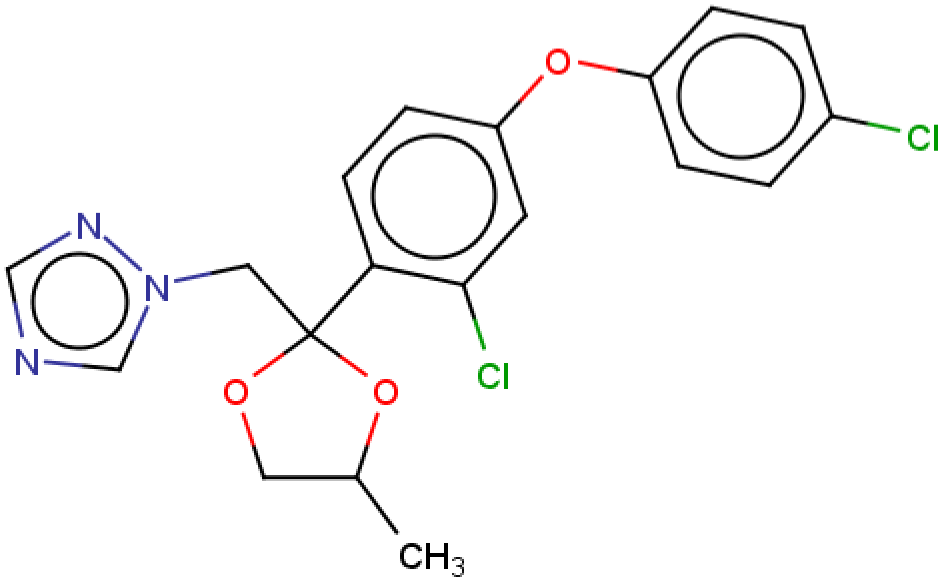

119446-68-3
Name: Difenoconazole
pIC50: 4.64
Rank: 455
Classes: Pesticide

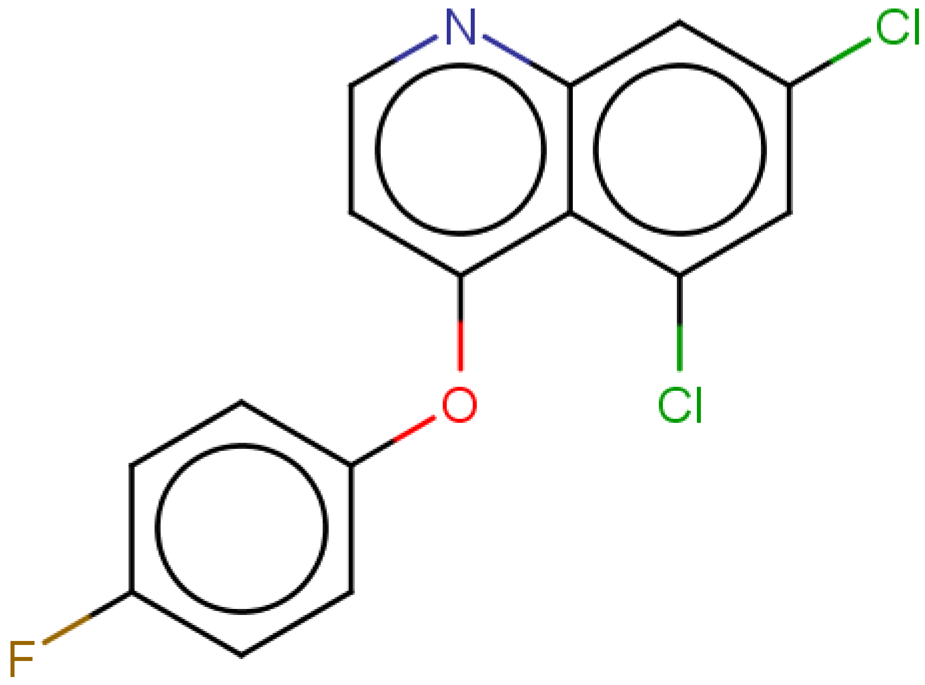

124495-18-7
Name: Quinoxyfen
pIC50: 4.64
Rank: 456
Classes: Pesticide

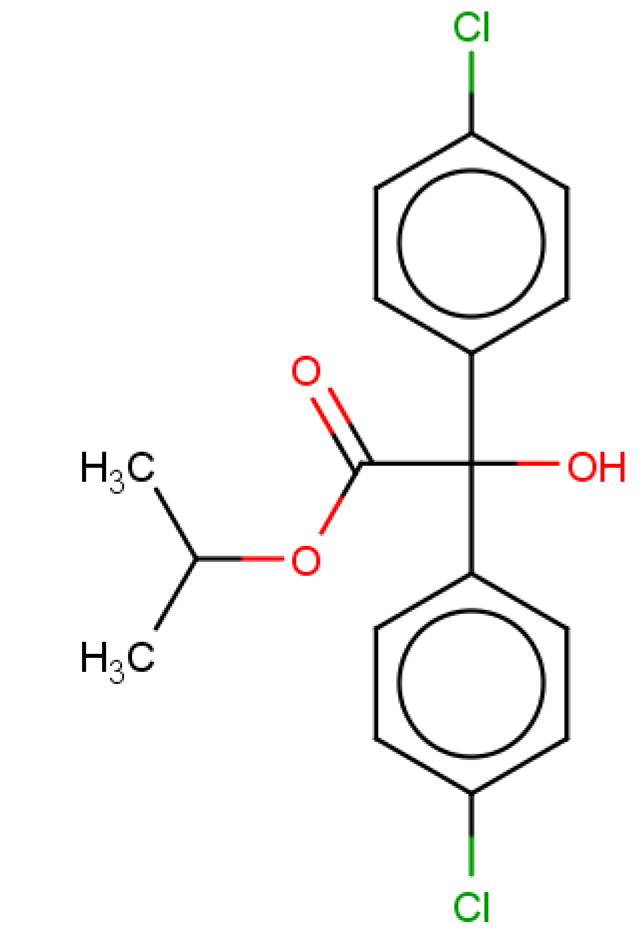

5836-10-2
Name: Chloropropylate
pIC50: 4.64
Rank: 457
Classes: No defined

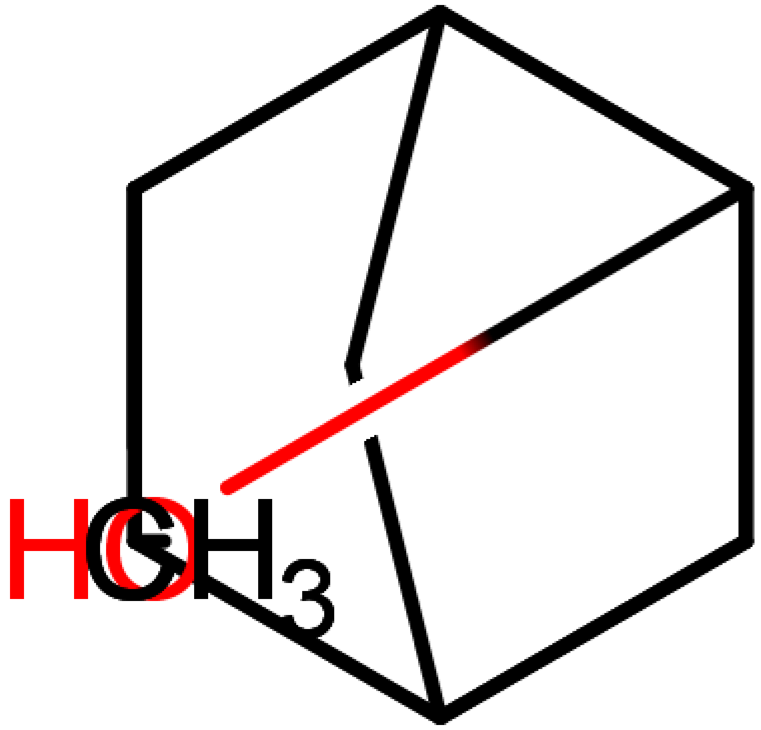

3407-42-9
Name: 3-(5 5 6-Trimethylbicyclo[2.2.1]hept-2-y[illegible]
pIC50: 4.64
Rank: 458
Classes: fragrance--TSCA

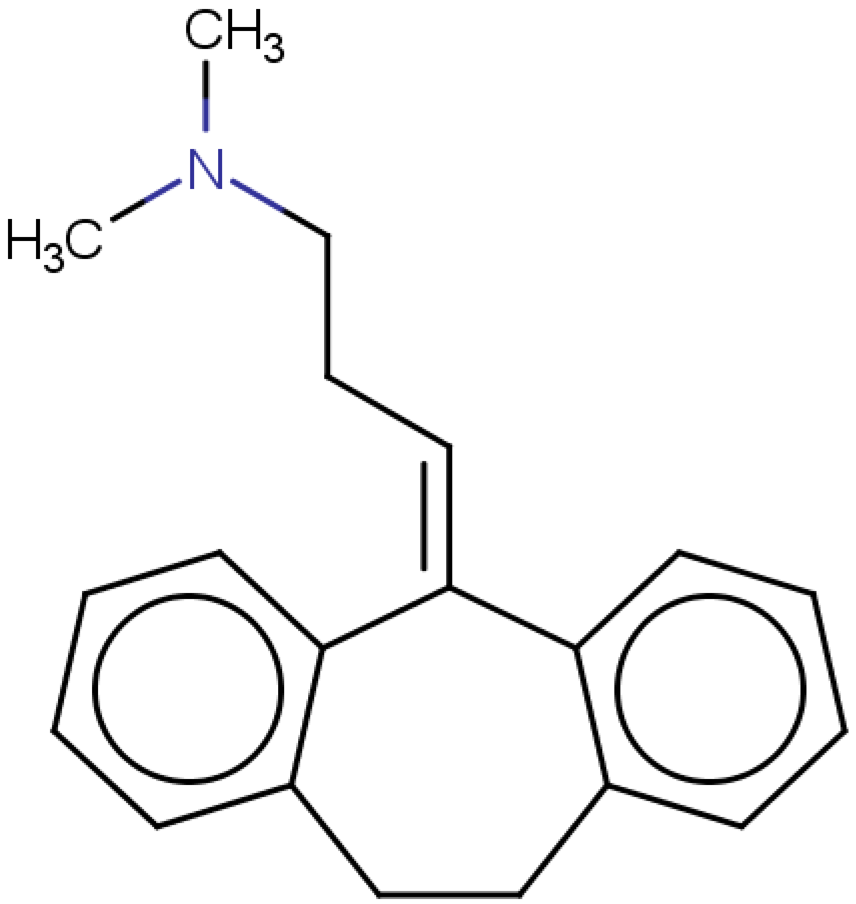

549-18-8
Name: Amitriptyline hydrochloride
pIC50: 4.64
Rank: 459
Classes: No defined

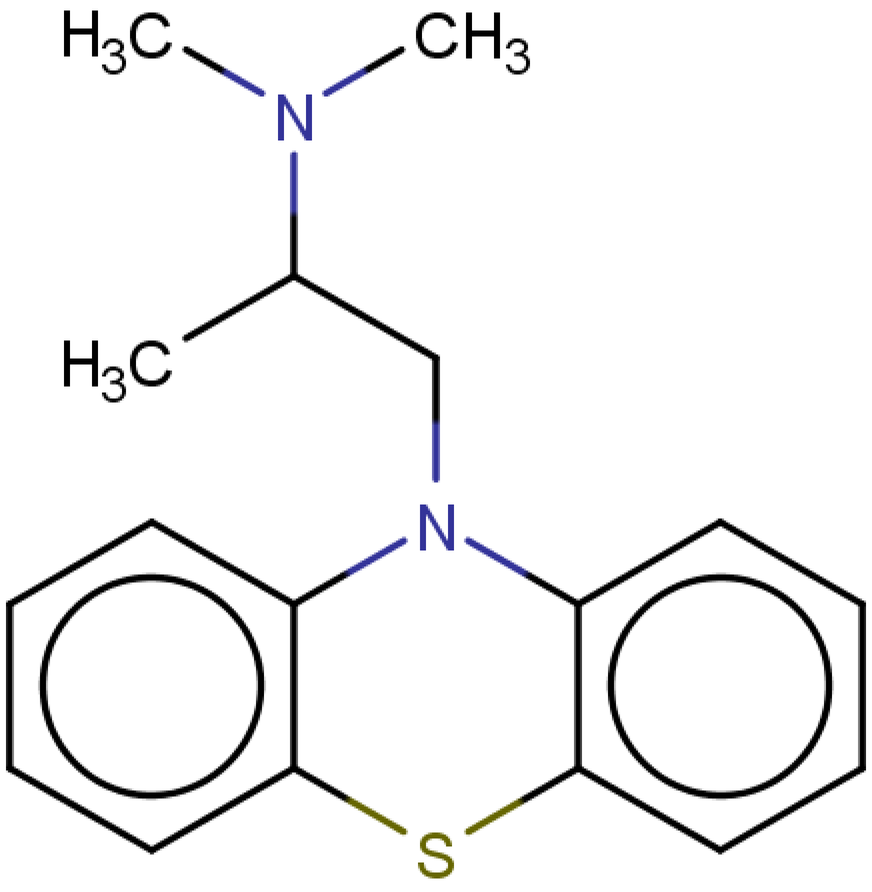

58-33-3
Name: Promethazine hydrochloride
pIC50: 4.64
Rank: 460
Classes: No defined

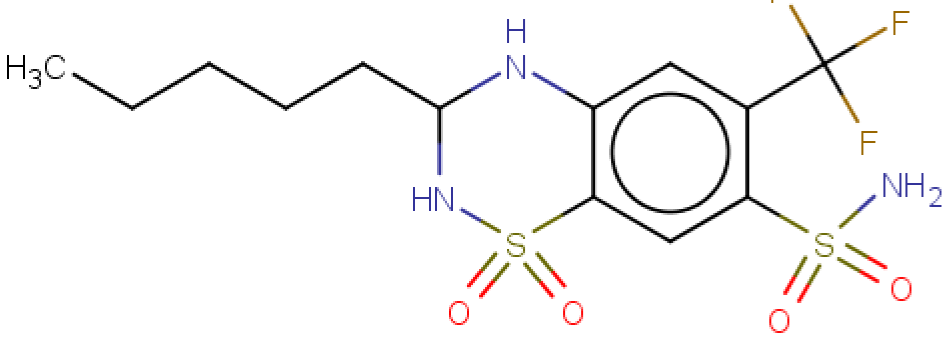

1766-91-2
Name: Penflutizide
pIC50: 4.64
Rank: 461
Classes: No defined

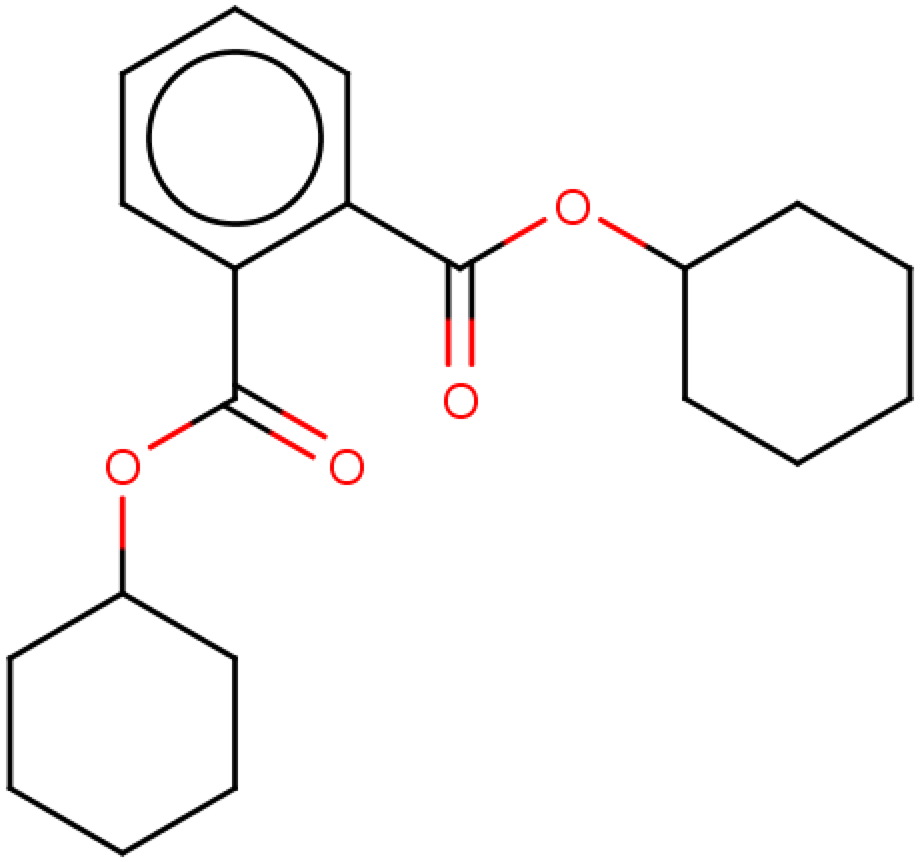

84-61-7
Name: Dicyclohexyl phthalate
pIC50: 4.64
Rank: 462
Classes: plastic

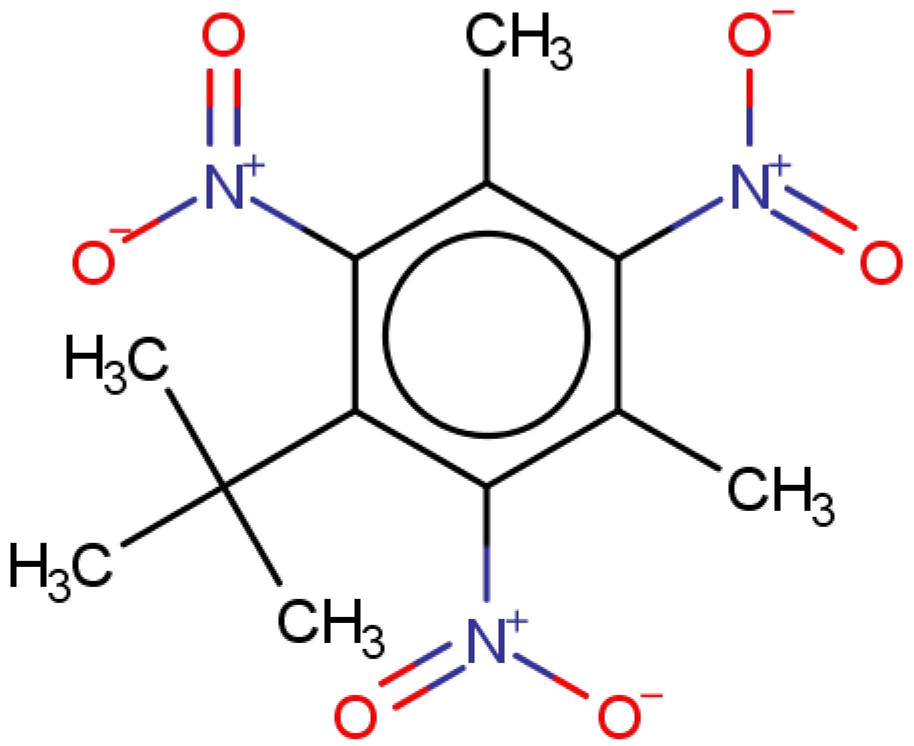

81-15-2
Name: 2 4 6-Trinitro-1 3-dimethyl-5-tert-butylbenzene
pIC50: 4.64
Rank: 463
Classes: perfumer--TSCA

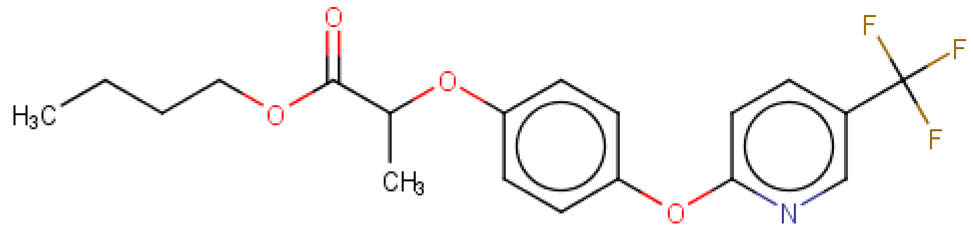

69806-50-4
Name: Fluazifop-butyl
pIC50: 4.64
Rank: 464
Classes: Pesticide

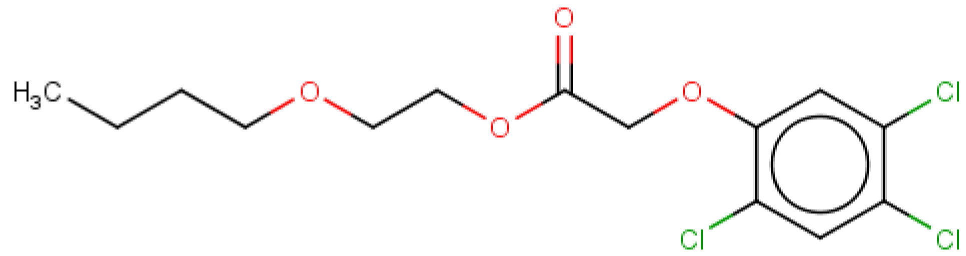

2545-59-7
Name: 2 4 5-T-butotyl
pIC50: 4.64
Rank: 465
Classes: No defined

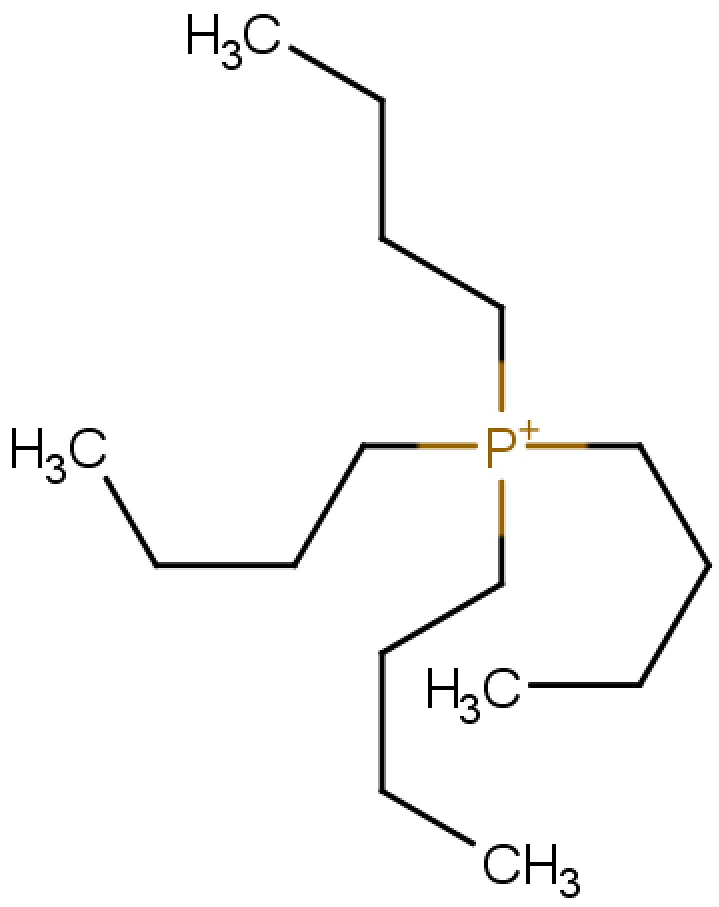

2304-30-5
Name: Tetrabutylphosphonium chloride
pIC50: 4.63
Rank: 466
Classes: catalyst

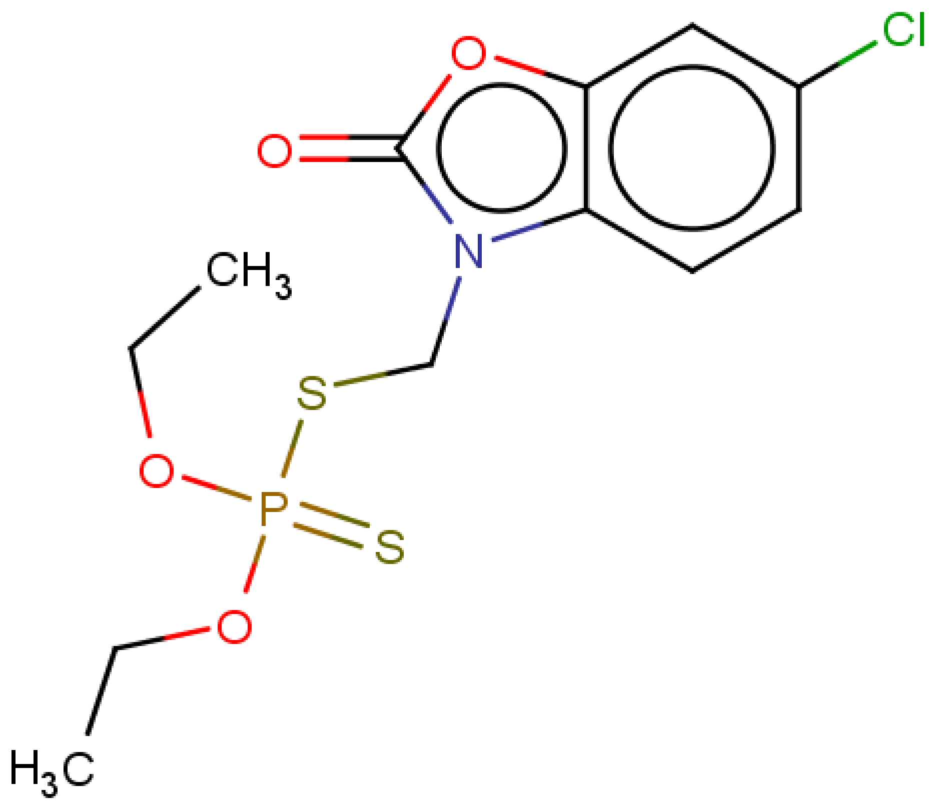

2310-17-0
Name: Phosalone
pIC50: 4.63
Rank: 467
Classes: Pesticide

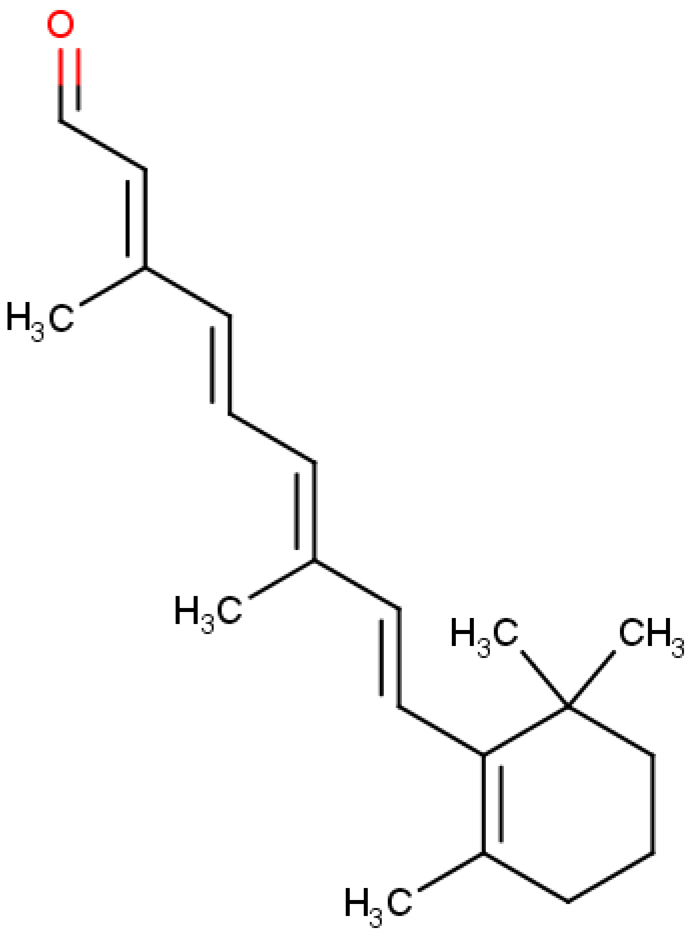

116-31-4
Name: Retinal
pIC50: 4.63
Rank: 468
Classes: skin conditioner

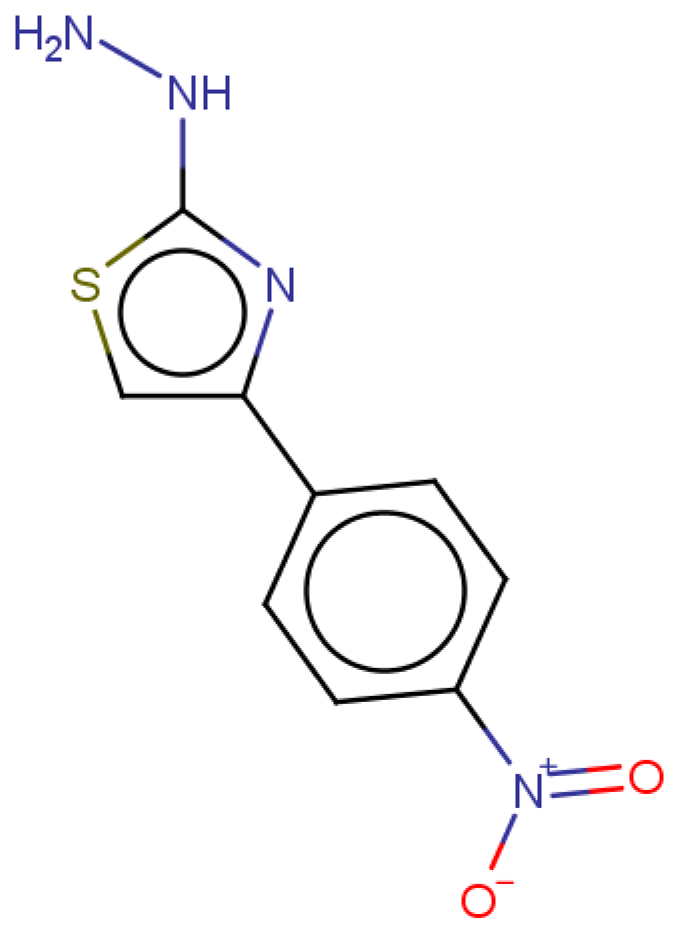

26049-70-7
Name: 2-Hydrazino-4-(4-nitrophenyl)thiazole
pIC50: 4.63
Rank: 469
Classes: No defined

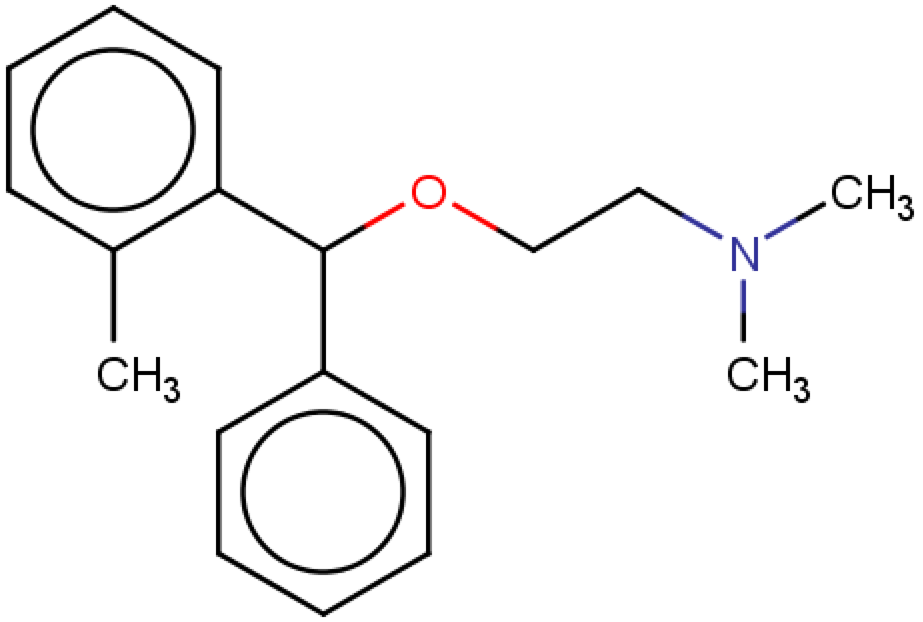

341-69-5
Name: Orphenadrine hydrochloride
pIC50: 4.63
Rank: 470
Classes: No defined

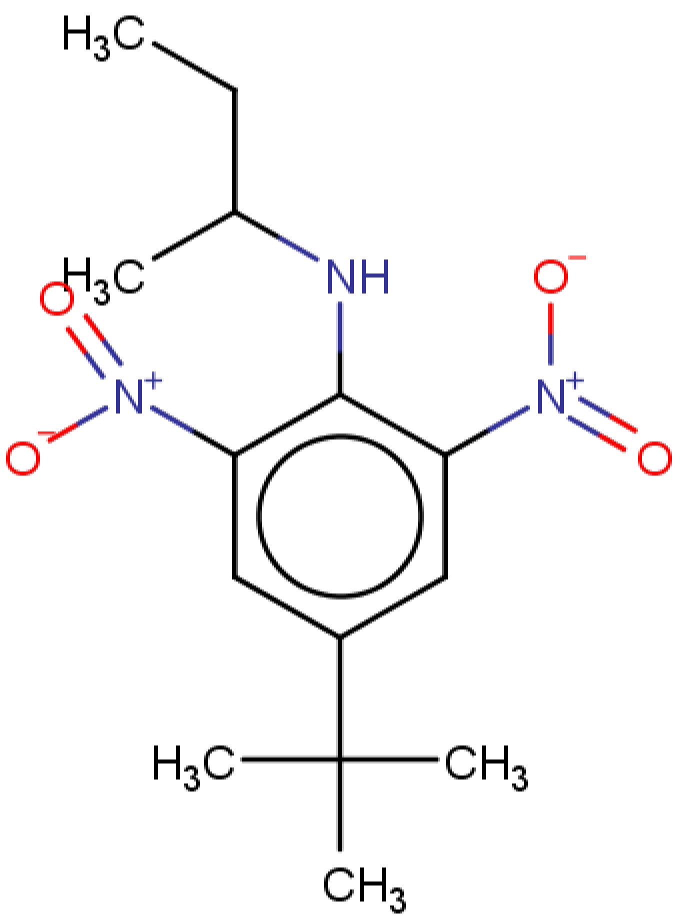

33629-47-9
Name: Butralin
pIC50: 4.63
Rank: 471
Classes: Pesticide

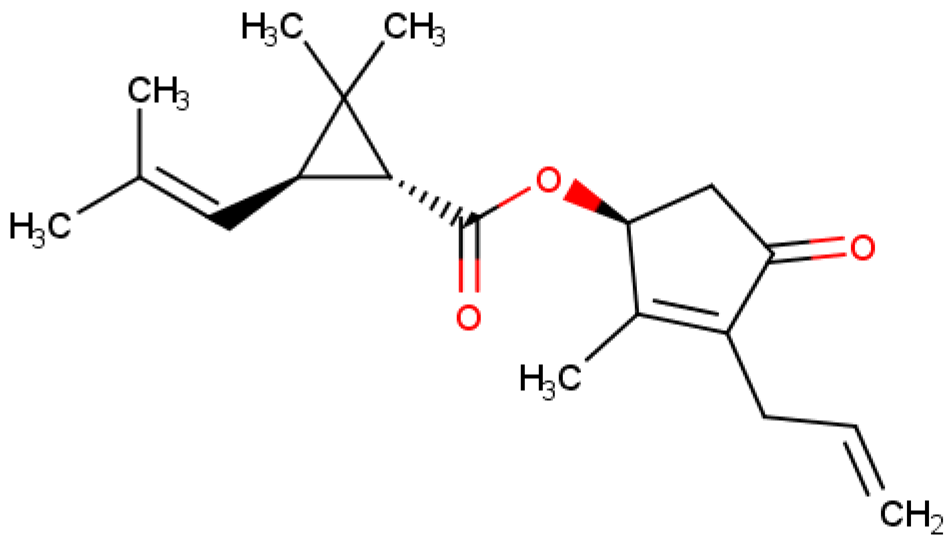

28434-00-6
Name: S-Bioallethrin
pIC50: 4.63
Rank: 472
Classes: masking agent

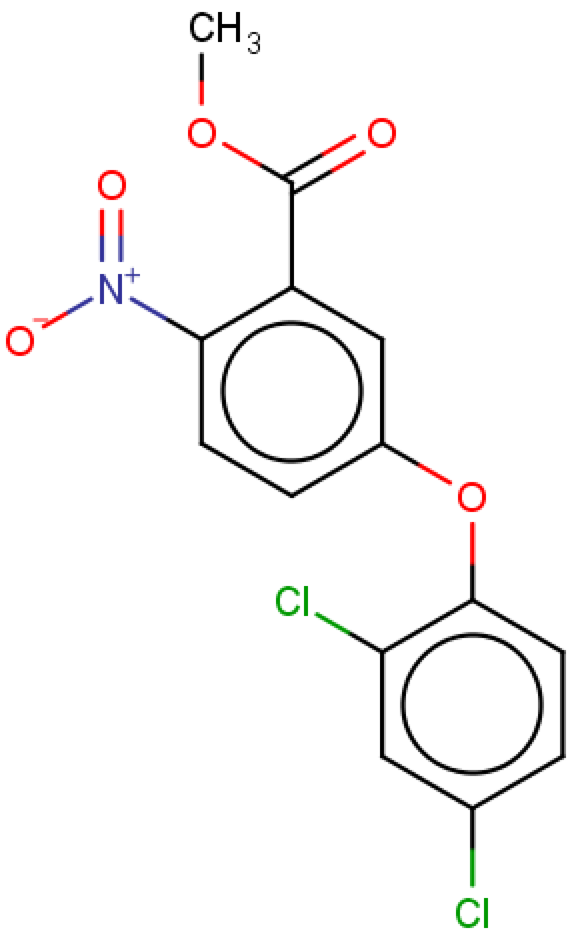

42576-02-3
Name: Bifenox
pIC50: 4.63
Rank: 473
Classes: No defined

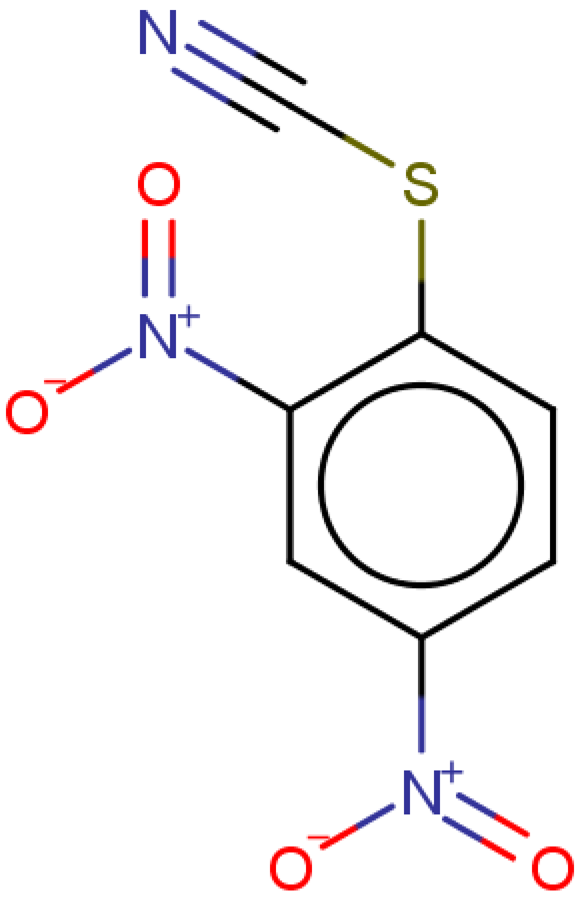

1594-56-5
Name: 2 4-Dinitrophenyl thiocyanate
pIC50: 4.63
Rank: 474
Classes: No defined

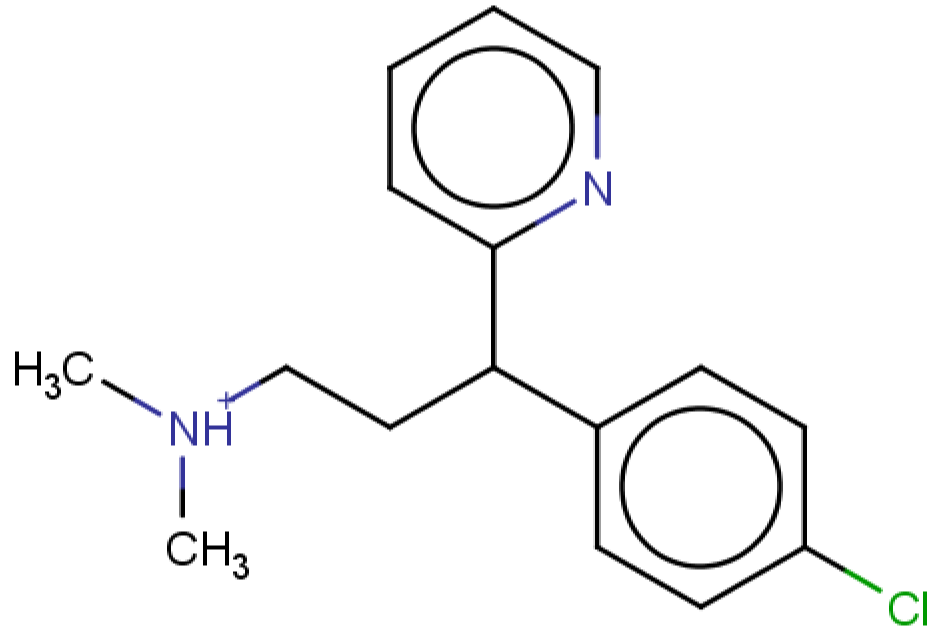

113-92-8
Name: Chlorpheniramine maleate
pIC50: 4.63
Rank: 475
Classes: No defined

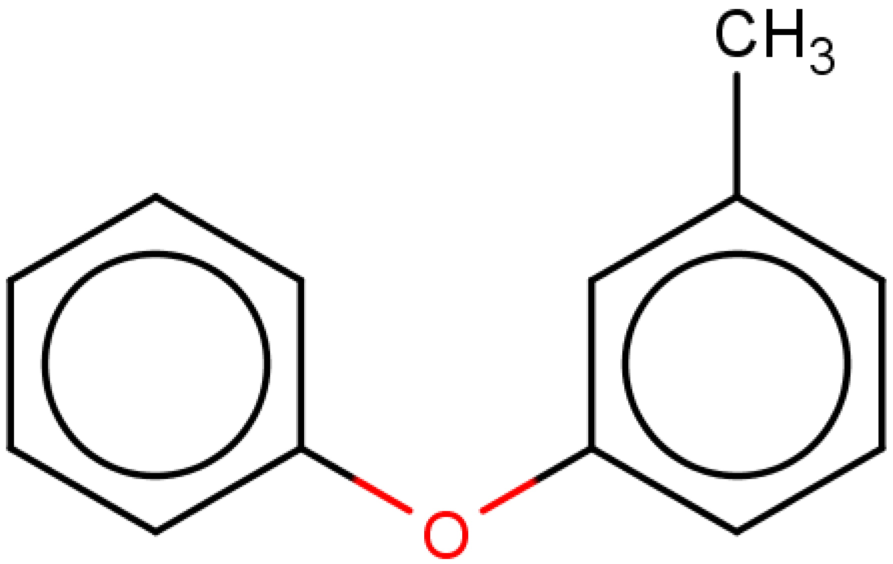

3586-14-9
Name: 1-Methyl-3-phenoxybenzene
pIC50: 4.62
Rank: 476
Classes: No defined

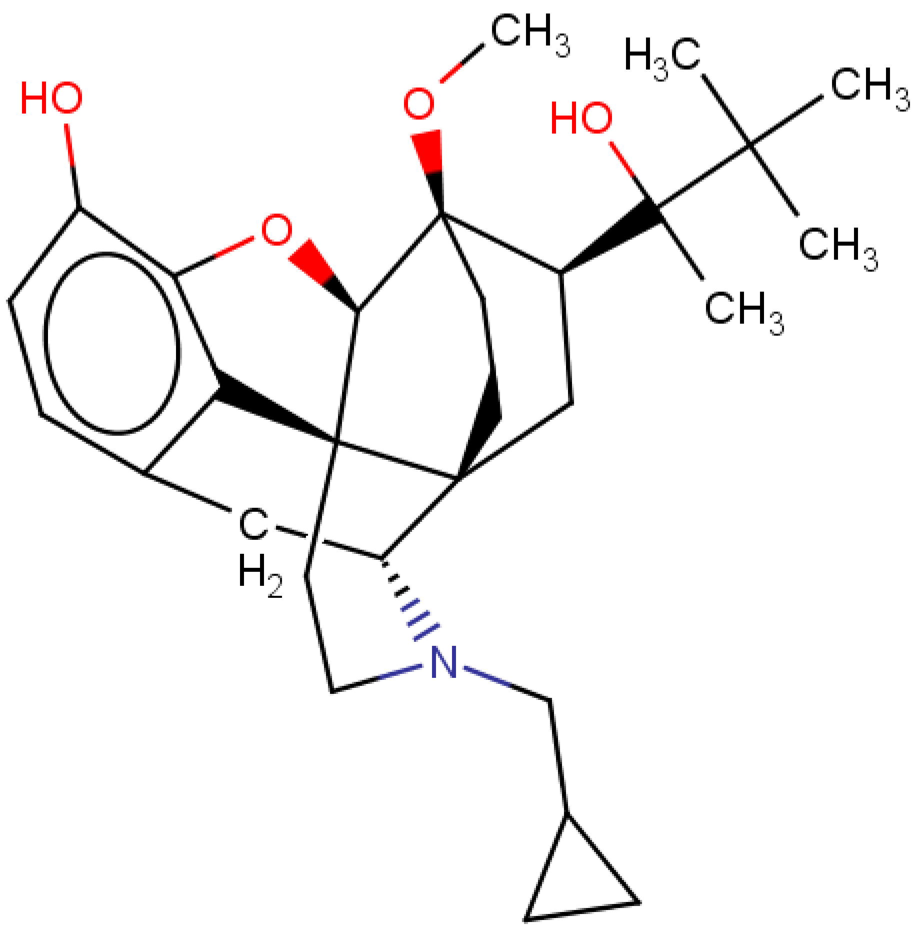

53152-21-9
Name: Buprenorphine hydrochloride
pIC50: 4.61
Rank: 477
Classes: No defined

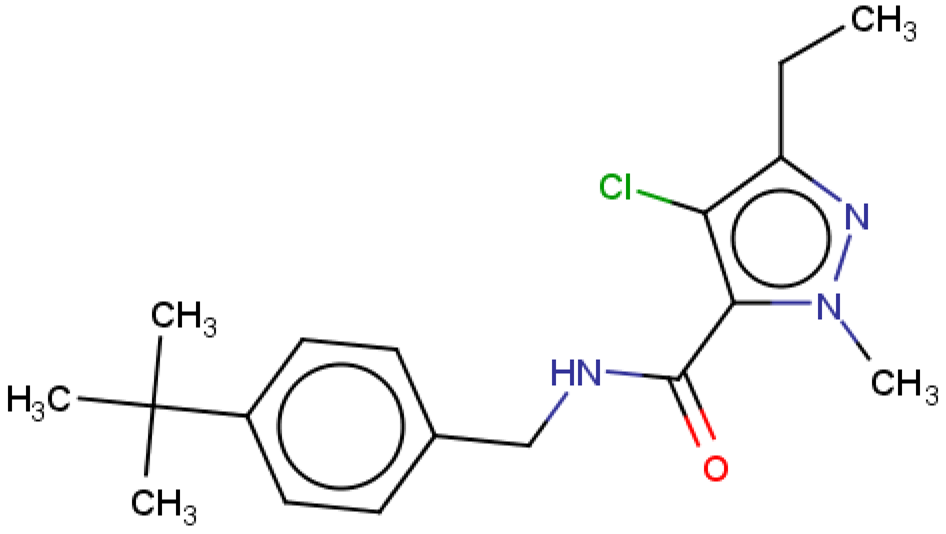

119168-77-3
Name: Tebufenpyrad
pIC50: 4.61
Rank: 478
Classes: Pesticide

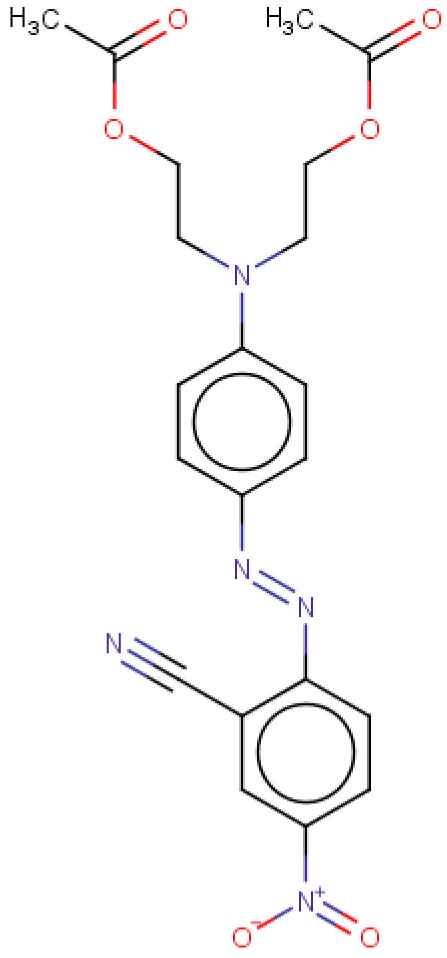

30124-94-8
Name: Disperse Red 82
pIC50: 4.61
Rank: 479
Classes: No defined

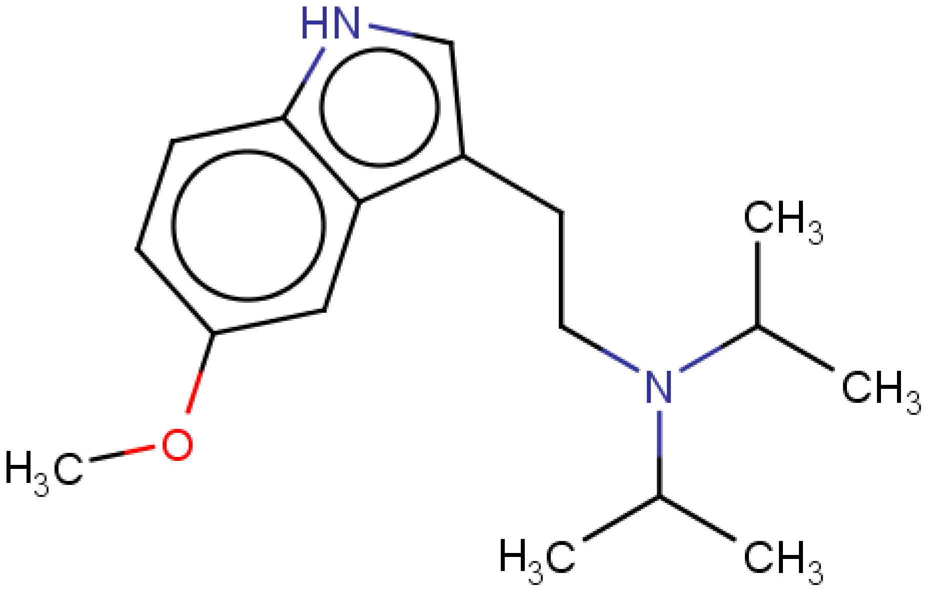

2426-63-3
Name: 5-Methoxy-N N-diisopropyltryptamine
pIC50: 4.6
Rank: 480
Classes: No defined

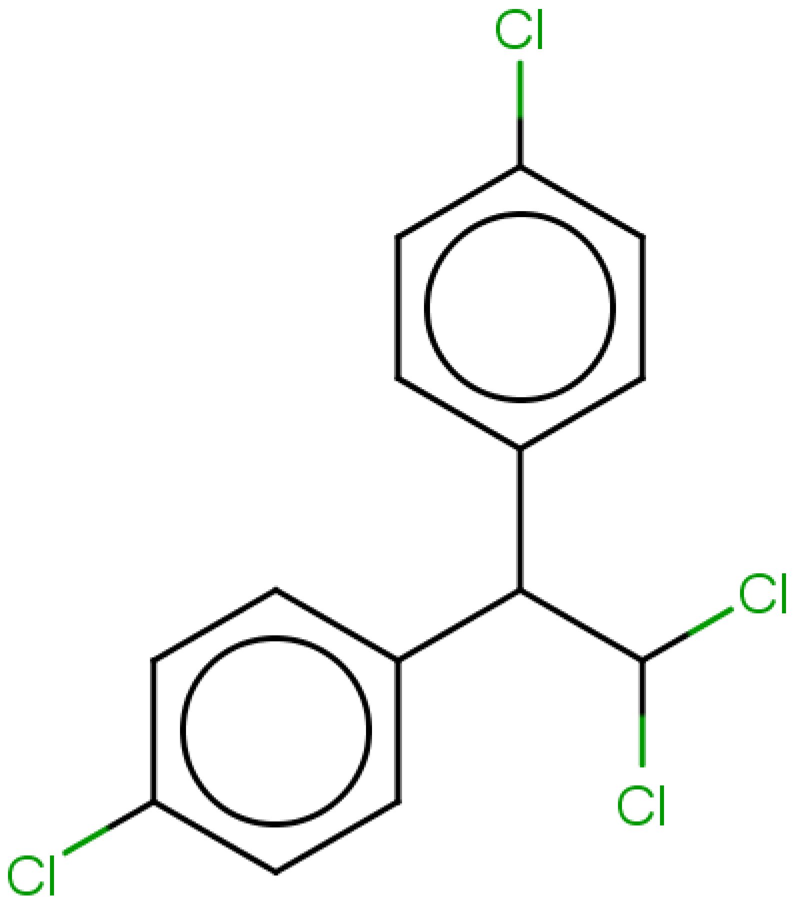

72-54-8
Name: p p'-DDD
pIC50: 4.59
Rank: 481
Classes: No defined

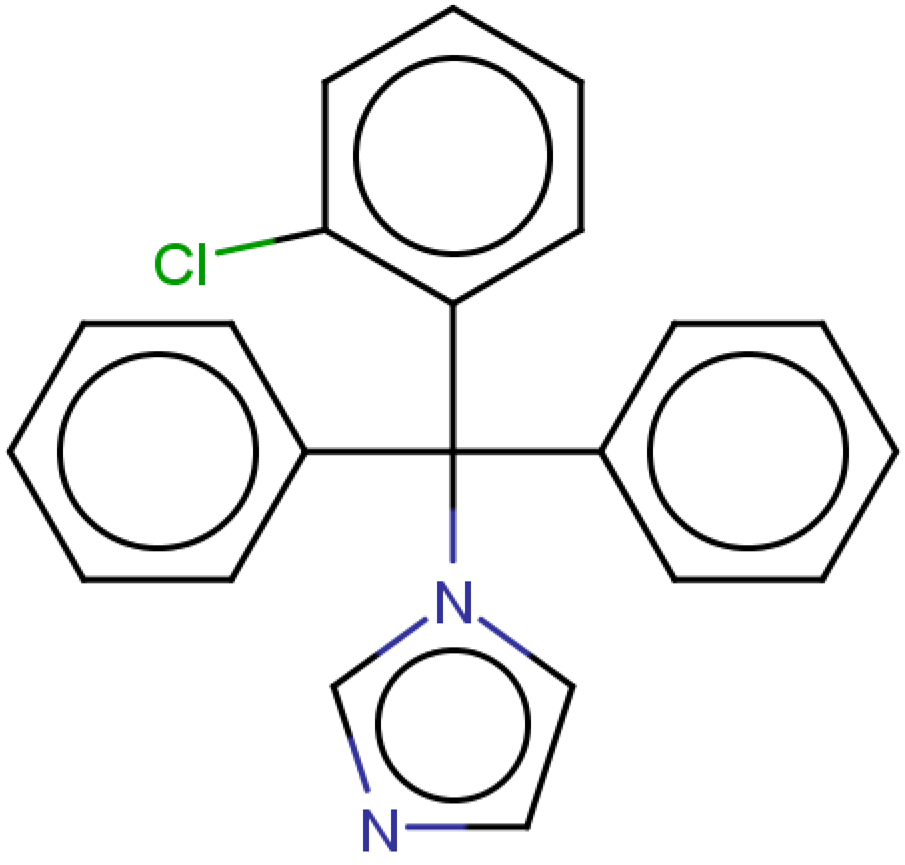

23593-75-1
Name: Clotrimazole
pIC50: 4.59
Rank: 482
Classes: antimicrobial--Drug

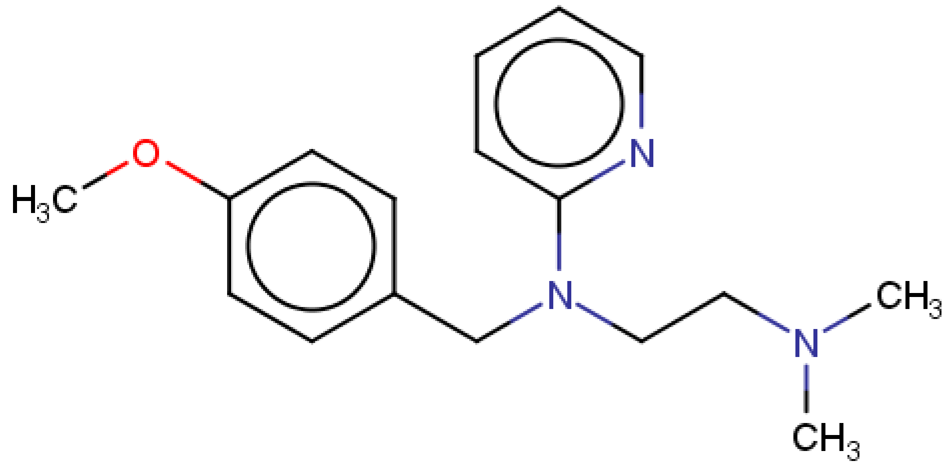

91-84-9
Name: Pyrilamine
pIC50: 4.56
Rank: 483
Classes: Drug

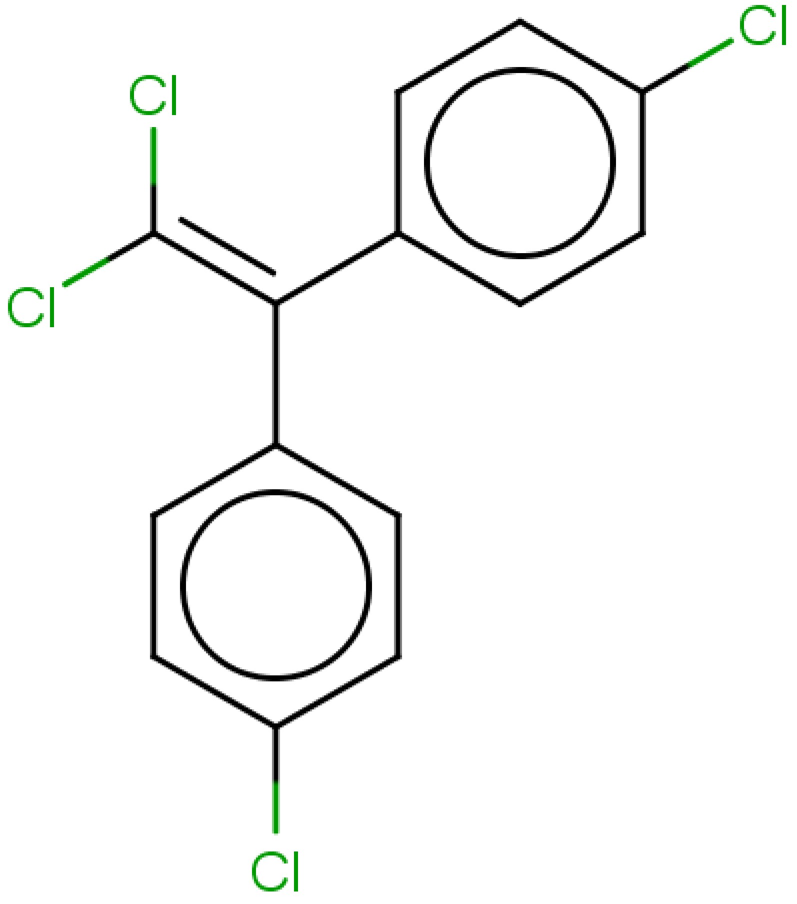

72-55-9
Name: p p'-DDE
pIC50: 4.56
Rank: 484
Classes: No defined

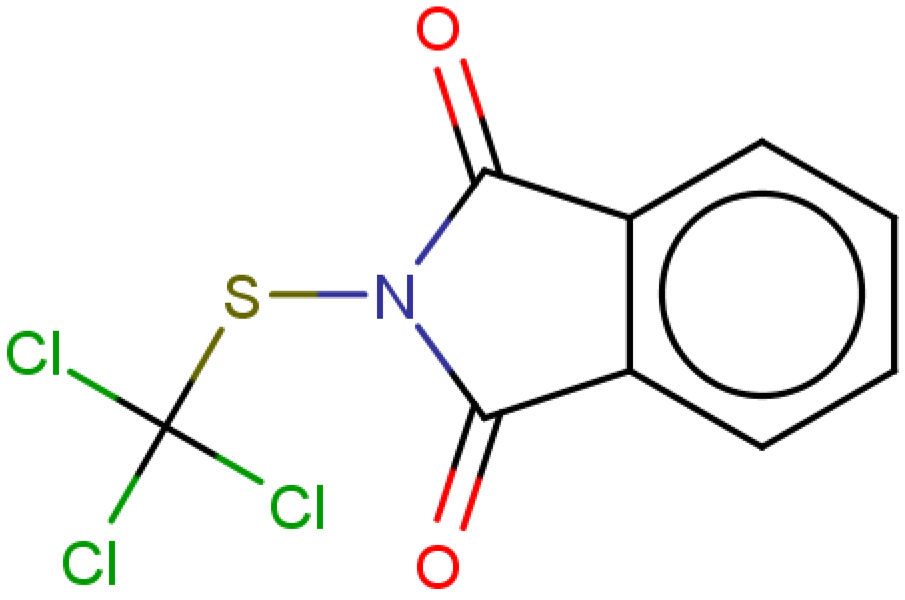

133-07-3
Name: Folpet
pIC50: 4.56
Rank: 485
Classes: antimicrobial--Pesticide--TSCA

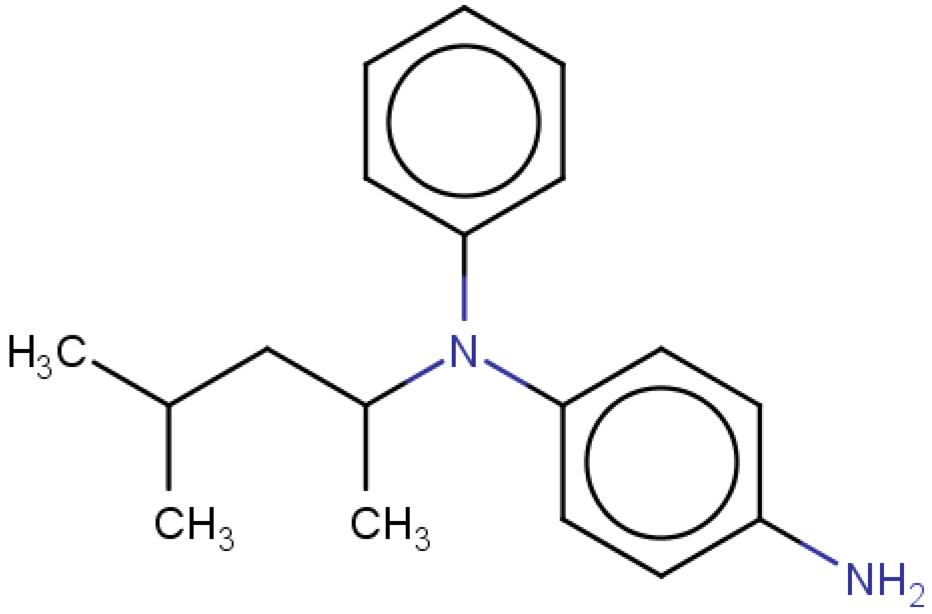

61931-82-6
Name: N-(4-Methylpentan-2-yl)-N-phenylbenz[illegible]
pIC50: 4.56
Rank: 486
Classes: No defined

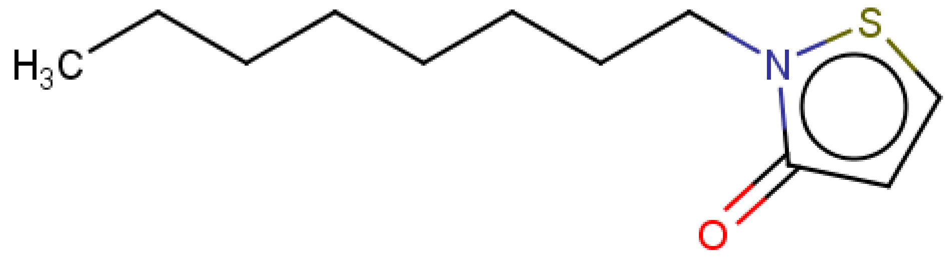

26530-20-1
Name: Octhilinone
pIC50: 4.56
Rank: 487
Classes: antimicrobial--TSCA

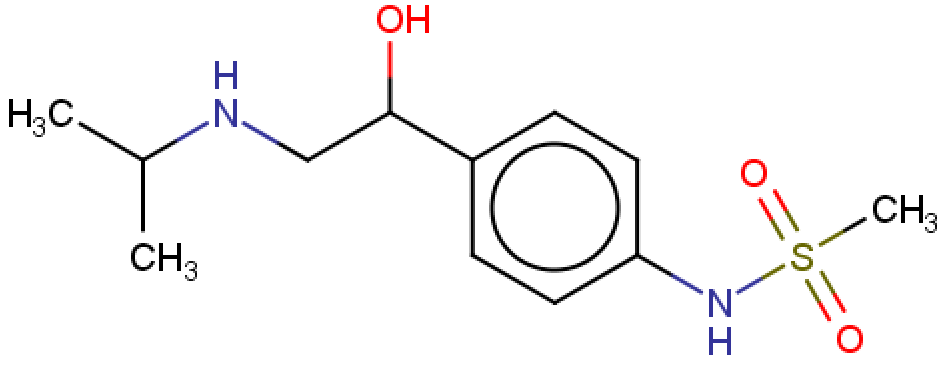

959-24-0
Name: Sotalol hydrochloride
pIC50: 4.56
Rank: 488
Classes: No defined

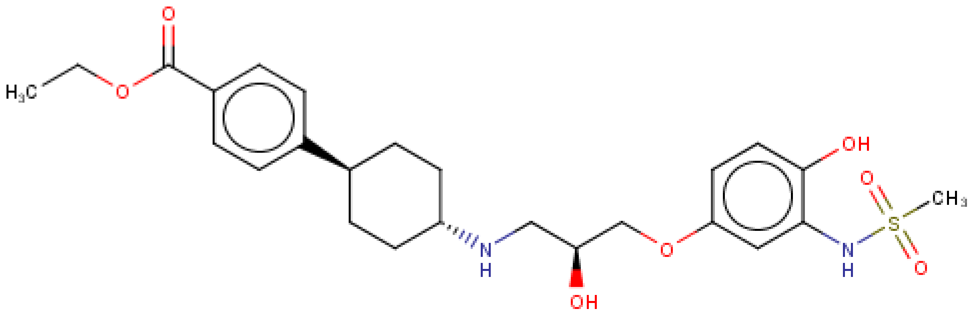

433212-21-6
Name: SAR 150640
pIC50: 4.55
Rank: 489
Classes: No defined

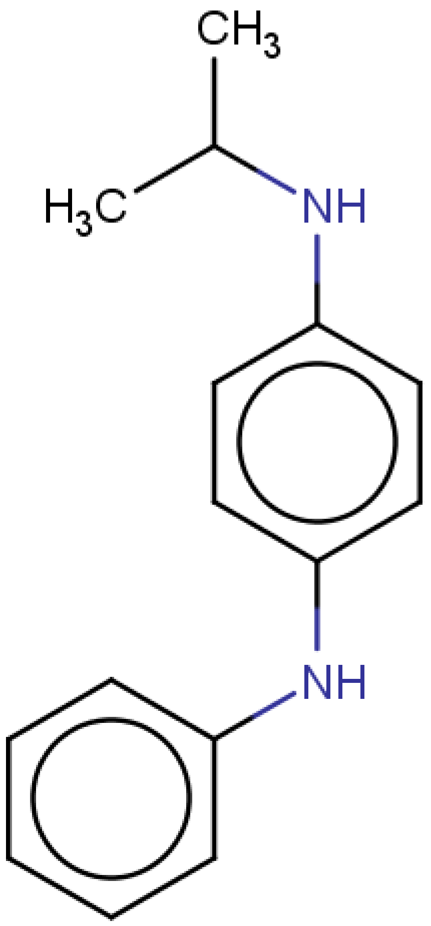

101-72-4
Name: N-Isopropyl-N'-phenyl-p-phenylenediamine
pIC50: 4.55
Rank: 490
Classes: rubber additive--TSCA

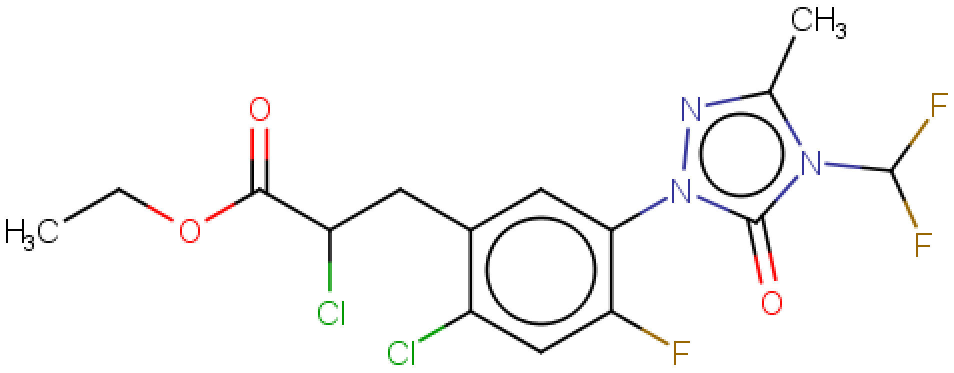

128639-02-1
Name: Carfentrazone-ethyl
pIC50: 4.54
Rank: 491
Classes: Pesticide

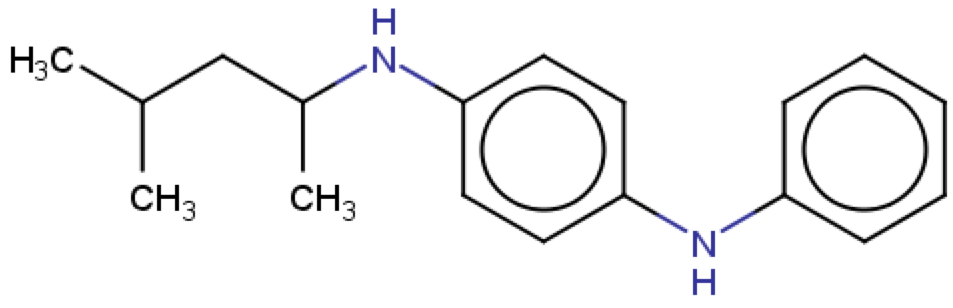

793-24-8
Name: N-(1 3-Dimethylbutyl)-N'-phenyl-p-phe
pIC50: 4.54
Rank: 492
Classes: rubber additive

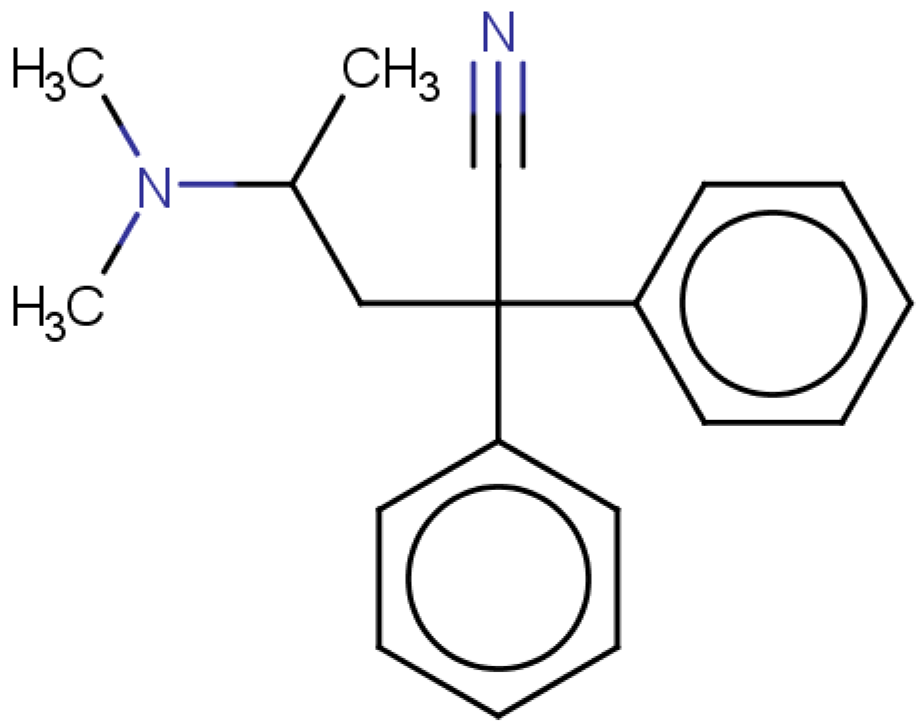

125-79-1
Name: 4-Dimethylamino-2 2-diphenylvaleronitrile
pIC50: 4.53
Rank: 493
Classes: No defined

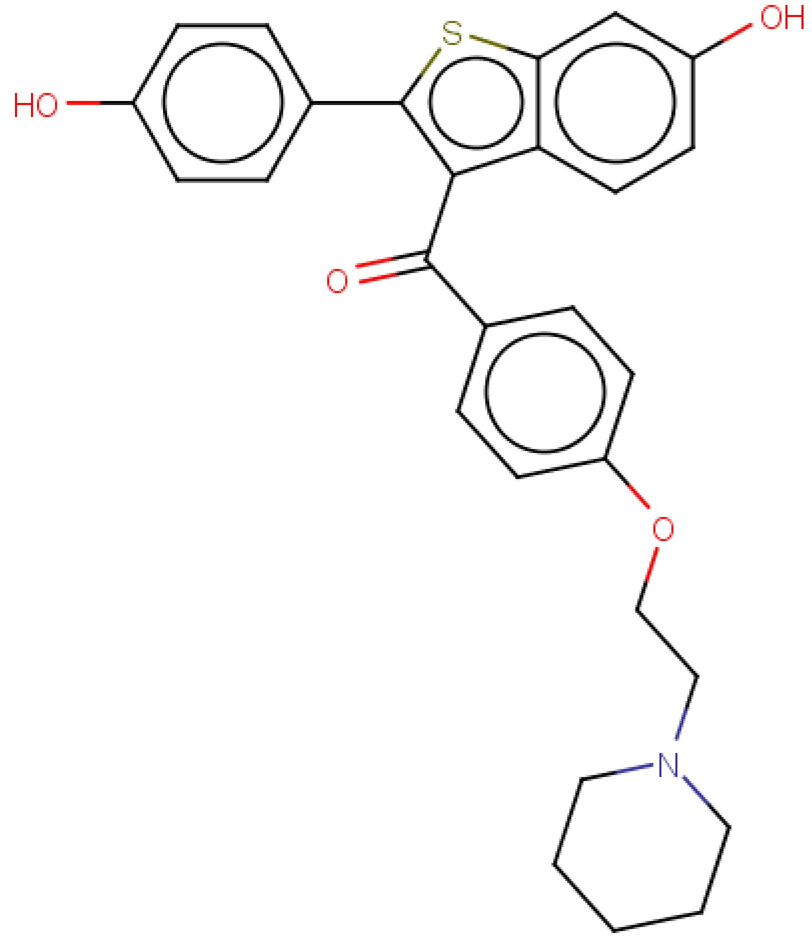

82640-04-8
Name: Raloxifene hydrochloride
pIC50: 4.53
Rank: 494
Classes: No defined

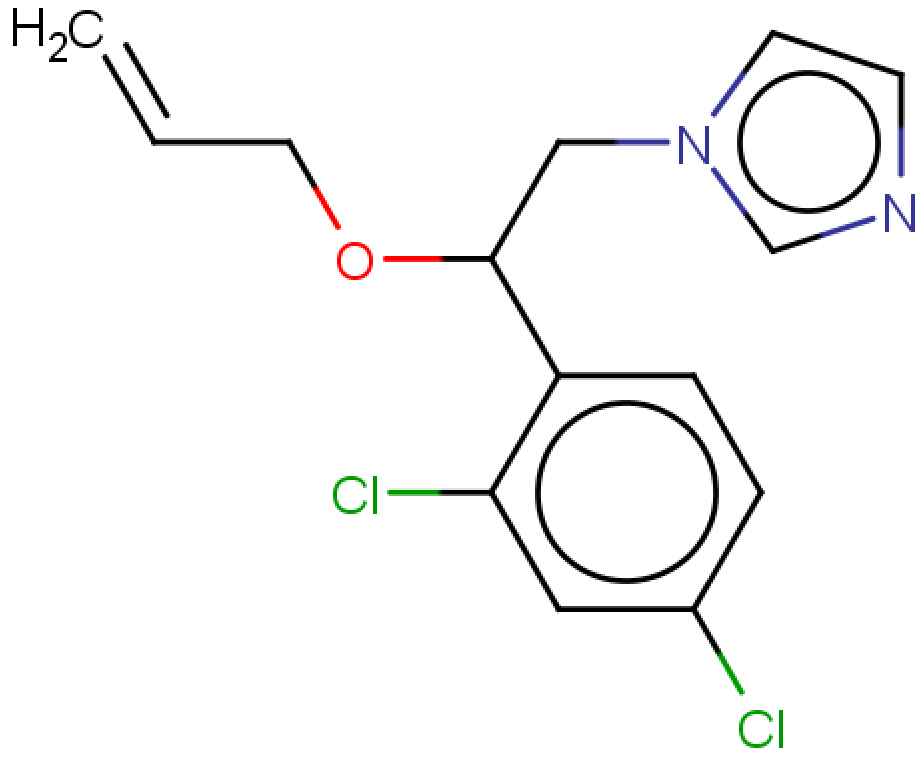

35554-44-0
Name: Imazalil
pIC50: 4.53
Rank: 495
Classes: Pesticide

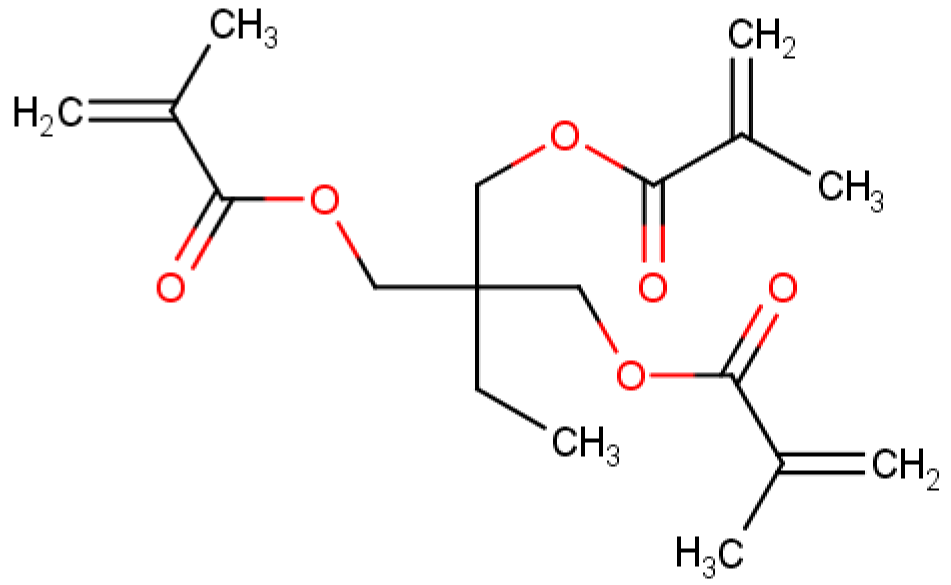

3290-92-4
Name: Trimethylolpropane trimethacrylate
pIC50: 4.52
Rank: 496
Classes: crosslinker--TSCA

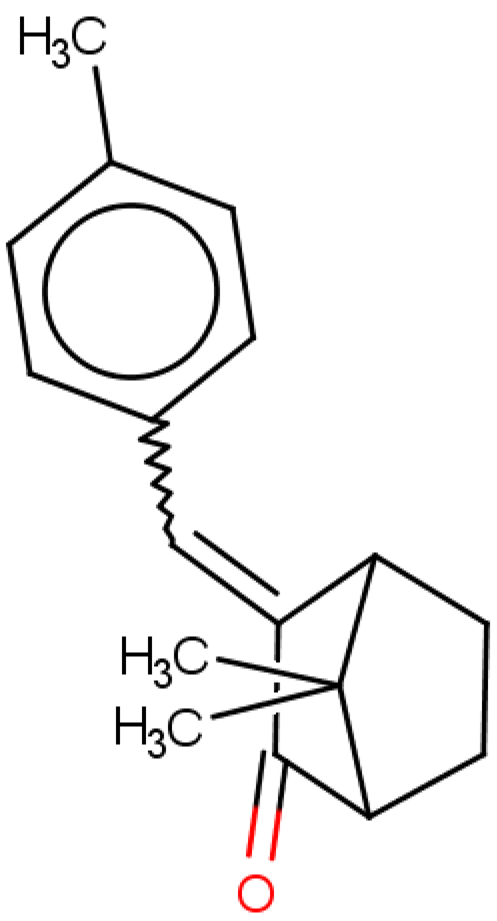

36861-47-9
Name: Enzacamene
pIC50: 4.51
Rank: 497
Classes: UV absorber--TSCA

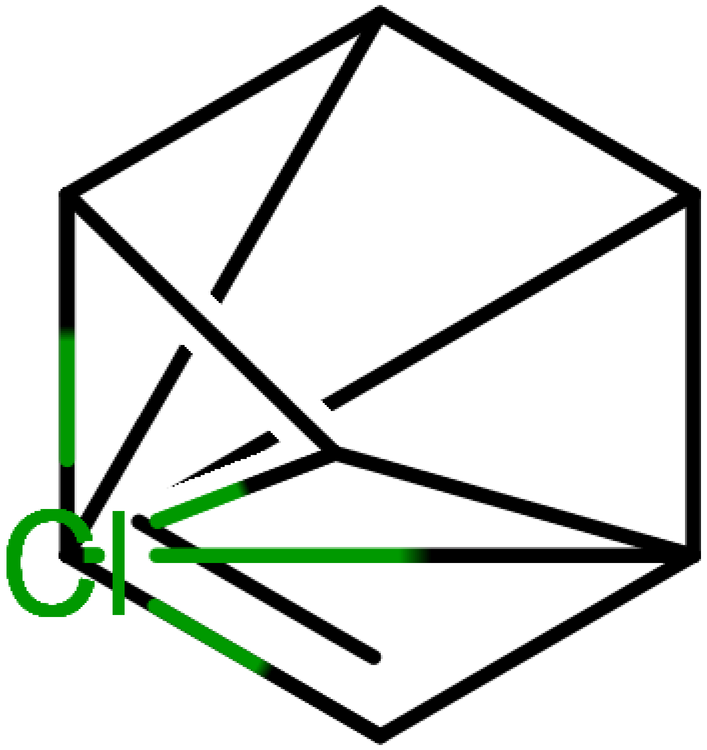

57-74-9
Name: Chlordane
pIC50: 4.51
Rank: 498
Classes: No defined

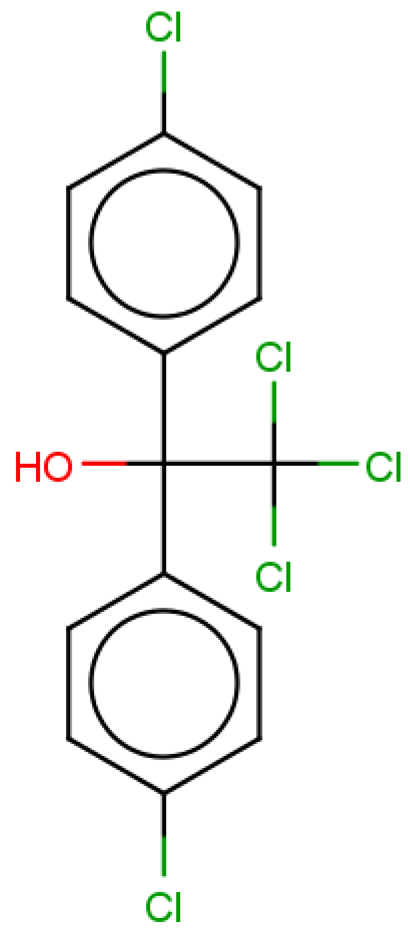

115-32-2
Name: Dicofol
pIC50: 4.51
Rank: 499
Classes: flame retardant--Pesticide

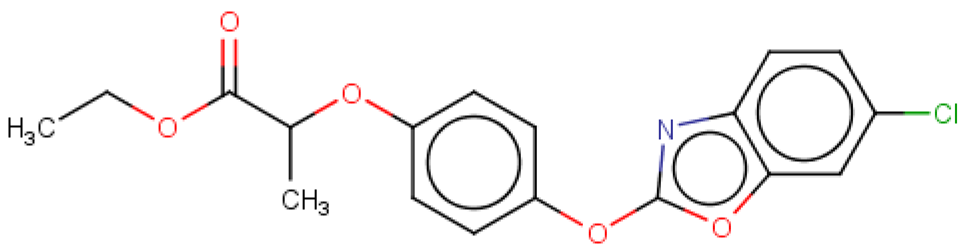

66441-23-4
Name: Fenoxaprop-ethyl
pIC50: 4.51
Rank: 500
Classes: Drug

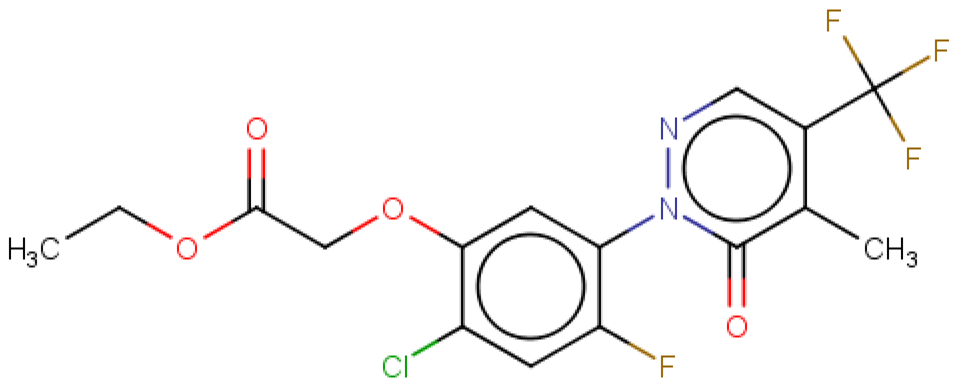

188489-07-8
Name: Flufenpyr-ethyl
pIC50: 4.51
Rank: 501
Classes: Pesticide

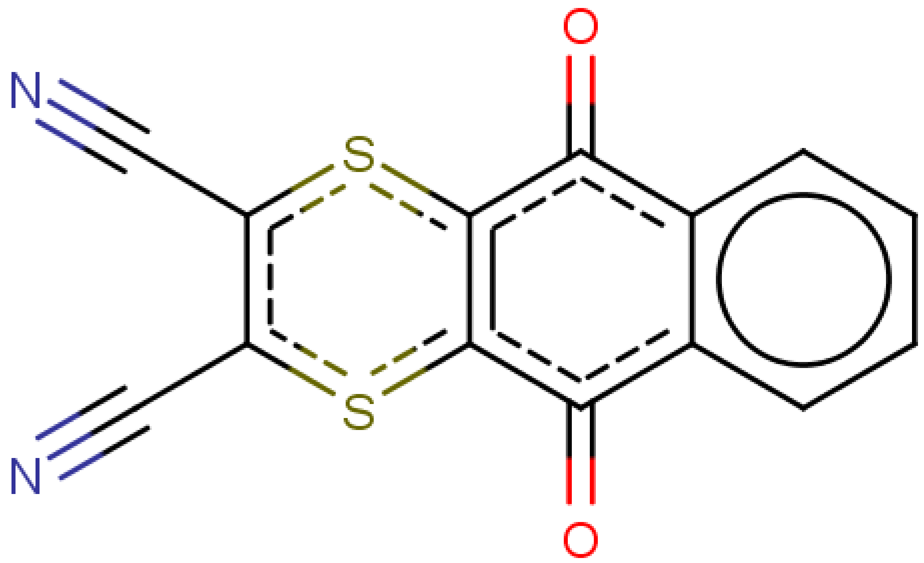

3347-22-6
Name: Dithianon
pIC50: 4.51
Rank: 502
Classes: Pesticide

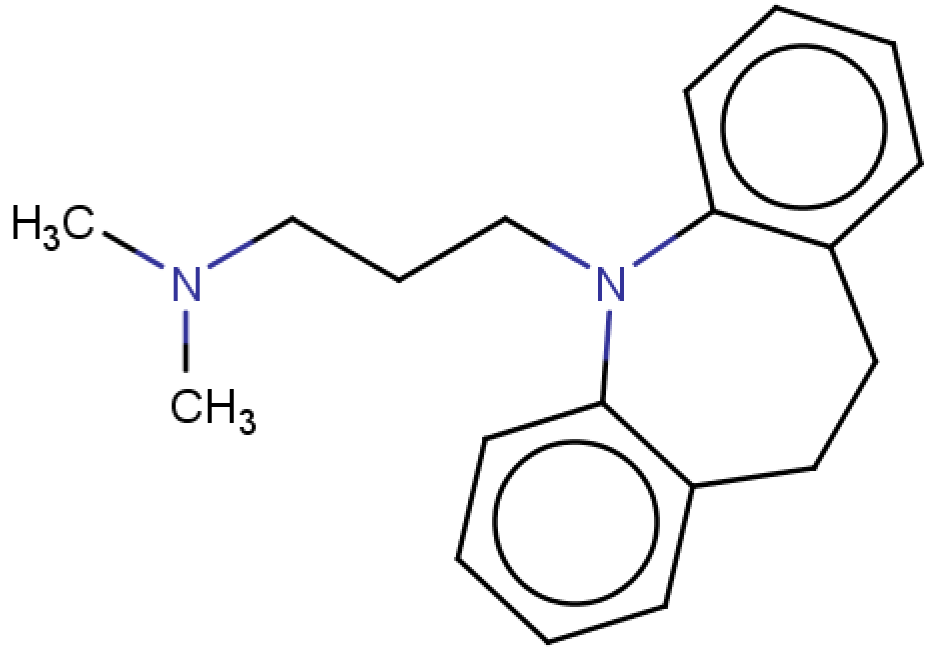

113-52-0
Name: Imipramine hydrochloride
pIC50: 4.51
Rank: 503
Classes: No defined

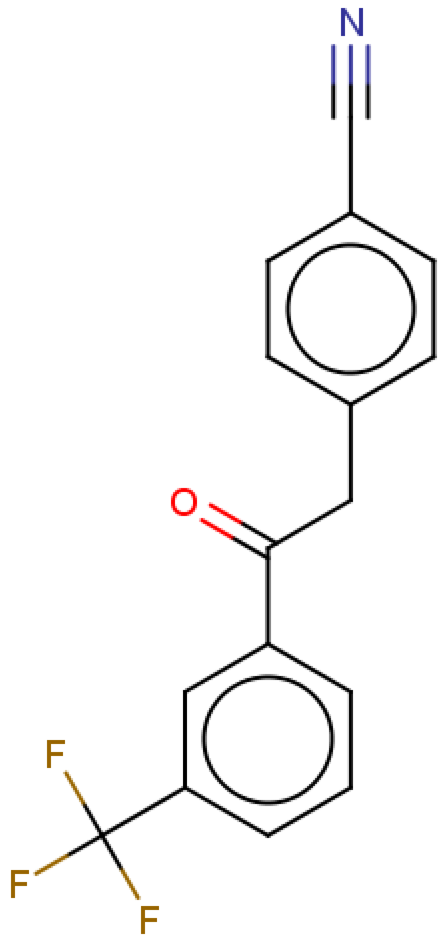

146653-56-7
Name: 4-{2-Oxo-2-[3-(trifluoromethyl)phenyl]e
pIC50: 4.51
Rank: 504
Classes: No defined

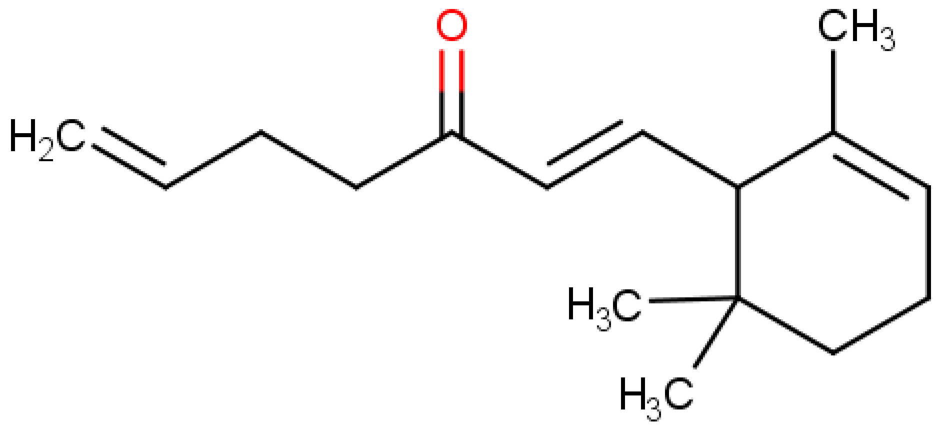

79-78-7
Name: Allyl Ionone
pIC50: 4.51
Rank: 505
Classes: fragrance

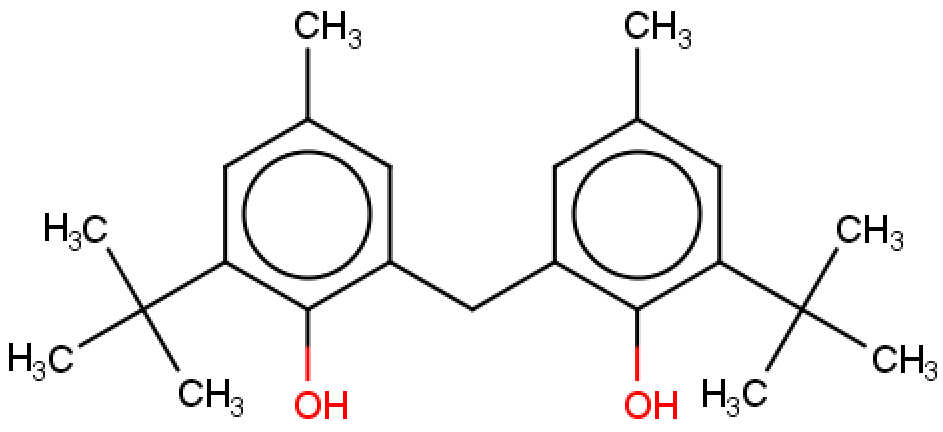

119-47-1
Name: 2 2'-Methylenebis(4-methyl-6-tert-butylphenol)
pIC50: 4.51
Rank: 506
Classes: heat stabilizer--TSCA

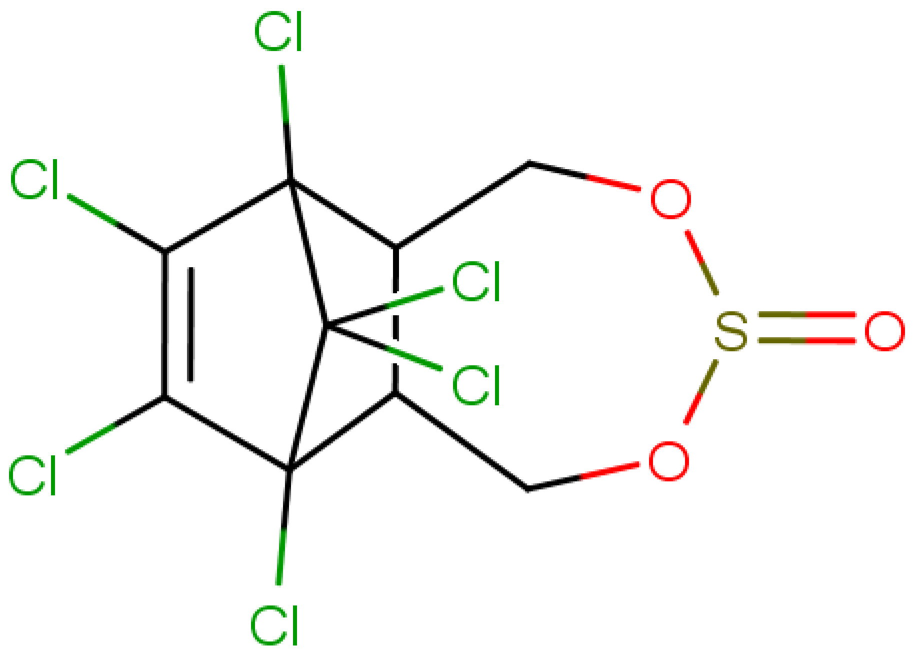

115-29-7
Name: Endosulfan
pIC50: 4.49
Rank: 507
Classes: flame retardant--Pesticide

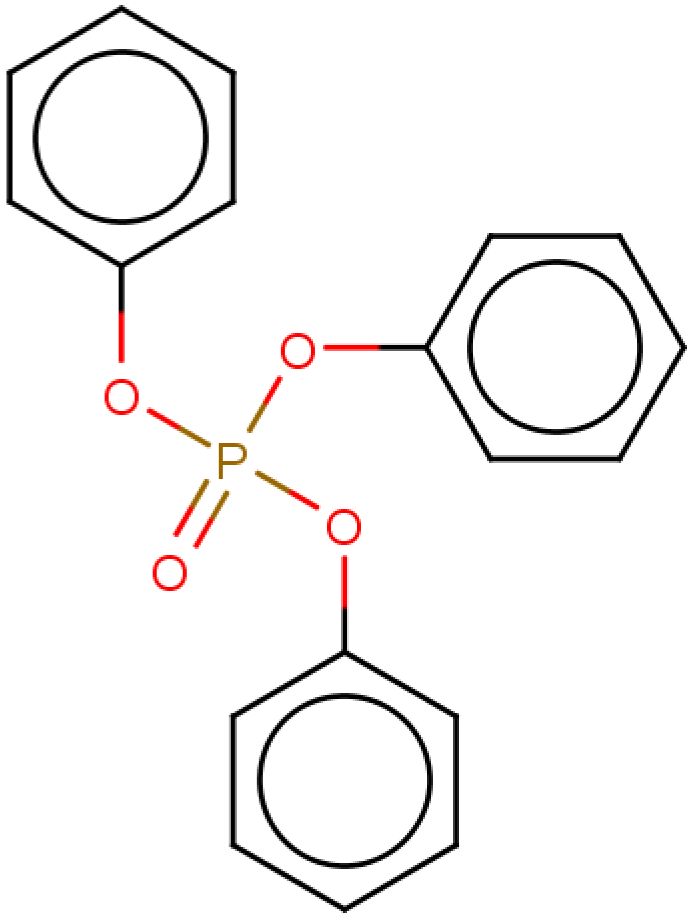

115-86-6
Name: Triphenyl phosphate
pIC50: 4.49
Rank: 508
Classes: flame retardant--TSCA

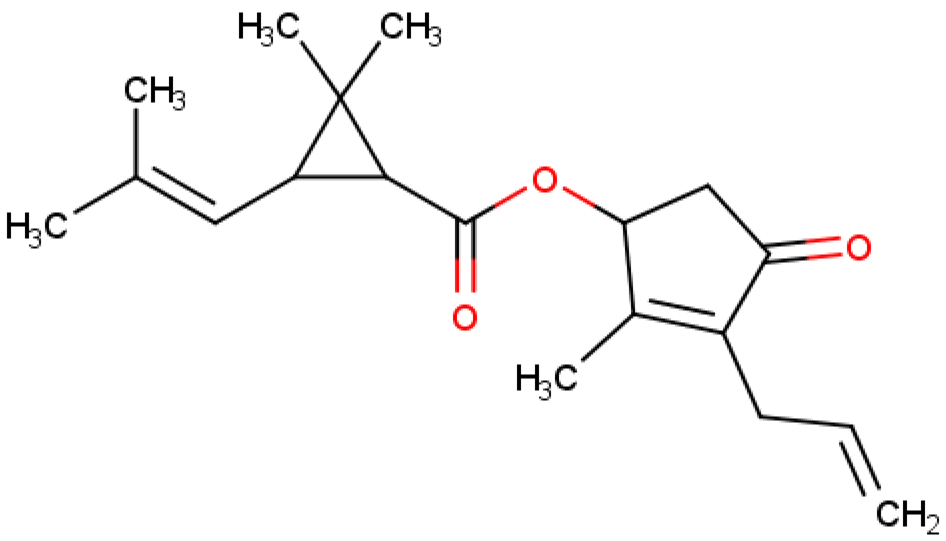

584-79-2
Name: Allethrin
pIC50: 4.49
Rank: 509
Classes: masking agent--Pesticide--TSCA

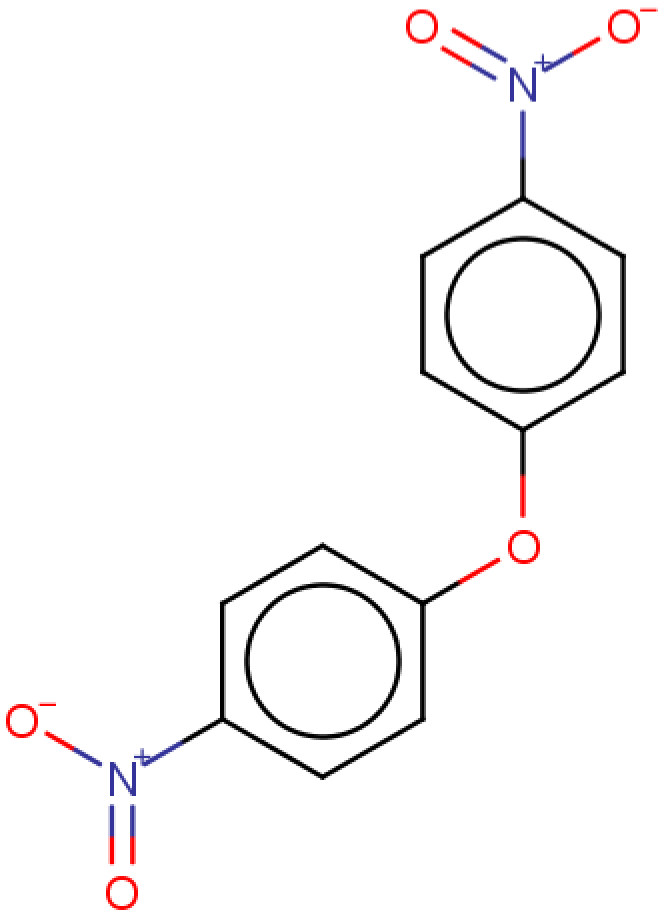

101-63-3
Name: 1 1'-Oxybis(4-nitrobenzene)
pIC50: 4.48
Rank: 510
Classes: No defined

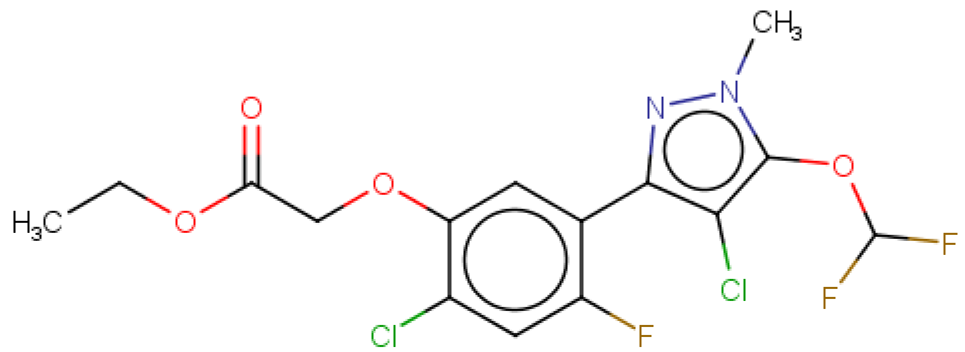

129630-19-9
Name: Pyraflufen-ethyl
pIC50: 4.48
Rank: 511
Classes: Pesticide

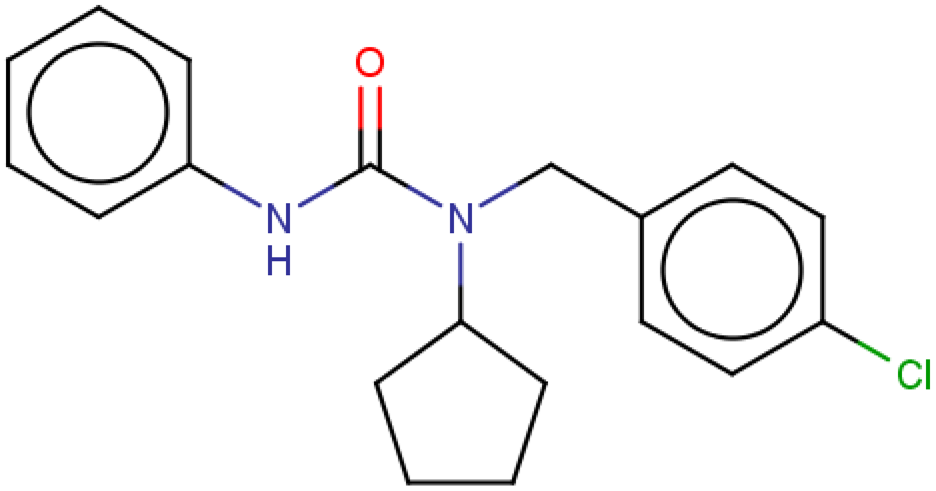

66063-05-6
Name: Pencycuron
pIC50: 4.48
Rank: 512
Classes: No defined

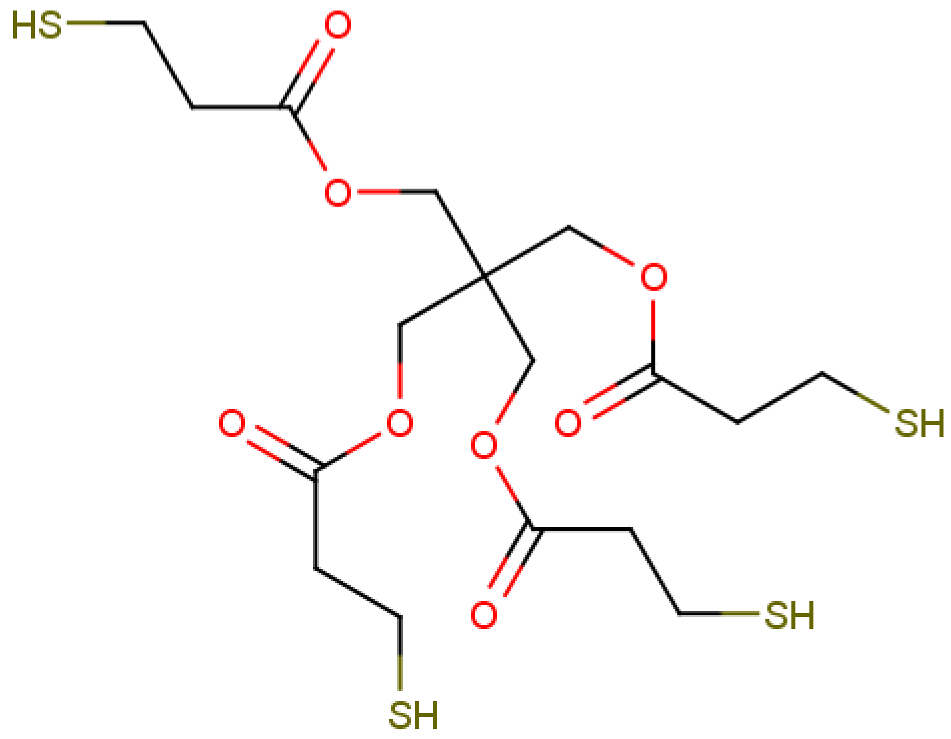

7575-23-7
Name: 3-[(3-Sulfanylpropanoyl)oxy]-2 2-bis{[(3
pIC50: 4.48
Rank: 513
Classes: crosslinker

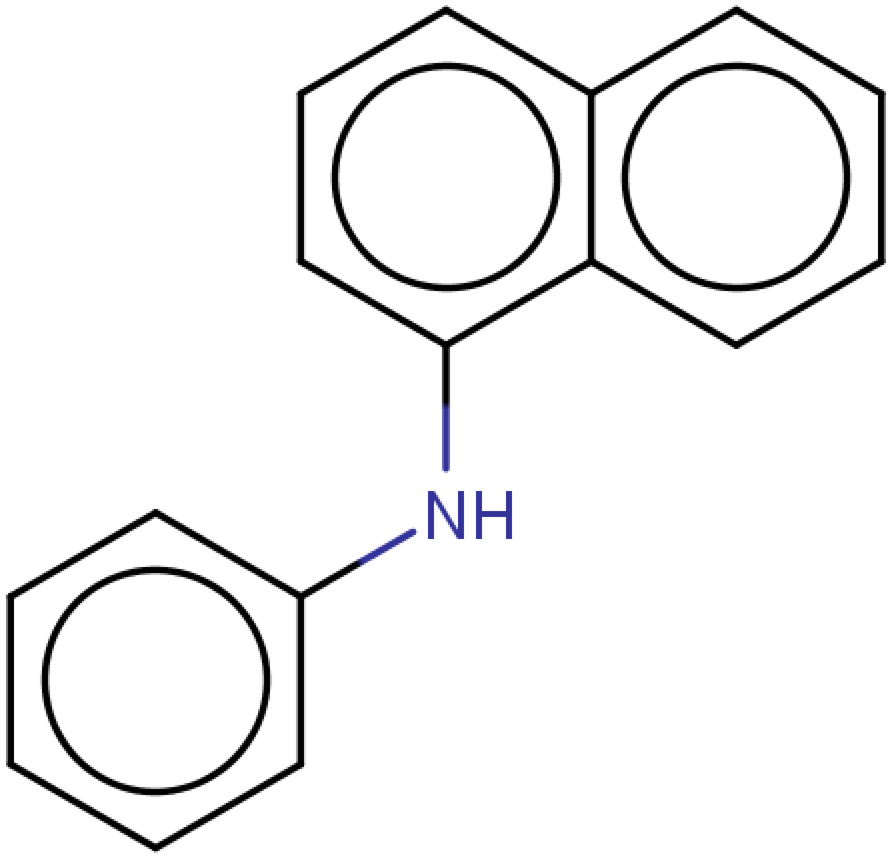

90-30-2
Name: N-Phenyl-1-naphthylamine
pIC50: 4.48
Rank: 514
Classes: antioxidant--TSCA

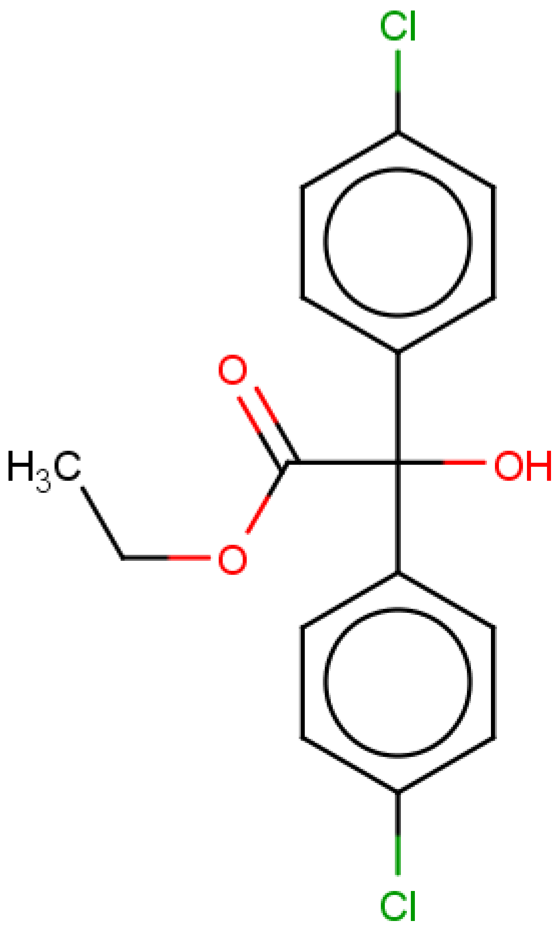

510-15-6
Name: Chlorobenzilate
pIC50: 4.47
Rank: 515
Classes: No defined

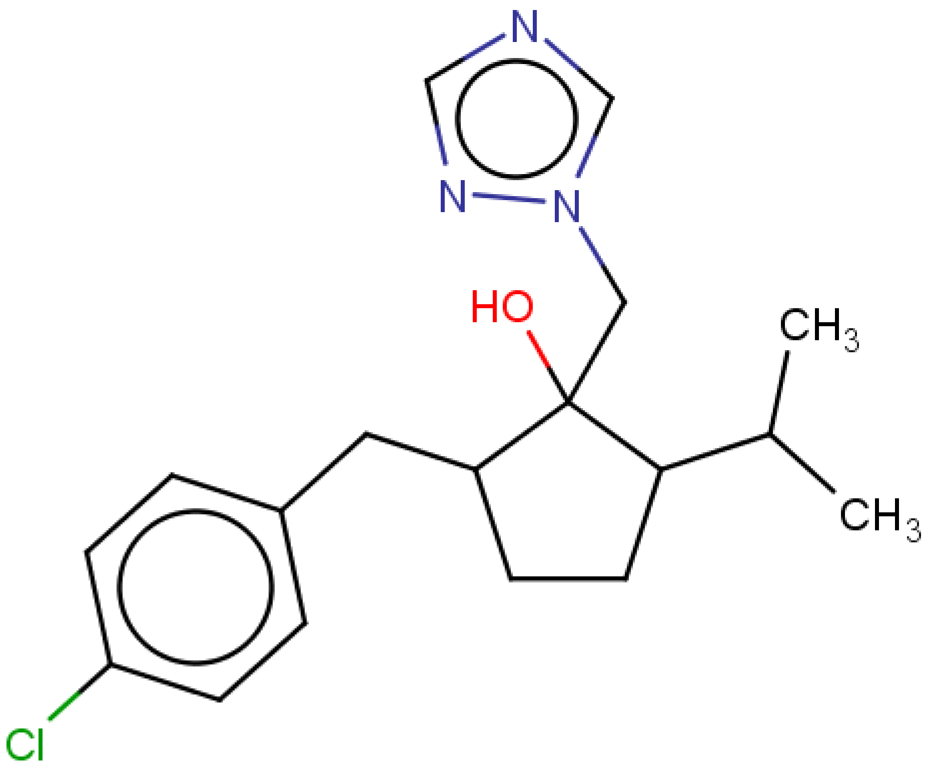

125225-28-7
Name: Ipconazole
pIC50: 4.46
Rank: 516
Classes: Pesticide

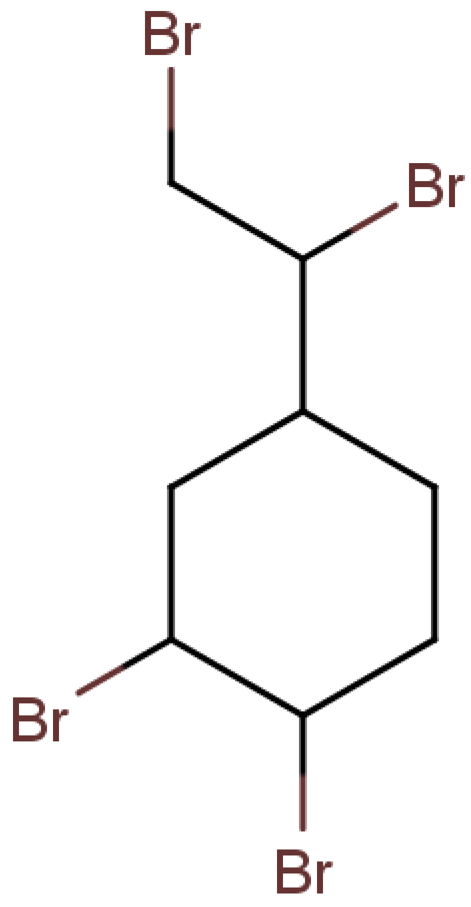

3322-93-8
Name: 1 2-Dibromo-4-(1 2-dibromoethyl)cyclo[illegible]
pIC50: 4.46
Rank: 517
Classes: No defined

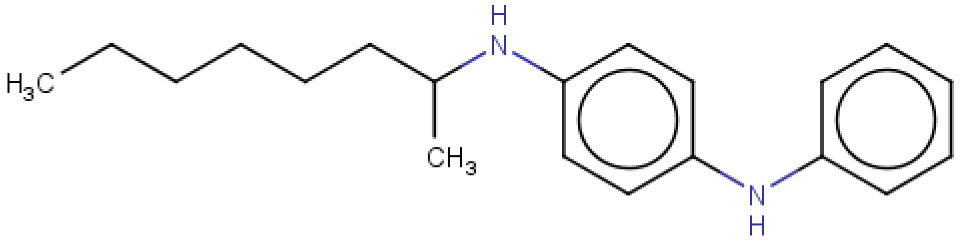

15233-47-3
Name: N-(1-Methylheptyl)-N'-phenyl-1 4-benze[illegible]
pIC50: 4.46
Rank: 518
Classes: rubber additive

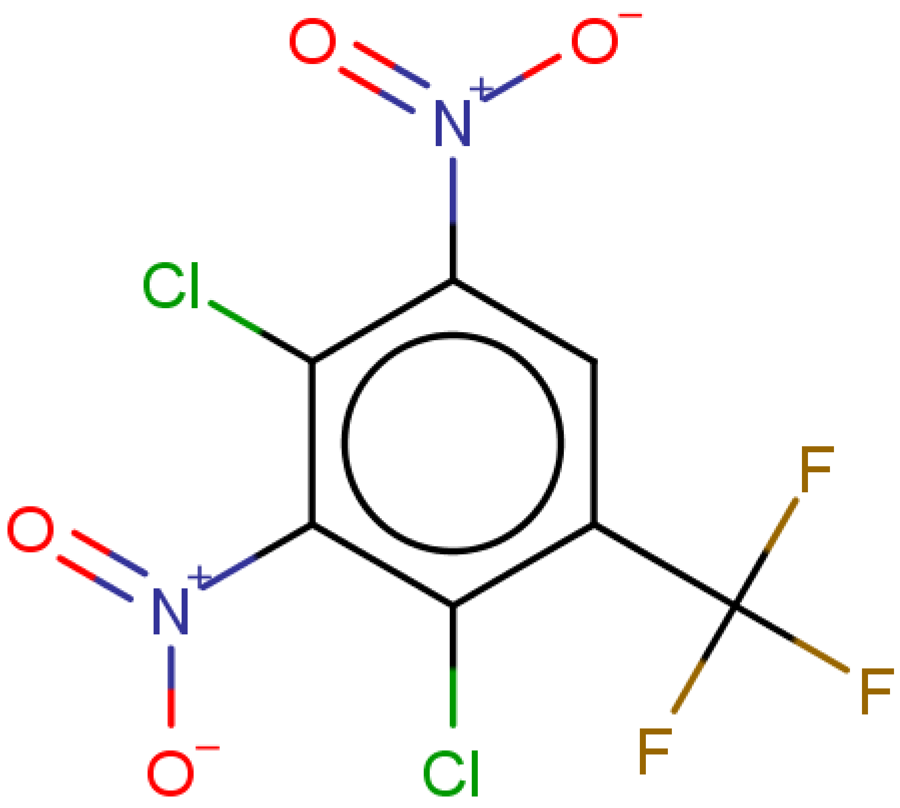

29091-09-6
Name: 2 4-Dichloro-1 3-dinitro-5-(trifluoromet[illegible]
pIC50: 4.46
Rank: 519
Classes: TSCA

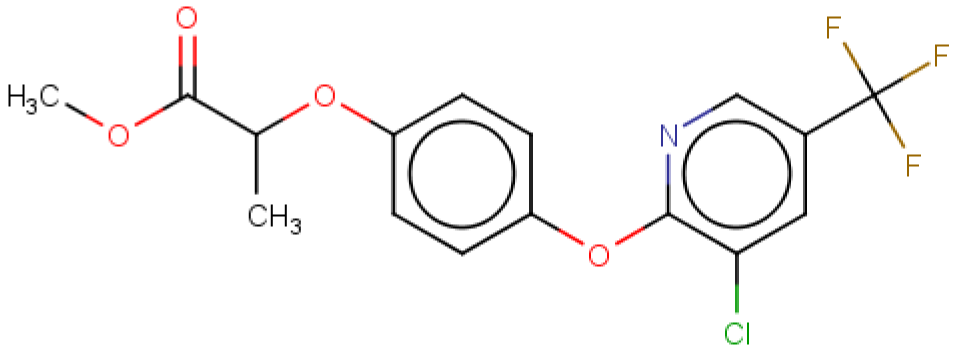

69806-40-2
Name: Haloxyfop-methyl
pIC50: 4.45
Rank: 520
Classes: No defined

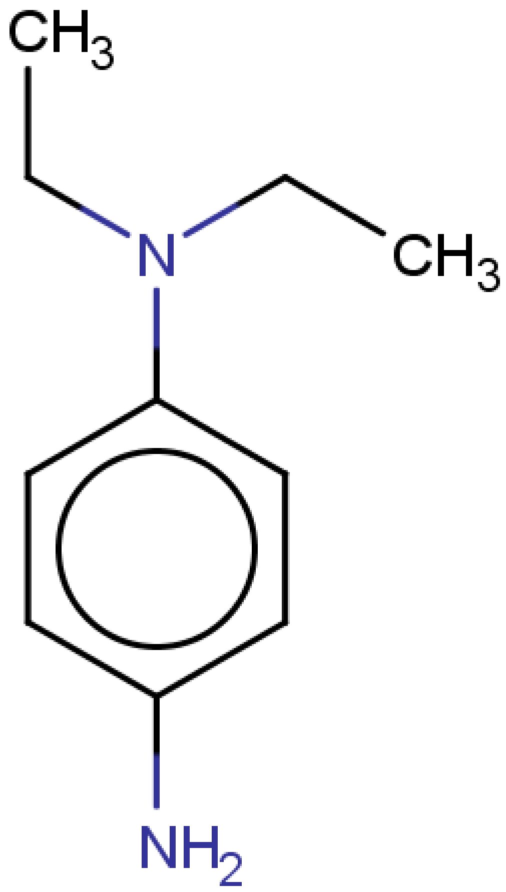

93-05-0
Name: N N-Diethylbenzene-1 4-diamine
pIC50: 4.45
Rank: 521
Classes: No defined

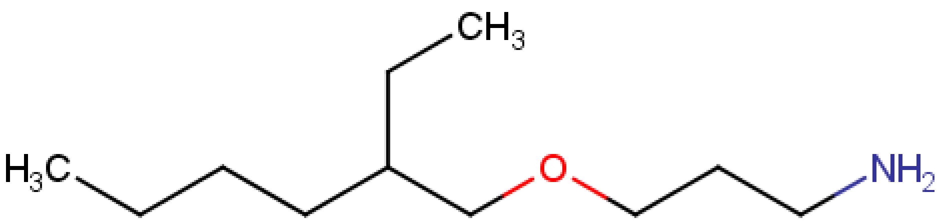

5397-31-9
Name: 3-((2-Ethylhexyl)oxy)propylamine
pIC50: 4.45
Rank: 522
Classes: No defined

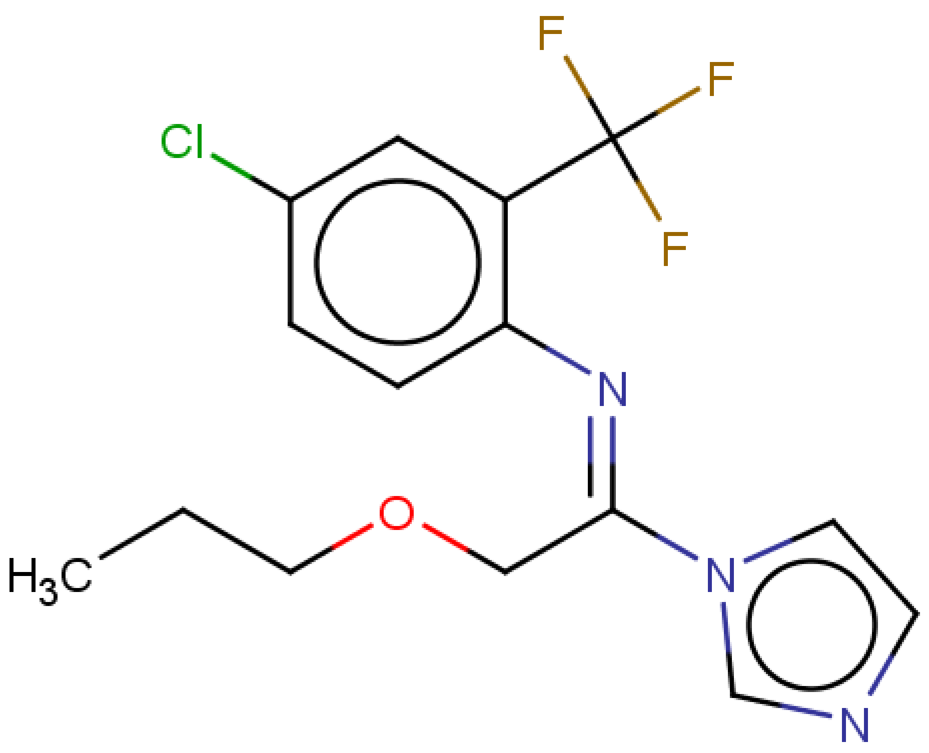

68694-11-1
Name: Triflumizole
pIC50: 4.44
Rank: 523
Classes: Pesticide

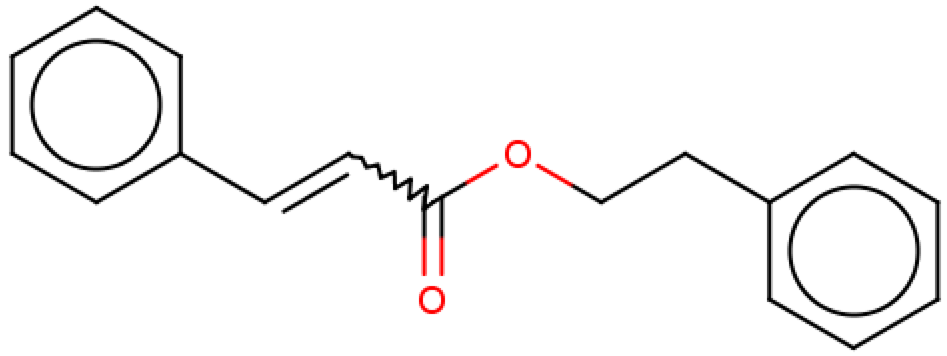

103-53-7
Name: 2-Phenylethyl 3-phenylprop-2-enoate
pIC50: 4.44
Rank: 524
Classes: fragrance

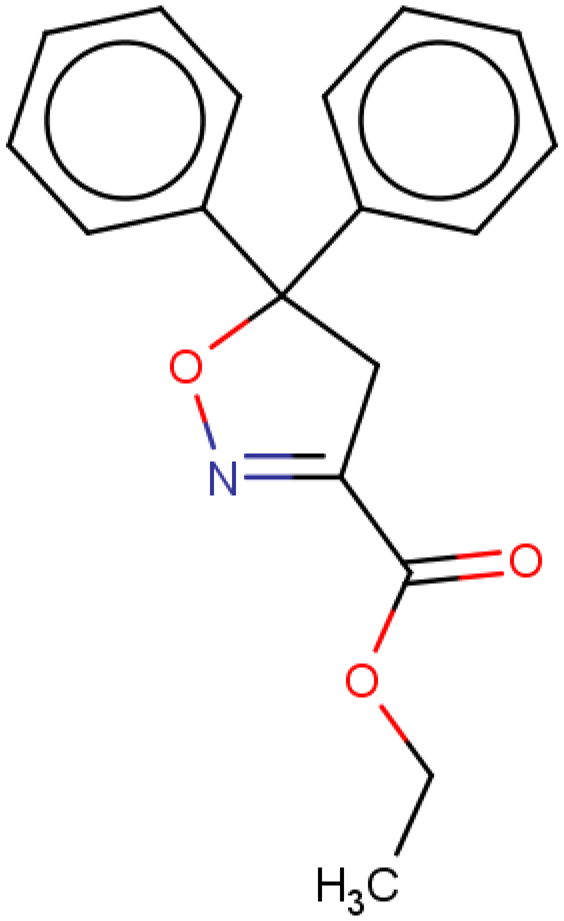

163520-33-0
Name: Isoxadifen-ethyl
pIC50: 4.44
Rank: 525
Classes: Pesticide

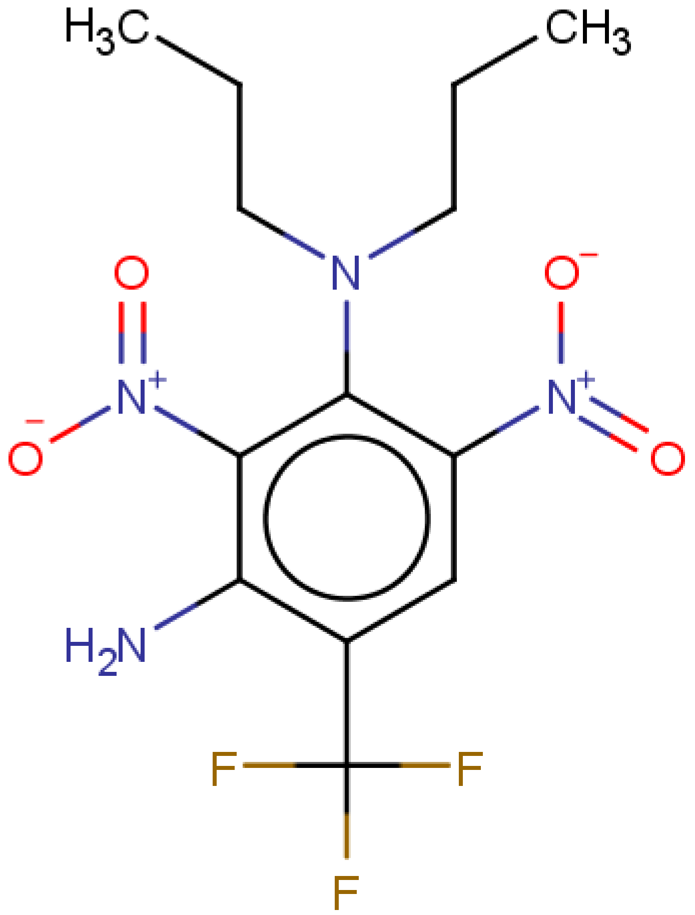

29091-21-2
Name: Prodiamine
pIC50: 4.43
Rank: 526
Classes: Pesticide--TSCA

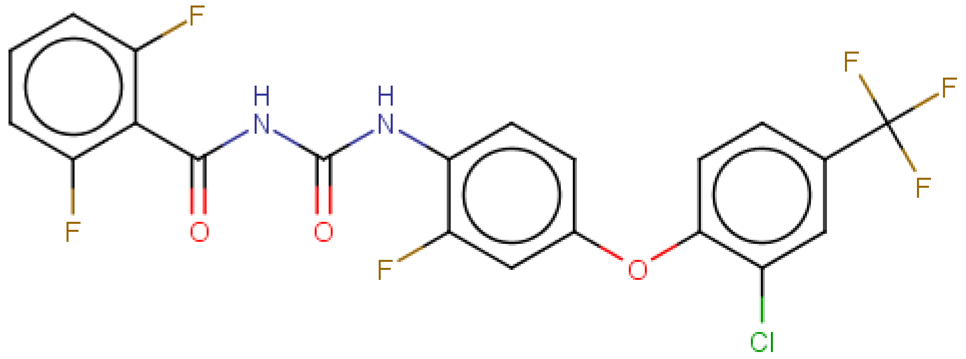

101463-69-8
Name: Flufenoxuron
pIC50: 4.43
Rank: 527
Classes: Pesticide

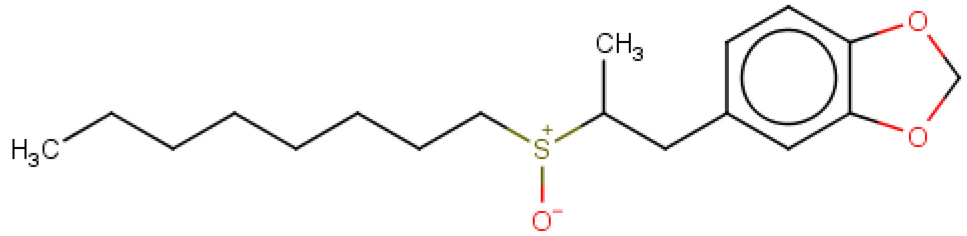

120-62-7
Name: Piperonyl sulfoxide
pIC50: 4.42
Rank: 528
Classes: No defined

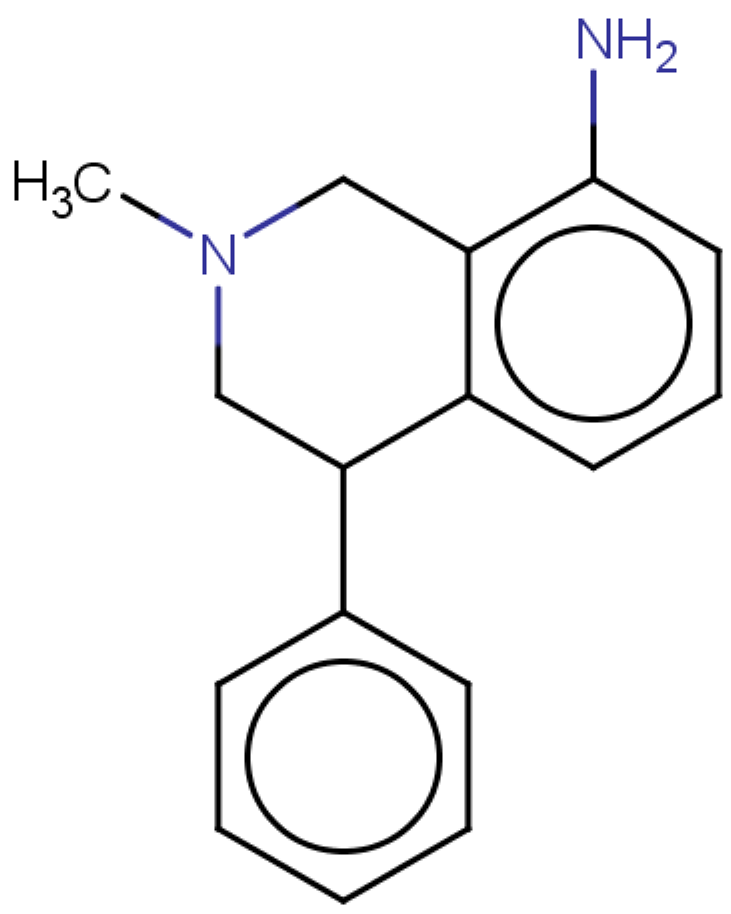

24526-64-5
Name: Nomifensine
pIC50: 4.41
Rank: 529
Classes: Drug

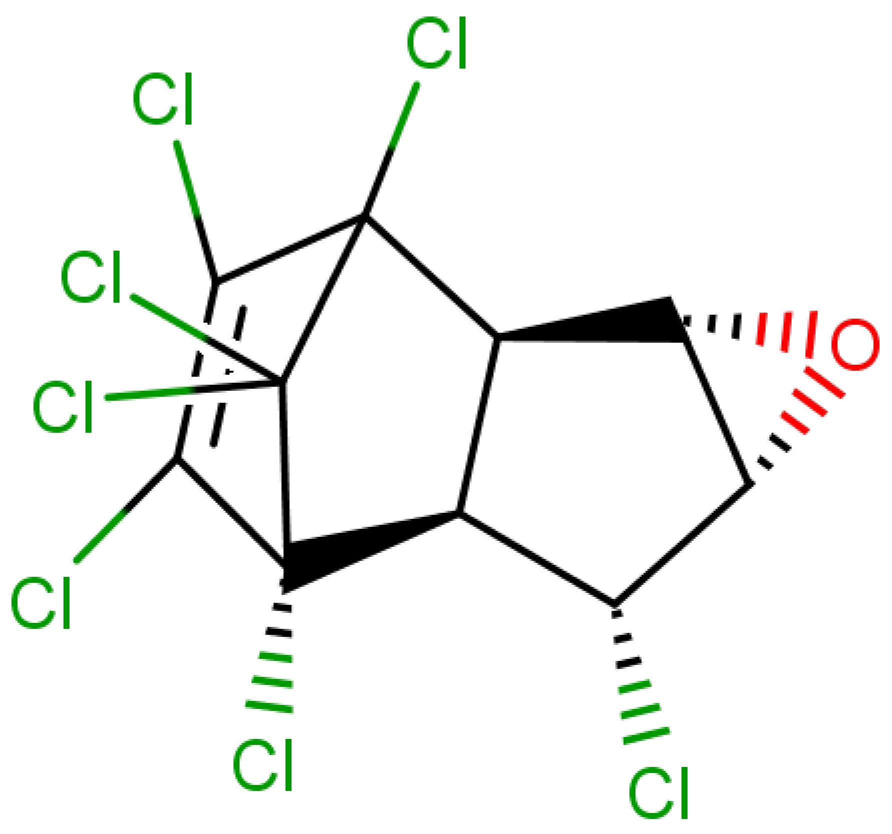

1024-57-3
Name: Heptachlor epoxide B
pIC50: 4.41
Rank: 530
Classes: No defined

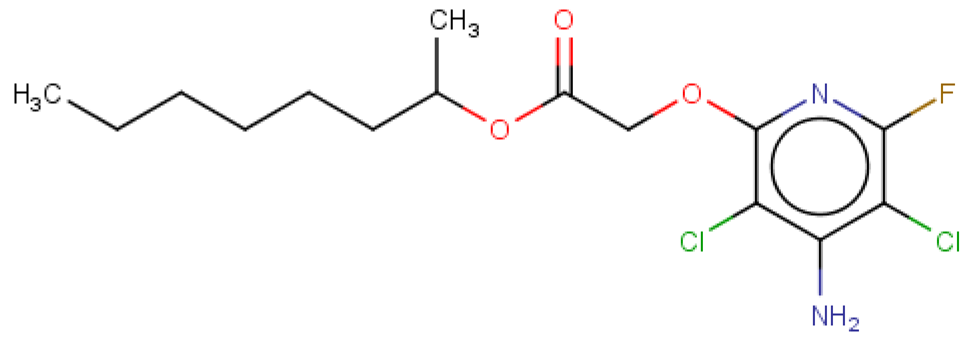

81406-37-3
Name: Fluroxypyr-meptyl
pIC50: 4.41
Rank: 531
Classes: Pesticide

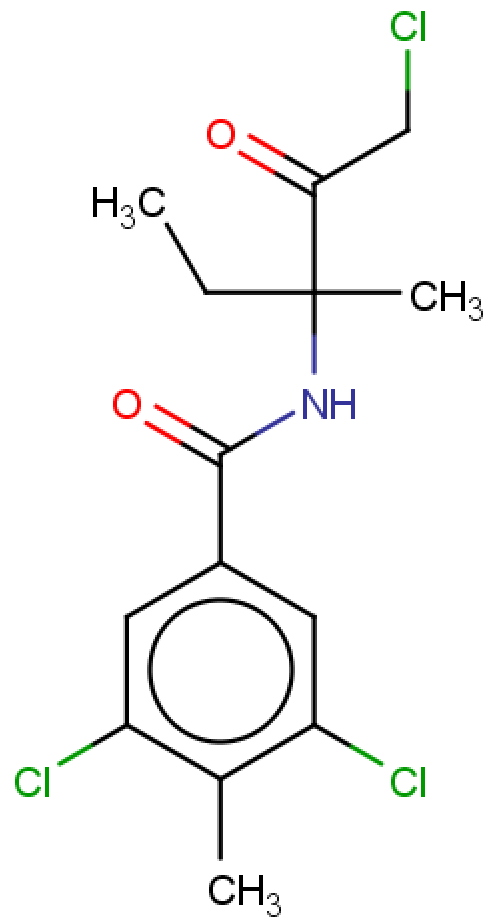

156052-68-5
Name: Zoxamide
pIC50: 4.41
Rank: 532
Classes: Pesticide

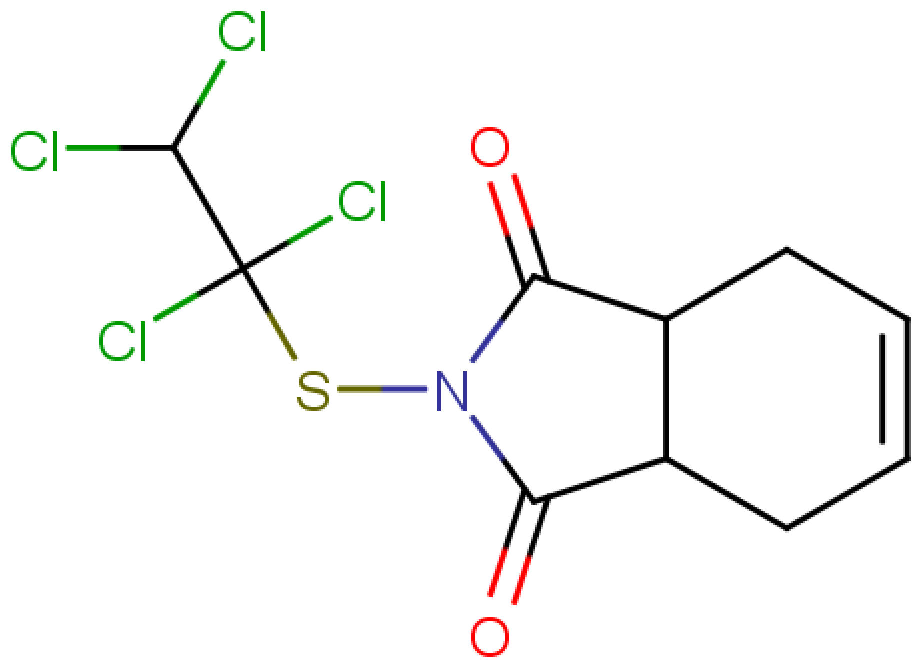

2425-06-1
Name: Captafol
pIC50: 4.41
Rank: 533
Classes: No defined

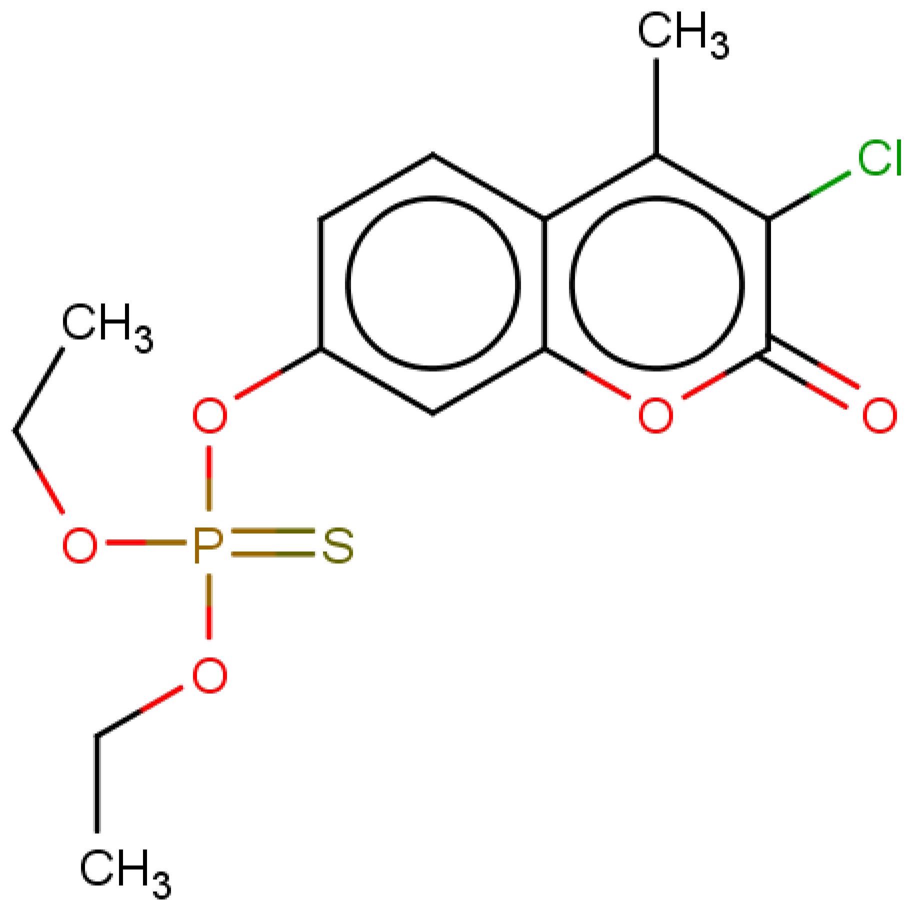

56-72-4
Name: Coumaphos
pIC50: 4.39
Rank: 534
Classes: Pesticide

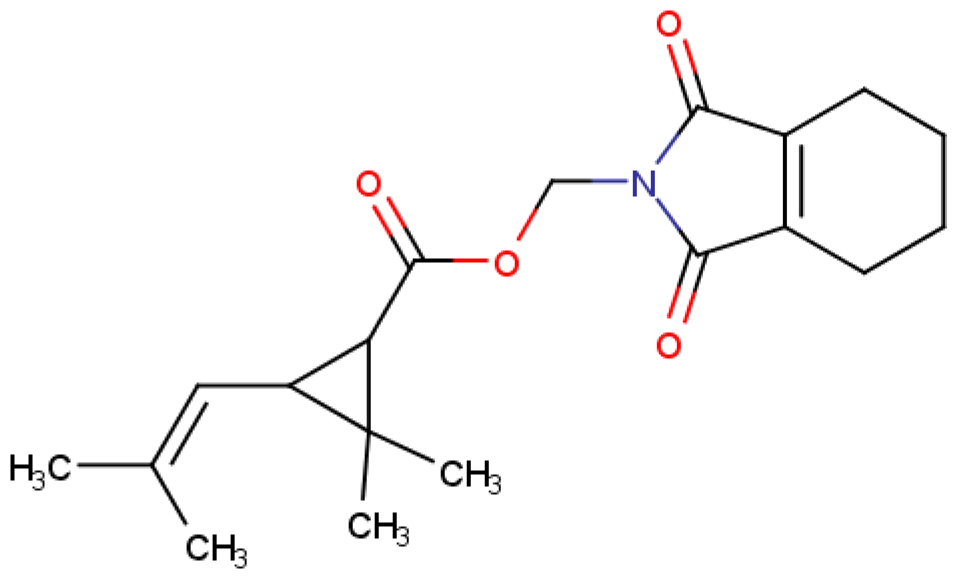

7696-12-0
Name: Tetramethrin
pIC50: 4.39
Rank: 535
Classes: antimicrobial--Pesticide

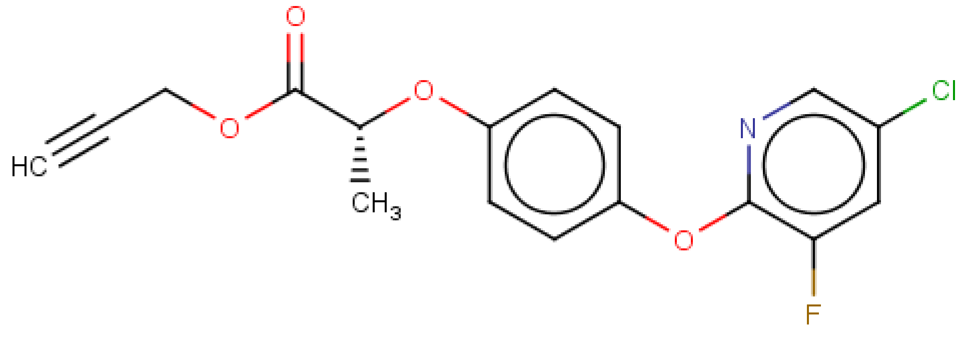

105512-06-9
Name: Clodinafop-propargyl
pIC50: 4.39
Rank: 536
Classes: Pesticide

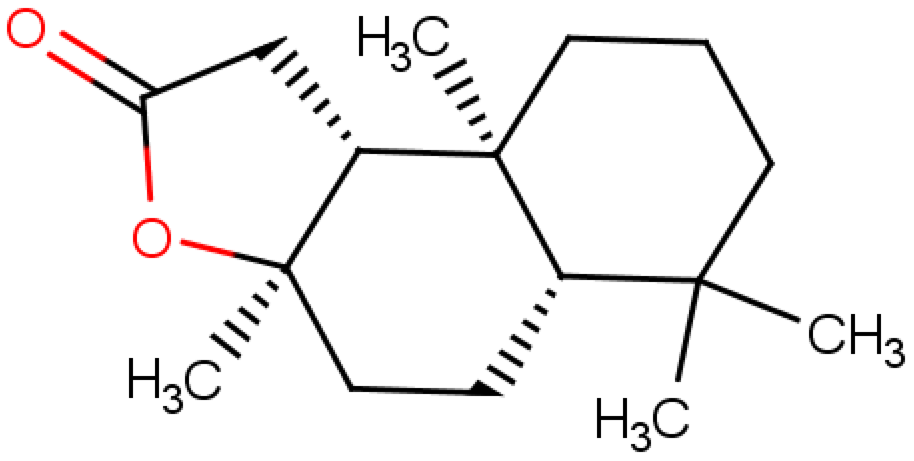

564-20-5
Name: (3aR)-(+)-Sclareolide
pIC50: 4.38
Rank: 537
Classes: masking agent

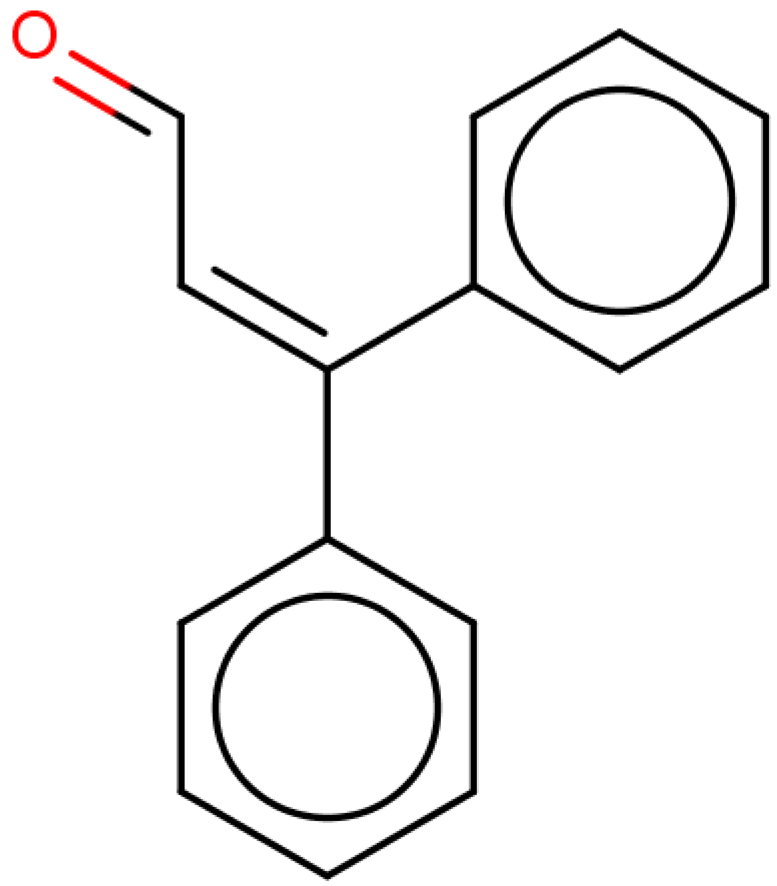

1210-39-5
Name: 3 3-Diphenylacrylaldehyde
pIC50: 4.37
Rank: 538
Classes: No defined

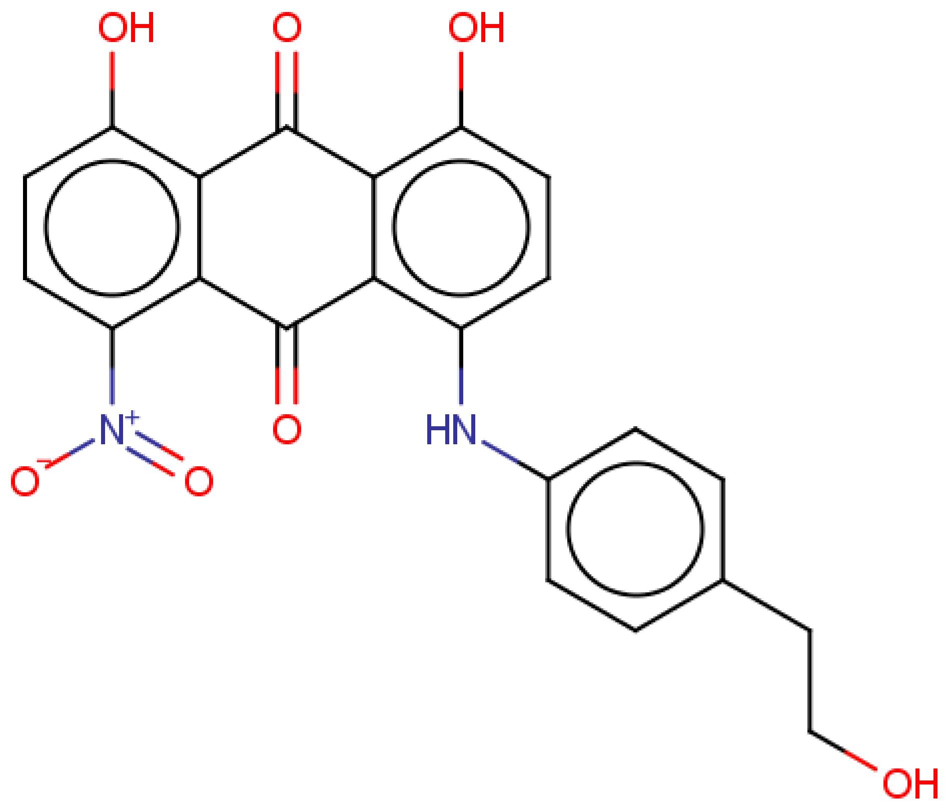

15791-78-3
Name: C.I. Disperse Blue 27
pIC50: 4.36
Rank: 539
Classes: No defined

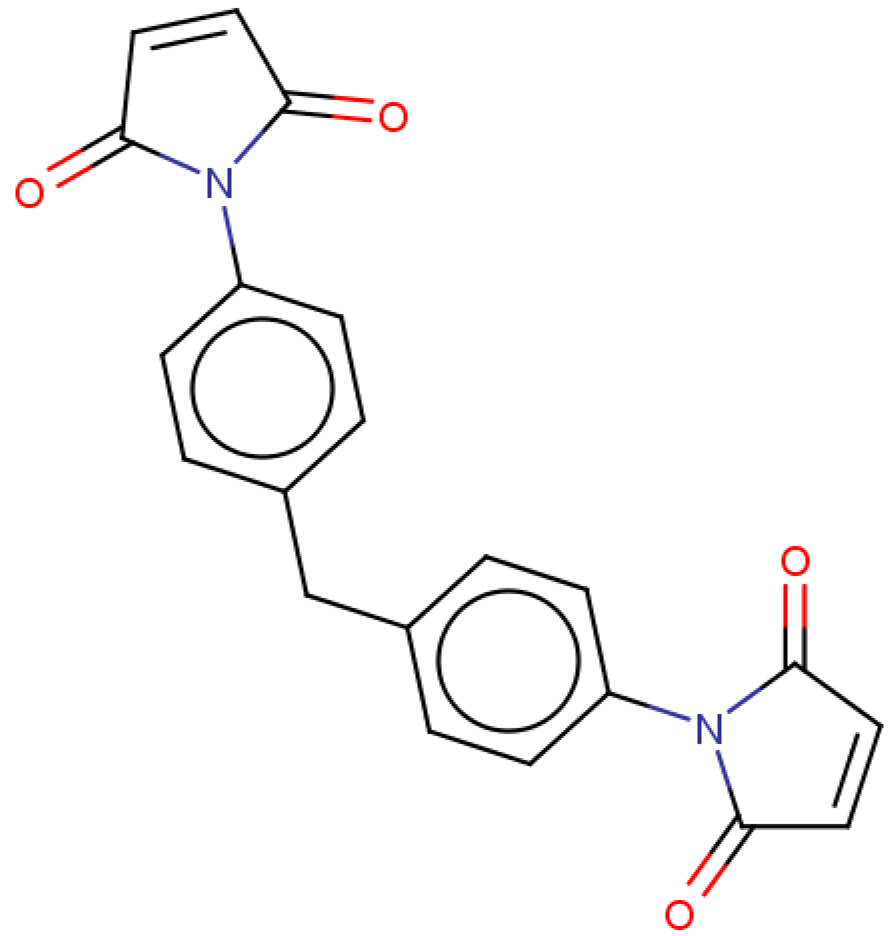

13676-54-5
Name: Bismaleimide
pIC50: 4.36
Rank: 540
Classes: polyimide--TSCA

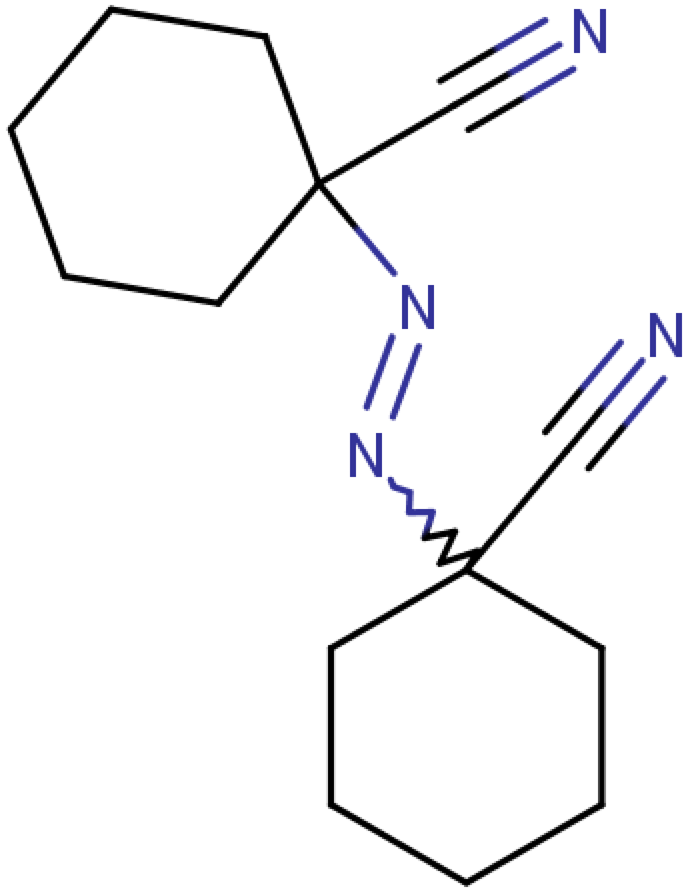

2094-98-6
Name: 1 1'-Diazene-1 2-diyldicyclohexanecarbonitrile
pIC50: 4.36
Rank: 541
Classes: catalyst--TSCA

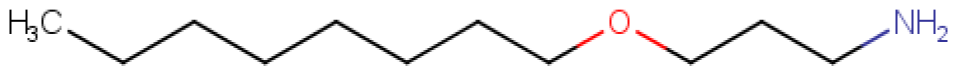

15930-66-2
Name: 3-(Octyloxy)propan-1-amine
pIC50: 4.36
Rank: 542
Classes: TSCA

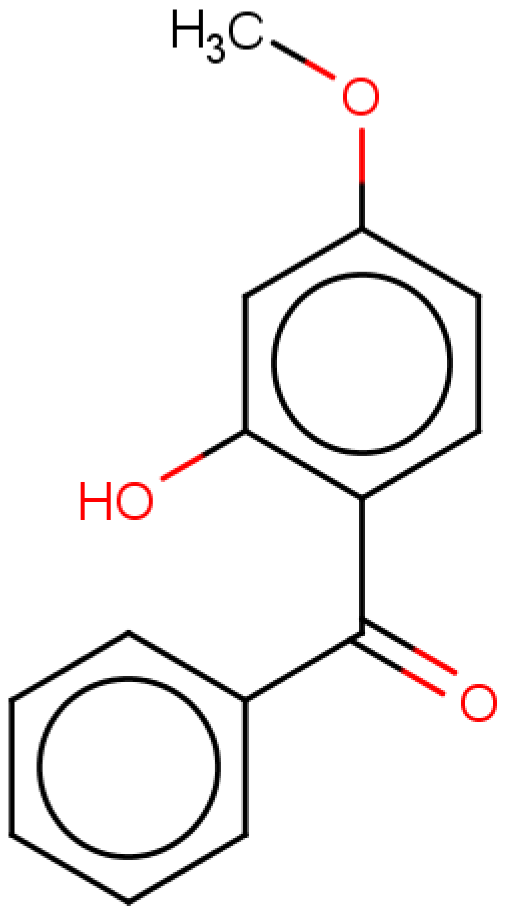

131-57-7
Name: 2-Hydroxy-4-methoxybenzophenone
pIC50: 4.36
Rank: 543
Classes: UV absorber--Drug--TSCA

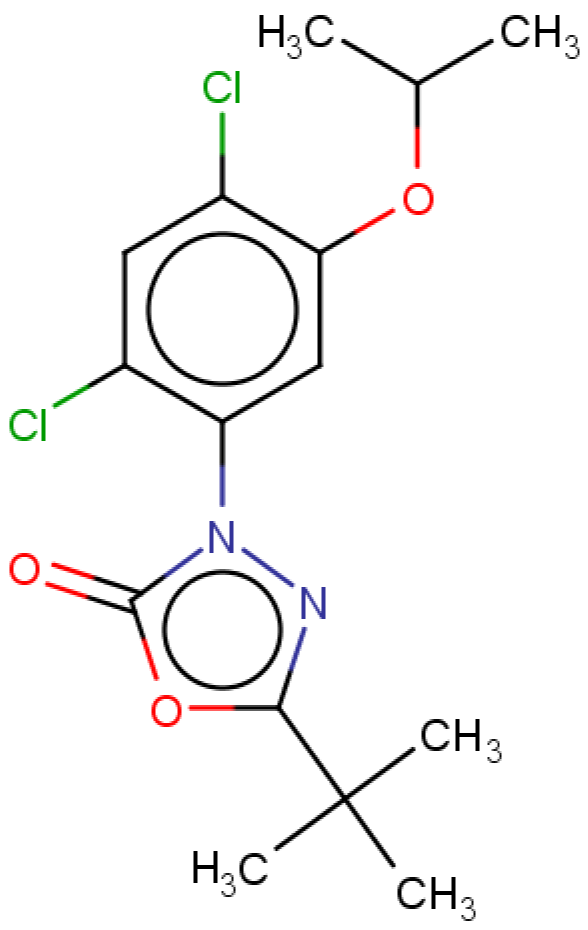

19666-30-9
Name: Oxadiazon
pIC50: 4.35
Rank: 544
Classes: Pesticide

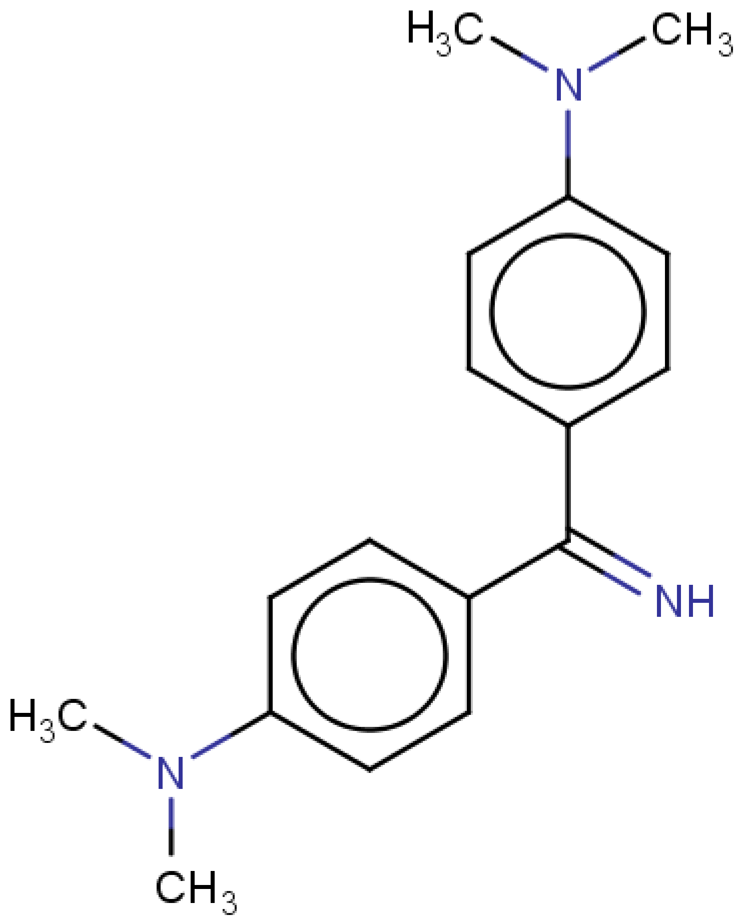

2465-27-2
Name: Auramine hydrochloride
pIC50: 4.35
Rank: 545
Classes: TSCA

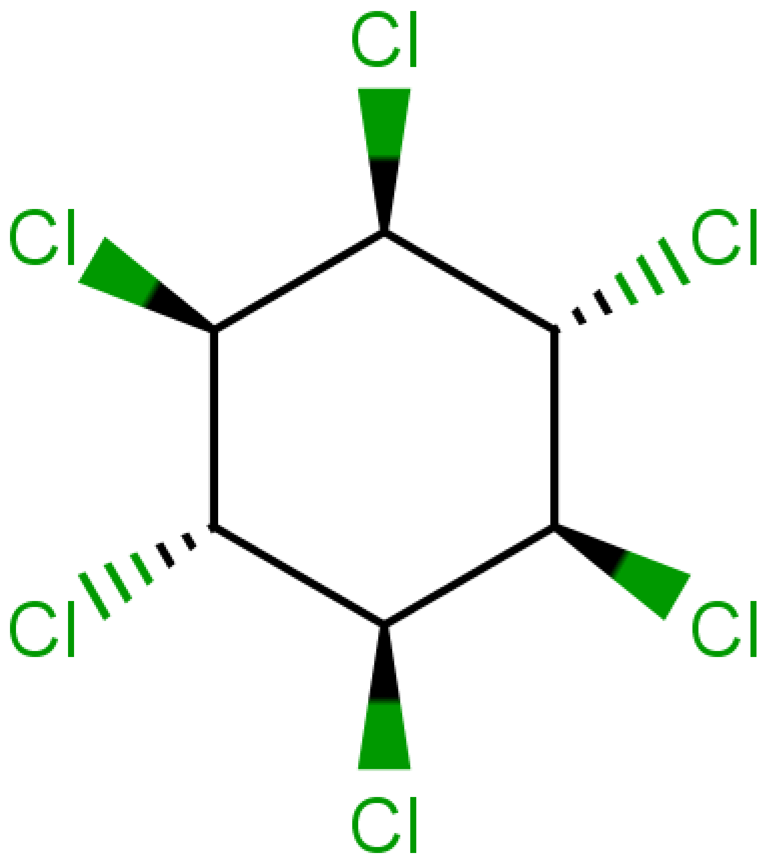

58-89-9
Name: Lindane
pIC50: 4.35
Rank: 546
Classes: flame retardant--Drug--Pesticide

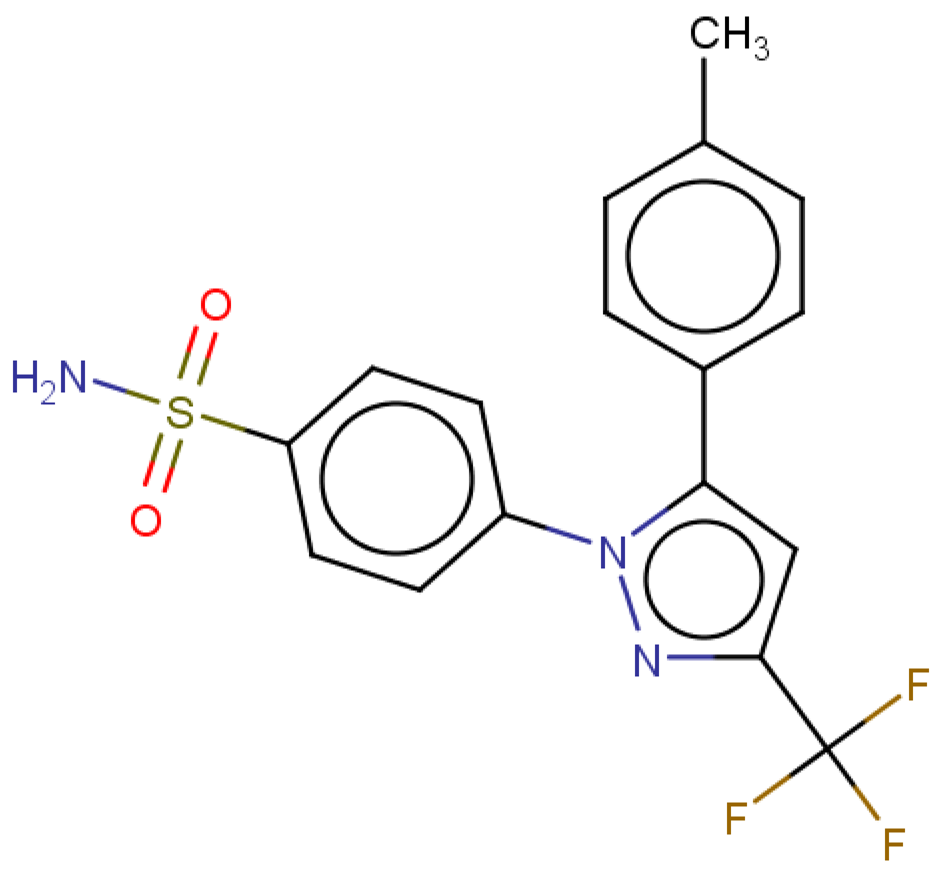

169590-42-5
Name: Celecoxib
pIC50: 4.34
Rank: 547
Classes: Drug

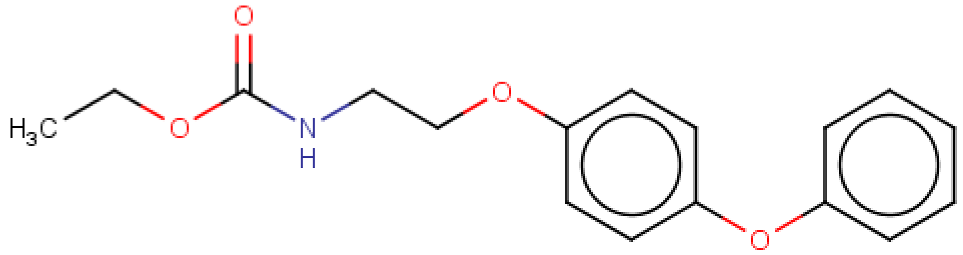

72490-01-8
Name: Fenoxycarb
pIC50: 4.34
Rank: 548
Classes: Pesticide

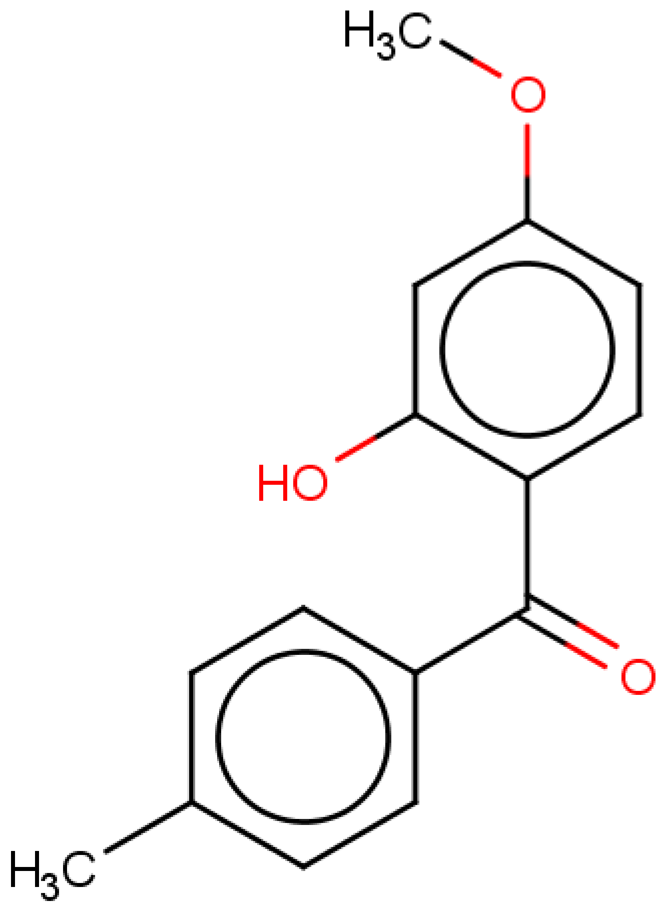

1641-17-4
Name: Mexenone
pIC50: 4.34
Rank: 549
Classes: UV absorber

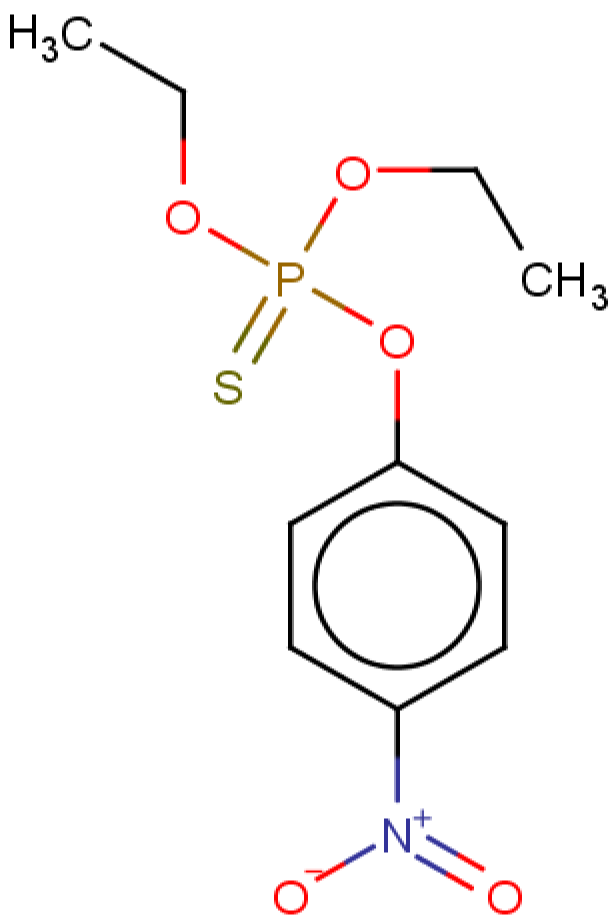

56-38-2
Name: Parathion
pIC50: 4.34
Rank: 550
Classes: No defined

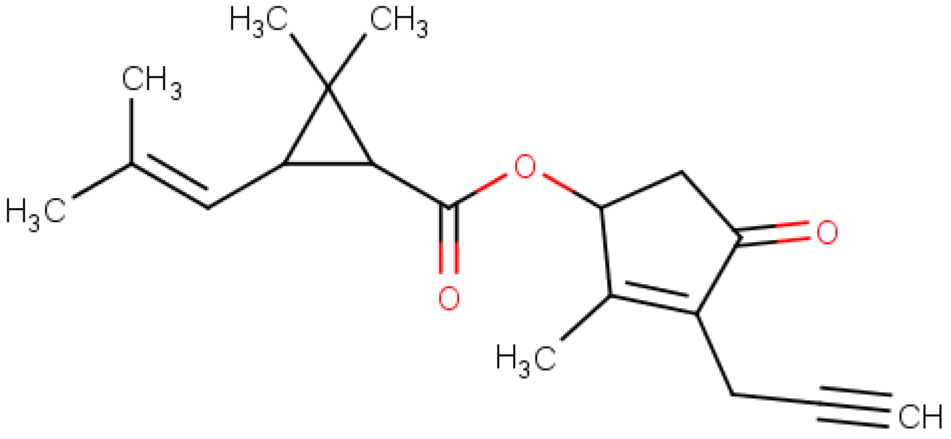

23031-36-9
Name: Prallethrin
pIC50: 4.33
Rank: 551
Classes: active ingredient--Pesticide

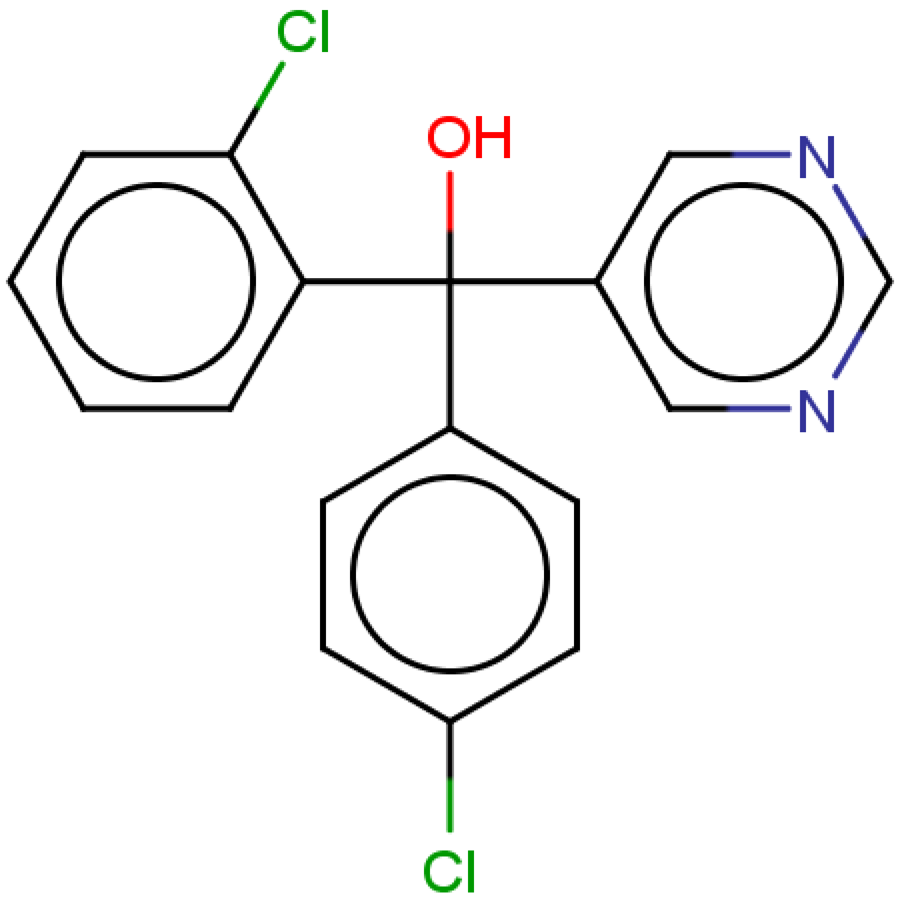

60168-88-9
Name: Fenarimol
pIC50: 4.32
Rank: 552
Classes: Pesticide

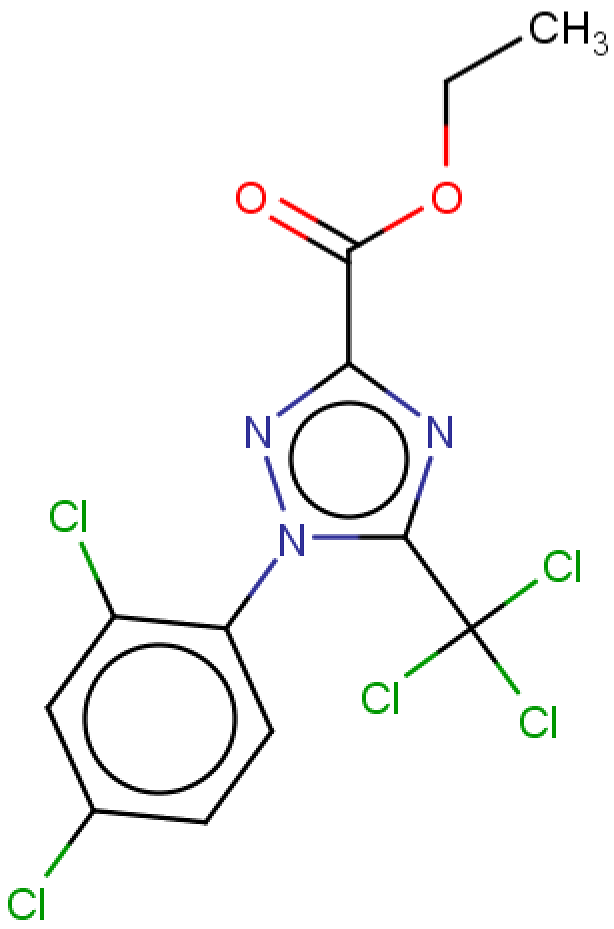

103112-35-2
Name: Fenchlorazole-ethyl
pIC50: 4.31
Rank: 553
Classes: No defined

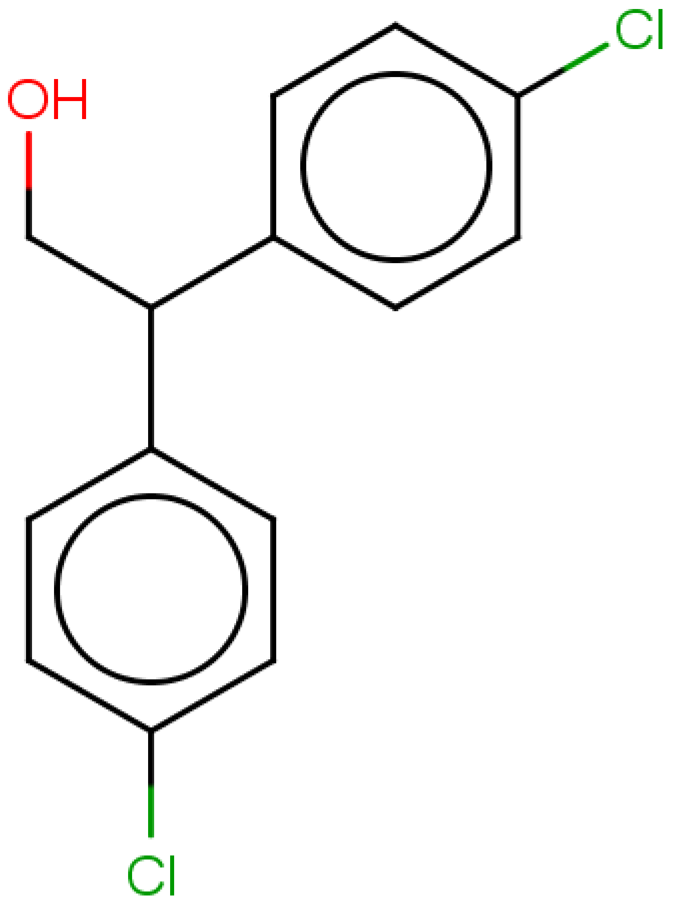

2642-82-2
Name: 2 2-Bis(4-chlorophenyl)ethanol
pIC50: 4.31
Rank: 554
Classes: No defined

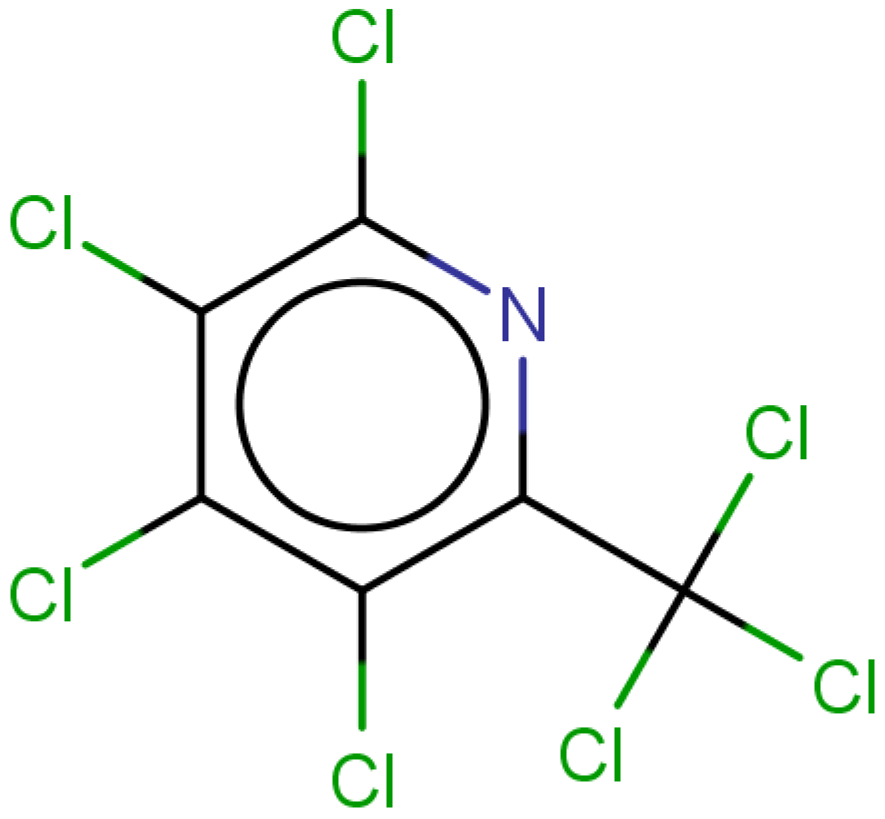

1134-04-9
Name: 2 3 4 5-Tetrachloro-6-(trichloromethyl)p
pIC50: 4.31
Rank: 555
Classes: TSCA

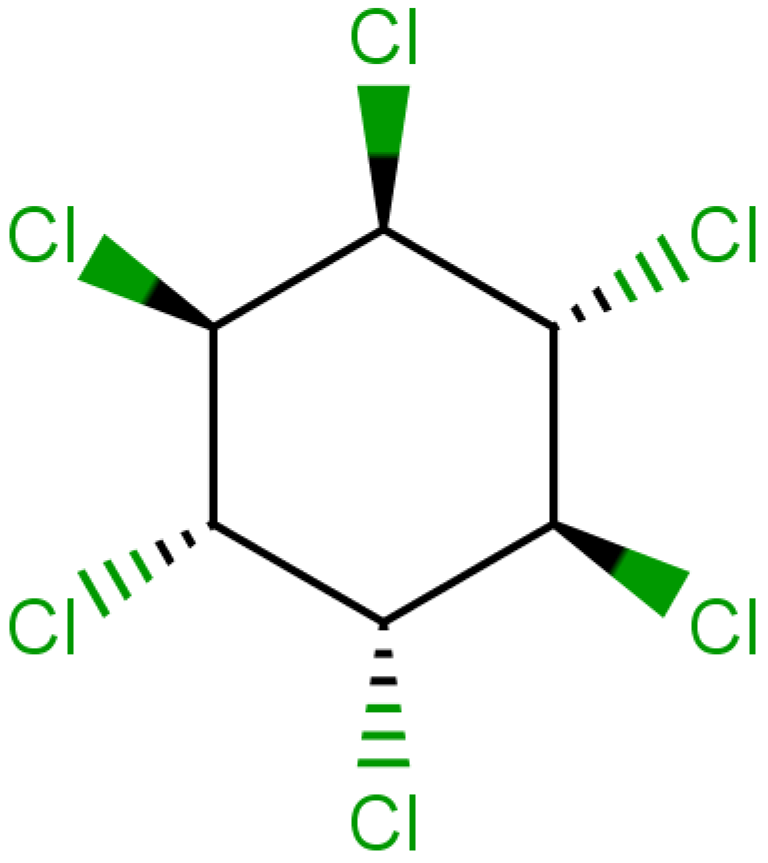

319-84-6
Name: alpha-1 2 3 4 5 6-Hexachlorocyclohexane
pIC50: 4.31
Rank: 556
Classes: No defined

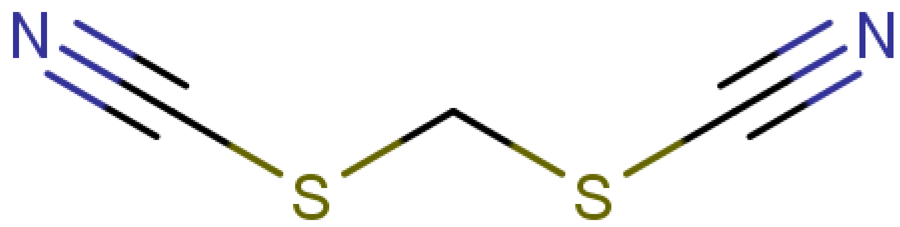

6317-18-6
Name: Methylene bis(thiocyanate)
pIC50: 4.3
Rank: 557
Classes: antimicrobial

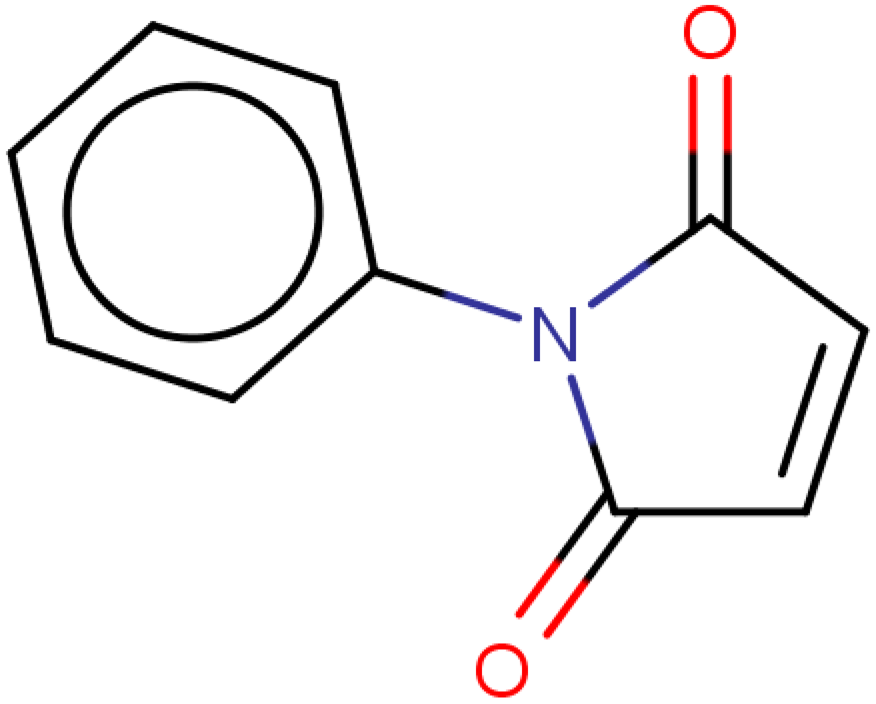

941-69-5
Name: 1-Phenyl-1H-pyrrole-2 5-dione
pIC50: 4.29
Rank: 558
Classes: TSCA
