# Supplementary figures and images for: High-Throughput Chemical Screening and Structure-Based Models to Predict hERG Inhibition
Source: Biology (Basel). 2022 Jan 28;11(2):209. doi: 10.3390/biology11020209 (PMC8869358; doi:10.3390/biology11020209)

101477-54-7

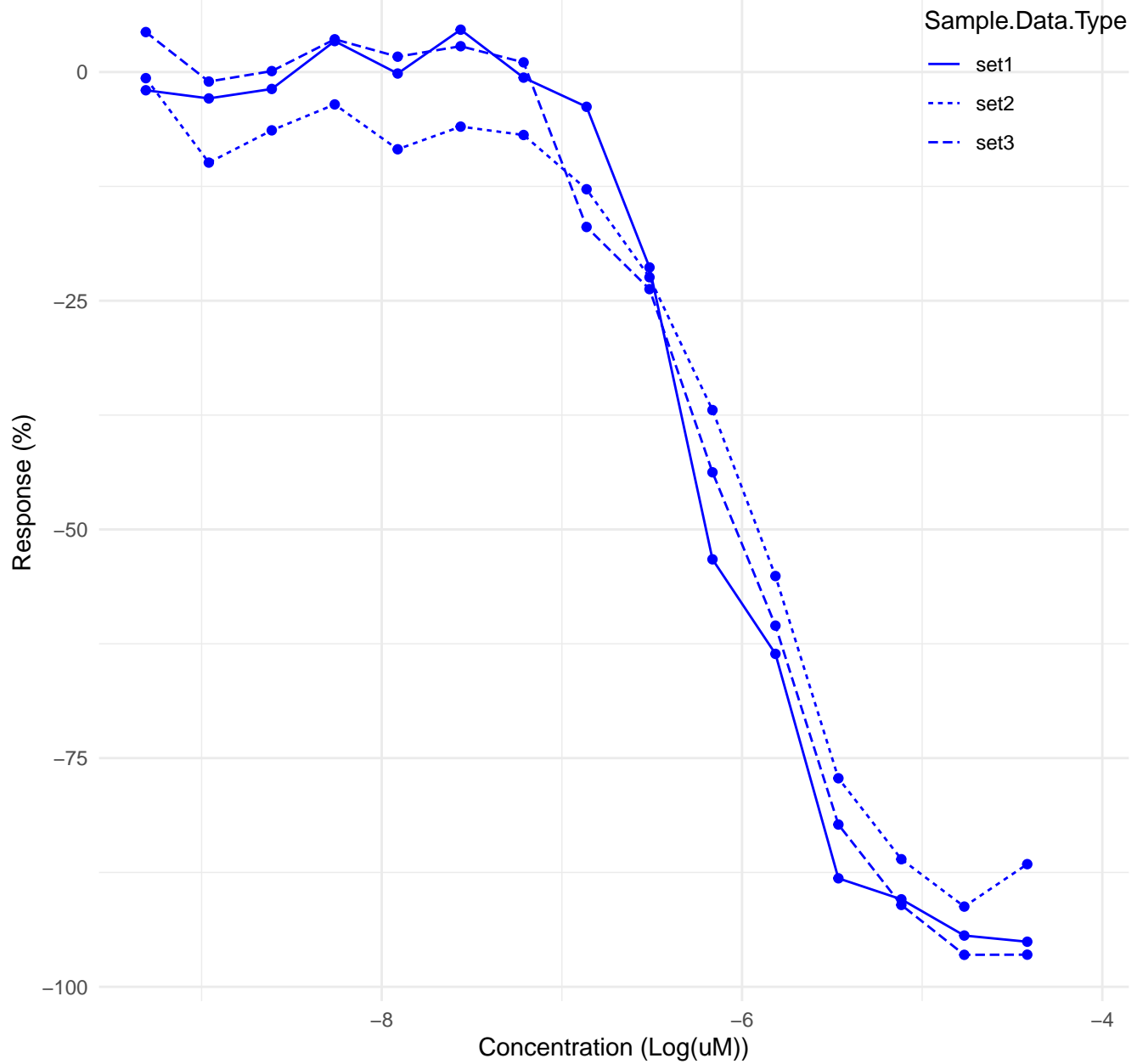

10457-90-6

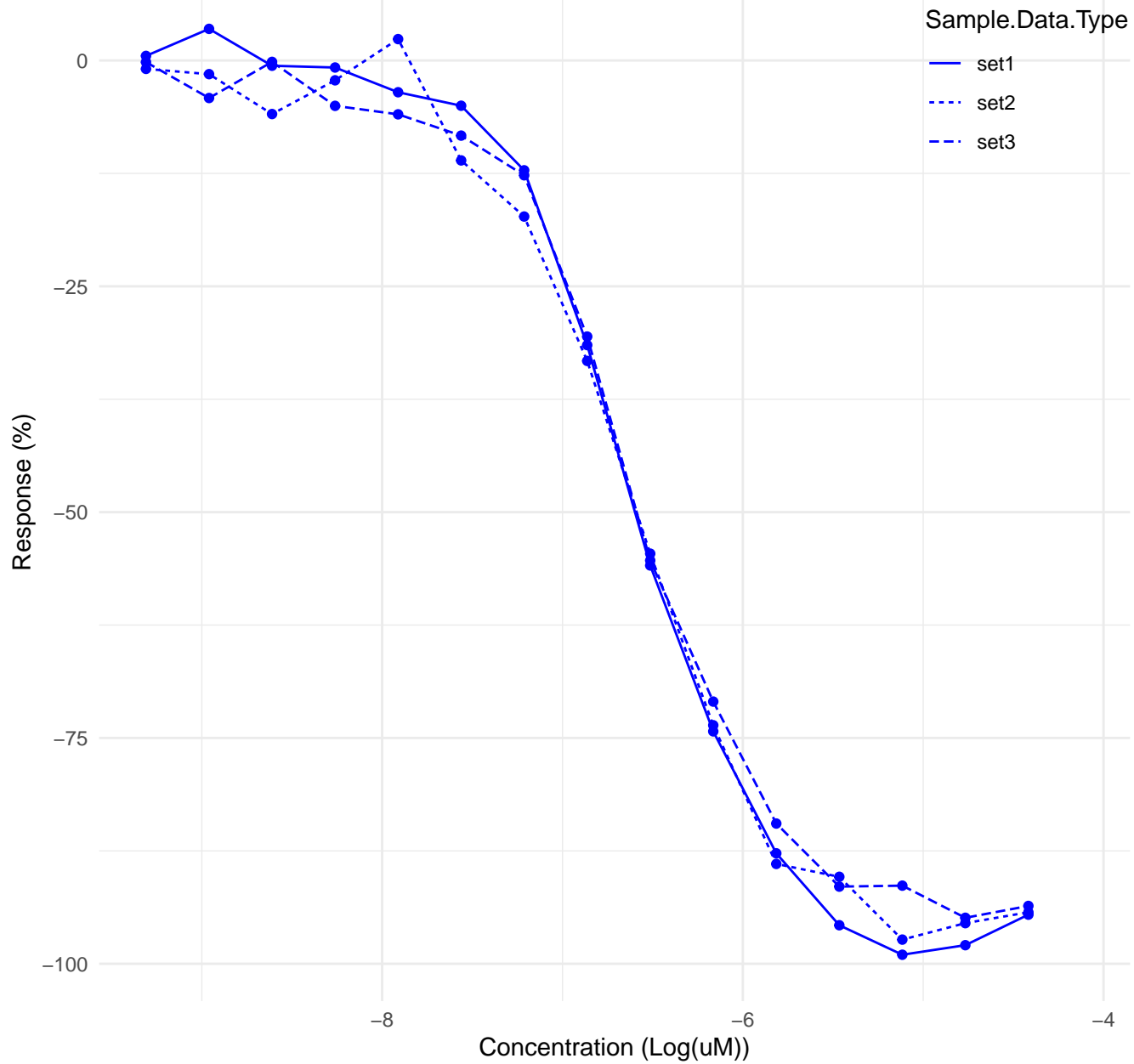

106516-24-9

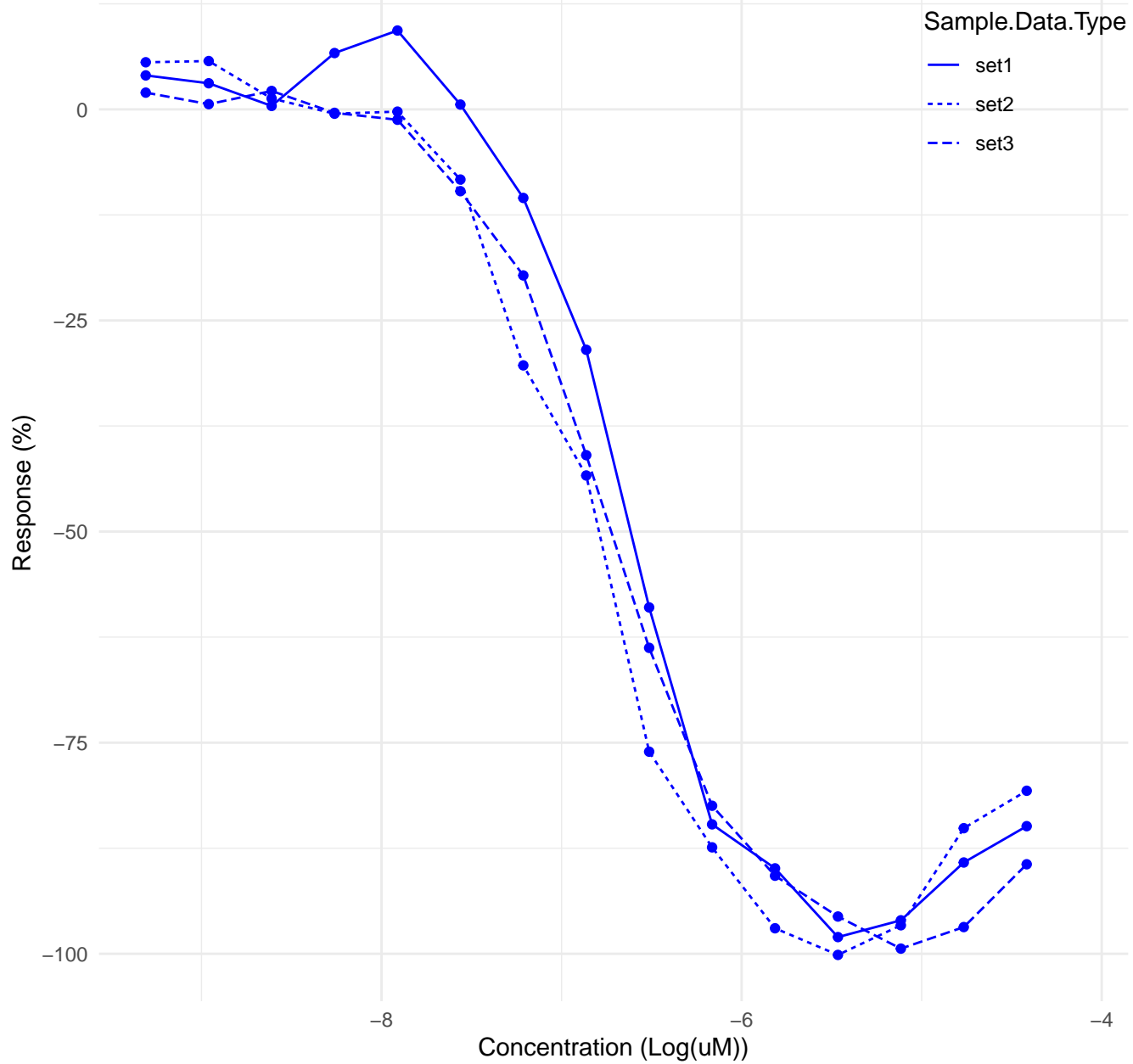

1135280-78-2

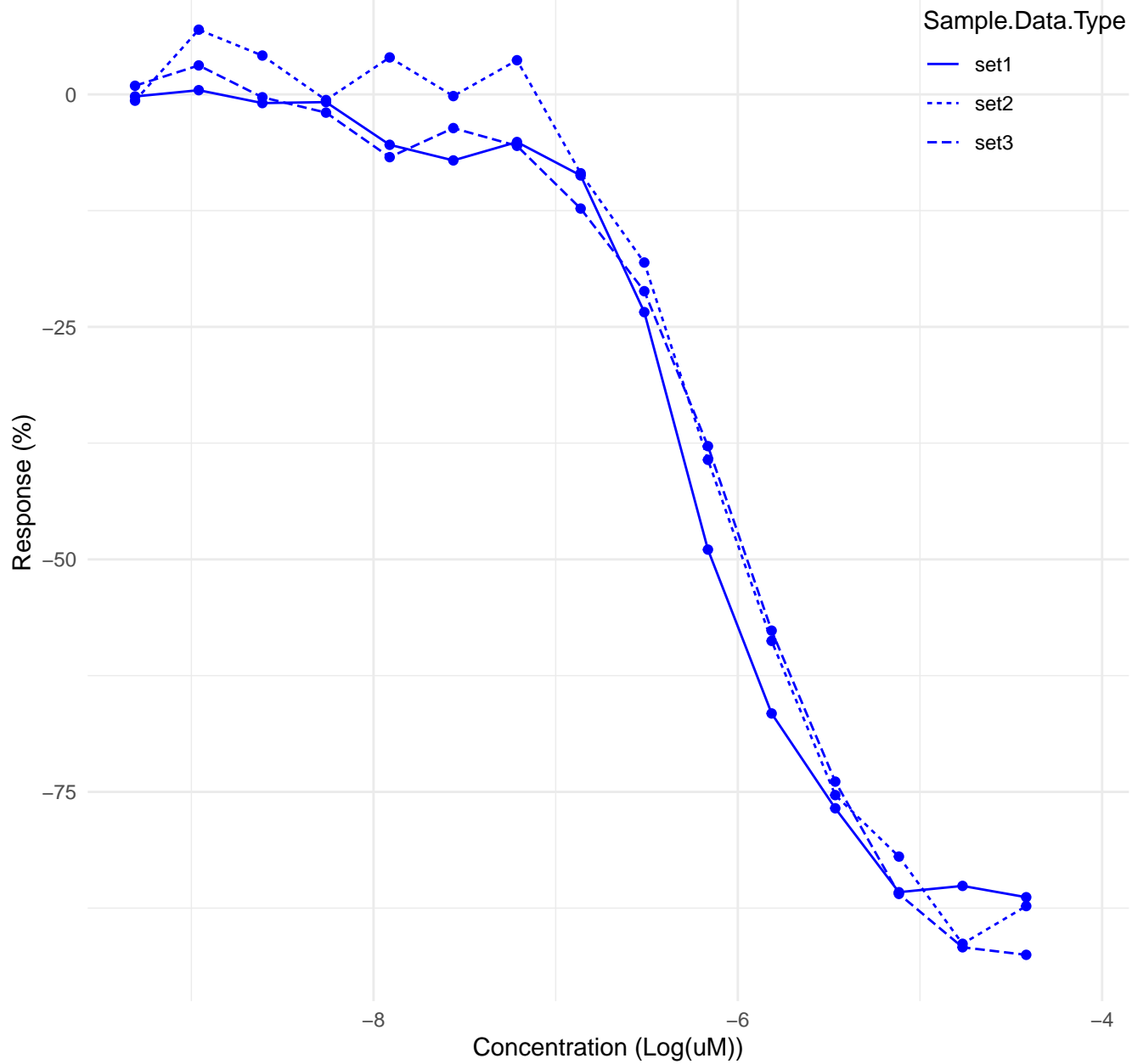

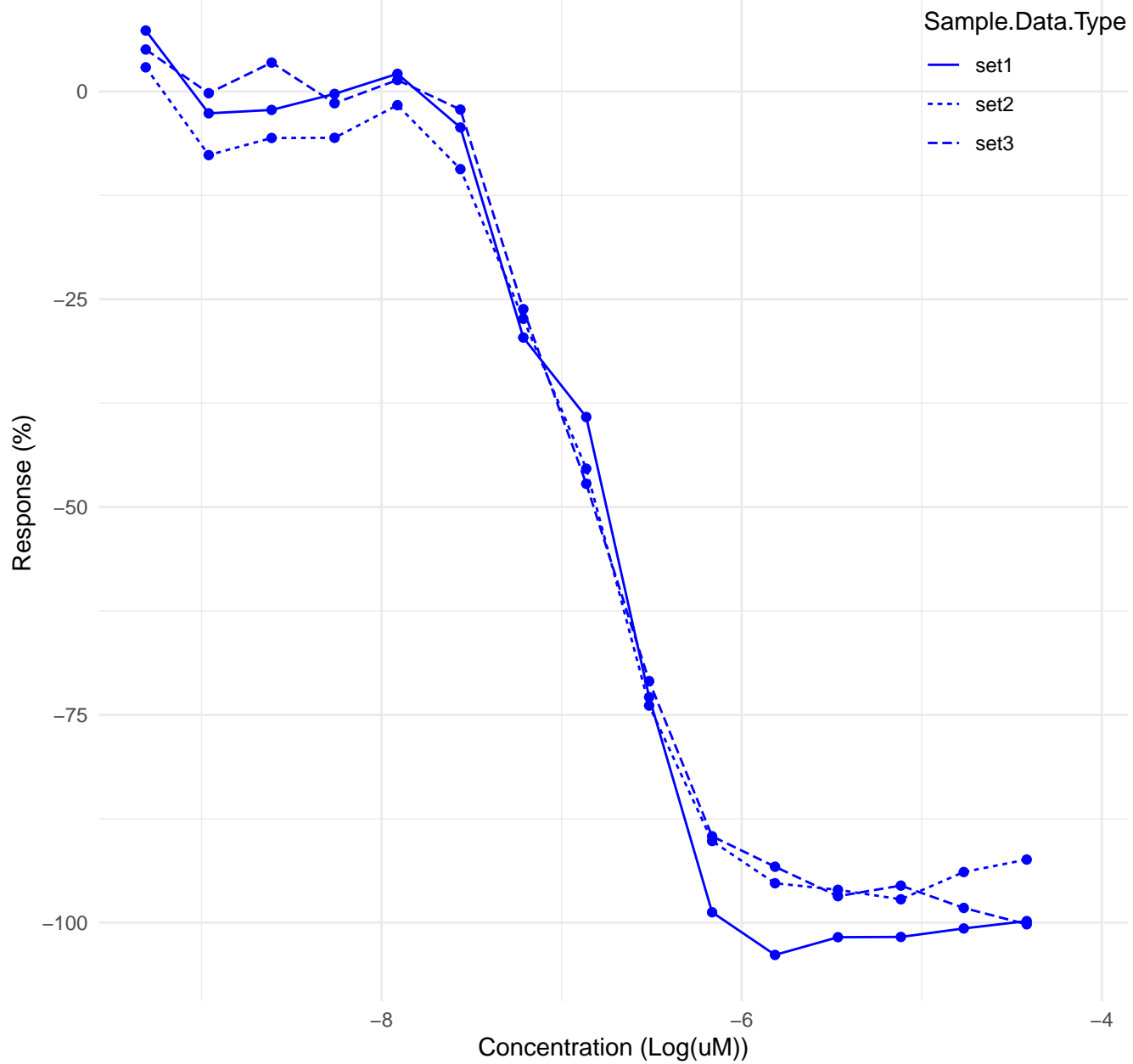

119431-25-3

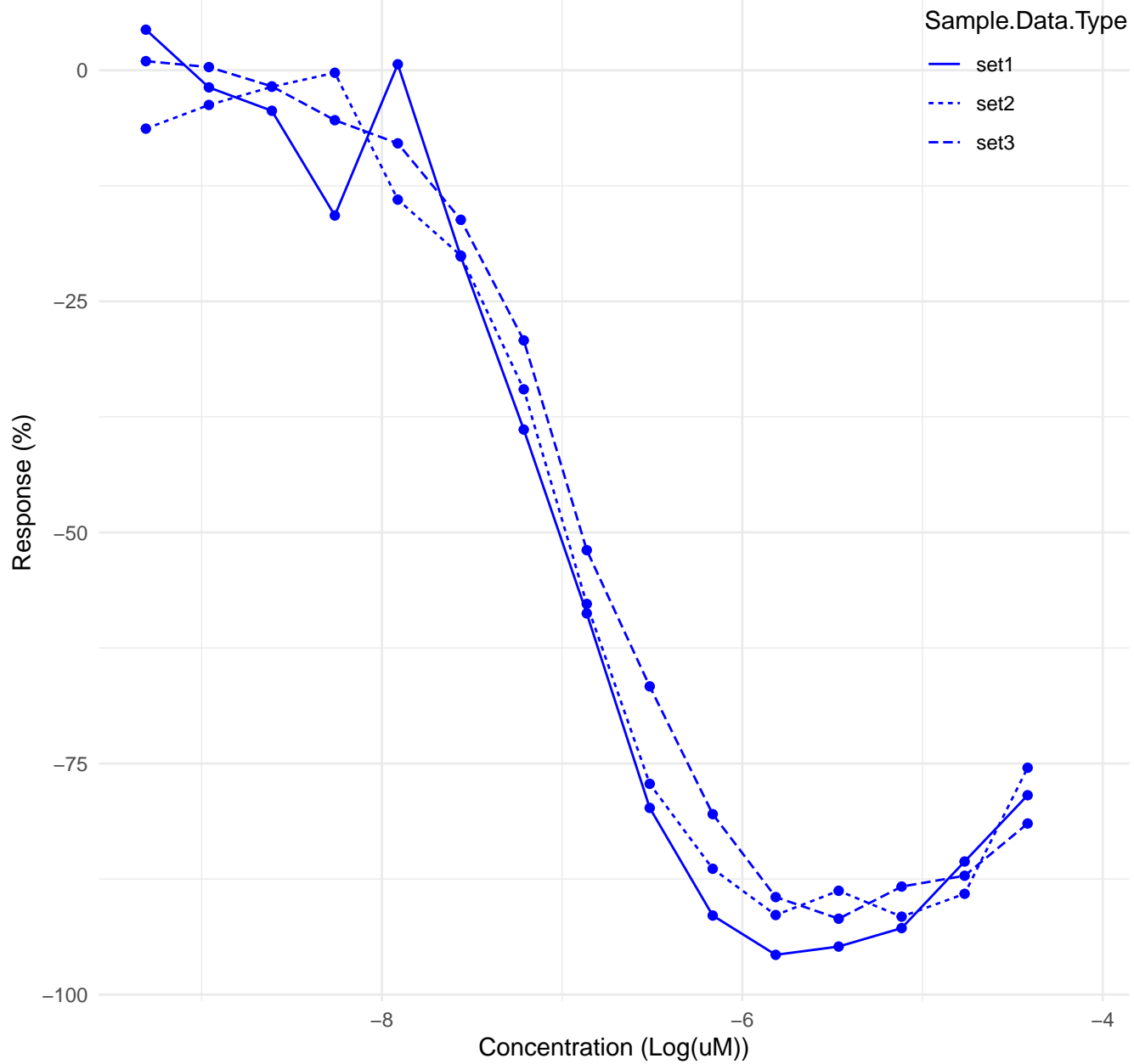

121-54-0

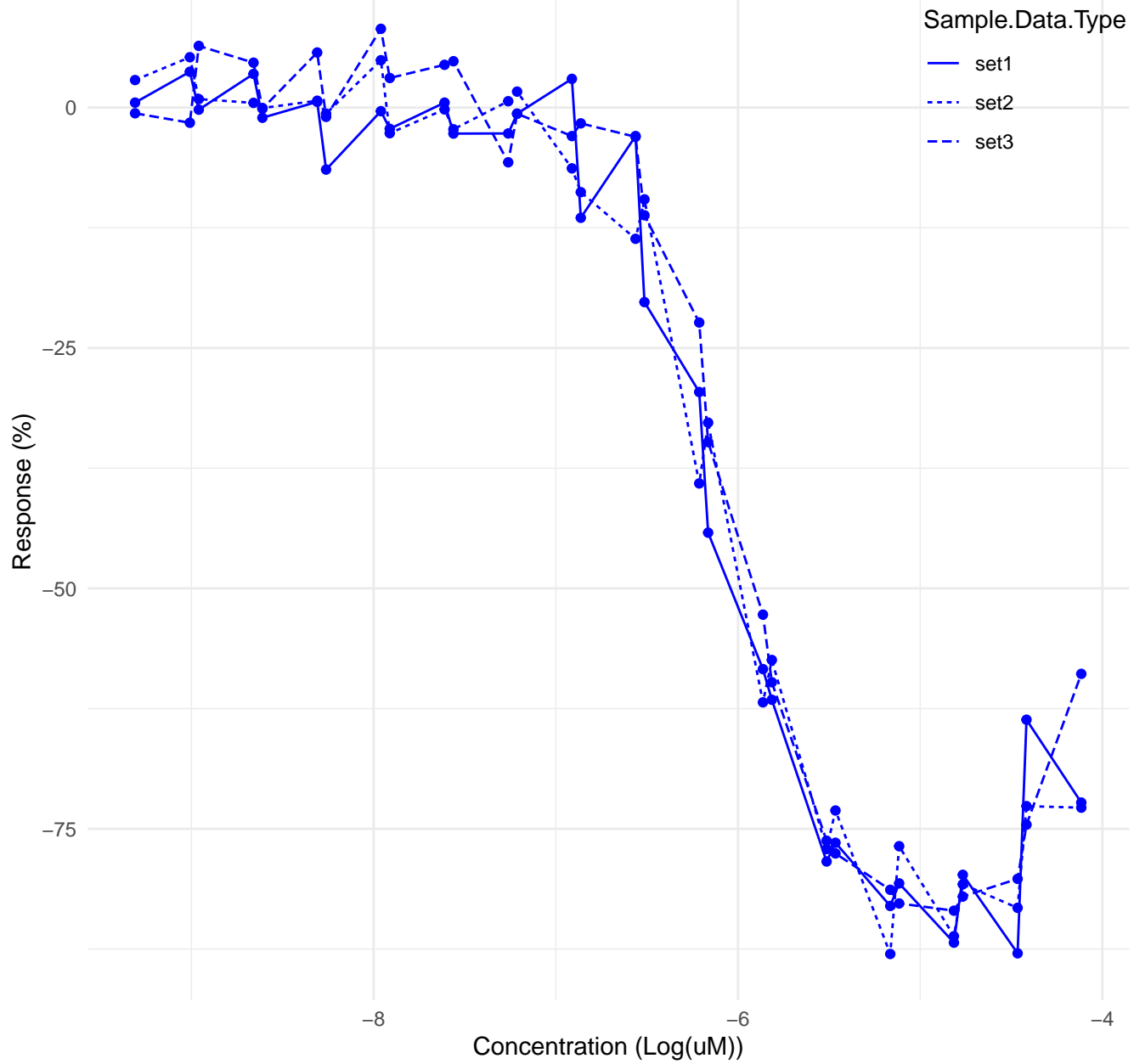

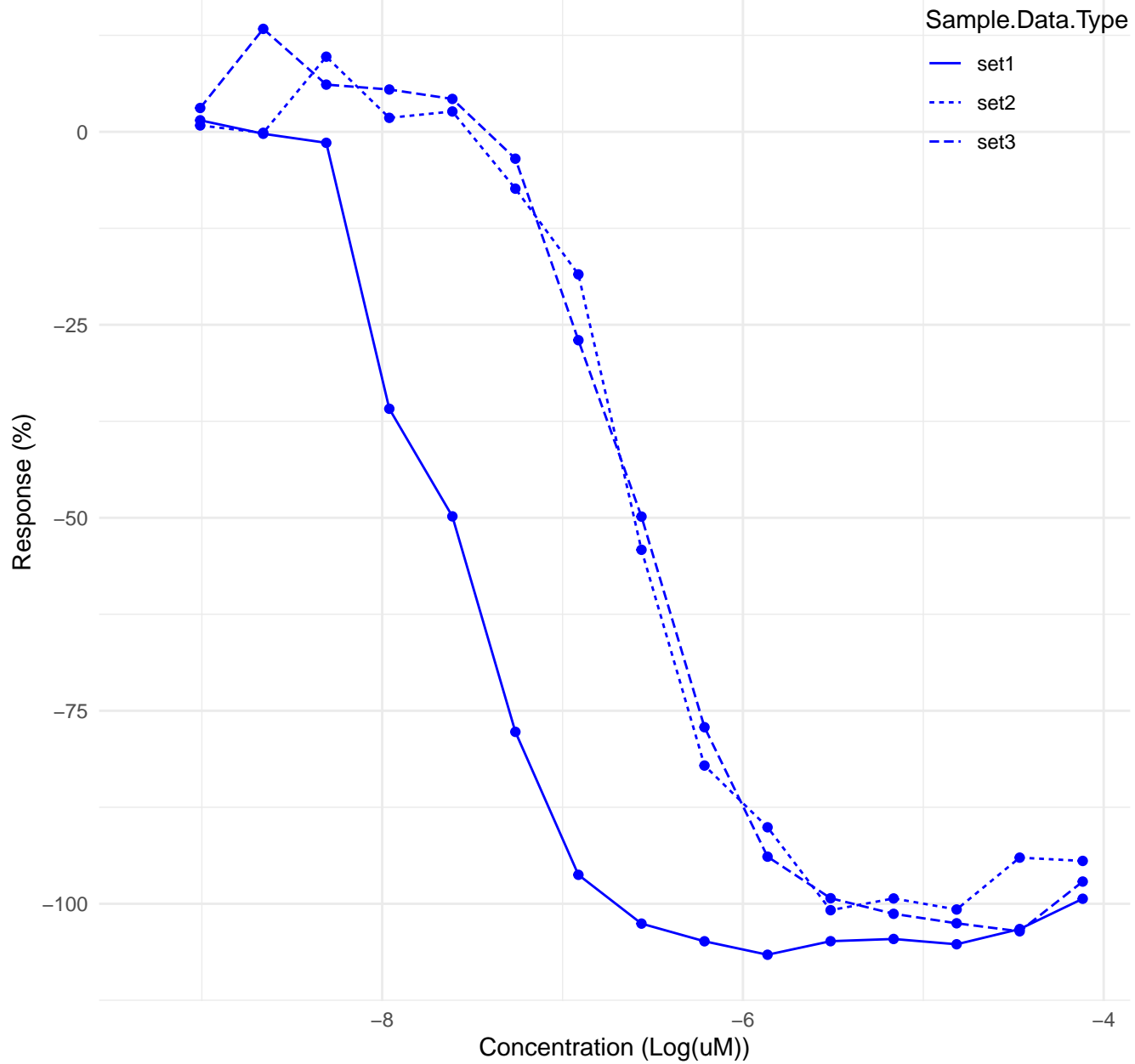

124937-51-5

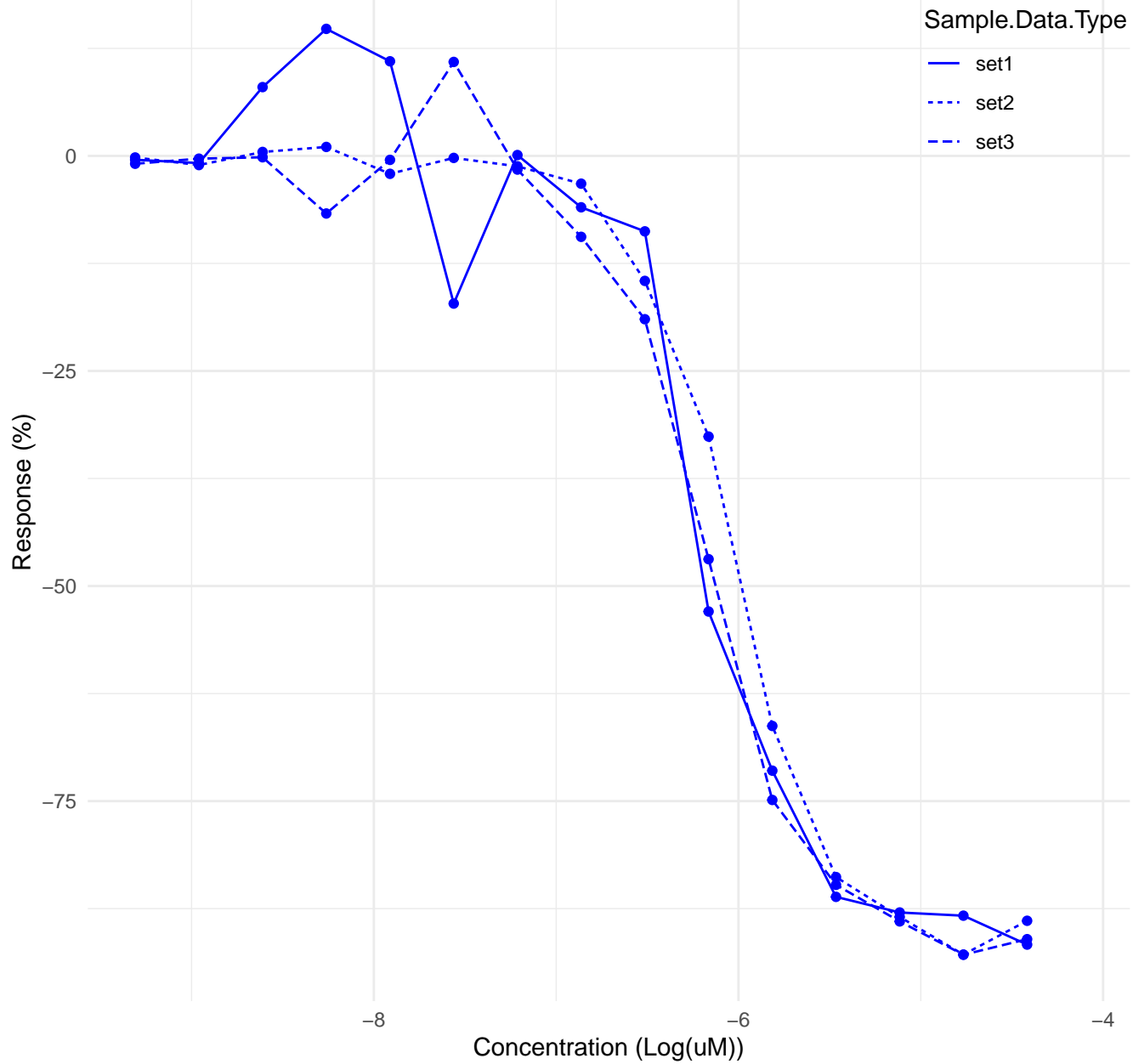

127308-82-1

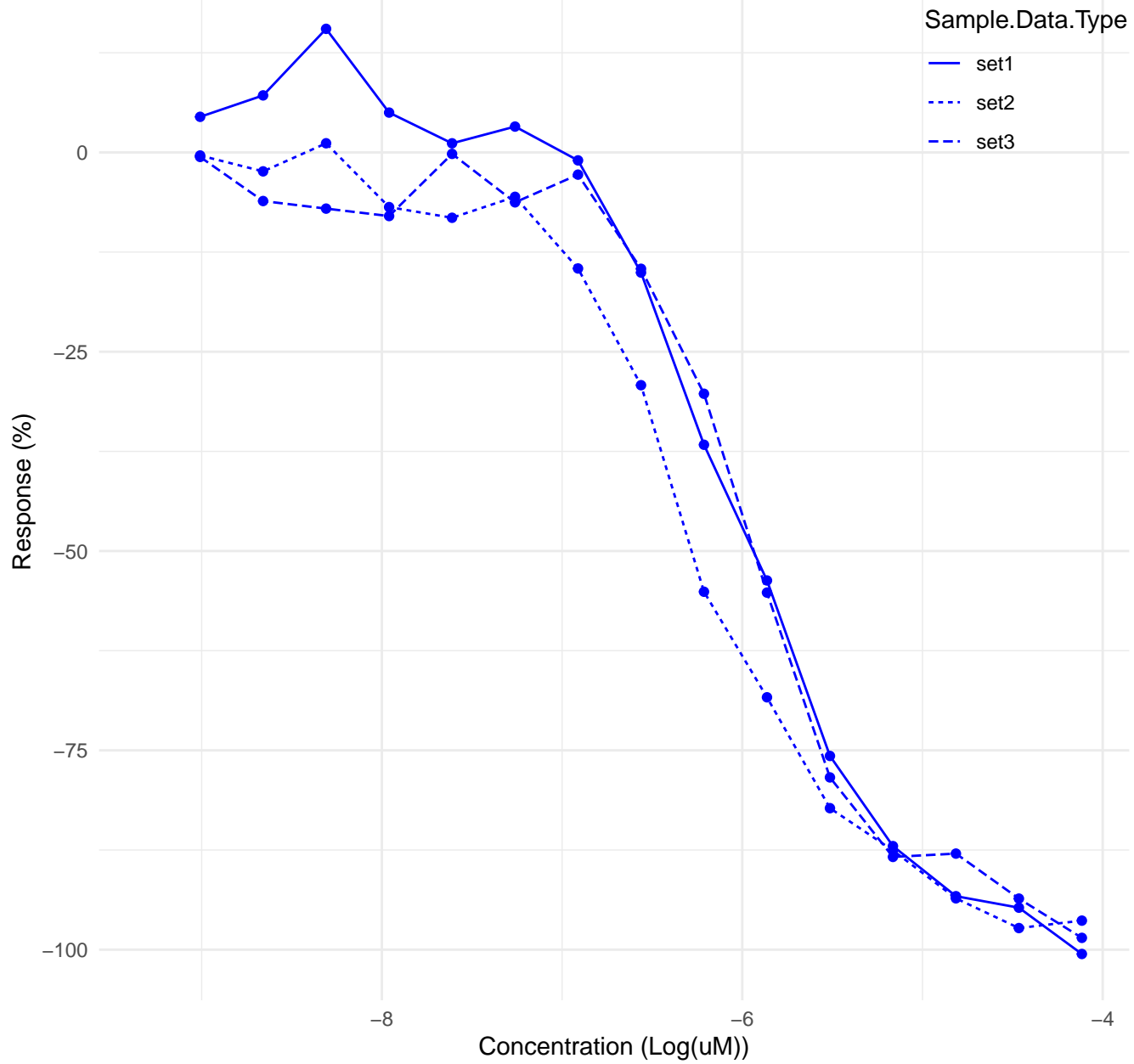

127308-98-9

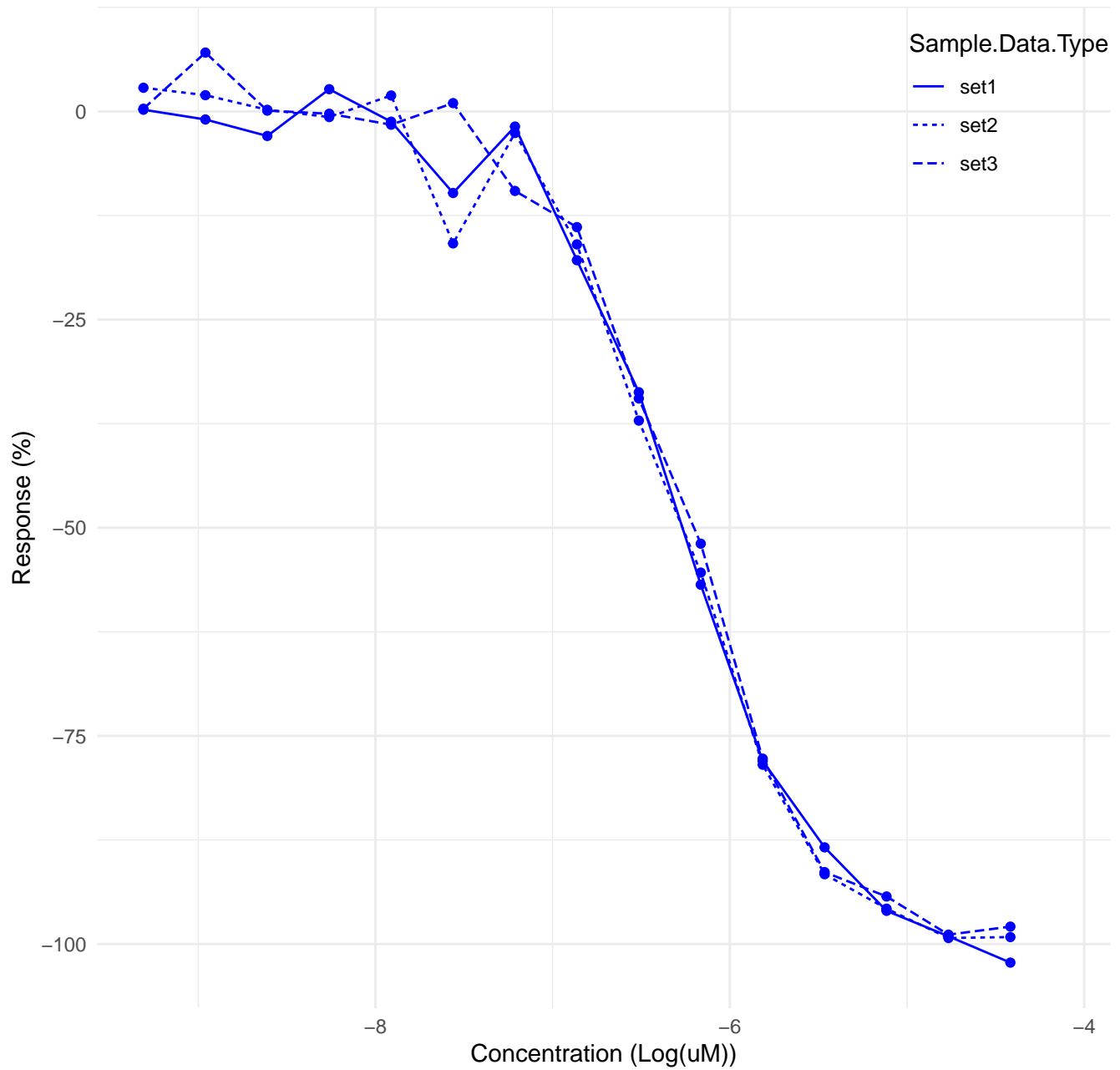

141626-36-0

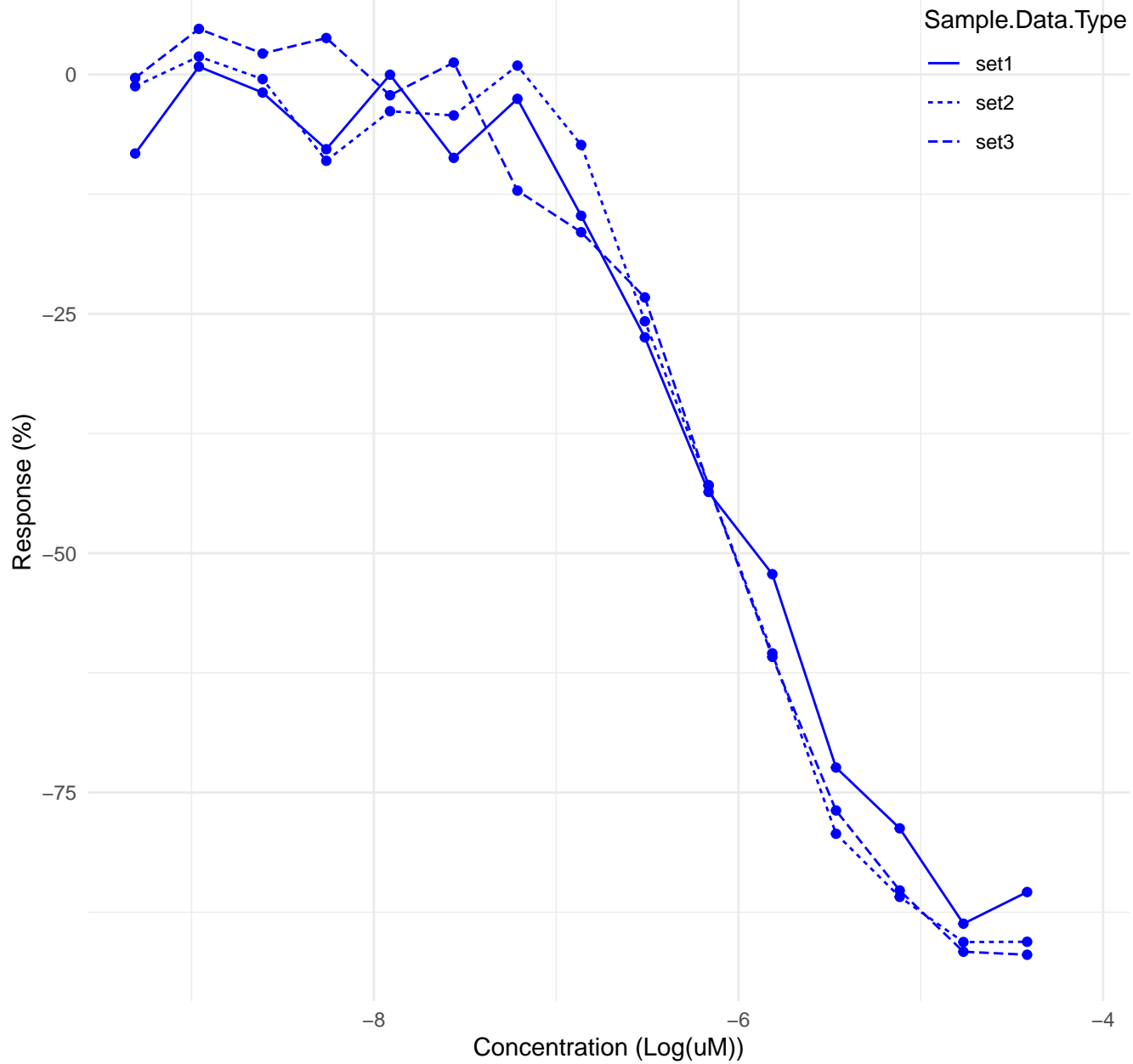

14866-33-2

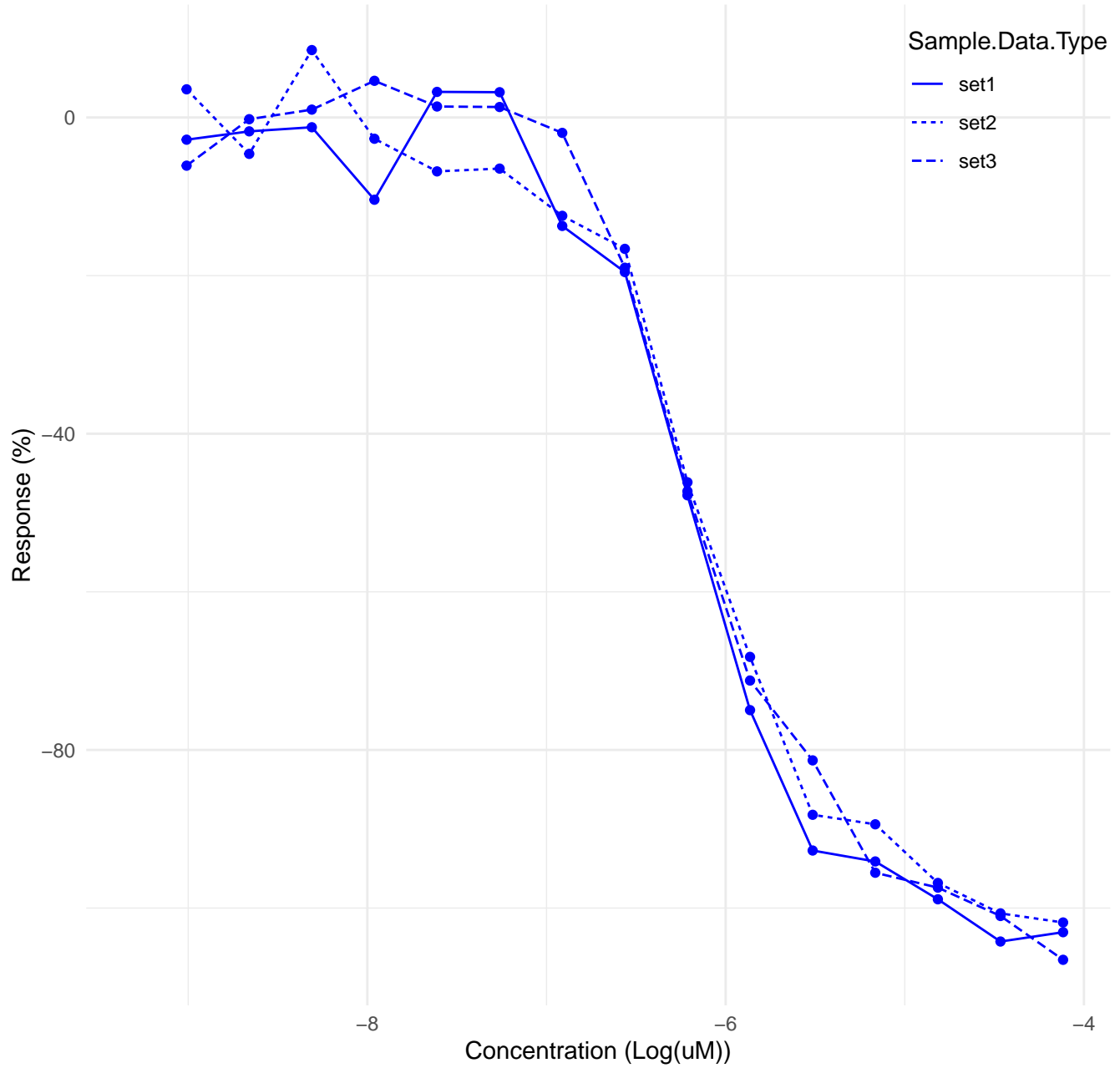

14976-57-9

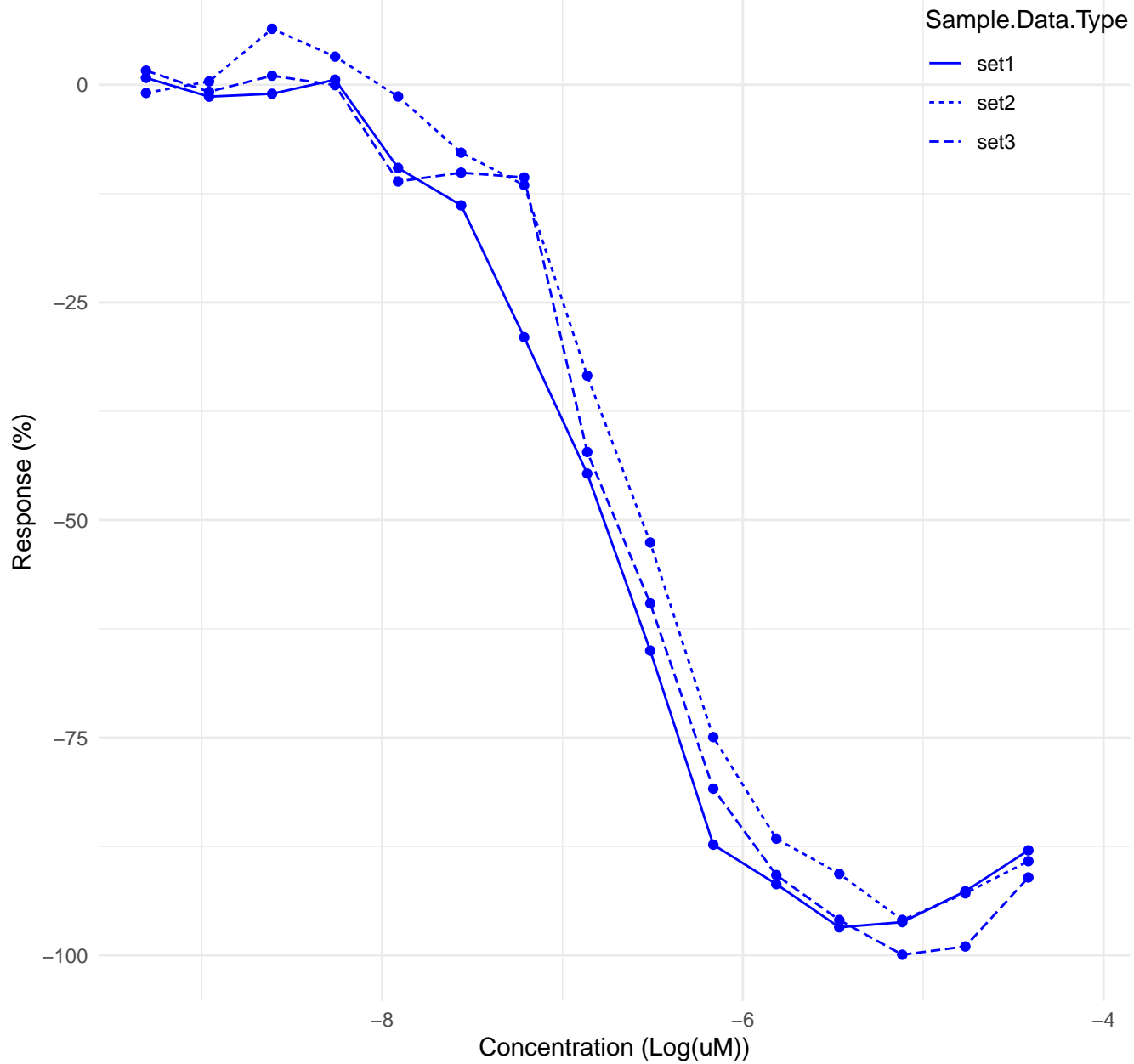

14984-68-0

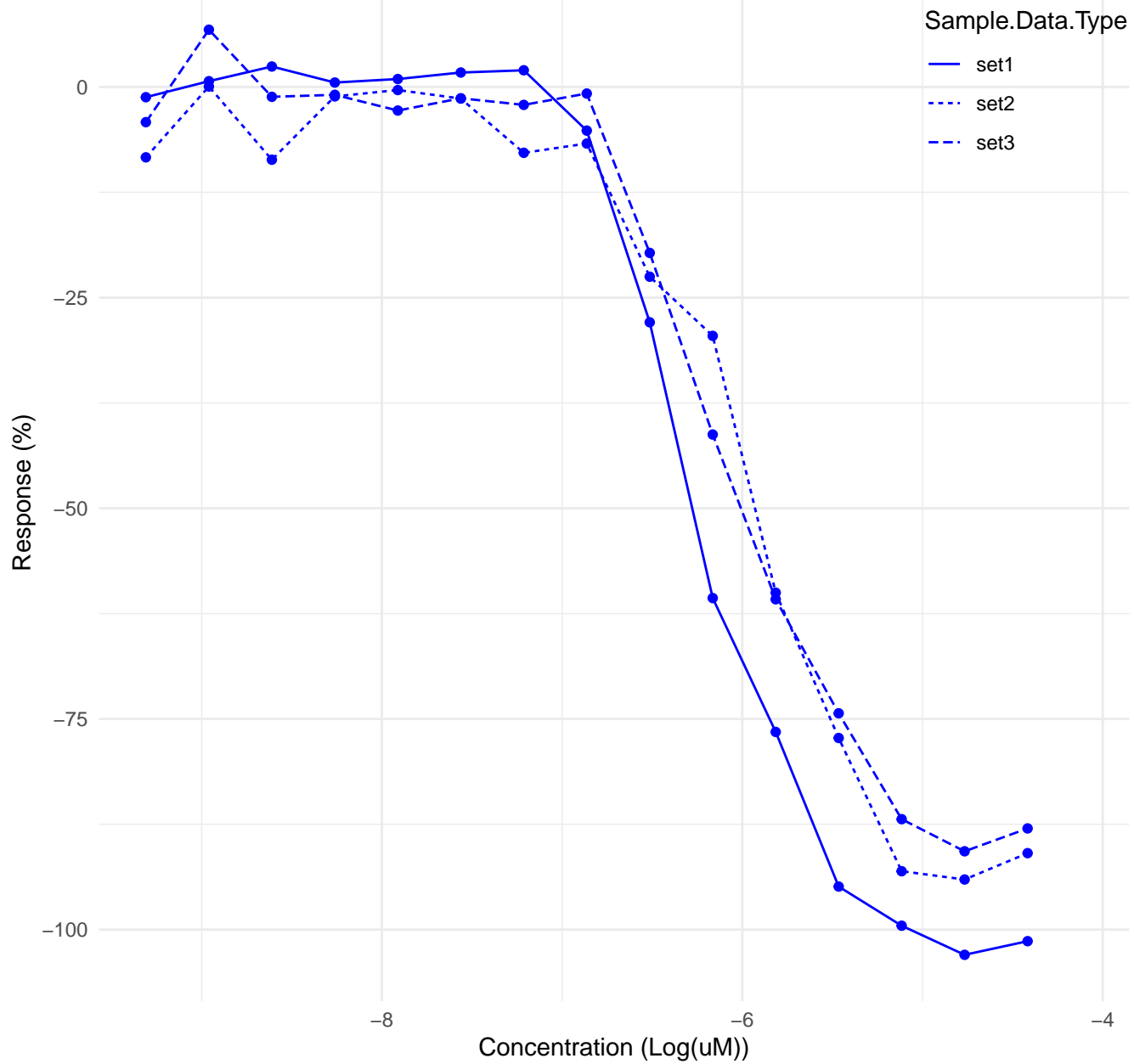

1841-19-6

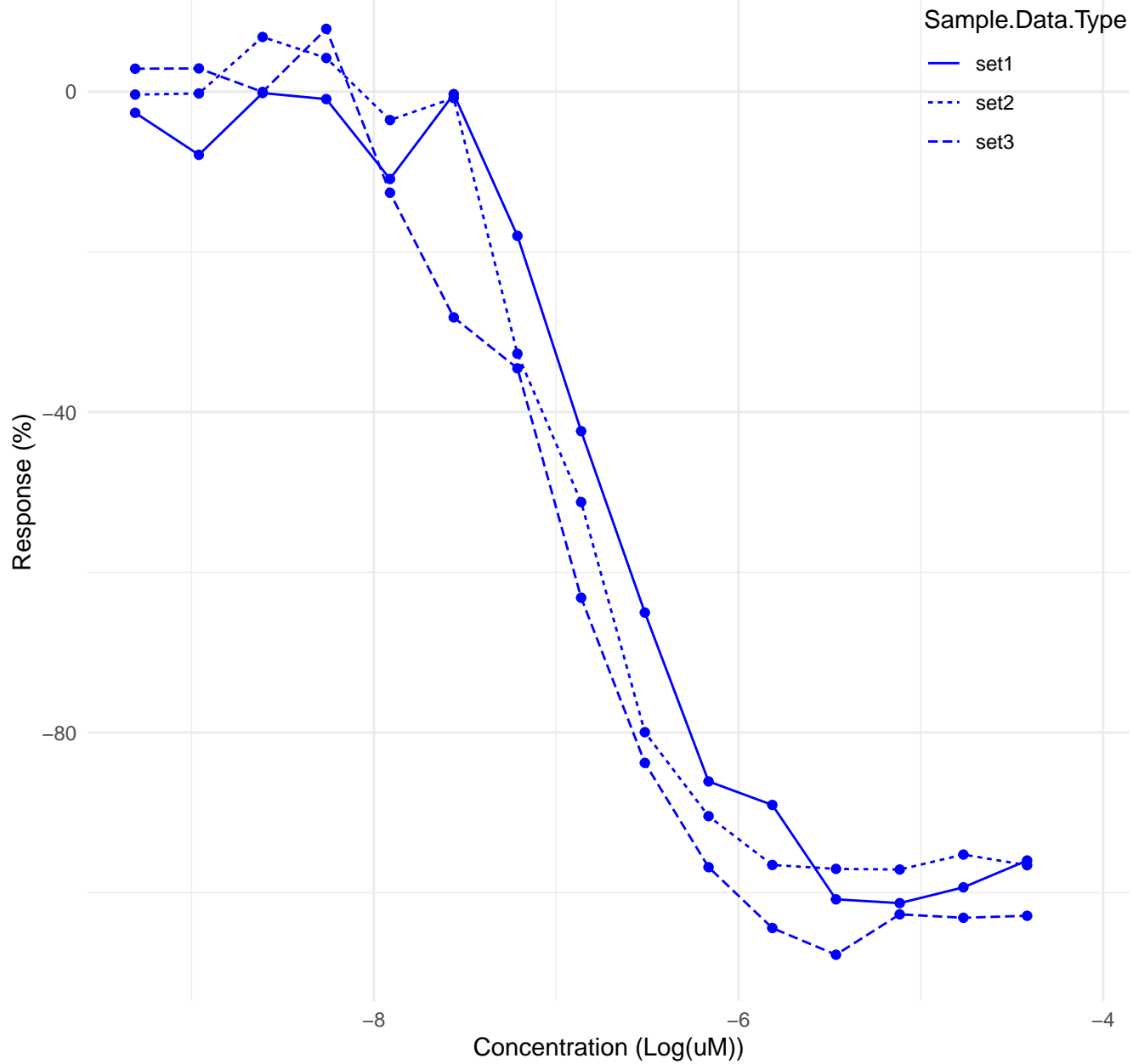

1951-25-3

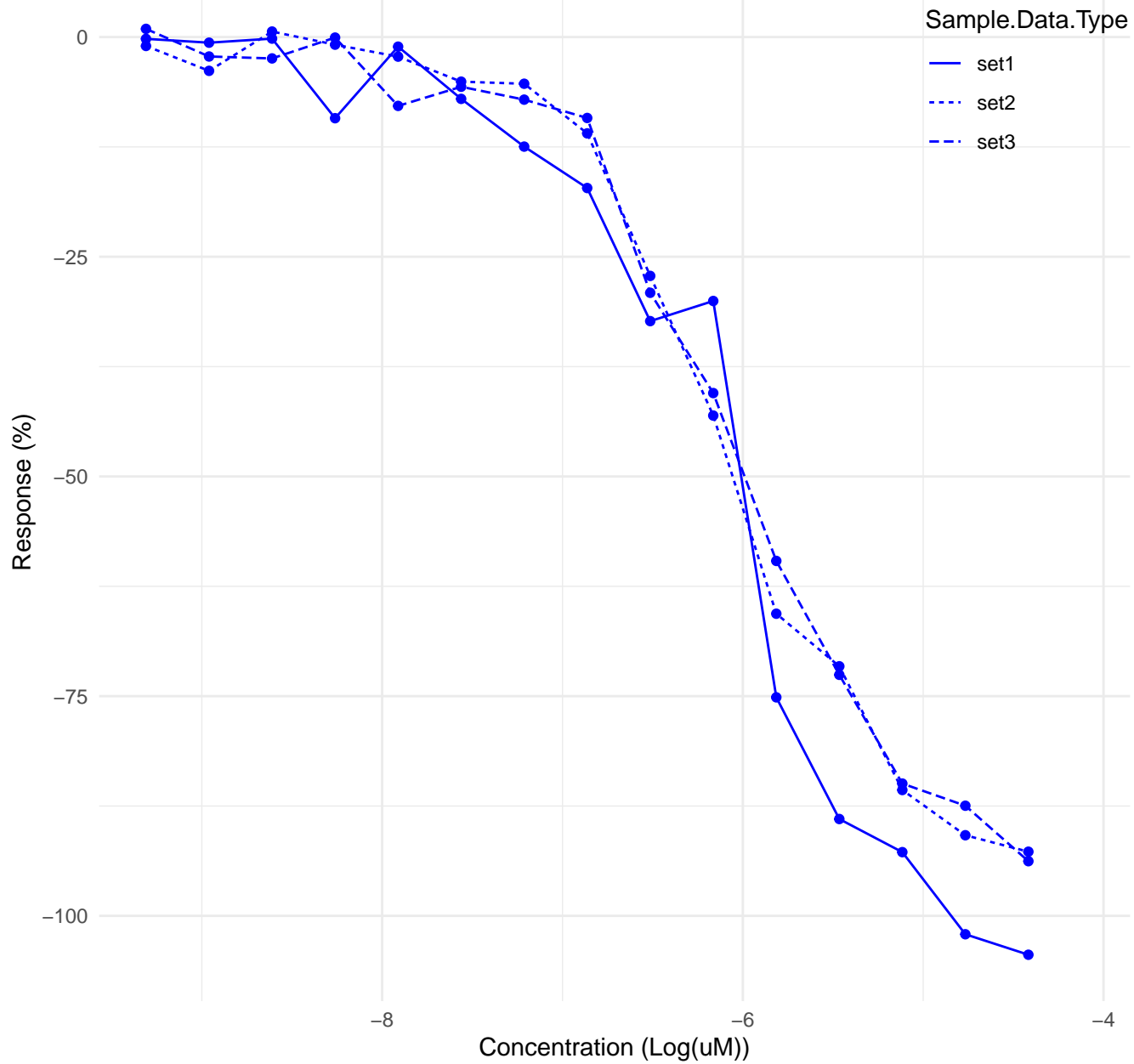

19774-82-4

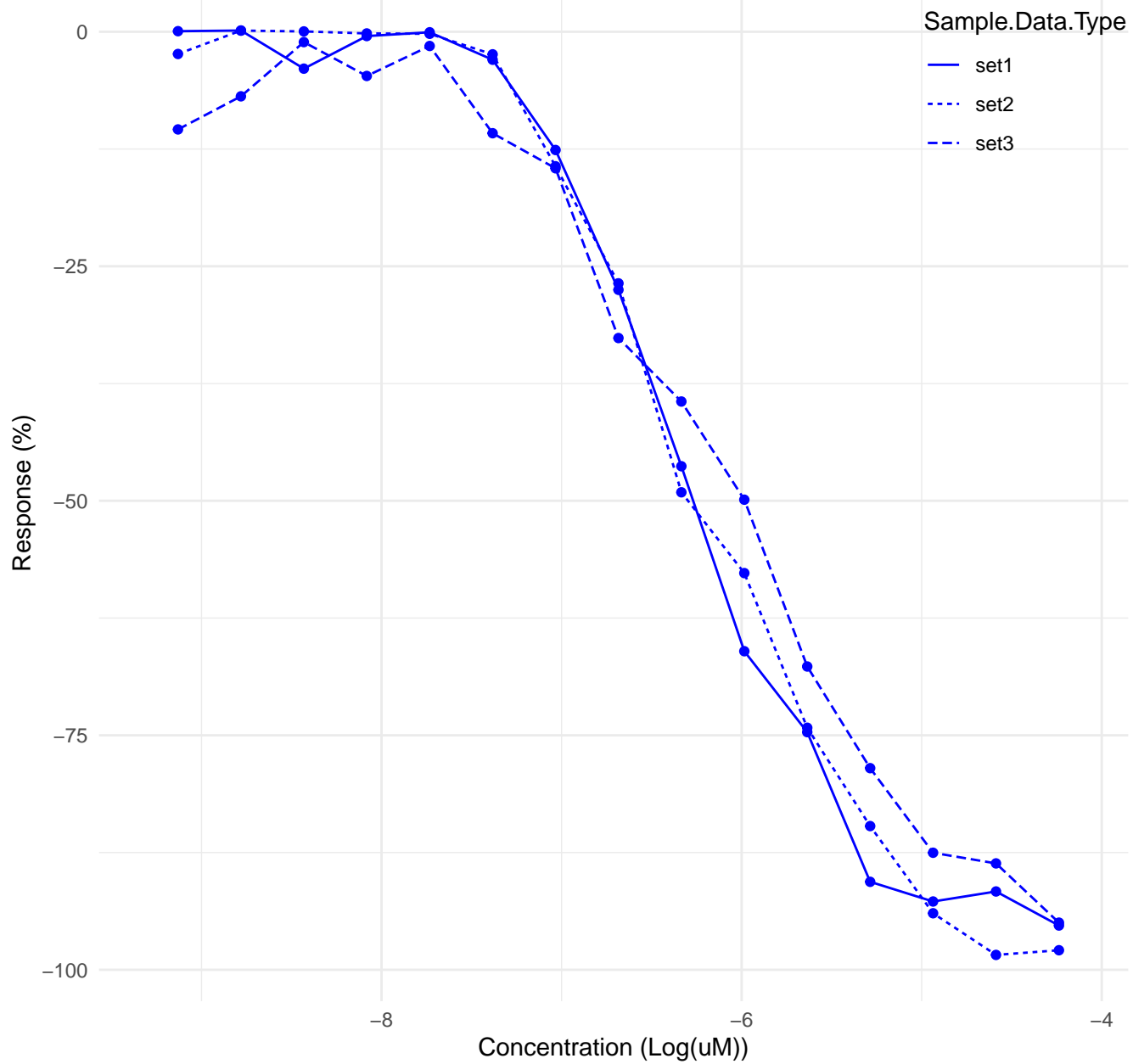

2062-77-3

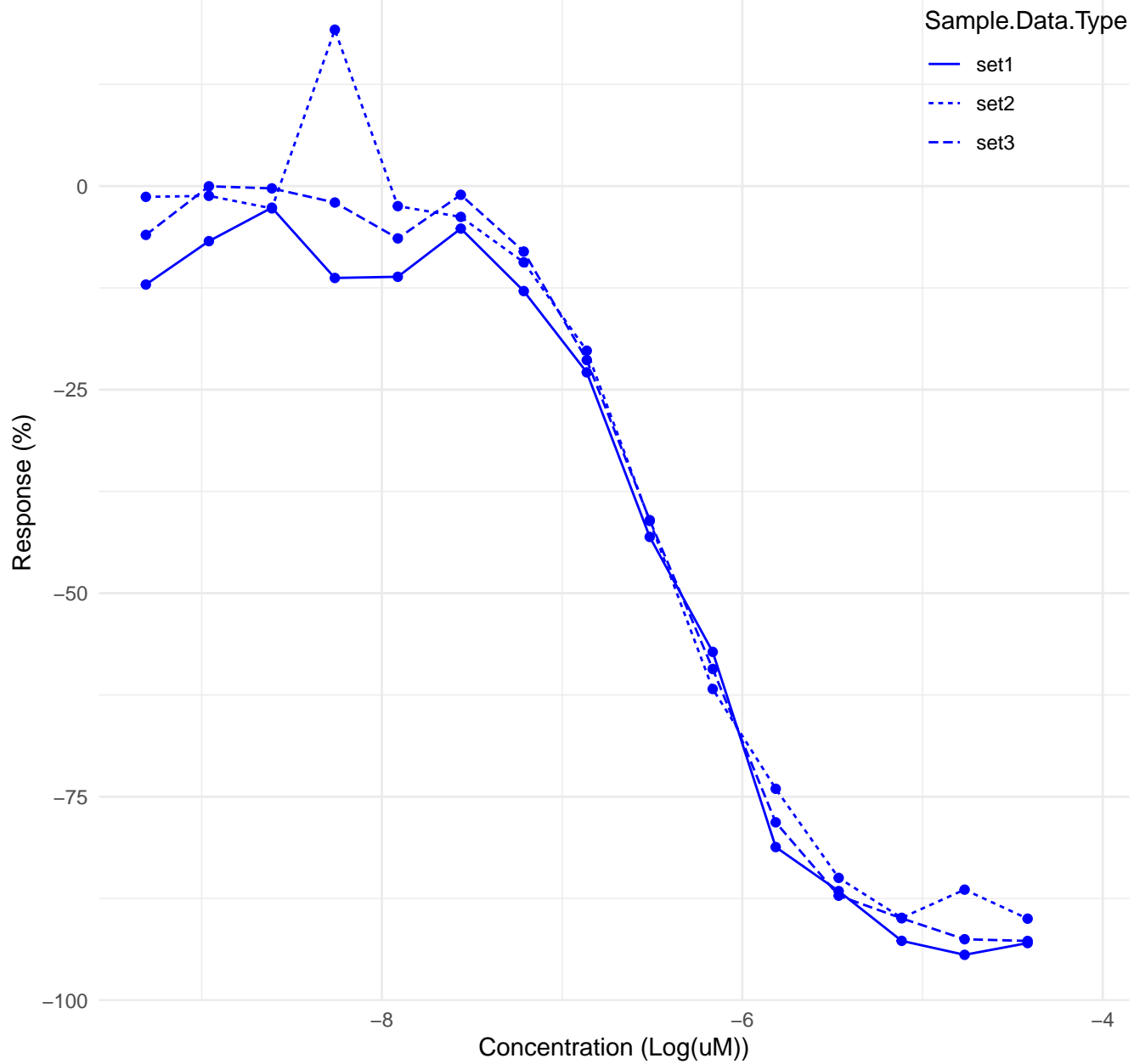

2062-78-4

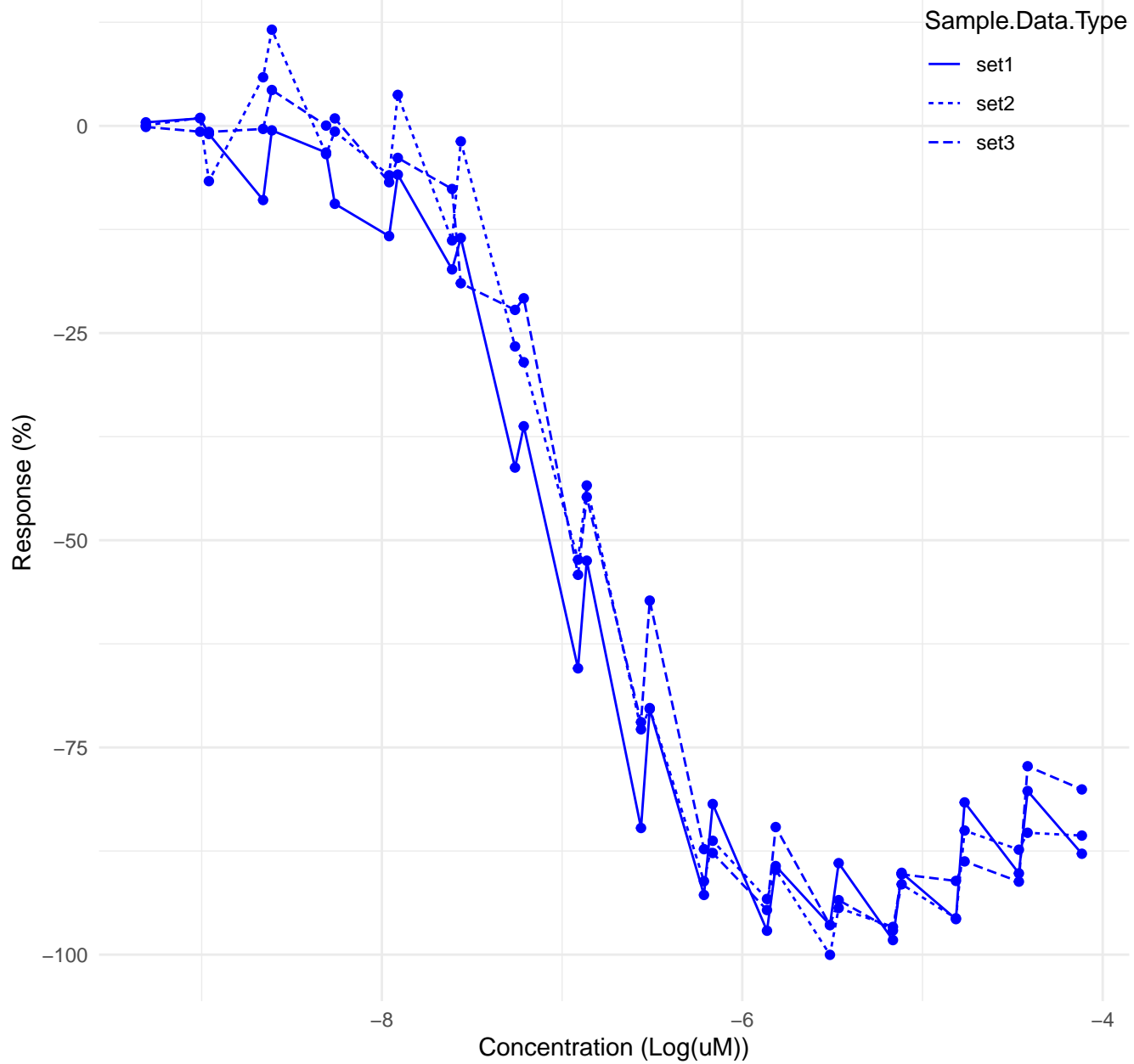

2104-64-5

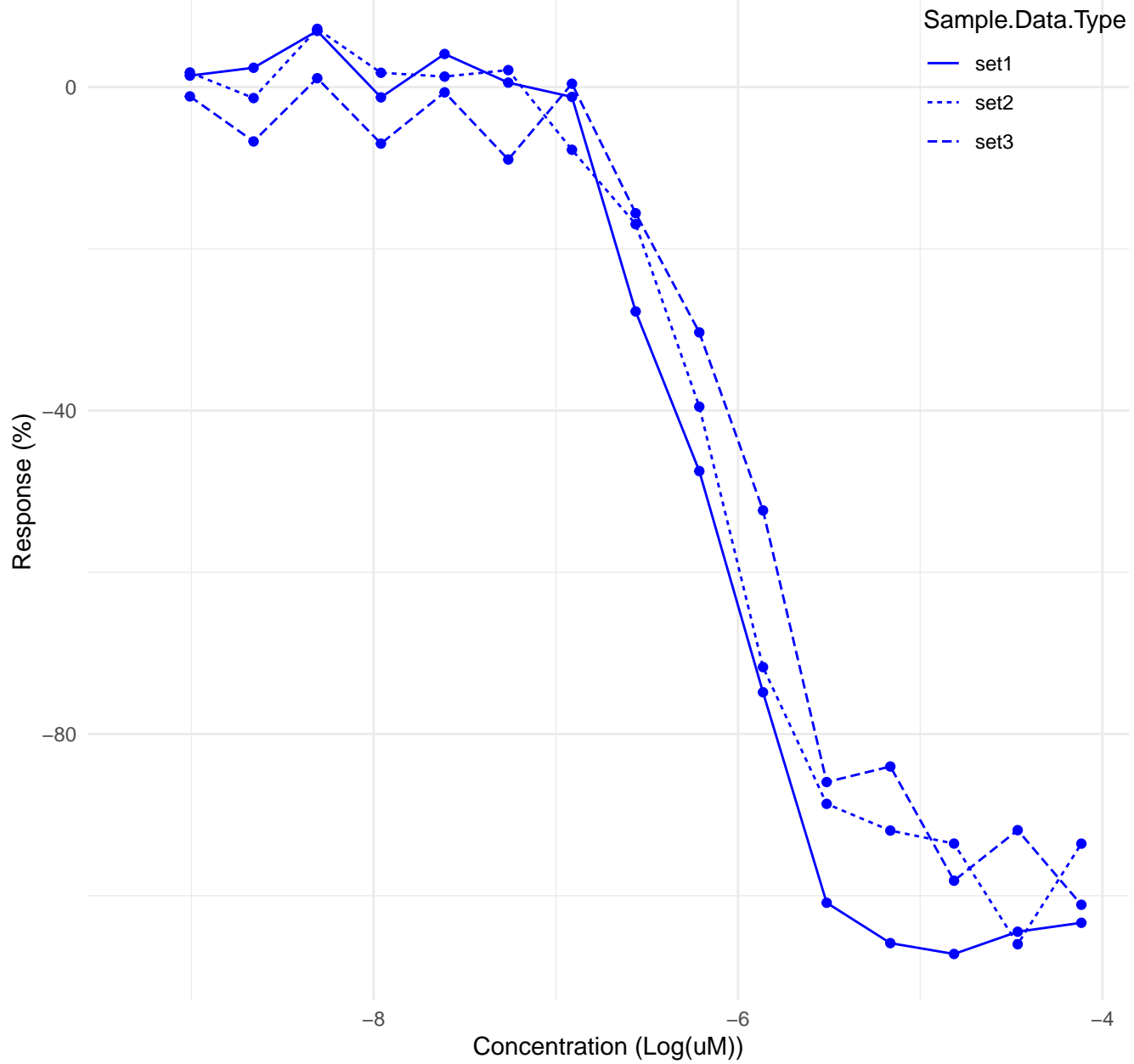

211915-06-9

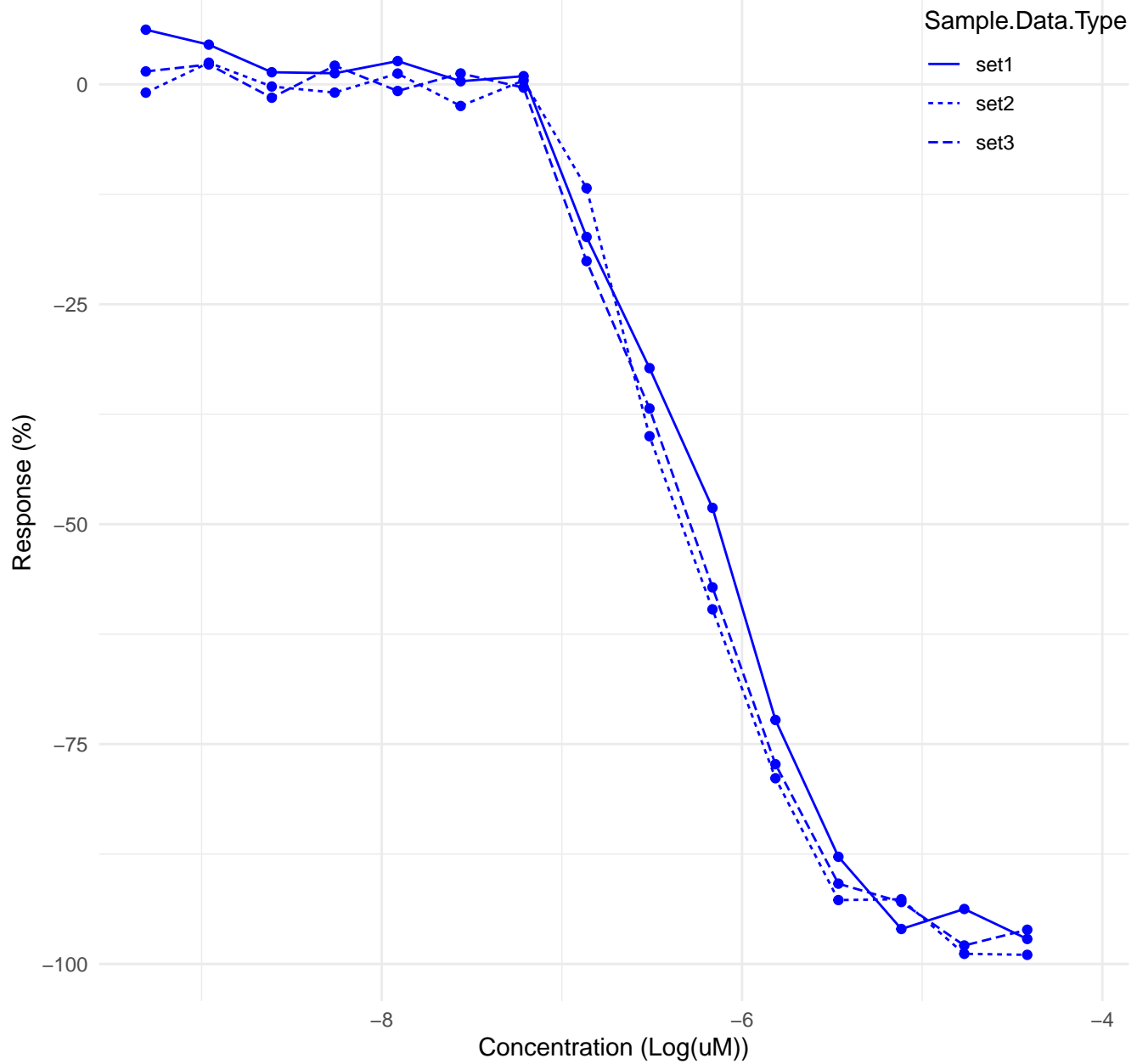

2390-60-5

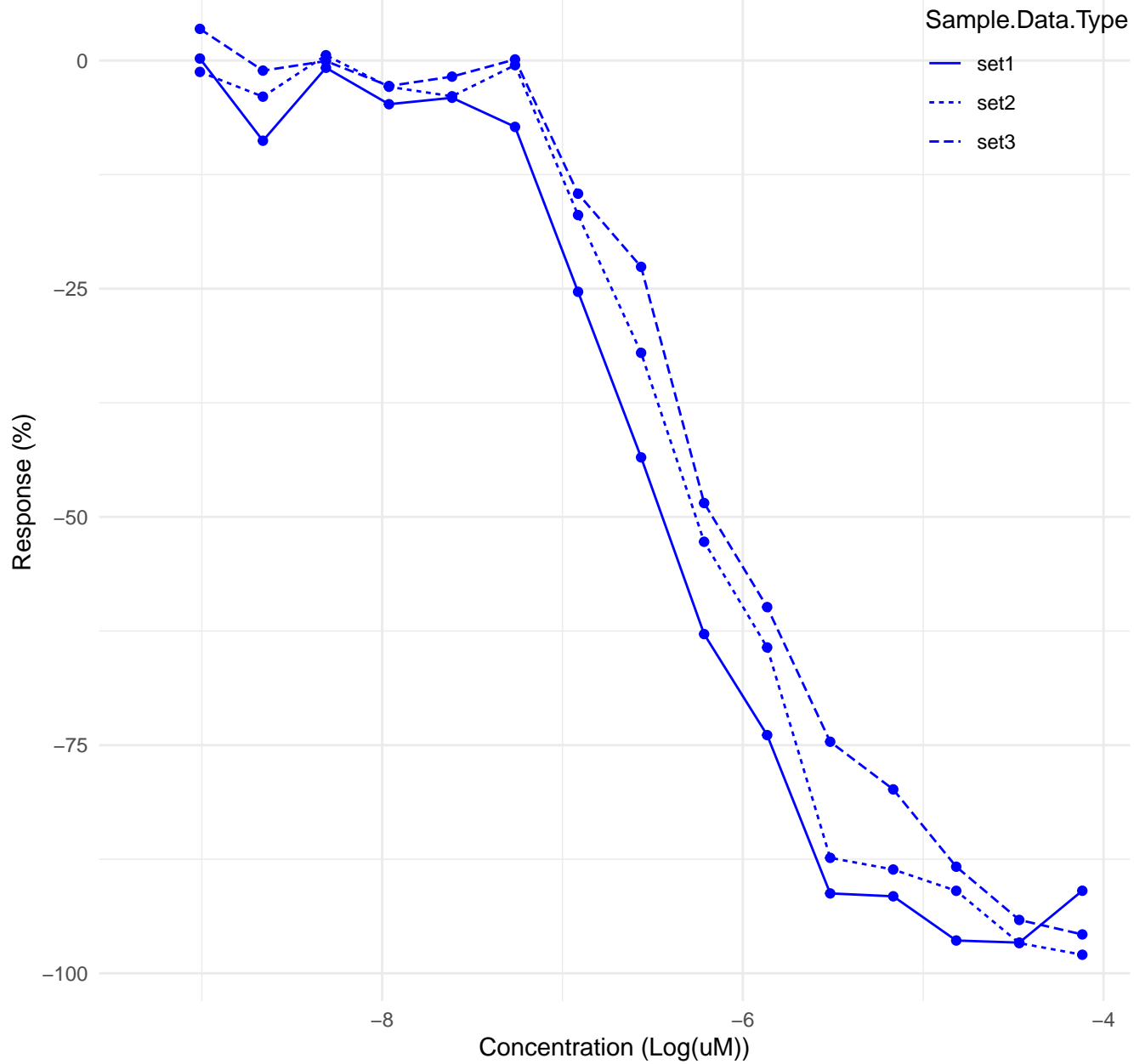

23906-97-0

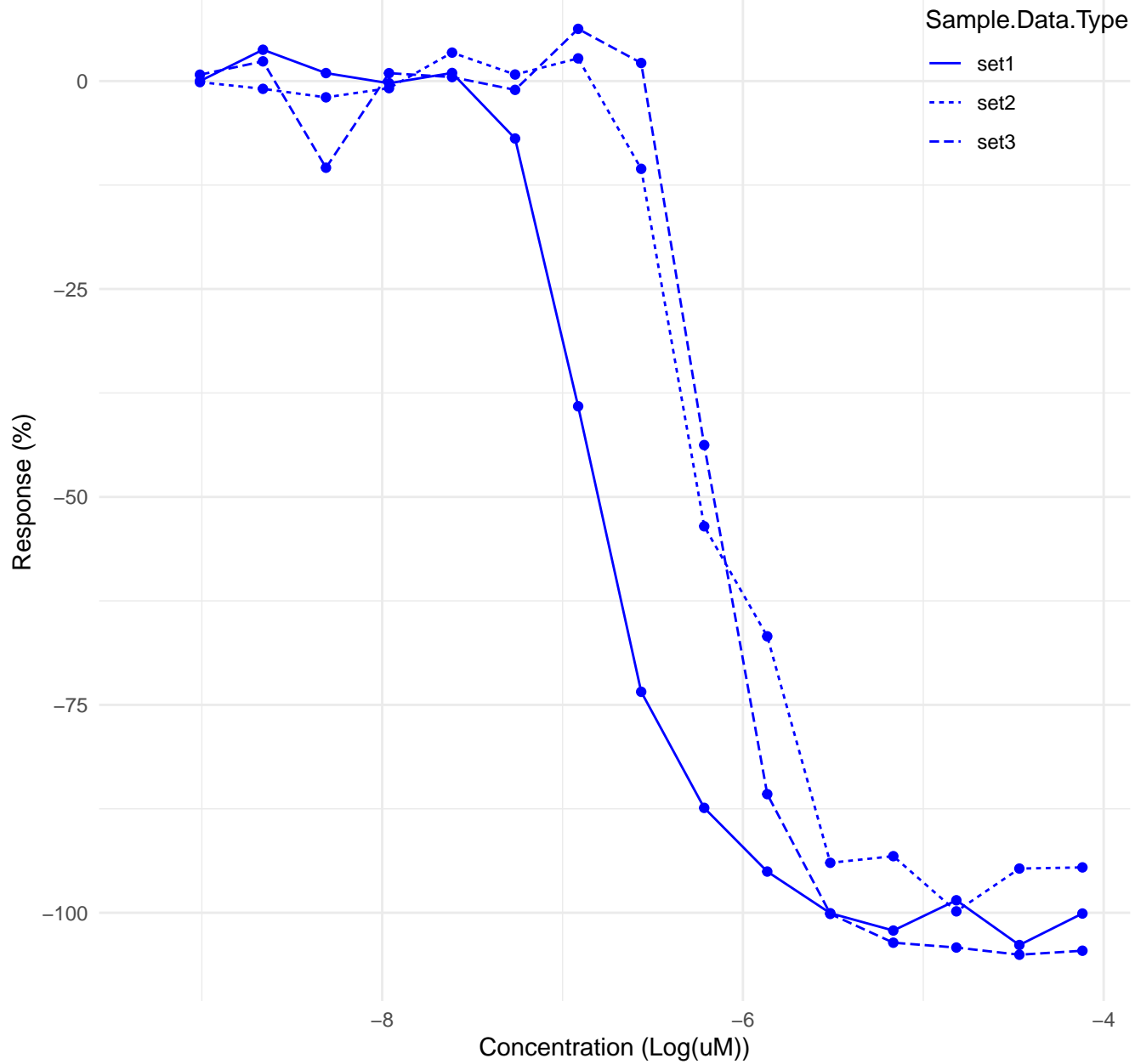

25155-18-4

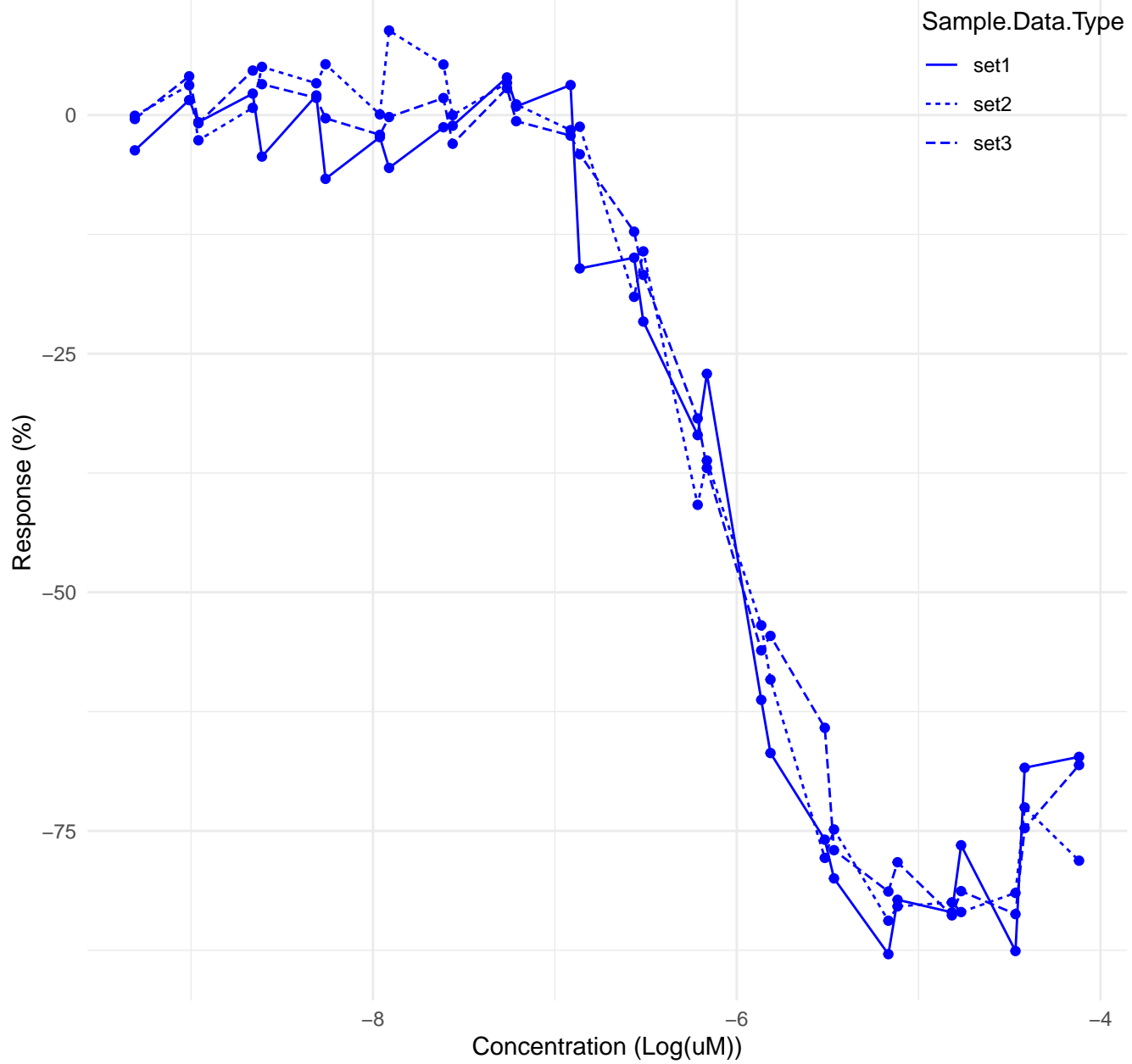

258864-54-9

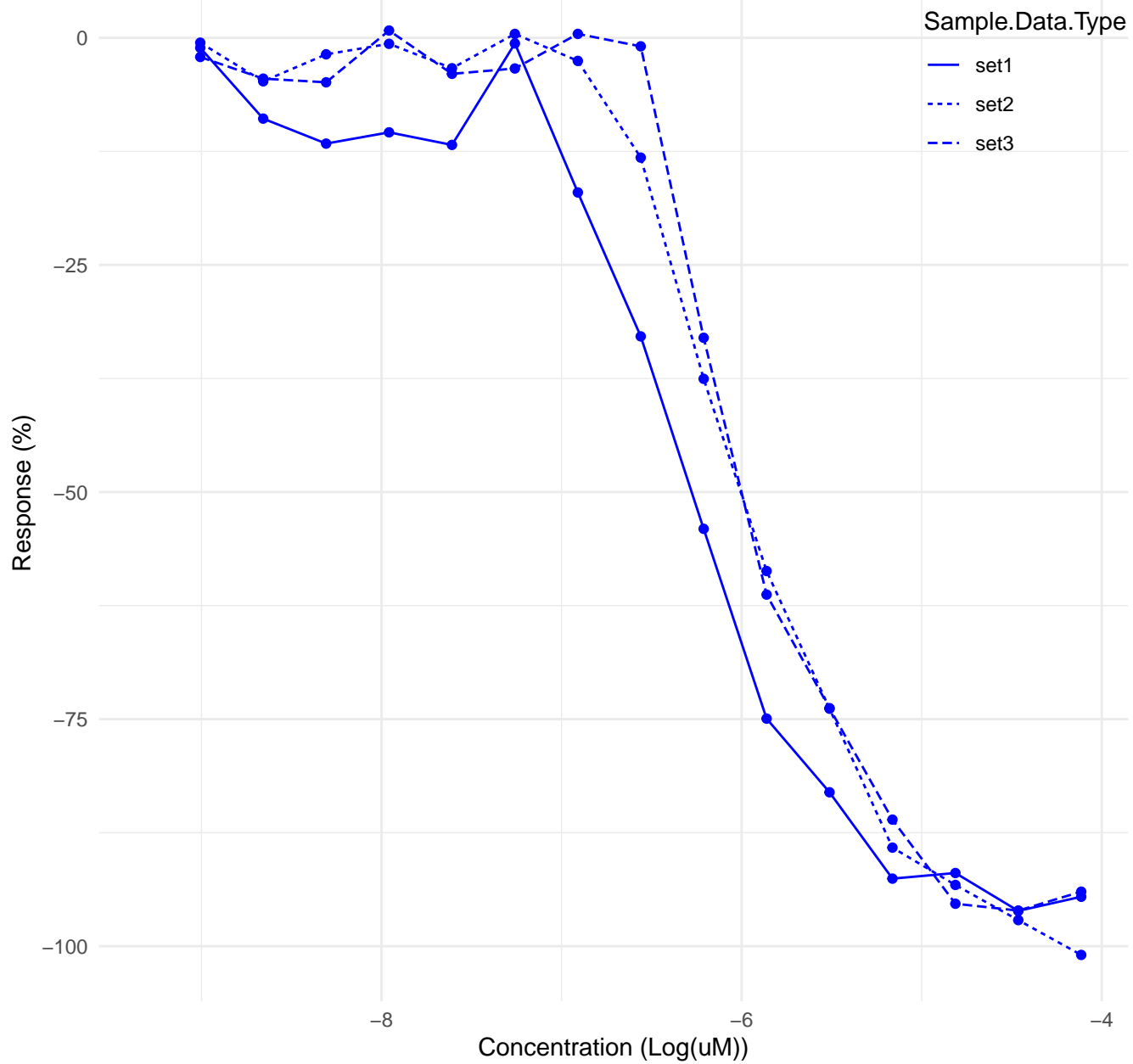

26864-56-2

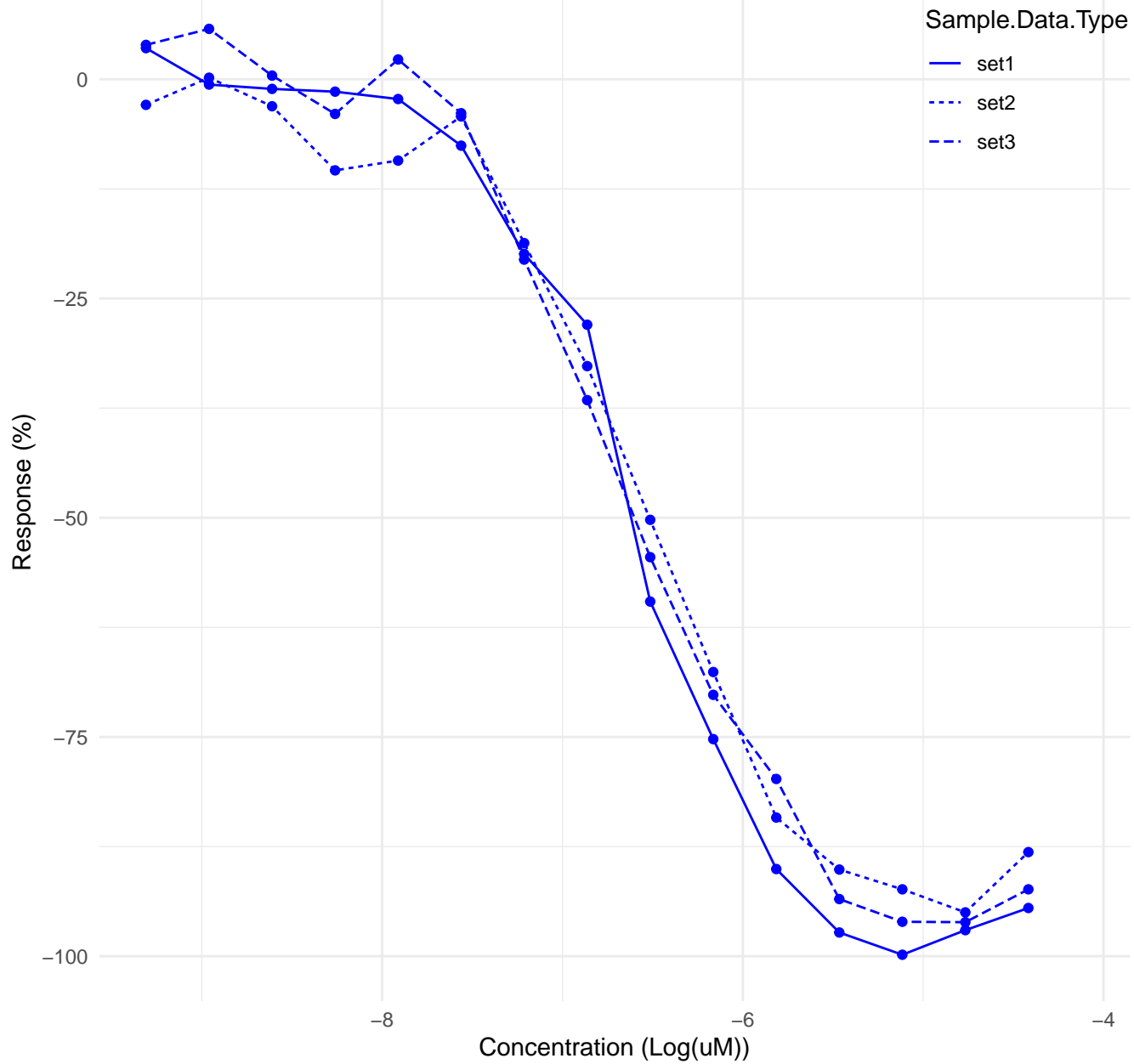

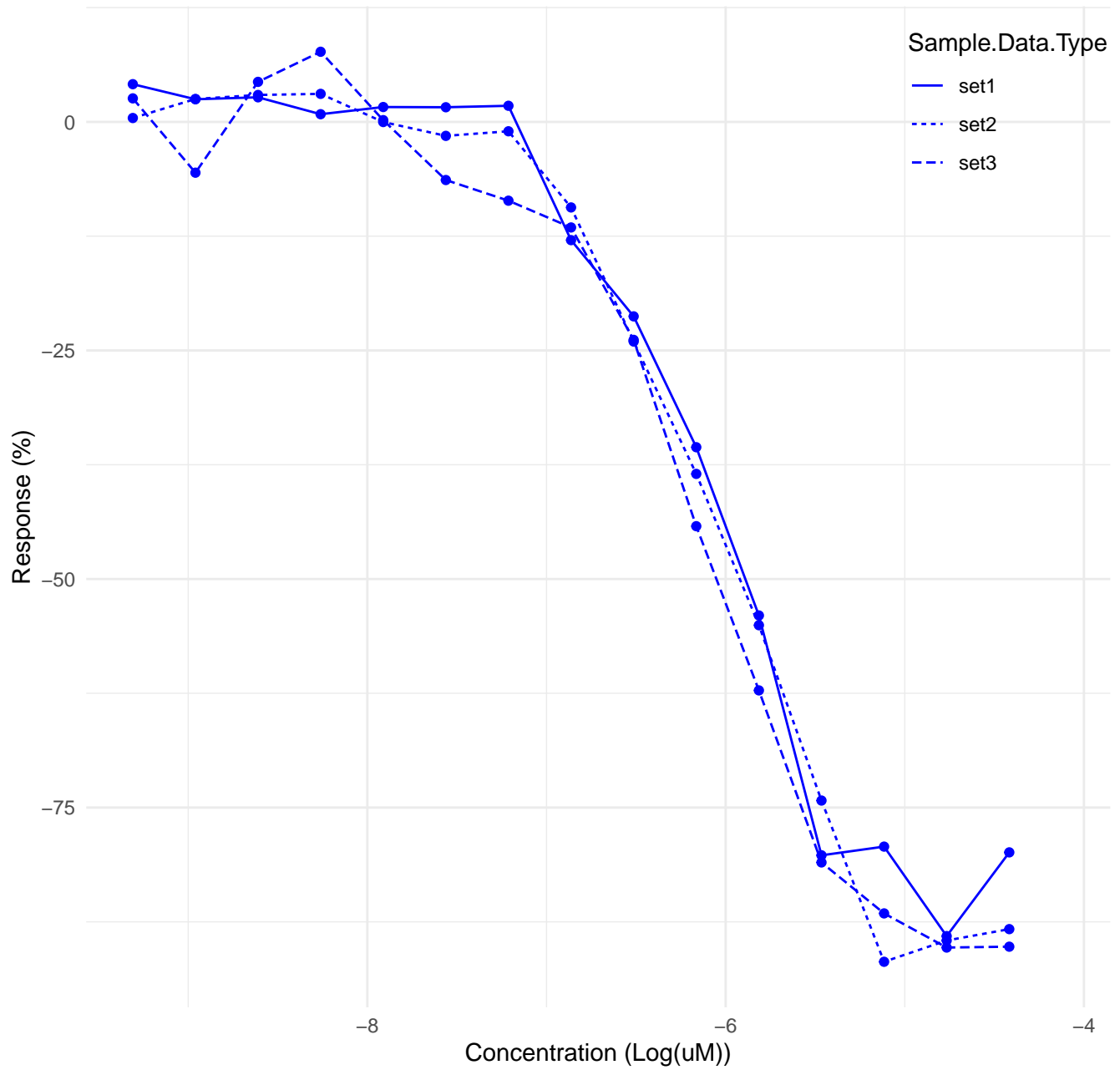

3416-26-0

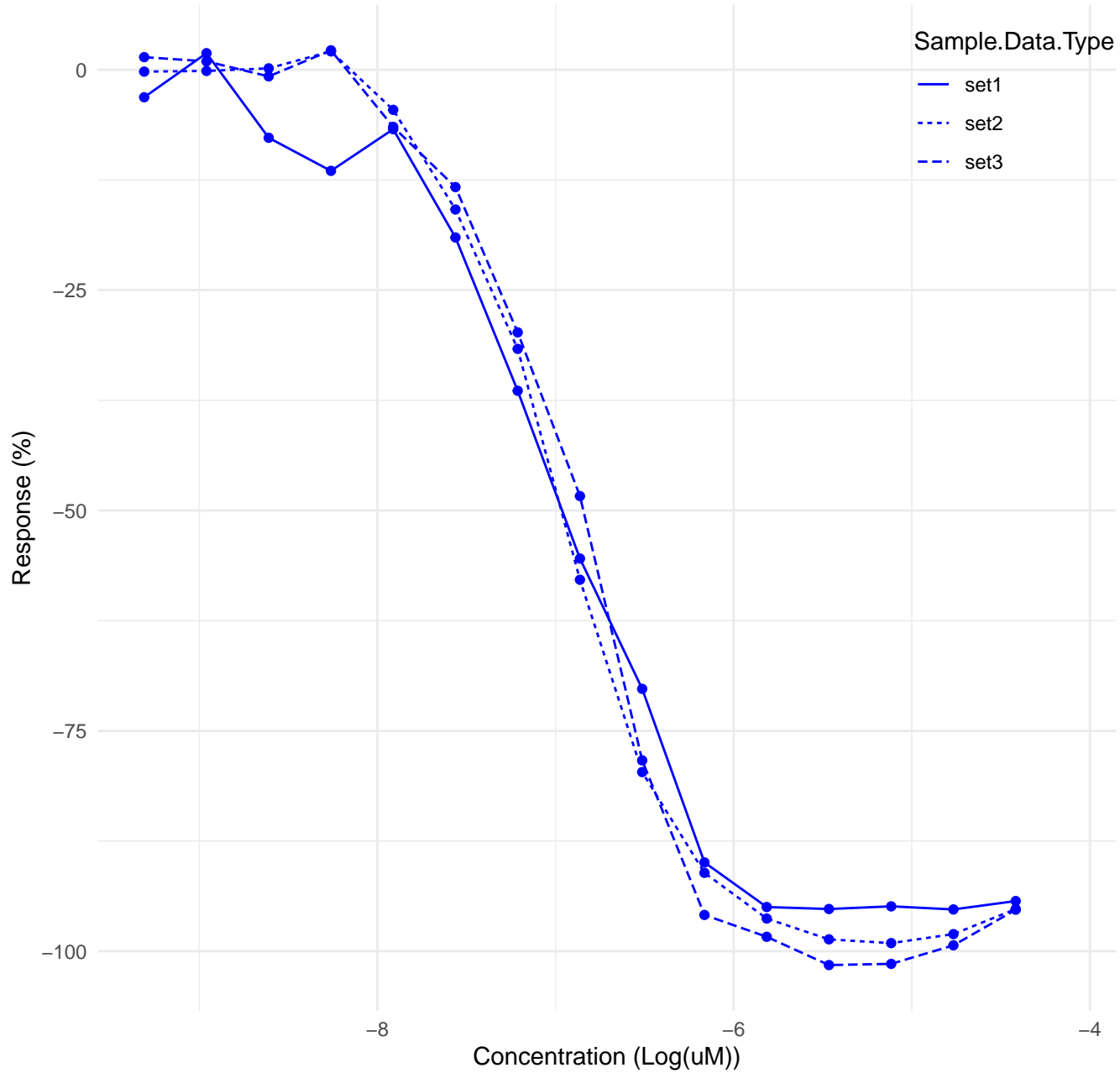

36167-63-2

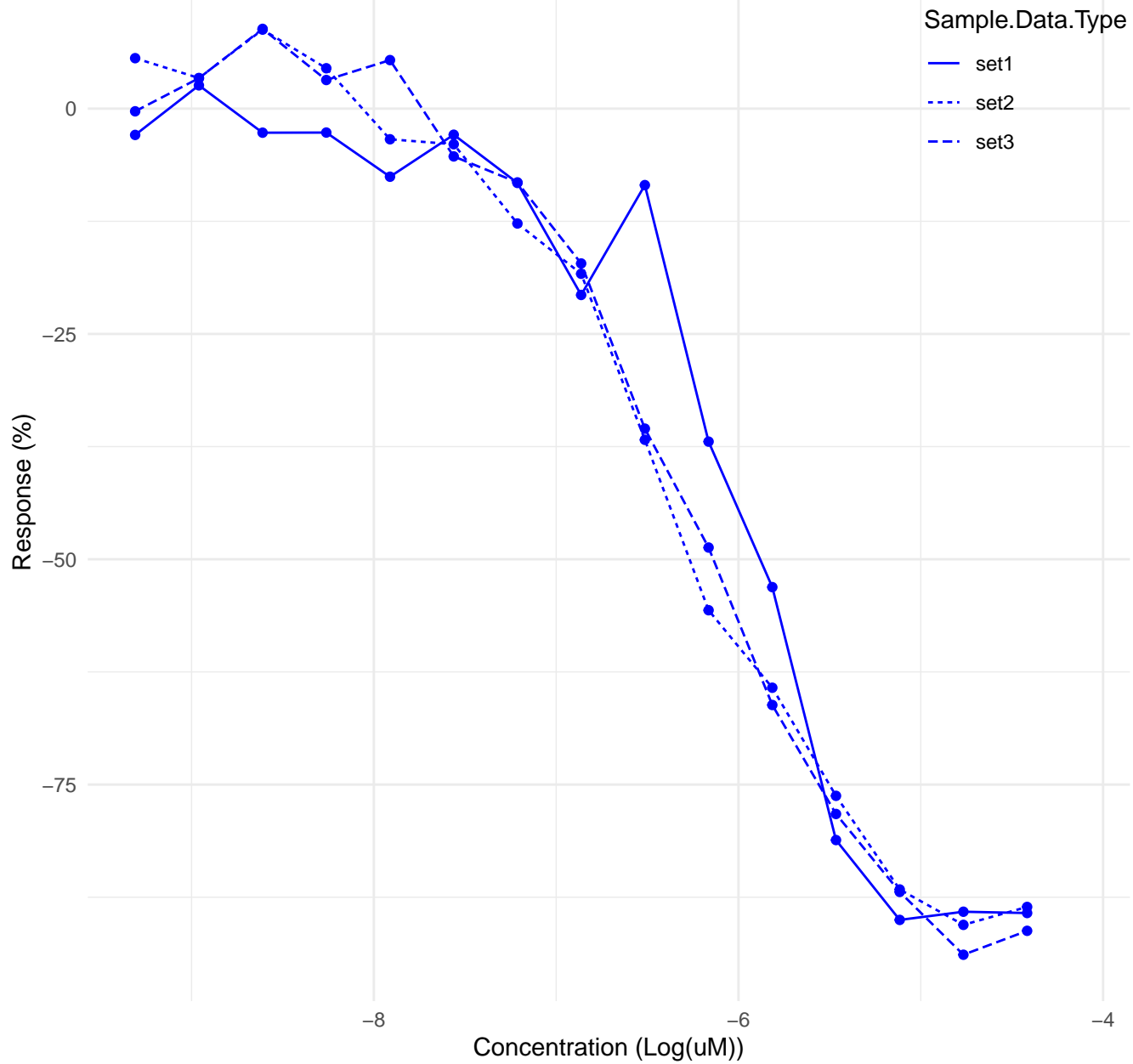

3735-85-1

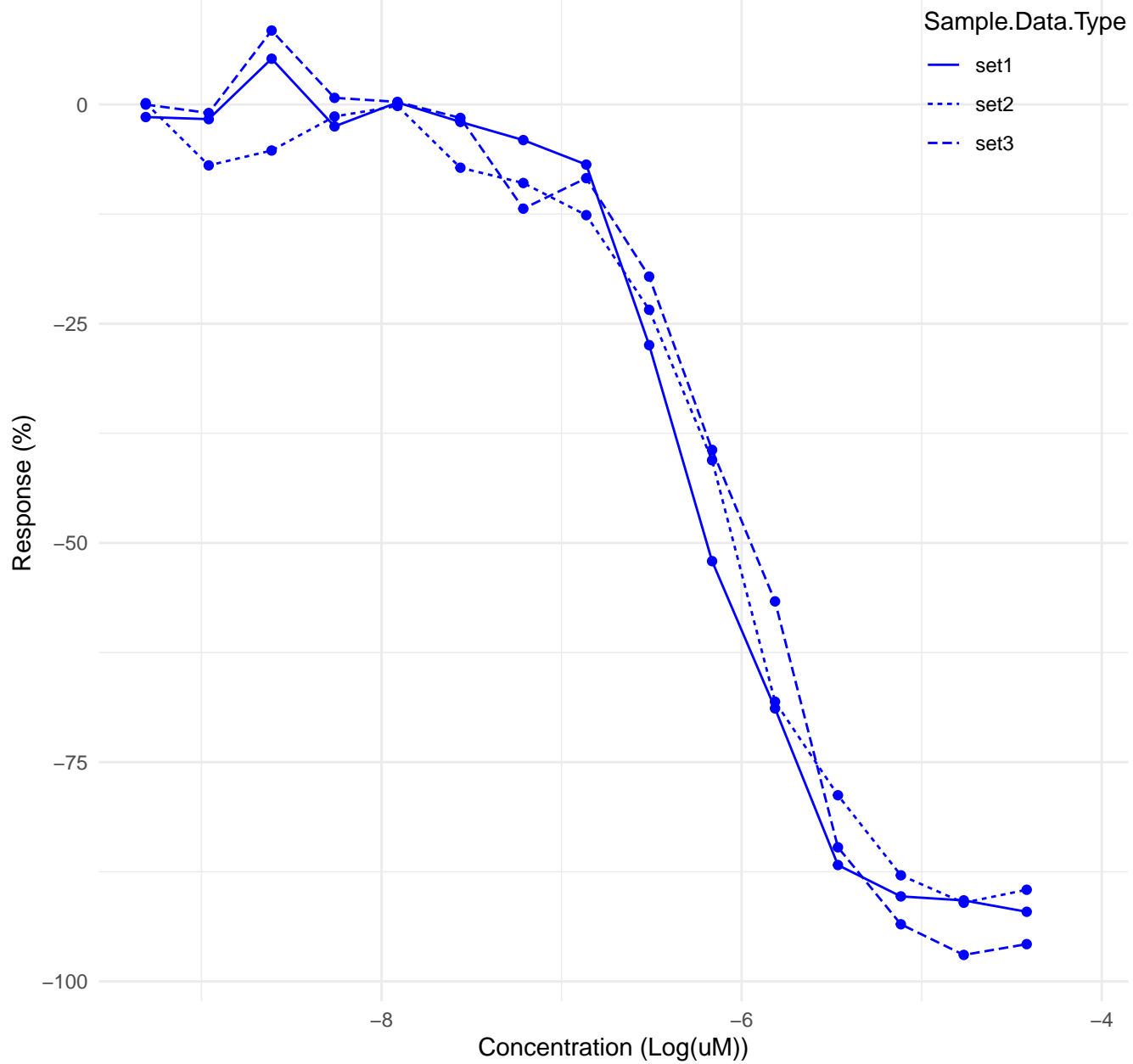

374683-44-0

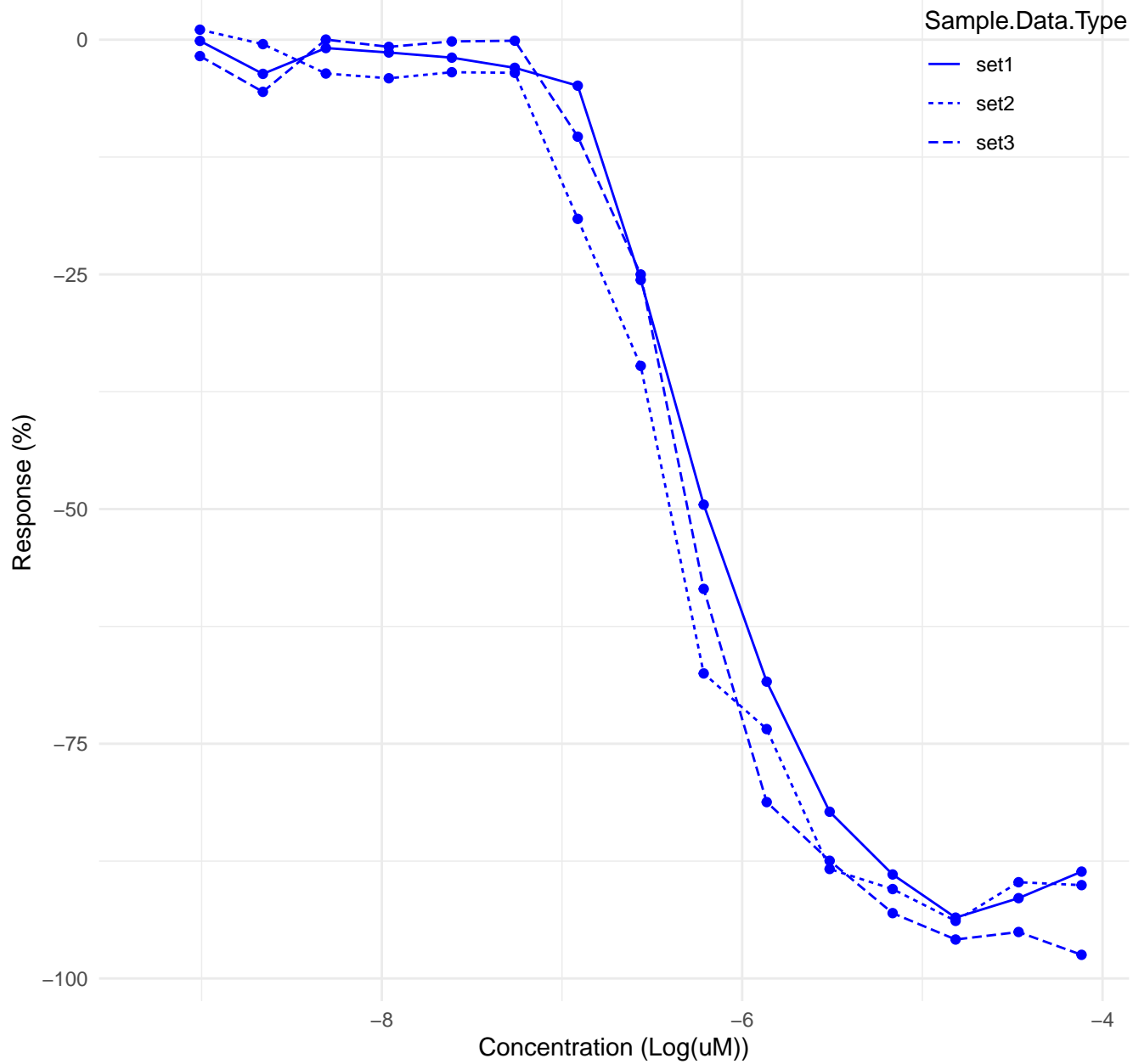

375395-33-8

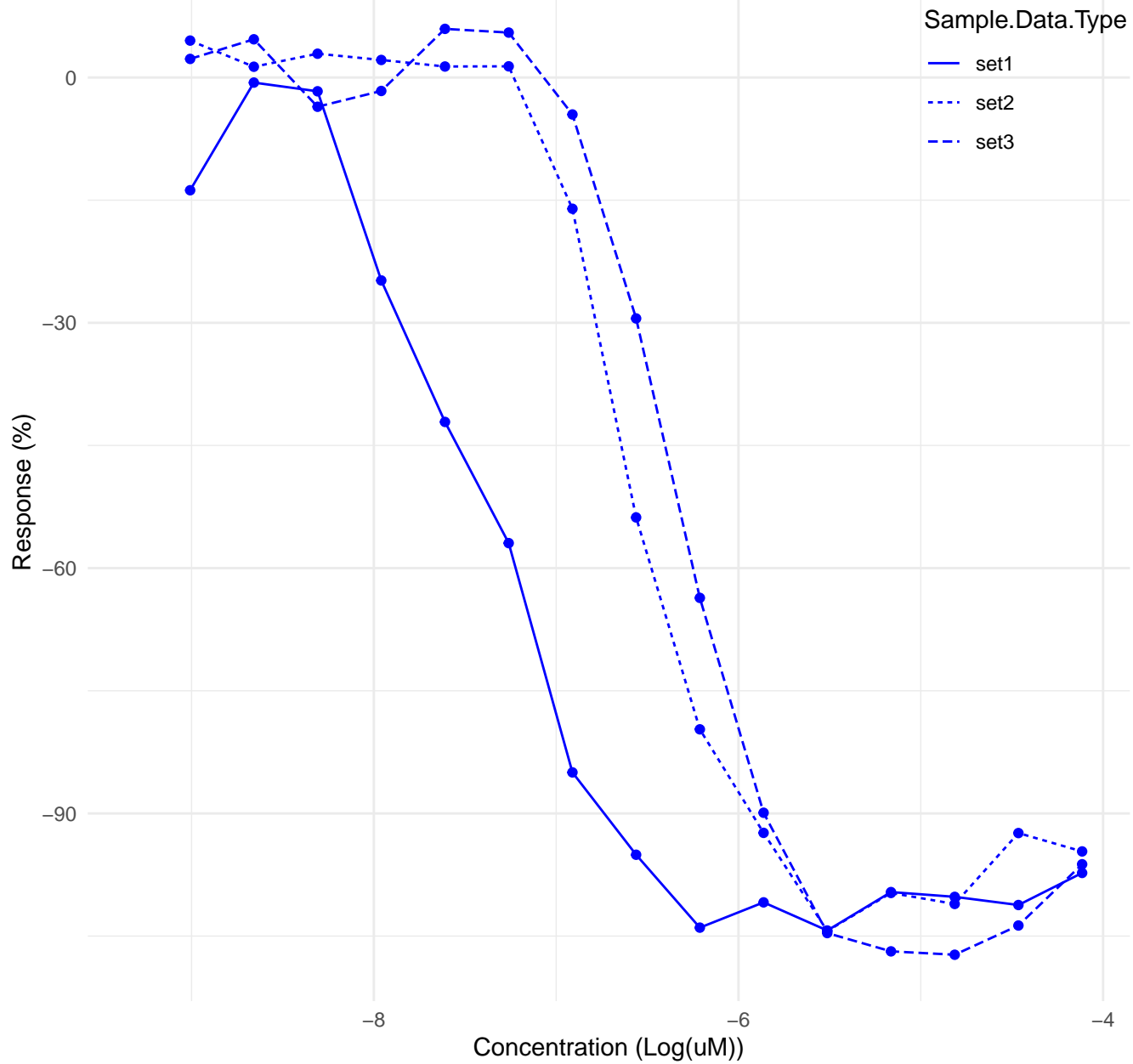

3810-80-8

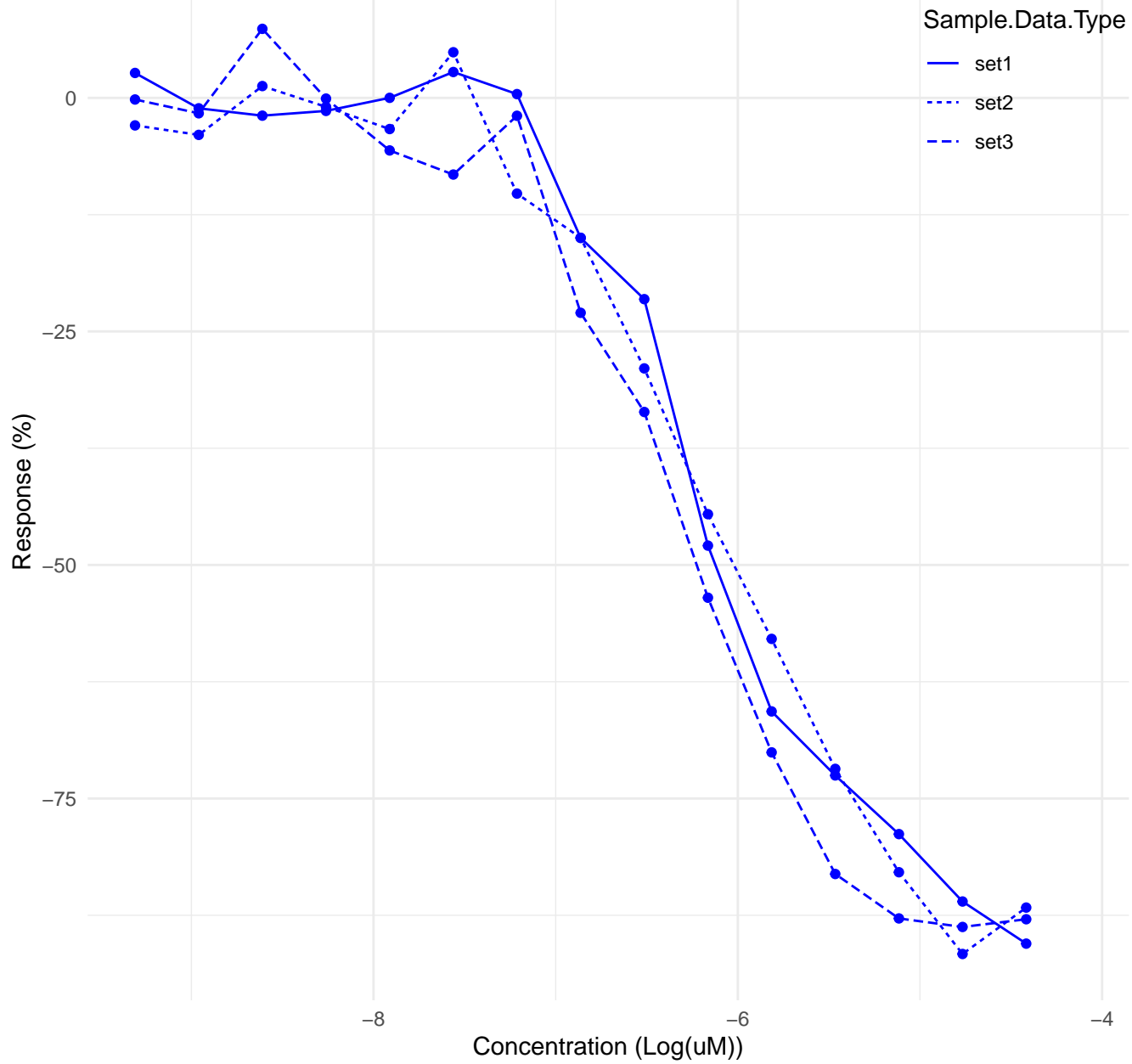

42971-09-5

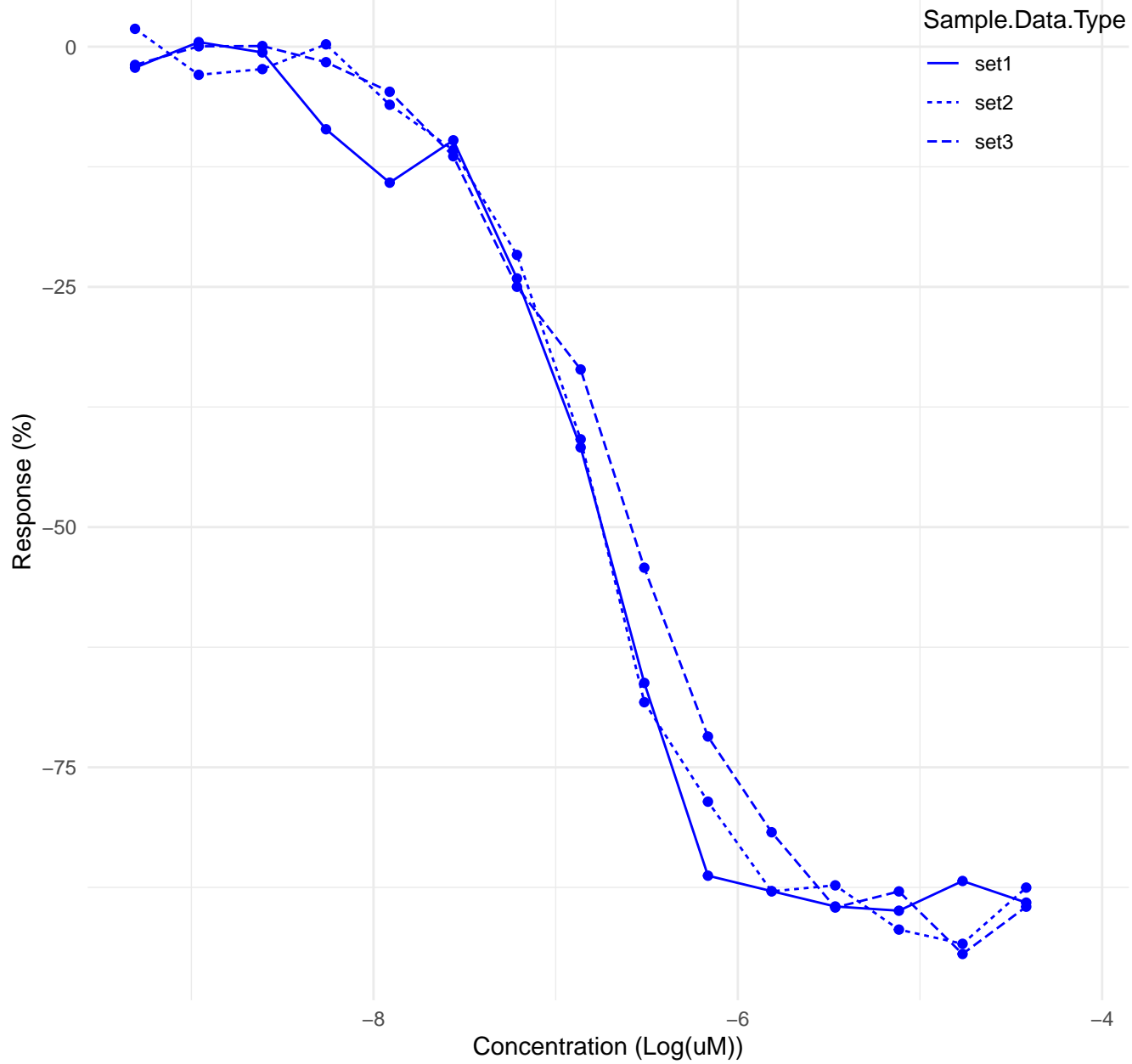

460092-03-9

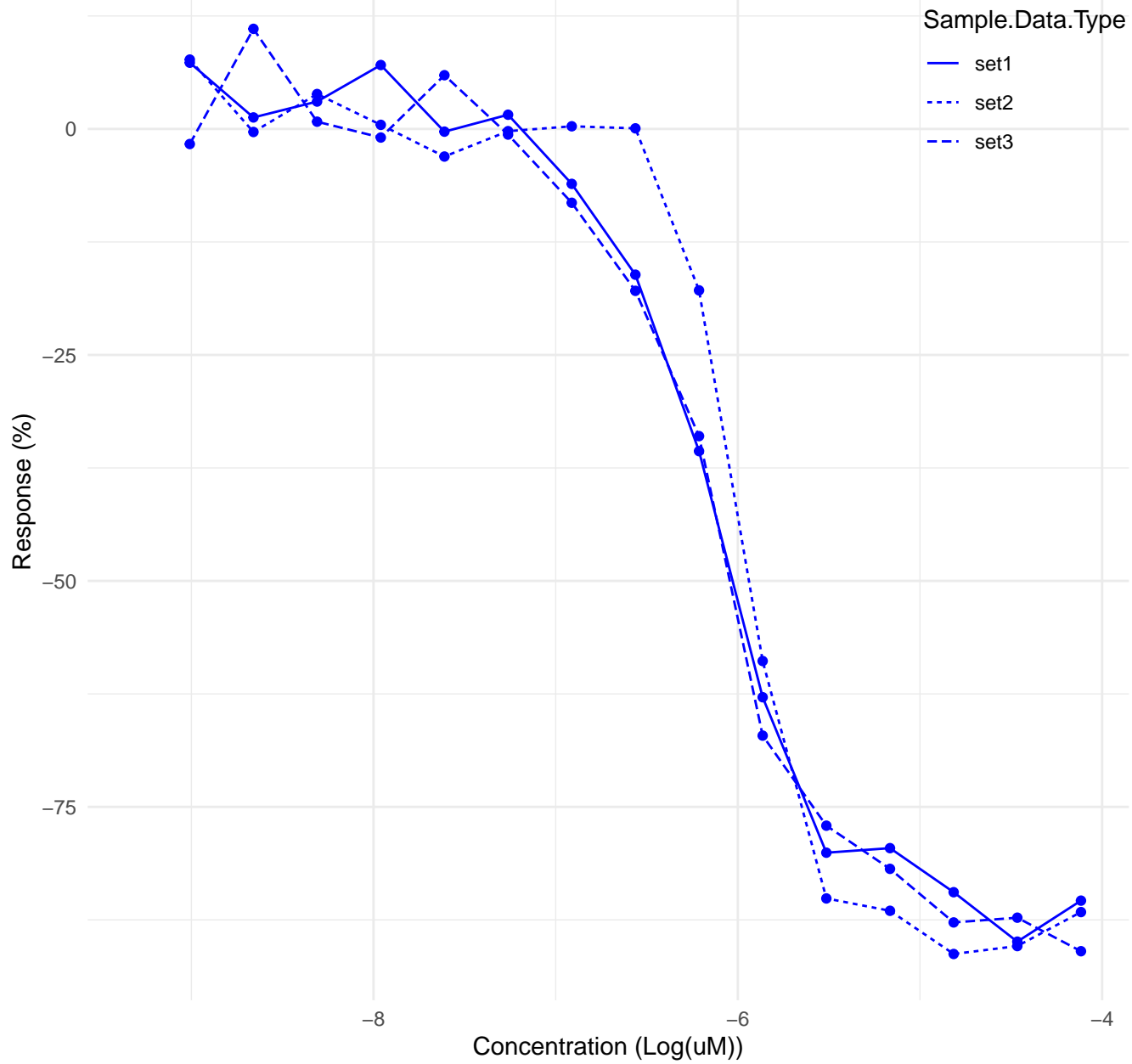

4789-68-8

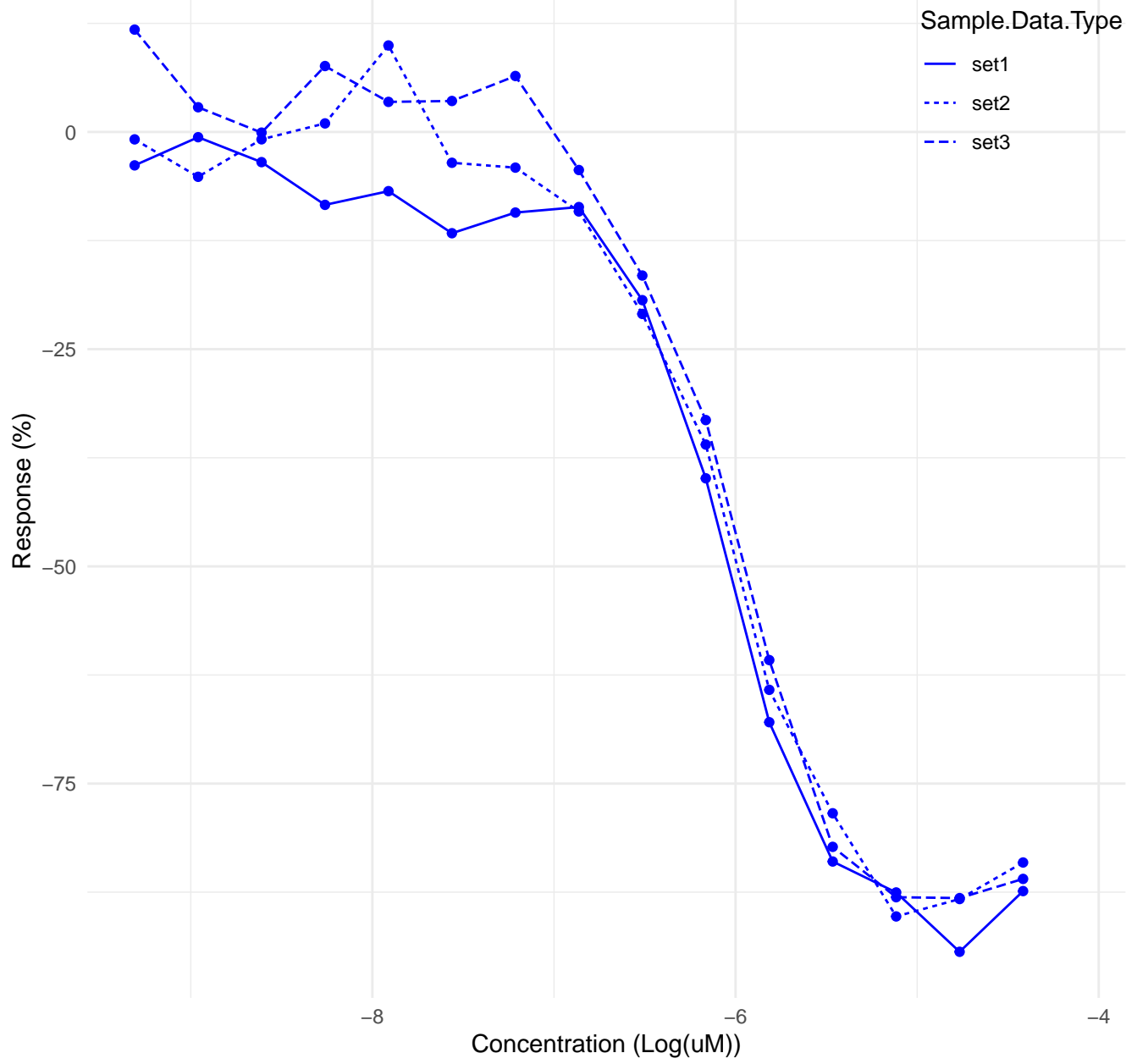

50679-08-8

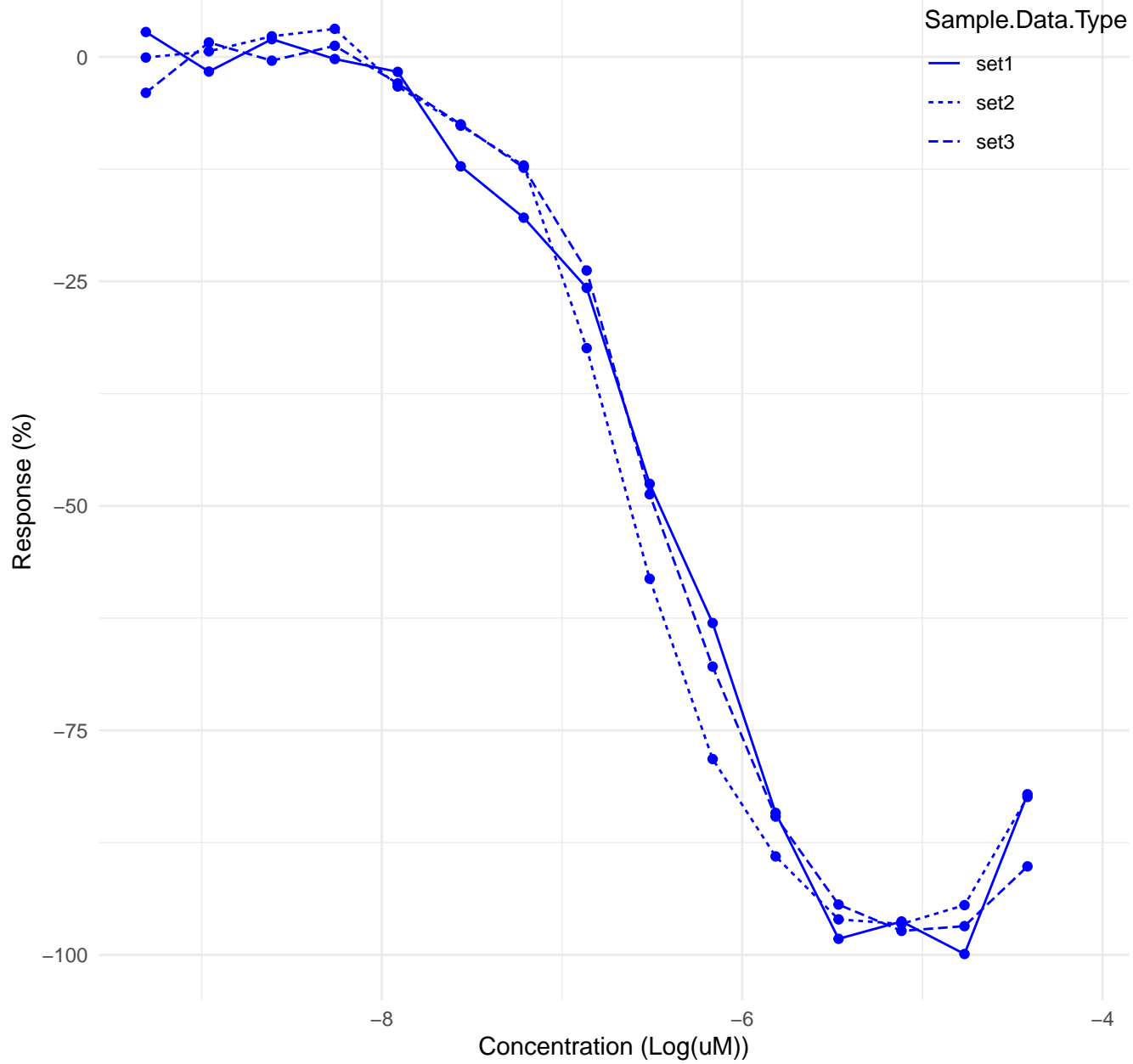

510-74-7

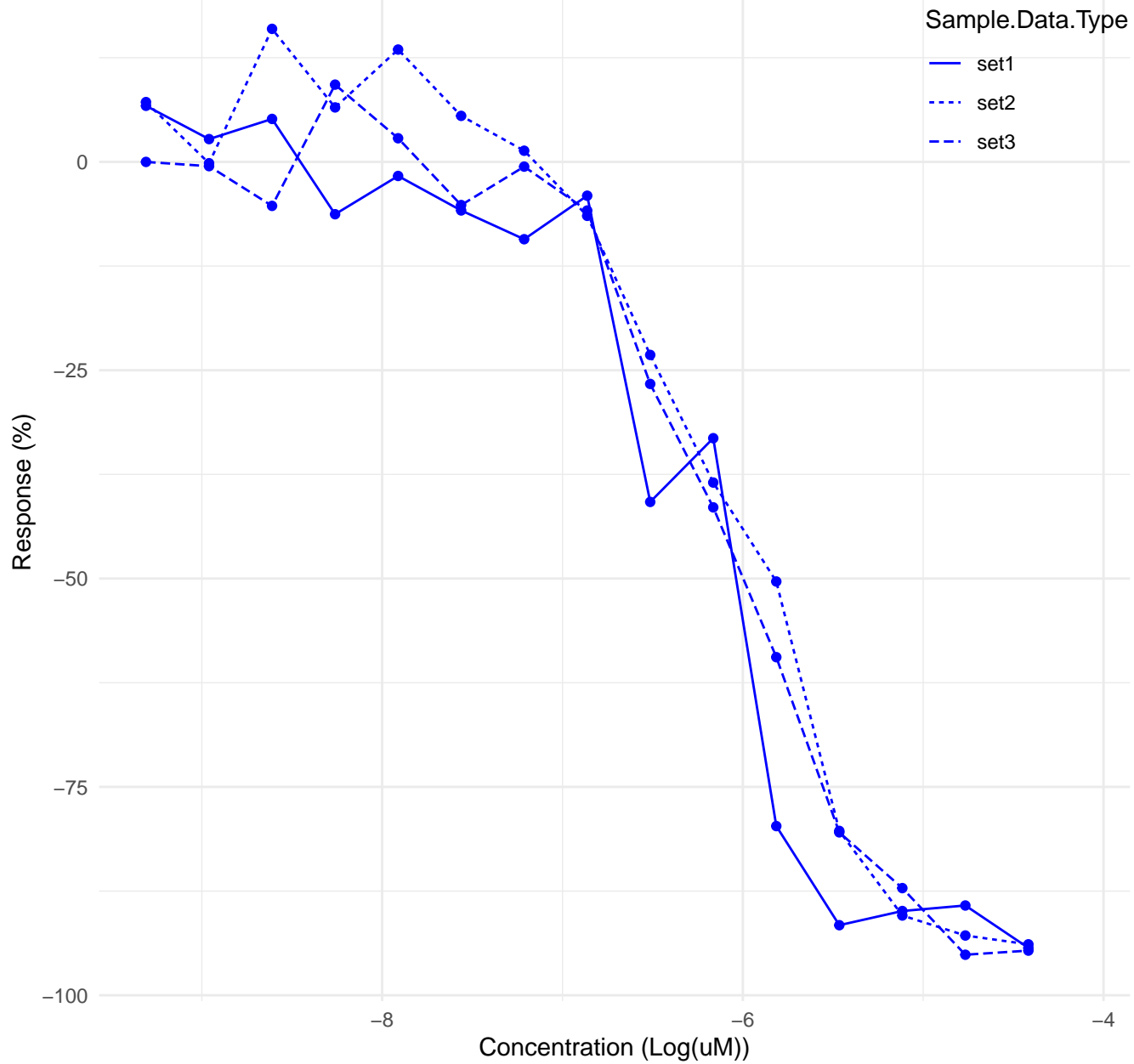

5137-55-3

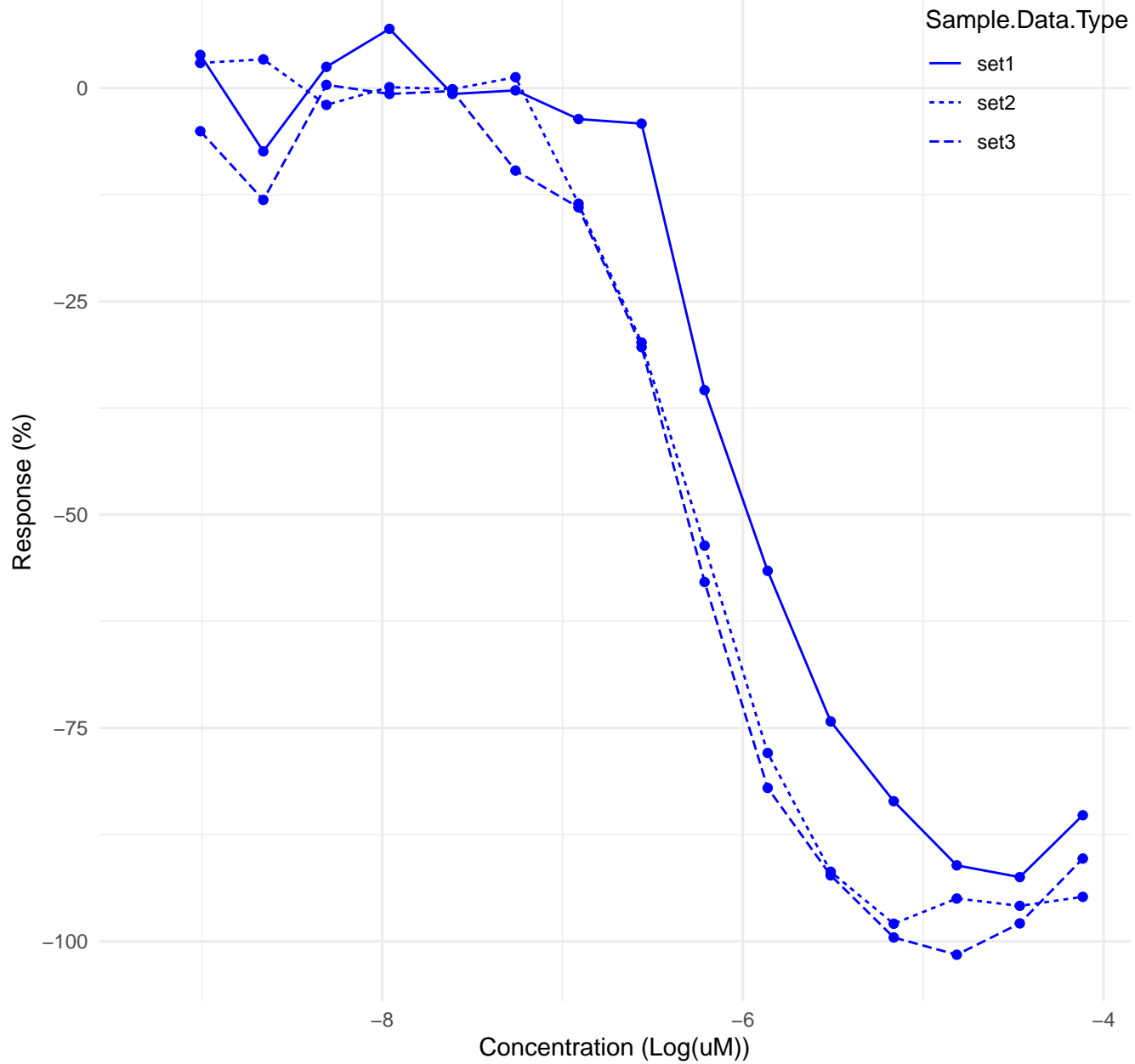

52-86-8

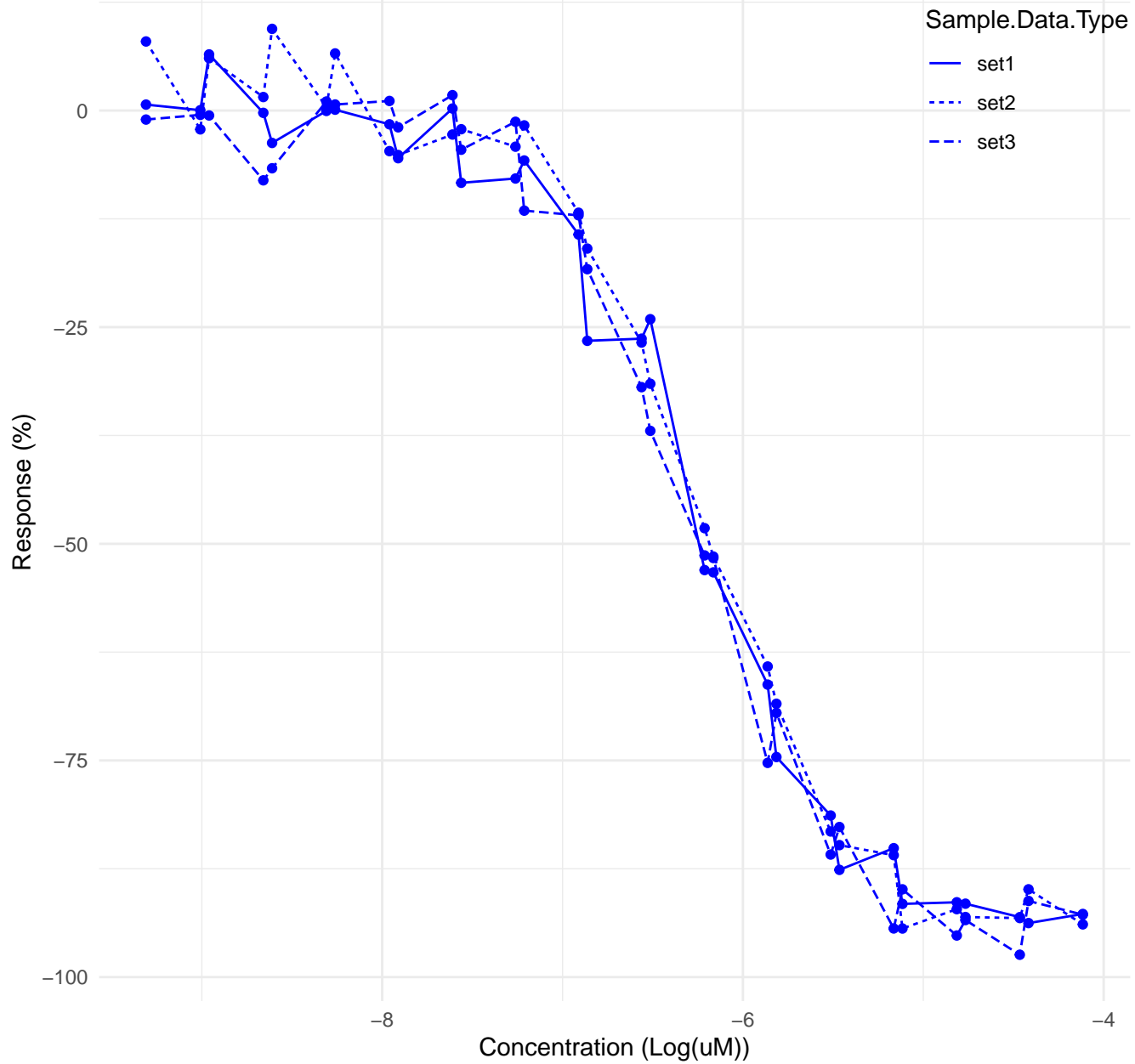

52468-60-7

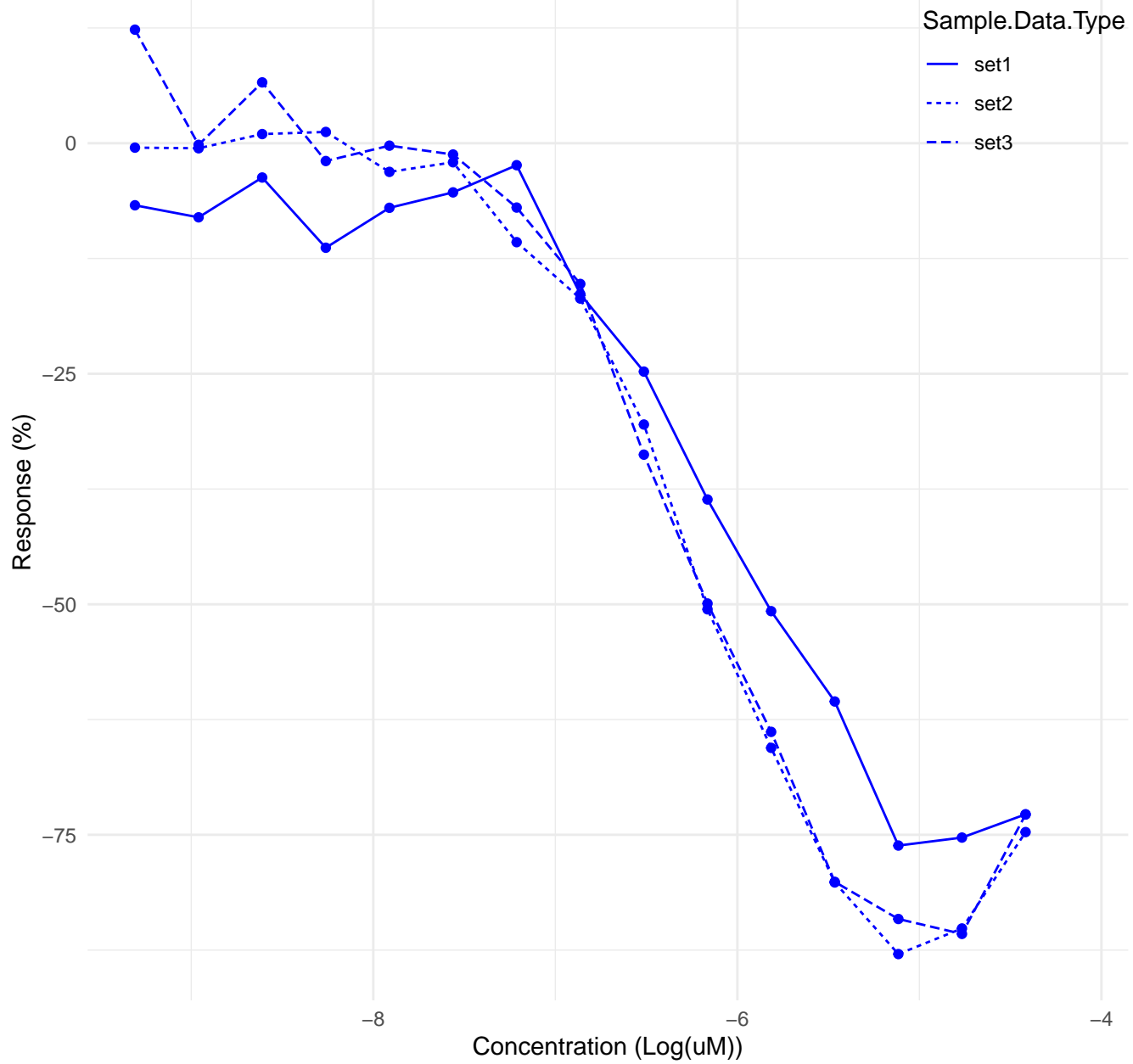

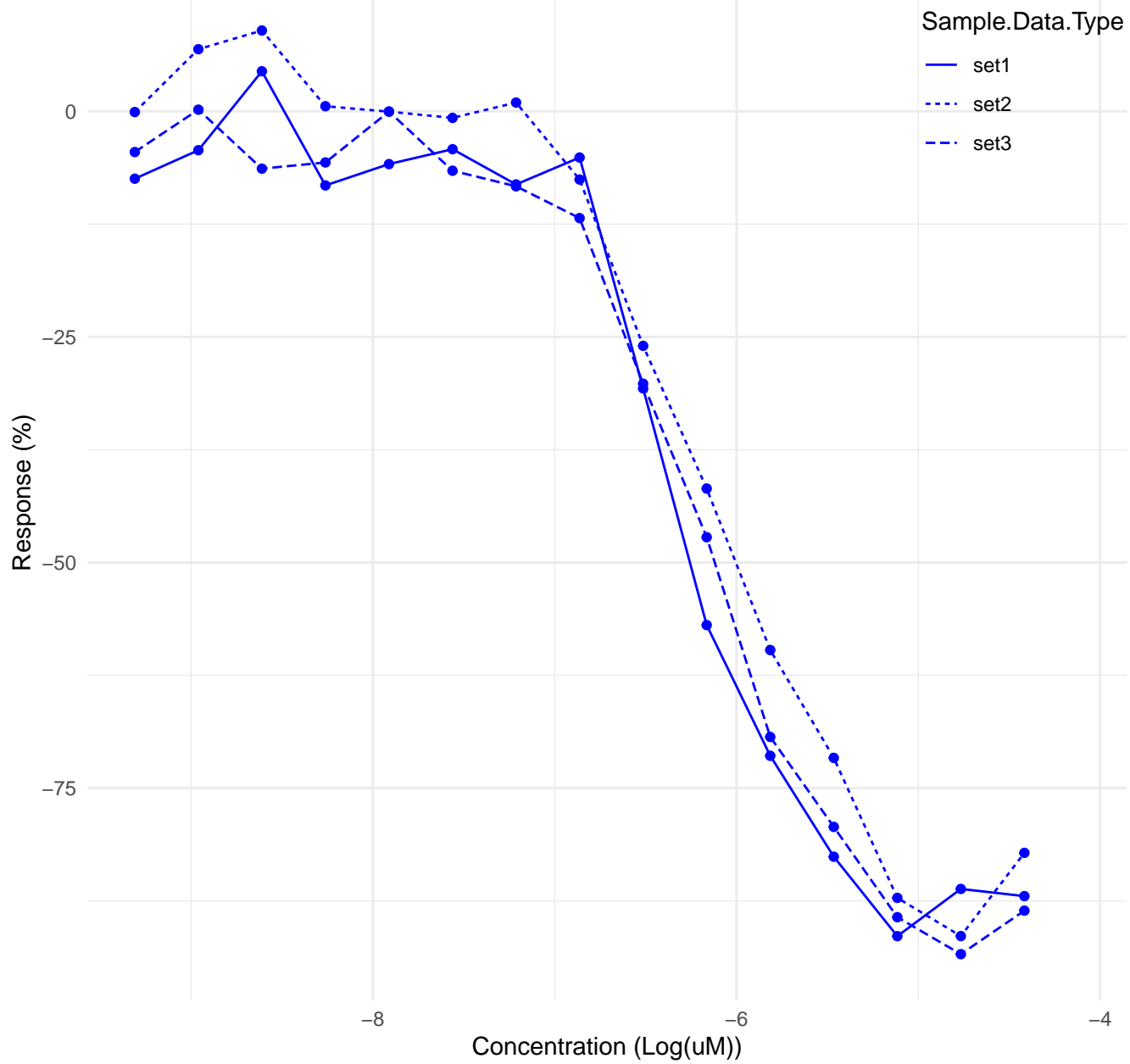

553-08-2

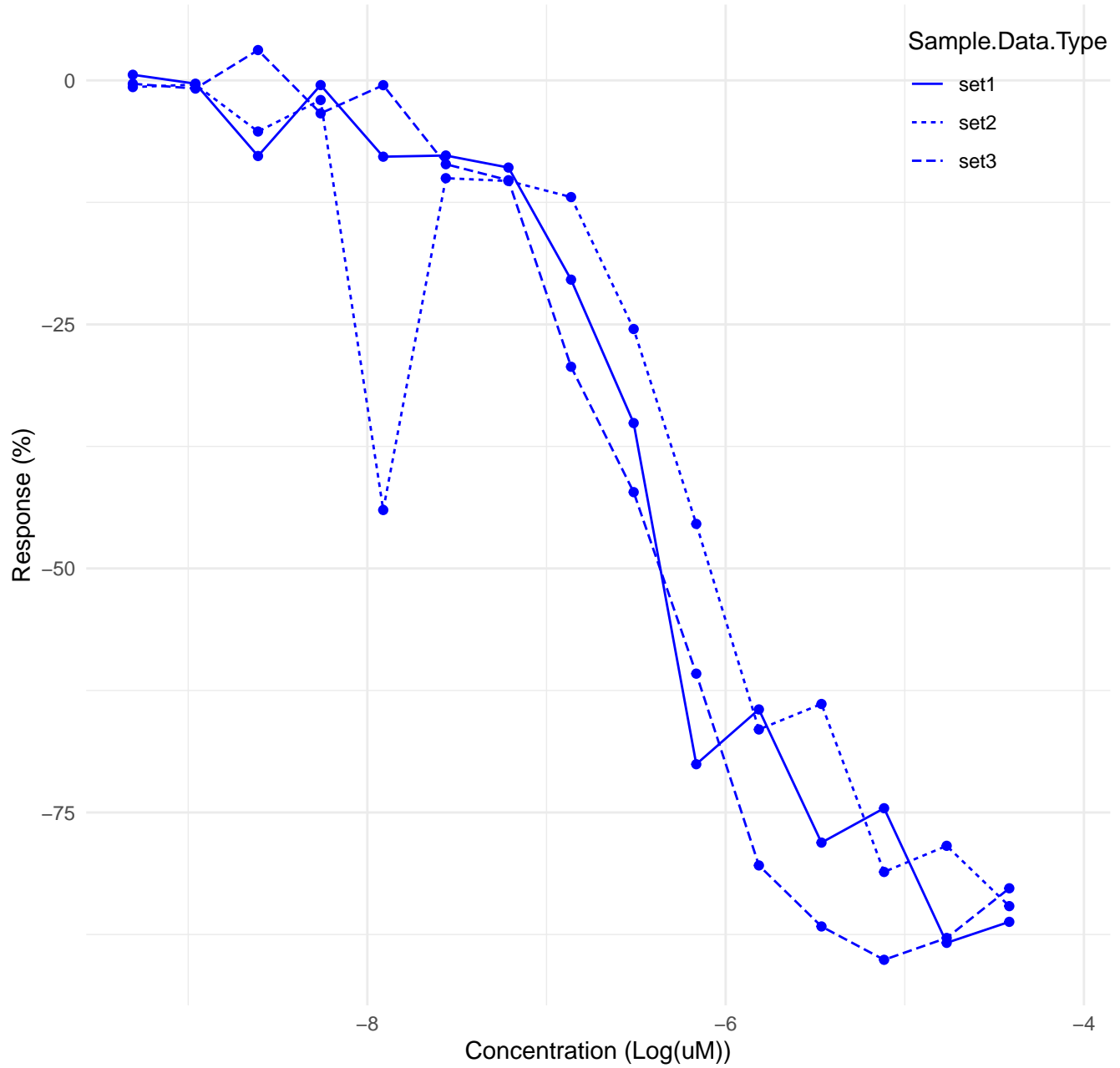

57132-53-3

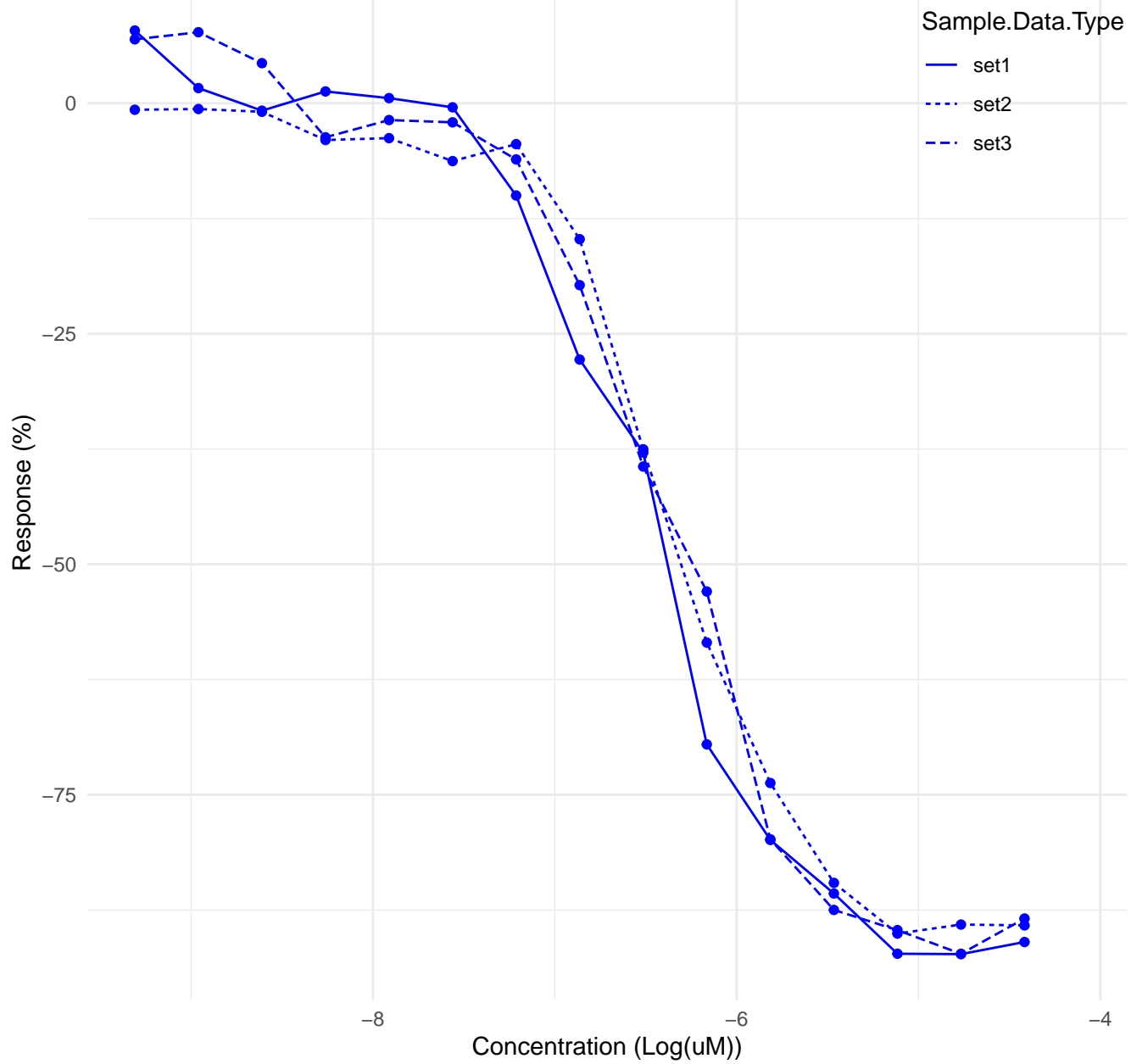

57808-66-9

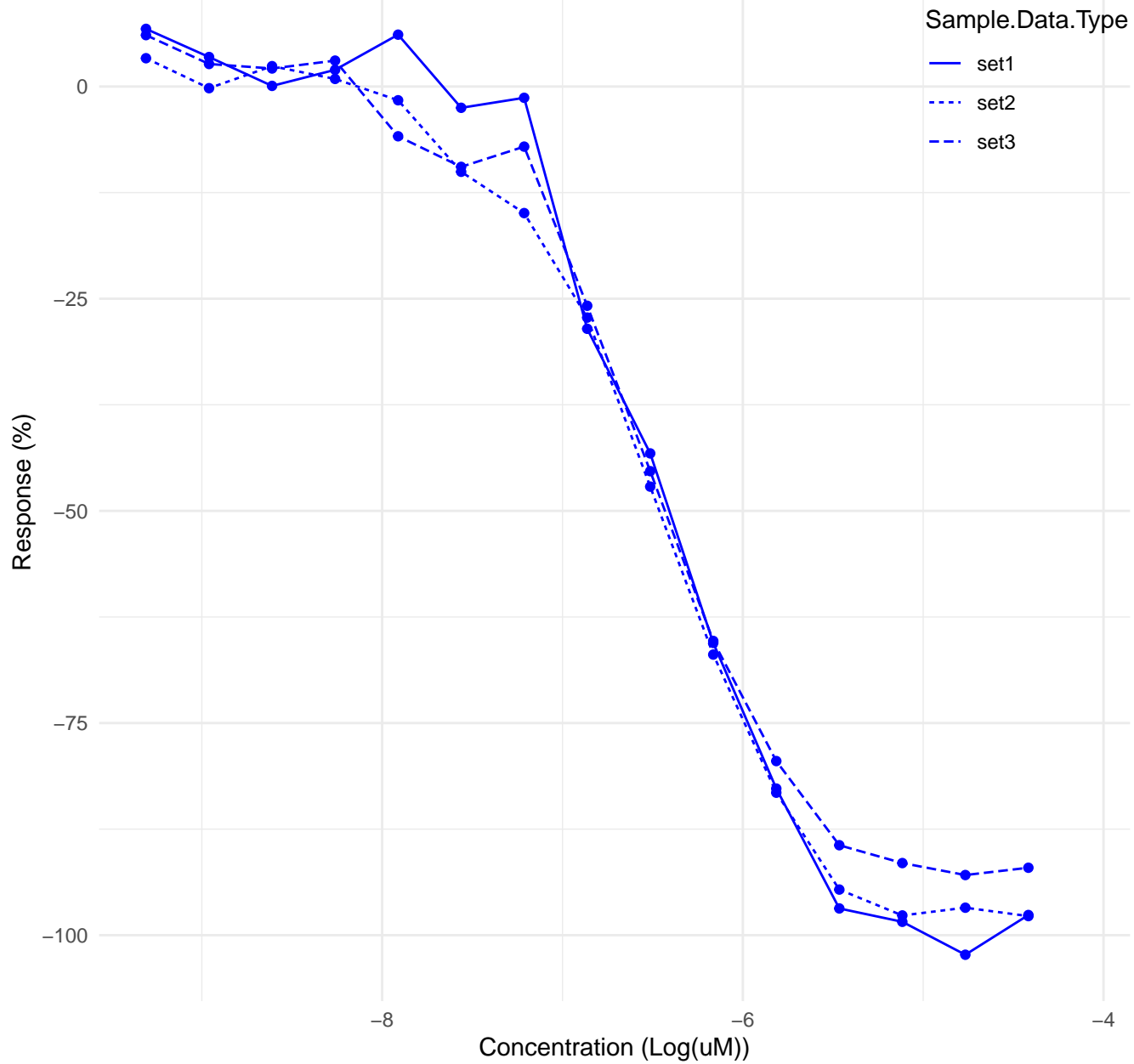

60561-17-3

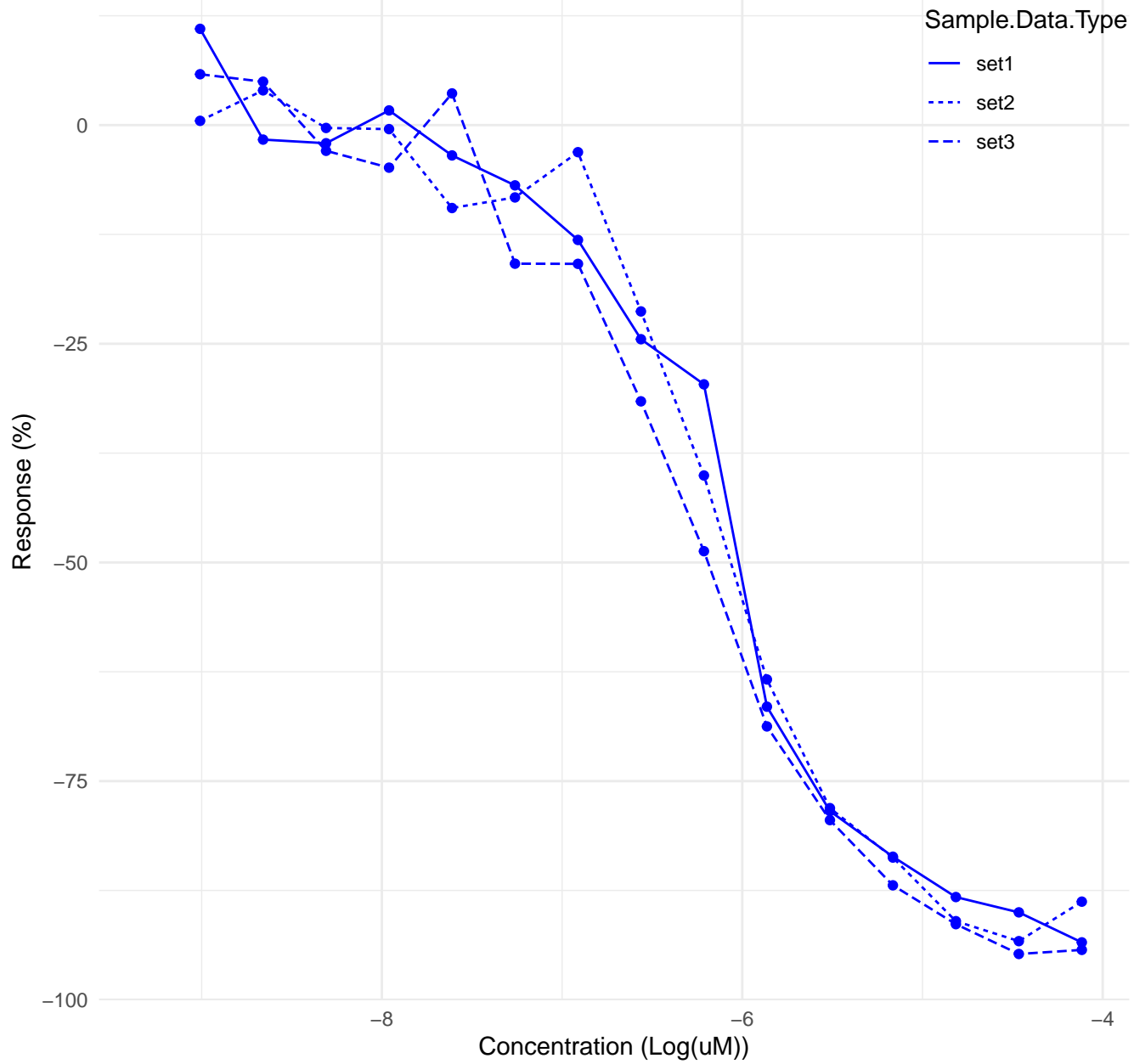

60607-34-3

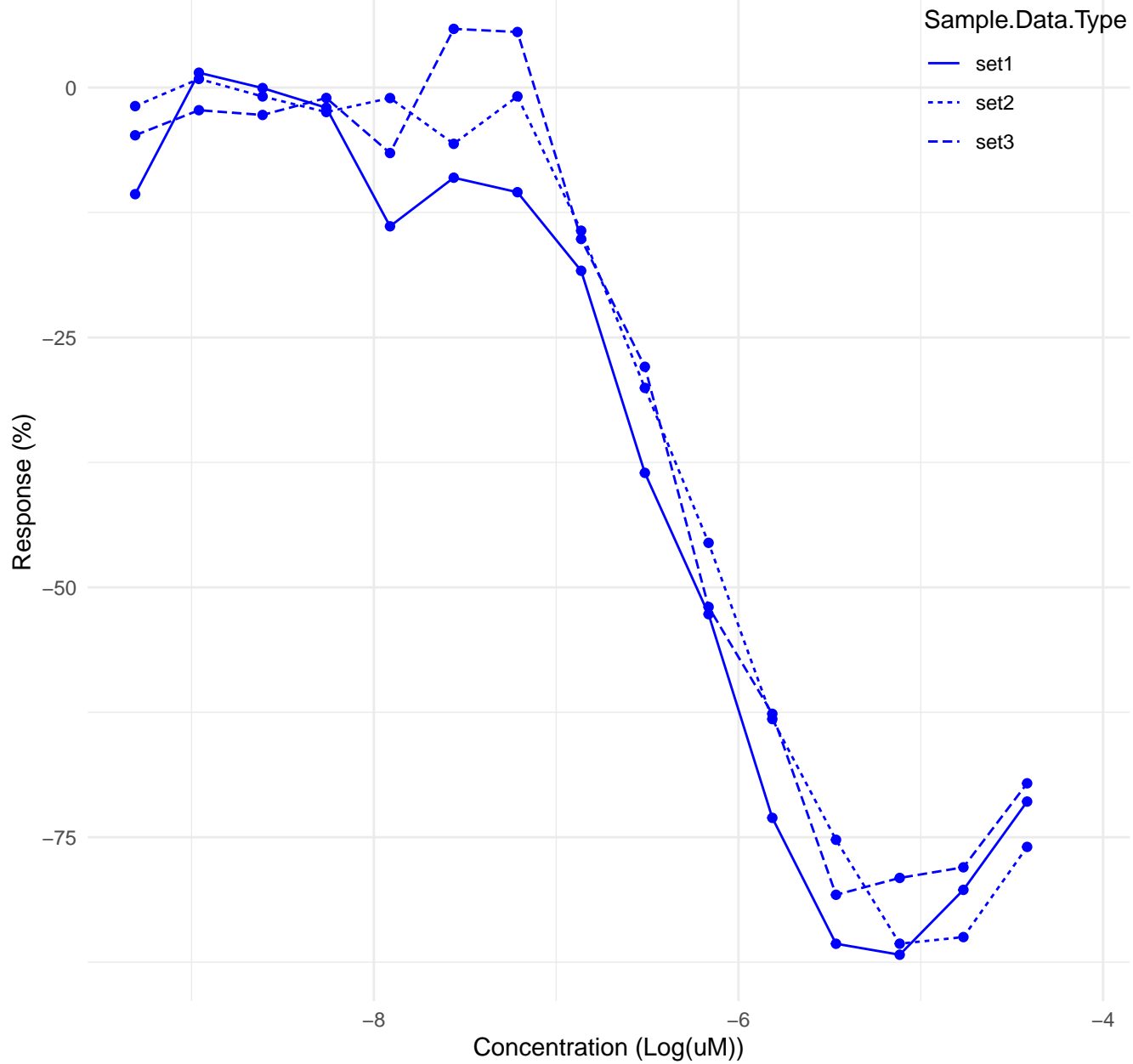

64706-54-3

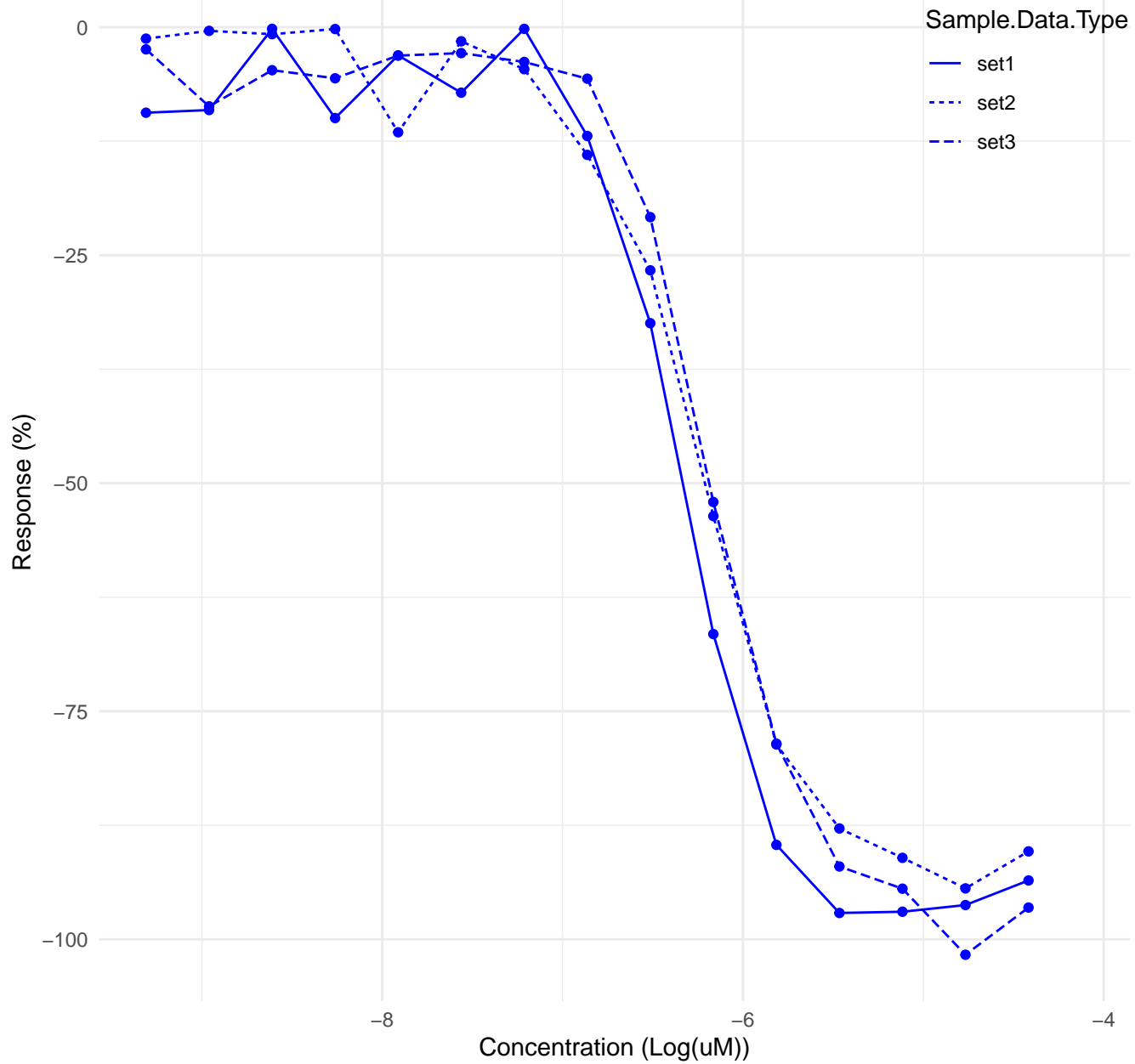

654057-97-3

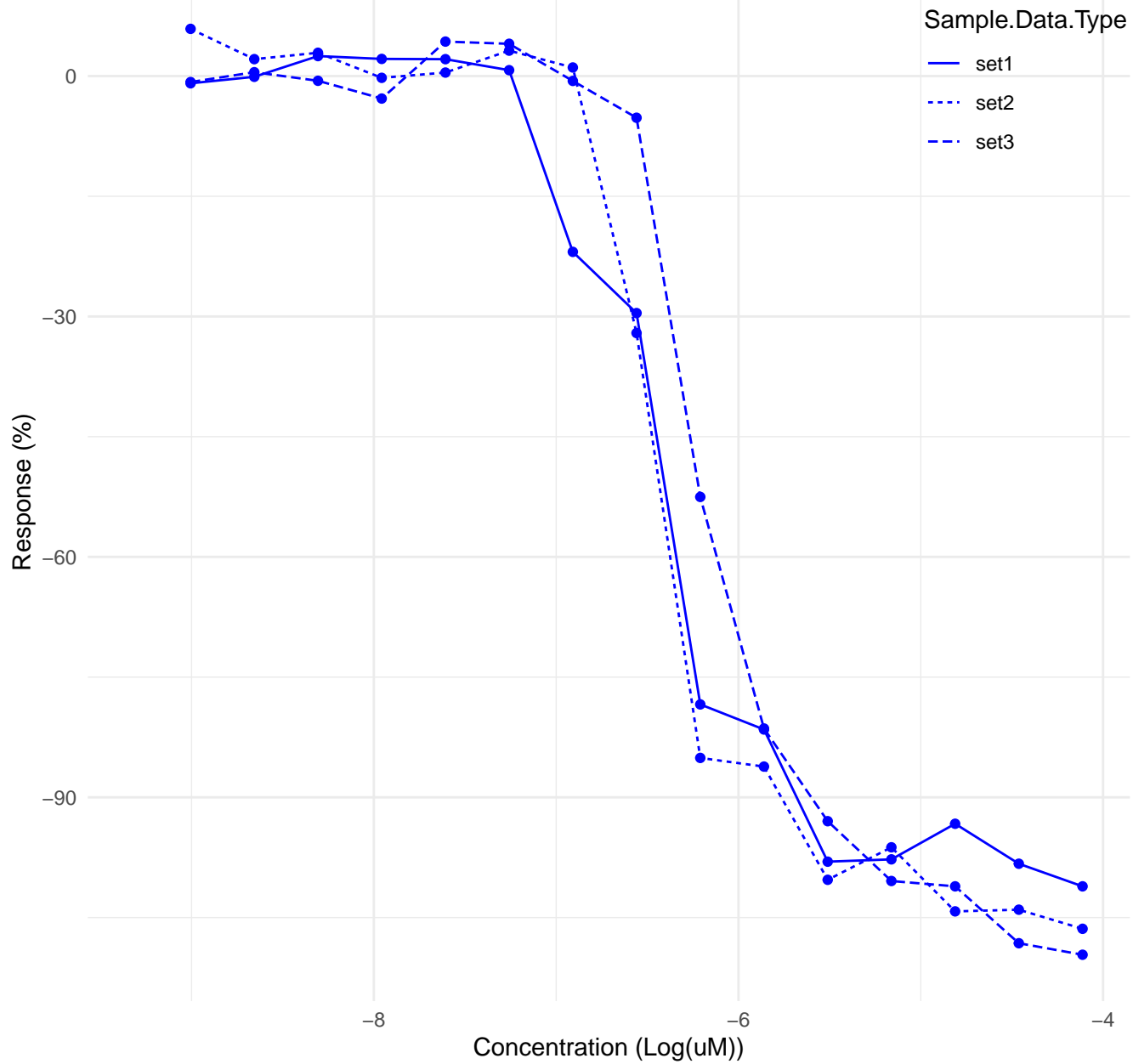

67469-78-7

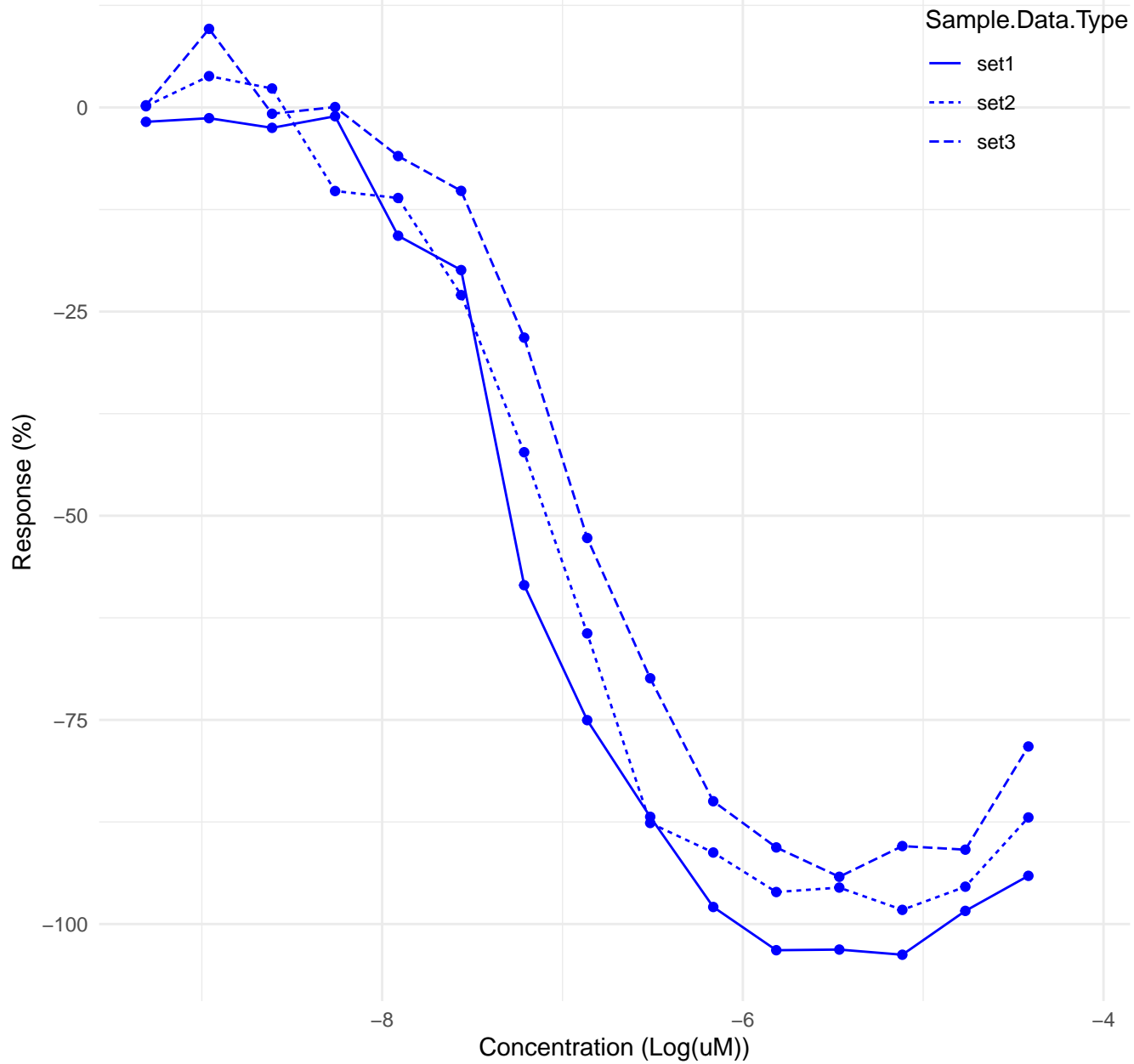

67485-29-4

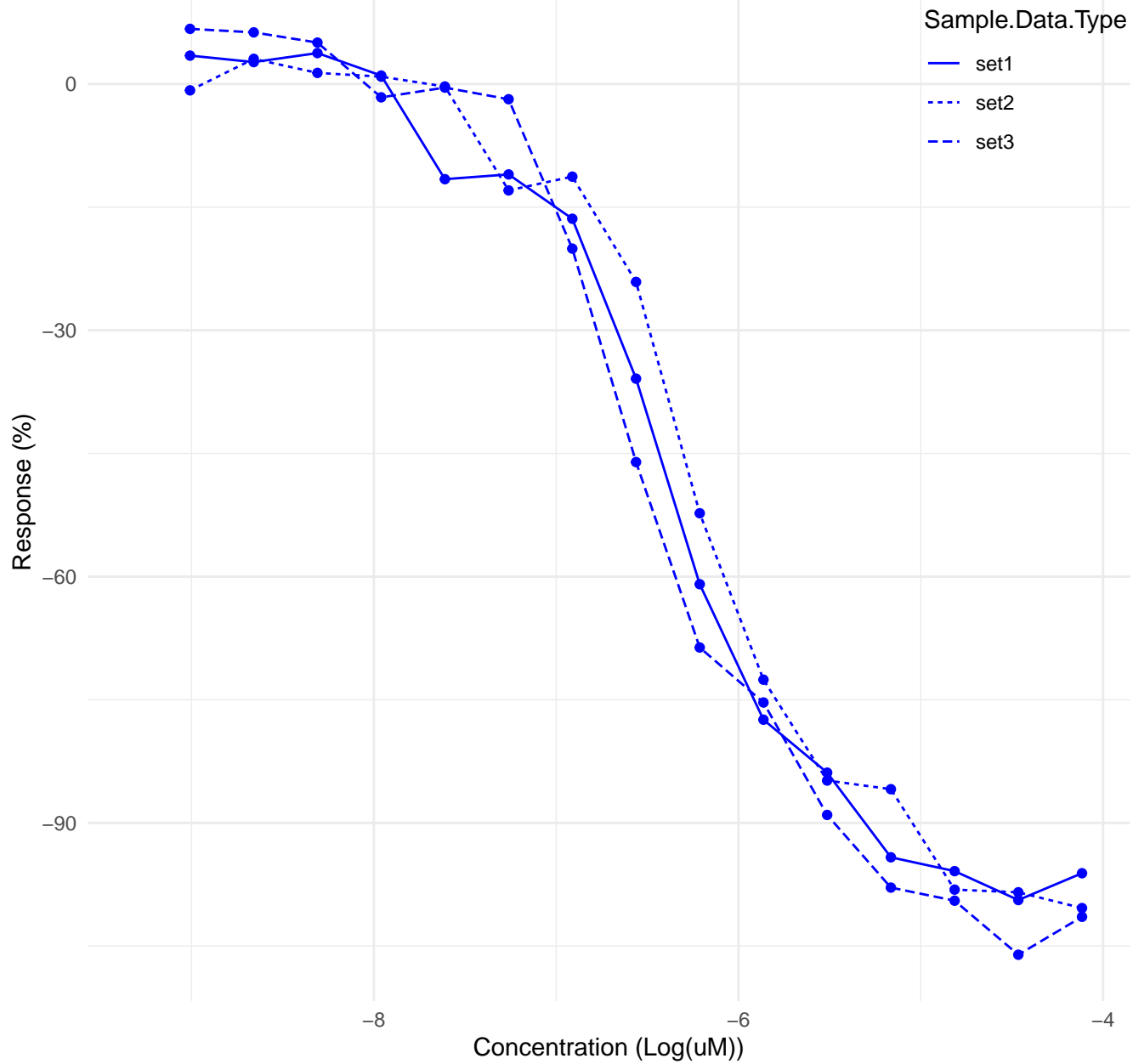

68844-77-9

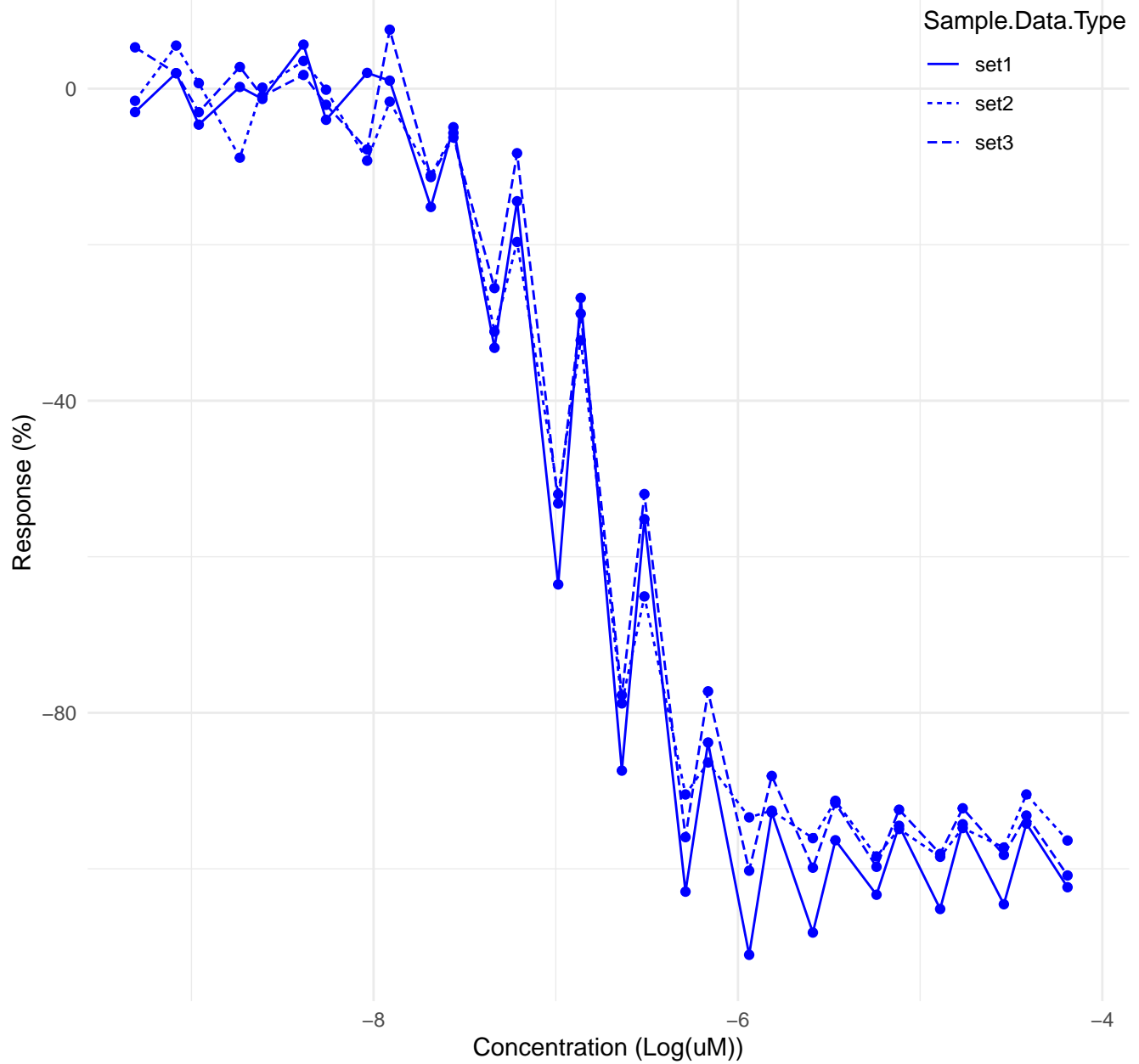

701921-71-3

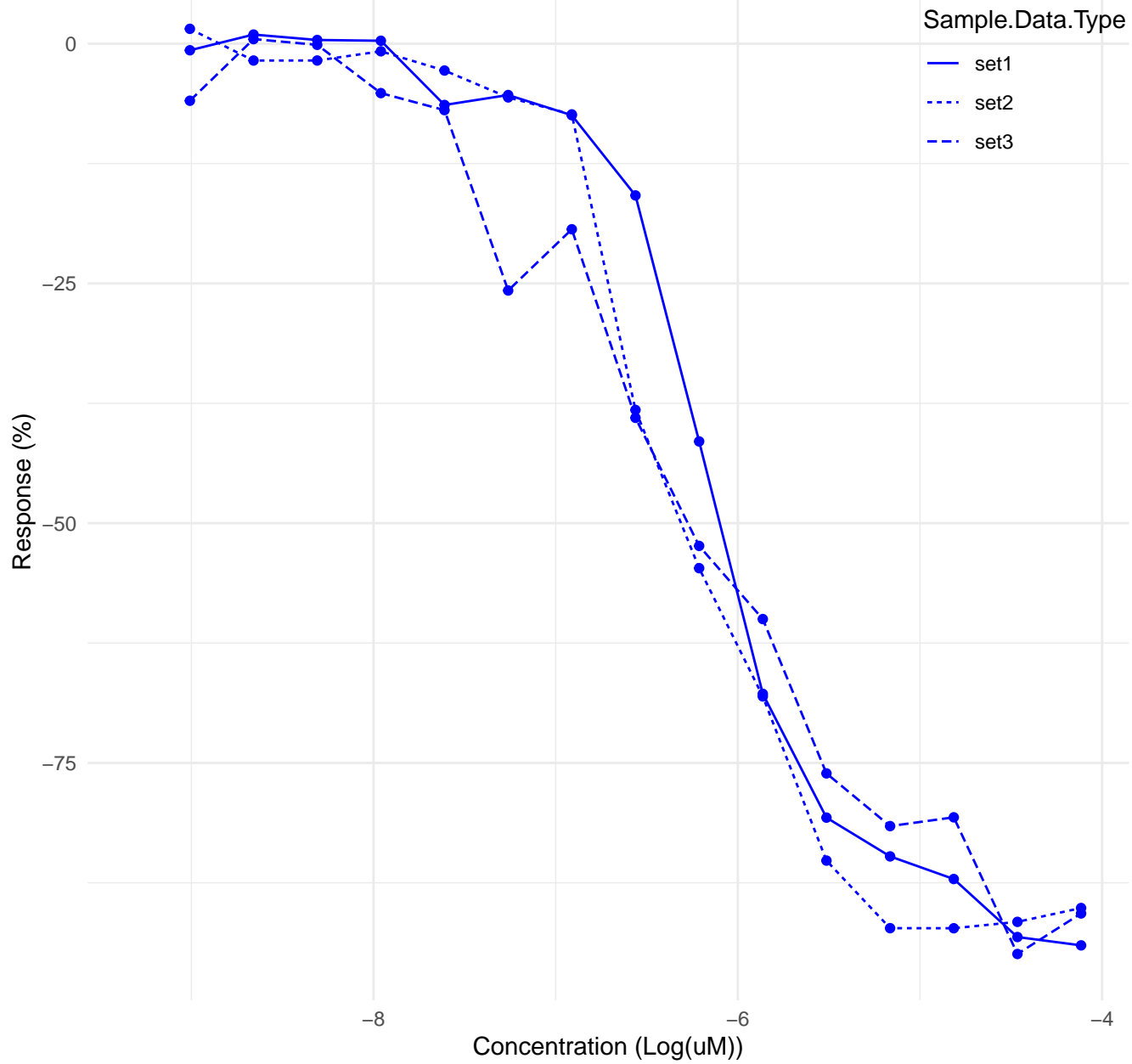

70862-65-6

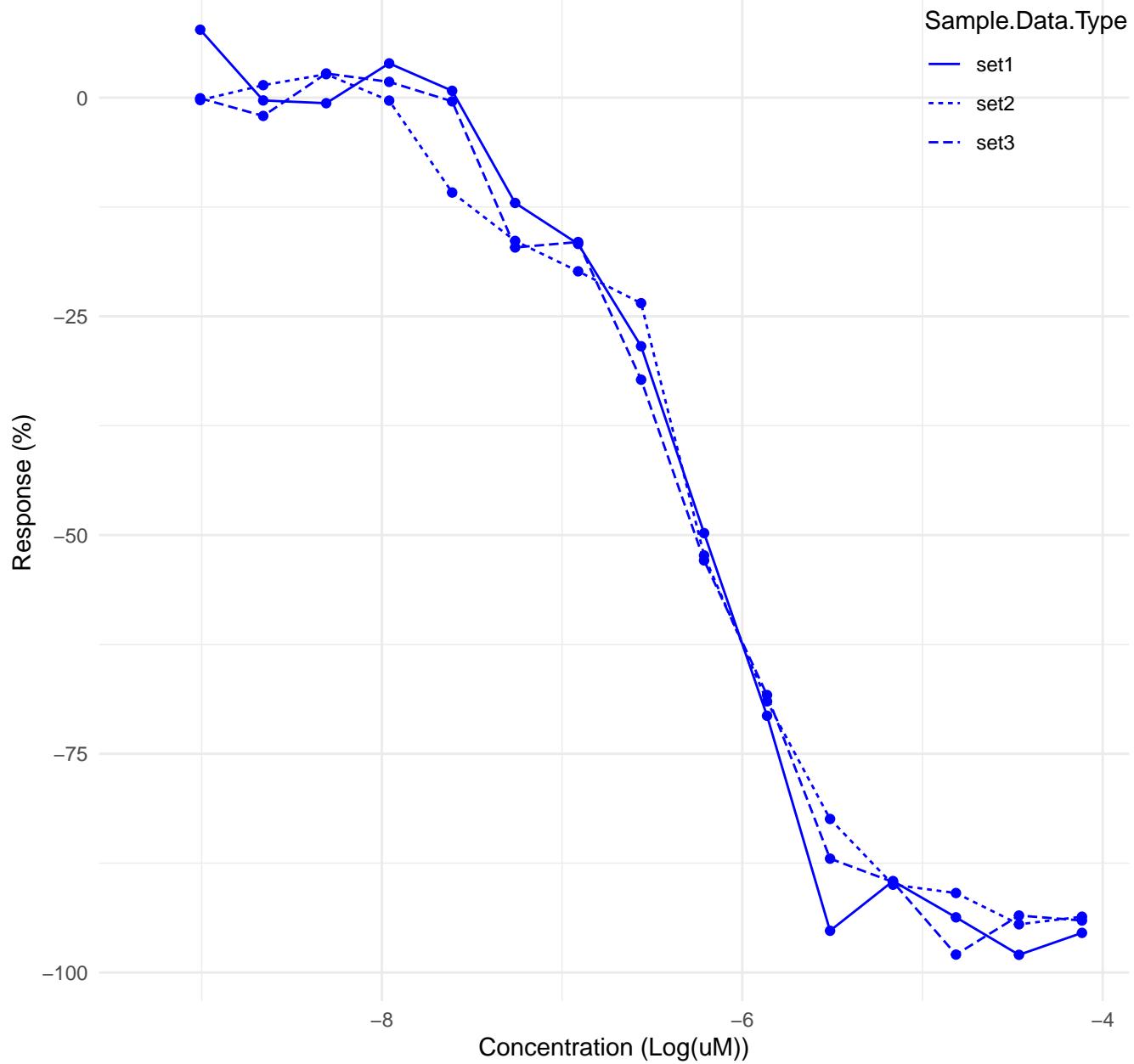

7173-51-5

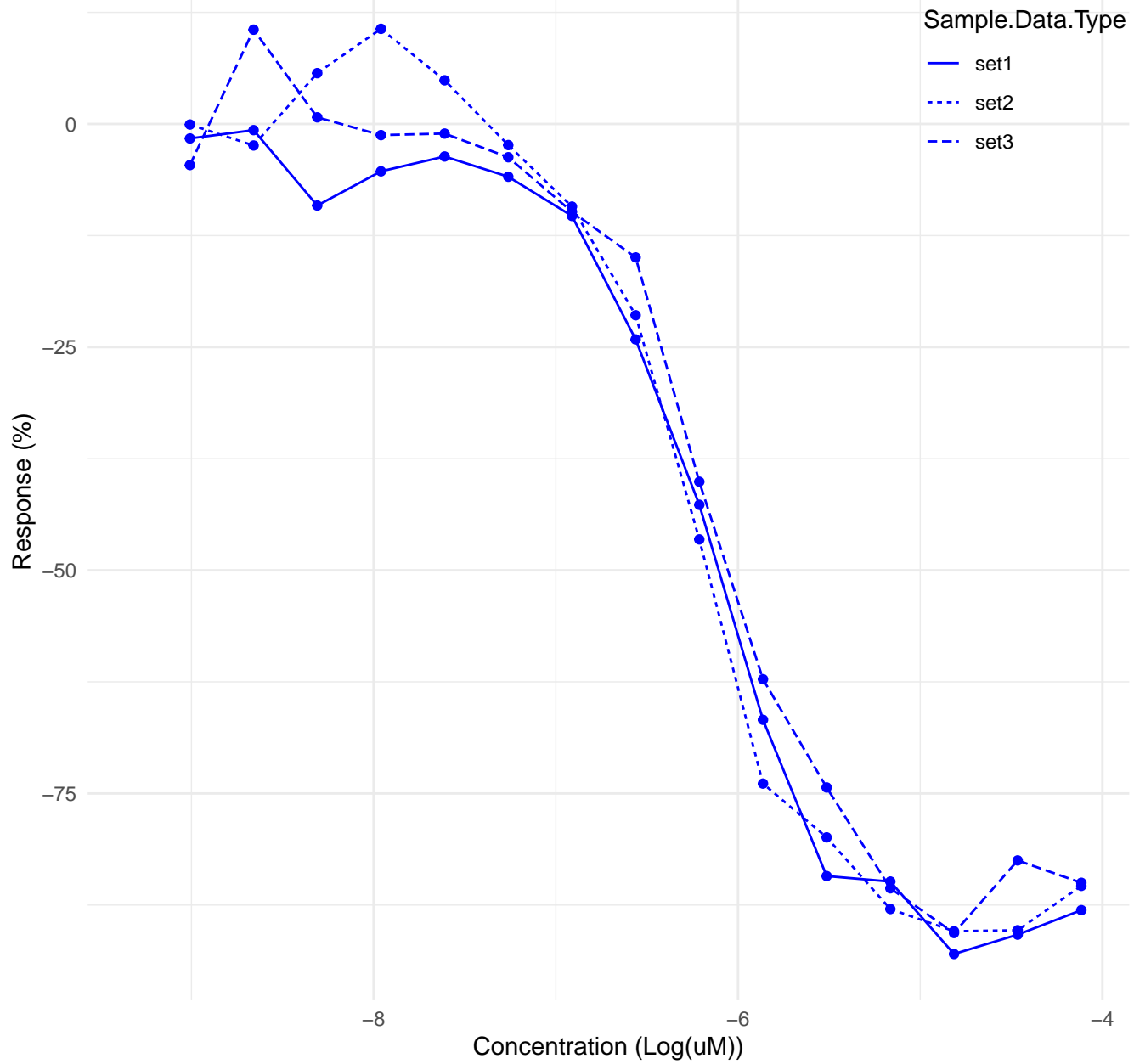

75529-73-6

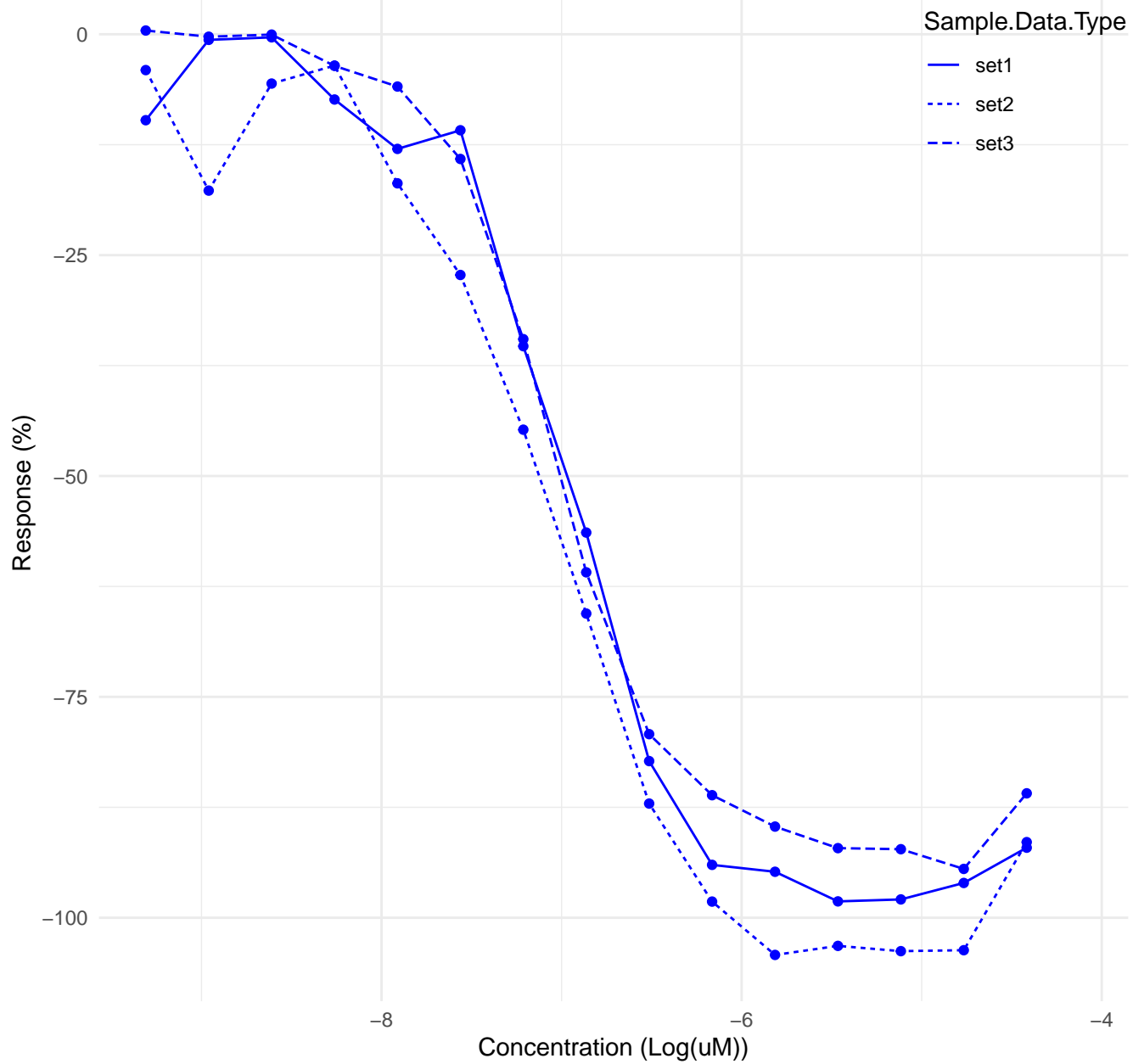

79307-93-0

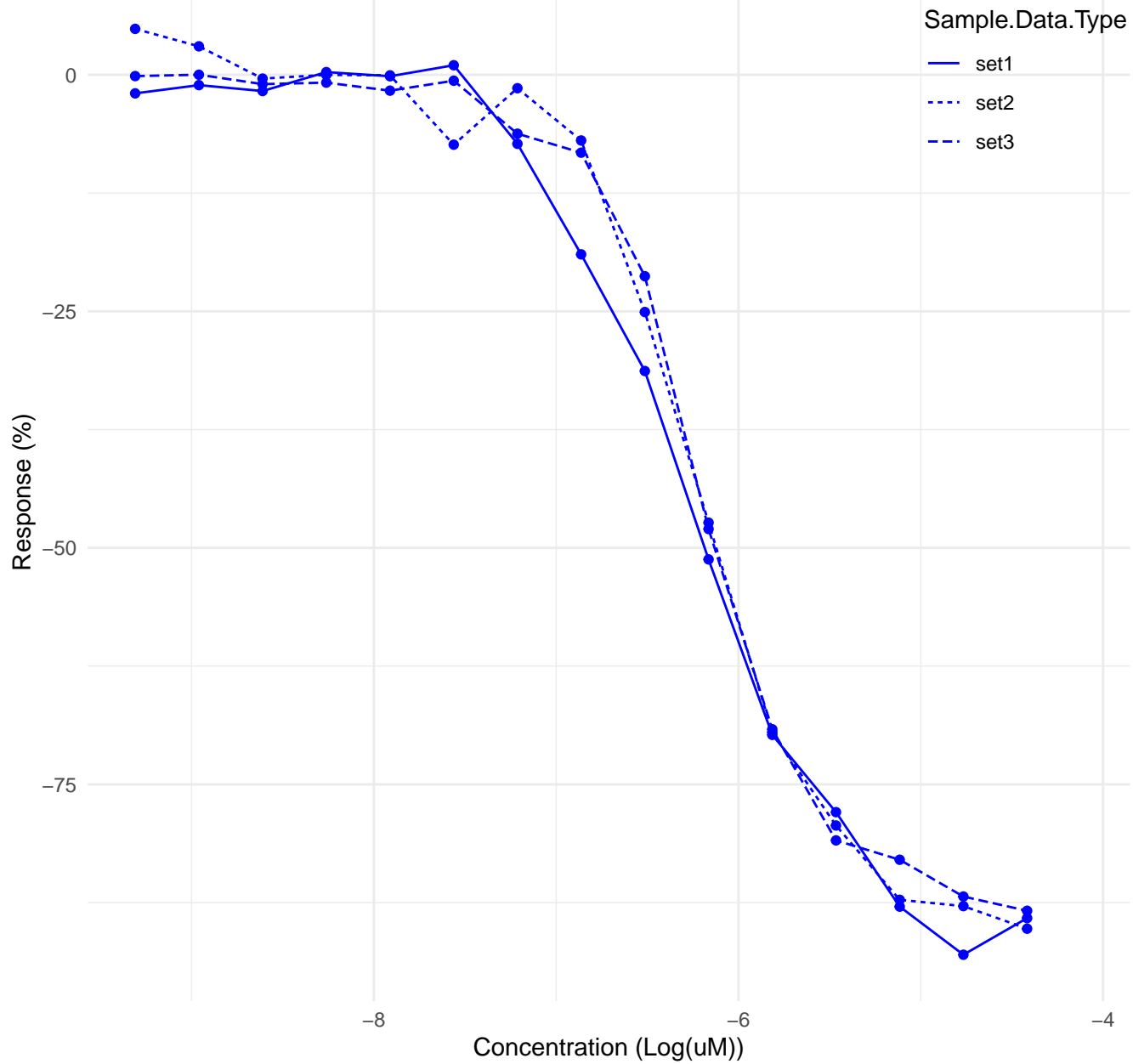

81098-60-4

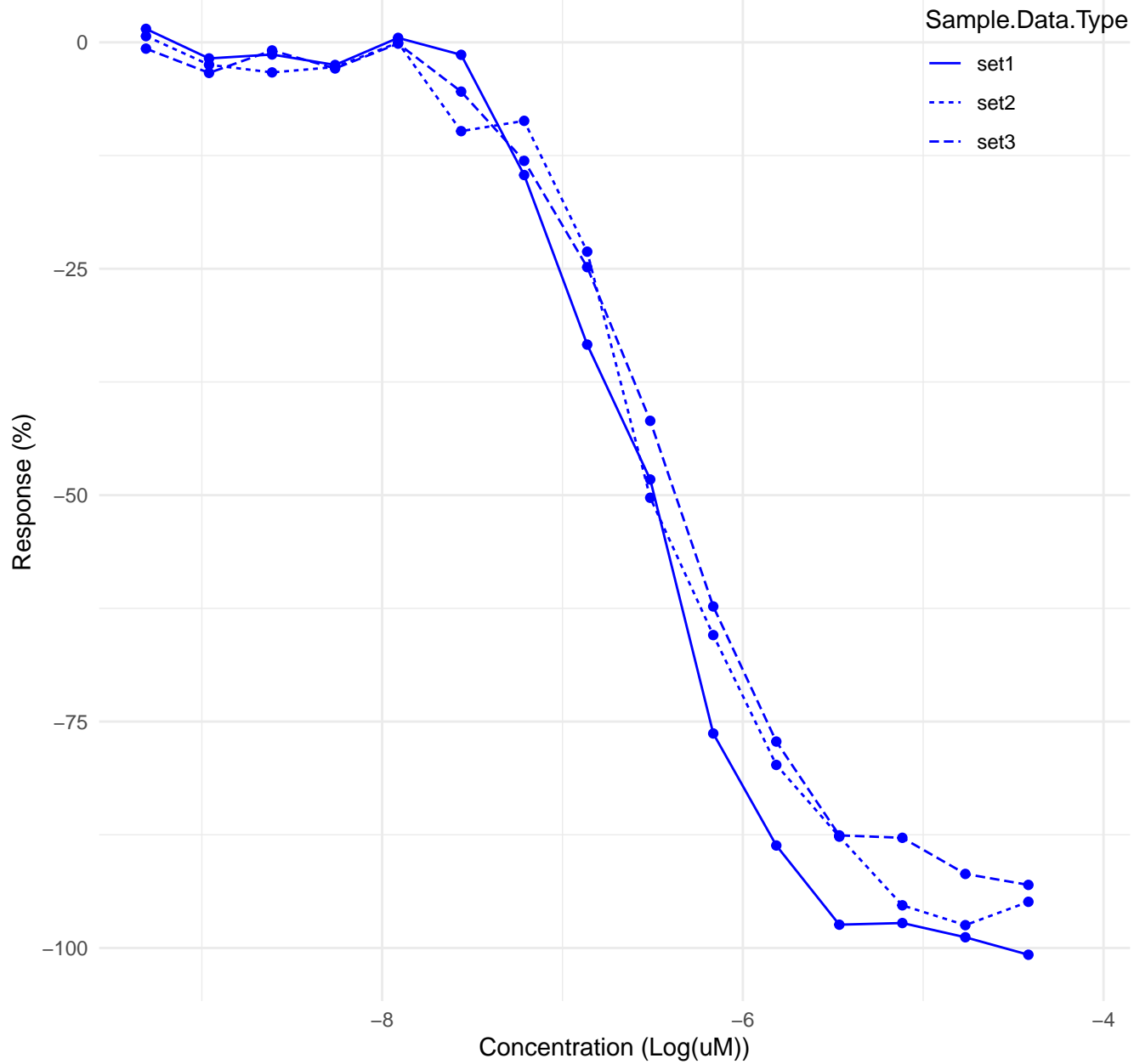

81741-28-8

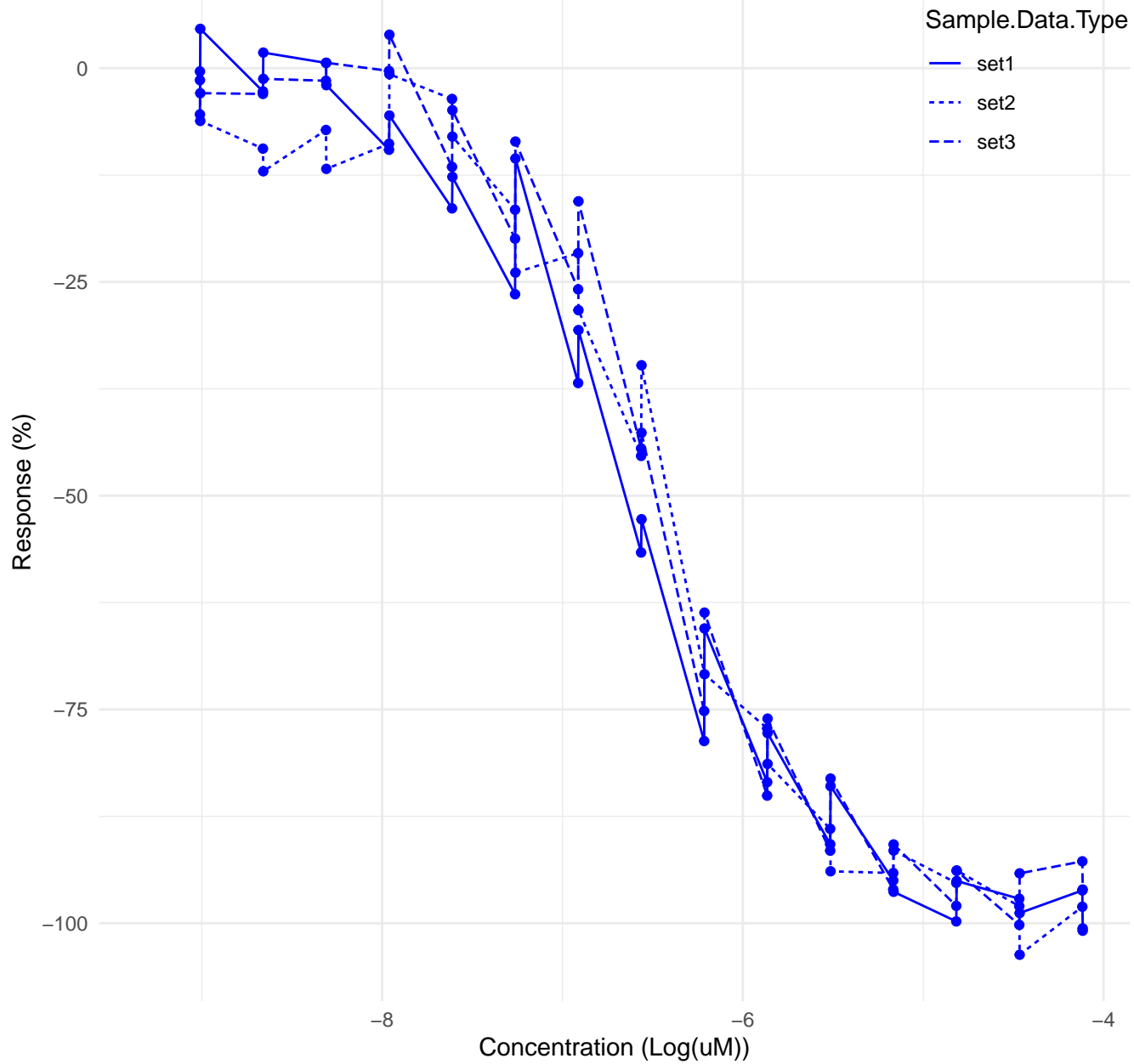

87051-43-2

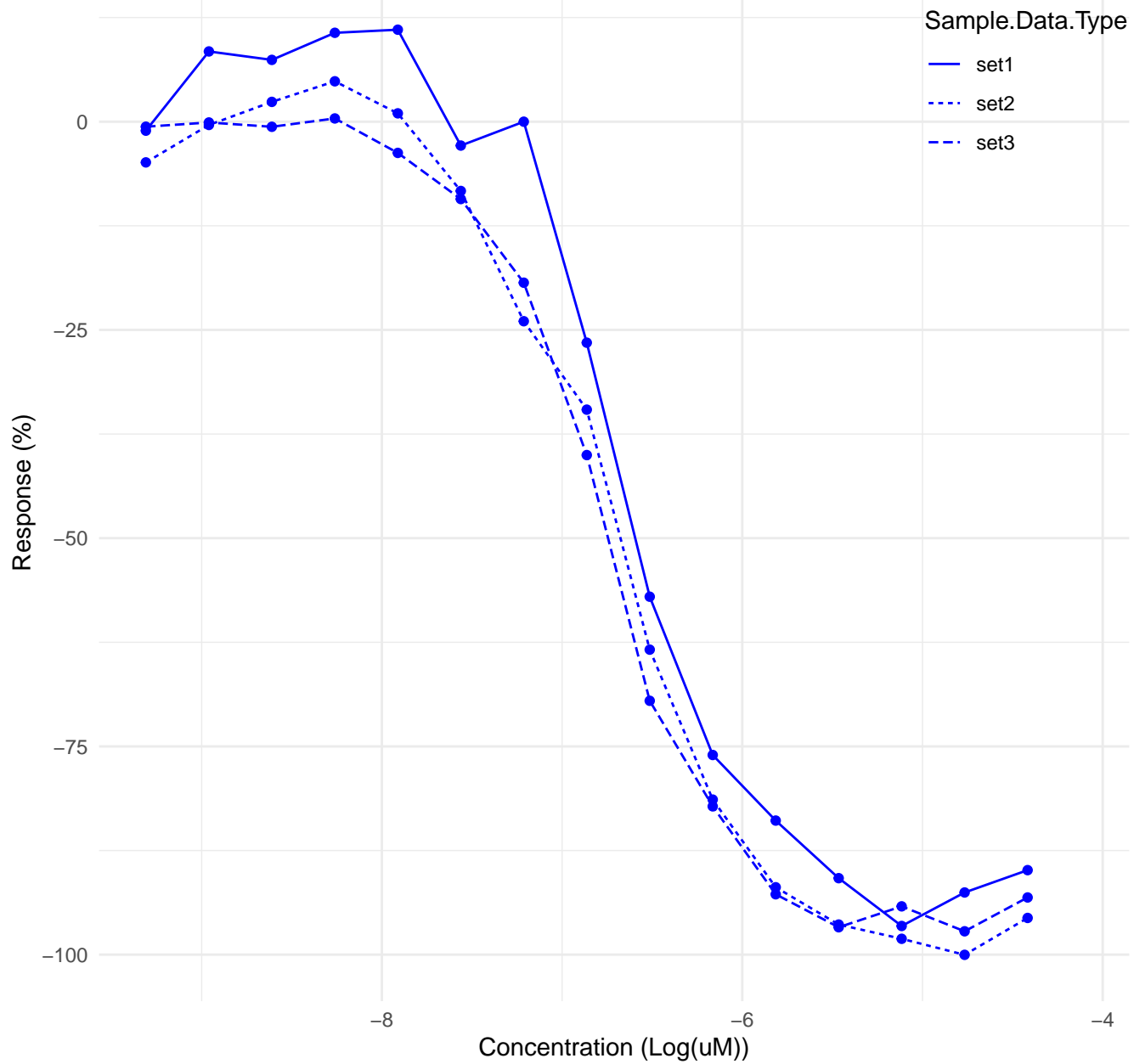

89419-40-9

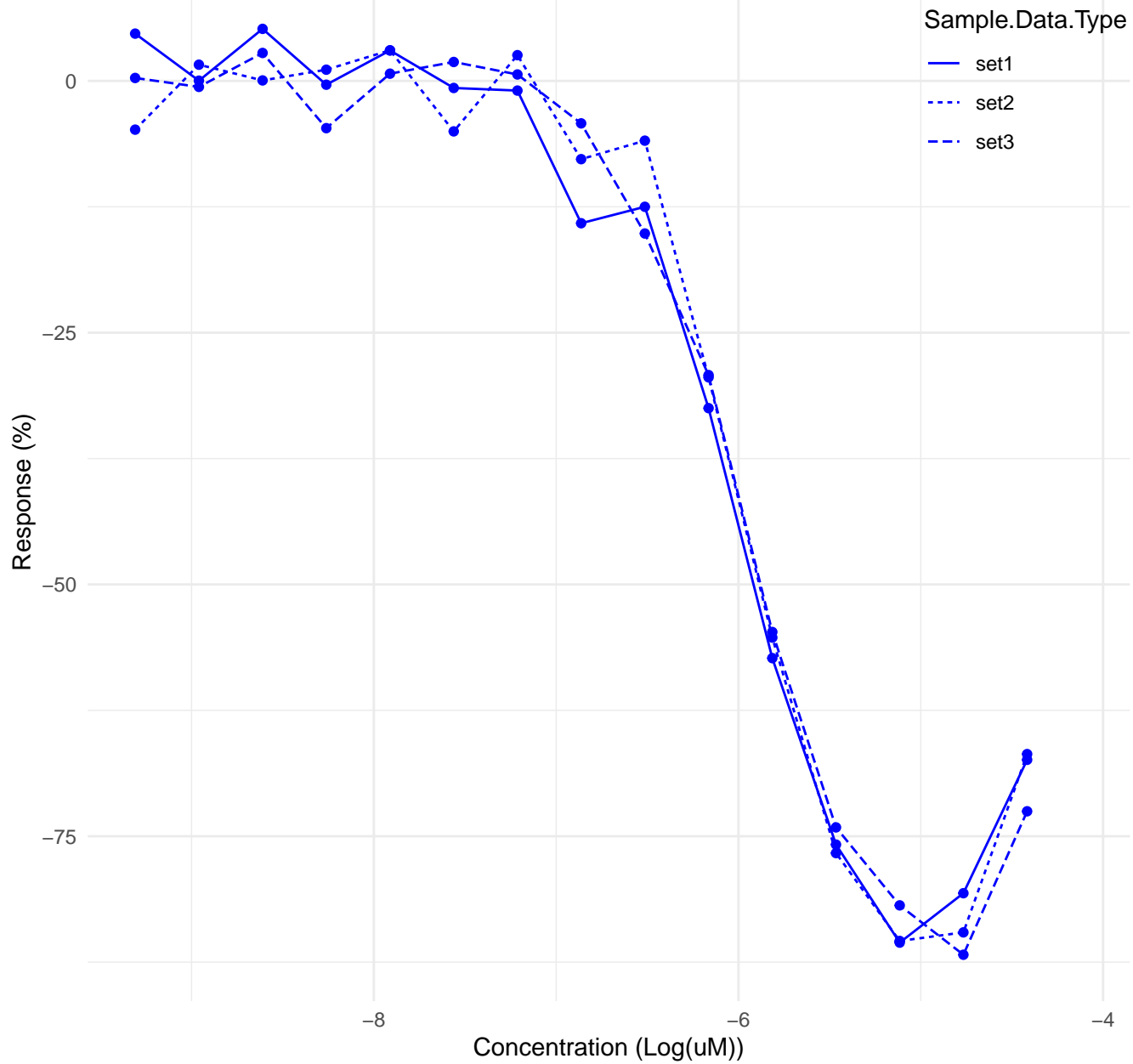

90729-43-4

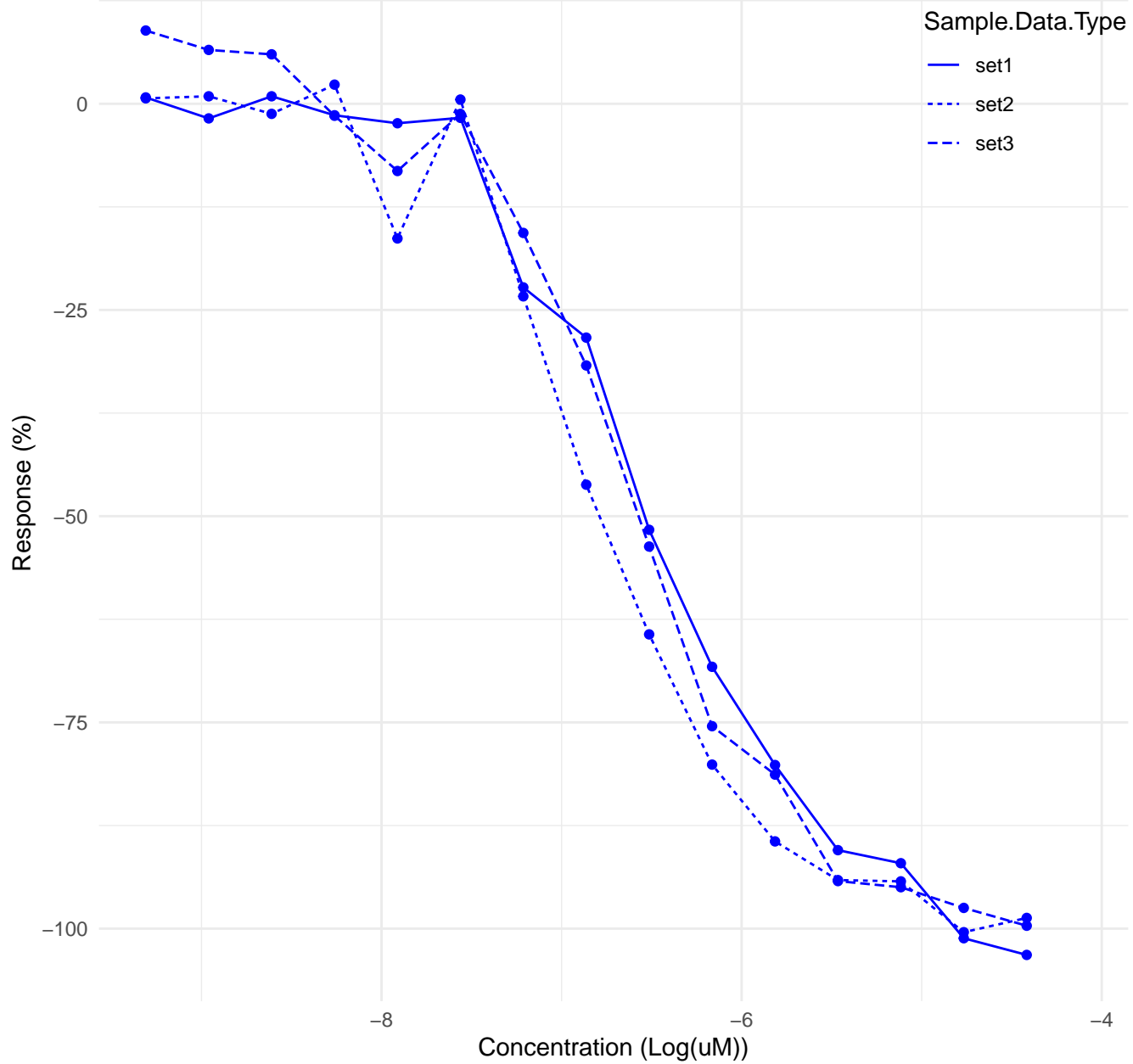

92953-10-1

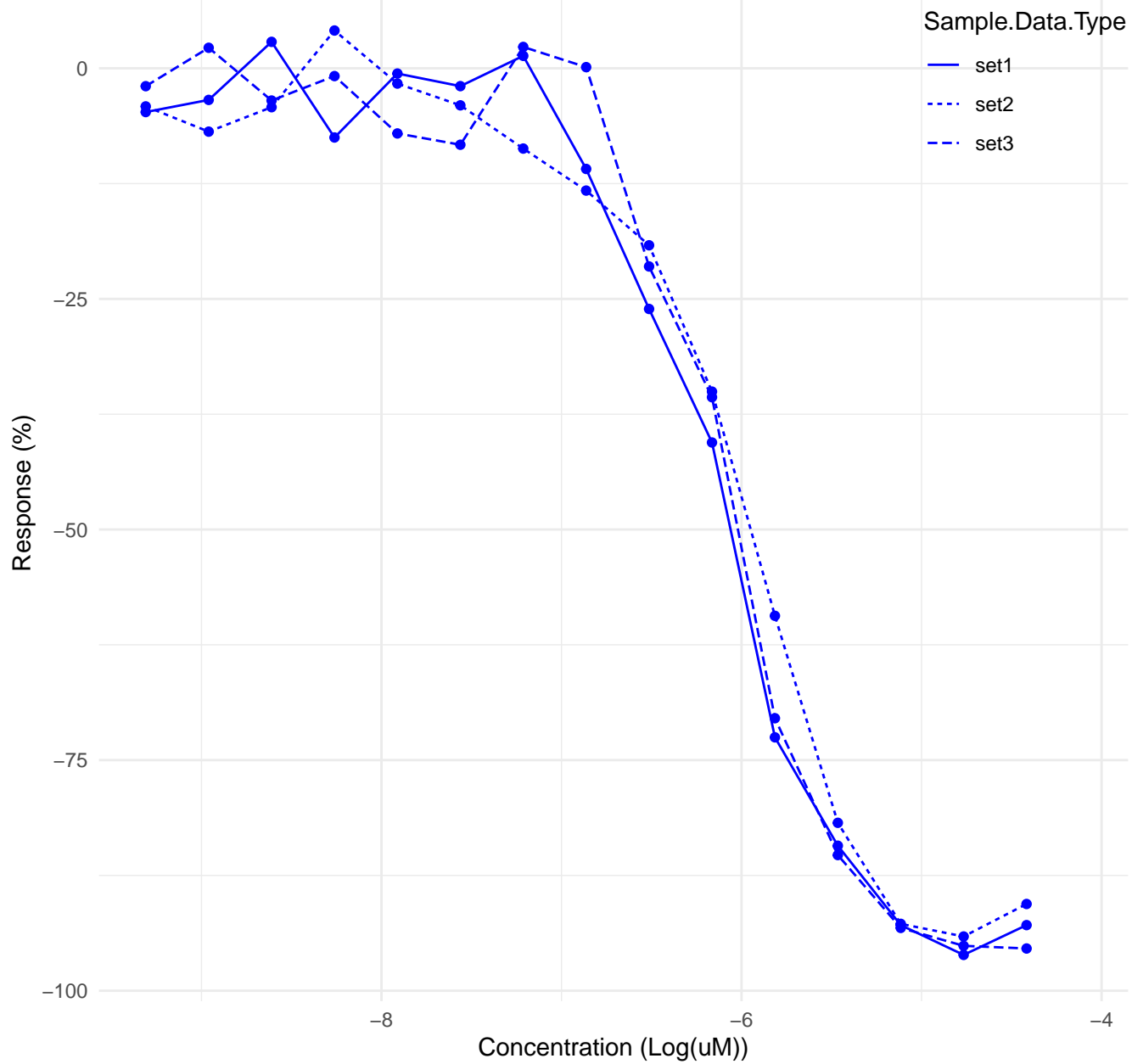

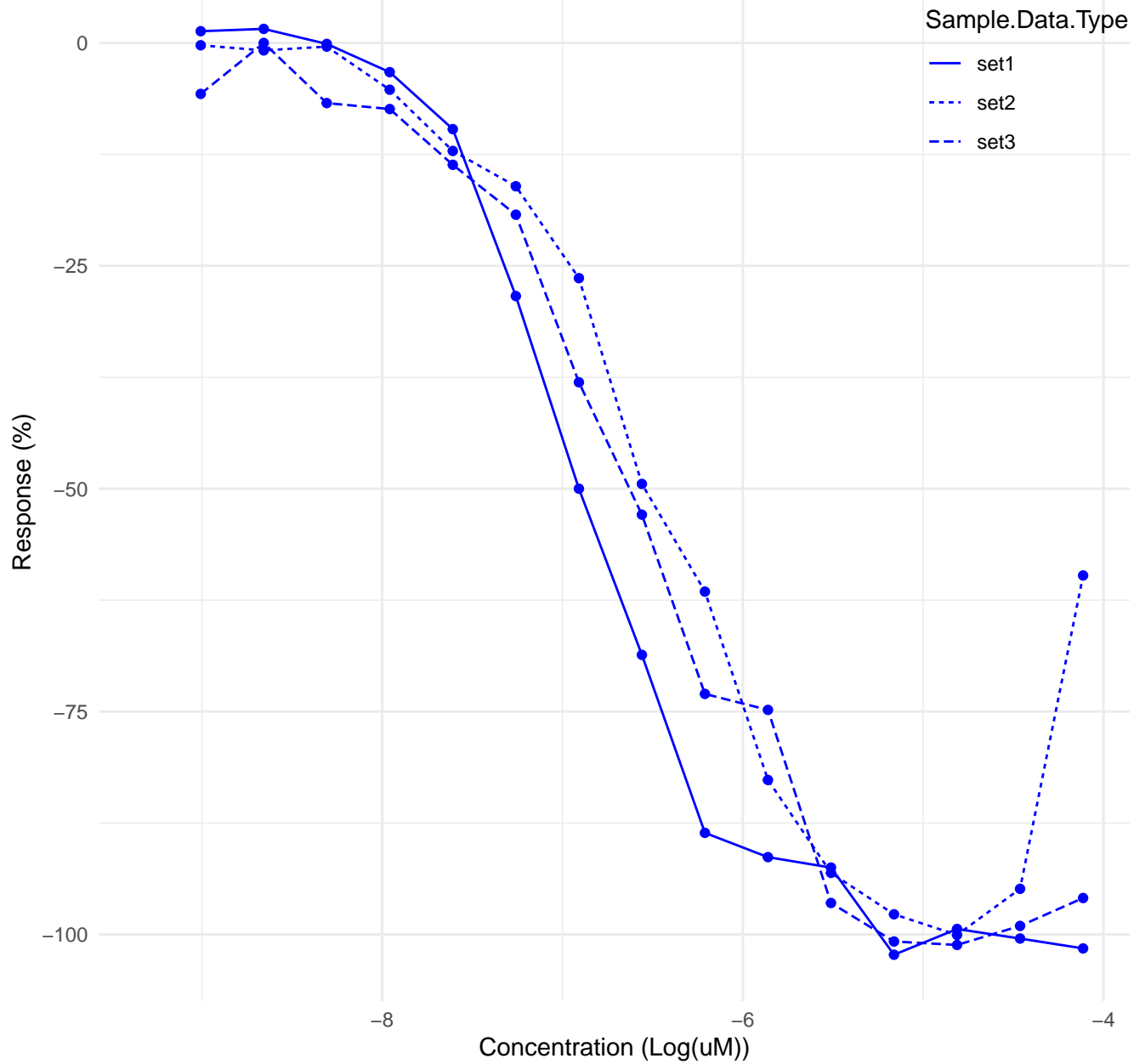

219947-96-3

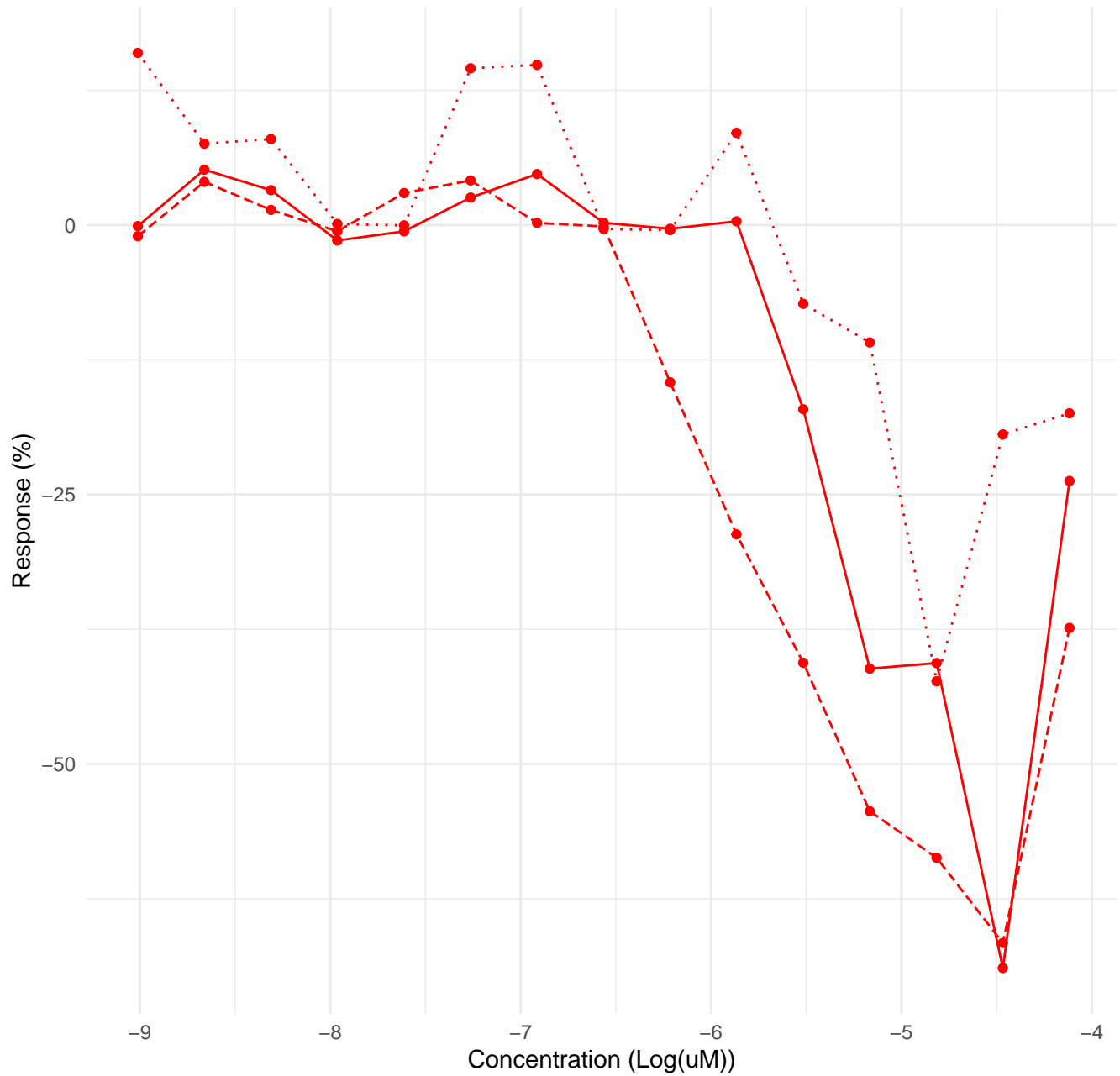

2321-07-5

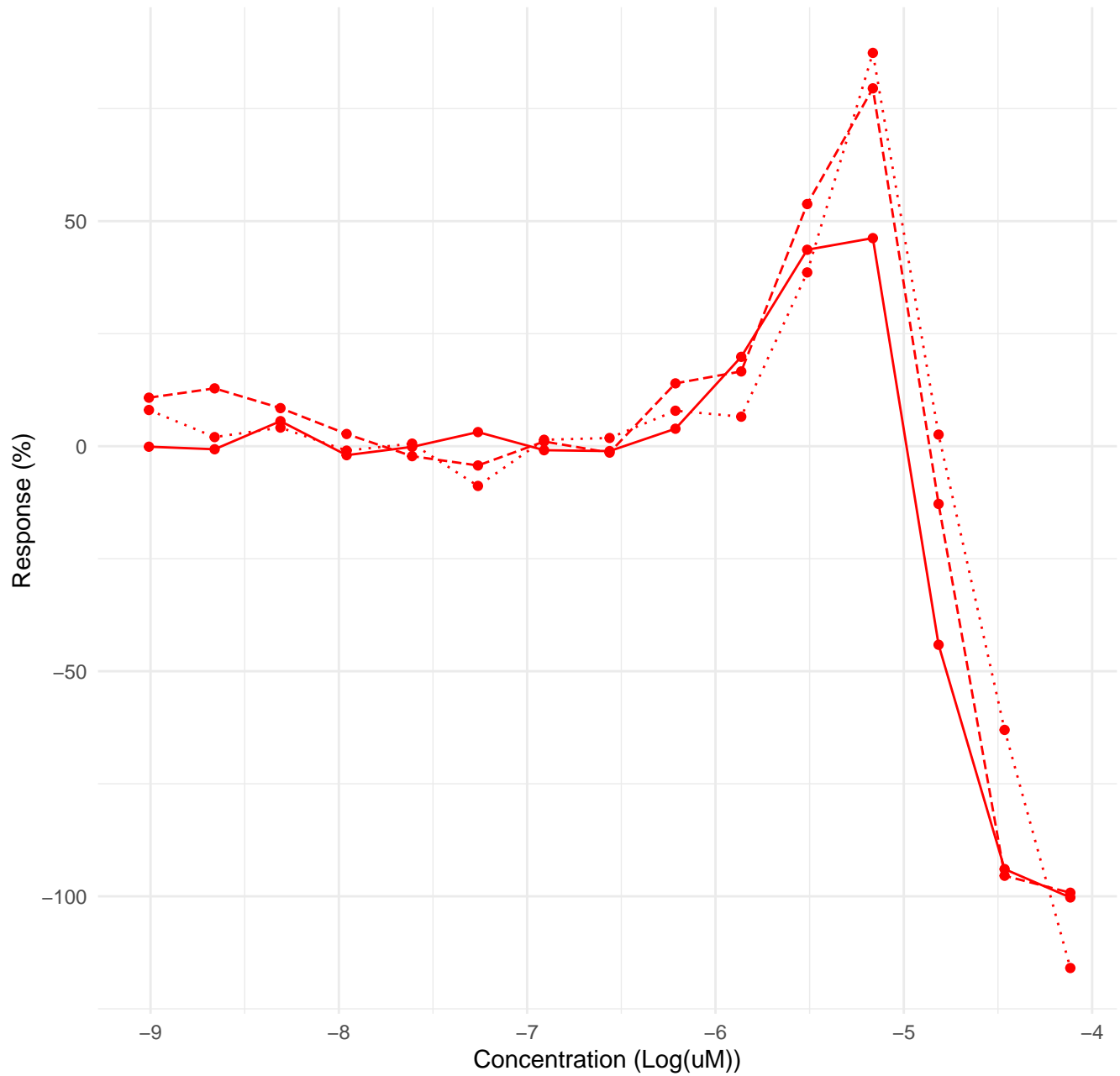

29761-21-5

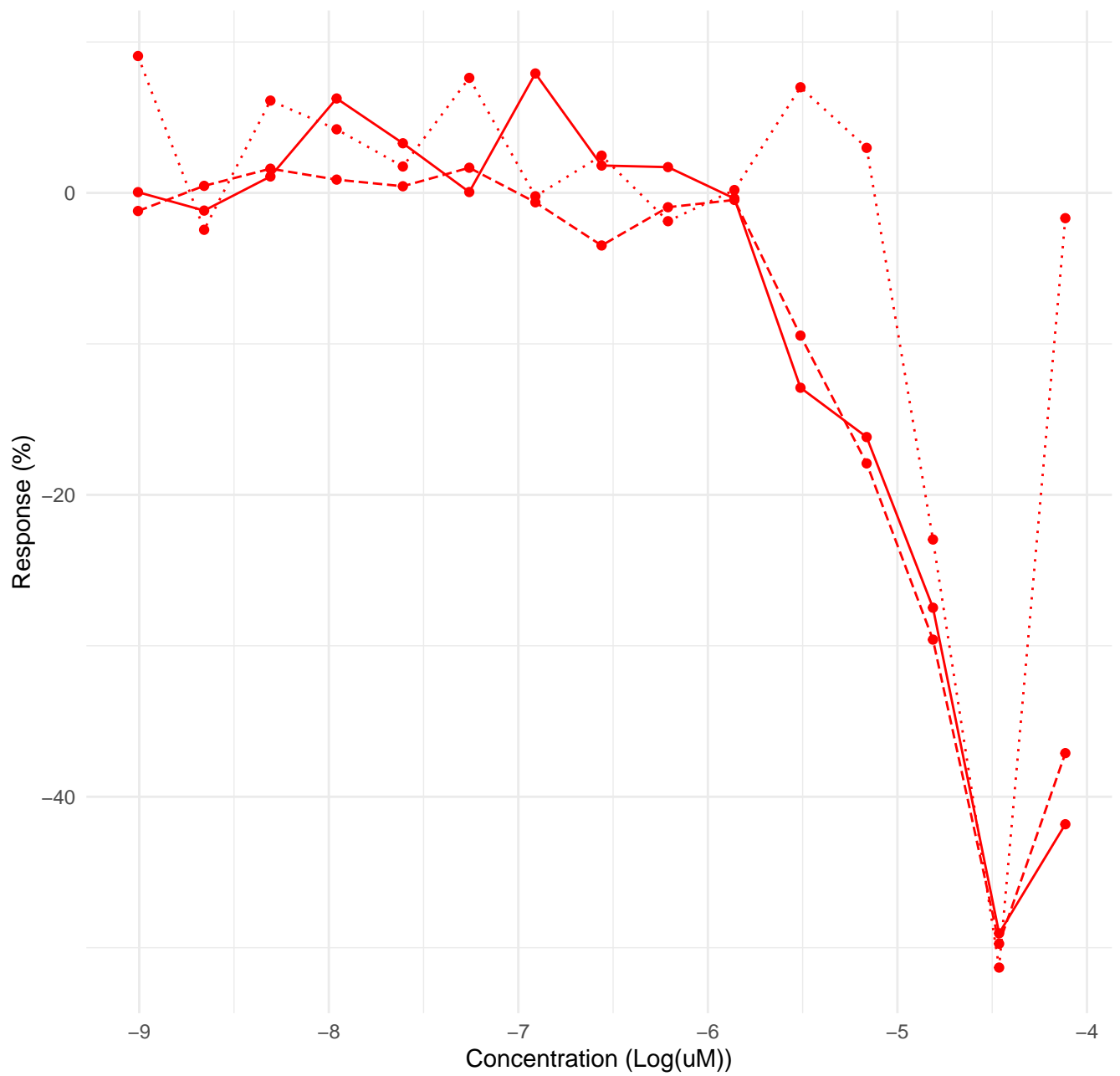

594-30-9

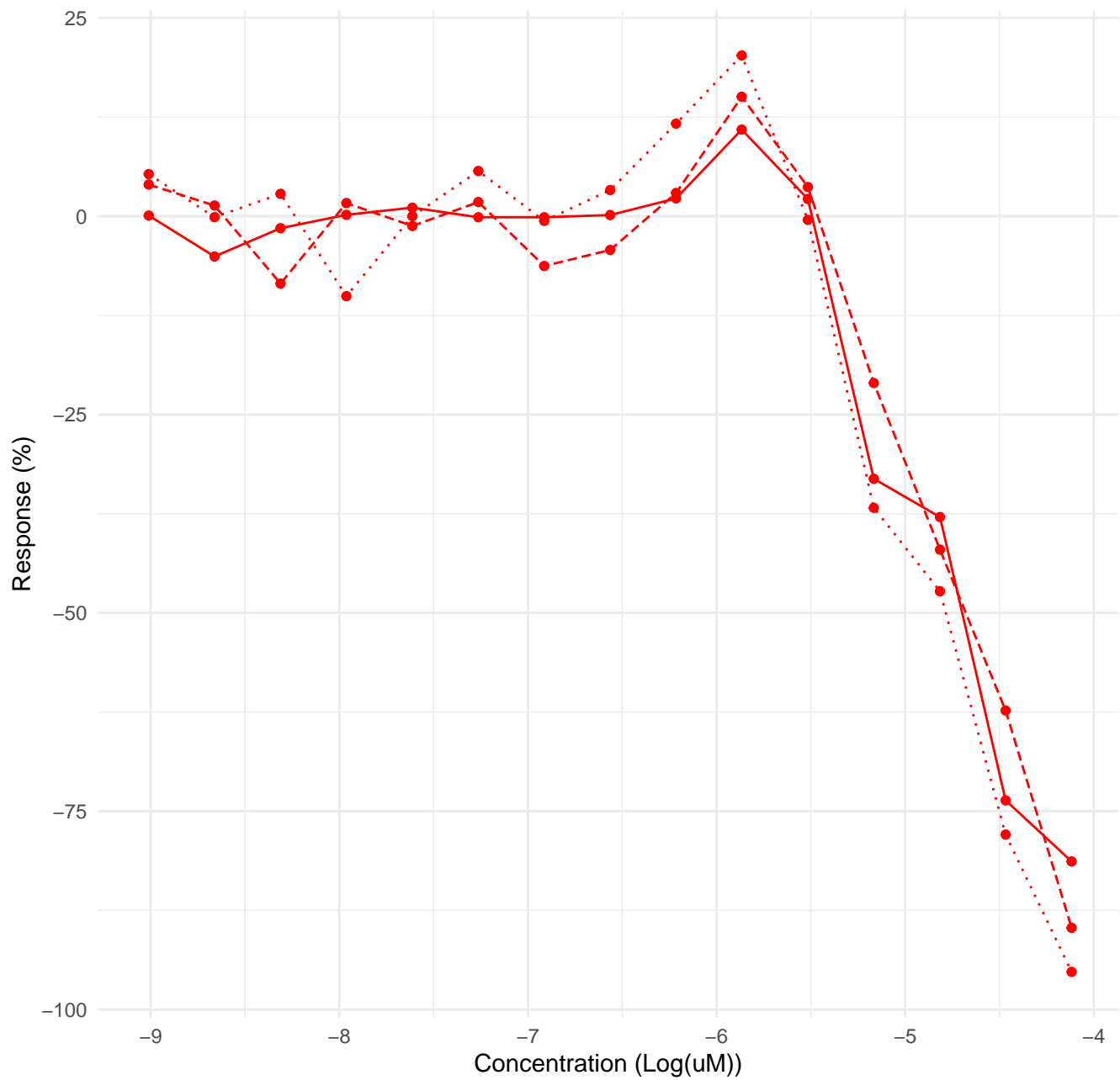

596-03-2

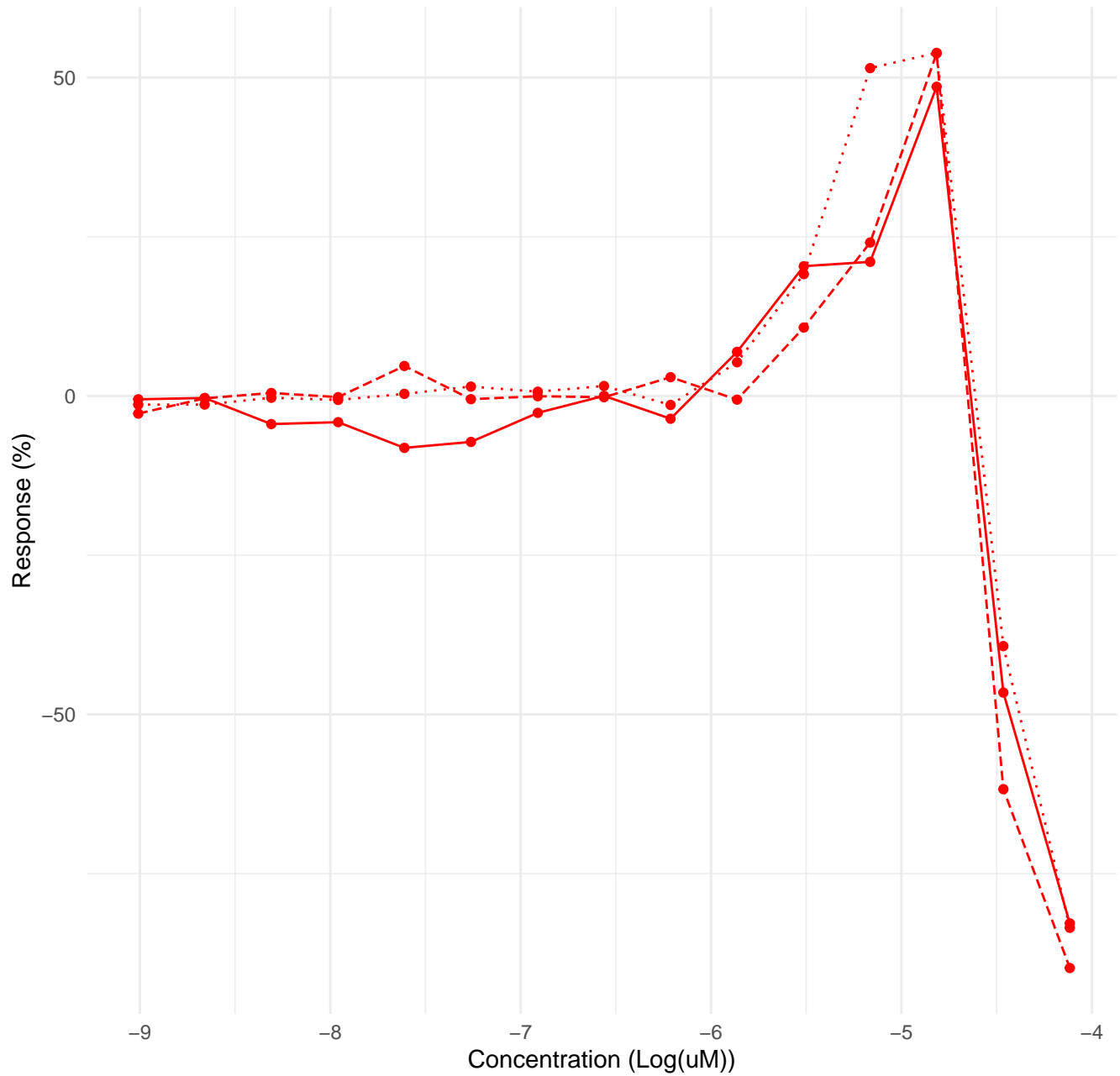

900-95-8

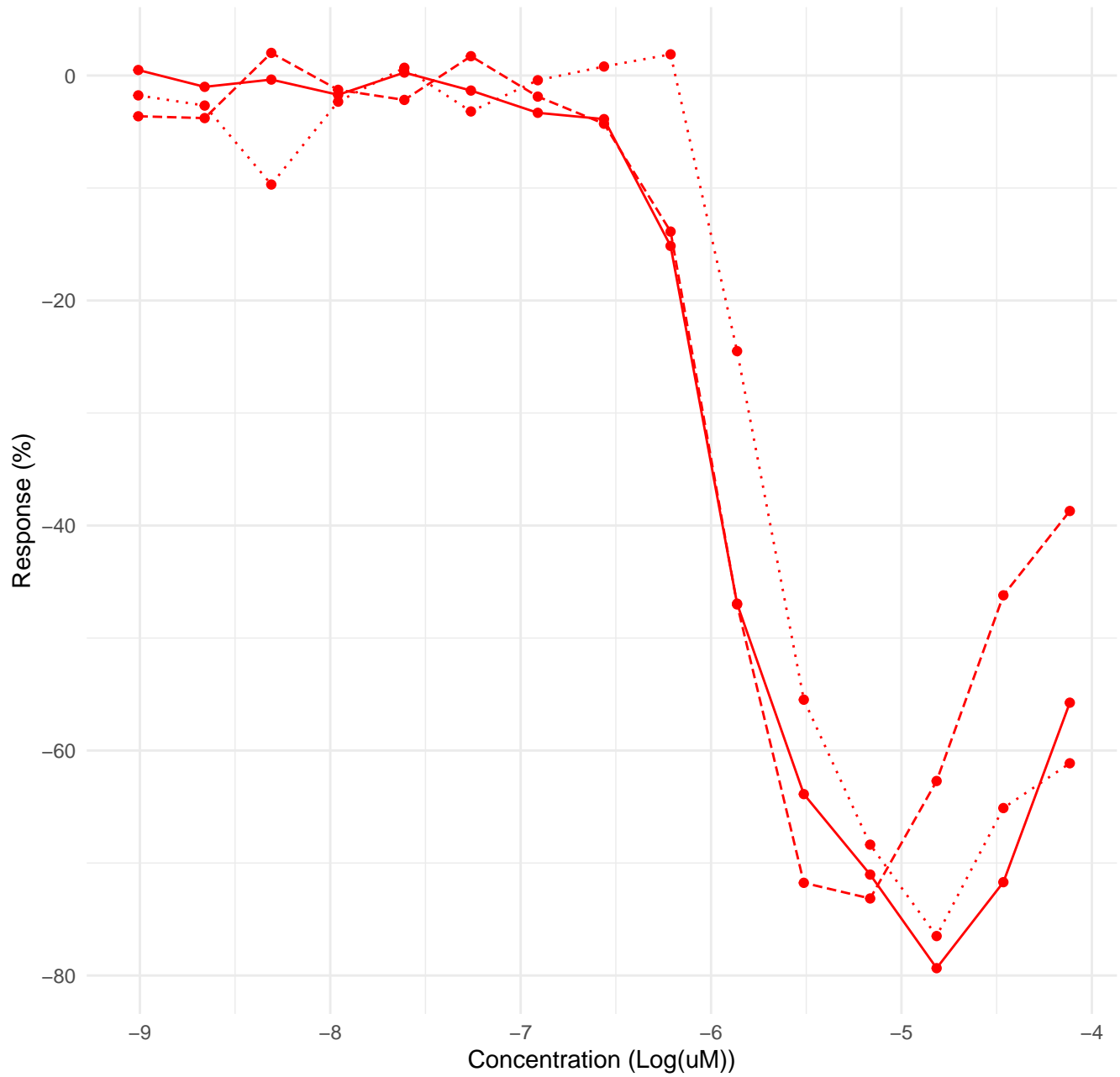

989-38-8

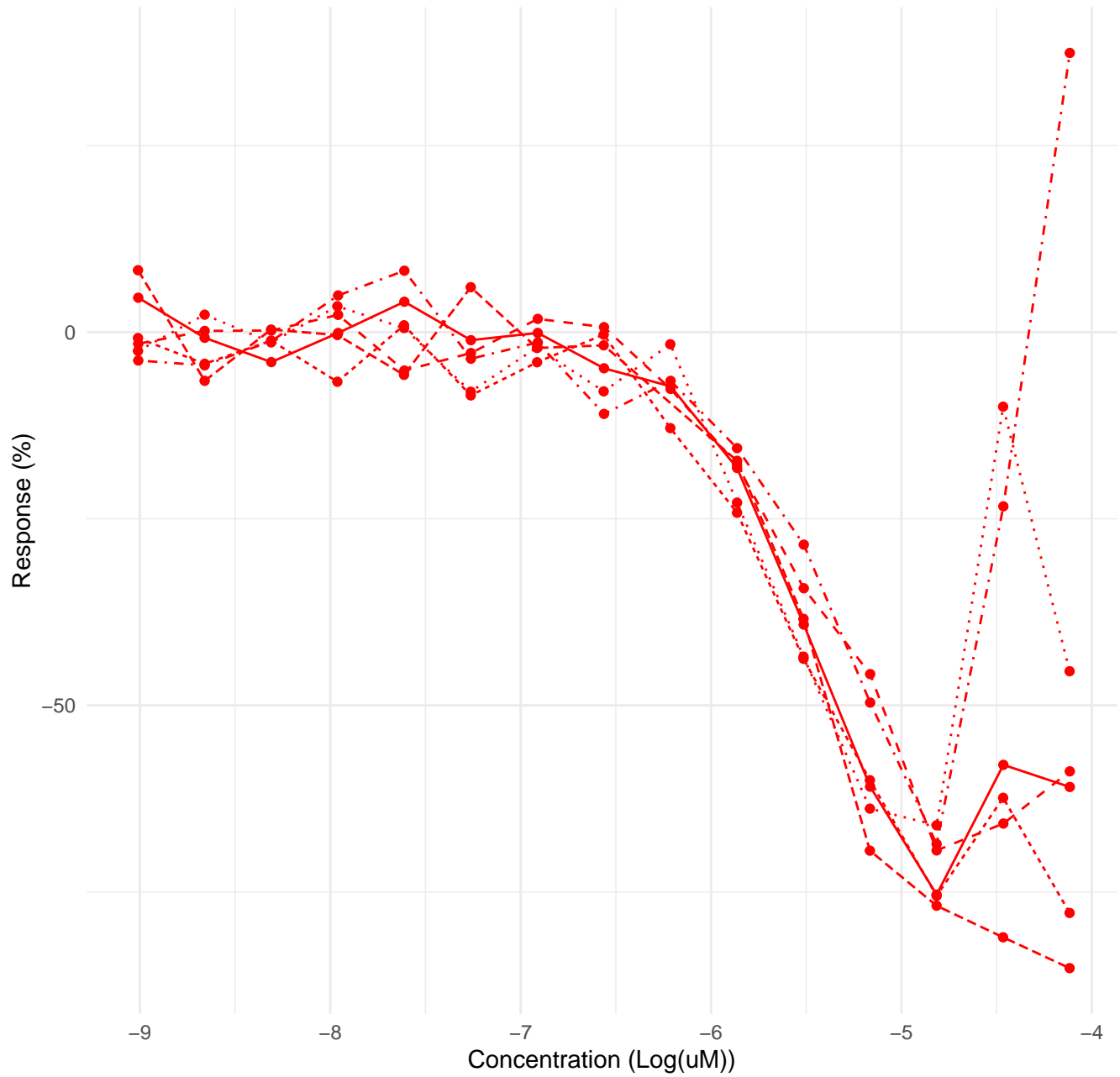

NOCAS\_47377

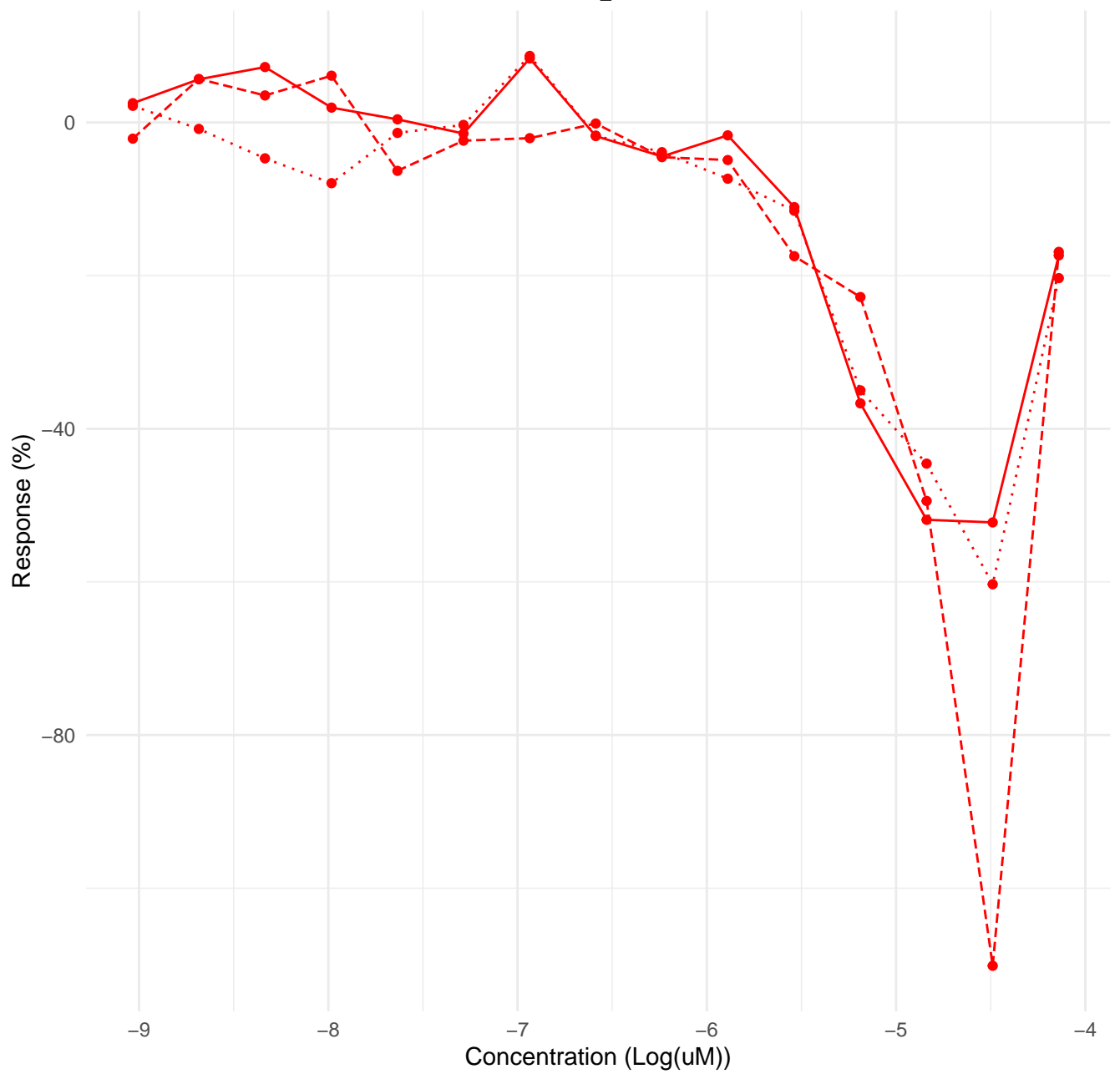

Supplement: Supplementary file 1 [file biology-11-00209-s001.zip › SupplementaryA.pdf]
